# 3‐Oxabicyclo[3.1.1]heptane as an Isostere of *meta*‐Benzene

**DOI:** 10.1002/anie.202505519

**Published:** 2025-05-22

**Authors:** Dmitry Dibchak, Pavel K. Mykhailiuk

**Affiliations:** ^1^ Enamine Ltd. Winston Churchill st. 78 Kyiv 02094 Ukraine

**Keywords:** 3‐Oxabicyclo[3.1.1]heptane, Bicyclo[3.1.1]heptane, Bioisosteres, Medicinal chemistry, *meta*‐Benzene

## Abstract

3‐Oxabicyclo[3.1.1]heptanes were designed as saturated isosteres of *meta*‐benzene. Crystallographic analysis revealed that these structures and *meta*‐benzene have identical geometric properties. Replacement of the central benzene ring in the anticancer drug *Sonidegib* with 3‐oxabicyclo[3.1.1]heptane provided a patent‐free analogue with a nanomolar potency, reduced lipophilicity, and improved water solubility (>500%).

## Graphical abstract

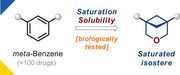

## Introduction

Benzene is the most popular ring in drugs and natural products.^[^ ^1^, ^2^, ^3^ ^]^ Since the introduction of the concept “Escape from Flatland” in 2009,^[^ ^4^ ^]^ medicinal chemists have started to look more in the direction of F(sp^3^)‐rich molecules. In particular, in 2012, scientists replaced the central benzene ring in a γ‐secretase inhibitor with bicyclo[1.1.1]pentane and obtained a bioactive compound with improved physicochemical properties.^[^ ^5^ ^]^ Since then, analogous replacements of benzene rings with saturated scaffolds have become popular in chemistry.^[^ ^6^, ^7^, ^8^ ^]^

**Scheme 1:**
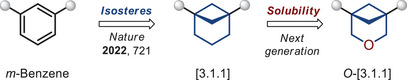
Bicyclo[3.1.1]heptanes as isosteres of *meta*‐benzenes. This work: 3‐oxabicyclo[3.1.1]heptanes as *meta*‐benzene isosteres.

In 2022, scientists showed that bicyclo[3.1.1]heptane could mimic the fragment of *meta*‐benzene in a biologically active compound.^[^ ^9^ ^]^ Both cores had a similar distance between substituents (4.8–5.0 Å), similar angles between exit vectors (119°–120°), and similar physicochemical properties (Scheme 1). As a result, bicyclo[3.1.1]heptane was proposed to be a saturated isostere of *meta*‐benzene.^[^ ^10^, ^11^, ^12^, ^13^, ^14^ ^]^

**Figure 1:**
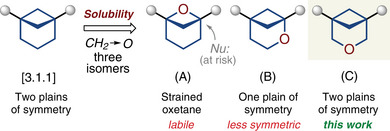
Design of isosteres of *meta*‐benzenes with improved solubility.

In this work, we have designed, synthesized, and biologically tested a new generation of *meta*‐benzene isosteres—3‐oxabicyclo[3.1.1]heptanes (Figure 1).^[^ ^15^ ^]^

## Results and Discussion

### Design

The methylene for oxygen replacement in saturated scaffolds leads to an improvement in solubility.^[^ ^16^, ^17^ ^]^ An analogous maneuver in bicyclo[3.1.1]heptane could potentially give three isomers: A, B, and C (Figure 1). Isomer A is a strained oxetane and therefore is expected to be chemically labile.^[^ ^18^ ^]^ Isomer B is less symmetric than the original core, as it only has one plane of symmetry.^[^ ^19^, ^20^ ^]^ Isomer C, on the other hand, should be a) chemically stable and b) has two plains of symmetry similar to the original bicyclo[3.1.1]heptane. In this context, we decided to focus our attention on the latter isomer—3‐oxabicyclo[3.1.1]heptane—as a potential *meta*‐benzene isostere.

### Synthesis

The scientific literature offers isolated reports for *poly*‐substituted 3‐oxabicyclo[3.1.1]heptanes,^[^ ^21^, ^22^, ^23^ ^]^ but there is no general and modular approach toward 3‐oxabicyclo[3.1.1]heptanes with only two substituents at the bridgehead positions of the core (to mimic *meta*‐substituted benzene).^[^ ^24^ ^]^

**Scheme 2:**
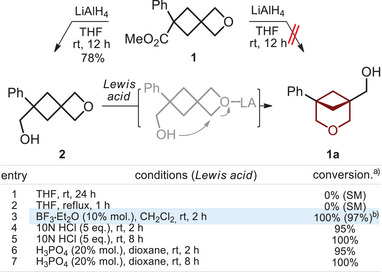
Attempted synthesis of compound **1a** from oxetane **1**. Isomerization of alcohol **2** into 3‐oxabicyclo[3.1.1]heptane **1a**. ^a)^Reaction conversion analyzed by ^1^H NMR. SM: starting material. ^b)^Isolated yield.

Previously, we showed that the reduction of spirocyclic oxetanyl nitriles with LiAlH_4_ led to the formation of 3‐azabicyclo[3.1.1]heptanes.^[^ ^25^ ^]^ Here, we envisioned that the alternative reaction would also work for the corresponding esters. The reduction of ester **1** with LiAlH_4_ gave, however, only alcohol **2** (Scheme 2). The formation of the desired 3‐oxabicyclo[3.1.1]heptane **1a** was not observed.

Alcohol **2** did not isomerize into compound **1a** at room temperature (Scheme 2, entry 1) or under heating (entry 2). Therefore, we tried next the isomerization under the Lewis acid catalysis. Indeed, the isomerization proceeded smoothly with BF_3_•Et_2_O, aqueous HCl, and aqueous H_3_PO_4_ at room temperature (entries 3–7) leading to the formation of the desired compound **1a**. In the experiment with BF_3_•Et_2_O, product **1a** was isolated in 97% yield.

### Scalable Synthesis

**Scheme 3:**
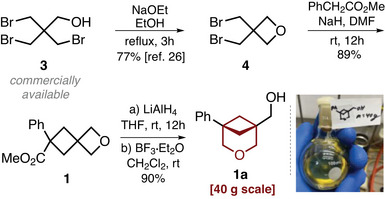
Scalable synthesis of compound **1a**.

Having developed conditions for the isomerization of alcohol **2**, we next elaborated the multigram scale synthesis of compound **1a**. The optimized procedure commenced from the commercially available bromide **3** (Scheme 3). Treatment of the latter with NaOEt in ethanol under reflux led to the formation of oxetane **4** following the literature protocol.^[^ ^26^ ^]^ Alkylation of PhCH_2_CO_2_Me with bromide **4** in the presence of sodium hydride in DMF cleanly proceeded at room temperature to provide spirocyclic compound **1** with an 89% yield. Reduction of the ester group with LiAlH_4_ provided alcohol **2** with ca. 90% purity. To ensure a higher overall yield of the synthesis, we did not purify product **2** at this point but used the crude material directly in the subsequent isomerization step with BF_3_•Et_2_O in dichloromethane. As a result, the desired product **1a** was obtained in 90% yield over two steps from oxetane **1**. This optimized sequence allowed us to prepare 40 g of the target 3‐oxabicyclo[3.1.1]heptane **1a** in one run (Scheme 3).

### Scope

**Scheme 4:**
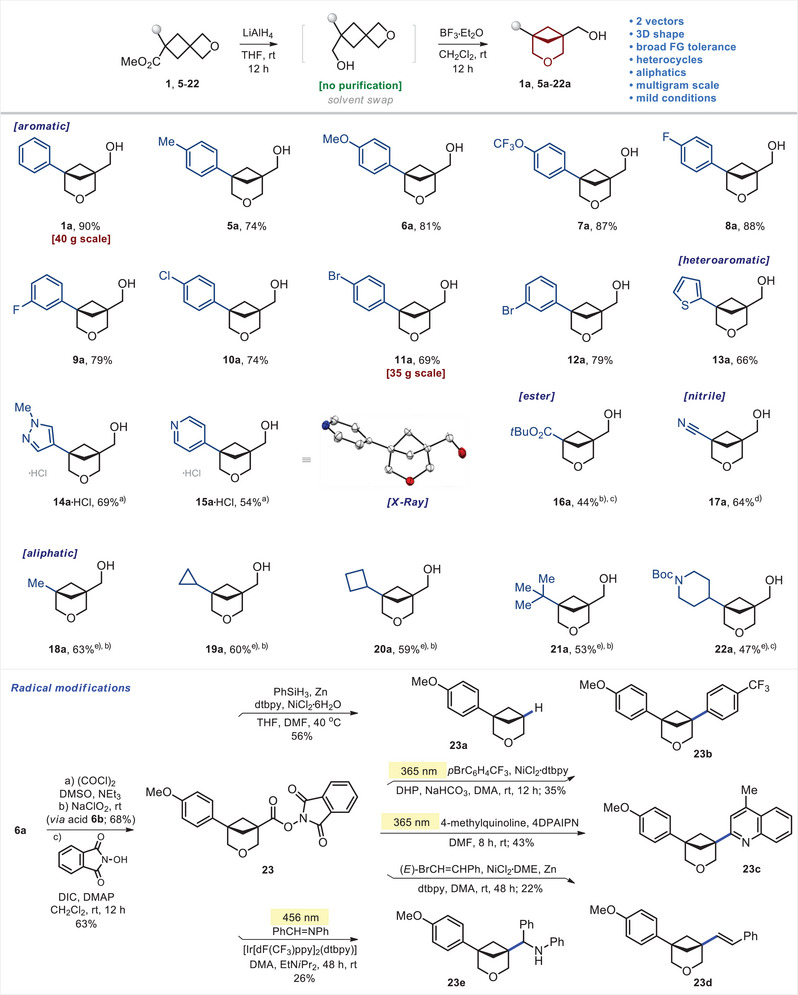
Synthesis and radical modifications of 3‐oxabicyclo[3.1.1]heptanes. ^a)^Products were isolated as hydrochloride salts. 10 M HCl was used instead of BF_3_•Et_2_O at the cyclization step. ^b)^Stepwise combination of DIBAL and NaBH_4_ was used instead of LiAlH_4_. ^c)^H_3_PO_4_ in dioxane was used instead of BF_3_•Et_2_O. ^d)^NaBH_4_ was used instead of LiAlH_4_. ^e)^Starting materials **18**–**22** had the nitrile group (─CN) instead of the ester group (─CO_2_Me). X‐ray crystal structure of compound **15a**•HCl (carbon: grey, oxygen: red, nitrogen: blue). Ellipsoids are shown at a 30% probability level. Hydrogen and chlorine atoms are omitted for clarity. 4DPAIPN: 2,4,5‐tris(diphenylamino)isophthalonitrile. Dtbpy: 4,4′‐di‐tert‐butyl‐2,2′‐bipyridine. DHP: 1,4‐dihydropyridine Hantzsch ester. DME: dimethoxyethane. DMA: dimethylacetamide. DMF: dimethylformamide.

Moving forward, we studied the generality of the developed method. The reduction–isomerization sequence tolerated various substituents at the aromatic core (Scheme 4). Among them were the alkyl group (**5a**), methoxy group (**6a**), trifluoromethoxy group (**7a**), fluorine (**8a**, **9a**), chlorine (**10a**), and bromine atoms (**11a**, **12a**). The reaction was also compatible with the medicinal chemistry relevant heterocyclic cores: thiophene (**13a**), pyrazole (**14a**), and pyridine (**15a**). Even the ─CO_2_ *t*Bu (**16a**) and the ─CN (**17a**) groups performed well in the reaction; however, in this case, the reduction step was realized with diisobutylaluminum hydride (DIBAL)/NaBH_4_ (**16a**) and NaBH_4_ (**17a**), correspondingly.

Aliphatic substituents (**18a**–**21a**) could also be used in this strategy. The corresponding starting materials contained, however, the nitrile group (─CN) instead of the ester group (─CO_2_Me), and the reaction step was undertaken with the consecutive combination of DIBAL and NaBH_4_. Following this modified tactic, the *N*‐Boc piperidine‐containing linker **22a** was obtained in 47% yield. Most compounds were isolated as free NH‐amines; however, in some cases (**14a**, **15a**), we converted them into crystalline hydrochloride salts. All syntheses were performed on milligram and gram scales, but products **1a** and **11a** were also synthesized in 35–40 g amounts. The structure of compound **15a**•HCl was confirmed by X‐ray analysis (Scheme 4).^[^ ^27^ ^]^

Radical modifications of the 3‐oxabicyclo[3.1.1]heptane core at the bridgehead position were also possible (Scheme 4). Oxidation of alcohol **6a** gave carboxylic acid **6b** in 68% yield. The reaction of the latter with *N*‐hydroxyphtalimide in the presence of *N,N′*‐diisopropylcarbodiimide (DIC)/4‐dimethylaminopyridine (DMAP) in dichloromethane at room temperature provided redox‐active ester **23**.^[^ ^28^, ^29^ ^]^ The [Ni]‐catalyzed decarboxylative reduction of the latter with PhSiH_3_/Zn gave product **23a** in 56% yield. The photochemical [Ni]‐catalyzed C(sp^3^)–C(sp^2^) cross‐coupling of the redox‐active ester **23** with *p*BrC_6_H_4_CF_3_ in the presence of the Hantzsch ester provided product **23b** in 24% yield. The photochemical Minisci‐type reaction of ester **23** with 4‐methylquinoline gave compound **23c**. The [Ni]‐catalyzed coupling of **23** with BrCH═CHPh in the presence of zinc resulted in the formation of alkene **23d**. Finally, the reaction of **23** with PhCH═NPh under the reductive “photo‐redox” conditions—EtN*i*Pr_2_, [Ir], 456 nm—gave amine **23e**.

### Modifications

Modifications of some representative 3‐oxabicyclo[3.1.1]heptanes into the building blocks (compounds with one or two functional groups) for medicinal chemistry were undertaken in the next step.

**Scheme 5:**
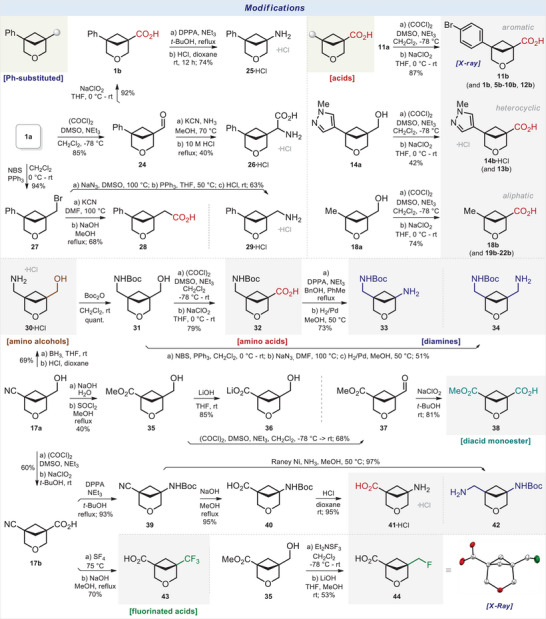
Synthesis of functionalized 3‐oxabicyclo[3.1.1]heptanes for medicinal chemistry. X‐ray crystal structure of compound **44** (carbon: white, oxygen: red, fluorine: green). Hydrogen and chlorine atoms are omitted for clarity. Ellipsoids are shown at a 30% probability level. DPPA: diphenylphosphoryl azide. NBS: *N*‐bromosuccinimide.

The standard Swern oxidation of alcohol **1a** gave aldehyde **24** (Scheme 5). The subsequent oxidation of the latter with NaClO_2_ provided the carboxylic acid **1b**. The Curtius reaction of **1a** followed by the acidic *N*‐Boc deprotection resulted in the formation of amine **25**•HCl. The Strecker reaction of aldehyde **24** afforded α‐amino acid **26**. The Appel reaction of alcohol **1a** with *N‐*bromosuccinimide (NBS) and PPh_3_ smoothly provided alkyl bromide **27**. The reaction of the latter with KCN in DMF under heating and the subsequent alkali hydrolysis of the nitrile group provided carboxylic acid **28**. The reaction of bromide **27** with NaN_3_ followed by the Staudinger reaction of the intermediate azide led to the formation of amine **29**.

The stepwise oxidation of alcohol **11a** using the Swern protocol and sodium chlorite provided carboxylic acid **11b**. Following this tactic, aromatic carboxylic acids **5b**–**10b**, **12b**; heterocyclic carboxylic acids **13b**, **14b**; and even aliphatic carboxylic acids **18b**–**22b** were also easily synthesized from the corresponding alcohols (Scheme 5).

Reduction of the nitrile group in compound **17a** with the BH_3_•THF complex afforded amino alcohol **30** (Scheme 5). The *N*‐Boc protection (via **31**) and the subsequent full oxidation of the alcohol group gave *N*‐Boc amino acid **32**. The Curtius reaction of the latter and the *N*‐Cbz cleavage with H_2_/Pd in methanol gave *N*‐Boc diamine **33**. The Appel reaction of alcohol **31** with NBS/PPh_3_, treatment with sodium azide in DMF under heating, and the reduction of the intermediate nitrile provided *N*‐Boc diamine **34**.

Alkali hydrolysis of the nitrile group in compound **17a** and the subsequent acidic esterification gave methyl ester **35** (Scheme 5). Hydrolysis of the ester group afforded the hydroxy acid **36**. The Swern oxidation of the alcohol group in **35** gave aldehyde **37**. The subsequent oxidation of the latter with sodium chlorite gave monoester **38**.

Oxidation of the alcohol group in compound **17a** gave carboxylic acid **17b** in 60% yield. The Curtius reaction and the hydrolysis of the nitrile group in the formed compound **39** gave an interesting *N*‐Boc amino acid **40**. The acidic *N*‐Boc deprotection provided amino acid **41**•HCl. In addition, the reduction of the nitrile group in **40** with Raney nickel resulted in the formation of *N*‐Boc diamine **42**.

The reaction of carboxylic acid **17b** with sulfur tetrafluoride and the alkali hydrolysis of the nitrile group gave the CF_3_‐substituted carboxylic acid **43**.^[^ ^30^ ^]^ The reaction of alcohol **35** with Et_2_NSF_3_ in dichloromethane at room temperature followed by the hydrolysis of the ester group provided the F‐substituted carboxylic acid **44**.

### Stability

Because of the strained structure of all 3‐oxabicyclo[3.1.1]heptanes (Schemes 4 and 5), we also monitored the stability of the obtained products. All compounds were kept on the shelf at room temperature in closed vials. Their regular ^1^H NMR and LC‐MS inspection every three months revealed no detectable decomposition in any of them after at least six months of storage.

In addition, we checked the preliminary thermal stability of two representative 3‐oxabicyclo[3.1.1]heptanes: carboxylic acid **11b** and *N*‐Boc amino acid **40**. The compounds indeed remained stable under heating at 100 °C for 5 min, as monitored by ^1^H NMR and LC‐MS (see Supporting Information pp. S52–S53).

### Steric Volume

**Figure 2:**
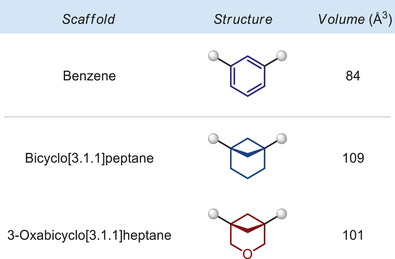
Calculated molecular volume (Å^3^) of benzene, bicyclo[3.1.1]heptane, and 3‐oxabicyclo[3.1.1]heptane.

To estimate the size of the 3‐oxabicyclo[3.1.1]heptane scaffold compared to the benzene ring, we calculated^[^ ^31^ ^]^ and compared their steric volumes (Figure 2). The bicyclo[3.1.1]heptane scaffold was also included for comparison, representing an already established isosteric benzene replacement.^[^ ^9^ ^]^

The obtained data show that the volume of the bicyclo[3.1.1]heptane scaffold is bigger than that of the benzene ring: 109 Å^3^ (311) vs. 84 Å^3^ (benzene). At the same time, the replacement of the methylene group for the oxygen atom led to a significant reduction of the volume, making the formed 3‐oxabicyclo[3.1.1]heptane core more similar to the benzene ring: 101 Å^3^ (O‐311) vs. 84 Å^3^ (benzene).

### Crystallographic Analysis

**Figure 3:**
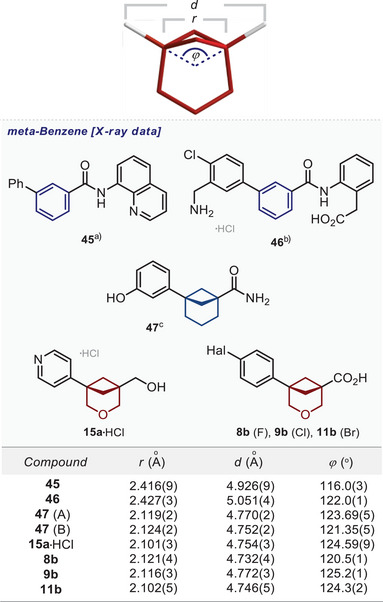
Definition of distances *r*, *d*, and angle *γ* (the 3‐oxabicyclo[3.1.1]heptane core is shown as an example). Geometric parameters *r*, *d*, and *γ* for the *meta*‐substituted benzene ring (**45**, **46**); bicyclo[3.1.1]heptane **47**; and 3‐oxabicyclo[3.1.1]heptanes **15a**•HCl, **8b**, **9b**, **11b**. ^a)^The data is taken from Ref. [32]. ^b)^The data is taken from Ref. [33]. ^c)^The data is taken from Ref. [9].

Next, we compared the geometric properties of 3‐oxabicyclo[3.1.1]heptanes with those of *meta*‐benzenes. We also included the bicyclo[3.1.1]heptane scaffold for comparison.^[^ ^9^ ^]^ For that, we measured two C─C distances, *r* and *d*, to see the overall similarity of cores and angle *φ* between two exit vectors to see the similarity of angular models (Figure 3).

We obtained the values of *r*, *d*, and *φ* of 3‐oxabicyclo[3.1.1]heptanes from the X‐ray data of compounds **15a**•HCl, **8b**, **9b**, and **11b**. The corresponding parameters for the *meta*‐substituted benzenes **45**,^[^ ^32^ ^]^ **46**,^[^ ^33^ ^]^ and bicyclo[3.1.1]heptane **47** ^[^ ^9^ ^]^ were calculated from the X‐ray data published in the literature.

The distance *r* in 3‐oxabicyclo[3.1.1]heptanes was ca. 0.3 Å shorter than that in *meta*‐benzene: 2.10–2.12 Å vs. 2.42–2.43 Å (*meta*‐benzene). The distance *d* between substituents in 3‐oxabicyclo[3.1.1]heptanes was also ca. 0.2 Å shorter than that in *meta*‐benzene: 4.75–4.77 Å vs. 4.93–5.05 Å (*meta*‐benzene). However, angle *φ* was similar in both scaffolds and was very close to the ideal value of 120°: 120°–124° vs. 116°–122° (*meta*‐benzene). It must also be noted that both saturated scaffolds—bicyclo[3.1.1]heptane and 3‐oxabicyclo[3.1.1]heptane—were almost identical according to the measured parameters.

**Figure 4:**
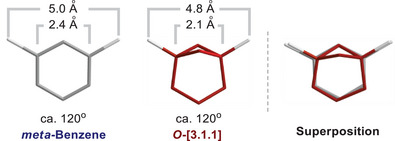
A visual comparison of *meta*‐substituted benzene and 3‐oxabicyclo[3.1.1]heptane.

In short, the 3‐oxabicyclo[3.1.1]heptane core resembled closely the *meta*‐benzene ring, as the geometric characteristics *r*, *d*, and *φ* remained similar. The superposition of both scaffolds visualizes their excellent overlap (Figure 4).

### The Acidity of Functional Groups

**Figure 5:**
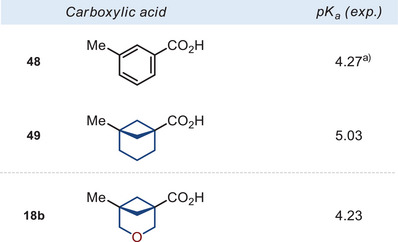
Experimental p*K*_a_ values of carboxylic acids **49** and **18b**. ^a)^For compound **48**, the data is taken from Ref. [34].

We also studied the influence of the replacement of the methylene group for the oxygen atom in the bicyclo[3.1.1]heptane skeleton on the electronic properties. For that, we experimentally measured p*K*_a_ values of the bicyclo[3.1.1]heptane carboxylic acid **49**, the 3‐oxabicyclo[3.1.1]heptane carboxylic acid **18b**, and *meta*‐methyl benzoic acid (**48**) as a reference (Figure 5).^[^ ^34^ ^]^

It is important to mention that the acidity of *meta*‐methyl benzoic acid (**48**) and 3‐oxabicyclo[3.1.1]heptane **18b** were almost identical (Figure 5). Indeed, the replacement of the benzene ring with the saturated bicyclo[3.1.1]heptane core reduced the acidity by ca. 0.7 p*K*_a_ units. However, incorporation of the oxygen atom into the latter almost ideally restored it: p*K*_a_ = 4.3 (**48**) vs. 5.0 (**49**) vs. 4.2 (**18b**). Because the acidity/basicity of functional groups is often responsible for the potency, selectivity, and toxicity of bioactive compounds,^[^ ^35^ ^]^ the fine‐tuning of the p*K*_a_ value by replacing the benzene ring with 3‐oxabicyclo[3.1.1]heptane could be a useful solution.

### Incorporation into a Drug

**Figure 6:**
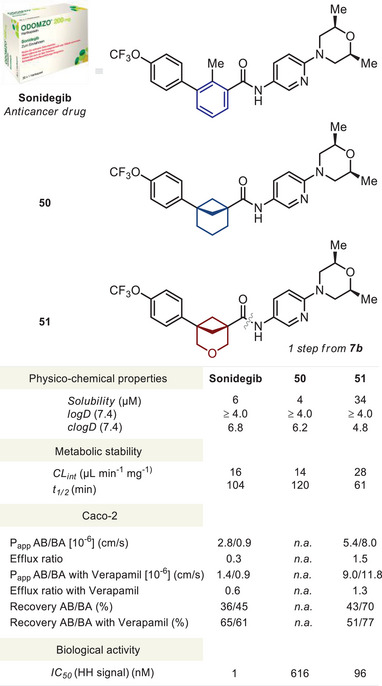
Properties of *Sonidegib* and its saturated analogues **50** and **51**. Solubility: the experimental kinetic solubility in phosphate‐buffered saline, pH 7.4 (µM). logD (7.4): the experimental distribution coefficient in *n*‐octanol/phosphate‐buffered saline, pH 7.4. Reliable logD values could be obtained within a range of 1.0–4.0. clogD (7.4): the calculated lipophilicity at pH 7.4. CL_int_: the experimental metabolic stability in human liver microsomes (µL min^−1^ mg^−1^). *t*_1/2_ (min): the experimental half‐time of a metabolic decomposition. The experimental permeability of *Sonidegib* and saturated analogues **50** and **51** in the bidirectional Caco‐2 assay. The experimental inhibition, IC_50_ (nM), of the Hedgehog signaling pathway by *Sonidegib* and saturated analogues **50** and **51** in the Gli reporter NIH3T3 cell line.

Having developed a general practical approach toward 3‐oxabicyclo[3.1.1]heptanes (Scheme 4) and their functionalized derivatives (Scheme 5), we wanted to study the effects of the replacement of *meta*‐benzene on physicochemical and biological properties of bioactive compounds. Toward this goal, we synthesized the 3‐oxabicyclo[3.1.1]heptane analogue **51** of the FDA‐approved anticancer drug *Sonidegib* from carboxylic acid **7b** (for details, please see Supporting Information, p. S50). For comparison, we also included the previously reported bicyclo[3.1.1]heptane‐containing compound **50** (Figure 6).^[^ ^9^ ^]^

### Physicochemical Properties

First, we studied experimental physicochemical properties—water solubility, lipophilicity (see Supporting Information pp. S241–S249), and metabolic stability (see Supporting Information pp. S250–256)—of *Sonidegib* and its saturated analogues **50** and **51**.

Replacement of the benzene ring in *Sonidegib* with bicyclo[3.1.1]heptane slighly decreased the water solubility: 6 µM (*Sonidegib*) vs. 4 µM (**50**). At the same time, the analogous replacement with 3‐oxabicyclo[3.1.1]heptane led to a significant fivefold increase in the solubility: 6 µM (*Sonidegib*) vs. 34 µM (**51**).

An effect of the replacement of the benzene ring in *Sonidegib* with saturated scaffolds on the experimental lipophilicity (logD, 7.4) was not observed due to too high lipophilicity of all three compounds, outside of the sensitivity range of the experimental method. Therefore, we used the calculated lipophilicity index (clogD, 7.4).^[^ ^36^ ^]^ Replacement of the benzene ring with bicyclo[3.1.1]heptane slightly decreased clogD: 6.8 (*Sonidegib*) vs. 6.2 (**50**). However, the effect of such replacement with the 3‐oxabicyclo[3.1.1]heptane core was more than significant; the сlogD index decreased by two units: 6.8 (*Sonidegib*) vs. 4.8 (**51**).

The replacement of the benzene ring in *Sonidegib* with saturated scaffolds either preserved the metabolic stability or slightly reduced it: CL_int_ (µL min^−1^ mg^−1^) = 16 (*Sonidegib*) vs. 14 (**50**) vs. 28 (**51**).

In summary, the replacement of the benzene ring in *Sonidegib* with 3‐oxabicyclo[3.1.1]heptane (**51**) led to a slight decrease in metabolic stability (within a normal range), a notable reduction of lipophilicity, and a significant improvement of solubility (>500%).

### Biological Activity

Finally, we wanted to answer the key question—could 3‐oxabicyclo[3.1.1]heptane indeed mimic *meta*‐benzene in bioactive compounds? Toward this goal, we measured the biological activity of the marketed drug *Sonidegib* and its saturated analogues **50** and **51**.

The mechanism of action of the anticancer drug *Sonidegib* involves inhibiting the Hedgehog signaling pathway. Thus, we measured the inhibition of this signaling pathway caused by *Sonidegib* ^[^ ^37^ ^]^ vs. its saturated analogues **50** and **51** in Gli‐Luc reporter NIH3T3 cells (Figure 6; see also Supporting Information, pp. S263–S265). On one hand, the *O*‐[3.1.1]‐containing analogue **51** was two orders of magnitude less potent than the original drug. On the other hand, the patent‐free compound **51** demonstrated a level of inhibition in a nanomolar range and was six times more active than the known saturated analogue **50**, IC_50_ (nM): 6 (*Sonidegib*) vs. 616 (**50**) vs. 96 (**51**) (Figure 6).

### Conclusions

In 2022, bicyclo[3.1.1]heptanes were proposed to mimic *meta*‐benzenes in biologically active compounds.^[^ ^9^ ^]^ In this work, we designed, synthesized, and tested biologically a new generation of saturated isosteres of *meta*‐benzene—3‐oxabicyclo[3.1.1]heptanes. The key synthesis step was an intramolecular Lewis‐acid‐catalyzed ring opening of spirocyclic oxetanes. The patent‐free analogue **51** of the anticancer drug *Sonidehib* had a nanomolar potency, reduced lipophilicity, and improved solubility (>500%) compared to the original drug.

We expect that after this work, the 3‐oxabicyclo[3.1.1]heptane core will find immediate application in medicinal chemistry and agrochemistry as a promising bioisostere of *meta*‐benzene.

## Conflict of Interests

D.D. and P.K.M. are employees of a chemical supplier, Enamine.

## Supporting information



Supporting Information

Supporting Information

## Data Availability

The data that support the findings of this study are available in the Supporting Information of this article.
