# Supplementary material for: 3‐Oxabicyclo[3.1.1]heptane as an Isostere of meta‐Benzene
Source: Angew Chem Int Ed Engl. 2025 May 22;64(25):e202505519. doi: 10.1002/anie.202505519 (PMC12171319; doi:10.1002/anie.202505519)
Supplement: Supplementary file 1 — Supporting Information [file ANIE-64-e202505519-s002.pdf]

Supporting Information

3-Oxabicyclo[3.1.1]heptane as an Isostere of *meta*-Benzene

Dmitry Dibchak, Pavel K. Mykhailiuk*

Enamine Ltd; Winston Churchill st. 78, 02094 Kyiv (Ukraine)

Table of contents

1. General Considerations S4
2. List of abbreviations S8
3. Experimental Section. Data description and procedures S9
3.1. Synthesis of starting compounds S9
3.2. Synthesis of alcohols 2, 1a and 5a-22a S14
3.3. Synthesis of carboxylic acids S25
3.4. Radical modifications S34
3.5. Modifications S38
3.6. Synthesis of analogs of Sonidegib S50
4. Stability test S52
5. Copies of ^{1}H, $^{13}C\{^{1}H\}$ and $^{19}F\{^{1}H\}$ NMR spectra S54
Compound 4 .... S54
Compound 1 .... S56
Compound 2 .... S58
Compound 6 .... S60
Compound 7 .... S62
Compound 9 .... S65
Compound 13 .... S69
Compound 15 .... S71
Compound 18 .... S73
Compound 19 .... S75
Compound 20 .... S77
Compound 22 .... S79
Compound 1a S81
Compound 5a S83
Compound 6a S85
Compound 7a S87

Compound 8a.......... S90
Compound 9a.......... S93
Compound 10a.......... S96
Compound 11a.......... S98
Compound 12a.......... S100
Compound 13a.......... S102
Compound 14a.......... S104
Compound 15a.......... S106
Compound 16a.......... S108
Compound 17a.......... S110
Compound 18a.......... S112
Compound 19a.......... S114
Compound 20a.......... S116
Compound 21a.......... S118
Compound 22a.......... S120
Compound 1b S122
Compound 5b S124
Compound 6b S126
Compound 7b S128
Compound 8b S131
Compound 9b S134
Compound 10b S138
Compound 11b S140
Compound 12b S143
Compound 13b S145
Compound 14b S147
Compound 17b S149
Compound 18b S151
Compound 19b S154
Compound 20b S156
Compound 21b S158
Compound 22b S160
Compound 23 .... S162
Compound 23a.......... S164
Compound 23b S166

Compound 23c S169
Compound 23d S171
Compound 23e S173
Compound 24 .... S175
Compound 25 .... S177
Compound 26 .... S179
Compound 27 .... S181
Compound 28 .... S183
Compound 29 .... S185
Compound 30 .... S187
Compound 31 .... S189
Compound 32 .... S191
Compound 33 .... S193
Compound 34 .... S197
Compound 35 .... S199
Compound 36 .... S201
Compound 37 .... S203
Compound 38 .... S205
Compound 39 .... S207
Compound 40 .... S209
Compound 41 .... S211
Compound 42 .... S213
Compound 43 .... S215
Compound 44 .... S218
Compound 51 .... S221
6. Crystallographic Data (X-ray) S224
X-Ray Structure Determinations of 15a, 8b, 10b, 11b, and 44 .... S224
7. Analysis of Aqueous Solubility S239
8. Determination of Distribution Coefficient (LogD, pH 7.4) S245
9. Metabolic Stability in Human Liver Microsomes S248
10. Assessment of Caco-2 Permeability of Sonidegib and its Saturated Analog 51 .... S255
11. Analysis of novel Hedgehog signaling pathway inhibitors in the cell-based Gli-Luc reporter system S261
12. References S264

1. General Considerations

Unless otherwise noted, all reactions were carried out under an atmosphere of argon in oven-dried glassware. All chemicals were provided by Enamine Ltd. (www.enamine.net). All solvents were commercially supplied. DMF was dried over 4 Å molecular sieves (activated at 300 °C under vacuum for 24 h) for at least 48 h, then stored under argon. Alternatively, distilled under reduced pressure over P_2O_5 and stored over molecular sieves under an inert atmosphere. DMSO was dried over 4 Å molecular sieves (activated at 300 °C under vacuum for 24 h) for at least 48 h, then stored under argon. Alternatively, dried by stirring over CaH_2 for 24 h, followed by distillation under reduced pressure and storage under an inert atmosphere. CH_2Cl_2 was distilled over $CaCl_2$ under Ar. THF was distilled over sodium/benzophenone under an argon atmosphere and additionally distilled over $LiAlH_4$ and stored over activated 4 Å molecular sieves. The water content of all dried solvents was checked by Karl Fischer titration as part of quality control by our analytical department to ensure minimal moisture levels. All solvents were degassed before use when necessary.

All reactions were monitored by thin-layer chromatography (TLC) and were visualized using UV light or TLC stains. Product purification was performed using silica gel column chromatography. TLC characterization was performed with pre-coated silica gel GF254 (0.2 mm), while column chromatography characterization was performed with silica gel (100-200 mesh).

^{1}H-NMR spectra were recorded at 400, 500, or 600 MHz (Varian); ^{19}F-NMR spectra were recorded at 376 MHz (Varian), ^{13}C NMR spectra were recorded at 101, 126, or 151 MHz (Varian). ^{1}H-NMR chemical shifts are calibrated using residual undeuterated solvents $CHCl_3$ (δ = 7.26 ppm), DMSO (δ = 2.50 ppm) or H_2O (δ = 4.79 ppm). ^{13}C-NMR chemical shifts for ^{13}C-NMR are reported relative to the central signal $CHCl_3$ (δ = 77.16 ppm) or DMSO (δ = 39.52 ppm). Coupling constants are given in Hz. Samples were typically 20 mg dissolved in about 500 µL of the suitable deuterated solvent.

The analytical laboratory determined the melting points and melting ranges of chemical substances using the MPA100 Melting Point Apparatus. The determination was performed using the capillary method, where the sample was placed in a glass capillary and heated at a controlled rate until it melted. The MPA100 was equipped with a microprocessor-controlled temperature system and a built-in digital camera. It offered programmable heating rates ranging from 0.1 °C/min to 20 °C/min, in 0.1 °C/min increments, providing flexibility in measurement. The operator selected the appropriate heating rate based on the specific requirements of the task. Both camera recordings and visual observations were used, as needed, to accurately determine the melting temperature.

High-resolution mass spectra (HRMS) were recorded on an Agilent LC/MSD TOF mass spectrometer by electrospray ionization time of flight reflectron experiments. Liquid chromatography-mass spectrometry (LC-MS) was done with chemical ionization (CI). LC-MS is

the most common method for the qualitative and quantitative analysis of sample purity. It is particularly effective for compounds containing chromophores. In the production of screening compounds, LC-MS serves as the primary method for analyzing synthesis products (Table S1). Instrument specifications: Agilent 1100 Series LC/MSD system with DAD\ELSD Alltech 2000ES and Agilent LC\MSD VL (G1956B), SL (G1956B) mass-spectrometer; Agilent 1200 Series LC/MSD system with DAD\ELSD Alltech 3300 and Agilent LC\MSD G6130A, G6120B mass-spectrometer; Agilent Technologies 1260 Infinity LC/MSD system with DAD\ELSD Alltech 3300 and Agilent; LC\MSD G6120B mass-spectrometer; Agilent Technologies 1260 Infinity II LC/MSD system with DAD\ELSD G7102A 1290 Infinity II and Agilent LC\MSD G6120B mass-spectrometer; Agilent 1260 Series LC/MSD system with DAD\ELSD and Agilent LC\MSD (G6120B) mass-spectrometer; UHPLC Agilent 1290 Series LC/MSD system with DAD\ELSD and Agilent LC\MSD (G6125B) mass-spectrometer. All the LC/MS data were obtained using positive/negative mode switching. Preparation of the sample: the selected and prepared aliquot is carefully measured and then diluted with solvents classified as "for HPLC" (such as DMSO, CH_3CN, MeOH, H_2O). The sample concentration is adjusted to 1 mg of substance per 0.5 mL of solution, with a small tolerance in concentration (2-3 mg/mL). For reaction mixtures containing solvents like DMF, DMA, DMSO, or toluene, it is recommended to use DMSO as the dilution solvent to ensure consistent results.

Table S1. General information about methods

**Method**	**General Parameters**	**Binary Pump**	**Application**
SUPOR	**Temperature**: 60 °C<br>**Injection volume**: 0.5 μL or 2 μL<br>**DAD**: 215 nm, 254 nm, 280 nm<br>**Scan range**: *m/z* = 83 – 600 (1000)<br>**Ionization mode:** *Electrospray ionization* (ESI), POS/NEG<br>**Column:** Agilent Poroshell 120 SB-C18 4.6 × 30 mm 2.7 μm with UHPLC Guard Infinity<br>Lab Poroshell 120 SB-C18 4.6 × 5 mm 2.7 μm<br>**ELSD Gas flow** = 3 L/min; T = 40 °C	**Flow** = 3 mL/min<br>Solv. A: 95%AcN + 5%H_2O (0.1% FA)<br>Solv. B: H_2O (0.1% FA)<br>**Stoptime**: 1.95 (2.47) min.<br>T, min A% B%<br>0.00 1 99<br>0.01 1 99<br>1.5 100 0<br>1.73(2.2) 100 0	Screening of all compounds. Analysis of poorly retained substances with mass greater than 600 *m/z.* Analysis of reaction mixtures.
6 min_4-6 × 30_1-5	**Temperature**: 60 °C<br>**Flow rate**: 1.5 mL/min<br>**Injection volume**: 0.5 μLor 2 μL<br>**DAD**: 215 nm, 254 nm, 280 nm<br>**Scan range**: *m/z* = 83 – 600<br>**Ionization mode:** *Electrospray*	**Flow** = 1.5 mL/min<br>Solv. A: 95%AcN + 5%H_2O (0.1% FA)<br>Solv. B: H_2O (0.1% FA)<br>**Stoptime:** 6.00 min.<br>T, min A% B%	Analysis for incompletely separated substances

	ionization (ESI), POS/NEG<br>**Column:** Agilent Poroshell 120 SB-C18<br>4.6 × 30 mm 2.7 µm with UHPLC Guard<br>Infinity<br>Lab Poroshell 120 SB-C18 4.6 × 5 mm 2.7 µm<br>**ELSD Gas flow** = 3 L/min; T = 40 °C	0.00 1 99<br>0.01 1 99<br>5.00 100 0<br>5.99 100 0	
N_SUPOR	**Temperature**: 60 °C<br>**Injection volume**: 0.5 µL or 2 µL<br>**DAD**: 215 nm, 254 nm, 280 nm<br>**Scan range**: *m/z* = 83 – 600<br>**Ionization mode:** *Electrospray ionization* (ESI), POS/NEG<br>**Column:** Agilent Poroshell 120 SB-C18<br>4.6 × 30 mm 2.7 µm with UHPLC Guard<br>Infinity<br>Lab Poroshell 120 SB-C18 4.6 × 5 mm 2.7 µm<br>**ELSD Gas flow** = 3 L/min; T = 40 °C	**Flow** = 3 mL/min<br>Solv. A: 95%AcN+5%H_2O<br>Solv. B: H_2O<br>**Stoptime:** 1.95 min.<br>T, min A% B%<br>0.00 1 99<br>0.01 1 99<br>1.5 100 0<br>1.73 100 0	Analysis of substances that are not stable in an acidic environment. Substances for which a neutral phase of chromatography is recommended. For substances that come out with a front in an acidic environment.
AC-GEMINI	**Temperature**: 25 °C<br>**Injection volume**: 0.5 µL or 2 µL<br>**DAD**: 215 nm, 254 nm, 280 nm<br>**Scan range**: *m/z* = 83 – 600<br>**Ionization mode:** *Electrospray ionization* (ESI), POS/NEG<br>**Column:** GEMINI NX-C18 4.6 × 50 mm 5 Micron with<br>Phenomenex Security Guard GEMINI NX-C18<br>4 × 3.0 mm 5 µm<br>**ELSD Gas flow** = 1.6 L/min; T = 64 °C	**Flow** = 2.3 mL/min<br>Solv. A: AcN<br>Solv. B: 10 mM NH_4Ac,<br>pH = 10<br>**Stoptime**: 4.1 min.<br>T, min A% B%<br>0.00 0 100<br>0.03 0 100<br>3.00 100 0<br>3.70 100 0	Analysis of substances for which the alkaline phase of chromatography is recommended.<br>Analysis of substances that in an acidic environment come out with the front.
POR1K	**Temperature**: 60 °C<br>**Injection volume**: 0.5 µL or 2 µL<br>**DAD**: 215 nm, 254 nm, 280 nm<br>**Scan range**: *m/z* = 83 – 1000<br>**Ionization mode:** *Electrospray ionization* (ESI), POS/NEG<br>**Column:** Agilent Poroshell 120 SB-C184.6 × 30 mm 2.7 µm with<br>UHPLC Guard InfinityLab Poroshell 120 SB-C18 4.6 × 5 mm 2.7 µm<br>**ELSD Gas flow** = 3 L/min; T = 40 °C	**Flow** = 3 mL/min<br>Solv. A: 95%AcN + 5%H_2O (0.1% FA)<br>Solv. B: H_2O (0.1% FA)<br>**Stoptime**: 1.95 min.<br>T, min A% B%<br>0.00 1 99<br>0.01 1 99<br>1.5 100 0<br>1.73 100 0	Analysis of substances with a mass greater than 600 *m/z.*

FA: formic acid; **AcN**: acetonitrile; NH_4Ac, pH = 10: an aqueous buffer based on ammonium acetate and ammonia with pH = 10.

GC/MS was used for the analysis of more volatile, non-retentive substances. Instrument specifications: Hewlett Packard GC/MS 5890/5972, Agilent GS/MS 6890N/5973N, Agilent VL GS/MS 7890A/5975C, Agilent GS/MS 7890A/5975C, Agilent GS/MS 8860/5977B. Column DB-5MS 25m × 0.2mm × 0.33 micron; oven 90 °C – 2 min, 90 °C – 280 °C – 20 °C/min, 280 °C – 5 min; injector temperature = 250 °C; detector temperature 280 °C; carrier gas Helium; gas flow 1 mL/min; ionization mode: electron Impact (EI); scan range m/z = 33 – 500; injection volume 1 μL.

All reactions requiring heating were conducted using an oil bath filled with liquid silicone grease (PMS-100) as the heat source.

The light source used in this study consisted of Luminus SST-10-UV diodes. In the mini-photoreactor, seven diodes were employed, each with an emission power of 810 mW, resulting in a total optical power output of 5.67 W. The peak emission wavelength was 365 nm, with the maximum emission wavelength at 370 nm. The distance from the light source to the irradiation vessel was 9.92 mm, and the irradiation vessel was made of 1 mm thick borosilicate glass.

For the flow photoreactor (manufactured by UORSY), the same Luminus SST-10-UV diodes were used, with an input power of 8 kW and a total emission power of 4.2 kW. The distance between the light source and the reaction zone was 68 mm, and the reaction medium flowed through a polytetrafluoroethylene spiral with a wall thickness of 0.6 mm.

2. List of abbreviations

GCMS	gas chromatography-mass spectrometry
HRMS	high-resolution mass spectrometry
LCMS	liquid chromatography-mass spectrometry
rt	room temperature
DPPA	diphenylphosphoryl azide
NBS	*N*-bromosuccinimide
DIBAL-H	diisobutylaluminum hydride
DAST	(diethylamino)sulfur trifluoride
NaHMDS	sodium *bis*(trimethylsilyl)amide solution
DMAP	4-dimethylaminopyridine
DIPEA	*N*,*N*-diisopropylethylamine
DMA	dimethylacetamide
DMF	dimethylformamide
DCC	*N*,*N′*-dicyclohexylcarbodiimide
4DPAIPN	*tris*(diphenylamino)isophthalonitrile
HATU	1-[*bis*(dimethylamino)methylene]-1*H*-1,2,3-triazolo[4,5-b]pyridinium 3-oxid hexafluorophosphate
$NiBr_2$·DME	nickel(II) bromide ethylene glycol dimethyl ether complex
$NiCl_2$·dtbbpy·	[4,4′-*bis*(1,1-dimethylethyl)-2,2′-bipyridine] nickel (II) dichloride
LED	light-emitting diode
Hantzsch ester	diethyl 2,6-dimethyl-1,4-dihydropyridine-3,5-dicarboxylate
TLC	thin-layer chromatography

3. Experimental Section. Data description and procedures

3.1. Synthesis of starting compounds

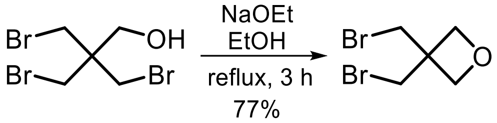

3,3-*bis(*Bromomethyl)oxetane (4)

A literature protocol from R. N. S. van der Haas, J. A. Dekker, J. Hassfeld, A. Hager, P. Fey, P. Rubenbauer, E. Damen. Synthesis and Properties of 2-Oxa-6-azaspiro[3.3]heptane Sulfonate Salts. *Synthesis* **2017**, *49,* 2394 was used.

To a stirred absolute EtOH (1000 mL) was added NaH (20.00 g, 0.50 mol, 1.04 equiv) at 0 °C (ice-water bath) over 1 h. Then 3-bromo-2,2-*bis*(bromomethyl)propan-1-ol (156.00 g, 0.48 mol, 1.00 equiv) was added. The reaction mixture was heated at 76 °C (in an oil bath with a thermocouple) for 3 h. GC analysis showed full conversion. The reaction mixture was cooled to room temperature and filtered. The filtrate was dissolved in a mixture of MeO*t*Bu (800 mL) and H_2O (2000 mL). The aqueous phase was separated and washed with MeO*t*Bu (500 mL). The combined organic layers were washed with H_2O (1 × 500 mL) and brine (1 × 300 mL), dried over Na_2SO_4, filtered, and concentrated in *vacuo*. The final product was purified by distillation (b.p. = 50-51 °C, 0.4 torr). Yield: 90.00 g, 0.368 mol, 77%, colorless oil. ^{1}H NMR (500 MHz, $CDCl_3$): δ 4.42 (s, 4H), 3.85 (s, 4H) ppm. $^{13}C\{^1H\}$ NMR (126 MHz, $CDCl_3$): δ 77.9, 45.1, 37.1 ppm. HRMS (ESI-TOF) *m/z*: [M + H]$^+$ calcd for $C_5H_9{}^{79}Br^{81}BrO$, 244.9000; found 244.8994.

General procedure A (compound **1** as an example)

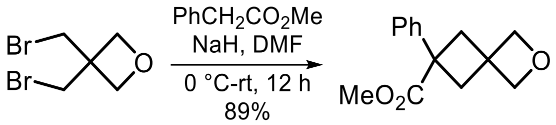

Methyl 6-phenyl-2-oxaspiro[3.3]heptane-6-carboxylate (1)

To a stirred suspension of NaH (60% in oil) (38.00 g, 0.96 mol, 2.20 equiv) in 2.5 L of DMF was added a solution of 3,3-*bis*(bromomethyl)oxetane (112.00 g, 0.458 mol 1.05 equiv) dropwise followed by the addition of methyl 2-phenylacetate (65.00 g, 0.436 mol, 1.00 equiv) in 110 mL of DMF at 0 °C (ice-water bath). Then the mixture was slowly warmed to room temperature and stirred overnight. A saturated aq. solution of NH_4Cl (200 mL) was added and the reaction mixture was extracted with hexane (3 × 200 mL). A DMF solution was evaporated to dryness, diluted with water (500 mL), and extracted with EtOAc (2 × 500 mL). The organic layers were combined, washed with water (1 × 200 mL), brine (1 × 200 mL), dried over Na_2SO_4, filtered, and concentrated

under reduced pressure. Yield: 90.00 g, 0.388 mol, 89%, colorless oil. ^{1}H NMR (500 MHz, $CDCl_3$): δ 7.36 – 7.29 (m, 2H), 7.27 – 7.20 (m, 3H), 4.76 (s, 2H), 4.53 (s, 2H), 3.62 (s, 3H), 3.11 (d, J = 13.2 Hz, 1H), 2.72 (d, J = 13.2 Hz, 1H) ppm. ^{13}C{^{1}H} NMR (126 MHz, $CDCl_3$): δ 176.0, 142.7, 128.5, 127.0, 126.5, 84.2, 82.9, 52.7, 47.4, 42.5, 38.8 ppm. GCMS $(M)^+$: 232. HRMS (ESI-TOF) *m/z*: $[M + H]^+$ calcd for $C_{14}H_{17}O_3^+$: 233.1172; found: 233.1174.

***Safety Consideration*!** Sodium hydride in DMF is widely used in organic synthesis; however, it poses significant risks due to its potential explosiveness, particularly on a larger scale. Proper precautions, including rigorous drying of solvents and controlled addition procedures, should be taken to mitigate hazards. For further details on handling and safety concerns, see Q. Yang, M. Sheng, J. J. Henkelis, S. Tu, E. Wiensch, H. Zhang, Y. Zhang, C. Tucker, D. E. Ejeh. Explosion Hazards of Sodium Hydride in Dimethyl Sulfoxide, *N,N*-Dimethylformamide, and *N,N*-Dimethylacetamide. *Org. Process Res. Dev.* **2019**, *23*, 2210-2217.

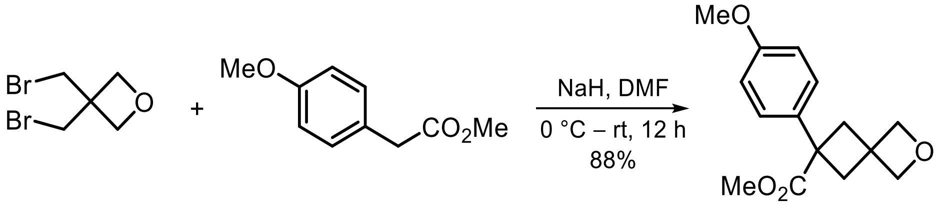

Methyl 6-(4-methoxyphenyl)-2-oxaspiro[3.3]heptane-6-carboxylate (6)

General procedure A. Yield: 60.26 g, 0.23 mol, 88%, yellow solid, m.p. = 59-60 °C. ^{1}H NMR (500 MHz, DMSO-d_6): δ 7.16 (d, J = 8.6 Hz, 2H), 6.88 (d, J = 8.6 Hz, 2H), 4.56 (s, 2H), 4.37 (s, 2H), 3.73 (s, 3H), 3.53 (s, 3H), 2.94 (d, J = 12.7 Hz, 2H), 2.64 (d, J = 12.7 Hz, 2H) ppm. ^{13}C{^{1}H} NMR (126 MHz, $CDCl_3$): δ 176.1, 158.7, 134.8, 127.6, 114.0, 84.1, 83.1, 55.4, 52.6, 46.8, 42.6, 38.8 ppm. HRMS (ESI-TOF) *m/z*: $[M + H]^+$ calcd for $C_{15}H_{19}O_4^+$: 263.1278; found: 263.1272.

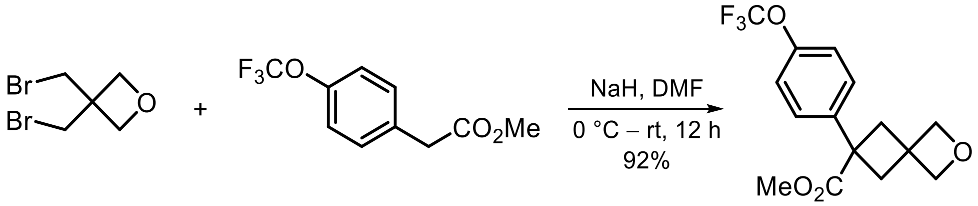

Methyl 6-(4-(trifluoromethoxy)phenyl)-2-oxaspiro[3.3]heptane-6-carboxylate (7)

General procedure A. Yield: 88.48 g, 0.28 mol, 92%, colorless oil. ^{1}H NMR (500 MHz, DMSO-d_6): δ 7.36 (d, J = 8.8 Hz, 2H), 7.32 (d, J = 8.6 Hz, 2H), 4.60 (s, 2H), 4.39 (s, 2H), 3.56 (s, 3H), 2.99 (d, J = 13.2 Hz, 2H), 2.69 (d, J = 13.2 Hz, 2H) ppm. ^{13}C{^{1}H} NMR (151 MHz, DMSO-d_6): δ 174.8, 147.0, 142.1, 128.4, 120.8, 120.1 (q, J = 256 Hz), 82.6, 81.3, 52.4, 46.4, 41.7, 38.0 ppm. ^{19}F{^{1}H} NMR (376 MHz, DMSO-d_6): δ -57.3 (s) ppm. HRMS (ESI-TOF) *m/z*: $[M + H]^+$ calcd for $C_{15}H_{16}F_3O_4^+$: 317.0995; found: 317.0990.

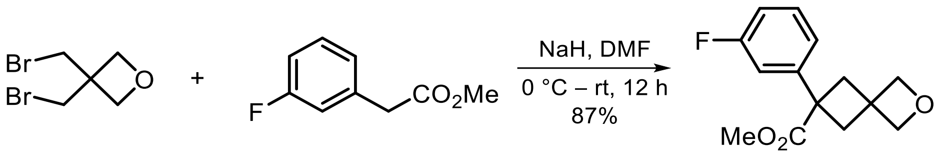

Methyl 6-(3-fluorophenyl)-2-oxaspiro[3.3]heptane-6-carboxylate (9)

General procedure A. Yield: 50.00 g, 0.20 mol, 87%, colorless oil. ^{1}H NMR (500 MHz, $CDCl_3$): δ 7.34 – 7.26 (m, 1H), 7.00 (d, *J* = 7.9 Hz, 1H), 6.97 – 6.88 (m, 2H), 4.76 (s, 2H), 4.53 (s, 2H), 3.63 (s, 3H), 3.10 (d, *J* = 13.3 Hz, 1H), 2.68 (d, *J* = 13.3 Hz, 1H) ppm. ^{13}C{^{1}H} NMR (151 MHz, $CDCl_3$): δ 175.4, 162.9 (d, *J* = 246 Hz), 145.2 (d, *J* = 7 Hz), 130.0 (d, *J* = 8 Hz), 122.1 (d, *J* = 3 Hz), 114.0 (d, *J* = 21 Hz), 113.8 (d, *J* = 22 Hz), 84.1, 82.7, 52.8, 47.3, 42.5, 38.7 ppm. ^{19}F{^{1}H} NMR (376 MHz, $CDCl_3$): δ -113.2 (s) ppm. LCMS (M + H)$^{+}$: 251. HRMS (ESI-TOF) *m/z*: [M + H]$^{+}$ calcd for $C_{14}H_{16}FO_3^{+}$: 251.1078; found: 251.1076.

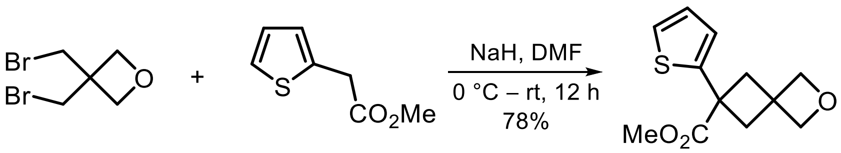

Methyl 6-(thiophen-2-yl)-2-oxaspiro[3.3]heptane-6-carboxylate (13)

General procedure A. Yield: 55.93 g, 0.235 mol, 78%, yellow oil. ^{1}H NMR (500 MHz, $CDCl_3$): δ 7.21 (d, *J* = 5.0 Hz, 1H), 6.96 – 6.92 (m, 1H), 6.91 (d, *J* = 3.3 Hz, 1H), 4.70 (s, 2H), 4.58 (s, 2H), 3.68 (s, 3H), 3.08 (d, *J* = 12.8 Hz, 2H), 2.76 (d, *J* = 12.8 Hz, 2H) ppm. ^{13}C{^{1}H} NMR (151 MHz, $CDCl_3$): δ 174.7, 146.1, 126.9, 124.9 (d, *J* = 6 Hz), 83.4 (d, *J* = 15 Hz), 52.8, 44.4, 44.2, 38.7 ppm. LCMS (M + H)$^{+}$: 239. HRMS (ESI-TOF) *m/z*: [M + H]$^{+}$ calcd for $C_{12}H_{15}O_3S^{+}$: 239.0736; found: 239.0735.

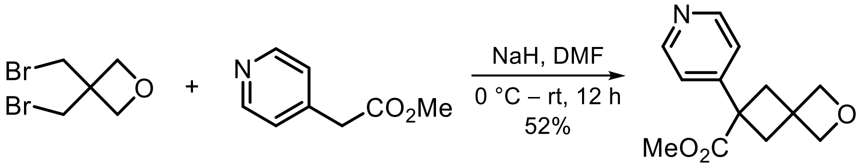

Methyl 6-(pyridin-3-yl)-2-oxaspiro[3.3]heptane-6-carboxylate (15)

General procedure A. Yield: 46.60 g, 0.20 mol, 52%, yellow oil. ^{1}H NMR (500 MHz, $CDCl_3$): δ 8.55 (dd, *J* = 4.5, 1.5 Hz, 2H), 7.14 (dd, *J* = 4.5, 1.5 Hz, 2H), 4.76 (s, 2H), 4.52 (s, 2H), 3.63 (s, 3H), 3.10 (d, *J* = 13.4 Hz, 2H), 2.67 (d, *J* = 13.4 Hz, 2H) ppm. ^{13}C{^{1}H} NMR (126 MHz, $CDCl_3$): δ 174.5, 151.4, 150.1, 121.6, 84.0, 82.6, 52.9, 47.1, 42.2, 38.9 ppm. HRMS (ESI-TOF) *m/z*: [M + H]$^{+}$ calcd for $C_{13}H_{16}NO_3^{+}$: 234.1125; found: 234.1124.

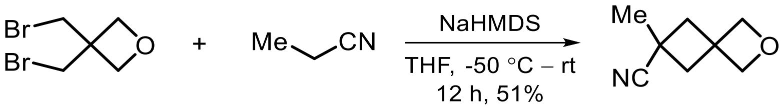

6-Methyl-2-oxaspiro[3.3]heptane-6-carbonitrile (18)

A mixture of propiononitrile (55.00 g, 1.00 mol, 1.11 equiv) and 3,3-*bis*(bromomethyl)oxetane (220.00 g, 0.90 mol, 1.00 equiv) in 0.5 L of THF was added to a solution of NaHMDS (3 L, 1M in THF, 3.00 mol, 3.33 equiv) at dropwise -50 °C. A mixture was slowly warmed to room temperature and stirred overnight. The solution was concentrated under reduced pressure, diluted with water (300 mL), and extracted with EtOAc (3 × 200 mL). The combined organic layers were washed with water (1 × 200 mL), brine (1 × 200 mL), dried over Na_2SO_4, filtered, and concentrated under reduced pressure. The final product was purified by distillation (0.4 mbar, b.p. = 65-66 °C). Yield: 69.87 g, 0.51 mol, 51%, yellow oil. ^{1}H NMR (400 MHz, $CDCl_3$): δ 4.74 (s, 2H), 4.66 (s, 2H), 2.78 (d, J = 12.9 Hz, 2H), 2.30 (d, J = 12.9 Hz, 2H), 1.42 (s, 3H) ppm. ^{13}C{^{1}H} NMR (126 MHz, $CDCl_3$): δ 124.6, 83.2, 83.1, 43.8, 38.8, 26.2, 25.0 ppm. HRMS (ESI-TOF) *m/z*: $[M + H]^+$ calcd for $C_8H_{12}NO^+$: 138.0913; found: 138.0915.

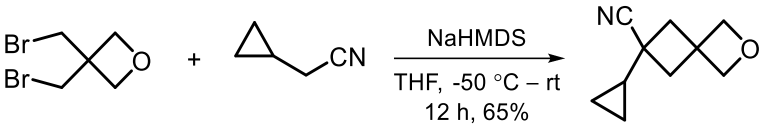

6-Cyclopropyl-2-oxaspiro[3.3]heptane-6-carbonitrile (19)

A mixture of 2-cyclopropylacetonitrile (81.00 g, 1.00 mol, 1.11 equiv) and 3,3-*bis*(bromomethyl)oxetane (220.00 g, 0.90 mol, 1.00 equiv) in 0.5 L of THF was added to a solution of NaHMDS (3 L, 1M in THF, 3.00 mol, 3.33 equiv) at dropwise -50 °C. A mixture was slowly warmed to room temperature and stirred overnight. The solution was concentrated under reduced pressure, diluted with water (500 mL), and extracted with EtOAc (3 × 300 mL). The combined organic layers were washed with water (1 × 200 mL), brine (1 × 200 mL), dried over Na_2SO_4, filtered, and concentrated under reduced pressure. The final product was purified by distillation (0.4 mbar, b.p. = 64-65 °C). Yield: 105.95 g, 0.65 mol, 65%, colorless oil. ^{1}H NMR (500 MHz, $CDCl_3$): δ 4.77 (s, 2H), 4.64 (s, 2H), 2.70 (d, J = 13.3 Hz, 2H), 2.32 (d, J = 13.3 Hz, 2H), 1.06 – 0.91 (m, 1H), 0.58 (q, J = 5.4 Hz, 2H), 0.37 (q, J = 5.4 Hz, 2H) ppm. ^{13}C{^{1}H} NMR (126 MHz, $CDCl_3$): δ 122.9, 83.4, 82.7, 41.6, 38.8, 32.9, 16.8, 2.3 ppm. HRMS (ESI-TOF) *m/z*: $[M + H]^+$ calcd for $C_{10}H_{14}NO^+$: 164.1070; found: 164.1071.

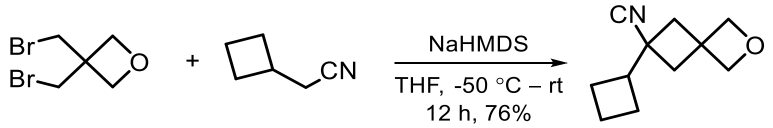

6-Cyclobutyl-2-oxaspiro[3.3]heptane-6-carbonitrile (20)

A mixture of 2-cyclobutylacetonitrile (95.00 g, 1.00 mol, 1.11 equiv) and 3,3-*bis*(bromomethyl)oxetane (220.00 g, 0.90 mol, 1.00 equiv) in 0.5 L of THF was added to a solution of NaHMDS (3 L, 1M in THF, 3.00 mol, 3.33 equiv) at dropwise -50 °C. A mixture was slowly warmed to room temperature and stirred overnight. The solution was concentrated under reduced pressure, diluted with water (500 mL), and extracted with EtOAc (3 × 300 mL). The combined organic layers were washed with water (1 × 200 mL), brine (1 × 200 mL), dried over Na_2SO_4, filtered, and concentrated under reduced pressure. The final product was purified by distillation (0.4 mbar, b.p. = 62-63 °C). Yield: 134.52 g, 0.76 mol, 76%, yellow oil. ^{1}H NMR (500 MHz, $CDCl_3$): δ 4.74 (s, 2H), 4.63 (s, 2H), 2.64 (d, *J* = 13.3 Hz, 1H), 2.51 – 2.40 (m, 1H), 2.28 (d, *J* = 13.3 Hz, 2H), 2.09 – 1.96 (m, 2H), 1.94 – 1.76 (m, 4H) ppm. $^{13}C\{^1H\}$ NMR (126 MHz, $CDCl_3$): δ 123.3, 83.4, 83.2, 40.9, 40.2, 38.6, 34.5, 24.1, 16.9 ppm. HRMS (ESI-TOF) *m/z*: $[M + H]^+$ calcd for $C_{11}H_{16}NO^+$: 178.1226; found: 178.1229.

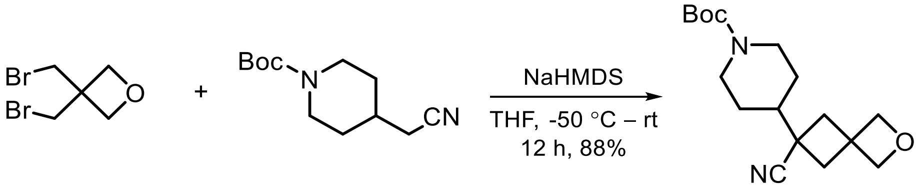

***tert*-Butyl 4-(6-cyano-2-oxaspiro[3.3]heptan-6-yl)piperidine-1-carboxylate (22)**

A mixture of of *tert*-butyl 4-(cyanomethyl)piperidine-1-carboxylate (110.00 g, 0.50 mol, 1.00 equiv) and 3,3-*bis*(bromomethyl)oxetane (122.00 g, 0.50 mol, 1.00 equiv) in 0.5 L of THF was added to a solution of NaHMDS (1.5 L, 1M in THF, 1.50 mol, 3.00 equiv) at dropwise -50 °C. A mixture was slowly warmed to room temperature and stirred overnight. The solution was concentrated under reduced pressure, diluted with water (500 mL), and extracted with EtOAc (3 × 300 mL). The combined organic layers were washed with water (1 × 200 mL), brine (1 × 200 mL), dried over Na_2SO_4, filtered, and concentrated under reduced pressure. Yield: 134.64 g, 0.44 mol, 88%, yellow solid, m.p. = 123-124 °C. ^{1}H NMR (500 MHz, $CDCl_3$): δ 4.83 (s, 2H), 4.59 (s, 2H), 4.22 (br s, 2H), 2.72 (d, *J* = 13.5 Hz, 2H), 2.60 (br s, 2H), 2.34 (d, *J* = 13.5 Hz, 2H), 1.64 (d, *J* = 12.4 Hz, 2H), 1.44 (s, 4H), 1.43 – 1.36 (m, 1H), 1.33 – 1.19 (m, 2H) ppm. $^{13}C\{^1H\}$ NMR (126 MHz, $CDCl_3$): δ 154.7, 122.7, 83.8, 82.4, 79.9, 43.9, 43.2 (br s), 41.1, 38.8, 35.9, 28.5, 26.8 ppm. HRMS (ESI-TOF) *m/z*: $[M + Na]^+$ calcd for $C_{17}H_{26}N_2NaO_3^+$: 329.1836; found: 329.1836.

3.2. Synthesis of alcohols 2, 1a and 5a-22a

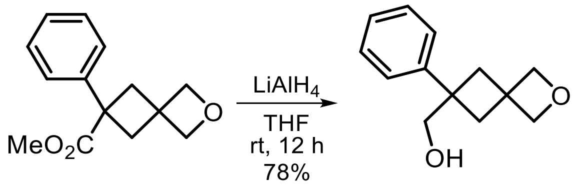

(6-Phenyl-2-oxaspiro[3.3]heptan-6-yl)methanol (2)

To a stirred suspension of $LiAlH_4$ (10.00 g, 0.26 mol, 1.20 equiv) in 1 L of THF was added a solution of methyl 6-phenyl-2-oxaspiro[3.3]heptane-6-carboxylate (50.00 g, 0.216 mol, 1.00 equiv) in 100 mL of THF dropwise at 0 °C (ice-water bath). Then the reaction mixture was stirred at the same temperature for 5 h and NaOH (11.00 g, 0.26 mol, 1.20 equiv) in 24 mL of H_2O was added dropwise at 0 °C. The reaction mixture was filtered. The precipitate was washed with THF (2 × 200 mL). The combined organic layers were concentrated under reduced pressure. Yield: 34.27 g, 0.168 mol, 78%, white solid, m.p. = 88-89 °C. ^{1}H NMR (500 MHz, DMSO-*d*$_6$): δ 7.28 (t, *J* = 7.6 Hz, 2H), 7.15 (t, *J* = 7.3 Hz, 1H), 7.10 (d, *J* = 7.1 Hz, 2H), 4.89 (t, *J* = 5.4 Hz, 1H), 4.62 (s, 2H), 4.38 (s, 2H), 3.27 (d, *J* = 5.4 Hz, 2H), 2.59 (d, *J* = 13.1 Hz, 2H), 2.36 (d, *J* = 13.1 Hz, 2H) ppm. ^{13}C{^{1}H} NMR (126 MHz, DMSO-*d*$_6$): δ 147.6, 127.8, 126.0, 125.4, 83.9, 82.1, 69.4, 42.8, 37.2 ppm. HRMS (ESI-TOF) *m/z*: [2M + H]$^+$ calcd for $C_{26}H_{33}O_4^+$: 409.2373; found: 409.2377.

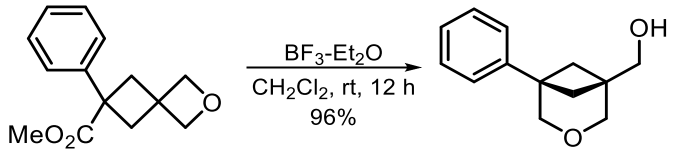

5-Phenyl-3-oxabicyclo[3.1.1]heptan-1-yl)methanol (1a)

(6-Phenyl-2-oxaspiro[3.3]heptan-6-yl)methanol (34.27 g, 0.168 mol, 1.00 equiv) was dissolved in 1.2 L of CH_2Cl_2 and cooled to 0 °C (ice-water bath). $BF_3{\cdot}Et_2O$ (5 mL) was added dropwise at 0 °C and the mixture was slowly warmed to room temperature and stirred overnight. Then 100 mL of MeOH was added and the mixture was concentrated under reduced pressure. The residue was dissolved in 1 L of MeOH and heated under reflux for 2 h. The solution was cooled to room temperature and concentrated under reduced pressure. The final product was purified by column chromatography (SiO_2, hexane/THF, 2:1). Yield: 32.84 g, 0.161 mol, 96%, colorless oil.

General procedure B

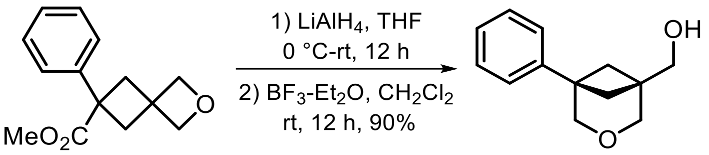

5-Phenyl-3-oxabicyclo[3.1.1]heptan-1-yl)methanol (1a)

To a stirred suspension of $LiAlH_4$ (10.00 g, 0.26 mol, 1.20 equiv) in 1 L of THF was added a solution of methyl 6-phenyl-2-oxaspiro[3.3]heptane-6-carboxylate (50.00 g, 0.216 mol, 1.00 equiv) in 100 mL of THF dropwise at 0 °C (ice-water bath). Then the reaction mixture was stirred at the same temperature for 5 h and NaOH (11.00 g, 0.26 mol, 1.20 equiv) in 24 mL of H_2O was added dropwise. The reaction mixture was warmed to 80 °C and filtered. The precipitate was washed with hot THF (2 × 200 mL). The combined organic layers were concentrated under reduced pressure. The residue was dissolved in 1.2 L of CH_2Cl_2 and cooled to 0 °C (ice-water bath). $BF_3·Et_2O$ (5 mL) was added dropwise at 0 °C and the mixture was slowly warmed to room temperature and stirred overnight. Then 100 mL of MeOH was added and the mixture was concentrated under reduced pressure. The residue was dissolved in 1 L of MeOH and heated under reflux for 2 h. The solution was cooled to room temperature and concentrated under reduced pressure. The final product was purified by column chromatography (SiO_2, hexane/THF, 2:1). Yield: 40.00 g, 0.196 mol, 90%, colorless oil. ^{1}H NMR (500 MHz, $CDCl_3$): δ 7.32 (t, *J* = 7.5 Hz, 2H), 7.22 (t, *J* = 7.3 Hz, 1H), 7.12 (d, *J* = 7.4 Hz, 2H), 3.92 (s, 2H), 3.88 (s, 2H), 3.51 (s, 2H), 2.08 (dd, *J* = 6.3, 2.0 Hz, 2H), 2.02 (d, *J* = 7.2 Hz, 2H), 1.66 (br s, 1H) ppm. ^{13}C{^{1}H} NMR (126 MHz, $CDCl_3$): δ 144.9, 128.5, 126.6, 125.6, 76.7, 72.1, 66.6, 44.0, 41.3, 36.8 ppm. GCMS $(M)^+$: 204. HRMS (ESI-TOF) *m/z*: $[M + NH_4]^+$ calcd for $C_{13}H_{20}NO_2^+$: 222.1489; found: 222.1489.

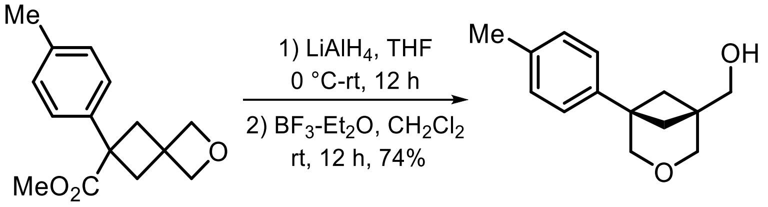

(5-(*p*-Tolyl)-3-oxabicyclo[3.1.1]heptan-1-yl)methanol (5a)

General procedure B. The final product was purified by column chromatography (SiO_2, hexane/THF, 2:1). Yield: 35.00 g, 0.16 mol, 74%, beige solid, m.p. = 69-70 °C. ^{1}H NMR (500 MHz, $CDCl_3$): δ 7.13 (d, *J* = 7.7 Hz, 2H), 7.02 (d, *J* = 7.9 Hz, 2H), 3.91 (s, 2H), 3.86 (s, 2H), 3.50 (s, 2H), 2.33 (s, 3H), 2.06 (dd, *J* = 6.2, 2.1 Hz, 2H), 1.99 (d, *J* = 7.3 Hz, 2H), 1.57 (br s, 1H) ppm. ^{13}C{^{1}H} NMR (126 MHz, $CDCl_3$): δ 142.0, 136.2, 129.2, 125.5, 76.8, 72.1, 66.6, 43.6, 41.3, 36.9,

21.2 ppm. GCMS $(M)^+$: 218. HRMS (ESI-TOF) *m/z*: $[M + NH_4]^+$ calcd for $C_{14}H_{22}NO_2^+$: 236.1645; found: 236.1643.

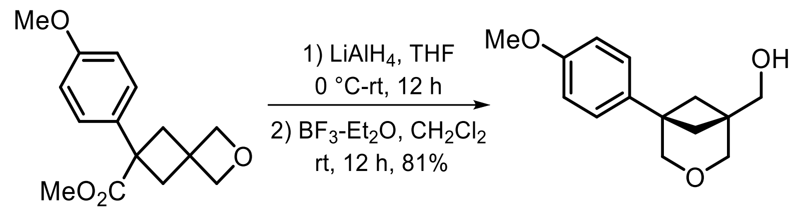

5-(4-Methoxyphenyl)-3-oxabicyclo[3.1.1]heptan-1-yl)methanol (6a)

General procedure B. The final product was purified by column chromatography (SiO_2, hexane/THF, 2:1). Yield: 40.95 g, 0.175 mol, 81%, white solid, m.p. = 49-50 °C. ^{1}H NMR (500 MHz, DMSO-d_6): δ 7.03 (d, J = 8.5 Hz, 2H), 6.86 (d, J = 8.5 Hz, 2H), 4.57 (t, J = 5.4 Hz, 1H), 3.73 (s, 2H), 3.71 (s, 3H), 3.69 (s, 2H), 3.25 (d, J = 5.4 Hz, 2H), 1.91 – 1.75 (m, 4H) ppm. ^{13}C{^{1}H} NMR (126 MHz, DMSO-d_6): δ 157.7, 137.1, 126.4, 113.7, 75.8, 71.4, 64.3, 55.0, 42.4, 40.9, 36.7 ppm. GCMS $(M)^+$: 234. HRMS (ESI-TOF) *m/z*: not detected under the experimental conditions.

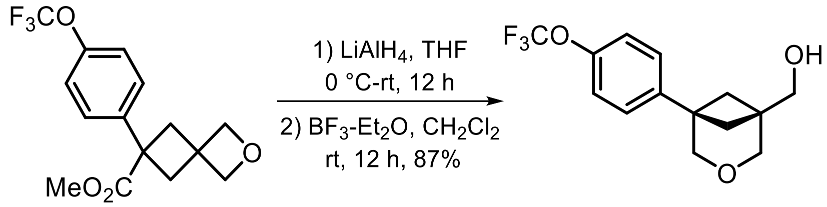

5-(4-(Trifluoromethoxy)phenyl)-3-oxabicyclo[3.1.1]heptan-1-yl)methanol (7a)

General procedure B. The final product was purified by column chromatography (SiO_2, hexane/THF, 2:1). Yield: 54.00 g, 0.1875 mol, 87%, beige solid, m.p. = 41-42 °C. ^{1}H NMR (500 MHz, $CDCl_3$): δ 7.16 (d, J = 8.3 Hz, 2H), 7.12 (d, J = 8.3 Hz, 2H), 3.91 (s, 2H), 3.85 (s, 2H), 3.51 (s, 2H), 2.07 (dd, J = 6.0, 1.7 Hz, 2H), 2.01 (d, J = 5.8 Hz, 2H), 1.54 (br s, 1H) ppm. ^{13}C{^{1}H} NMR (151 MHz, $CDCl_3$): δ 147.9, 143.7, 127.0, 121.1, 120.6 (q, J = 257 Hz), 76.5, 72.1, 66.3, 43.6, 41.3, 36.8 ppm. ^{19}F{^{1}H} NMR (376 MHz, $CDCl_3$): δ -58.3 (s) ppm. GCMS $(M)^+$: 288. HRMS (ESI-TOF) *m/z*: $[M + HCO_2]^-$ calcd for $C_{15}H_{16}F_3O_5^-$: 333.0955; found: 333.0952.

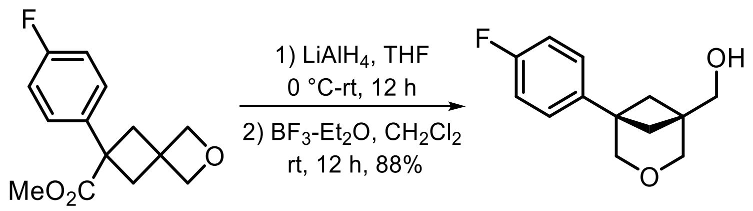

5-(4-Fluorophenyl)-3-oxabicyclo[3.1.1]heptan-1-yl)methanol (8a)

General procedure B. The final product was purified by column chromatography (SiO_2, hexane/THF, 2:1). Yield: 41.96 g, 0.189 mol, 88%, white solid. ^{1}H NMR (500 MHz, $CDCl_3$): δ 7.10

– 7.02 (m, 2H), 6.99 (t, *J* = 8.6 Hz, 2H), 3.90 (s, 2H), 3.84 (s, 2H), 3.50 (s, 2H), 2.05 (d, *J* = 6.3 Hz, 2H), 1.99 (d, *J* = 6.8 Hz, 2H), 1.54 (s, 1H) ppm. $^{13}C\{^1H\}$ NMR (126 MHz, $CDCl_3$): δ 161.7 (d, *J* = 245 Hz), 140.7, 127.2 (d, *J* = 8 Hz), 115.3 (d, *J* = 21 Hz), 76.7, 72.1, 66.4, 43.5, 41.3, 36.9 ppm. $^{19}F\{^1H\}$ NMR (376 MHz, $CDCl_3$): δ -117.0 (s) ppm. GCMS $(M)^+$: 222. HRMS (ESI-TOF) *m/z*: $[M + H – H_2O]^+$ calcd for $C_{13}H_{14}FO^+$: 205.1023; found: 205.1024.

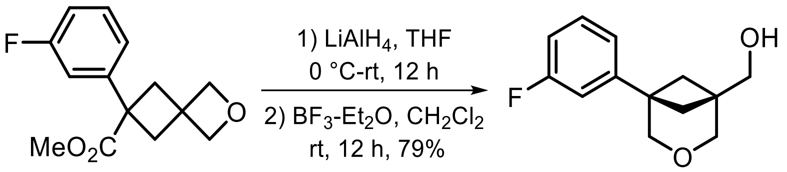

5-(3-Fluorophenyl)-3-oxabicyclo[3.1.1]heptan-1-yl)methanol (9a)

General procedure B. The final product was purified by column chromatography (SiO_2, hexane/THF, 2:1). Yield: 37.96 g, 0.171 mol, 79%, colorless oil. ^1H NMR (500 MHz, $CDCl_3$): δ 7.31 – 7.24 (m, 1H), 6.94 – 6.85 (m, 2H), 6.80 (d, *J* = 9.7 Hz, 1H), 3.91 (s, 2H), 3.86 (s, 2H), 3.52 (s, 2H), 2.06 (d, *J* = 6.3 Hz, 2H), 2.01 (d, *J* = 6.2 Hz, 2H), 1.44 (br s, 1H) ppm. $^{13}C\{^1H\}$ NMR (151 MHz, $CDCl_3$): δ 163.0 (d, *J* = 246 Hz), 147.5 (d, *J* = 7 Hz), 130.1 (d, *J* = 8 Hz), 121.24 (d, *J* = 2.6 Hz), 113.47 (d, *J* = 21.1 Hz), 112.7 (d, *J* = 21 Hz), 76.4, 72.1, 66.3, 43.9, 41.3, 36.8 ppm. $^{19}F\{^1H\}$ NMR (376 MHz, $CDCl_3$): δ -113.5 (s) ppm. GCMS $(M)^+$: 222. HRMS (ESI-TOF) *m/z*: $[M + H – H_2O]^+$ calcd for $C_{13}H_{14}FO^+$: 205.1023; found: 205.1022.

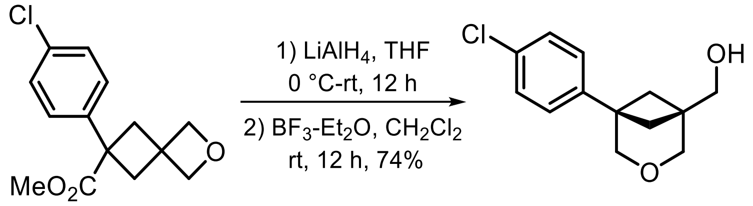

5-(4-Chlorophenyl)-3-oxabicyclo[3.1.1]heptan-1-yl)methanol (10a)

General procedure B. The final product was purified by column chromatography (SiO_2, hexane/THF, 2:1). Yield: 38.08 g, 0.16 mol, 74%, yellow solid. ^1H NMR (500 MHz, $CDCl_3$): δ 7.27 (d, *J* = 8.3 Hz, 2H), 7.03 (d, *J* = 8.3 Hz, 2H), 3.90 (s, 2H), 3.83 (s, 2H), 3.50 (s, 2H), 2.05 (dd, *J* = 6.2, 2.1 Hz, 2H), 1.99 (d, *J* = 6.0 Hz, 2H), 1.54 (br s, 1H) ppm. $^{13}C\{^1H\}$ NMR (126 MHz, $CDCl_3$): δ 143.4, 132.5, 128.7, 127.1, 76.5, 72.1, 66.4, 43.6, 41.3, 36.8 ppm. GCMS $(M)^+$: 238. HRMS (ESI-TOF) *m/z*: $[M + H – H_2O]^+$ calcd for $C_{13}H_{14}ClO^+$: 221.0728; found: 221.0727.

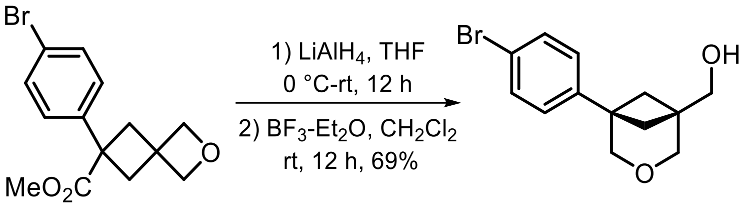

5-(4-Bromophenyl)-3-oxabicyclo[3.1.1]heptan-1-yl)methanol (11a)

General procedure B. The final product was purified by column chromatography (SiO_2, hexane/THF, 2:1). Yield: 42.30 g, 0.15 mol, 69%, yellow solid, m.p. = 80-81 °C. ^{1}H NMR (400 MHz, $CDCl_3$): δ 7.43 (d, *J* = 8.5 Hz, 2H), 6.98 (d, *J* = 8.5 Hz, 2H), 3.90 (s, 2H), 3.83 (s, 2H), 3.51 (s, 2H), 2.07 – 2.02 (m, 2H), 2.02 – 1.96 (m, 2H), 1.48 (br s, 1H) ppm. ^{13}C{^{1}H} NMR (101 MHz, $CDCl_3$): δ 143.9, 131.6, 127.4, 120.5, 76.4, 72.0, 66.4, 43.7, 41.3, 36.7 ppm. GCMS $(M)^+$: 282. HRMS (ESI-TOF) *m/z*: $[M + H – H_2O]^+$ calcd for $C_{13}H_{14}BrO^+$: 265.0223; found: 265.0220.

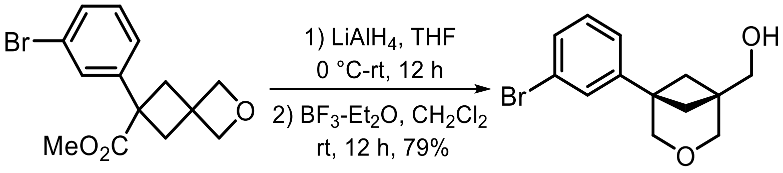

5-(3-Bromophenyl)-3-oxabicyclo[3.1.1]heptan-1-yl)methanol (12a)

General procedure B. The final product was purified by column chromatography (SiO_2, hexane/THF, 2:1). Yield: 48.11 g, 0.17 mol, 79%, yellow oil. ^{1}H NMR (500 MHz, $CDCl_3$): δ 7.35 (d, *J* = 7.8 Hz, 1H), 7.25 (d, *J* = 10.2 Hz, 1H), 7.18 (t, *J* = 7.8 Hz, 1H), 7.03 (d, *J* = 7.6 Hz, 1H), 3.90 (s, 2H), 3.84 (s, 2H), 3.50 (s, 2H), 2.05 (d, *J* = 6.4 Hz, 2H), 2.00 (d, *J* = 6.4 Hz, 2H), 1.52 (br s, 1H) ppm. ^{13}C{^{1}H} NMR (126 MHz, $CDCl_3$): δ 147.3, 130.2, 129.7, 128.9, 124.3, 122.7, 76.4, 72.0, 66.3, 43.8, 41.3, 36.8. HRMS (ESI-TOF) *m/z*: $[M + H – H_2O]^+$ calcd for $C_{13}H_{14}BrO^+$: 265.0223; found: 265.0223.

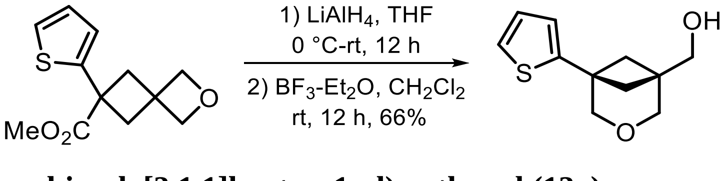

5-(Thiophen-2-yl)-3-oxabicyclo[3.1.1]heptan-1-yl)methanol (13a)

General procedure B. The final product was purified by column chromatography (SiO_2, hexane/THF, 2:1). Yield: 30.03 g, 0.143 mol, 66%, orange oil. ^{1}H NMR (500 MHz, $CDCl_3$): δ 7.17 (d, *J* = 5.0 Hz, 1H), 6.94 (dd, *J* = 4.8, 3.6 Hz, 1H), 6.76 (d, *J* = 3.3 Hz, 1H), 4.01 (s, 2H), 3.90 (s, 2H), 3.51 (s, 2H), 2.12 (d, *J* = 6.3 Hz, 2H), 2.04 (dd, *J* = 6.3, 2.2 Hz, 2H), 1.63 (br s, 1H) ppm. ^{13}C{^{1}H} NMR (126 MHz, $CDCl_3$): δ 148.5, 127.0, 123.8, 122.6, 75.8, 72.0, 66.3, 41.7, 41.0, 39.2 ppm. GCMS $(M)^+$: 210. HRMS (ESI-TOF) *m/z*: $[M + H]^+$ calcd for $C_{11}H_{15}O_2S^+$: 211.0787; found: 211.0786.

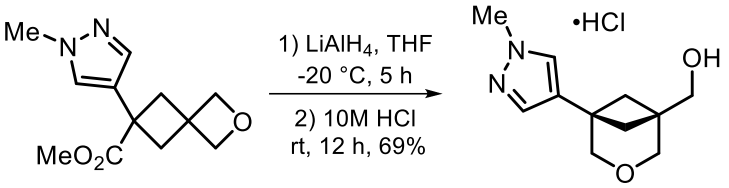

5-(1-Methyl-1*H*-pyrazol-4-yl)-3-oxabicyclo[3.1.1]heptan-1-yl)methanol hydrochloride (14a)

To a stirred suspension of $LiAlH_4$ (10.00 g, 0.26 mol, 1.20 equiv) in 1 L of THF was added a solution of methyl 6-(1-methyl-1*H*-pyrazol-4-yl)-2-oxaspiro[3.3]heptane-6-carboxylate (51.00 g, 0.216 mol, 1.00 equiv) in 100 mL of THF at -20 °C dropwise. Then the reaction mixture was stirred at the same temperature for 5 h and NaOH (11.00 g, 0.26 mol, 1.20 equiv) in 24 mL of H_2O was added dropwise at the same temperature. After this, the reaction mixture was warmed to 80 °C and filtered. The precipitate was washed with hot THF (2 × 100 mL). The combined organic layers were concentrated under reduced pressure. The residue was dissolved in 200 mL of water and cooled to 0 °C and 25 mL of 10M HCl was added dropwise. The reaction mixture was slowly warmed to room temperature and stirred overnight. The mixture was concentrated under reduced pressure. The final product was purified by column chromatography (SiO_2, MeO*t*Bu/MeOH, 1:1). Yield: 36.40 g, 0.149 mol, 69%, yellow solid, m.p. = 122-123 °C. ^1H NMR (500 MHz, DMSO-d_6): δ 7.59 (s, 1H), 7.39 (s, 1H), 6.95 (br s, 2H), 3.79 (s, 5H), 3.70 (s, 2H), 3.23 (s, 1H), 1.87 (d, J = 5.8 Hz, 2H), 1.73 (dd, J = 6.0, 2.1 Hz, 2H) ppm. $^{13}C\{^1H\}$ NMR (126 MHz, DMSO-d_6): δ 135.2, 128.0, 125.0, 74.1, 71.5, 64.4, 41.4, 38.5, 36.0 ppm. LCMS $(M + H)^+$: 209. HRMS (ESI-TOF) *m/z*: $[M + H]^+$ calcd for $C_{11}H_{17}N_2O_2^+$: 209.1285; found: 209.1284.

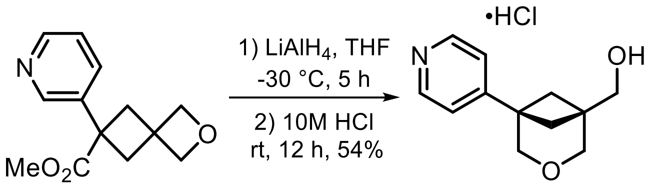

5-(Pyridin-4-yl)-3-oxabicyclo[3.1.1]heptan-1-yl)methanol hydrochloride (15a)

To a stirred suspension of $LiAlH_4$ (10.00 g, 0.26 mol, 1.20 equiv) in 1 L of THF was added a solution of methyl 6-(pyridin-4-yl)-2-oxaspiro[3.3]heptane-6-carboxylate (50.00 g, 0.216 mol, 1.00 equiv) in 100 mL of THF at -30 °C dropwise. Then the reaction mixture was stirred at the same temperature for 5 h and NaOH (11.00 g, 0.26 mol, 1.20 equiv) in 24 mL of H_2O was added dropwise at the same temperature. After this, the reaction mixture was warmed to 80 °C and filtered. The precipitate was washed with hot THF (2 × 100 mL). The combined organic layers were concentrated under reduced pressure. The residue was dissolved in 200 mL of water, cooled to 0 °C, and 25 mL of 10M HCl was added dropwise. The reaction mixture was slowly warmed to room temperature and stirred overnight. The mixture was concentrated under reduced pressure. The final product was purified by column chromatography (SiO_2, MeO*t*Bu/MeOH, 1:1). Yield: 28.01 g,

0.116 mol, 54%, beige solid, m.p. = 162-163 °C. ^{1}H NMR (500 MHz, DMSO-d_6): δ 8.85 (d, J = 5.9 Hz, 1H), 7.83 (d, J = 5.9 Hz, 1H), 3.83 (s, 1H), 3.78 (s, 1H), 3.27 (s, 1H), 2.11 – 1.96 (m, 2H). ^{13}C{^{1}H} NMR (126 MHz, DMSO-d_6): δ 163.2, 141.7, 124.2, 73.2, 71.3, 63.6, 43.9, 40.9, 36.3 ppm. LCMS (M + H)$^+$: 206. HRMS (ESI-TOF) *m/z*: [M + H]$^+$ calcd for $C_{12}H_{16}NO_2^+$: 206.1176; found: 206.1175.

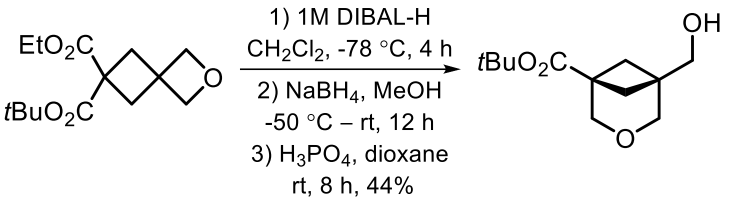

***tert*-Butyl 5-(hydroxymethyl)-3-oxabicyclo[3.1.1]heptane-1-carboxylate (16a)**

To a stirred solution of 6-*tert*-butyl 6-ethyl 2-oxaspiro[3.3]heptane-6,6-dicarboxylate (54.00 g, 0.20 mol, 1.00 equiv) in CH_2Cl_2 (1 L) was added a solution of 1M DIBAL-H in cyclohexane (400 mL, 0.40 mol, 2.00 equiv) dropwise at -78 °C. The resulting mixture was stirred at the same temperature for 4 h, and then a solution of citric acid (96.00 g, 0.50 mol, 2.50 equiv) in 500 mL of H_2O was added dropwise. The reaction mixture was slowly warmed to room temperature. The organic layer was separated and concentrated under reduced pressure. The residue was dissolved in MeOH (400 ml), cooled to -50 °C, and $NaBH_4$ (15.00 g, 0.40 mol, 2.00 equiv) was added in small portions maintaining temperature at the same point. The solution was slowly warmed to room temperature and stirred overnight. The mixture was concentrated under reduced pressure, diluted with 1 L of water, and extracted with CH_2Cl_2 (2 × 500 mL). The combined organic layers were washed with brine (1 × 200 mL), dried over Na_2SO_4, filtered, and concentrated under reduced pressure. *tert*-Butyl 6-(hydroxymethyl)-2-oxaspiro[3.3]heptane-6-carboxylate (30.00 g, 0.13 mol) was dissolved in 300 mL of dioxane and 30 mL of H_3PO_4 was added at room temperature. The progress of the recyclization was monitored by TLC (~ 8 h). If partial conversion was observed, an additional 30 mL portion of H_3PO_4 was added. After the reaction was finished, the mixture was slowly poured into water, alkalized with K_2CO_3 to pH = 8, and extracted with CH_2Cl_2 (2 × 500 mL). The combined organic layers were washed with brine (1 × 200 mL), dried over Na_2SO_4, filtered, and concentrated under reduced pressure. The final product was purified by column chromatography (SiO_2, hexane/THF, 1:1). Yield over 3 steps: 20.06 g, 0.088 mol, 44%, colorless oil. ^{1}H NMR (500 MHz, $CDCl_3$): δ 3.97 (s, 2H), 3.80 (s, 2H), 3.46 (s, 2H), 2.13 (d, J = 6.4 Hz, 2H), 1.77 (dd, J = 6.2, 2.1 Hz, 2H), 1.42 (s, 9H) ppm. ^{13}C{^{1}H} NMR (151 MHz, $CDCl_3$): δ 172.2, 81.0, 72.0, 70.9, 66.1, 44.4, 41.2, 35.6, 28.1 ppm. HRMS (ESI-TOF) *m/z*: [M + NH_4]$^+$ calcd for $C_{12}H_{24}NO_4^+$: 246.1700; found: 246.1698.

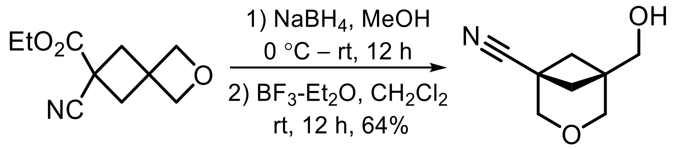

5-(Hydroxymethyl)-3-oxabicyclo[3.1.1]heptane-1-carbonitrile (17a)

To a stirred solution of ethyl 6-cyano-2-oxaspiro[3.3]heptane-6-carboxylate(120.00 g, 0.61 mol, 1.00 equiv) in MeOH (2 L) was added $NaBH_4$ (53.00 g, 1.40 mol, 2.30 equiv) in portions at 0 °C. The resulting mixture was slowly warmed to room temperature, stirred overnight and concentrated under reduced pressure. The residue was diluted with 500 mL of water, and extracted with EtOAc (3 × 500 mL). The combined organic layers were dried over Na_2SO_4, filtered through a pad of celite, and concentrated under reduced pressure. 6-(Hydroxymethyl)-2-oxaspiro[3.3]heptane-6-carbonitrile (84.00 g, 0.55 mol) was dissolved in 2 L of CH_2Cl_2, cooled to 0 °C and $BF_3{\cdot}Et_2O$ (6 mL, 0.05 mol) was added. The reaction mixture was stirred overnight at room temperature and concentrated under reduced pressure. The residue was dissolved in 500 mL of MeOH and heated under reflux for 3 h. The solution was cooled to room temperature and concentrated under reduced pressure. The final product was purified by distillation (0.4 mbar, b.p. = 115-116 °C). Yield over two steps: 59.67 g, 0.39 mol, 64%, colorless oil. ^{1}H NMR (500 MHz, $CDCl_3$): δ 4.02 (s, 2H), 3.80 (s, 2H), 3.44 (s, 2H), 2.38 (d, J = 8.0 Hz, 2H), 2.01 (dd, J = 6.5, 2.3 Hz, 2H), 1.77 (br s, 1H) ppm. ^{13}C{^{1}H} NMR (126 MHz, $CDCl_3$): δ 120.1, 71.5, 70.3, 64.7, 43.4, 37.0, 30.7 ppm. GCMS $(M)^+$: 153. HRMS (ESI-TOF) *m/z*: $[M + NH_4]^+$ calcd for $C_8H_{12}NO_2^+$: 154.0863; found: 154.0861.

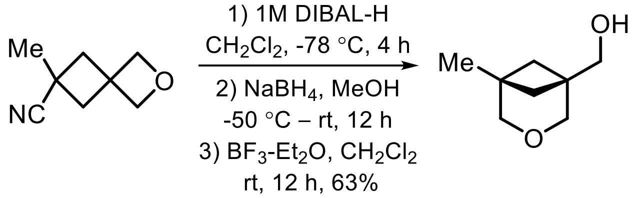

5-Methyl-3-oxabicyclo[3.1.1]heptan-1-yl)methanol (18a)

To a stirred solution of 6-methyl-2-oxaspiro[3.3]heptane-6-carbonitrile (70.00 g, 0.51 mol, 1.00 equiv) in CH_2Cl_2 (1 L) was added a solution of 1M DIBAL-H in cyclohexane (600 mL, 0.60 mol, 1.18 equiv) dropwise at -78 °C. The resulting mixture was stirred at the same temperature for 4 h and then a solution of citric acid (98.00 g, 0.51 mol, 1.00 equiv) in 500 mL of H_2O was added dropwise. The reaction mixture was slowly warmed to room temperature and stirred overnight. The organic layer was separated and concentrated under reduced pressure. The residue was dissolved in MeOH (400 mL), and cooled to 0 °C, and $NaBH_4$ (23.00 g, 0.60 mol, 1.18 equiv) was added in small portions maintaining temperature at the same point. The solution was slowly warmed to room temperature and stirred overnight. The mixture was concentrated under reduced pressure, diluted with 1 L of water, and extracted with CH_2Cl_2 (2 × 500 mL). The combined organic layers were

washed with brine (1 × 200 mL), dried over Na_2SO_4, filtered, and concentrated under reduced pressure. (6-Methyl-2-oxaspiro[3.3]heptan-6-yl)methanol (60.00 g, 0.42 mol) was dissolved in 2 L of CH_2Cl_2, cooled to 0 °C, and $BF_3·Et_2O$ (6 mL, 0.05mol) was added. The reaction mixture was stirred overnight and concentrated under reduced pressure. The residue was dissolved in 500 mL of MeOH and heated under reflux for 3 h. The solution was cooled to room temperature and concentrated under reduced pressure. The final product was purified by distillation (0.4 mbar, b.p. = 72-73 °C). Yield over 3 steps: 45.44 g, 0.32 mol, 63%, colorless oil. ^1H NMR (500 MHz, $CDCl_3$): δ 3.78 (s, 2H), 3.66 (s, 2H), 3.41 (s, 2H), 1.72 (br s, 1H), 1.61 (dd, *J* = 6.3, 1.9 Hz, 2H), 1.55 (d, *J* = 7.2 Hz, 2H), 0.97 (s, 3H) ppm. $^{13}C\{^1H\}$ NMR (151 MHz, $CDCl_3$): δ 76.2, 72.2, 66.7, 41.5, 38.1, 36.7, 22.5 ppm. HRMS (ESI-TOF) *m/z*: $[M + H]^+$ calcd for $C_8H_{15}O_2^+$: 143.1067; found: 143.1065.

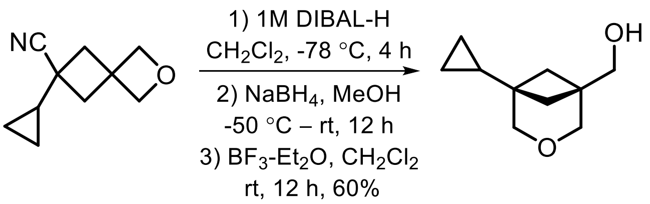

5-Cyclopropyl-3-oxabicyclo[3.1.1]heptan-1-yl)methanol (19a)

To a stirred solution of 6-cyclopropyl-2-oxaspiro[3.3]heptane-6-carbonitrile (105.00 g, 0.65 mol, 1.00 equiv) in CH_2Cl_2 (1 L) was added a solution of 1M DIBAL-H in cyclohexane (700 mL, 0.70 mol, 1.08 equiv) dropwise at -78 °C. The resulting mixture was stirred at the same temperature for 4 h and then a solution of citric acid (147.00 g, 0.70 mol, 1.08 equiv) in 500 mL of H_2O was added dropwise. The reaction mixture was slowly warmed to room temperature and stirred overnight. The organic layer was separated and concentrated under reduced pressure. The residue was dissolved in MeOH (400 mL), and cooled to 0 °C, and $NaBH_4$ (27.00 g, 0.70 mol, 1.08 equiv) was added in small portions maintaining temperature at the same point. The solution was slowly warmed to room temperature and stirred overnight. The mixture was concentrated under reduced pressure, diluted with 1 L of water, and extracted with CH_2Cl_2 (2 × 500 mL). The combined organic layers were washed with brine (1 × 200 mL), dried over Na_2SO_4, filtered, and concentrated under reduced pressure. 6-Cyclopropyl-2-oxaspiro[3.3]heptan-6-yl)methanol (80.00 g, 0.48 mol) was dissolved in 2 L of CH_2Cl_2, cooled to 0 °C and $BF_3·Et_2O$ (6 mL, 0.05 mol) was added. The reaction mixture was stirred overnight and concentrated under reduced pressure. The residue was dissolved in 500 mL of MeOH and heated under reflux for 3 h. The solution was cooled to room temperature and concentrated under reduced pressure. The final product was purified by distillation (0.4 mbar, b.p. = 102-103 °C). Yield over 3 steps: 65.52 g, 0.39 mol, 60%, colorless oil. ^1H NMR (500 MHz, $CDCl_3$): δ 3.78 (s, 4H), 3.39 (s, 2H), 1.71 (s, 1H), 1.46 – 1.32 (m, 4H), 0.67 – 0.56 (m, 1H), 0.31 (q, *J* = 4.9 Hz, 1H), 0.14 (q, *J* = 4.9 Hz, 2H) ppm. $^{13}C\{^1H\}$ NMR (126 MHz, $CDCl_3$): δ 76.0, 72.7,

66.8, 40.7, 40.5, 34.0, 14.3, 0.1 ppm. HRMS (ESI-TOF) *m/z*: $[M + NH_4]^+$ calcd for $C_{10}H_{20}NO_2^+$: 186.1489; found: 186.1489.

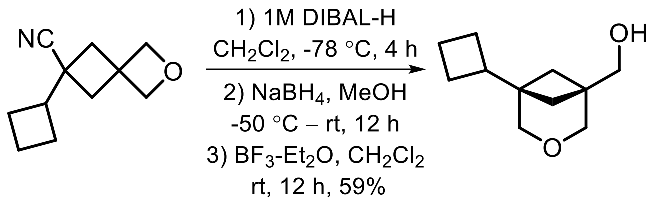

5-Cyclobutyl-3-oxabicyclo[3.1.1]heptan-1-yl)methanol (20a)

The same procedure as described for compound **19a** was applied. The final product was purified by distillation (0.4 mbar, b.p. = 111-112 °C). Yield over 3 steps: 81.90 g, 0.45 mol, 59%, colorless oil. ^{1}H NMR (500 MHz, $CDCl_3$): δ 3.80 (s, 2H), 3.61 (s, 2H), 3.43 (s, 2H), 2.16 (p, *J* = 8.2 Hz, 1H), 1.86 – 1.63 (m, 9H), 1.41 (dd, *J* = 6.2, 2.5 Hz, 1H) ppm. $^{13}C\{^1H\}$ NMR (126 MHz, $CDCl_3$): δ 73.9, 72.8, 66.9, 42.4, 40.6, 38.3, 32.3, 23.0, 18.1 ppm. GCMS $(M)^+$: 184. HRMS (ESI-TOF) *m/z*: $[M + H]^+$ calcd for $C_{11}H_{19}O_2^+$: 183.1380; found: 183.1379.

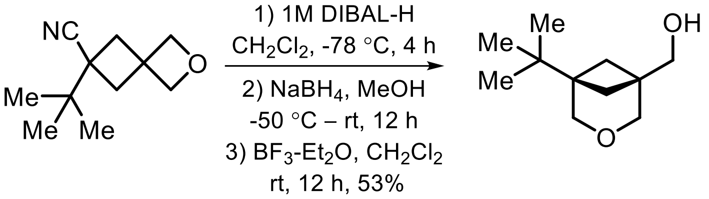

5-(*tert*-Butyl)-3-oxabicyclo[3.1.1]heptan-1-yl)methanol (21a)

The same procedure as described for compound **19a** was applied. The final product was purified by distillation (0.4 mbar, b.p. = 82-83 °C). Yield over 3 steps: 77.28 g, 0.42 mol, 53%, colorless oil. ^{1}H NMR (500 MHz, $CDCl_3$): δ 3.84 (s, 2H), 3.74 (s, 2H), 3.43 (s, 2H), 1.74 (d, *J* = 6.4 Hz, 2H), 1.44 (dd, *J* = 6.4, 2.6 Hz, 2H), 0.81 (s, 9H) ppm. $^{13}C\{^1H\}$ NMR (126 MHz, $CDCl_3$): δ 72.6, 72.4, 67.1, 45.9, 39.4, 32.0, 25.3 ppm. GCMS $(M)^+$: 184. HRMS (ESI-TOF) *m/z*: $[M + H]^+$ calcd for $C_{11}H_{21}O_2^+$: 185.1536; found: 185.1534.

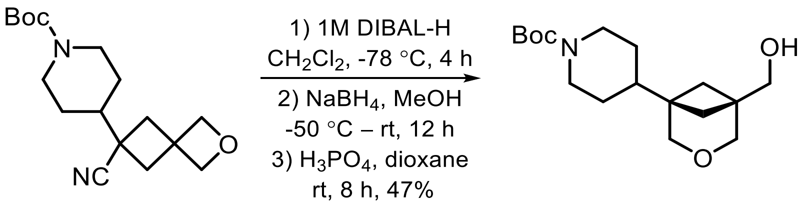

***tert*-Butyl 4-(5-(hydroxymethyl)-3-oxabicyclo[3.1.1]heptan-1-yl)piperidine-1-carboxylate (22a)**

The same procedure as described for compound **16a** was applied. The final product was purified by column chromatography (SiO_2, hexane/THF, 1:1). Yield over 3 steps: 65.31 g, 0.21 mol, 47%, colorless oil. ^{1}H NMR (500 MHz, $CDCl_3$): δ 4.14 (br s, 2H), 3.77 (s, 1H), 3.74 (s, 2H), 3.42 (s, 2H),

2.57 (br s, 2H), 1.63 – 1.46 (m, 7H), 1.44 (s, 9H), 1.36 – 1.21 (m, 2H), 1.14 – 1.05 (m, 2H) ppm. $^{13}C\{^{1}H\}$ NMR (151 MHz, $CDCl_3$): δ 154.9, 79.6, 73.1, 72.6, 66.6, 44.4 (br s), 42.4, 42.1, 41.0, 34.5, 28.6, 26.7 ppm. HRMS (ESI-TOF) *m/z*: $[M + Na]^+$ calcd for $C_{17}H_{29}NNaO_4^+$: 334.1989; found: 334.1988.

3.3. Synthesis of carboxylic acids

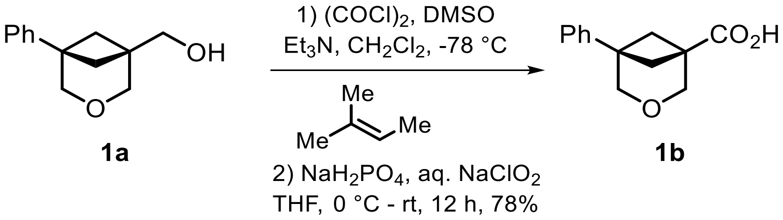

5-Phenyl-3-oxabicyclo[3.1.1]heptane-1-carboxylic acid (1b)

To a stirred solution of oxalyl chloride (19 mL, 0.19 mol, 1.30 equiv) in 2 L of CH_2Cl_2 was added a solution of DMSO (16 mL, 0.22 mol, 1.50 equiv) in 100 mL of CH_2Cl_2 dropwise at -78 °C. The resulting mixture was stirred at the same temperature for 1 h, and the solution of (5-phenyl-3-oxabicyclo[3.1.1]heptan-1-yl)methanol (30.00 g, 0.147 mol, 1.00 equiv) in 150 mL of CH_2Cl_2 was added dropwise. The mixture was stirred at -60 °C for 4 h and then Et_3N (82 mL, 0.59 mol, 4.00 equiv) was added. The resulting solution was slowly warmed and stirred at room temperature overnight. The organic layer was washed with water (2 × 300 mL), dried over Na_2SO_4, filtered, and evaporated to dryness under reduced pressure. The residue was dissolved in 1 L of THF and 2-methylbut-2-ene (48 mL, 0.45 mol, 3.06 equiv) and NaH_2PO_4 (21.60 g, 0.18 mol, 1.22 equiv) in 1 L of water were added. Then a solution of $NaClO_2$ (16.20 g, 0.18 mol, 1.22 equiv) in 100 mL of water was added to the mixture dropwise at 0 °C. The reaction mixture was slowly warmed to room temperature and stirred overnight. The mixture was concentrated under reduced pressure. The residue was diluted with water and acidified with aq. conc. HCl to pH ~ 5. The solution was extracted with EtOAc (3 × 200 mL). The combined organic layers were washed with brine (1 × 200 mL), dried over Na_2SO_4, filtered, and concentrated under reduced pressure. The final product was purified by crystallization from hexane. Yield: 25.07 g, 0.115 mol, 78%, white solid, m.p. = 162-163 °C. ^{1}H NMR (500 MHz, DMSO-d_6): δ 12.59 (br s, 1H), 7.32 (t, J = 7.5 Hz, 2H), 7.23 (t, J = 7.3 Hz, 1H), 7.14 (d, J = 7.3 Hz, 2H), 3.96 (s, 2H), 3.75 (s, 2H), 2.32 (d, J = 6.2 Hz, 2H), 2.20 (dd, J = 6.2, 2.1 Hz, 2H) ppm. ^{13}C{^{1}H} NMR (126 MHz, DMSO-d_6): δ 173.6, 144.0, 128.3, 126.5, 125.2, 74.6, 69.2, 42.8, 42.5, 38.1 ppm. LCMS (M - H)$^-$: 217. HRMS (ESI-TOF) m/z: [M - H]$^-$ calcd for $C_{13}H_{13}O_3^-$: 217.0870; found: 217.0866.

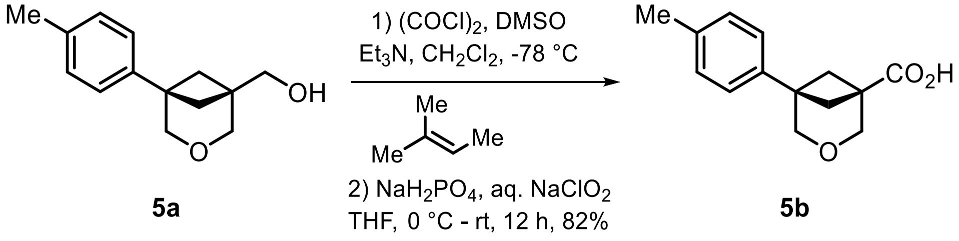

5-(*p*-Tolyl)-3-oxabicyclo[3.1.1]heptane-1-carboxylic acid (5b)

The same procedure as for **1b** was used. The final product was purified by crystallization from hexane. Yield: 28.07 g, 0.121 mol, 82%, white solid, m.p. = 158-159 °C. ^{1}H NMR (500 MHz, DMSO-d_6): δ 12.57 (s, 1H), 7.13 (d, *J* = 7.2 Hz, 2H), 7.02 (d, *J* = 7.2 Hz, 2H), 3.95 (s, 2H), 3.72 (s, 2H), 2.27 (br s, 2H), 2.26 (s, 3H), 2.16 (d, *J* = 4.8 Hz, 2H) ppm. ^{13}C{^{1}H} NMR (126 MHz, DMSO-d_6): δ 173.6, 141.0, 135.5, 128.9, 125.1, 74.6, 69.2, 42.5, 38.1, 20.6 ppm. LCMS (M - H)$^-$: 231. HRMS (ESI-TOF) *m/z*: [M - H]$^-$ calcd for $C_{14}H_{15}O_3^-$: 231.1027; found: 231.1026.

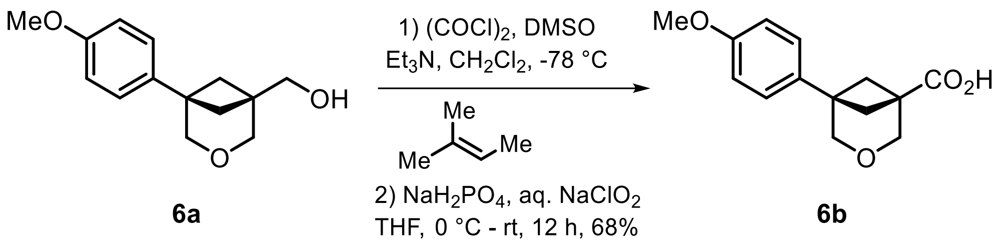

6a **6b**

5-(4-Methoxyphenyl)-3-oxabicyclo[3.1.1]heptane-1-carboxylic acid (6b)

The same procedure as for **1b** was used. The final product was purified by crystallization from hexane. Yield: 26.04 g, 0.105 mol, 68%, white solid, m.p. = 122-123 °C. ^{1}H NMR (500 MHz, CDCl$_3$): δ 7.02 (d, *J* = 8.5 Hz, 2H), 6.86 (d, *J* = 8.5 Hz, 2H), 4.12 (s, 2H), 3.86 (s, 2H), 3.79 (s, 3H), 2.52 (d, *J* = 6.3 Hz, 2H), 2.27 (dd, *J* = 6.3, 2.2 Hz, 2H) ppm. ^{13}C{^{1}H} NMR (126 MHz, CDCl$_3$): δ 178.4, 158.6, 135.9, 126.5, 114.1, 75.9, 69.6, 55.4, 43.10, 43.06, 38.7 ppm. LCMS (M - H)$^-$: 247. HRMS (ESI-TOF) *m/z*: [M - H]$^-$ calcd for $C_{14}H_{15}O_4^-$: 247.0976; found: 247.0973.

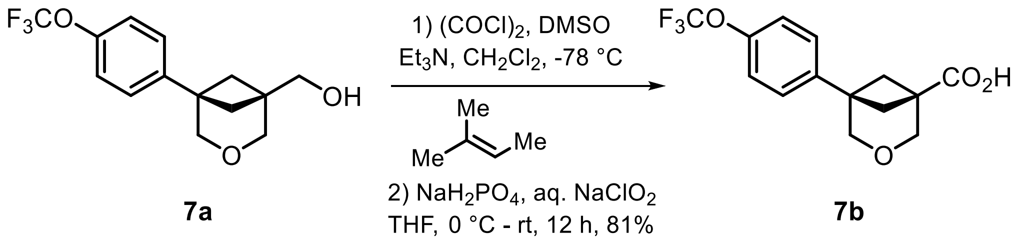

7a **7b**

5-(4-(Trifluoromethoxy)phenyl)-3-oxabicyclo[3.1.1]heptane-1-carboxylic acid (7b)

The same procedure as for **1b** was used. The final product was purified by crystallization from hexane. Yield: 36.00 g, 0.119 mol, 81%, yellow solid, m.p. = 114-115 °C. ^{1}H NMR (500 MHz, DMSO-d_6): δ 12.62 (br s, 1H), 7.32 (d, *J* = 8.6 Hz, 2H), 7.27 (d, *J* = 8.6 Hz, 2H), 3.96 (s, 2H), 3.76 (s, 2H), 2.33 (d, *J* = 6.3 Hz, 2H), 2.21 (dd, *J* = 6.3, 2.1 Hz, 2H) ppm. ^{13}C{^{1}H} NMR (151 MHz, DMSO-d_6): δ 173.5, 147.0, 143.4, 127.4, 121.0, 120.1 (q, *J* = 256 Hz), 74.2, 69.2, 42.5, 42.4, 38.1 ppm. ^{19}F{^{1}H} NMR (376 MHz, DMSO-d_6): δ -57.3 (s) ppm. LCMS (M - H)$^-$: 301. HRMS (ESI-TOF) *m/z*: [M - H]$^-$ calcd for $C_{14}H_{12}F_3O_4^-$: 301.0693; found: 301.0688.

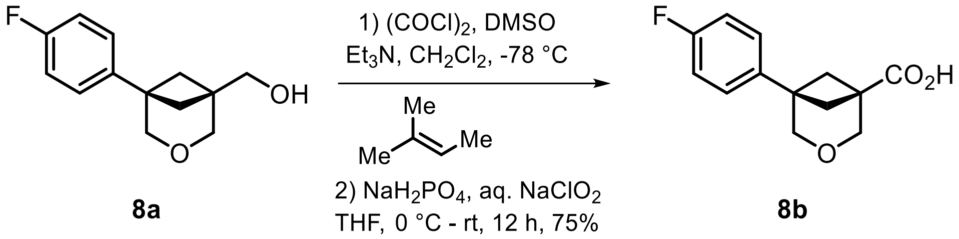

8a **8b**

5-(4-Fluorophenyl)-3-oxabicyclo[3.1.1]heptane-1-carboxylic acid (8b)

The same procedure as for **1b** was used. The final product was purified by crystallization from hexane. Yield: 26.00 g, 0.11 mol, 75%, white solid, m.p. = 140-142 °C. ^{1}H NMR (500 MHz, DMSO-*d$_6$*): δ 12.54 (br s, 1H), 7.21 – 7.10 (m, 4H), 3.95 (s, 2H), 3.74 (s, 2H), 2.31 (d, *J* = 8.1 Hz, 2H), 2.19 (dd, *J* = 6.3, 2.4 Hz, 2H) ppm. ^{13}C{^{1}H} NMR (151 MHz, DMSO-*d$_6$*): δ 173.5, 161.0 (d, *J* = 243 Hz), 140.2 (d, *J* = 3 Hz), 127.3 (d, *J* = 8 Hz), 115.1 (d, *J* = 21 Hz), 74.5, 69.2, 42.4, 42.4, 38.1 ppm. ^{19}F{^{1}H} NMR (376 MHz, DMSO-*d$_6$*): δ -116.8 (s) ppm. LCMS (M - H)$^-$: 235. HRMS (ESI-TOF) *m/z*: [M - H]$^-$ calcd for $C_{13}H_{12}FO_3^-$: 235.0776; found: 235.0771.

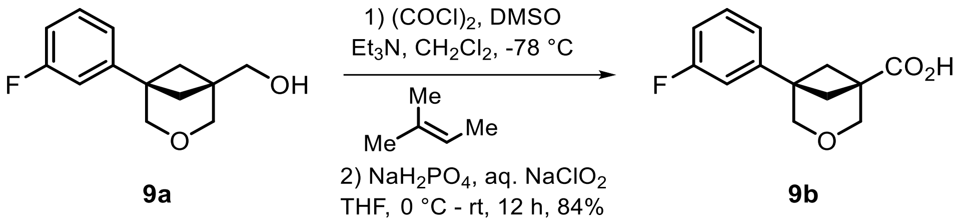

9a **9b**

5-(3-Fluorophenyl)-3-oxabicyclo[3.1.1]heptane-1-carboxylic acid (9b)

The same procedure as for **1b** was used. The final product was purified by crystallization from hexane. Yield: 29.03 g, 0.123 mol, 84%, white solid, m.p. = 94-95 °C. ^{1}H NMR (500 MHz, DMSO-*d$_6$*): δ 12.58 (s, 1H), 7.37 (dd, *J* = 14.4, 7.5 Hz, 1H), 7.10 – 7.03 (m, 1H), 6.99 (d, *J* = 7.5 Hz, 2H), 3.95 (s, 2H), 3.76 (s, 2H), 2.34 – 2.30 (m, 2H), 2.20 (dd, *J* = 6.3, 2.4 Hz, 2H) ppm. ^{13}C{^{1}H} NMR (126 MHz, DMSO-*d$_6$*): δ 173.3, 162.2 (d, *J* = 244 Hz), 146.8 (d, *J* = 7 Hz), 130.3 (d, *J* = 8 Hz), 121.3, 113.3 (d, *J* = 21 Hz), 112.3 (d, *J* = 21 Hz), 74.1, 69.2, 42.7, 42.4, 38.0 ppm. ^{19}F{^{1}H} NMR (376 MHz, DMSO-*d$_6$*): δ -113.5 (s) ppm. LCMS (M - H)$^-$: 235. HRMS (ESI-TOF) *m/z*: [M - H]$^-$ calcd for $C_{13}H_{12}FO_3^-$: 235.0776; found: 235.0779.

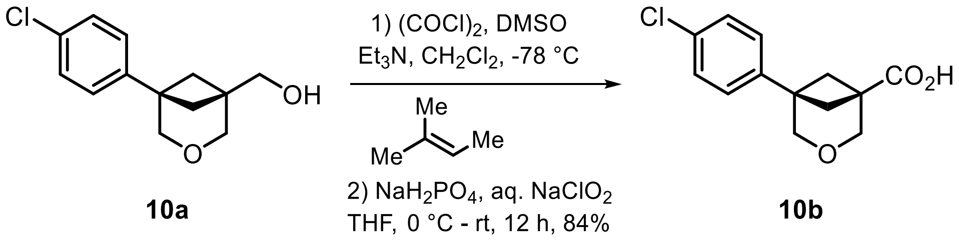

10a **10b**

5-(4-Chlorophenyl)-3-oxabicyclo[3.1.1]heptane-1-carboxylic acid (10b)

The same procedure as for **1b** was used. The final product was purified by crystallization from hexane. Yield: 32.00 g, 0.127 mol, 84%, yellow solid, m.p. = 146-147 °C. ^{1}H NMR (500 MHz, DMSO-d_6): δ 12.59 (br s, 1H), 7.37 (d, J = 8.3 Hz, 2H), 7.17 (d, J = 8.3 Hz, 2H), 3.95 (s, 2H), 3.74 (s, 2H), 2.31 (d, J = 6.2 Hz, 2H), 2.19 (dd, J = 6.2, 1.9 Hz, 2H) ppm. ^{13}C{^{1}H} NMR (126 MHz, DMSO-d_6): δ 173.4, 142.9, 131.2, 128.3, 127.3, 74.2, 69.2, 42.44, 42.41, 38.0 ppm. LCMS (M - H)$^-$: 251. HRMS (ESI-TOF) *m/z*: [M - H]$^-$ calcd for $C_{13}H_{12}ClO_3^-$: 251.0480; found: 251.0480.

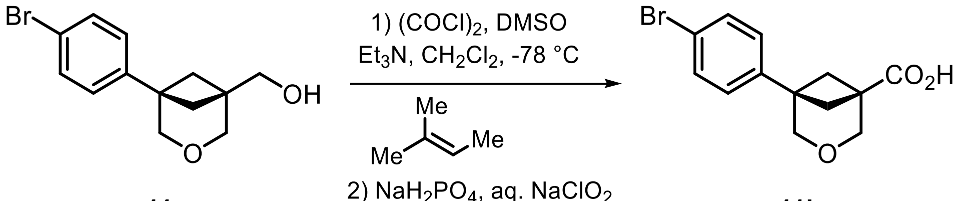

11a **11b**

5-(4-Bromophenyl)-3-oxabicyclo[3.1.1]heptane-1-carboxylic acid (11b)

The same procedure as for **1b** was used. The final product was purified by crystallization from hexane. Yield: 38.02 g, 0.128 mol, 87%, yellow solid, m.p. = 142-143 °C. ^{1}H NMR (500 MHz, DMSO-d_6): δ 12.60 (s, 1H), 7.51 (d, J = 8.3 Hz, 2H), 7.11 (d, J = 8.3 Hz, 2H), 3.95 (s, 2H), 3.73 (s, 2H), 2.30 (d, J = 6.2 Hz, 2H), 2.19 (dd, J = 6.2, 2.0 Hz, 2H) ppm. ^{13}C{^{1}H} NMR (126 MHz, DMSO-d_6): δ 173.4, 143.3, 131.2, 127.7, 119.7, 74.1, 69.2, 42.5, 42.4, 38.0 ppm. LCMS (M - H)$^-$: 296. HRMS (ESI-TOF) *m/z*: [M - H]$^-$ calcd for $C_{13}H_{12}BrO_3^-$: 294.9975; found: 294.9971.

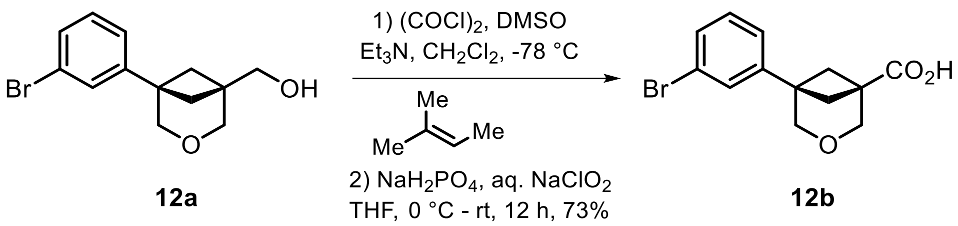

12a **12b**

5-(3-Bromophenyl)-3-oxabicyclo[3.1.1]heptane-1-carboxylic acid (12b)

The same procedure as for **1b** was used. The final product was purified by crystallization from hexane. Yield: 32.18 g, 0.108 mol, 73%, white solid, m.p. = 100-101 °C. ^{1}H NMR (500 MHz, DMSO-d_6): δ 12.61 (s, 1H), 7.43 (d, J = 7.2 Hz, 1H), 7.32 (s, 1H), 7.28 (t, J = 7.7 Hz, 1H), 7.16 (d, J = 7.2 Hz, 1H), 3.95 (s, 2H), 3.75 (s, 2H), 2.32 (d, J = 4.9 Hz, 2H), 2.20 (d, J = 4.0 Hz, 2H) ppm. ^{13}C{^{1}H} NMR (151 MHz, DMSO-d_6): δ 173.4, 146.7, 130.6, 129.5, 128.2, 124.5, 121.8, 74.1, 69.2, 42.7, 42.5, 38.0 ppm. LCMS (M - H)$^-$: 297. HRMS (ESI-TOF) *m/z*: [M - H]$^-$ calcd for $C_{13}H_{12}BrO_3^-$: 294.9975; found: 294.9970.

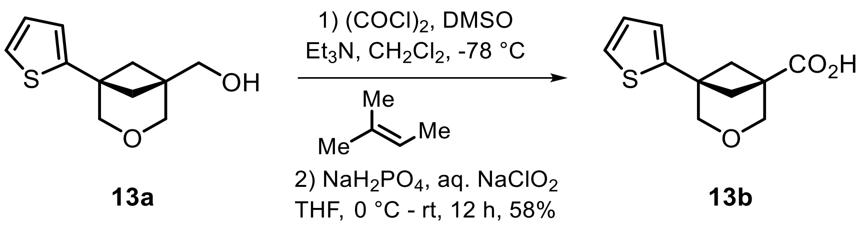

5-(Thiophen-2-yl)-3-oxabicyclo[3.1.1]heptane-1-carboxylic acid (13b)

The same procedure as for **1b** was used. The final product was purified by crystallization from a mixture of hexane/toluene, 4:1. Yield: 19.04 g, 0.085 mol, 58%, beige solid, m.p. = 74-75 °C. ^{1}H NMR (500 MHz, DMSO-*d*$_6$): δ 12.68 (s, 1H), 7.42 (d, *J* = 4.9 Hz, 1H), 6.98 (dd, *J* = 4.7, 3.6 Hz, 1H), 6.89 (d, *J* = 2.6 Hz, 1H), 3.95 (s, 2H), 3.90 (s, 2H), 2.42 (d, *J* = 6.2 Hz, 2H), 2.16 (dd, *J* = 6.2, 2.2 Hz, 2H) ppm. ^{13}C{^{1}H} NMR (126 MHz, DMSO-*d*$_6$): δ 173.3, 147.0, 127.2, 124.3, 123.1, 73.5, 69.1, 42.6, 40.6 ppm. LCMS (M - H)$^-$: 223. HRMS (ESI-TOF) *m/z*: [M - H]$^-$ calcd for $C_{11}H_{11}O_3S^-$: 223.0434; found: 223.0435.

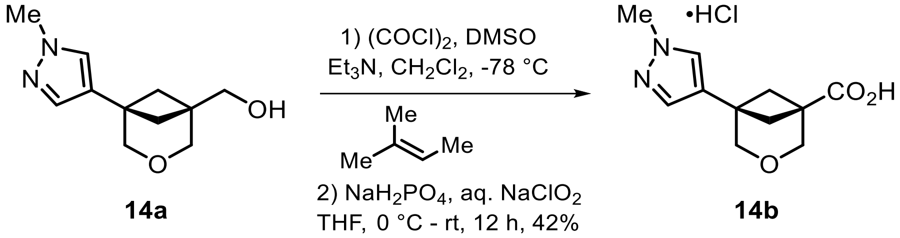

5-(1-Methyl-1*H*-pyrazol-4-yl)-3-oxabicyclo[3.1.1]heptane-1-carboxylic acid hydrochloride (14b)

To a stirred solution of oxalyl chloride (19 mL, 0.19 mol, 1.30 equiv) in 2 L of CH_2Cl_2 was added a solution of DMSO (16 mL, 0.22 mol, 1.50 equiv) in 100 mL of CH_2Cl_2 dropwise at -78 °C. The resulting mixture was stirred at the same temperature for 1 h, and the solution of 5-(1-methyl-1*H*-pyrazol-4-yl)-3-oxabicyclo[3.1.1]heptan-1-yl)methanol (30.58 g, 0.147 mol, 1.00 equiv) in 150 mL of CH_2Cl_2 was added dropwise. The mixture was stirred for 4 h at -60 °C and Et_3N (82 mL, 0.59 mol, 4.00 equiv) was added. The resulting solution was slowly warmed and stirred at room temperature overnight. The organic layer was washed with water (2 × 300 mL), dried over Na_2SO_4, filtered, and evaporated to dryness under reduced pressure. The residue was dissolved in 1 L of THF and 2-methylbut-2-ene (48 mL, 0.45 mol, 3.06 equiv) and NaH_2PO_4 (21.60 g, 0.18 mol, 1.22 equiv) in 1 L of water were added. Then a solution of $NaClO_2$ (16.20 g, 0.18 mol, 1.22 equiv) in 100 mL of water was added to the mixture dropwise at 0 °C. The reaction mixture was slowly warmed to room temperature and stirred overnight. The organic layer was separated and concentrated under reduced pressure. The residue was heated in *i*-PrOH (200 mL) under reflux and filtered (3 times). The combined filtrates were concentrated under reduced pressure. The residue was diluted with water (100 mL), acidified with 10M HCl to pH ~ 4, and concentrated under

reduced pressure. The final product was recrystallized from *i*-PrOH. Yield: 16.00 g, 0.0617 mol, 42%, beige solid, m.p. = 139-140 °C. ^{1}H NMR (500 MHz, DMSO-d_6): δ 8.75 (br s, 2H), 7.62 (s, 1H), 7.39 (s, 1H), 3.91 (s, 2H), 3.81 (s, 2H), 3.78 (s, 3H), 2.31 (d, J = 6.2 Hz, 2H), 2.00 (dd, J = 6.2, 1.9 Hz, 2H) ppm. ^{13}C{^{1}H} NMR (151 MHz, DMSO-d_6): δ 173.7, 135.4, 128.0, 123.9, 73.0, 69.3, 42.8, 38.4, 35.8 ppm. LCMS (M + H)$^+$: 223. HRMS (ESI-TOF) *m/z*: [M + H]$^+$ calcd for $C_{11}H_{15}N_2O_3^+$: 223.1077; found: 223.1078.

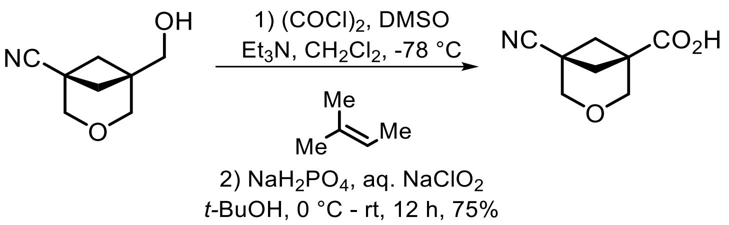

5-Cyano-3-oxabicyclo[3.1.1]heptane-1-carboxylic acid (17b)

To a stirred solution of oxalyl chloride (68 mL, 0.80 mol, 1.60 equiv) in 2 L of CH_2Cl_2 was added a solution of DMSO (57 mL, 0.80 mol, 1.60 equiv) in 100 mL of CH_2Cl_2 dropwise at -78 °C. The resulting mixture was stirred at the same temperature for 1 h, and the solution of 5-(hydroxymethyl)-3-oxabicyclo[3.1.1]heptane-1-carbonitrile (77.00 g, 0.50 mol, 1.00 equiv) in 150 mL of CH_2Cl_2 was added dropwise. The mixture was stirred for 4 h at -60 °C and Et_3N (348 mL, 2.50 mol, 5.00 equiv) was added. The resulting solution was slowly warmed and stirred at room temperature overnight. The solution was concentrated under reduced pressure, diluted with 2 L of EtOAc, filtered throught a pad of celite, and concentrated under reduced pressure. To a stirred solution of 5-formyl-3-oxabicyclo[3.1.1]heptane-1-carbonitrile (60.00 g, 0.40 mol, 1.00 equiv), 2-methylbut-2-ene (211 mL, 4.00 mol, 10.0 equiv) and NaH_2PO_4 (60.00 g, 0.50 mol, 1.25 equiv) in a mixture of 1.2 L of water and 1.2 L of *t*-BuOH was added a solution of $NaClO_2$ (45.00 g, 0.50 mol, 1.25 equiv) in 100 mL of water dropwise at 0 °C (ice-water bath). The reaction mixture was slowly warmed to room temperature and stirred for 4 h. The solution was diluted with water (2 L), acidified with $NaHSO_4$ to pH = 4, and extracted with EtOAc (2 × 1 L). The combined organic layers were washed with water (2 × 1 L), brine (1 × 500 mL), dried over Na_2SO_4, filtered, and evaporated to dryness under reduced pressure. The residue was diluted with hexane (500 mL) and left in the fridge overnight. The precipitate was filtered, washed with hexane (100 mL), and dried. Additionally, the product can be recrystallized from hexane. Yield over 2 steps: 50.00 g, 0.30 mol, 60%, white solid, m.p. = 145-146 °C. ^{1}H NMR (500 MHz, DMSO-d_6): δ 12.75 (br s, 1H), 3.96 (s, 1H), 3.89 (s, 1H), 2.69 – 2.64 (m, 2H), 2.21 – 2.14 (m, 2H) ppm. ^{13}C{^{1}H} NMR (126 MHz, DMSO-d_6): δ 215.3, 171.9, 119.7, 68.8, 68.3, 44.0, 37.7, 29.5 ppm. HRMS (ESI-TOF) *m/z*: [M - H]$^-$ calcd for $C_8H_8NO_3^-$: 166.0510; found: 166.0512.

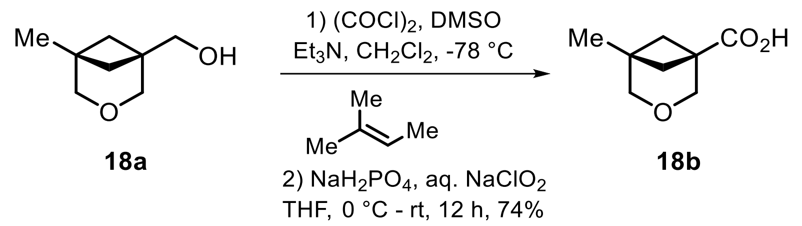

5-Methyl-3-oxabicyclo[3.1.1]heptane-1-carboxylic acid (18b)

To a stirred solution of oxalyl chloride (27 mL, 0.32 mol, 1.30 equiv) in 2 L of CH_2Cl_2 was added a solution of DMSO (26 mL, 0.37 mol, 1.51 equiv) in 100 mL of CH_2Cl_2 dropwise at -78 °C. The resulting mixture was stirred at the same temperature for 1 h, and the solution of (5-methyl-3-oxabicyclo[3.1.1]heptan-1-yl)methanol (35.00 g, 0.245 mol, 1.00 equiv) in 150 mL of CH_2Cl_2 was added dropwise. The mixture was stirred for 4 h at -60 °C and Et_3N (139 mL, 1.00 mol, 4.00 equiv) was added. The resulting solution was slowly warmed and stirred at room temperature overnight. The organic layer was washed with water (2 × 300 mL), dried over Na_2SO_4, filtered, and evaporated to dryness under reduced pressure. The residue was dissolved in 1 L of THF and 2-methylbut-2-ene (79 mL, 0.75 mol, 3.06 equiv) and NaH_2PO_4 (34.00 g, 0.30 mol, 1.22 equiv) in 1 L of water were added. Then a solution of $NaClO_2$ (29.00 g, 0.30 mol, 1.22 equiv) in 100 mL of water was added to the mixture dropwise at 0 °C. The reaction mixture was slowly warmed to room temperature and stirred overnight. The mixture was concentrated under reduced pressure. The residue was diluted with water and acidified with aq. conc. HCl to pH ~ 5. The solution was extracted with EtOAc (3 × 200 mL). The combined organic layers were washed with brine (1 × 100 mL), dried over Na_2SO_4, filtered, and concentrated under reduced pressure. The final product was purified by crystallization from hexane. Yield: 28.00 g, 0.18 mol, 74%, white solid, m.p. = 65-66 °C. ^1H NMR (500 MHz, DMSO-d_6): δ 12.40 (br s, 1H), 3.82 (s, 2H), 3.55 (s, 2H), 1.96 (d, *J* = 6.0 Hz, 2H), 1.71 (dd, *J* = 6.0, 2.3 Hz, 2H), 0.94 (s, 3H) ppm. $^{13}C\{^1H\}$ NMR (126 MHz, DMSO-d_6): δ 173.8, 74.2, 69.2, 42.8, 35.8, 21.7 ppm. HRMS (ESI-TOF) *m/z*: $[M - H]^-$ calcd for $C_8H_{11}O_3^-$: 155.0714; found: 155.0715.

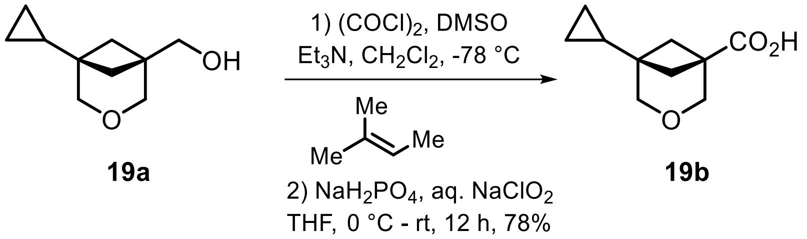

5-Cyclopropyl-3-oxabicyclo[3.1.1]heptane-1-carboxylic acid (19b)

The same procedure as for **18b** was used. The final product was purified by crystallization from hexane. Yield: 34.50 g, 0.19 mol, 78%, white solid. ^1H NMR (500 MHz, DMSO-d_6): δ 12.43 (s, 1H), 3.83 (s, 2H), 3.67 (s, 2H), 1.78 (d, *J* = 6.2 Hz, 2H), 1.54 (dd, *J* = 6.2, 2.3 Hz, 2H), 0.72 – 0.61 (m, 1H), 0.33 – 0.26 (m, 2H), 0.14 (q, *J* = 5.0 Hz, 2H). $^{13}C\{^1H\}$ NMR (126 MHz, DMSO-d_6): δ

173.8, 73.9, 69.8, 42.0, 35.4, 13.8, -0.2 ppm. HRMS (ESI-TOF) *m/z*: [M - H]$^-$ calcd for $C_{10}H_{13}O_3^-$: 181.0870; found: 181.0870.

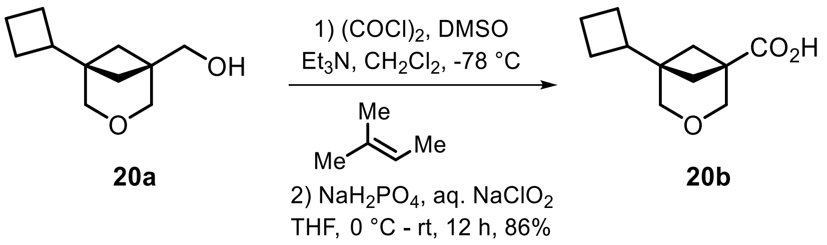

5-Cyclobutyl-3-oxabicyclo[3.1.1]heptane-1-carboxylic acid (20b)

The same procedure as for **18b** was used. The final product was purified by crystallization from hexane. Yield: 41.16 g, 0.21 mol, 86%, yellow solid. ^{1}H NMR (500 MHz, DMSO-*d$_6$*): δ 12.47 (s, 1H), 3.84 (s, 2H), 3.50 (s, 2H), 2.21 – 2.10 (m, 1H), 2.04 (d, *J* = 5.5 Hz, 2H), 1.86 – 1.62 (m, 6H), 1.58 – 1.41 (m, 2H) ppm. ^{13}C{^{1}H} NMR (126 MHz, DMSO-*d$_6$*): δ 174.0, 71.8, 69.8, 42.0, 41.4, 37.3, 33.7, 22.2, 17.4 ppm. HRMS (ESI-TOF) *m/z*: [M - H]$^-$ calcd for $C_{11}H_{15}O_3^-$: 195.1027; found: 195.1025.

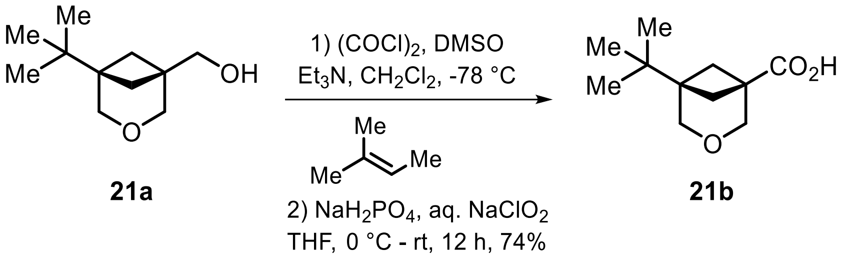

5-(*tert*-Butyl)-3-oxabicyclo[3.1.1]heptane-1-carboxylic acid (21b)

The same procedure as for **18b** was used. The final product was purified by crystallization from hexane. Yield: 35.28 g, 0.18 mol, 74%, white solid, m.p. = 137-138 °C. ^{1}H NMR (500 MHz, CDCl$_3$): δ 3.96 (s, 2H), 3.84 (s, 2H), 2.30 (d, *J* = 8.2 Hz, 2H), 1.70 (dd, *J* = 6.6, 2.2 Hz, 2H), 0.83 (s, 9H) ppm. ^{13}C{^{1}H} NMR (151 MHz, CDCl$_3$): δ 179.3, 71.8, 69.9, 45.8, 41.6, 34.0, 32.1, 25.2 ppm. HRMS (ESI-TOF) *m/z*: [M - H]$^-$ calcd for $C_{11}H_{17}O_3^-$: 197.1183; found: 197.1185.

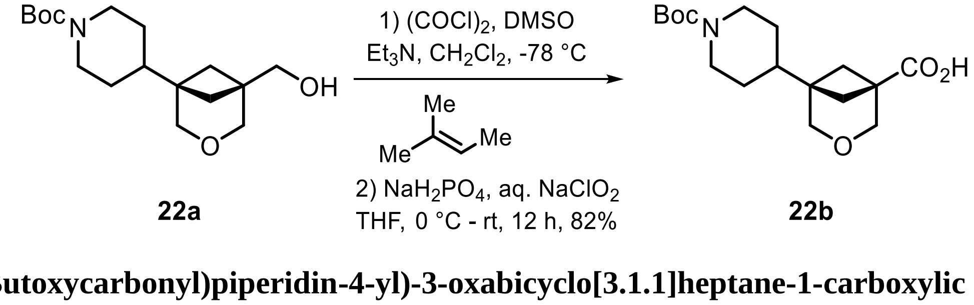

5-(1-(*tert*-Butoxycarbonyl)piperidin-4-yl)-3-oxabicyclo[3.1.1]heptane-1-carboxylic acid (22b)

The same procedure as for **18b** was used. The final product was purified by crystallization from hexane. Yield: 29.25 g, 0.09 mol, 82%, white solid, m.p. = 116-117 °C. ^{1}H NMR (500 MHz,

$CDCl_3$): δ 10.00 (br s, 1H), 4.15 (br s, 2H), 3.99 (s, 2H), 3.75 (s, 2H), 2.58 (t, *J* = 11.3 Hz, 2H), 2.14 (d, *J* = 6.8 Hz, 2H), 1.81 (d, *J* = 6.8 Hz, 2H), 1.49 (d, *J* = 12.6 Hz, 2H), 1.39 (s, 9H), 1.37 – 1.29 (m, 1H), 1.11 (qd, *J* = 12.5, 3.8 Hz, 2H) ppm. $^{13}C\{^1H\}$ NMR (151 MHz, $CDCl_3$): δ 178.1, 154.9, 79.79 (s), 72.3, 70.1, 44.2 (br s), 42.8, 42.5, 41.7, 36.4, 28.6, 26.6 ppm. LCMS $(M - H)^-$: 324. HRMS (ESI-TOF) *m/z*: $[M – tBu + 2H]^+$ calcd for $C_{13}H_{20}NO_5^+$: 270.1336; found: 270.1337.

3.4. Radical modifications

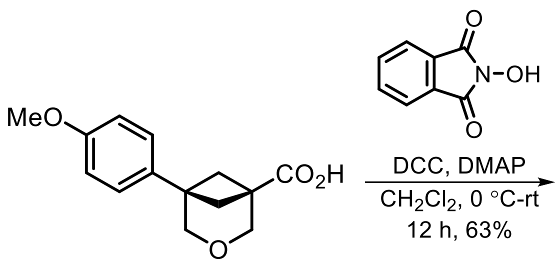

1,3-Dioxoisoindolin-2-yl 5-(4-methoxyphenyl)-3-oxabicyclo[3.1.1]heptane-1-carboxylate (23)

To a stirred solution of 5-(4-methoxyphenyl)-3-oxabicyclo[3.1.1]heptane-1-carboxylic acid (10.00 g, 0.04 mol, 1.00 equiv), DMAP (0.50 g, 0.004 mol) and 2-hydroxy-1*H*-isoindole-1,3(2*H*)-dione (6.52 g, 0.04 mol, 1.00 equiv) at 0 °C in 500 mL of CH_2Cl_2 was added *N,N'*-dicyclohexylcarbodiimide (DCC) (9.30 g, 0.045 mol, 1.13 equiv) in portions. The mixture was slowly warmed to room temperature and stirred overnight. The solution was filtered and the organic layer was washed with water and concentrated under reduced pressure. The final product was recrystallized from MeO*t*Bu. Yield: 10.00 g, 0.025 mol, 63%, white solid, m.p. = 125-126 °C. ^{1}H NMR (500 MHz, $CDCl_3$): δ 7.89 (dd, *J* = 5.4, 3.1 Hz, 2H), 7.79 (dd, *J* = 5.4, 3.1 Hz, 2H), 7.04 (d, *J* = 8.5 Hz, 2H), 6.88 (d, *J* = 8.5 Hz, 2H), 4.28 (s, 2H), 3.92 (s, 2H), 3.80 (s, 3H), 2.75 (d, *J* = 7.9 Hz, 2H), 2.48 (dd, *J* = 6.4, 2.3 Hz, 2H) ppm. ^{13}C{^{1}H} NMR (126 MHz, $CDCl_3$): δ 168.5, 161.9, 158.7, 135.4, 134.9, 129.0, 126.5, 124.1, 114.1, 75.9, 69.2, 55.5, 43.5, 42.0, 39.0 ppm. HRMS (ESI-TOF) *m/z*: $[M + NH_4]^+$ calcd for $C_{22}H_{23}N_2O_6^+$: 411.1551; found: 411.1546.

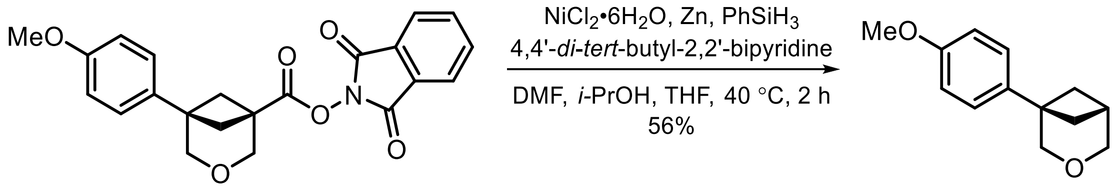

1-(4-Methoxyphenyl)-3-oxabicyclo[3.1.1]heptane (23a)

A two-neck round bottom flask was charged with $NiCl_2•6H_2O$ (0.06 g, 0.25 mmol, 10 mol%) and 4,4'-*di-tert*-butyl-2,2'-bipyridine (0.16 g, 0.50 mmol, 20 mol%). The flask was equipped with a reflux condenser and rubber septum, and the apparatus was purged with Ar from a balloon for 5 min. DMF (2.3 mL, anhydrous) was added, and the mixture was stirred for 10 min. THF (10 mL) and *i*-PrOH (1.2 mL) were then added, followed by 1,3-dioxoisoindolin-2-yl 5-(4-methoxyphenyl)-3-oxabicyclo[3.1.1]heptane-1-carboxylate (1.00 g, 2.50 mmol, 1.00 equiv) and Zn powder (0.082 g, 1.26 mmol, 0.50 equiv). Immediately following the addition of the Zn powder, $PhSiH_3$ (neat, 0.462 mL, 3.75 mmol, 1.50 equiv) was added dropwise. Upon completion of the addition of $PhSiH_3$, the

reaction mixture was placed in a preheated 40 °C oil bath and stirred for 2 h. After placing the flask in the oil bath, an additional 2 mL of THF was used to rinse the sides of the flask. After 2 h the mixture was allowed to cool to ambient temperature. H_2O (distilled) and a sat. aq. solution of NH_4Cl was added (1:1 v/v), and the mixture was transferred to a separatory funnel. The mixture was extracted with EtOAc, and the organic extracts were filtered over a small plug of silica gel. The filtrate was concentrated on a rotary evaporator under reduced pressure at 40 °C, and the crude product was purified by flash column chromatography (SiO_2, gradient, hexane/MeO*t*Bu). Yield: 284 mg, 1.40 mmol, 56%, yellow oil. ^{1}H NMR (500 MHz, $CDCl_3$): δ 7.02 (d, *J* = 8.5 Hz, 2H), 6.85 (d, *J* = 8.5 Hz, 2H), 4.00 (s, 2H), 3.88 (s, 2H), 3.79 (s, 3H), 2.41 (t, *J* = 6.3 Hz, 1H), 2.23 – 2.15 (m, 2H), 2.08 – 1.99 (m, 2H) ppm. ^{13}C{^{1}H} NMR (151 MHz, $CDCl_3$): δ 158.2, 137.9, 126.6, 113.9, 77.3, 70.4, 55.4, 46.6, 35.7, 31.2 ppm. LCMS $(M + H)^+$: 205. HRMS (ESI-TOF) *m/z*: $[M + H]^+$ calcd for $C_{13}H_{17}O_2^+$: 205.1223; found: 205.1216.

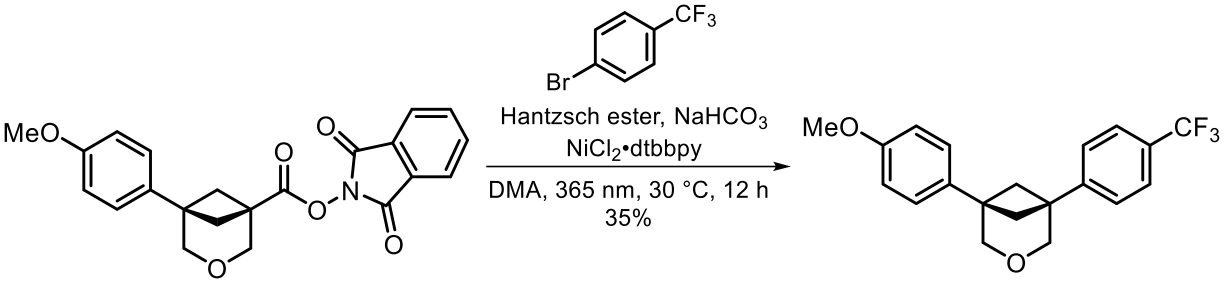

1-(4-Methoxyphenyl)-5-(4-(trifluoromethyl)phenyl)-3-oxabicyclo[3.1.1]heptane (23b)

To a mixture of dry reagents 1,3-dioxoisoindolin-2-yl 5-(4-methoxyphenyl)-3-oxabicyclo[3.1.1]heptane-1-carboxylate (1.00 g, 2.50 mmol, 1.00 equiv), Hantzsch ester (1.30 g, 5.00 mmol, 2.00 equiv), $NaHCO_3$ (0.84 g, 10.00 mmol, 4.00 equiv) and $NiCl_2$·dtbbpy (0.50 g, 1.25 mmol, 0.50 equiv) was added DMA (15 mL) followed by 1-bromo-4-(trifluoromethyl)benzene (0.84 g, 3.75 mmol, 1.50 equiv). The resulting solution was bubbled by argon through the solution for 5 min, then the reaction mixture was irradiated with 365 nm overnight maintaining the temperature at 30 °C. Then it was poured to water (30 mL) and extracted with EtOAc (3 × 10 mL). The combined organic layers were washed with water (2 × 10 mL), brine (1 × 10 mL), dried over Na_2SO_4, filtered, and evaporated to dryness. The final product was purified by flash column chromatography (SiO_2, gradient, hexane/MeO*t*Bu). Yield: 305 mg, 0.875 mmol, 35%, white solid. ^{1}H NMR (400 MHz, DMSO-d_6): δ 7.69 (d, *J* = 8.1 Hz, 2H), 7.41 (d, *J* = 8.1 Hz, 2H), 7.11 (d, *J* = 8.5 Hz, 2H), 6.89 (d, *J* = 8.5 Hz, 2H), 3.84 (s, 2H), 3.82 (s, 2H), 3.72 (s, 3H), 3.30 (s, 2H), 2.49 – 2.47 (m, 2H), 2.19 (d, *J* = 8.0 Hz, 2H) ppm. ^{13}C{^{1}H} NMR (151 MHz, DMSO-d_6): δ 157.9, 149.1, 136.2, 127.1 (q, *J* = 32 Hz), 126.5, 126.4, 125.2 (q, *J* = 4 Hz), 122.5 (q, *J* = 272 Hz), 113.8, 74.9, 74.2, 55.0, 42.9, 42.2 ppm. ^{19}F{^{1}H} NMR (376 MHz, DMSO-d_6): δ -62.9 (s) ppm. LCMS $(M + H)^+$: 349. HRMS (ESI-TOF) *m/z*: $[M + H]^+$ calcd for $C_{20}H_{20}F_3O_2^+$: 349.1410; found: 349.1410.

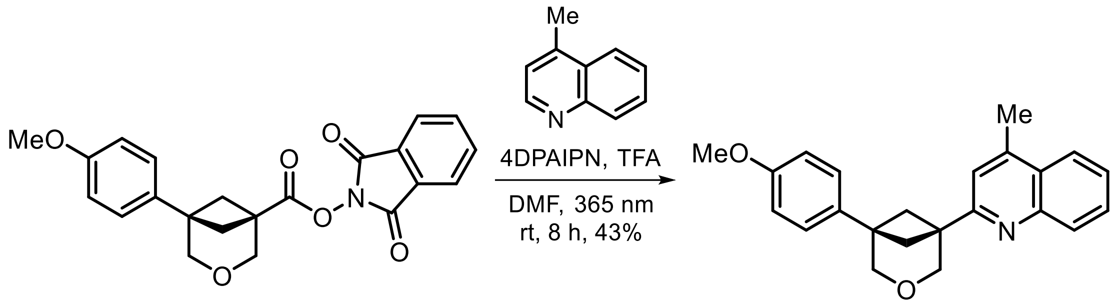

2-(5-(4-Methoxyphenyl)-3-oxabicyclo[3.1.1]heptan-1-yl)-4-methylquinoline (23c)

To a stirred solution of 4-methylquinoline (1.00 g, 7.50 mmol, 3.00 equiv) in 4 mL of DMF was added TFA (0.383 mL, 5 mmol, 2.00 equiv) at room temperature. The resulting mixture was stirred for 10 min, and then 1,3-dioxoisoindolin-2-yl 5-(4-methoxyphenyl)-3-oxabicyclo[3.1.1]heptane-1-carboxylate (1.00 g, 2.50 mmol, 1.00 equiv) was added followed by 2,4,5-*tris*(diphenylamino)isophthalonitrile (4DPAIPN) (0.10 g, 0.125 mmol, 0.05 equiv). The reaction mixture was stirred under the irradiation of a 36 W Blue Leds, 365 nm, at room temperature for 8 h. After this, the mixture was diluted with water (8 mL) and extracted with EtOAc (3 × 3 mL). The combined organic layers were washed with brine (1 × 2 mL), dried over Na_2SO_4, filtered, and evaporated to dryness. The final product was purified by flash column chromatography (SiO_2, gradient, hexane/MeO*t*Bu). Yield: 371 mg, 1.075 mmol, 43%, white solid. ^{1}H NMR (600 MHz, DMSO-*d*$_6$): δ 8.04 (d, *J* = 8.2 Hz, 1H), 7.95 (d, *J* = 8.2 Hz, 1H), 7.72 (t, *J* = 7.5 Hz, 1H), 7.57 (t, *J* = 7.5 Hz, 1H), 7.38 (s, 1H), 7.13 (d, *J* = 8.6 Hz, 2H), 6.89 (d, *J* = 8.6 Hz, 2H), 4.11 (s, 2H), 3.85 (s, 2H), 3.72 (s, 3H), 2.67 (s, 3H), 2.48 (dd, *J* = 6.3, 2.2 Hz, 2H), 2.41 (d, *J* = 7.8 Hz, 2H) ppm. ^{13}C{^{1}H} NMR (151 MHz, DMSO-*d*$_6$): δ 162.3, 157.8, 146.9, 144.7, 136.6, 129.3, 129.1, 126.5, 126.5, 125.9, 124.0, 119.0, 113.8, 75.2, 72.9, 55.0, 45.0, 42.1, 18.2 ppm. HRMS (ESI-TOF) *m/z*: [M + H]$^+$ calcd for $C_{23}H_{24}NO_2^+$: 346.1802; found: 346.1801.

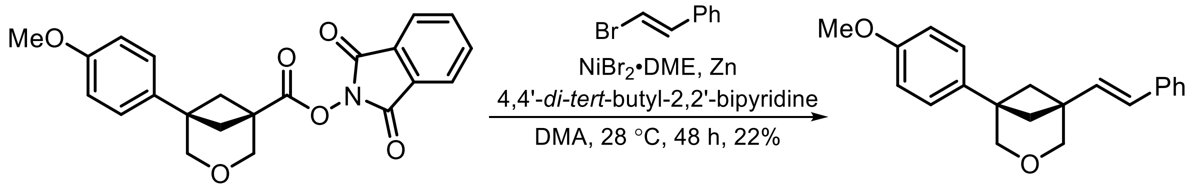

1-(4-Methoxyphenyl)-5-styryl-3-oxabicyclo[3.1.1]heptane (23d)

A vial containing a stir bar was charged with 4,4'-*di-tert*-butyl-2,2'-bipyridine (0.08 g, 0.30 mmol, 12 mol%) was brought into a N_2 filled glovebox and was charged with $NiBr_2$·DME (64 mg, 0.25 mmol, 10 mol%), and DMA (3 mL) and allowed to stir for 10 min. Then was added 1,3-dioxoisoindolin-2-yl 5-(4-methoxyphenyl)-3-oxabicyclo[3.1.1]heptane-1-carboxylate (1.00 g, 2.50 mmol, 1.00 equiv), (2-bromovinyl)benzene (675 mg, 3.75 mmol, 1.50 equiv) and Zn powder (328 mg, 5.00 mmol, 2.00 equiv). The vial was then sealed with a teflon-lined cap, removed from the

glovebox, placed in a pre-heated oil bath (28 °C), and allowed to stir for 48 h. Once the reaction was complete, the reaction mixture was diluted with Et_2O (3 mL) and passed through a short silica plug. Then a solution was concentrated under reduced pressure. The final product was purified by flash column chromatography (SiO_2, gradient, hexane/MeO*t*Bu). Yield: 168 mg, 0.55 mmol, 22%, yellow oil. A mixture ~ 5:1. ^{1}H NMR (500 MHz, $CDCl_3$): δ 7.35 (d, *J* = 7.4 Hz, 2H), 7.30 (t, *J* = 7.4 Hz, 2H), 7.22 (t, *J* = 7.1 Hz, 1H), 7.07 (d, *J* = 8.5 Hz, 2H), 6.87 (d, *J* = 8.5 Hz, 2H), 6.32 (d, *J* = 16.0 Hz, 1H), 6.22 (d, *J* = 16.0 Hz, 1H), 3.91 (s, 2H), 3.89 (s, 2H), 3.80 (s, 3H), 2.26 (d, *J* = 8.0 Hz, 2H), 2.19 (d, *J* = 6.4 Hz, 2H) ppm. ^{13}C{^{1}H} NMR (151 MHz, $CDCl_3$): δ 158.3, 137.2, 131.9, 129.3, 128.7, 127.5, 126.7, 126.3, 114.0, 76.5, 73.9, 55.4, 42.8, 41.9, 40.1, 35.7 ppm. LCMS $(M + H)^+$: 307. HRMS (ESI-TOF) *m/z*: $[M + H]^+$ calcd for $C_{21}H_{23}O_2^+$: 307.1693; found: 307.1682.

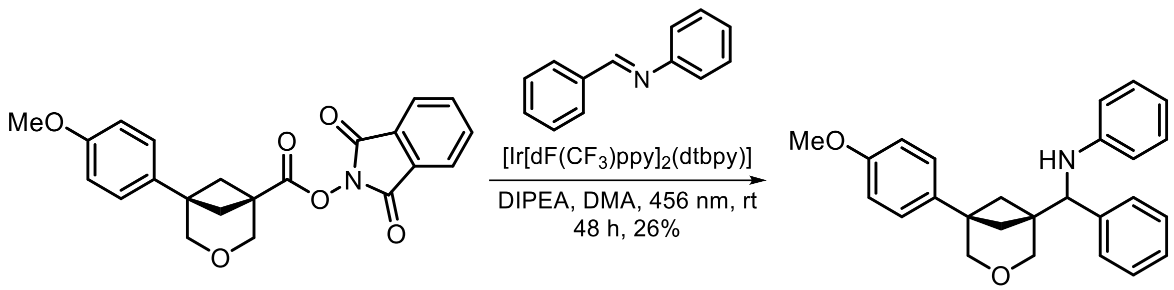

***N*-((5-(4-Methoxyphenyl)-3-oxabicyclo[3.1.1]heptan-1-yl)(phenyl)methyl)aniline (23e)**

To a flask equipped with a magnetic stir bar and a rubber septum was added [Ir[dF(CF_3)ppy]$_2$(dtbpy)] (126 mg, 0.125 mmol, 0.02 equiv), 1,3-dioxoisoindolin-2-yl 5-(4-methoxyphenyl)-3-oxabicyclo[3.1.1]heptane-1-carboxylate (1.00 g, 2.50 mmol, 1.00 equiv), (*E*)-*N*,1-diphenylmethanimine (0.91 g, 5.00 mmol, 2.00 equiv) in DMA (20 mL). DIPEA (1.3 mL, 7.50 mmol, 3.00 equiv) was then added via syringe under an argon atmosphere. Argon is then bubbled in the solution for 5 min. The vial was sealed with Parafilm and irradiated with a 456 nm LED lamp (300 W) for 48 h. Then it was poured into water (30 mL) and extracted with EtOAc (3 × 10 mL). The combined organic layers were washed with brine (1 × 10 mL), dried over Na_2SO_4, filtered, and evaporated to dryness. The final product was purified by flash column chromatography (SiO_2, gradient, hexane/MeO*t*Bu). Yield: 250 mg, 0.65 mmol, 26%, white solid. ^{1}H NMR (400 MHz, DMSO-d_6): δ 7.35 – 7.24 (m, 4H), 7.22 – 7.13 (m, 1H), 7.00 (d, *J* = 8.6 Hz, 2H), 6.94 (t, *J* = 7.8 Hz, 2H), 6.85 (d, *J* = 8.6 Hz, 2H), 6.58 (d, *J* = 7.8 Hz, 2H), 6.44 (t, *J* = 7.2 Hz, 1H), 5.98 (d, *J* = 8.1 Hz, 1H), 4.36 (d, *J* = 8.0 Hz, 1H), 3.81 (d, *J* = 9.3 Hz, 1H), 3.70 (s, 3H), 3.69 (d, *J* = 9.3 Hz, 3H), 3.63 (s, 2H), 2.12 (d, *J* = 8.2 Hz, 1H), 2.03 – 1.89 (m, 3H) ppm. ^{13}C{^{1}H} NMR (151 MHz, DMSO-d_6): δ 157.7, 148.0, 140.6, 137.0, 128.5, 128.0, 127.2, 126.7, 126.4, 115.7, 113.7, 113.0, 75.7, 71.2, 60.4, 55.0, 42.5, 42.1, 37.1, 36.7 ppm. LCMS $(M + H)^+$: 386. HRMS (ESI-TOF) *m/z*: $[M + H]^+$ calcd for $C_{26}H_{28}NO_2^+$: 386.2115; found: 386.2097.

3.5. Modifications

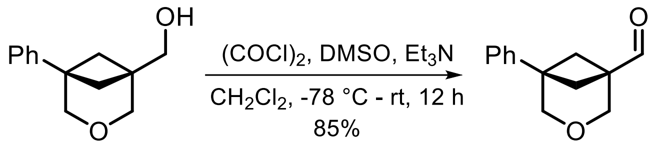

5-Phenyl-3-oxabicyclo[3.1.1]heptane-1-carbaldehyde (24)

To a stirred solution of oxalyl chloride (27 mL, 0.32 mol, 1.30 equiv) in 2 L of CH_2Cl_2 was added a solution of DMSO (26 mL, 0.37 mol, 1.51 equiv) in 100 mL of CH_2Cl_2 dropwise at -78 °C. The resulting mixture was stirred at the same temperature for 1 h, and the solution of (5-phenyl-3-oxabicyclo[3.1.1]heptan-1-yl)methanol (50.00 g, 0.245 mol, 1.00 equiv) in 150 mL of CH_2Cl_2 was added dropwise. The mixture was stirred for 4 h at -60 °C and Et_3N (139 mL, 1.00 mol, 4.00 equiv) was added. The resulting solution was slowly warmed and stirred at room temperature overnight. The organic layer was washed with water (2 × 300 mL), dried over Na_2SO_4, filtered, and evaporated to dryness under reduced pressure. The final product was purified by distillation (0.4 mbar, b.p. = 109-110 °C). Yield: 42.00 g, 0.208 mol, 85%, yellow oil. ^{1}H NMR (400 MHz, DMSO-d_6): δ 9.50 (s, 1H), 7.34 (t, J = 7.5 Hz, 2H), 7.24 (t, J = 7.2 Hz, 1H), 7.17 (d, J = 7.4 Hz, 2H), 4.01 (s, 2H), 3.81 (s, 2H), 2.40 (d, J = 8.1 Hz, 2H), 2.14 (dd, J = 6.3, 2.4 Hz, 2H) ppm. ^{13}C{^{1}H} NMR (126 MHz, $CDCl_3$): δ 200.5, 143.8, 128.7, 127.1, 125.3, 76.7, 68.4, 49.2, 44.8, 36.5 ppm. GCMS $(M)^+$: 202. HRMS (ESI-TOF) *m/z*: $[M + H]^+$ calcd for $C_{13}H_{15}O_2^+$: 203.1067; found: 203.1059.

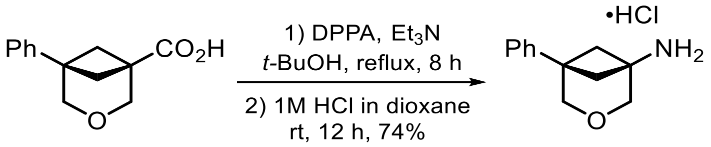

5-Phenyl-3-oxabicyclo[3.1.1]heptan-1-amine hydrochloride (25)

A mixture of 5-phenyl-3-oxabicyclo[3.1.1]heptane-1-carboxylic acid (26.00 g, 0.12 mol, 1.00 equiv), DPPA (30 mL, 0.14 mol, 1.17 equiv), Et_3N (20 mL, 0.14 mol, 1.14 equiv) in *t*-BuOH was heated under reflux (in an oil bath with a thermocouple) for 8 h. The solution was concentrated under reduced pressure, diluted with water (200 mL), alkalized with NaOH to pH = 9, and extracted with EtOAc (3 × 200 mL). The combined organic layers were washed with brine (1 × 200 mL), dried over Na_2SO_4, filtered, and evaporated to dryness under reduced pressure. The crude product was dissolved in MeO*t*Bu and 250 mL of 1M HCl in dioxane was added. The reaction mixture was stirred at room temperature overnight and filtered. The obtained precipitate was washed with MeO*t*Bu (100 mL) and dried. Yield: 20.07 g, 0.089 mol, 74%, beige solid, m.p. = 161-162 °C. ^{1}H NMR (500 MHz, DMSO-d_6): δ 8.70 (br s, 3H), 7.34 (t, J = 7.5 Hz, 2H), 7.26 (t, J = 7.3 Hz, 1H), 7.17 (d, J = 7.1 Hz, 2H), 3.90 (s, 2H), 3.72 (s, 2H), 2.40 – 2.28 (m, 4H) ppm. ^{13}C{^{1}H} NMR (151

MHz, DMSO-d_6): δ 142.7, 128.5, 126.7, 125.5, 74.0, 69.2, 50.4, 42.5 ppm. LCMS $(M + H)^+$: 190. HRMS (ESI-TOF) *m/z*: $[M + H]^+$ calcd for $C_{12}H_{16}NO^+$: 190.1226; found: 190.1223.

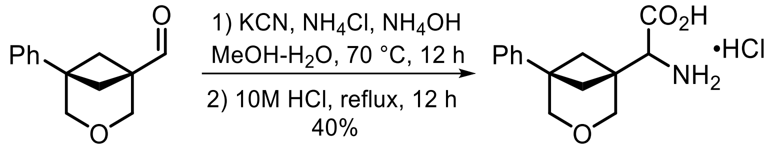

2-Amino-2-(5-phenyl-3-oxabicyclo[3.1.1]heptan-1-yl)acetic acid hydrochloride (26)

A solution of 5-phenyl-3-oxabicyclo[3.1.1]heptane-1-carbaldehyde (5.00 g, 0.025 mol, 1.00 equiv), NH_4Cl (2.6 g, 0.05 mol, 2.00 equiv), KCN (3.20 g, 0.05 mol, 2.00 equiv) and NH_4OH (40 mL) in a mixture of 50 mL of water and 250 mL of MeOH was stirred at 70 °C (in an oil bath with a thermocouple) for 12 h. The mixture was alkalized with a saturated aq. $NaHCO_3$ solution to pH = 9 and extracted with CH_2Cl_2 (3 × 100 mL). The combined organic layers were washed with brine (1 × 100 mL), dried over Na_2SO_4, filtered, and concentrated under reduced pressure. The residue was diluted with 50 mL of water and 50 mL of 10M HCl and heated under reflux overnight. The solution was cooled to 5 °C and filtered. The obtained precipitate was washed with cold water (~ 50 mL) and dried. Yield: 3.00 g, 0.01 mol, 40%, beige solid, m.p. = 202-203 °C. ^1H NMR (500 MHz, DMSO-d_6): δ 13.89 (br s, 1H), 8.58 (br s, 3H), 7.33 (t, J = 7.5 Hz, 2H), 7.23 (t, J = 7.2 Hz, 1H), 7.12 (d, J = 7.4 Hz, 2H), 3.99 (d, J = 9.5 Hz, 1H), 3.89 (s, 1H), 3.79 – 3.62 (m, 3H), 2.22 (dd, J = 34.1, 6.5 Hz, 2H), 2.10 (d, J = 6.8 Hz, 2H) ppm. $^{13}C\{^1H\}$ NMR (126 MHz, DMSO-d_6): δ 168.9, 148.0, 128.4, 126.5, 125.4, 74.7, 69.4, 55.6, 42.8, 38.4, 36.5 ppm. LCMS $(M + H)^+$: 248. HRMS (ESI-TOF) *m/z*: $[M + H]^+$ calcd for $C_{14}H_{18}NO_3^+$: 248.1281; found: 248.1284.

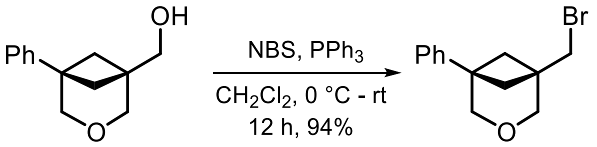

1-(Bromomethyl)-5-phenyl-3-oxabicyclo[3.1.1]heptane (27)

To a stirred solution of (5-phenyl-3-oxabicyclo[3.1.1]heptan-1-yl)methanol (20.00 g, 0.10 mol, 1.00 equiv) and Ph_3P (29.00 g, 0.11 mol, 1.10 equiv) in 400 mL of CH_2Cl_2 was added NBS (20.00 g, 0.11 mol, 1.10 equiv) in portions at 0 °C (ice-water bath). The resulting solution was slowly warmed to room temperature and stirred overnight. The mixture was filtered. The filtrate was washed with water (2 × 200 mL), dried over Na_2SO_4, filtered, and evaporated to dryness under reduced pressure. The residue was dilluted with 1 L of MeO*t*Bu and filtered. The filtrate was concentrated under reduced pressure. The final product was purified by column chromatography (SiO_2, hexane/THF, 1:1). Yield: 25.00 g, 0.094 mol, 94%, beige solid, m.p. = 57-58 °C. ^1H NMR (500 MHz, $CDCl_3$): δ 7.32 (t, J = 7.5 Hz, 2H), 7.23 (t, J = 7.3 Hz, 1H), 7.10 (d, J = 7.7 Hz, 2H),

3.94 (s, 2H), 3.85 (s, 2H), 3.35 (s, 2H), 2.19 (dd, J = 6.3, 2.4 Hz, 2H), 2.02 (d, J = 7.9 Hz, 2H) ppm. $^{13}C\{^{1}H\}$ NMR (126 MHz, $CDCl_3$): δ 144.3, 128.6, 126.7, 125.6, 76.1, 72.9, 42.9, 40.2, 39.2, 38.3 ppm. GCMS $(M)^+$: 268. HRMS (ESI-TOF) *m/z*: $[M + H]^+$ calcd for $C_{13}H_{16}{}^{79}BrO^+$: 267.0379; found: 267.0376; calcd for $C_{13}H_{16}{}^{80}BrO^+$: 269.0359; found: 269.0357.

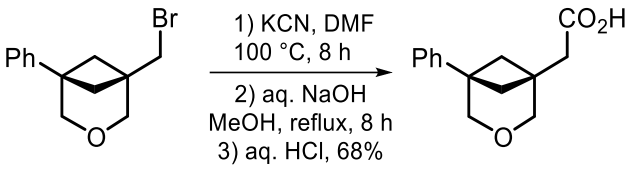

2-(5-Phenyl-3-oxabicyclo[3.1.1]heptan-1-yl)acetic acid (28)

A solution of 1-(bromomethyl)-5-phenyl-3-oxabicyclo[3.1.1]heptane (5.00 g, 0.019 mol, 1.00 equiv) and NaCN (3.00 g, 0.06 mol, 3.16 equiv) in 100 mL of DMSO was stirred at 100 °C (in an oil bath with a thermocouple) for 8 h. The solution was cooled to room temperature, diluted with water (300 mL), and extracted with (2 × 100 mL) EtOAc. The combined organic layers were washed with water (2 × 100 mL), brine (1 × 100 mL) dried over Na_2SO_4, filtered, and concentrated under reduced pressure. The residue was dissolved in 50 mL of MeOH and a solution of NaOH (4.00 g, 0.10 mol, 5.26 equiv) in 20 mL of water was added. The resulting mixture was heated under reflux for 8 h, cooled to room temperature, and concentrated under reduced pressure. The residue was diluted with 100 mL of water and washed with MeO*t*Bu (2 × 50 mL). The aqueous layer was acidified with con. aq. HCl to pH = 4 and filtered. The precipitate was washed with water (~ 50 mL) and dried. Yield: 3.10 g, 0.013 mol, 68%, yellow solid, m.p. = 96-97 °C. ^{1}H NMR (500 MHz, DMSO-d_6): δ 12.12 (s, 1H), 7.31 (t, J = 7.5 Hz, 2H), 7.21 (t, J = 7.4 Hz, 1H), 7.11 (d, J = 7.0 Hz, 2H), 3.77 (s, 2H), 3.71 (s, 2H), 2.30 (s, 2H), 2.10 (dd, J = 6.3, 2.5 Hz, 2H), 1.98 (dd, J = 6.2, 1.9 Hz, 2H) ppm. $^{13}C\{^{1}H\}$ NMR (151 MHz, DMSO-d_6): δ 172.2, 144.8, 128.3, 126.2, 125.3, 75.0, 72.7, 43.6, 40.4, 36.7 ppm. LCMS $(M + H)^+$: 231. HRMS (ESI-TOF) *m/z*: $[M - H]^-$ calcd for $C_{14}H_{15}O_3^-$: 231.1027; found: 231.1027.

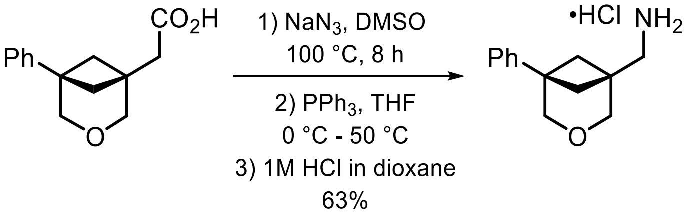

(5-Phenyl-3-oxabicyclo[3.1.1]heptan-1-yl)methanamine hydrochloride (29)

A solution of 1-(bromomethyl)-5-phenyl-3-oxabicyclo[3.1.1]heptane (5.00 g, 0.019 mol, 1.00 equiv) and NaN_3 (4.00 g, 0.06 mol, 3.16 equiv) in 100 mL of DMSO was stirred at 100 °C (in an oil bath with a thermocouple) for 8 h. The solution was diluted with water (200 mL) and extracted with

EtOAc (2 × 100 mL). The combined organic layers were washed with water (2× 100 mL), brine (1 × 200 mL), dried over Na_2SO_4, filtered, and evaporated to dryness under reduced pressure. The residue was dissolved in 100 mL of THF and Ph_3P (7.40 g, 0.028 mol, 1.47 equiv) was added in portions at 0 °C. The solution was warmed to room temperature, stirred for 4 h, and 100 mL of water was added. The mixture was stirred overnight at 50 °C (in an oil bath with a thermocouple) and concentrated under reduced pressure. The residue was diluted with 200 mL of MeO*t*Bu, acidified with 1M HCl in dioxane to pH = 5, and filtered. The obtained precipitate was washed with MeO*t*Bu (20 mL), acetone (20 mL), and dried. Yield: 2.90 g, 0.012 mol, 63%, white solid, m.p. = 212-213 °C. ^{1}H NMR (500 MHz, DMSO-d_6): δ 8.08 (br s, 3H), 7.33 (t, *J* = 7.5 Hz, 2H), 7.23 (t, *J* = 7.3 Hz, 1H), 7.13 (d, *J* = 7.1 Hz, 2H), 3.80 (s, 2H), 3.73 (s, 2H), 2.80 (s, 2H), 2.11 (d, *J* = 6.4 Hz, 2H), 2.02 (dd, *J* = 6.4, 2.3 Hz, 2H) ppm. ^{13}C{^{1}H} NMR (126 MHz, DMSO-d_6): δ 144.3, 128.2, 126.2, 125.2, 74.9, 70.8, 43.0, 42.4, 37.6, 37.2 ppm. LCMS (M + H)$^+$: 204. HRMS (ESI-TOF) *m/z*: [M + H]$^+$ calcd for $C_{13}H_{18}NO^+$: 204.1383; found: 204.1379.

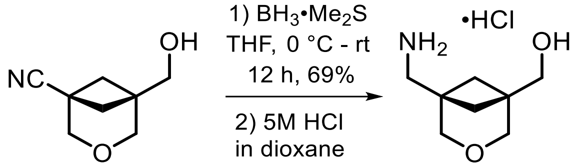

(5-(Aminomethyl)-3-oxabicyclo[3.1.1]heptan-1-yl)methanol hydrochloride (30)

To a stirred solution of 5-(hydroxymethyl)-3-oxabicyclo[3.1.1]heptane-1-carbonitrile (100.00 g, 0.65 mol, 1.00 equiv) in 3 L of THF at 0 °C (ice-water bath) was added dropwise $BH_3{\cdot}Me_2S$ (300 mL, 3.25 mol, 5.00 equiv). The mixture was slowly warmed to room temperature and stirred overnight. The solution was cooled to 0 °C (ice-water bath) and MeOH (400 mL, 10.00 mol, 16.38 equiv) was added dropowise. The mixture was concentrated under reduced pressure and diluted with 1 L of water and 650 mL of 10M HCl. The resulting solution was heated under reflux until all precipitate was dissolved. The mixture was cooled to room temperature and concentrated under reduced pressure. The residue was diluted with 1 L of water and alkalized with NaOH to pH = 9. The solution was concentrated under reduced pressure. The residue was dissolved in 2 L of THF, heated under reflux, and filtered. This procedure was repeated 3 times. The combined organic filtrates were concentrated under reduced pressure. The final product was purified by distillation (0.4 mbar, b.p. = 85-86 °C). Yield: 71.00 g, 0.45 mol, 69%, yellow oil. The product was dissolved in MeOH (200 mL) and 5M HCl in dioxane was added (300 mL). The solution was concentrated under reduced pressure. Yield: 87.08 g, 0.45 mol, 100%, beige solid, m.p. = 125-126 °C. ^{1}H NMR (500 MHz, DMSO-d_6): δ 7.99 (br s, 3H), 4.61 (br s, 1H), 3.68 (s, 2H), 3.64 (s, 2H), 3.20 (s, 2H), 2.71 (s, 2H), 1.74 (d, *J* = 6.3 Hz, 2H), 1.46 (dd, *J* = 6.3, 2.5 Hz, 2H) ppm. ^{13}C{^{1}H} NMR (126

MHz, DMSO-d_6): δ 71.7, 71.5, 64.4, 42.9, 41.3, 37.9, 34.7 ppm. LCMS (M + H)$^+$: 158. HRMS (ESI-TOF) *m/z*: [M + H]$^+$ calcd for $C_8H_{16}NO_2^+$: 158.1176; found: 158.1169.

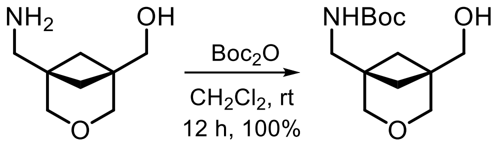

***tert*-Butyl ((5-(hydroxymethyl)-3-oxabicyclo[3.1.1]heptan-1-yl)methyl)carbamate (31)**

To a solution of (5-(aminomethyl)-3-oxabicyclo[3.1.1]heptan-1-yl)methanol (50.00 g, 0.32 mol, 1.00 equiv) in 1 L of CH_2Cl_2 was added a solution of Boc_2O (70.00 g, 0.32 mol, 1.00 equiv) in 100 mL of CH_2Cl_2 dropwise. The resulting solution was stirred overnight and evaporated to dryness under reduced pressure. Yield: 82.24 g, 0.32 mol, 100%, colorless oil. ^{1}H NMR (500 MHz, $CDCl_3$): δ 4.50 (br s, 1H), 3.81 (s, 2H), 3.76 (s, 2H), 3.45 (s, 2H), 3.04 (d, *J* = 6.1 Hz, 2H), 1.65 (d, *J* = 6.2 Hz, 1H), 1.59 (dd, *J* = 6.2, 1.8 Hz, 1H), 1.55 (s, 2H), 1.44 (s, 9H), 1.25 (br s, 1H) ppm. ^{13}C{^{1}H} NMR (126 MHz, $CDCl_3$): δ 156.3, 79.6, 73.2, 72.6, 66.4, 45.4, 41.7, 40.7, 34.6, 28.5 ppm. HRMS (ESI-TOF) *m/z*: [M + Na]$^+$ calcd for $C_{13}H_{23}NNaO_4^+$: 280.1519; found: 280.1514.

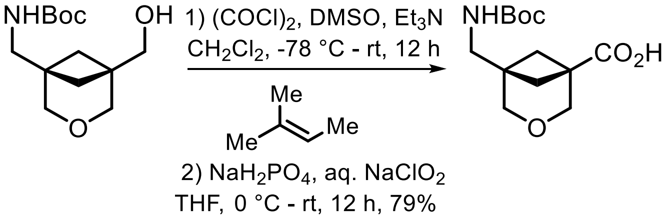

5-(((*tert*-Butoxycarbonyl)amino)methyl)-3-oxabicyclo[3.1.1]heptane-1-carboxylic acid (32)

To a stirred solution of oxalyl chloride (21 mL, 0.25 mol, 1.28 equiv) in 2 L of CH_2Cl_2 was added a solution of DMSO (21 mL, 0.29 mol, 1.49 equiv) in 100 mL of CH_2Cl_2 dropwise at -78 °C. The resulting mixture was stirred at the same temperature for 1 h, and the solution of *tert*-butyl ((5-(hydroxymethyl)-3-oxabicyclo[3.1.1]heptan-1-yl)methyl)carbamate (50.00 g, 0.195 mol, 1.00 equiv) in 150 mL of CH_2Cl_2 was added dropwise. The mixture was stirred for 4 h at -60 °C and Et_3N (139 mL, 1.00 mol, 5.12 equiv) was added. The resulting solution was slowly warmed and stirred at room temperature overnight. The organic layer was washed with water (2 × 300 mL), dried over Na_2SO_4, filtered, and evaporated to dryness under reduced pressure. To a stirred solution of *tert*-butyl ((5-formyl-3-oxabicyclo[3.1.1]heptan-1-yl)methyl)carbamate (45.00 g, 0.176 mol, 1.00 equiv), 2-methylbut-2-ene (79 mL, 0.75 mol, 4.26 equiv) and NaH_2PO_4 (21.00 g, 0.195 mol, 1.11 equiv) in a mixture of 1 L of water and 1 L of THF was added a solution of $NaClO_2$ (19.00 g, 0.195 mol, 1.11 equiv) in 100 mL of water dropwise at 0 °C (ice-water bath). The reaction mixture was slowly warmed to room temperature and stirred overnight. The solution was concentrated under reduced pressure, diluted with water (300 mL), acidified with $NaHSO_4$ to pH = 5, and extracted

with EtOAc (3 × 200 mL). The combined organic layers were washed with brine (1 × 200 mL), dried over Na_2SO_4, filtered, and evaporated to dryness under reduced pressure. The final product was purified by crystallization from hexane. Yield over 2 steps: 42.01 g, 0.155 mol, 79%, white solid, m.p. = 135-136 °C. ^{1}H NMR (500 MHz, DMSO-d_6): δ 12.44 (br s, 1H), 6.95 (t, J = 5.7 Hz, 1H), 3.82 (s, 2H), 3.59 (s, 2H), 3.33 (s, 2H), 2.83 (d, J = 6.1 Hz, 2H), 2.02 (d, J = 6.1 Hz, 2H), 1.58 (d, J = 6.2 Hz, 2H), 1.38 (s, 9H) ppm. Additional (duplicated) signals are observed due to N-Boc rotamers. ^{13}C{^{1}H} NMR (151 MHz, $CDCl_3$): δ 177.6, 177.0, 158.3, 156.3, 81.5, 79.9, 77.2, 72.7, 72.3, 70.4, 70.1, 44.9, 44.7, 43.3, 40.7, 40.3, 36.4, 35.4, 28.5 ppm. LCMS $(M - H)^-$: 270. HRMS (ESI-TOF) *m/z*: $[M + Na]^+$ calcd for $C_{13}H_{21}NNaO_5^+$: 294.1312; found: 294.1306.

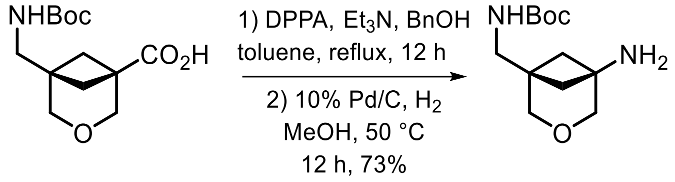

***tert*-Butyl ((5-amino-3-oxabicyclo[3.1.1]heptan-1-yl)methyl)carbamate (33)**

A mixture of 5-(((*tert*-butoxycarbonyl)amino)methyl)-3-oxabicyclo[3.1.1]heptane-1-carboxylic acid (30.00 g, 0.11 mol, 1.00 equiv), DPPA (28 mL, 0.13 mol, 1.18 equiv), Et_3N (18 mL, 0.13 mol, 1.18 equiv) and BnOH (50 mL, 0.50 mol, 4.55 equiv) in 1 L of toluene was heated under reflux (in an oil bath with a thermocouple) for 12 h. The solution was concentrated under reduced pressure, diluted with water (200 mL), alkalized with NaOH to pH = 9, and extracted with EtOAc (3 × 200 mL). The combined organic layers were washed with brine (1 × 200 mL), dried over Na_2SO_4, filtered, and evaporated to dryness under reduced pressure. The final product was purified by column chromatography (SiO_2, hexane/THF, 2:1). A solution of benzyl (5-(((*tert*-butoxycarbonyl)amino)methyl)-3-oxabicyclo[3.1.1]heptan-1-yl)carbamate (34.00 g, 0.09 mol) and 10% Pd/C (4.00 g) in 500 mL of MeOH was stirred at 50 °C (in an oil bath with a thermocouple) for 12 h in an atmosphere of H_2 (50 atm). The mixture was cooled to room temperature, filtered through a pad of celite, and concentrated under reduced pressure. Yield over 2 steps: 19.00 g, 0.08 mol, 73%, white solid, m.p. = 86-87 °C. ^{1}H NMR (500 MHz, $CDCl_3$): δ 4.52 (br s, 1H), 3.66 (s, 2H), 3.65 (s, 2H), 3.06 (d, J = 6.0 Hz, 2H), 1.81 (dd, J = 6.1, 2.2 Hz, 2H), 1.60 (dd, J = 6.1, 1.8 Hz, 1H), 1.43 (s, 9H) ppm. ^{13}C{^{1}H} NMR (126 MHz, $CDCl_3$): δ 156.2, 79.5, 75.5, 71.8, 52.3, 45.1, 41.8, 37.7, 28.5 ppm. LCMS $(M + H)^+$: 243. HRMS (ESI-TOF) *m/z*: $[M + H]^+$ calcd for $C_{12}H_{23}N_2O_3^+$: 243.1703; found: 243.1698.

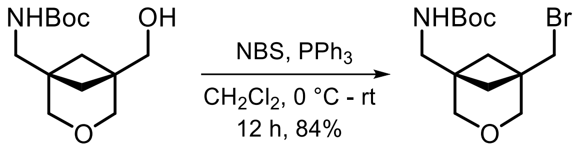

***tert*-Butyl ((5-(bromomethyl)-3-oxabicyclo[3.1.1]heptan-1-yl)methyl)carbamate**

To a stirred solution of *tert*-butyl ((5-(hydroxymethyl)-3-oxabicyclo[3.1.1]heptan-1-yl)methyl)carbamate (80.00 g, 0.31 mol, 1.00 equiv) and Ph_3P (105.00 g, 0.40 mol, 1.29 equiv) in 2 L of CH_2Cl_2 was added NBS (71.20 g, 0.40 mol, 1.29 equiv) in portions at 0 °C (ice-water bath). The resulting solution was slowly warmed to room temperature and stirred overnight. The mixture was filtered. The filtrate was washed with water (2 × 200 mL), dried over Na_2SO_4, filtered, and evaporated to dryness under reduced pressure. The residue was diluted with 1 L of MeO*t*Bu and filtered. The precipitate was heated under reflux in 1 L of MeO*t*Bu and filtered again. The combined filtrates were concentrated under reduced pressure. The final product was purified by column chromatography (SiO_2, hexane/THF, 3:1). Yield: 83.00 g, 0.26 mol, 84%, yellow oil. ^1H NMR (500 MHz, $CDCl_3$): δ 4.54 (br s, 1H), 3.82 (s, 2H), 3.73 (s, 2H), 3.28 (s, 2H), 3.04 (d, J = 5.9 Hz, 2H), 1.69 (d, J = 6.3 Hz, 2H), 1.64 (d, J = 6.6 Hz, 2H), 1.43 (s, 9H) ppm. $^{13}C\{^1H\}$ NMR (151 MHz, $CDCl_3$): δ 156.2, 79.7, 73.3, 72.5, 45.0, 40.5, 39.8, 38.4, 36.9, 28.5 ppm. HRMS (ESI-TOF) *m/z*: [M - *t*Bu + H]$^+$ calcd for $C_9H_{15}BrNO_3^+$: 264.0230; found: 264.0228.

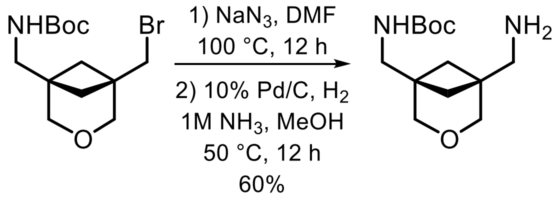

***tert*-Butyl ((5-(aminomethyl)-3-oxabicyclo[3.1.1]heptan-1-yl)methyl)carbamate (34)**

A solution of *tert*-butyl ((5-(bromomethyl)-3-oxabicyclo[3.1.1]heptan-1-yl)methyl)carbamate (51.52 g, 0.161 mol, 1.00 equiv) and NaN_3 (32.50 g, 0.50 mol, 3.08 equiv) in 1 L of DMF was stirred at 100 °C (in an oil bath with a thermocouple) for 12 h. The solution was concentrated under reduced pressure, diluted with water (500 mL), and extracted with EtOAc (2 × 300 mL). The combined organic layers were washed with water (2× 200 mL), brine (2× 200 mL), dried over Na_2SO_4, filtered, and evaporated to dryness under reduced pressure. The final product was purified by column chromatography (SiO_2, hexane/THF, 2:1). Then a solution of *tert*-butyl ((5-(azidomethyl)-3-oxabicyclo[3.1.1]heptan-1-yl)methyl)carbamate (35.00 g, 0.137 mol) and 10% Pd/C (4.00 g) in 500 mL of a 1M NH_3 solution in MeOH was stirred at 50 °C (in an oil bath with a thermocouple) for 12 h in an atmosphere of H_2 (50 atm). The mixture was cooled to room temperature, filtered throught a pad of celite, and concentrated under reduced pressure. Yield over 2 steps: 25.00 g, 0.098 mol, 60%, yellow oil. ^1H NMR (500 MHz, $CDCl_3$): δ 4.53 (br s, 1H), 3.77 (s,

2H), 3.75 (s, 2H), 3.04 (d, J = 6.0 Hz, 2H), 2.58 (s, 2H), 1.63 (br s, 2H), 1.57 (s, 4H), 1.43 (s, 9H) ppm. $^{13}C\{^1H\}$ NMR (151 MHz, $CDCl_3$): δ 156.2, 79.5, 73.5, 73.1, 47.2, 45.4, 41.8, 40.5, 35.0, 28.5 ppm. HRMS (ESI-TOF) *m/z*: $[M + H]^+$ calcd for $C_{13}H_{25}N_2O_3^+$: 257.1860; found: 257.1856.

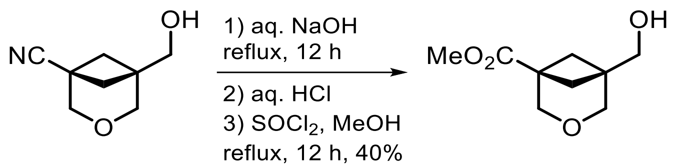

Methyl 5-(hydroxymethyl)-3-oxabicyclo[3.1.1]heptane-1-carboxylate (35)

A solution of 5-(hydroxymethyl)-3-oxabicyclo[3.1.1]heptane-1-carbonitrile (75.00 g, 0.50 mol, 1.00 equiv) in 400 mL of water and NaOH (100.00 g, 2.50 mol, 5.00 equiv) was heated under reflux (in an oil bath with a thermocouple) for 12 h. Then the mixture was cooled to room temperature and acidified with con. aq. HCl to pH = 5. The solution was concentrated under reduced pressure, diluted with 1 L of MeOH, and $SOCl_2$ (26 mL, 0.50 mol, 5.00 equiv) was added dropwise at 0 °C. The mixture was heated under reflux for 12 h, and then cooled to room temperature, filtered, and evaporated to dryness. The residue was diluted with 1 L of EtOAc, filtered through a pad of celite, and concentrated under reduced pressure (bath temperature ≤ 40 °C). The final product was purified by distillation (0.4 mbar, b.p. = 87 °C). Yield: 37.00 g, 0.20 mol, 40%, colorless oil. For further modifications, the crude product was used. ^1H NMR (500 MHz, $CDCl_3$): δ 4.03 (s, 2H), 3.82 (s, 2H), 3.68 (s, 3H), 3.47 (s, 2H), 2.22 (dd, J = 6.4, 2.3 Hz, 2H), 1.82 (dd, J = 6.3, 2.6 Hz, 2H), 1.57 (br s, 1H) ppm. $^{13}C\{^1H\}$ NMR (126 MHz, $CDCl_3$): δ 173.2, 71.9, 70.6, 65.9, 52.0, 43.6, 41.6, 35.8 ppm. HRMS (ESI-TOF) *m/z*: $[M + H]^+$ calcd for $C_9H_{15}O_4^+$: 187.0965; found: 187.0972.

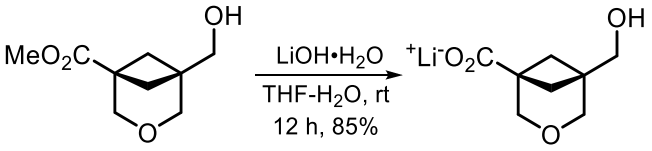

Lithium 5-(hydroxymethyl)-3-oxabicyclo[3.1.1]heptane-1-carboxylate (36)

A solution of methyl 5-(hydroxymethyl)-3-oxabicyclo[3.1.1]heptane-1-carboxylate (37.00 g, 0.20 mol, 1.00 equiv) and $LiOH{\cdot}H_2O$ (7.20 g, 0.18 mol, 0.90 equiv) in a mixture of 250 mL of water and 250 mL of THF was stirred at room temperature for 12 h. The solution was concentrated under reduced pressure, diluted with 250 mL of MeO*t*Bu, and filtered. The precipitate was washed with 100 mL of MeO*t*Bu and dried. Yield: 30.00 g, 0.17 mol, 85%, white solid, m.p. = 209-210 °C. ^1H NMR (500 MHz, DMSO-d_6): δ 4.49 (br s, 1H), 3.76 (s, 2H), 3.57 (s, 2H), 3.17 (s, 2H), 1.86 (d, J = 6.2 Hz, 2H), 1.37 (dd, J = 5.9, 1.9 Hz, 2H) ppm. $^{13}C\{^1H\}$ NMR (151 MHz, DMSO-d_6): δ 177.1, 72.6, 71.7, 65.0, 44.4, 40.5, 36.0 ppm. LCMS $(M + H)^+$: 171. HRMS (ESI-TOF) *m/z*: $[M - H]^-$ calcd for $C_8H_{11}O_4^-$: 171.0663; found: 171.0665.

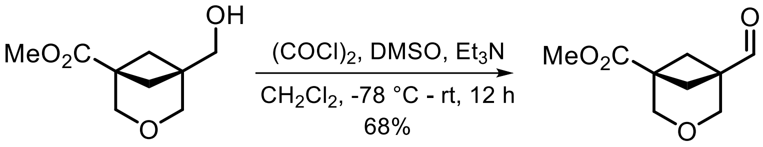

Methyl 5-formyl-3-oxabicyclo[3.1.1]heptane-1-carboxylate (37)

To a stirred solution of oxalyl chloride (21 mL, 0.25 mol, 1.28 equiv) in 2 L of CH_2Cl_2 was added a solution of DMSO (21 mL, 0.29 mol, 1.49 equiv) in 100 mL of CH_2Cl_2 dropwise at -78 °C. The resulting mixture was stirred at the same temperature for 1 h, and the solution of methyl 5-(hydroxymethyl)-3-oxabicyclo[3.1.1]heptane-1-carboxylate (37.00 g, 0.20 mol, 1.00 equiv) in 150 mL of CH_2Cl_2 was added dropwise. The mixture was stirred for 4 h at -60 °C and Et_3N (139 mL, 1.00 mol, 5.12 equiv) was added. The resulting solution was slowly warmed and stirred at room temperature overnight. The solution was concentrated under reduced pressure, diluted with 1 L of EtOAc, filtered throught a pad of celite, and concentrated under reduced pressure. The final product was purified by distillation (0.4 mbar, b.p. = 62 °C). Yield: 25.00 g, 0.136 mol, 68%, colorless oil. ^{1}H NMR (500 MHz, $CDCl_3$): δ 9.52 (s, 1H), 4.08 (s, 2H), 4.05 (s, 2H), 3.72 (s, 3H), 2.64 (dd, *J* = 6.5, 2.1 Hz, 1H), 2.00 (dd, *J* = 6.6, 2.4 Hz, 2H) ppm. ^{13}C{^{1}H} NMR (126 MHz, $CDCl_3$): δ 199.7, 172.2, 70.7, 68.3, 52.2, 48.9, 44.0, 35.5 ppm. HRMS (ESI-TOF) *m/z*: not detected under the experimental conditions.

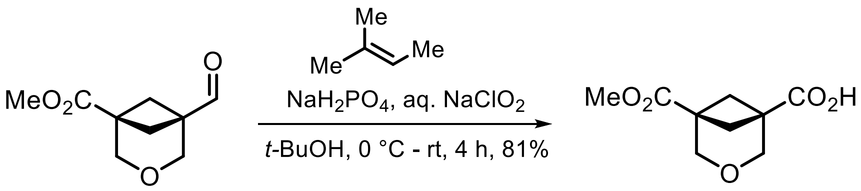

5-(Methoxycarbonyl)-3-oxabicyclo[3.1.1]heptane-1-carboxylic acid (38)

To a stirred solution of methyl 5-formyl-3-oxabicyclo[3.1.1]heptane-1-carboxylate (25.00 g, 0.136 mol, 1.00 equiv), 2-methylbut-2-ene (79 mL, 0.75 mol, 4.26 equiv) and NaH_2PO_4 (21.00 g, 0.195 mol, 1.11 equiv) in a mixture of 0.7 L of water and 0.7 L of *t*-BuOH was added a solution of $NaClO_2$ (19.00 g, 0.195 mol, 1.11 equiv) in 100 mL of water dropwise at 0 °C (ice-water bath). The reaction mixture was slowly warmed to room temperature and stirred for 4 h. The solution was diluted with water (2 L), acidified with $NaHSO_4$ to pH = 4, and extracted with EtOAc (2 × 1 L). The combined organic layers were washed with water (2 × 1 L), brine (1 × 500 mL), dried over Na_2SO_4, filtered, and evaporated to dryness under reduced pressure. The residue was diluted with hexane (500 mL) and left in the fridge overnight. The precipitate was filtered, washed with hexane (100 mL), and dried. Additionally, the product can be recrystallized from hexane. Yield: 22.00 g, 0.11 mol, 81%, white solid, m.p. = 83-84 °C. ^{1}H NMR (500 MHz, $CDCl_3$): δ 4.04 (s, 2H), 4.03 (s, 2H), 3.70 (s, 3H), 2.72 (dd, *J* = 6.6, 2.1 Hz, 2H), 2.08 (dd, *J* = 6.5, 2.5 Hz, 2H) ppm. ^{13}C{^{1}H} NMR

(126 MHz, $CDCl_3$): δ 177.5, 172.3, 69.9, 69.5, 52.2, 43.3, 43.0, 37.5 ppm. LCMS $(M + H)^+$: 201. HRMS (ESI-TOF) *m/z*: $[M - H]^-$ calcd for $C_9H_{11}O_5^-$: 199.0612; found: 199.0612.

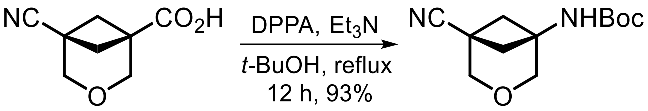

***tert*-Butyl (5-cyano-3-oxabicyclo[3.1.1]heptan-1-yl)carbamate (39)**

A mixture of 5-cyano-3-oxabicyclo[3.1.1]heptane-1-carboxylic acid (50.00 g, 0.30 mol, 1.00 equiv), DPPA (86 mL, 0.40 mol, 1.33 equiv), Et_3N (56 mL, 0.40 mol, 1.33 equiv) in *t*-BuOH (2 L) was heated under reflux (in an oil bath with a thermocouple) for 12 h. The solution was concentrated under reduced pressure, diluted with water (300 mL), alkalized with NaOH to pH = 8, and extracted with EtOAc (3 × 300 mL). The combined organic layers were washed with water (2 × 200 mL), brine (1 × 200 mL), dried over Na_2SO_4, filtered, and evaporated to dryness under reduced pressure. Yield: 67.00 g, 0.28 mol, 93%. ^{1}H NMR (500 MHz, $CDCl_3$): δ 4.72 (br s, 1H), 3.92 (s, 2H), 3.85 (s, 2H), 2.69 (s, 2H), 2.39 (d, J = 6.6 Hz, 2H), 1.42 (s, 9H) ppm. ^{13}C{^{1}H} NMR (101 MHz, $CDCl_3$): δ 154.2, 119.2, 72.3, 69.2, 52.2, 41.2, 29.6, 28.4 ppm. LCMS $(M + H)^+$: 239.

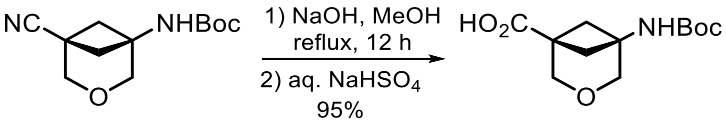

5-((*tert*-Butoxycarbonyl)amino)-3-oxabicyclo[3.1.1]heptane-1-carboxylic acid (40)

A solution of *tert*-butyl (5-cyano-3-oxabicyclo[3.1.1]heptan-1-yl)carbamate (30.00 g, 0.126 mol, 1.00 equiv) and NaOH (24.00 g, 0.60 mol, 4.76 equiv) in a mixture of 500 mL of water and 500 mL of MeOH was heated under reflux (in an oil bath with a thermocouple) for 12 h. The mixture was cooled to room temperature and concentrated under reduced pressure. The residue was diluted with water (1 L) and acidified with $NaHSO_4$ to pH = 4. The precipitate was filtered, washed with water (100 mL), and dried. Yield: 31.00 g, 0.12 mol, 95%, white solid, m.p. = 148-149 °C. ^{1}H NMR (500 MHz, $CDCl_3$): δ 4.69 (br s, 1H), 3.95 (s, 2H), 3.86 (s, 2H), 2.52 (s, 2H), 2.22 (d, J = 4.9 Hz, 2H), 1.44 (s, 9H) ppm. ^{13}C{^{1}H} NMR (151 MHz, DMSO-d_6): δ 173.2, 154.2, 77.9, 71.4, 68.7, 50.4, 41.7, 28.2. LCMS $(M - H)^-$: 256. HRMS (ESI-TOF) *m/z*: $[M + NH_4]^+$ calcd for $C_{12}H_{23}N_2O_5^+$: 275.1601; found: 275.1599.

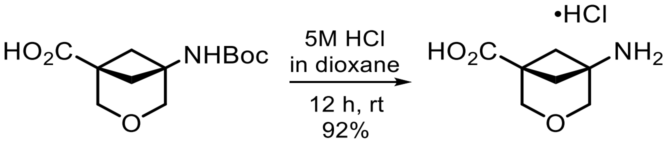

5-Amino-3-oxabicyclo[3.1.1]heptane-1-carboxylic acid hydrochloride (41)

A solution of 5-((*tert*-butoxycarbonyl)amino)-3-oxabicyclo[3.1.1]heptane-1-carboxylic acid (31.00 g, 0.12 mol) in 500 mL of MeO*t*Bu and 250 mL of 5M HCl in dioxane was stirred for 12 h at room temperature and filtered. The precipitate was washed with 1 L of MeO*t*Bu and dried. Yield: 21.00 g, 0.11 mol, 92%, white solid, m.p. = 189-190 °C. ^{1}H NMR (500 MHz, DMSO-d_6): δ 9.50 (br s, 3H), 3.83 (s, 2H), 3.79 (s, 2H), 2.47 (d, *J* = 8.0 Hz, 2H), 2.02 (d, *J* = 6.1 Hz, 2H) ppm. ^{13}C{^{1}H} NMR (151 MHz, DMSO-d_6): δ 172.2, 69.1, 68.6, 50.2, 41.9, 37.8 ppm. LCMS $(M + H)^+$: 158. HRMS (ESI-TOF) *m/z*: not detected under the experimental conditions.

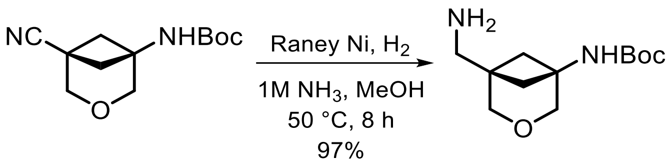

tert-Butyl (5-(aminomethyl)-3-oxabicyclo[3.1.1]heptan-1-yl)carbamate (42)

A mixture of *tert*-butyl (5-cyano-3-oxabicyclo[3.1.1]heptan-1-yl)carbamate (30.00 g, 0.126 mol) and Raney Ni (3.00 g) in a 1M NH_3 solution in MeOH was stirred at 50 °C (in an oil bath with a thermocouple) for 8 h in an atmosphere of H_2 (50 atm). The mixture was cooled to room temperature, filtered throught a pad of celite, and concentrated under reduced pressure. Yield: 29.00 g, 0.122 mol, 97%, beige solid, m.p. = 66-67 °C. ^{1}H NMR (500 MHz, $CDCl_3$): δ 4.64 (br s, 1H), 3.85 (s, 2H), 3.69 (s, 2H), 2.63 (s, 2H), 1.95 (s, 4H), 1.82 (br s, 2H), 1.43 (s, 9H) ppm. ^{13}C{^{1}H} NMR (126 MHz, $CDCl_3$): δ 154.6, 79.8, 73.4, 72.3, 51.6, 46.5, 40.7, 38.6, 28.5 ppm. HRMS (ESI-TOF) *m/z*: $[M + H]^+$ calcd for $C_{12}H_{23}N_2O_3^+$: 243.1703; found: 243.1700.

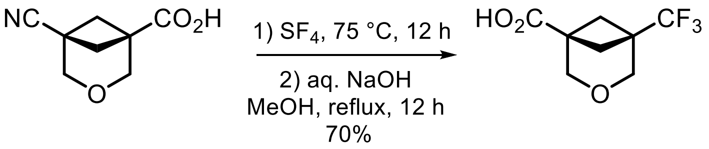

5-(Trifluoromethyl)-3-oxabicyclo[3.1.1]heptane-1-carboxylic acid (43)

A mixture of 5-cyano-3-oxabicyclo[3.1.1]heptane-1-carboxylic acid (60.00 g, 0.36 mol, 1.00 equiv) was stirred in an autoclave with SF_4 (60.00 g, 0.56 mol, 1.56 equiv) at 75 °C for 12 h. The autoclave was allowed to cool down to room temperature, and the gaseous products were vented off into a trap with aqueous solution of NaOH (1M). The residue was poured onto the aq suspension of $NaHCO_3$ (500 mL) and extracted with MeO*t*Bu (3 × 200 mL). The combined organic layers were washed with brine (1 × 200 mL), dried over Na_2SO_4, filtered, and concentrated under reduced pressure. The residue was diluted with 500 mL of water and 500 mL of MeOH, and then NaOH (40.00 g, 1.00 mol, 2.78 equiv) was added. The reaction mixture was heated under reflux for 12 h. The solution was cooled to room temperature, concentrated under reduced pressure, diluted with water (500 mL), and washed with MeO*t*Bu (2 × 500 mL). The aqueous layer was acidified with

$NaHSO_4$ to pH = 4 and filtered. The obtained precipitate was washed with water (100 mL) and dried. Yield: 52.00 g, 0.25 mol, 70%, beige solid, m.p. = 73-74 °C. ^1H NMR (500 MHz, $CDCl_3$): δ 4.05 (s, 2H), 3.97 (s, 2H), 2.61 (d, J = 8.6 Hz, 2H), 2.02 (dd, J = 6.6, 2.5 Hz, 2H) ppm. $^{13}C\{^1H\}$ NMR (151 MHz, $CDCl_3$): δ 177.5, 124.9 (q, J = 277 Hz), 69.6, 67.5, 42.5, 42.3 (q, J = 32 Hz), 34.1 ppm. $^{19}F\{^1H\}$ NMR (376 MHz, $CDCl_3$): δ -76.9 ppm. HRMS (ESI-TOF) *m/z*: $[M - H]^-$ calcd for $C_8H_8F_3O_3^-$: 209.0431; found: 209.0432.

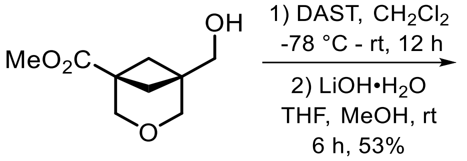

5-(Fluoromethyl)-3-oxabicyclo[3.1.1]heptane-1-carboxylic acid (44)

To a stirred solution of methyl 5-(hydroxymethyl)-3-oxabicyclo[3.1.1]heptane-1-carboxylate (35.00 g, 0.188 mol, 1.00 equiv) in 1 L of CH_2Cl_2 at -78 °C was added dropwise DAST (26 mL, 0.20 mol, 1.06 equiv). The solution was slowly warmed to room temperature and left overnight. The mixture was washed with a solution K_2CO_3 (60.00 g, 0.43 mol, 2.29 equiv) in 1 L of water, water (1 × 500 mL), brine (1 × 500 mL), dried over Na_2SO_4, filtered, and concentrated under reduced pressure. Then a solution of methyl 5-(fluoromethyl)-3-oxabicyclo[3.1.1]heptane-1-carboxylate (21.00 g, 0.11 mol, 1.00 equiv) and $LiOH{\cdot}H_2O$ (4.80 g, 0.12 mol, 1.09 equiv) in a mixture of 250 mL of water, 250 mL of THF and 100 mL of MeOH was stirred at room temperature for 6 h. The solution was concentrated under reduced pressure, diluted with water (200 mL), and acidified with $NaHSO_4$ to pH = 4. The formed precipitate was filtered, washed with water (~ 100 mL), and dried. Yield over 2 steps: 17.40 g, 0.10 mol, 53%, white solid, m.p. = 82-83 °C. ^1H NMR (500 MHz, DMSO-d_6): δ 12.56 (s, 1H), 4.28 (d, J = 47.6 Hz, 2H), 3.88 (s, 2H), 3.71 (s, 2H), 2.14 (dd, J = 6.2, 2.1 Hz, 2H), 1.73 (dd, J = 6.2, 2.5 Hz, 2H) ppm. $^{13}C\{^1H\}$ NMR (151 MHz, DMSO-d_6): δ 173.6, 85.2 (d, J = 165 Hz), 69.8, 69.7 (d, J = 6 Hz), 42.9, 34.8 (d, J = 6 Hz) ppm. $^{19}F\{^1H\}$ NMR (376 MHz, DMSO-d_6): δ -227.8 (s) ppm. LCMS $(M + H)^+$: 175. HRMS (ESI-TOF) *m/z*: $[M - H]^-$ calcd for $C_8H_{10}FO_3^-$: 173.0619; found: 173.0621.

3.6. Synthesis of analogs of Sonidegib

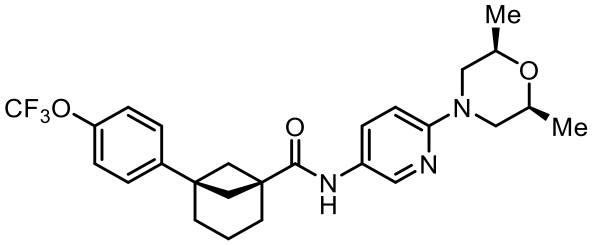

Compound **50** was generously provided by authors of N. Frank, J. Nugent, B. R. Shire, H. D. Pickford, P. Rabe, A. J. Sterling, T. Zarganes-Tzitzikas, T. Grimes, A. L. Thompson, R. C. Smith, C. J. Schofield, P. E. Brennan, F. Duarte, E. A. Anderson. Synthesis of *meta*-substituted arene bioisosteres from [3.1.1]propellane. *Nature* **2022**, *611*, 721-726.

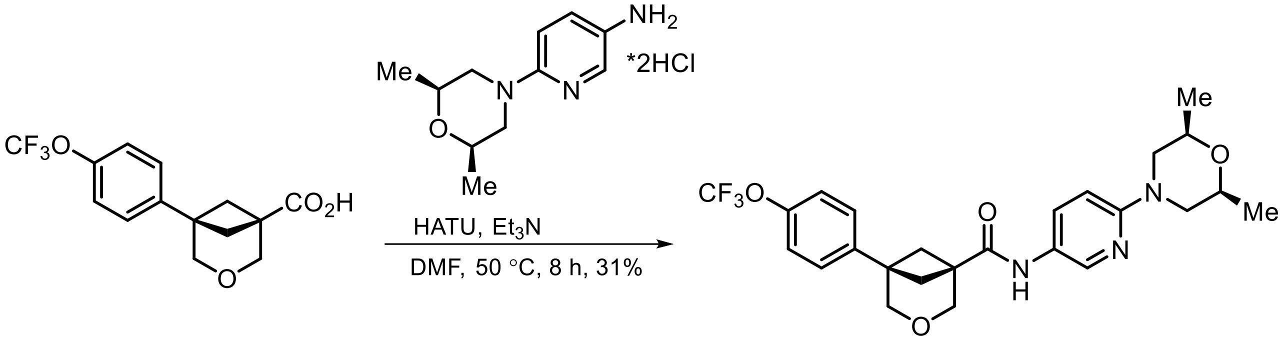

***N*-(6-(2,6-Dimethylmorpholino)pyridin-3-yl)-5-(4-(trifluoromethoxy)phenyl)-3-oxabicyclo[3.1.1]heptane-1-carboxamide (51)**

To a stirred mixture of 6-((2*S*,6*R*)-2,6-dimethylmorpholino)pyridin-3-amine dihydrochloride (0.28 g, 1.00 mmol, 1.00 equiv), 1-[bis(dimethylamino)methylene]-1*H*-1,2,3-triazolo[4,5-*b*]pyridinium 3-oxid hexafluorophosphate (HATU) (0.46 g, 1.20 mmol, 1.20 equiv) and 5-(4-(trifluoromethoxy)phenyl)-3-oxabicyclo[3.1.1]heptane-1-carboxylic acid (0.30 g, 1.00 mmol, 1.00 equiv) in DMF (5 mL) was added Et_3N (0.55 mL, 4.00 mmol, 4.00 equiv). The reaction mixture was stirred at 50 °C (in an oil bath with a thermocouple) for 8 h. The solution was poured into the water (10 mL) and extracted with EtOAc (3 × 5 mL). The combined organic layers were washed with water (2 × 3 mL), brine (1 × 3 mL), dried over Na_2SO_4, filtered, and concentrated under reduced pressure. The final product was purified by flash column chromatography (SiO_2, hexane/THF, 1:2). Yield: 0.15 g, 0.31 mmol, 31%, purple solid. ^{1}H NMR (500 MHz, DMSO-d_6): δ 9.49 (s, 1H), 8.30 (s, 1H), 7.79 (d, *J* = 8.8 Hz, 2H), 7.34 (d, *J* = 8.1 Hz, 2H), 7.30 (d, *J* = 8.5 Hz, 2H), 6.84 (d, *J* = 9.0 Hz, 1H), 4.02 (s, 4H), 3.78 (s, 2H), 3.60 (br s, 2H), 2.49 – 2.41 (m, 3H), 2.36 – 2.29 (m, 3H), 1.14 (d, *J* = 6.0 Hz, 6H) ppm. ^{13}C{^{1}H} NMR (151 MHz, DMSO-d_6): δ 170.7, 155.3, 146.9, 143.5, 139.3, 131.2, 127.5, 126.4, 121.0, 120.1 (q, *J* = 256 Hz), 106.9, 74.4, 70.8, 70.1, 50.7,

44.2, 41.9, 38.1, 18.8 ppm. $^{19}F\{^1H\}$ NMR (376 MHz, DMSO-d_6): δ -57.3 (s) ppm. LCMS $(M - H)^-$: 490. HRMS (ESI-TOF) *m/z*: $[M + H]^+$ calcd for $C_{25}H_{29}F_3N_3O_4^+$: 492.2105; found: 492.2100.

4. Stability test

1). Long-term stability test (16 May 2023 – 14 Oct 2024, ^{1}H NMR in DMSO-d_6).

Method: compound **11b** was stored under normal laboratory conditions for 17 months in our stock. Two ^{1}H NMR (500 MHz, DMSO-d_6) analyses were performed, with the final control recorded on 14 Oct 2024.

Results: no changes in chemical shifts, peak integrals, or splitting patterns were observed throughout the study.

Conclusion: compound **11b** was stable under normal conditions for at least 17 months, with no detectable structural degradation.

2). Thermal exposure:100 °C, 5 min, ^{1}H NMR in DMSO-d_6.

Method: compound **11b** was dissolved in DMSO-d_6, sealed in an NMR tube, and heated at 100 °C for 5 min before ^{1}H NMR (500 MHz) analysis.

Results: no changes in chemical shifts, peak integrals, or splitting patterns were observed.

Conclusion: compound **11b** was stable under these conditions, with no detectable structural modifications.

^{1}H NMR (500 MHz, DMSO-d_6) of compound **11b** (May 16, 2023)

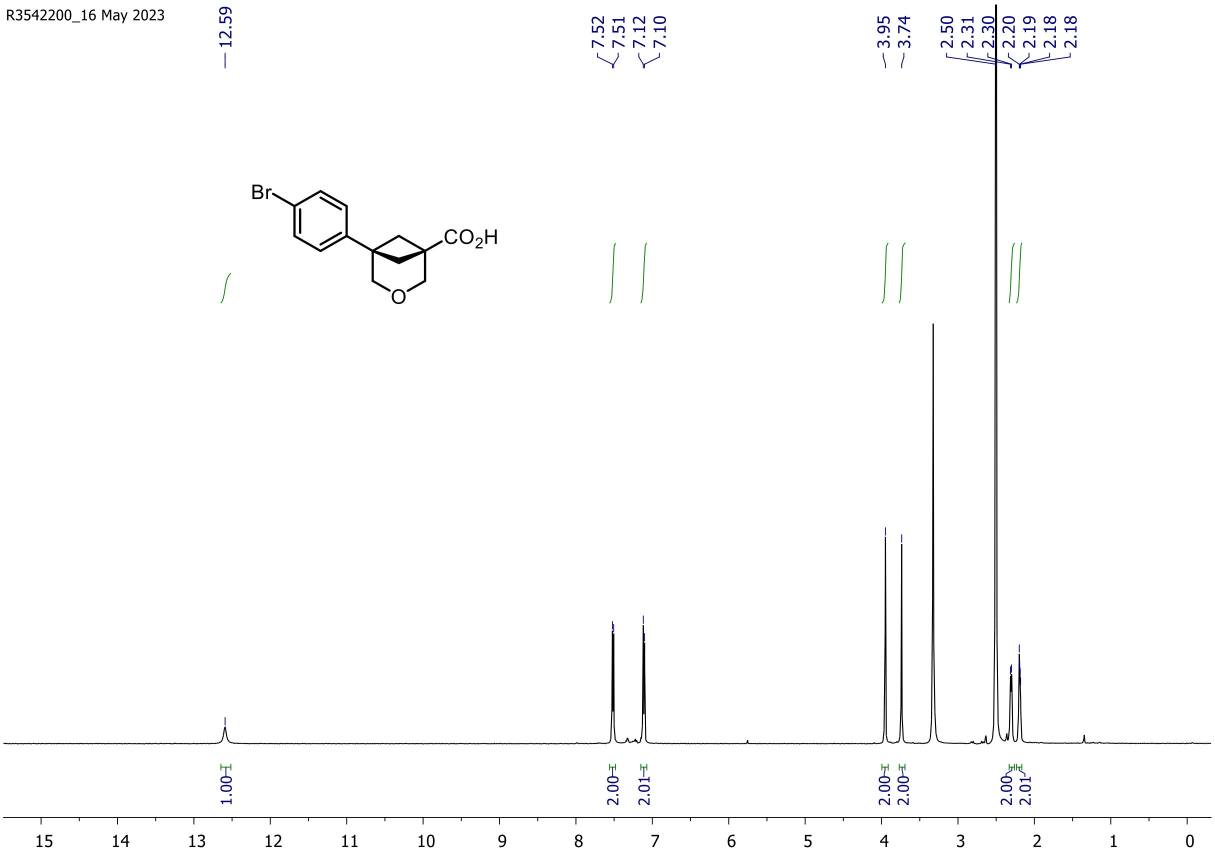

^{1}H NMR (500 MHz, DMSO-d_6) of compound **11b** (October 14, 2024)

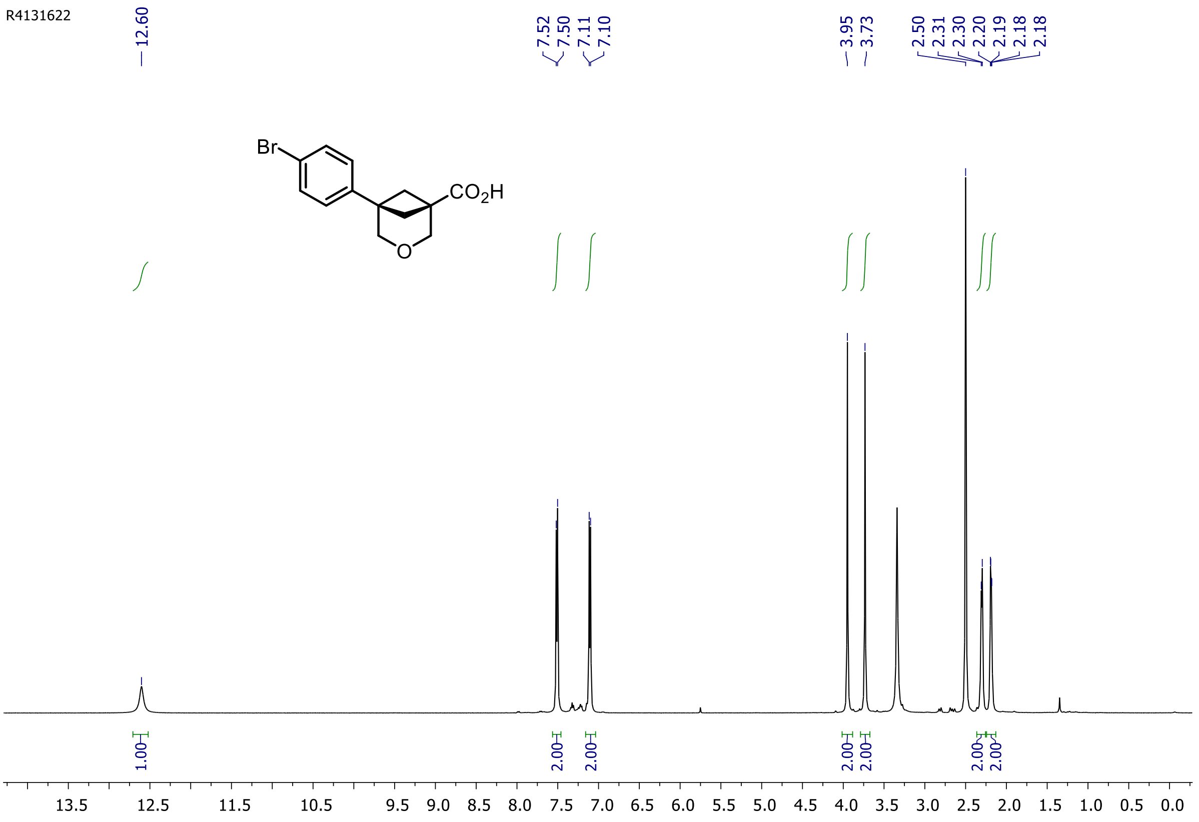

^{1}H NMR (500 MHz, DMSO-d_6) of compound **11b** after heating at 100 °C for 5 min (March 24, 2025)

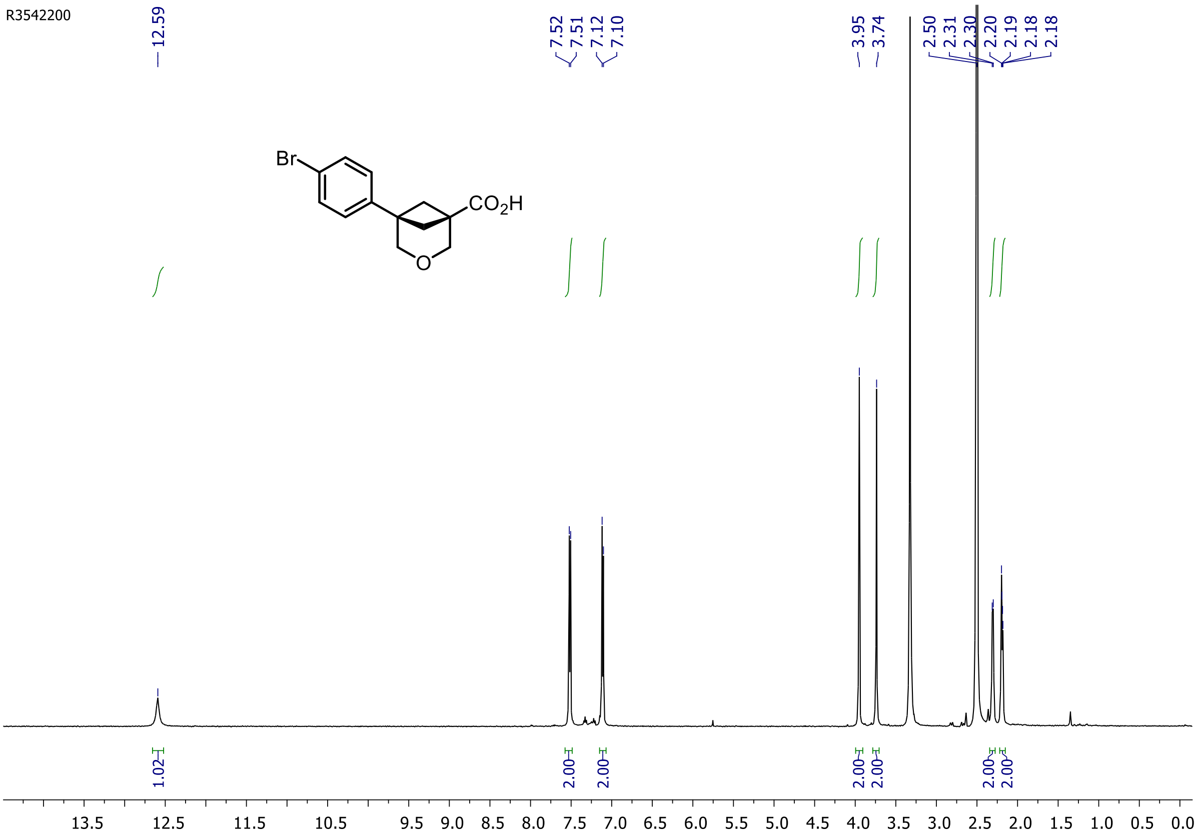

5. Copies of ^{1}H, $^{13}C\{^{1}H\}$ and $^{19}F\{^{1}H\}$ NMR spectra

Compound 4

^{1}H NMR (500 MHz, $CDCl_3$)

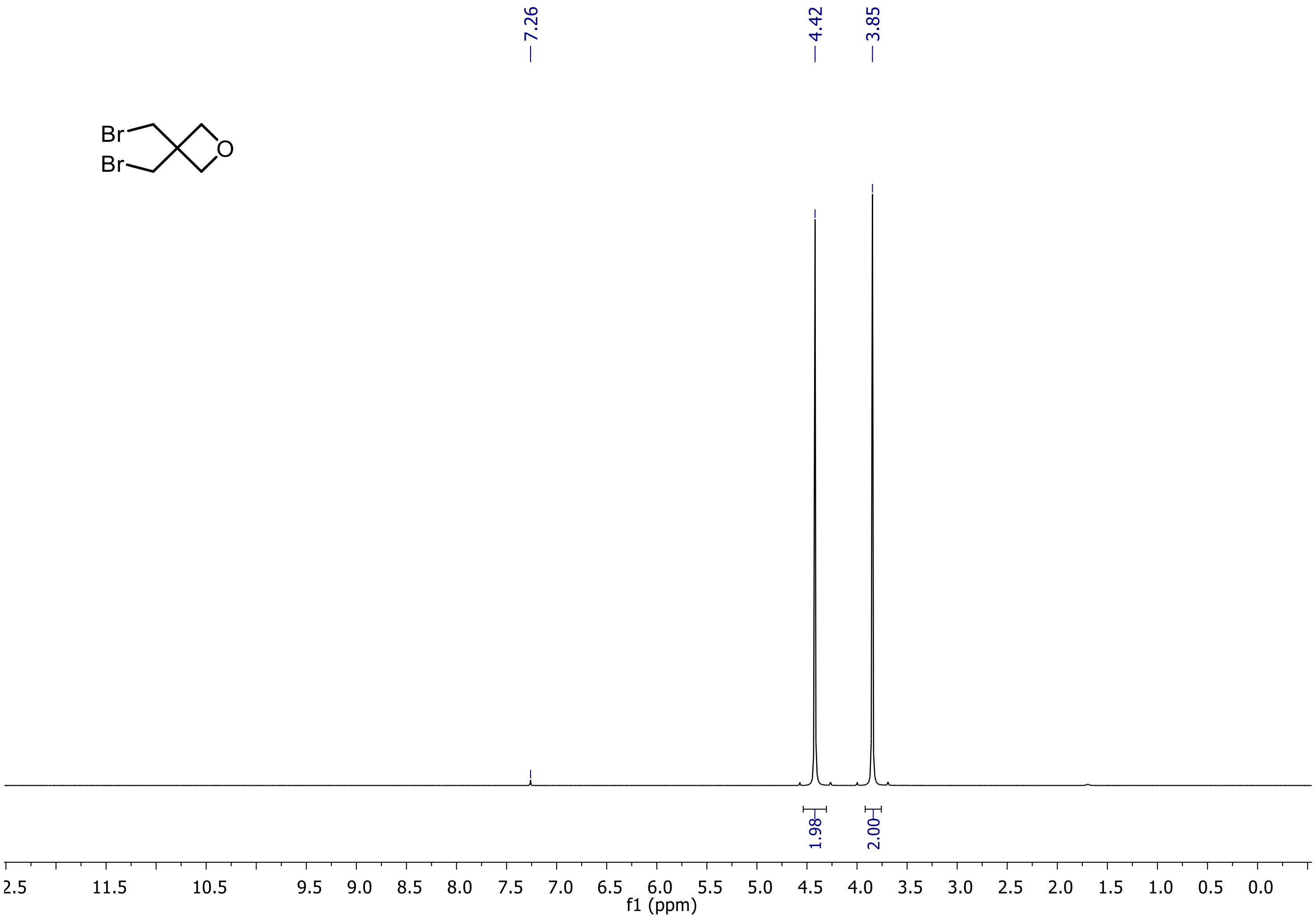

$^{13}C\{^1H\}$ NMR (126 MHz, $CDCl_3$)

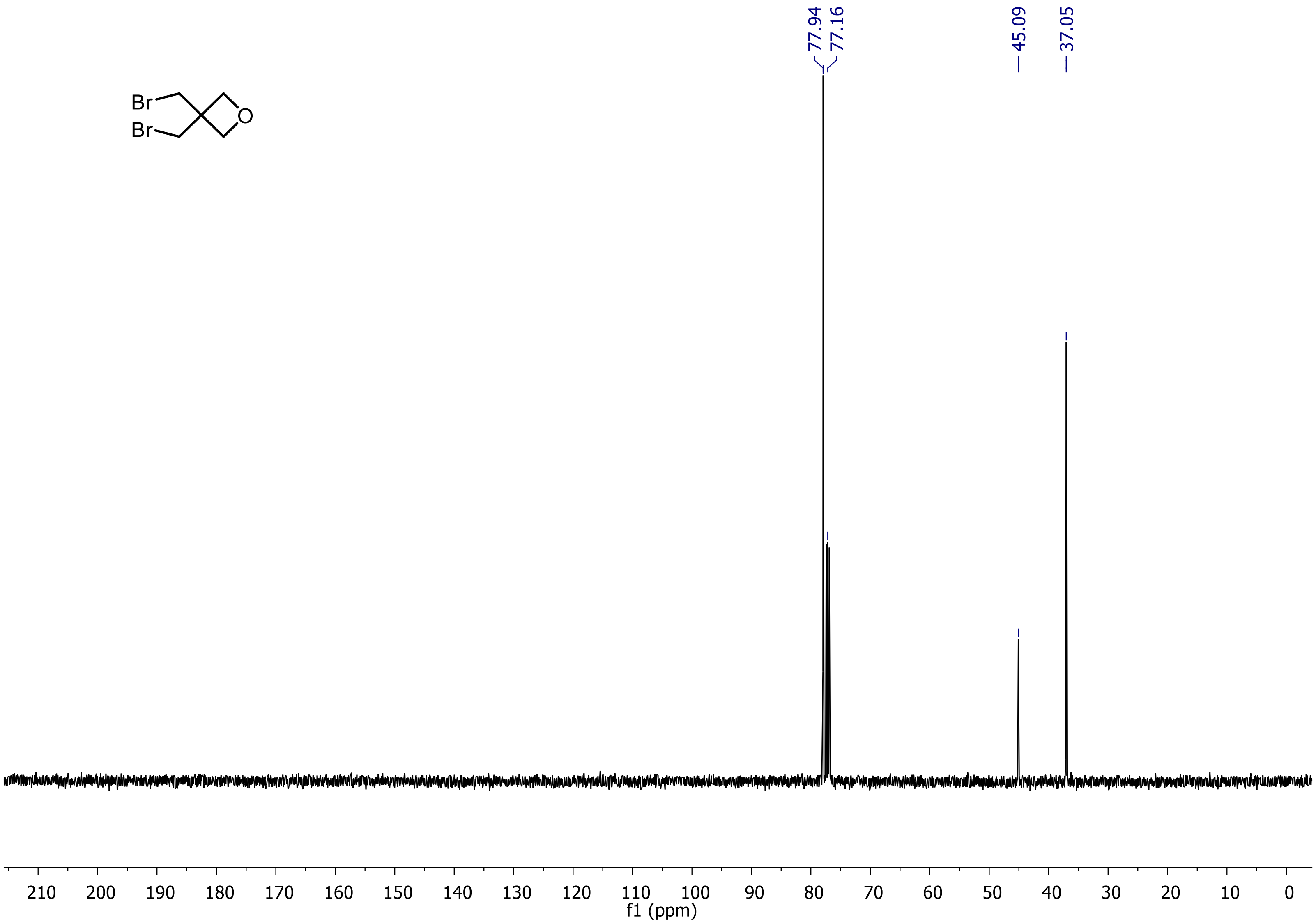

Compound 1

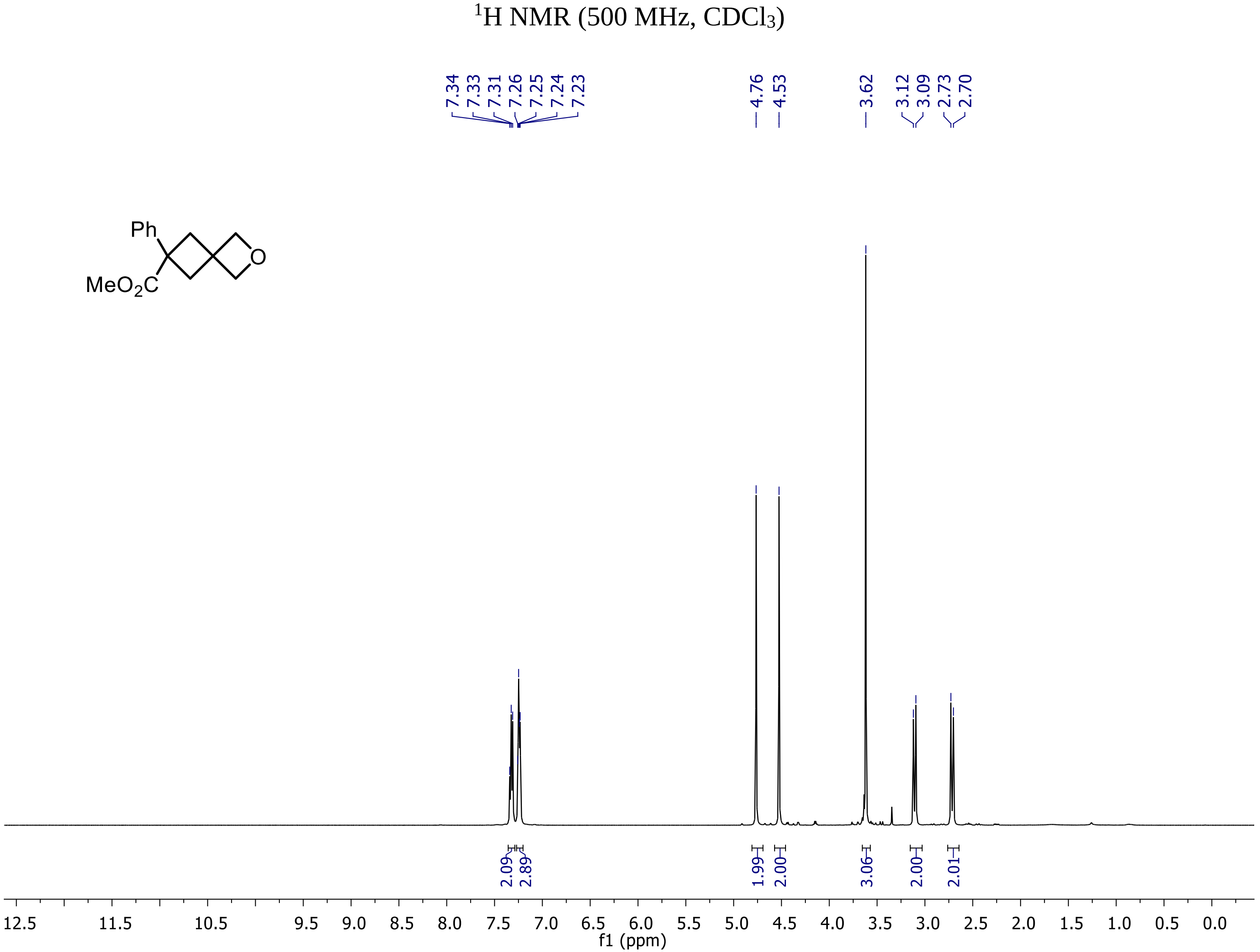

^{1}H NMR (500 MHz, CDCl$_3$)
7.34
7.33
7.31
7.26
7.25
7.24
7.23
4.76
4.53
3.62
3.12
3.09
2.73
2.70
Ph
MeO2C
O
2.09
2.89
1.99
2.00
3.06
2.00
2.01
12.5
11.5
10.5
9.5
9.0
8.5
8.0
7.5
7.0
6.5
6.0
5.5
5.0
4.5
4.0
3.5
3.0
2.5
2.0
1.5
1.0
0.5
0.0
f1 (ppm)

$^{13}C\{^1H\}$ NMR (126 MHz, $CDCl_3$)

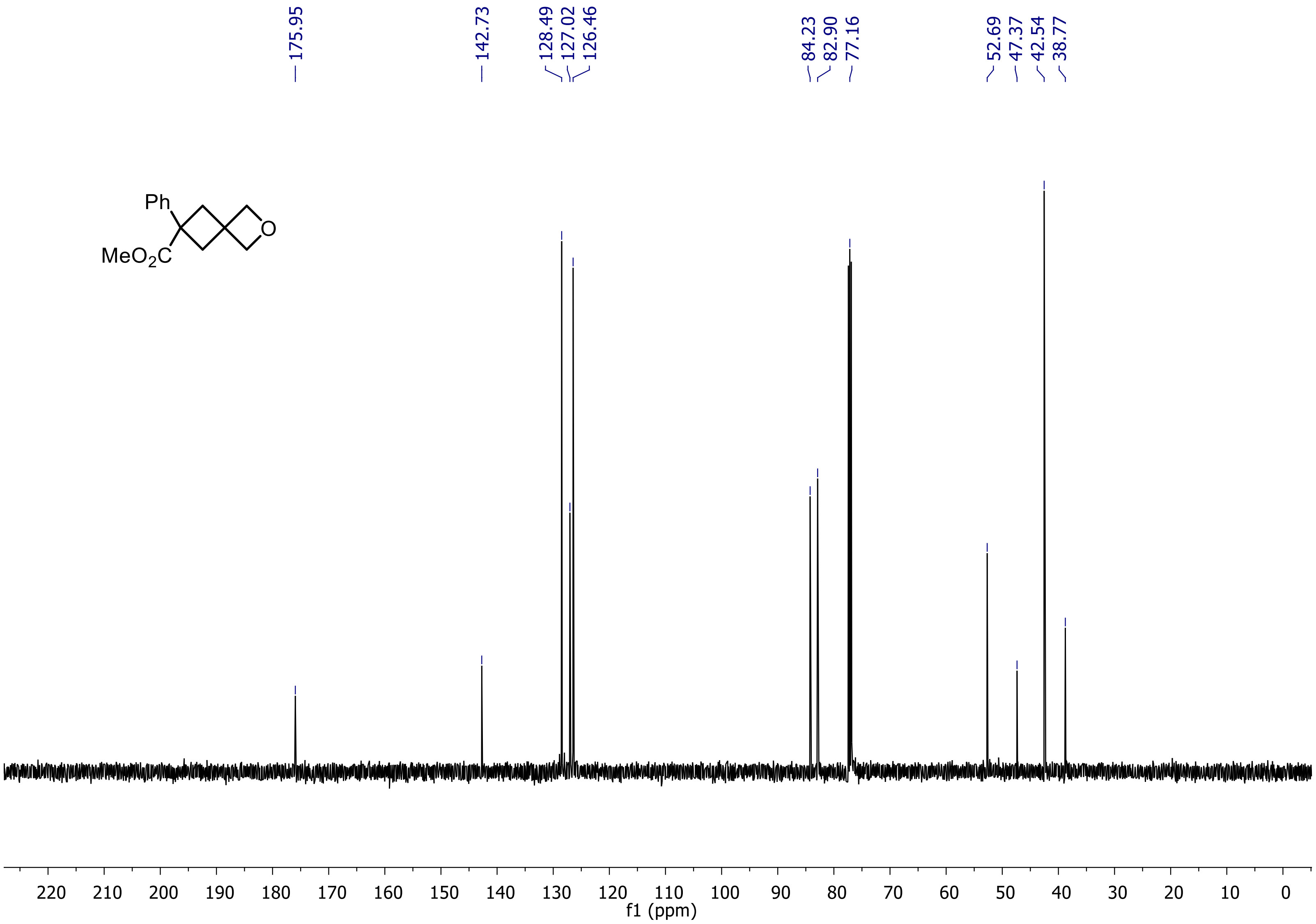

Compound 2

^{1}H NMR (500 MHz, DMSO-d_6)

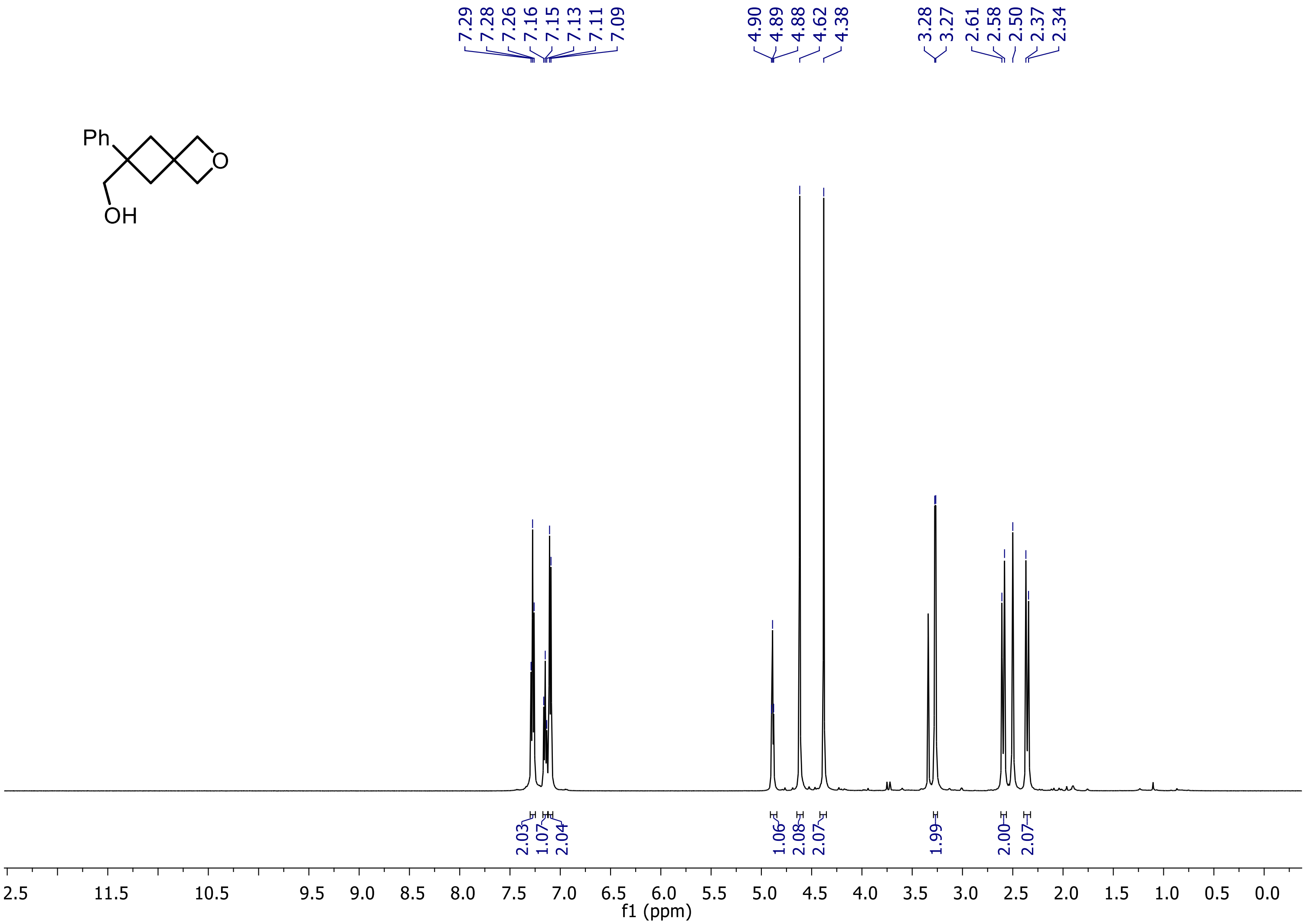

$^{13}C\{^{1}H\}$ NMR (126 MHz, DMSO-d_6)

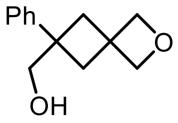

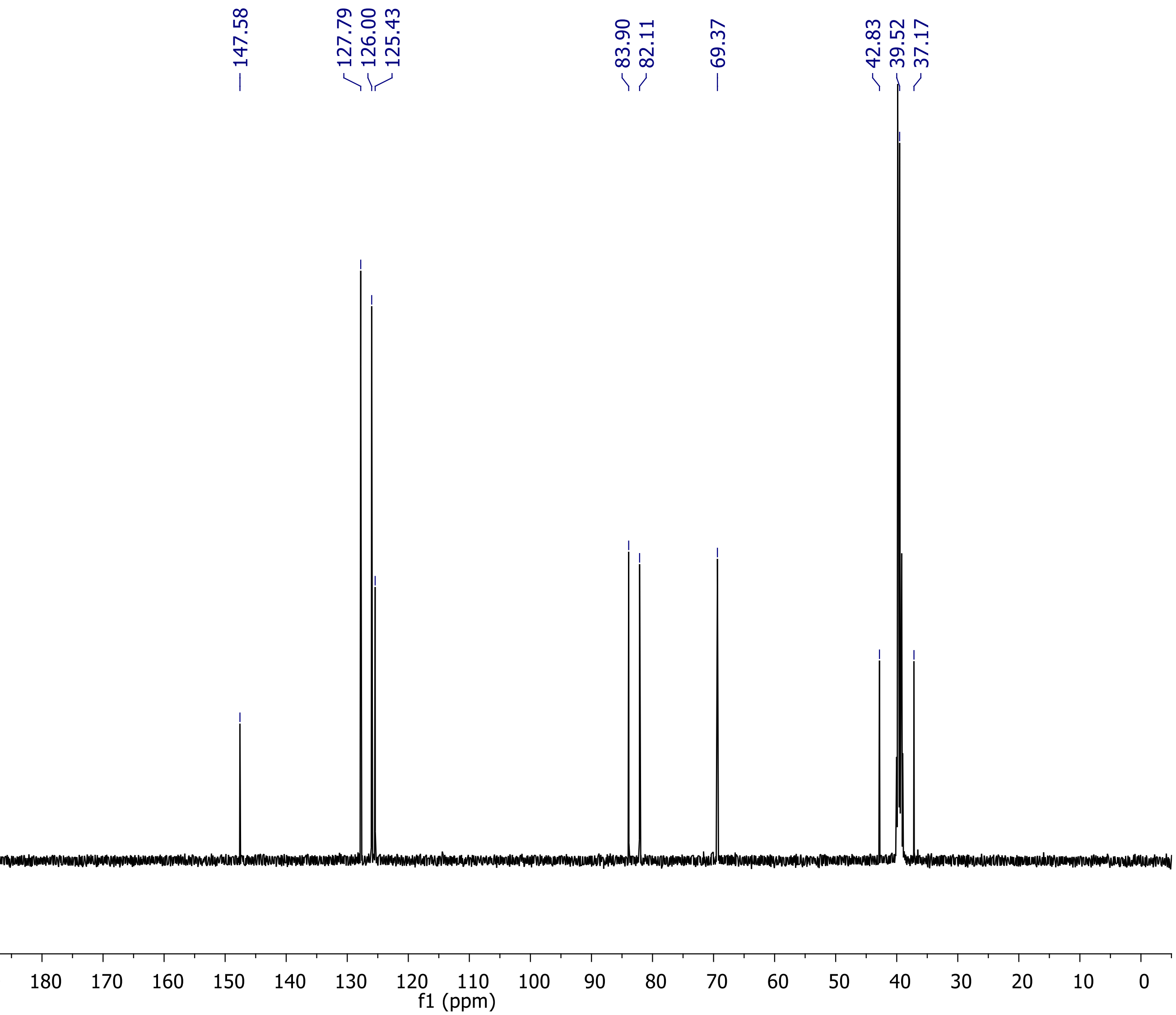

Compound 6

^{1}H NMR (500 MHz, DMSO-d_6)

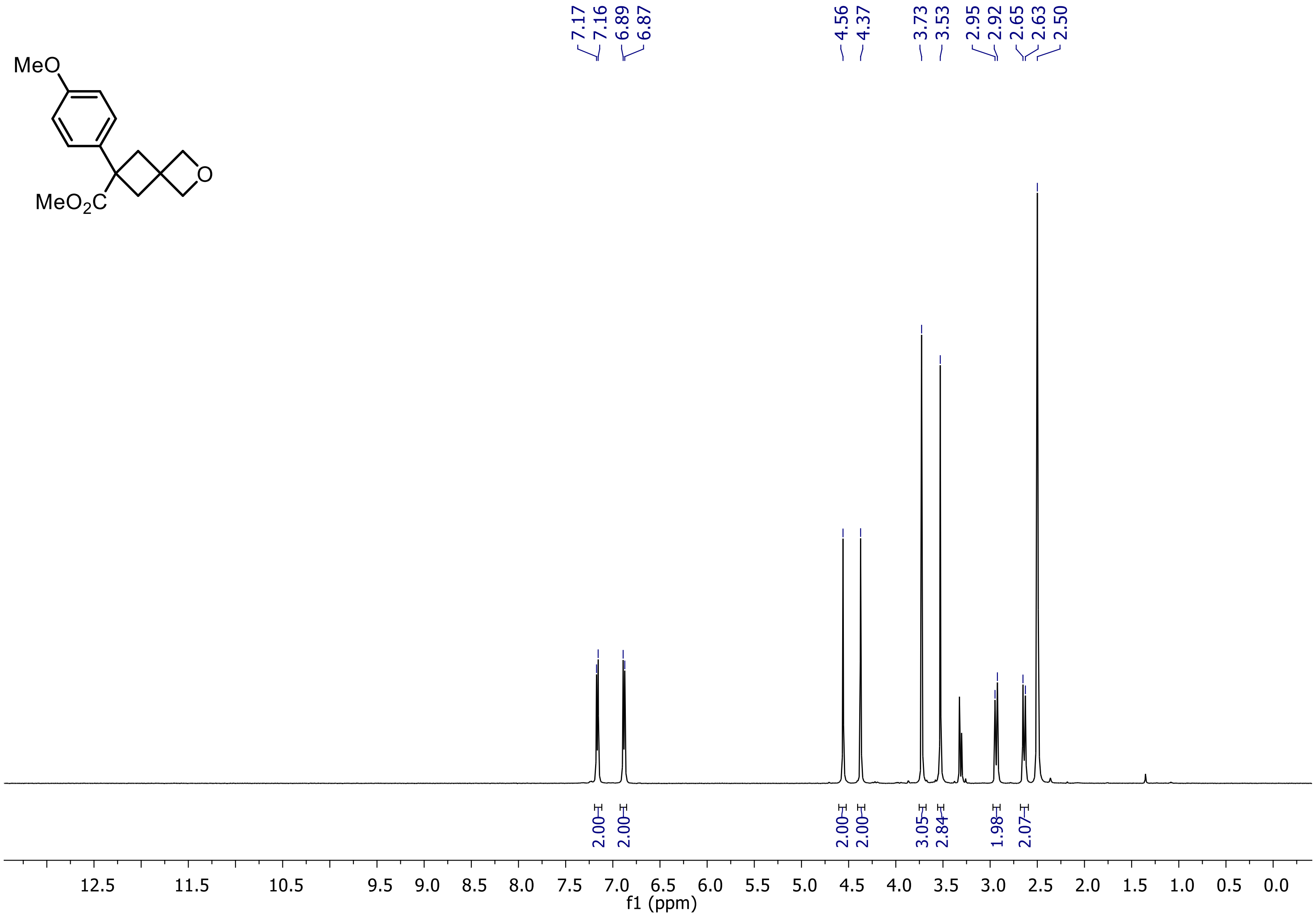

$^{13}C\{^1H\}$ NMR (126 MHz, $CDCl_3$)

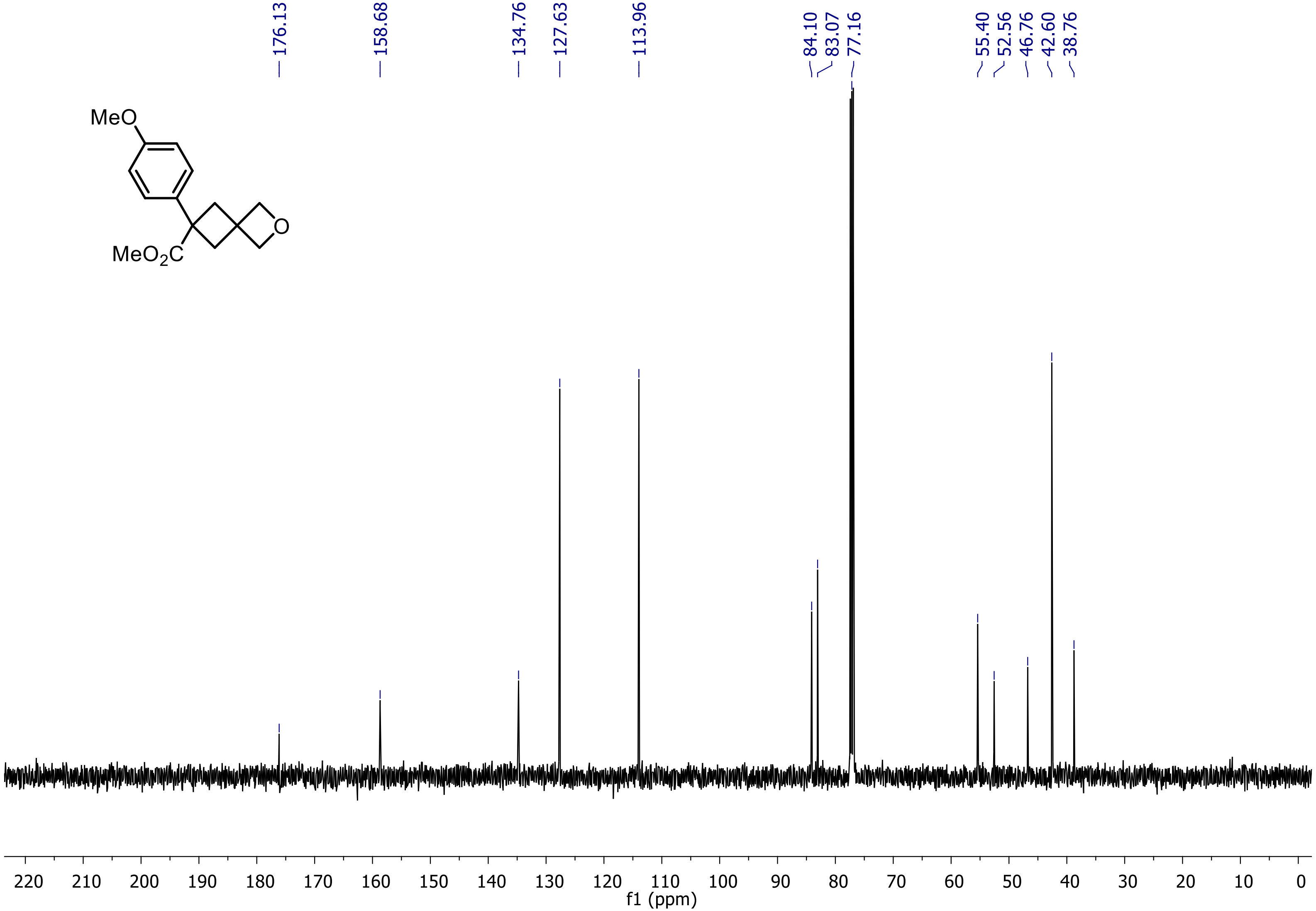

Compound 7

^{1}H NMR (500 MHz, DMSO-d_6)

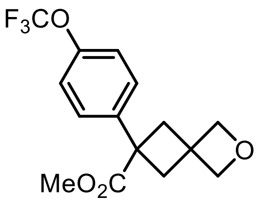

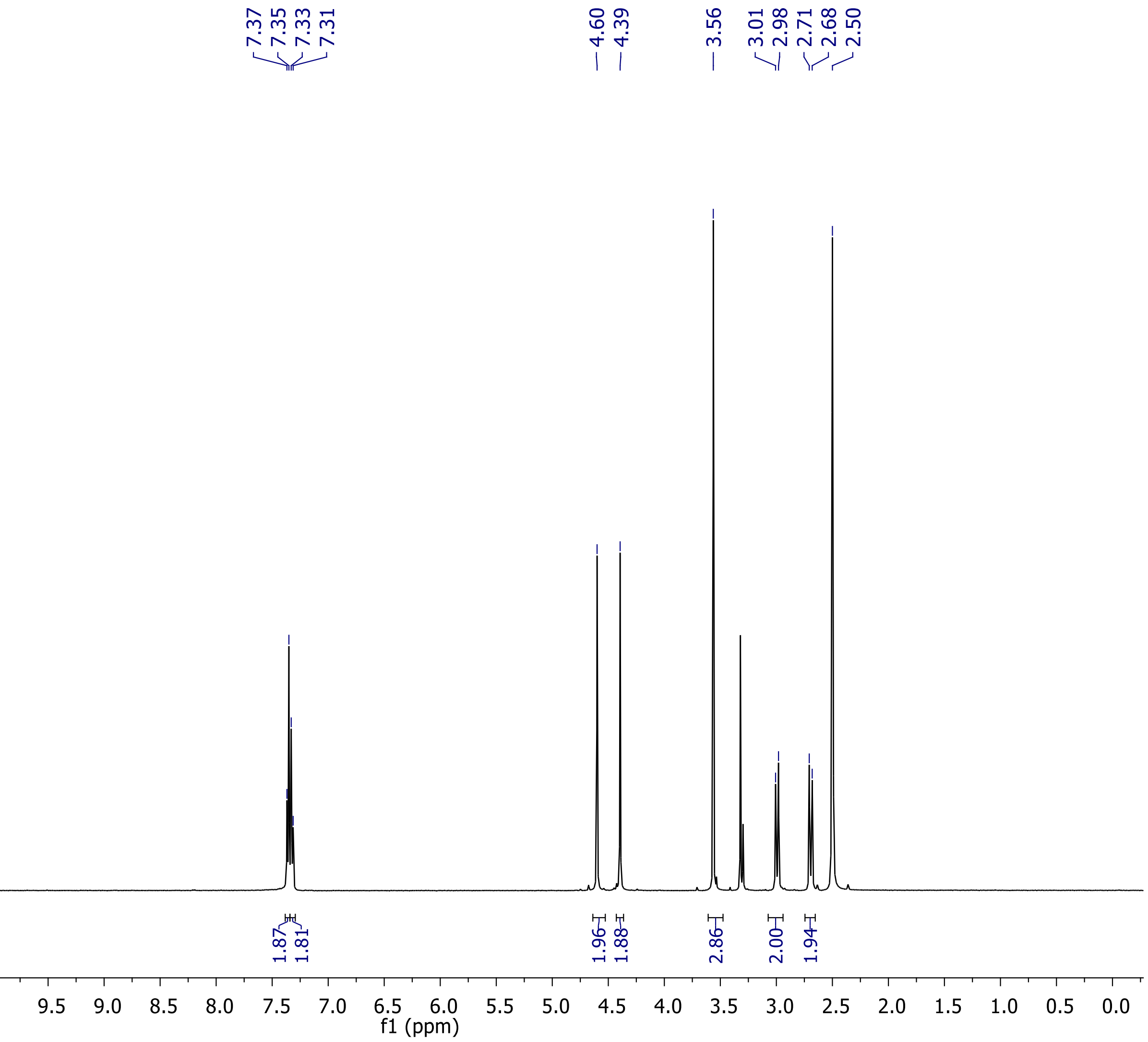

$^{13}C\{^{1}H\}$ NMR (151 MHz, DMSO-d_6)

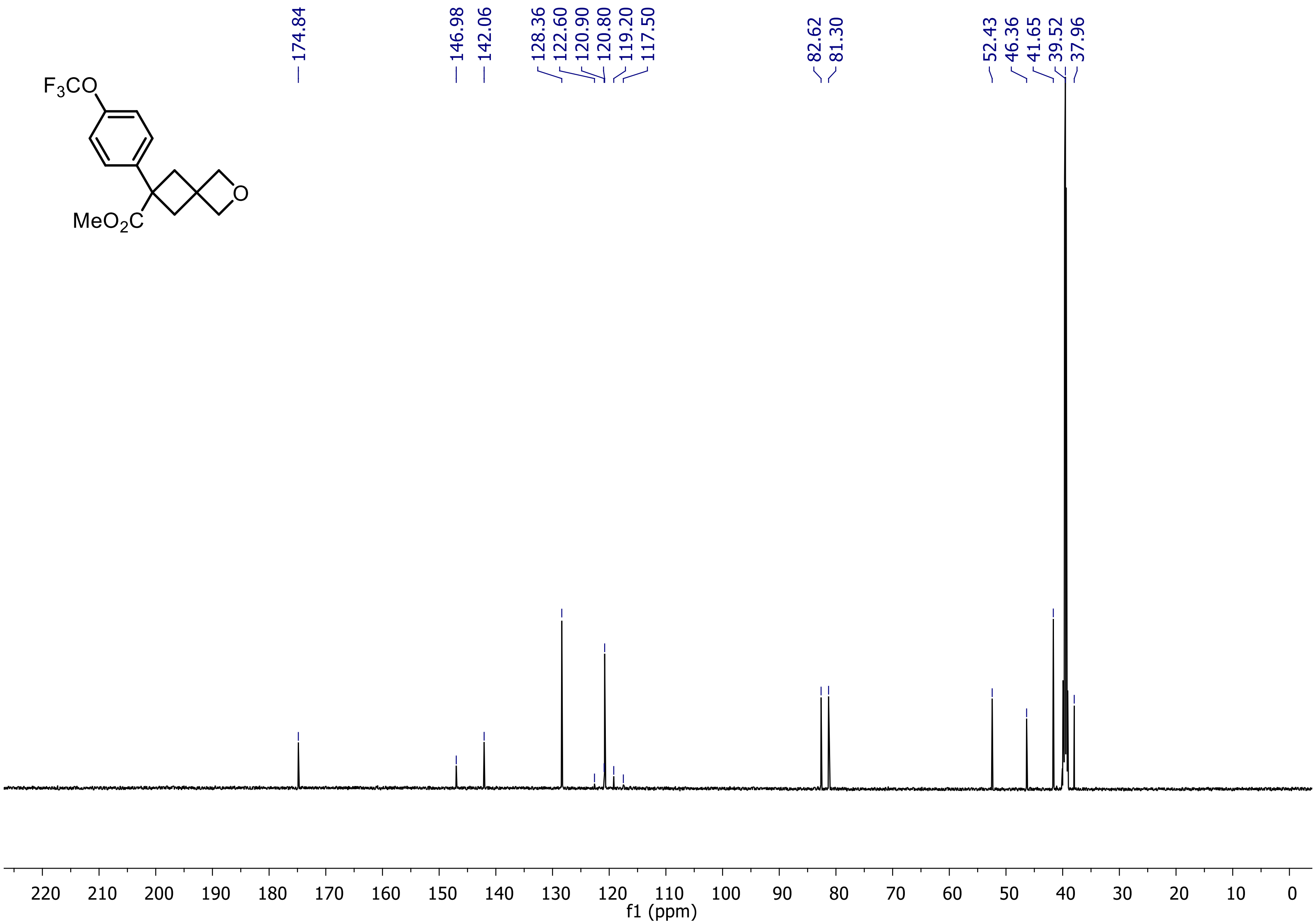

$^{19}F\{^{1}H\}$ NMR (376 MHz, DMSO-d_6)

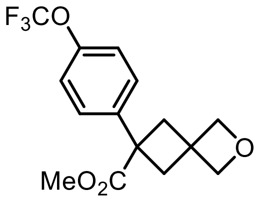

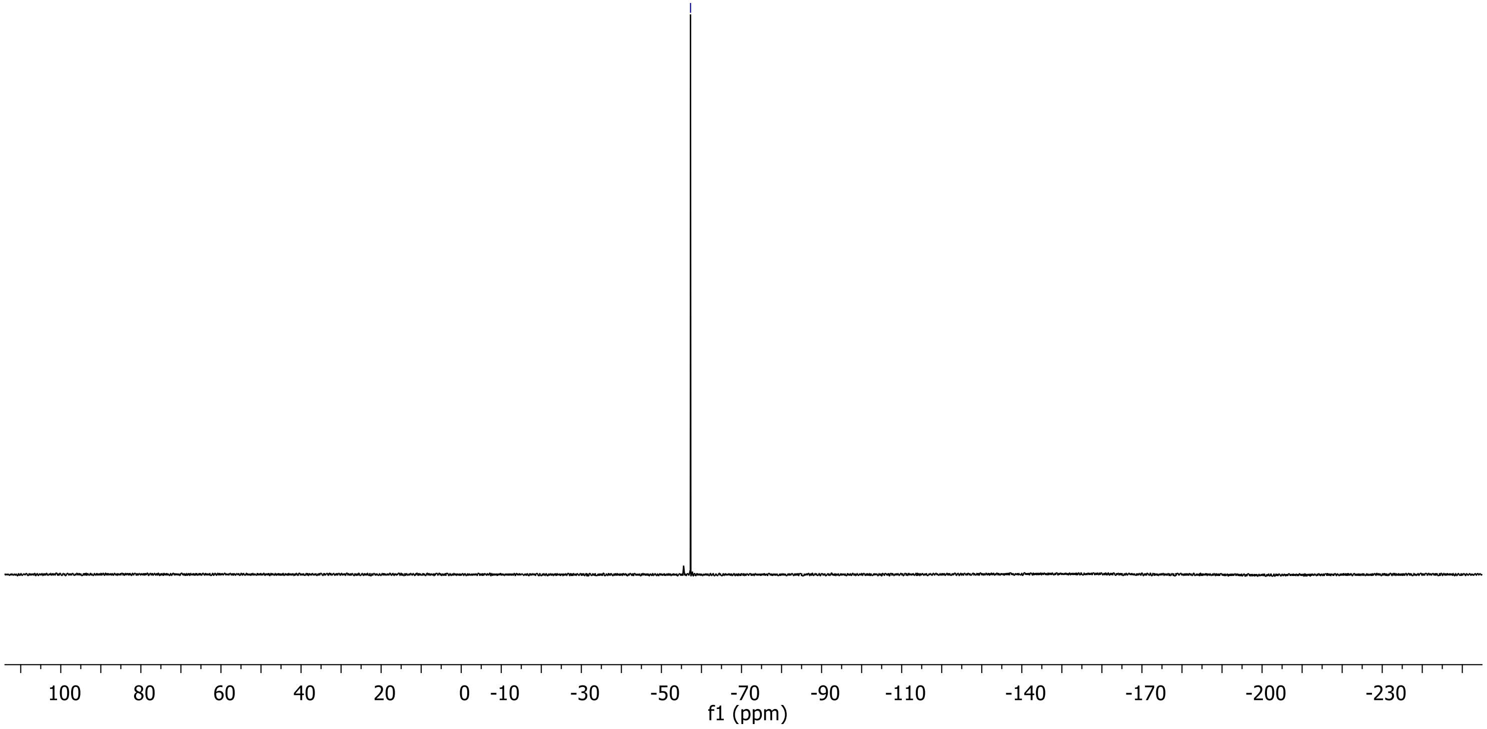

Compound 9

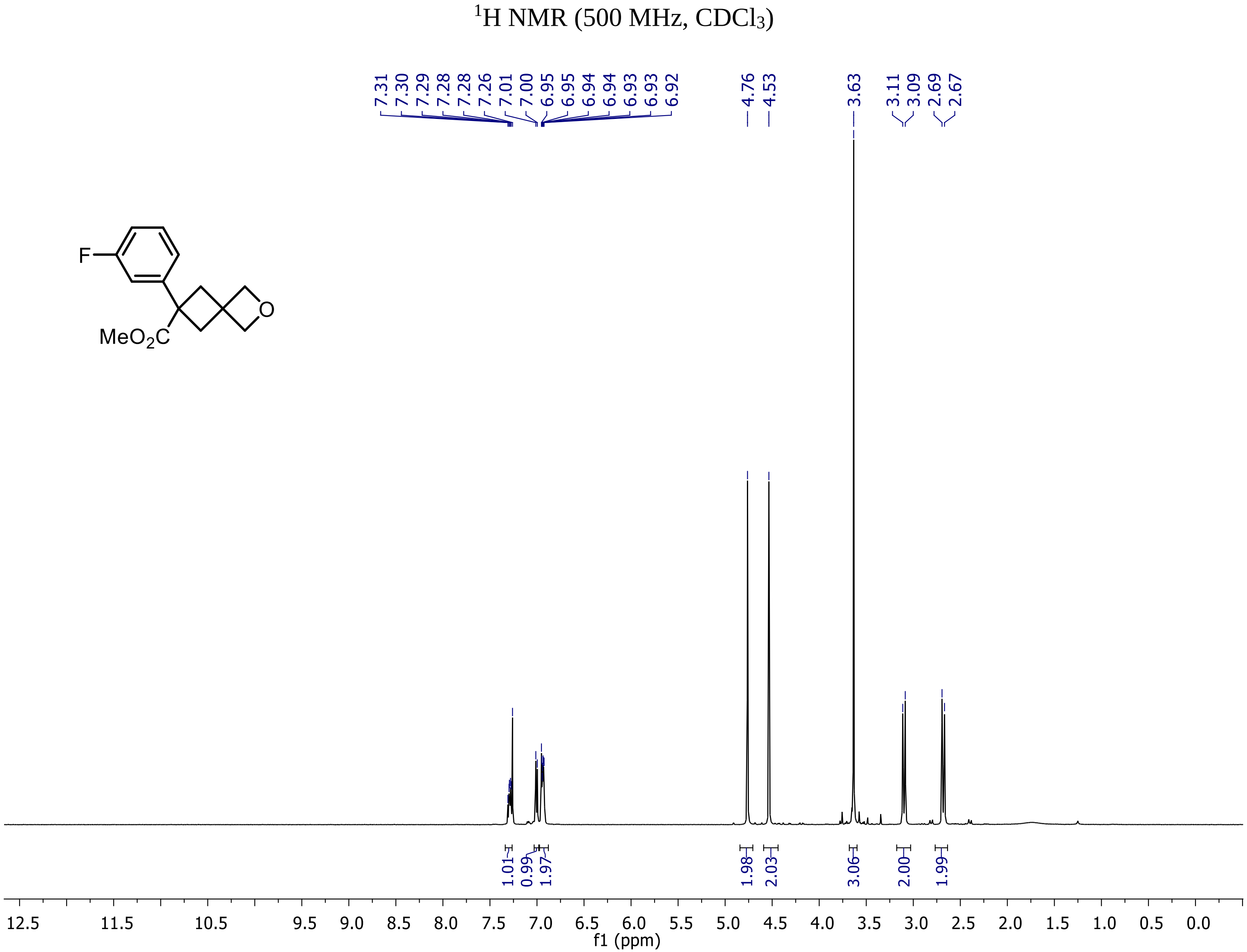

^{1}H NMR (500 MHz, CDCl$_3$)
7.31
7.30
7.29
7.28
7.28
7.26
7.01
7.00
6.95
6.95
6.94
6.94
6.93
6.93
6.92
4.76
4.53
3.63
3.11
3.09
2.69
2.67
1.01
0.99
1.97
1.98
2.03
3.06
2.00
1.99
12.5
11.5
10.5
9.5
9.0
8.5
8.0
7.5
7.0
6.5
6.0
5.5
5.0
4.5
4.0
3.5
3.0
2.5
2.0
1.5
1.0
0.5
0.0
f1 (ppm)

$^{13}C\{^{1}H\}$ NMR (151 MHz, $CDCl_3$)

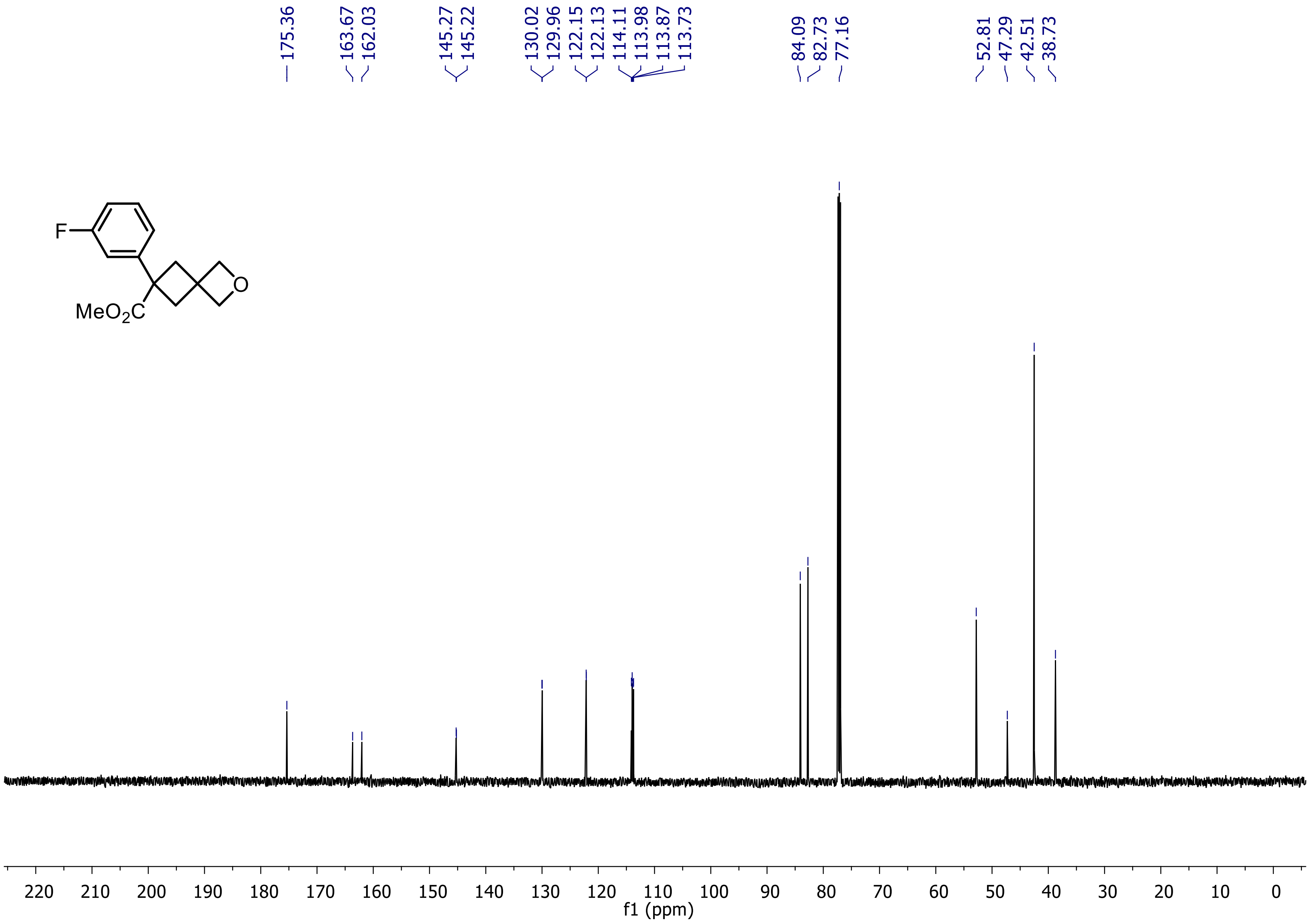

$^{19}F\{^1H\}$ NMR (376 MHz, $CDCl_3$)

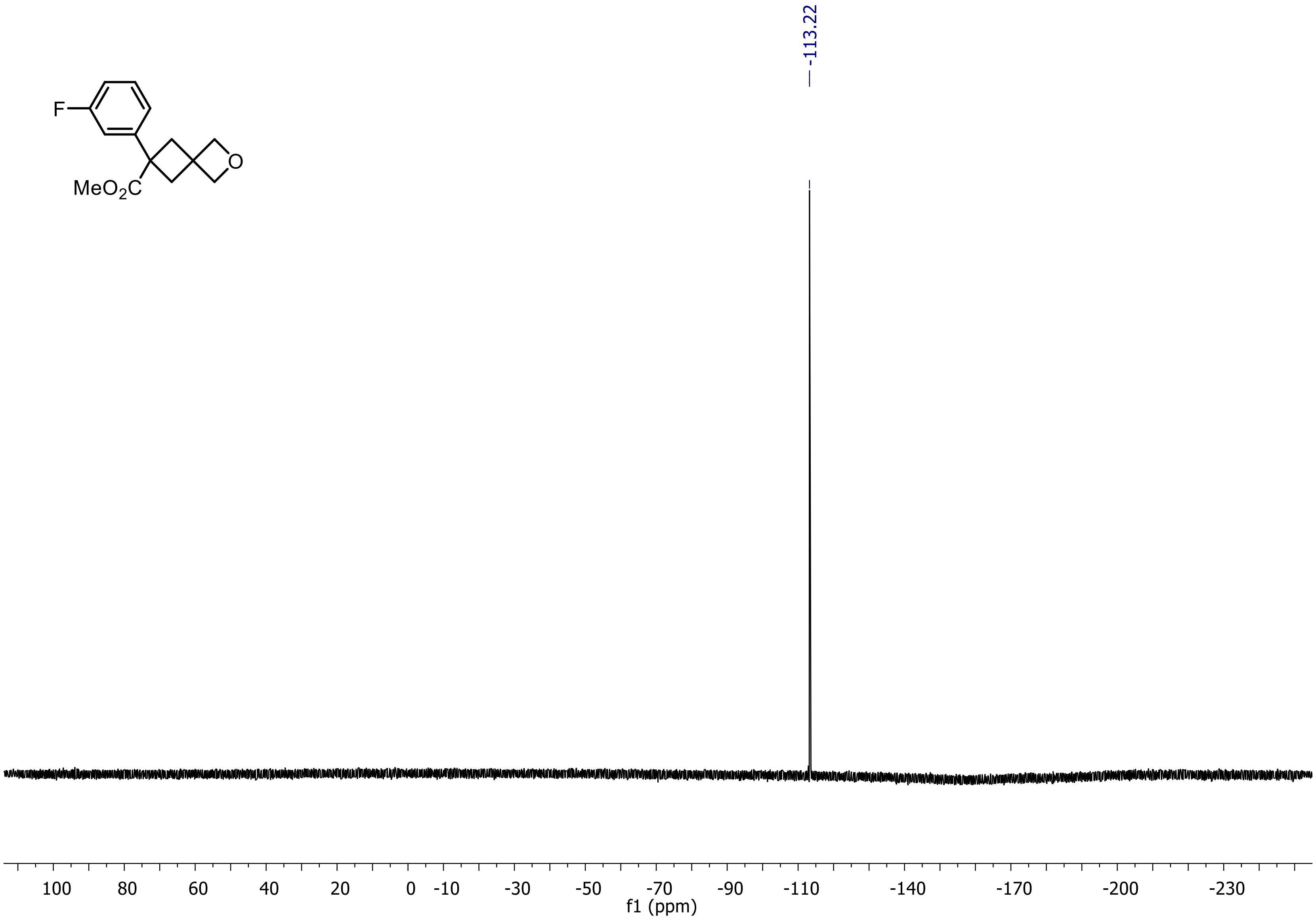

F
O
MeO2C
-113.22
100
80
60
40
20
0
-10
-30
-50
-70
-90
-110
-140
-170
-200
-230
f1 (ppm)

Compound 13

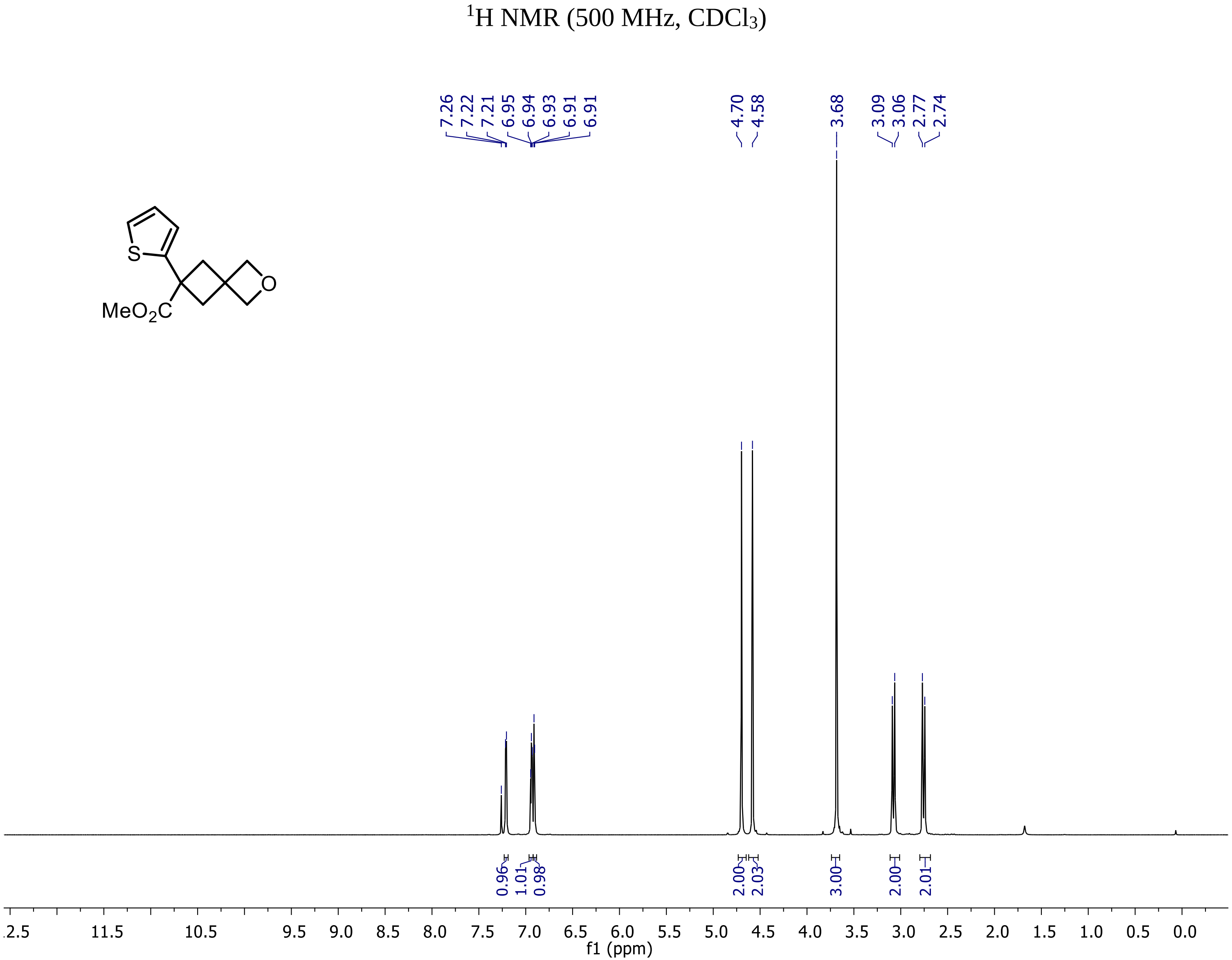
S
O
MeO2C
1H NMR (500 MHz, CDCl3)
7.26
7.22
7.21
6.95
6.94
6.93
6.91
6.91
4.70
4.58
3.68
3.09
3.06
2.77
2.74
0.96
1.01
0.98
2.00
2.03
3.00
2.00
2.01
f1 (ppm)

$^{13}C\{^{1}H\}$ NMR (151 MHz, $CDCl_3$)

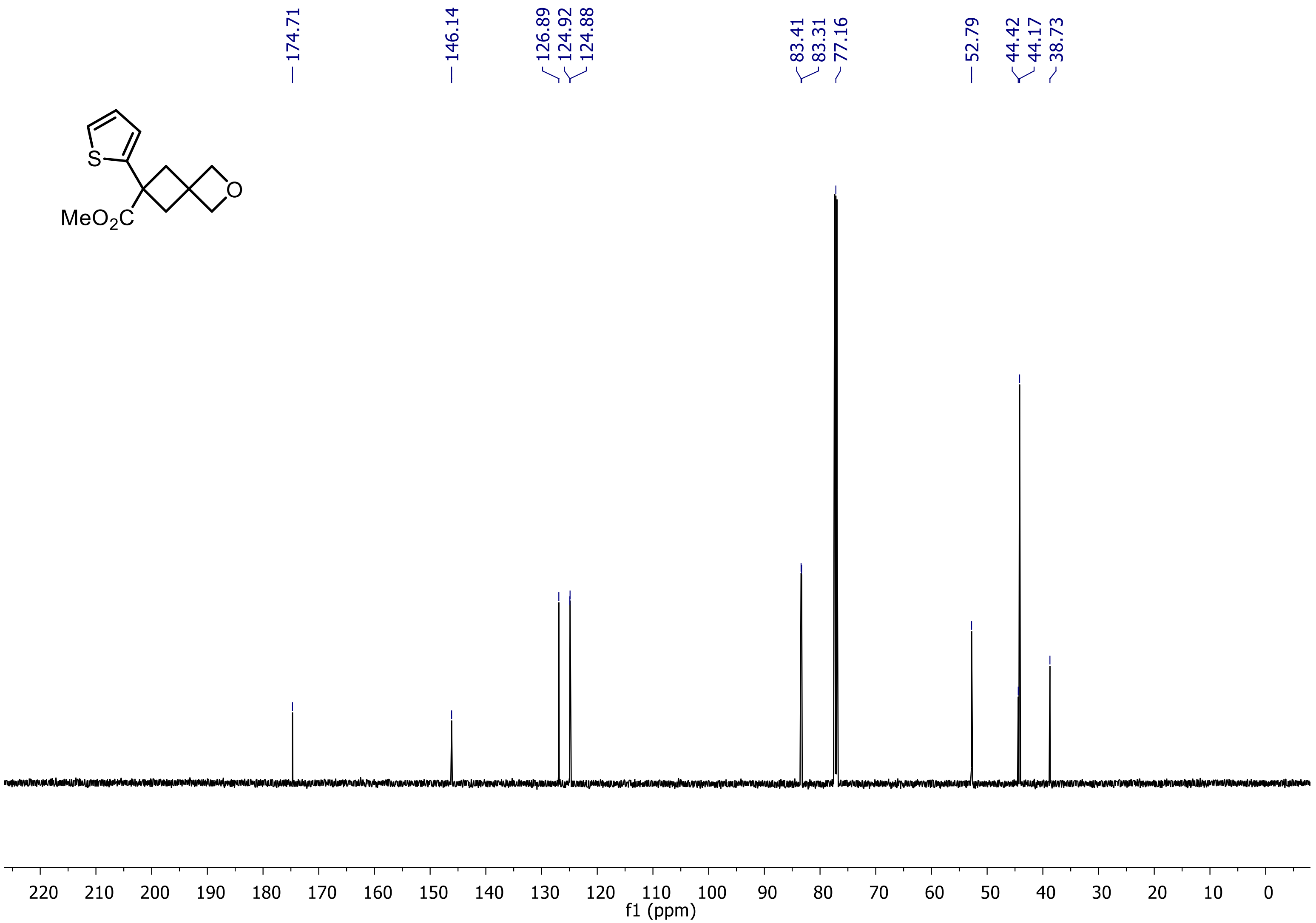

Compound 15

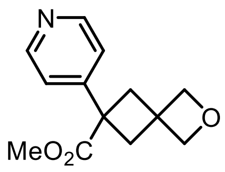
MeO2C
N
O

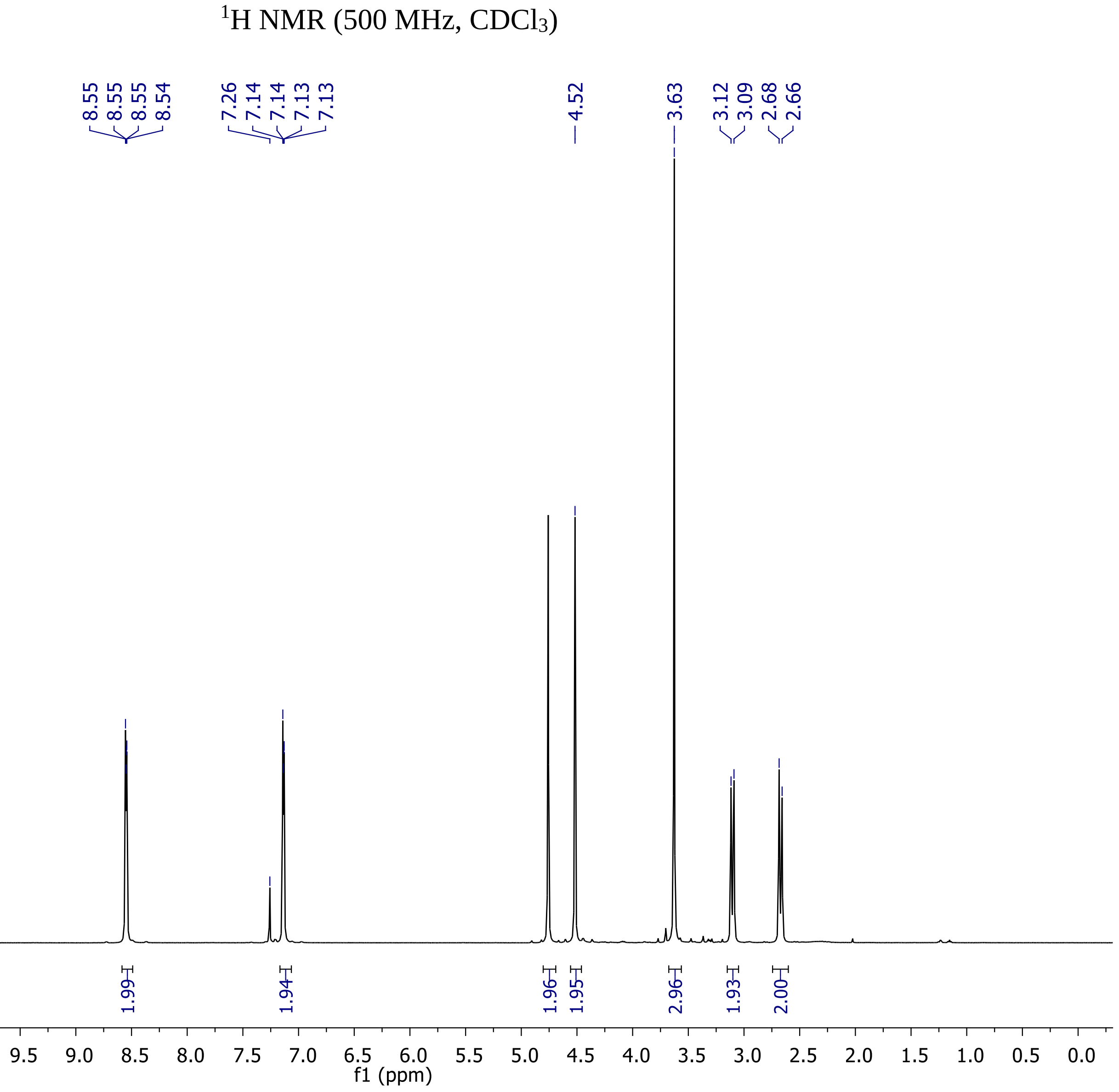
^{1}H NMR (500 MHz, CDCl$_3$)
8.55
8.55
8.55
8.54
7.26
7.14
7.14
7.13
7.13
4.52
3.63
3.12
3.09
2.68
2.66
1.99
1.94
1.96
1.95
2.96
1.93
2.00
f1 (ppm)

$^{13}C\{^1H\}$ NMR (126 MHz, $CDCl_3$)

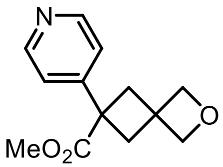

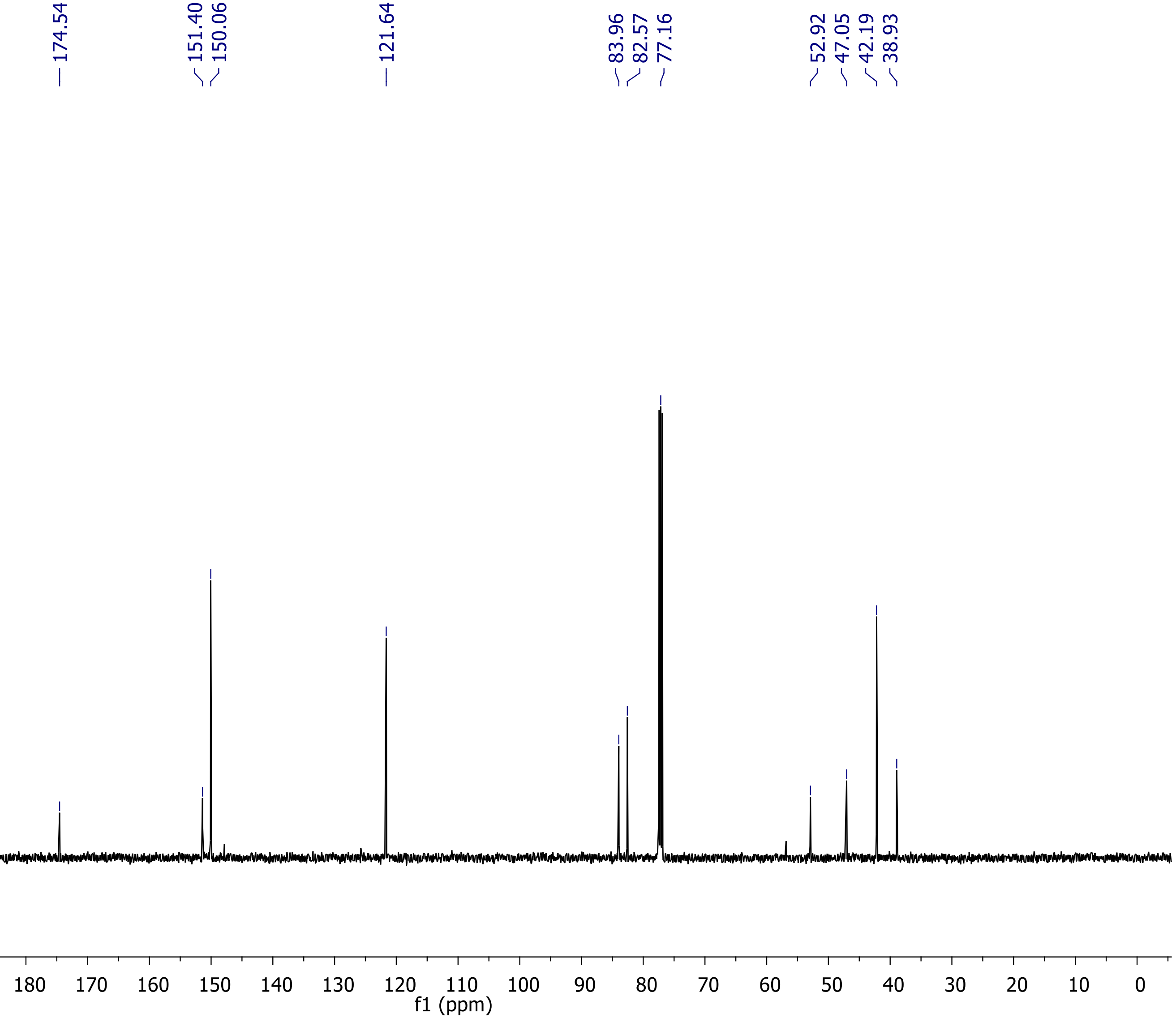

Compound 18

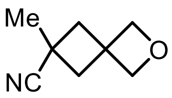
Me
NC
O

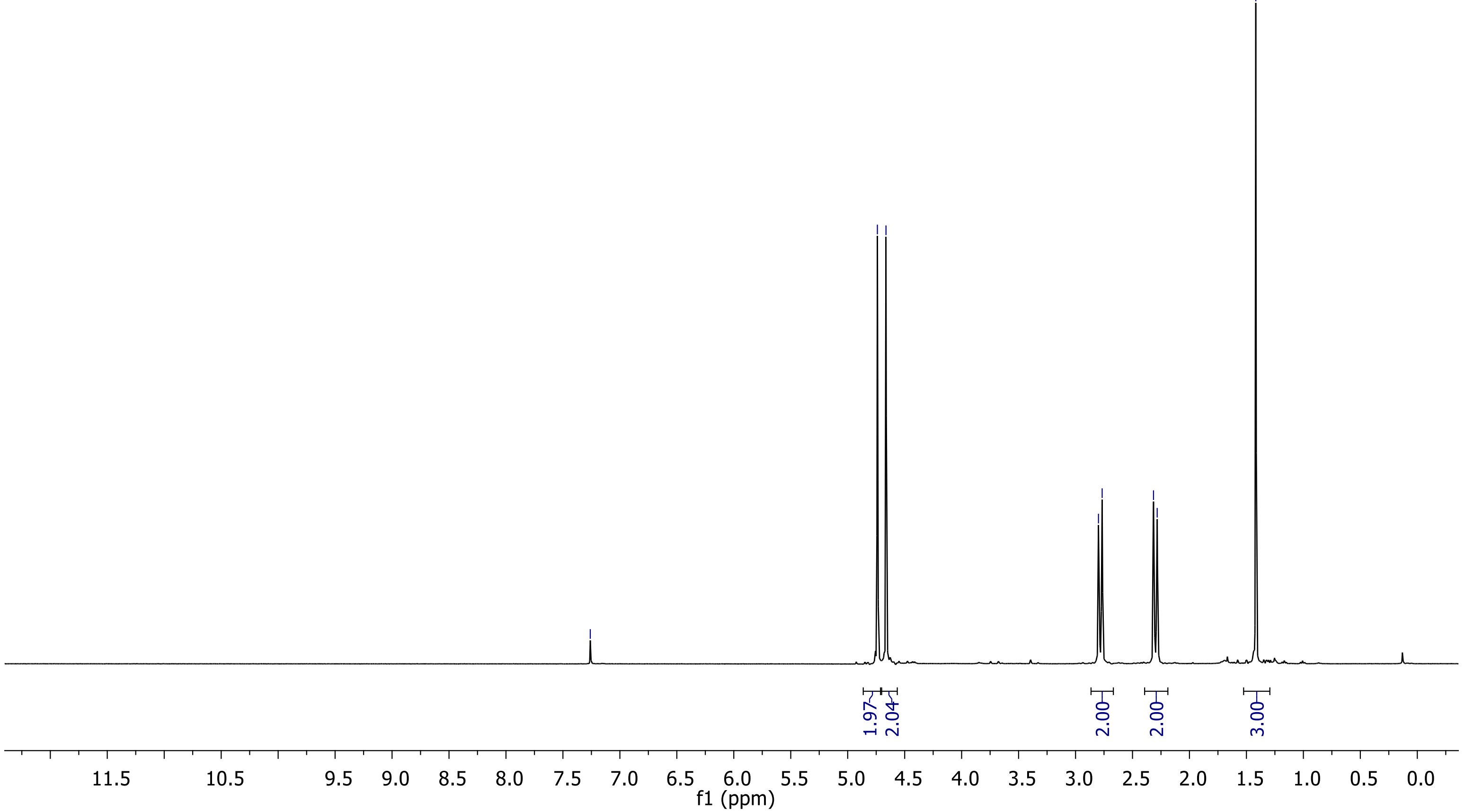
^{1}H NMR (400 MHz, $CDCl_3$)
7.26
4.74
4.66
2.80
2.77
2.32
2.28
1.42
1.97
2.04
2.00
2.00
3.00
11.5
10.5
9.5
9.0
8.5
8.0
7.5
7.0
6.5
6.0
5.5
5.0
4.5
4.0
3.5
3.0
2.5
2.0
1.5
1.0
0.5
0.0
f1 (ppm)

$^{13}C\{^1H\}$ NMR (126 MHz, $CDCl_3$)

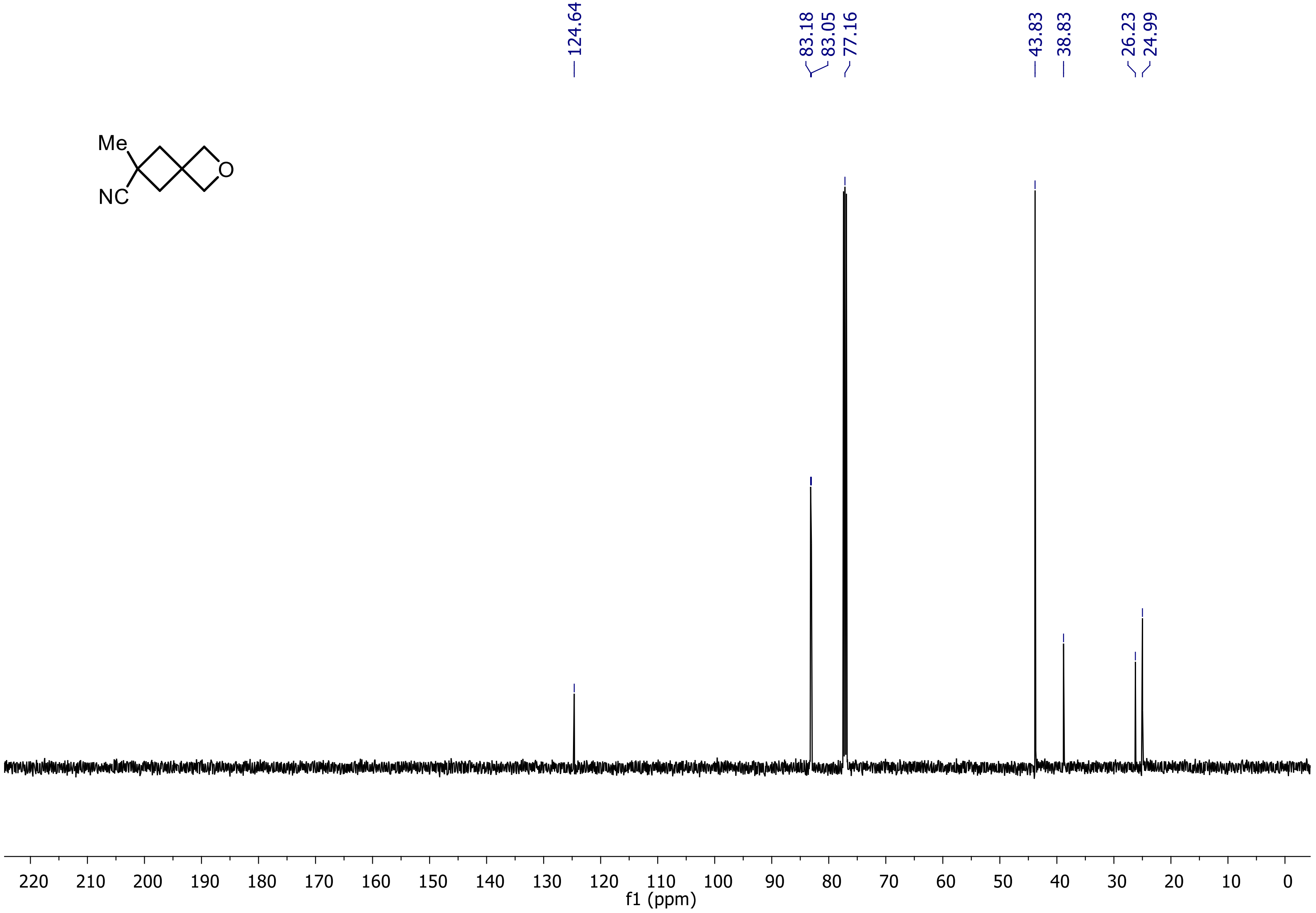

Compound 19

^{1}H NMR (500 MHz, $CDCl_3$)

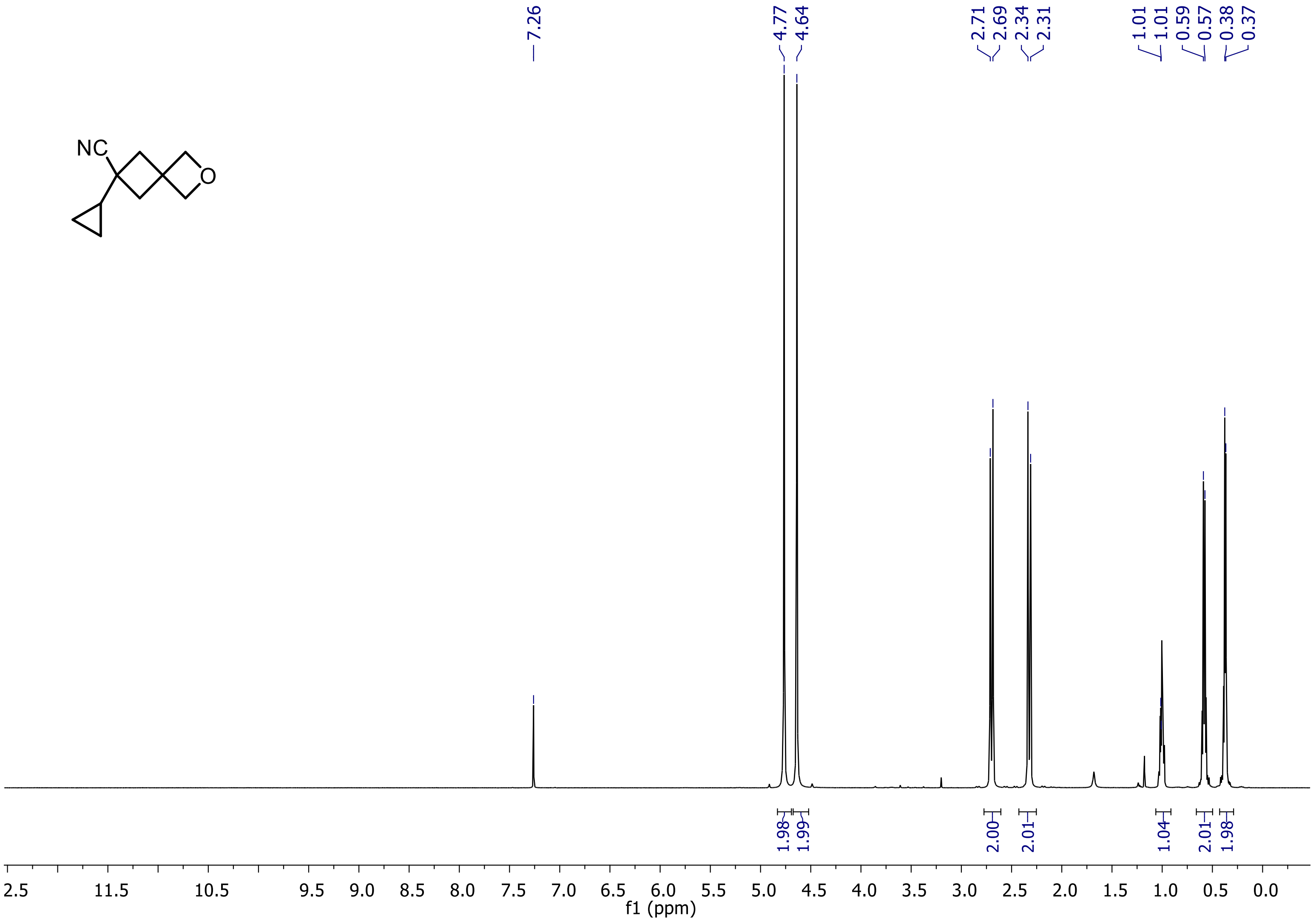

$^{13}C\{^{1}H\}$ NMR (126 MHz, $CDCl_3$)

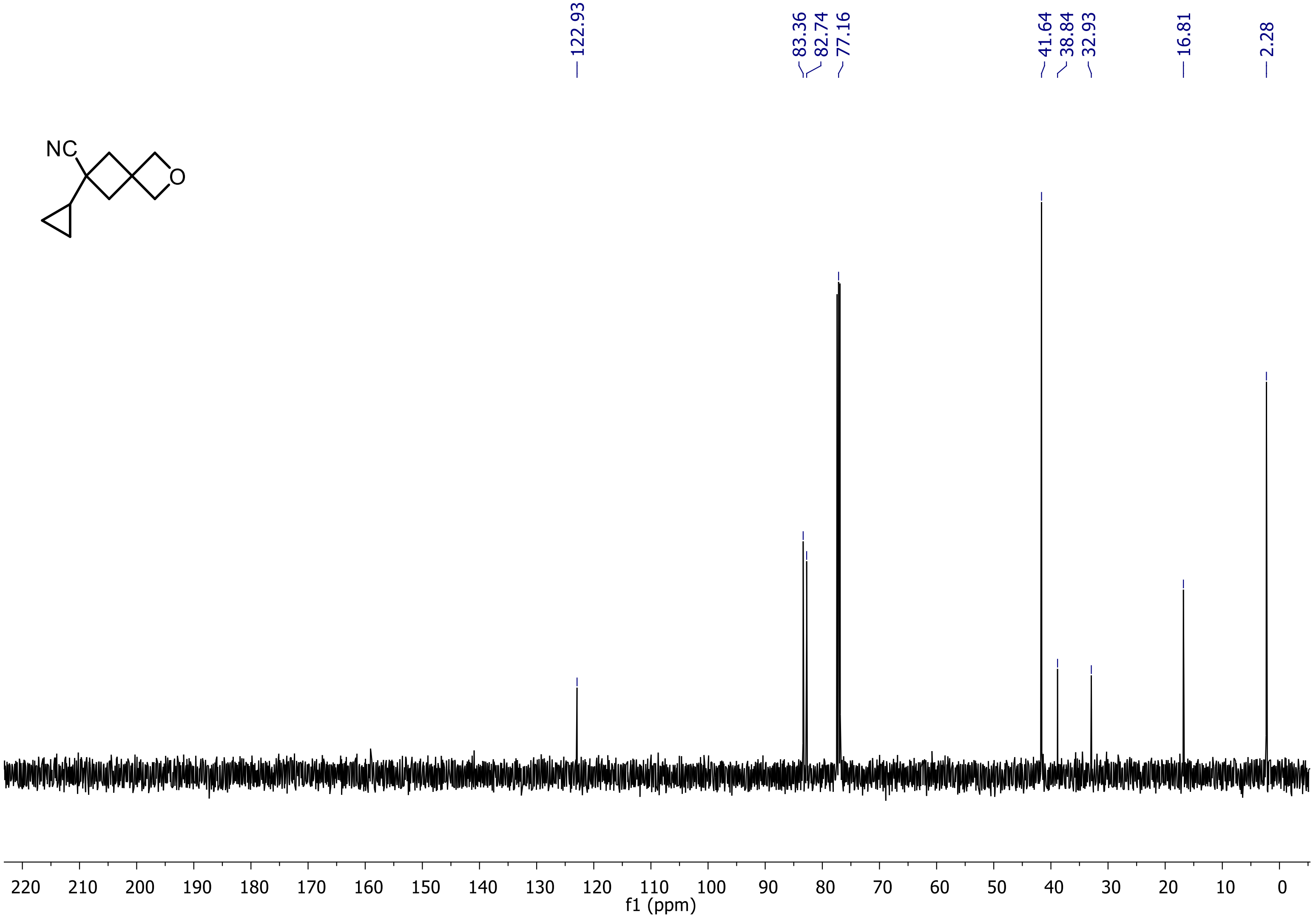

Compound 20

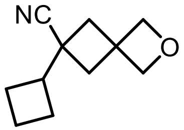
NC
O

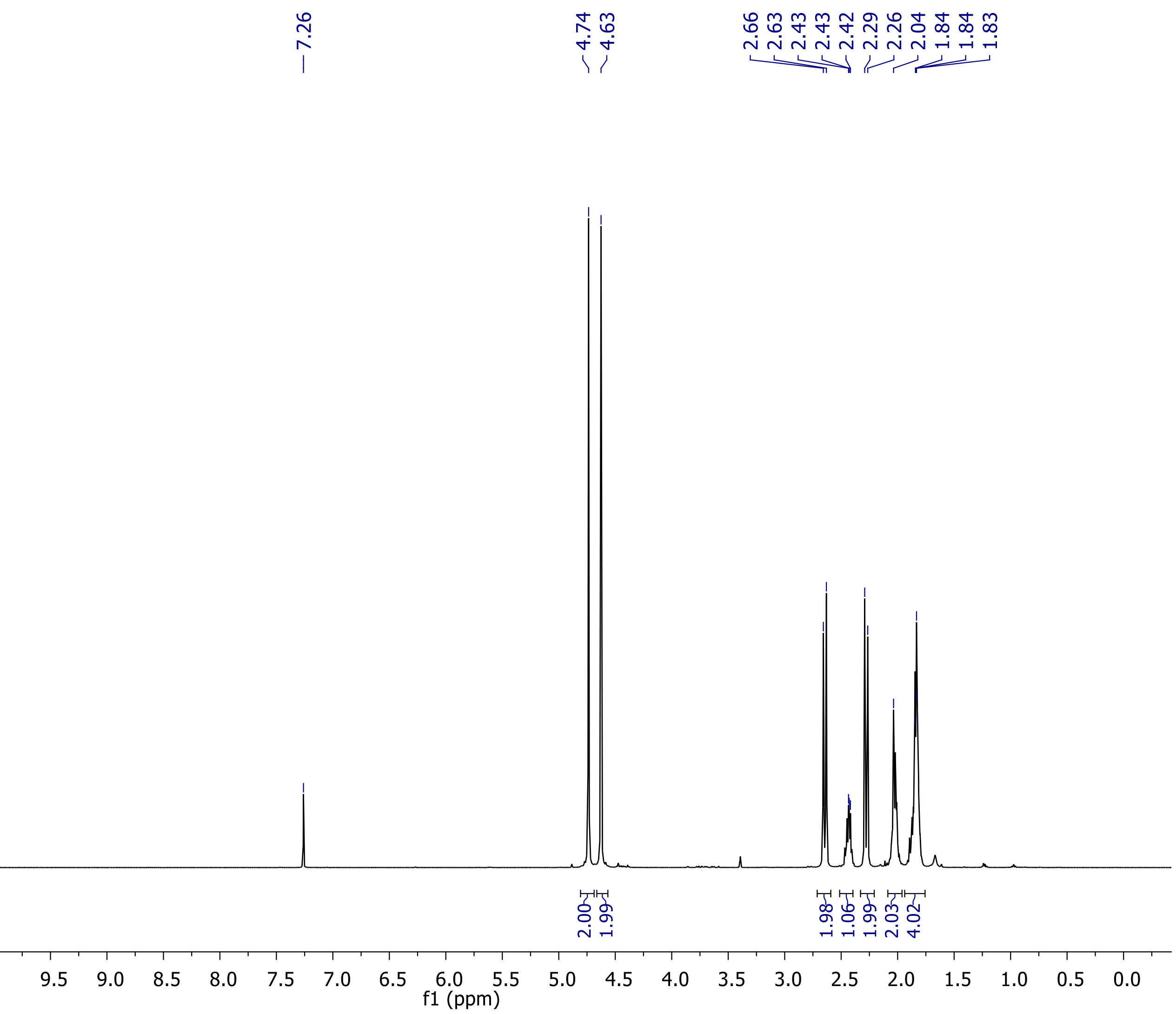
^{1}H NMR (500 MHz, CDCl$_3$)
7.26
4.74
4.63
2.66
2.63
2.43
2.43
2.42
2.29
2.26
2.04
1.84
1.84
1.83
2.00
1.99
1.98
1.06
1.99
2.03
4.02
11.5
10.5
9.5
9.0
8.5
8.0
7.5
7.0
6.5
6.0
5.5
5.0
4.5
4.0
3.5
3.0
2.5
2.0
1.5
1.0
0.5
0.0
f1 (ppm)

$^{13}C\{^{1}H\}$ NMR (126 MHz, $CDCl_3$)

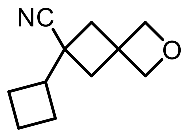

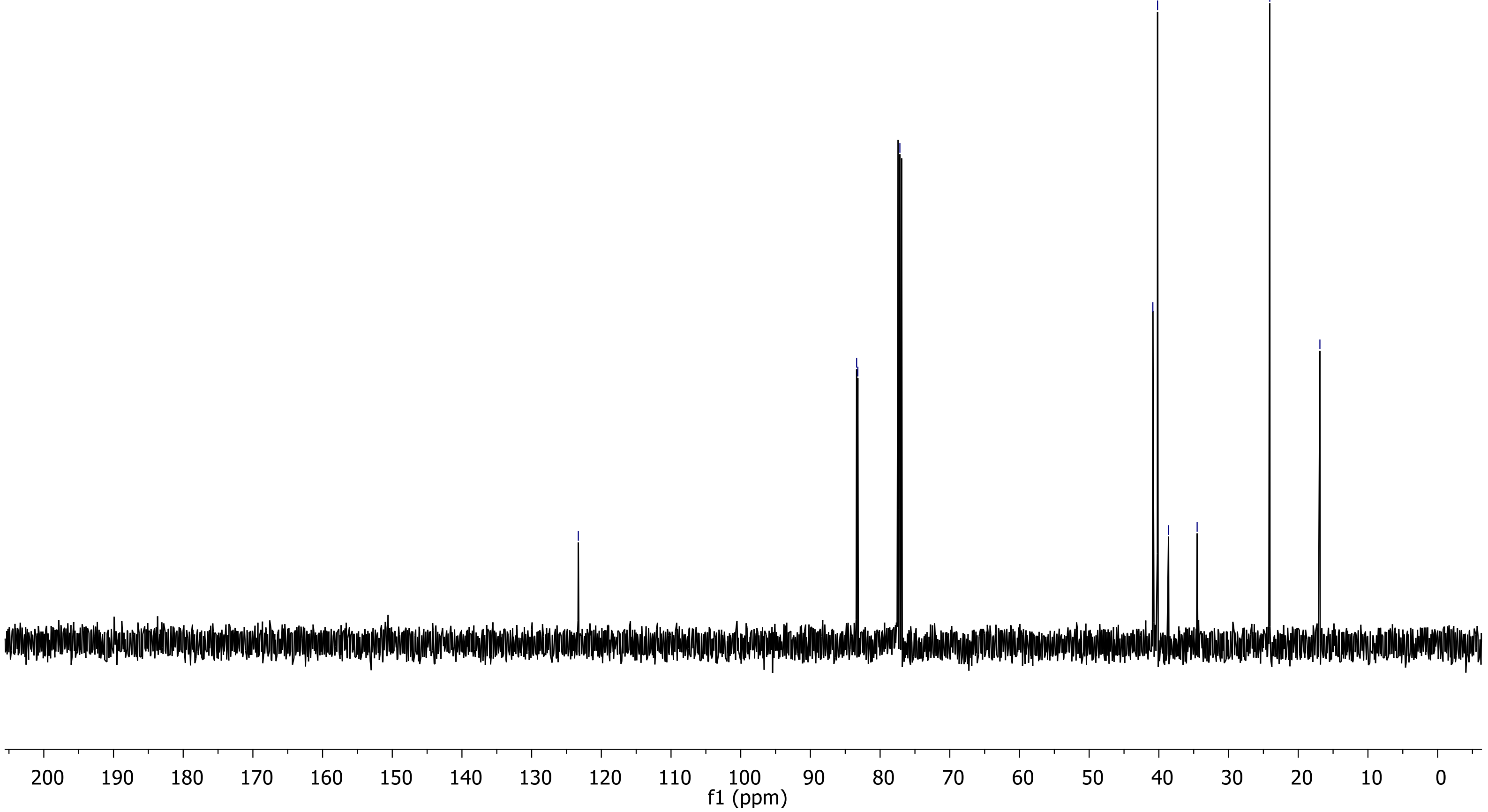

Compound 22

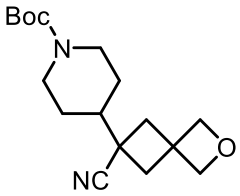

Boc
N
O
NC

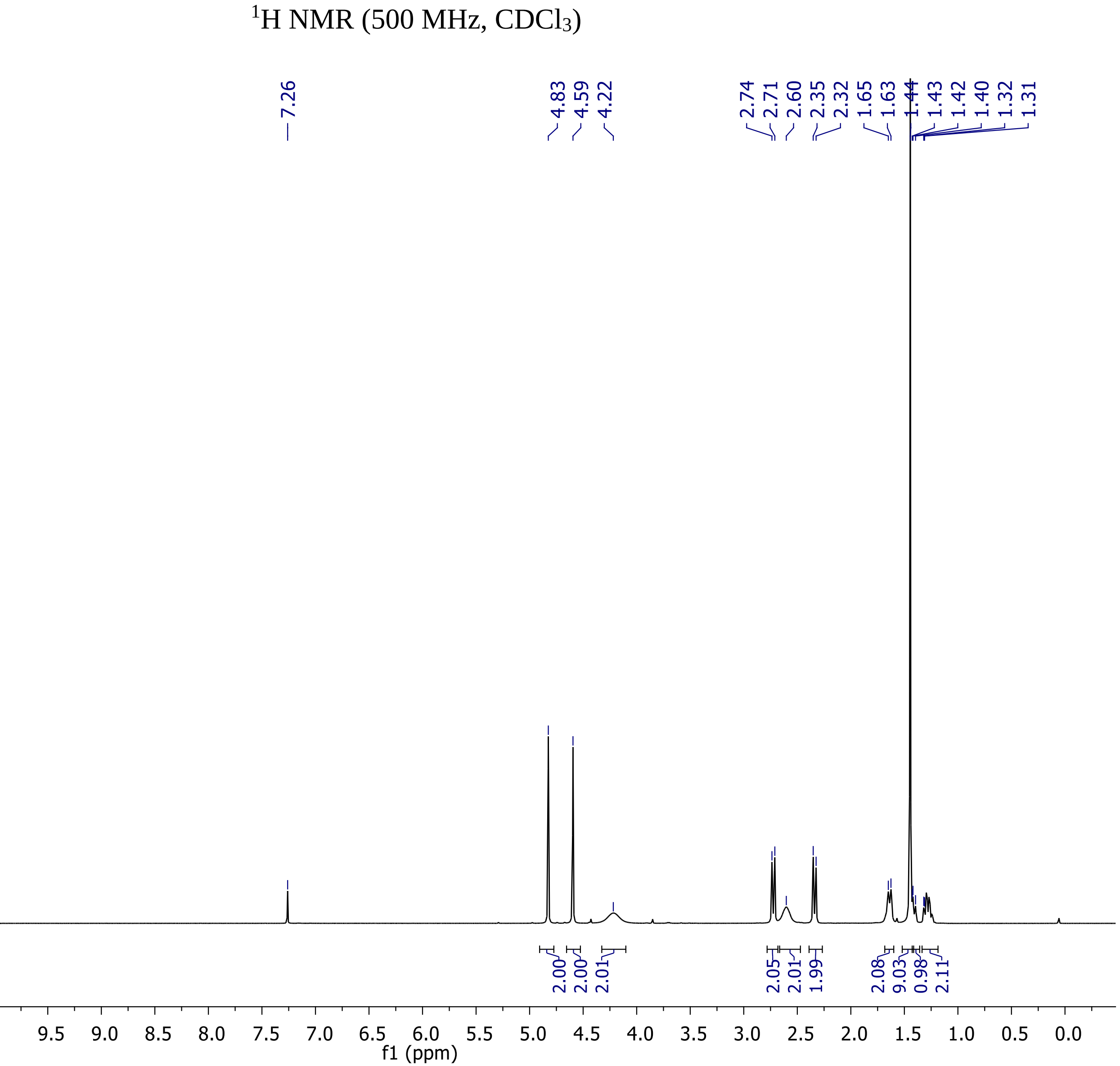

^{1}H NMR (500 MHz, $CDCl_3$)
7.26
4.83
4.59
4.22
2.74
2.71
2.60
2.35
2.32
1.65
1.63
1.44
1.43
1.42
1.40
1.32
1.31
2.00
2.00
2.01
2.05
2.01
1.99
2.08
9.03
0.98
2.11
12.5
11.5
10.5
9.5
9.0
8.5
8.0
7.5
7.0
6.5
6.0
5.5
5.0
4.5
4.0
3.5
3.0
2.5
2.0
1.5
1.0
0.5
0.0
f1 (ppm)

$^{13}C\{^{1}H\}$ NMR (126 MHz, $CDCl_3$)

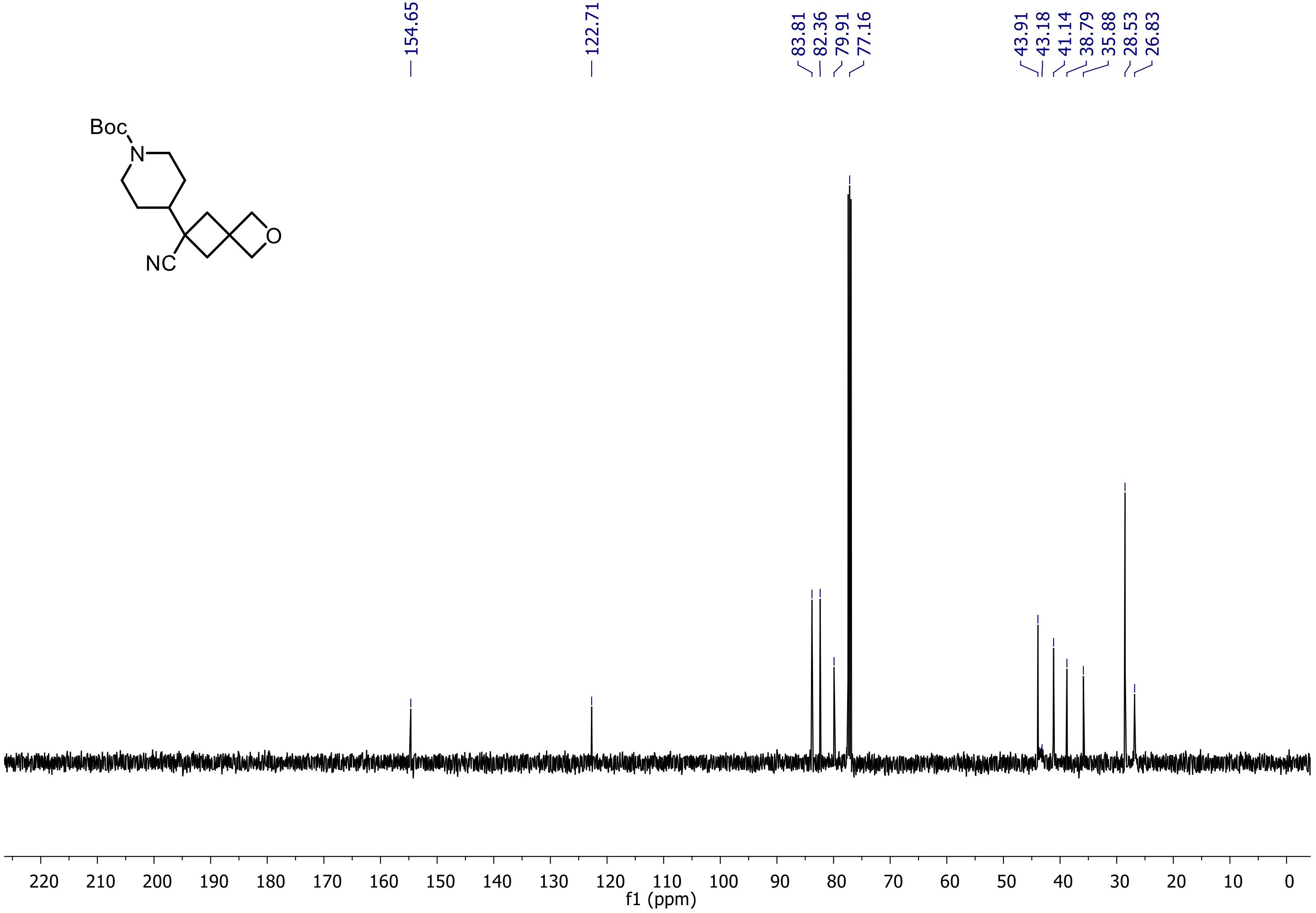

Compound 1a

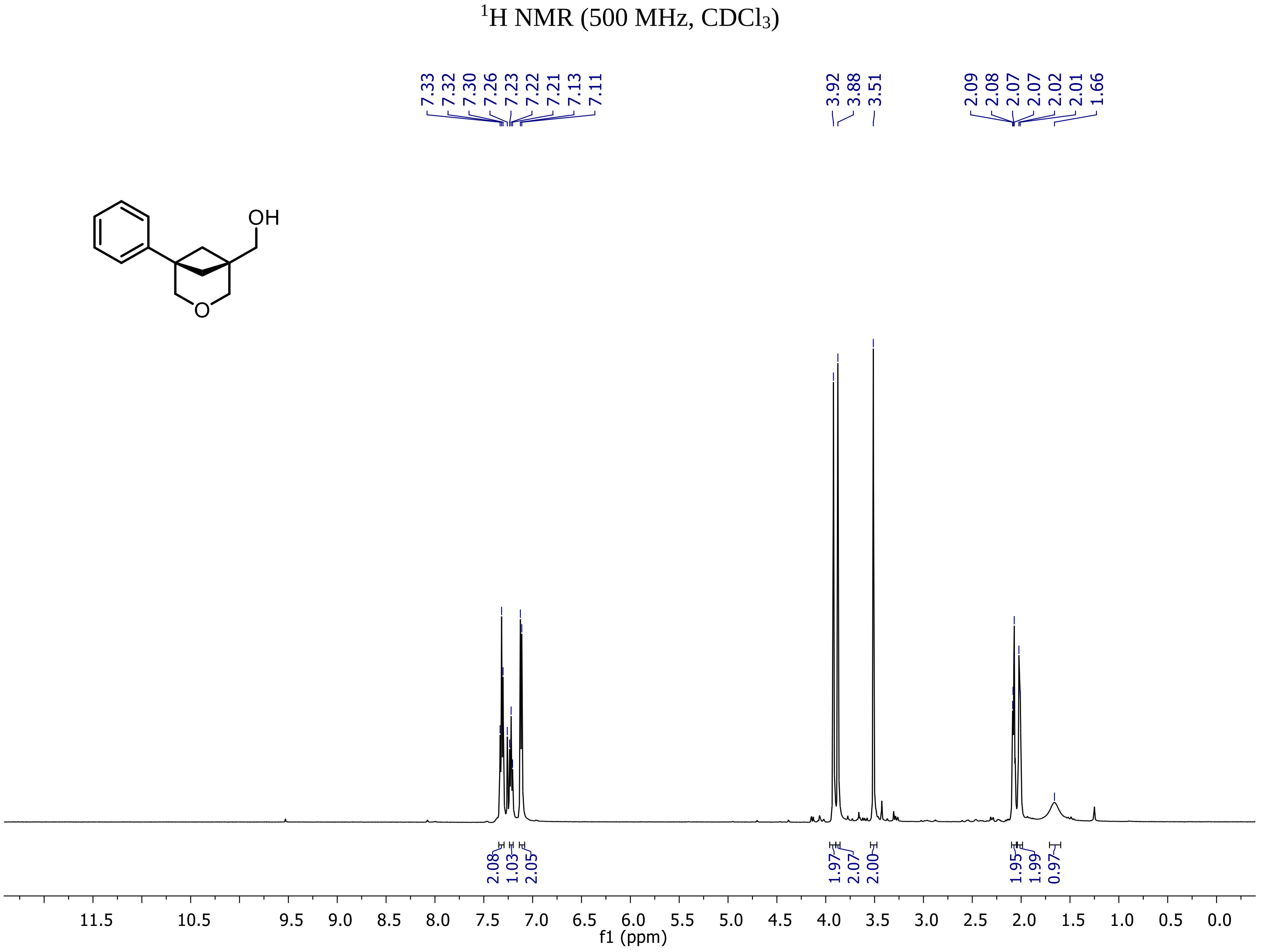

^{1}H NMR (500 MHz, CDCl$_3$)
7.33 7.32 7.30 7.26 7.23 7.22 7.21 7.13 7.11
3.92 3.88 3.51
2.09 2.08 2.07 2.07 2.02 2.01 1.66
OH
O
2.08 1.03 2.05
1.97 2.07 2.00
1.95 1.99 0.97
11.5 10.5 9.5 9.0 8.5 8.0 7.5 7.0 6.5 6.0 5.5 5.0 4.5 4.0 3.5 3.0 2.5 2.0 1.5 1.0 0.5 0.0
f1 (ppm)

$^{13}C\{^{1}H\}$ NMR (126 MHz, $CDCl_3$)

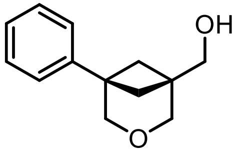

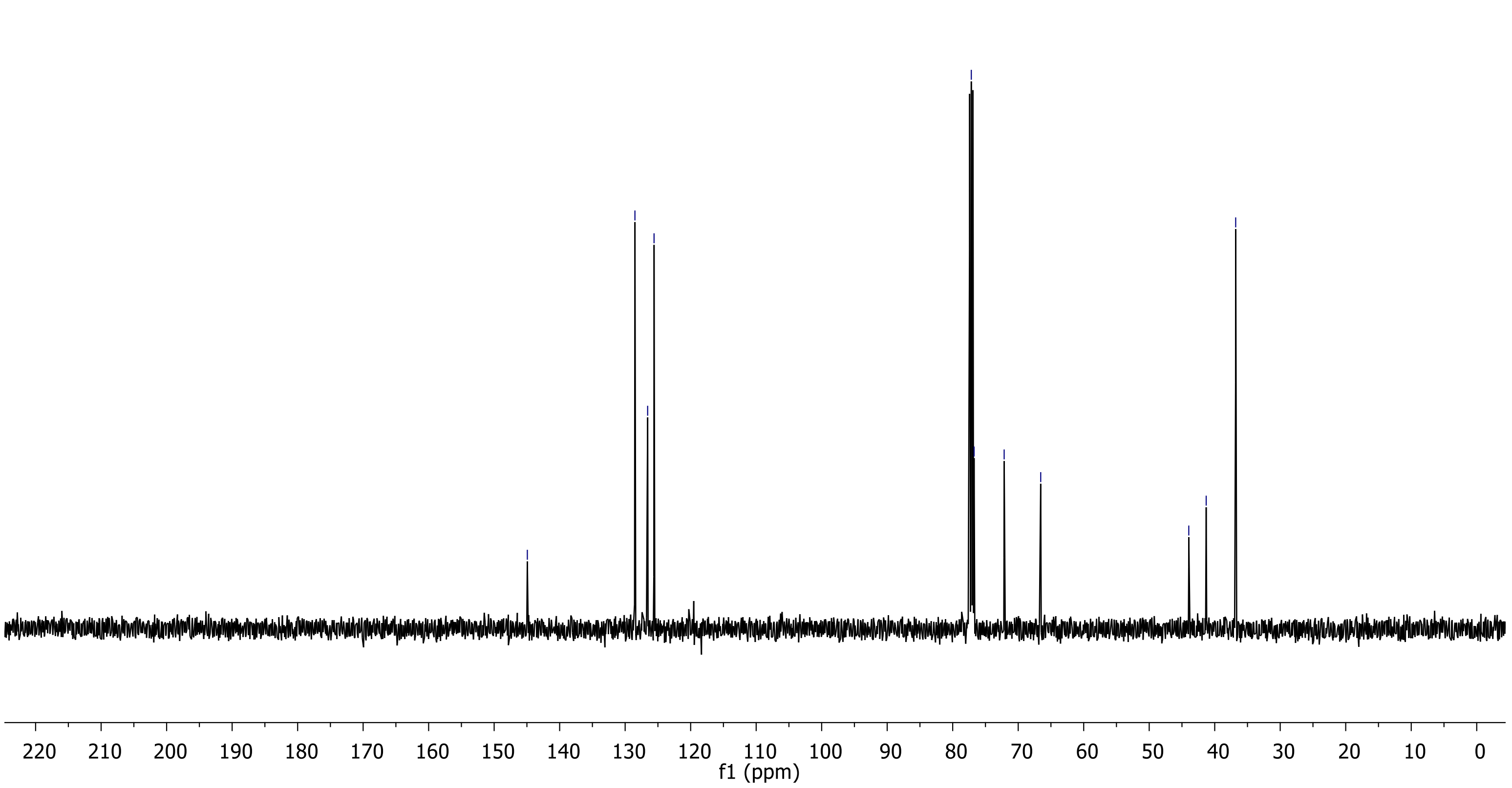

Compound 5a

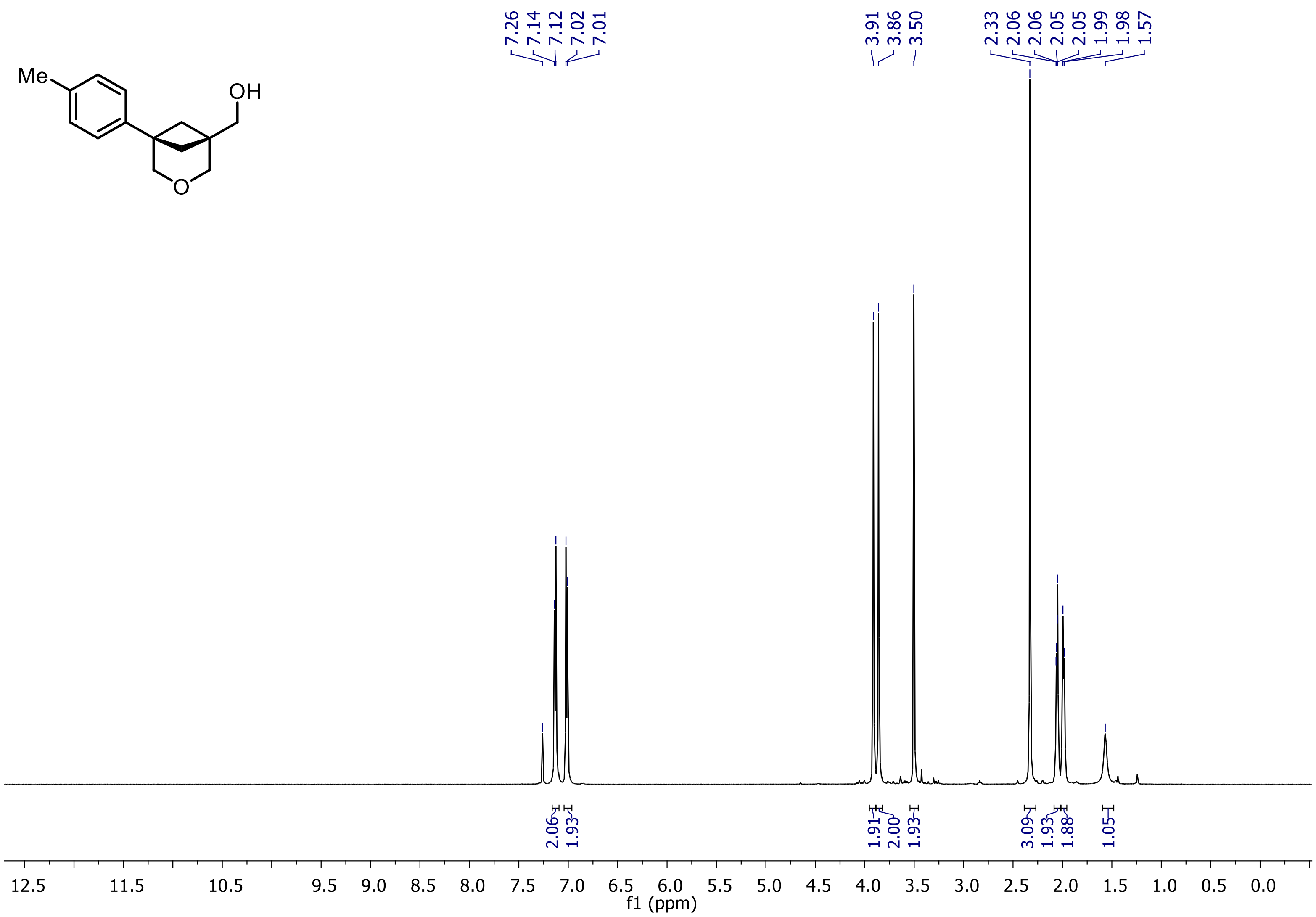

^{1}H NMR (500 MHz, CDCl$_3$)
7.26
7.14
7.12
7.02
7.01
3.91
3.86
3.50
2.33
2.06
2.06
2.05
2.05
1.99
1.98
1.57
2.06
1.93
1.91
2.00
1.93
3.09
1.93
1.88
1.05
12.5
11.5
10.5
9.5
9.0
8.5
8.0
7.5
7.0
6.5
6.0
5.5
5.0
4.5
4.0
3.5
3.0
2.5
2.0
1.5
1.0
0.5
0.0
f1 (ppm)

$^{13}C\{^{1}H\}$ NMR (126 MHz, $CDCl_3$)

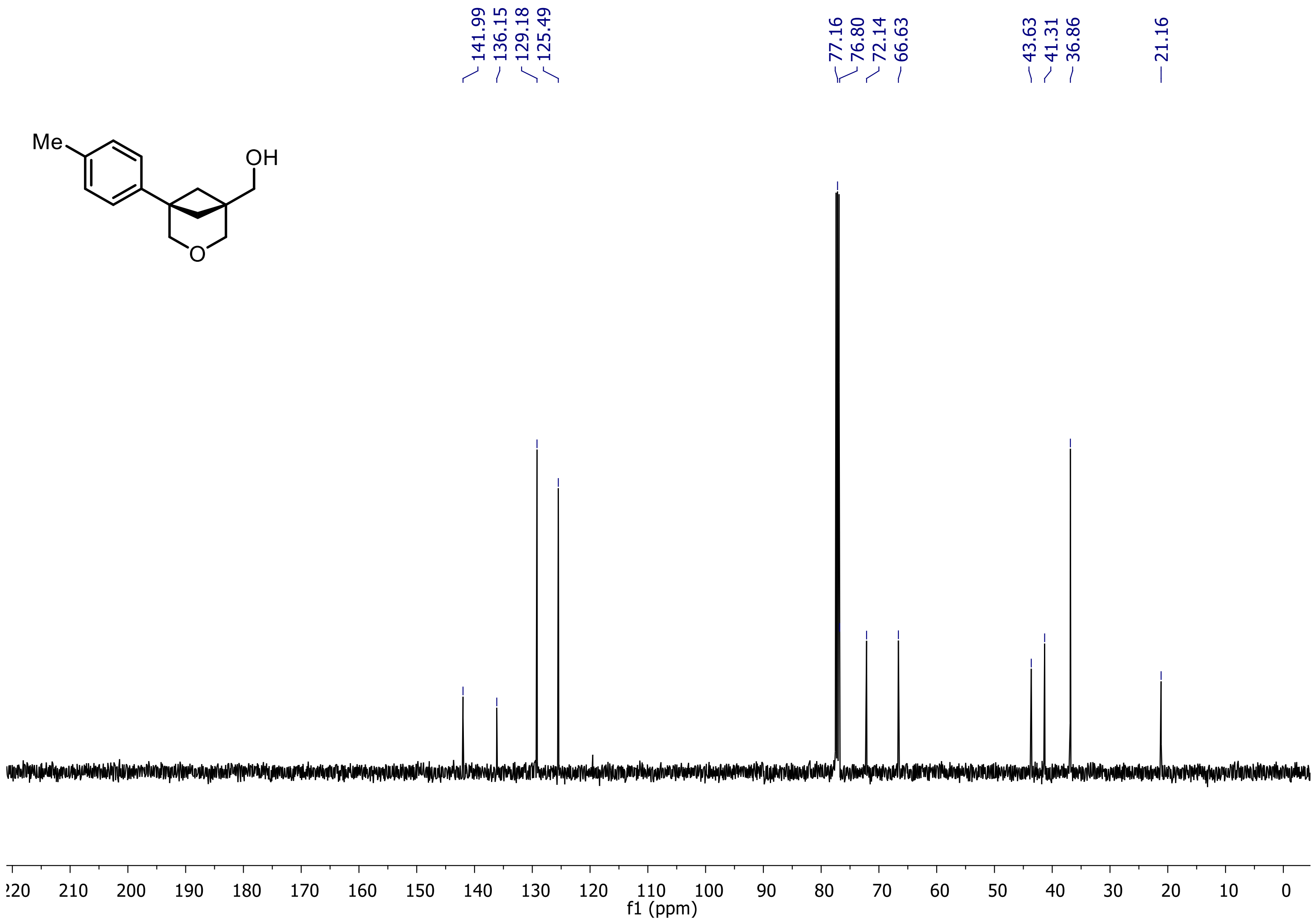

Compound 6a

^{1}H NMR (500 MHz, DMSO-d_6)

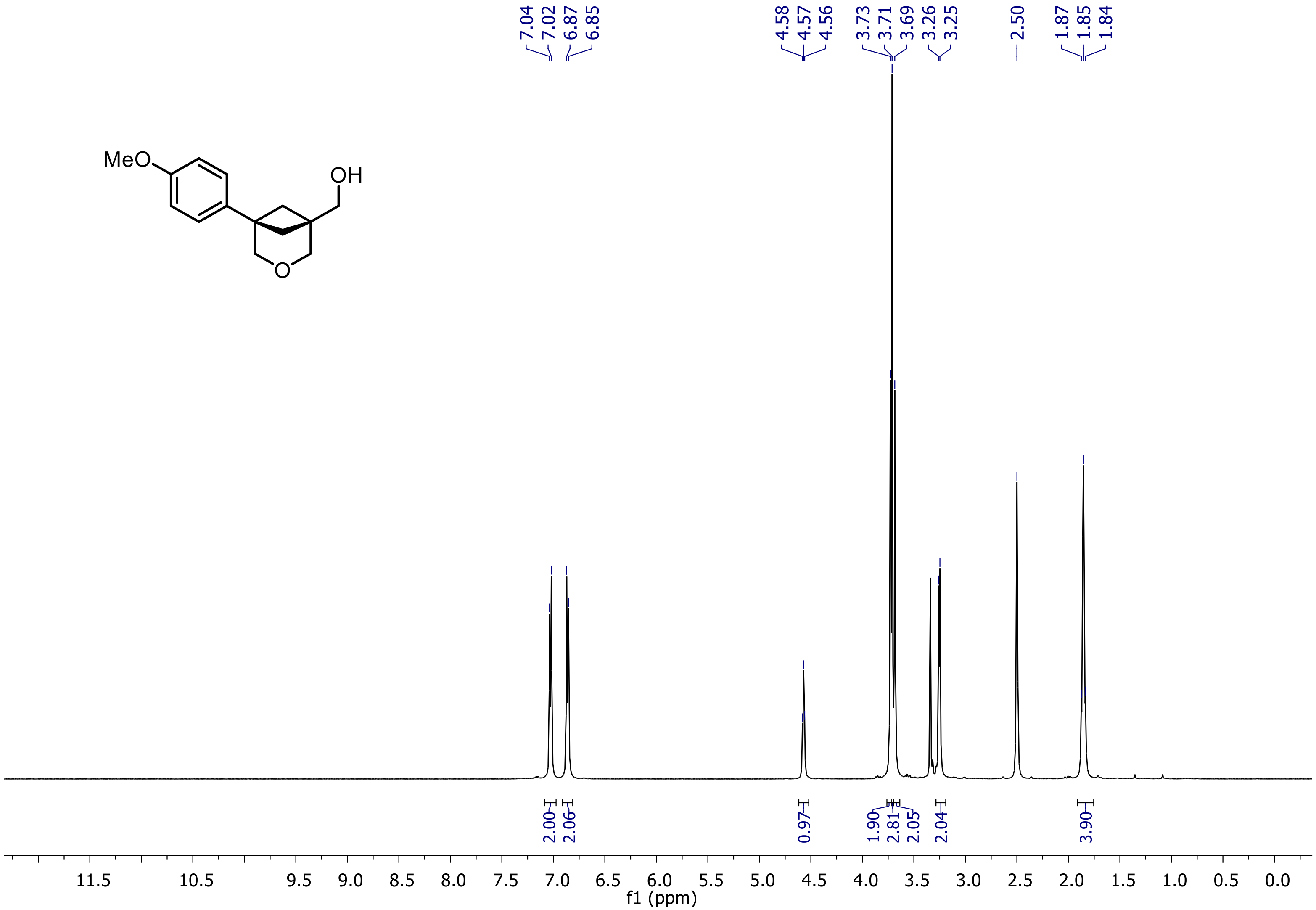

$^{13}C\{^{1}H\}$ NMR (126 MHz, DMSO-d_6)

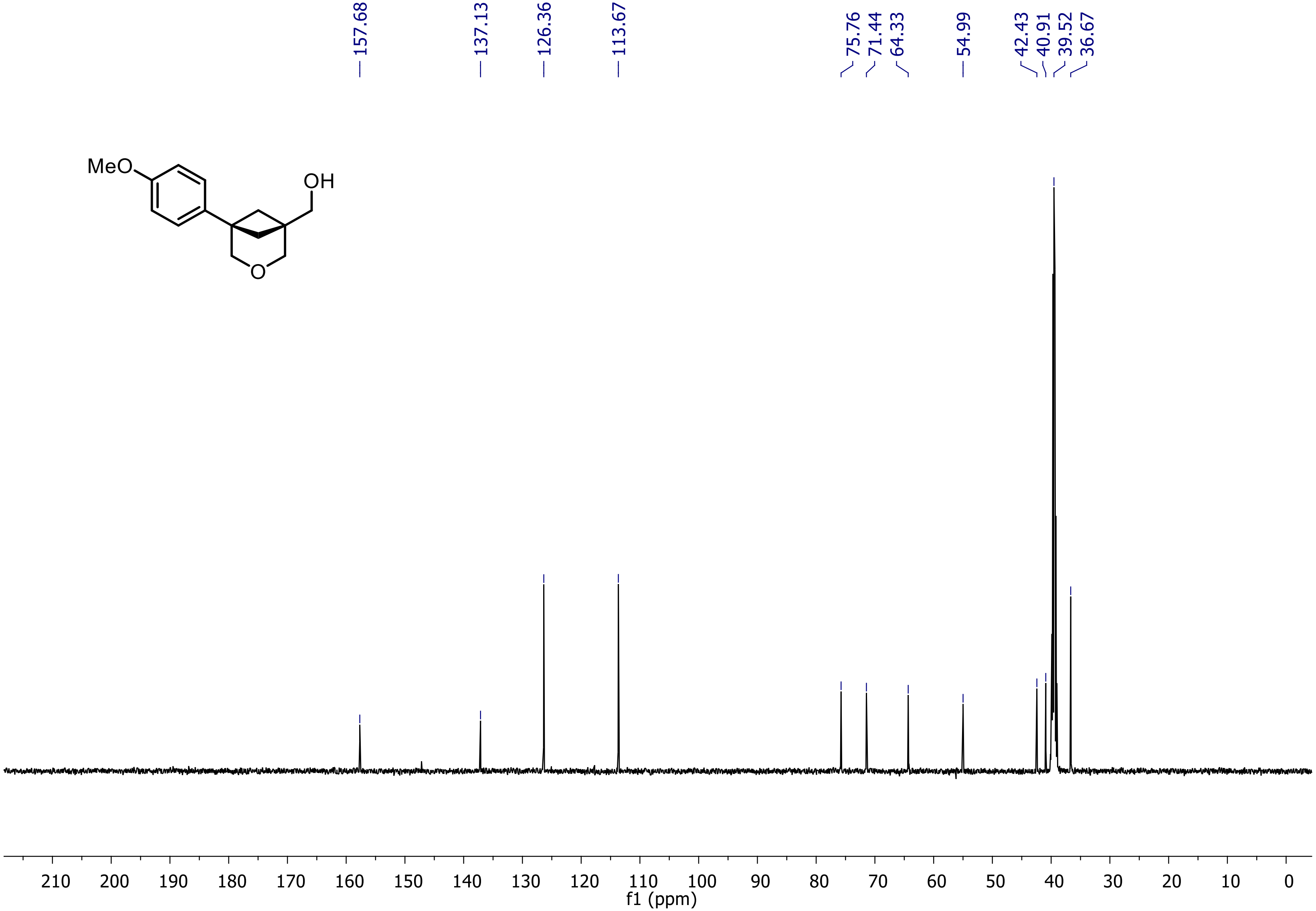

Compound 7a

^{1}H NMR (500 MHz, $CDCl_3$)

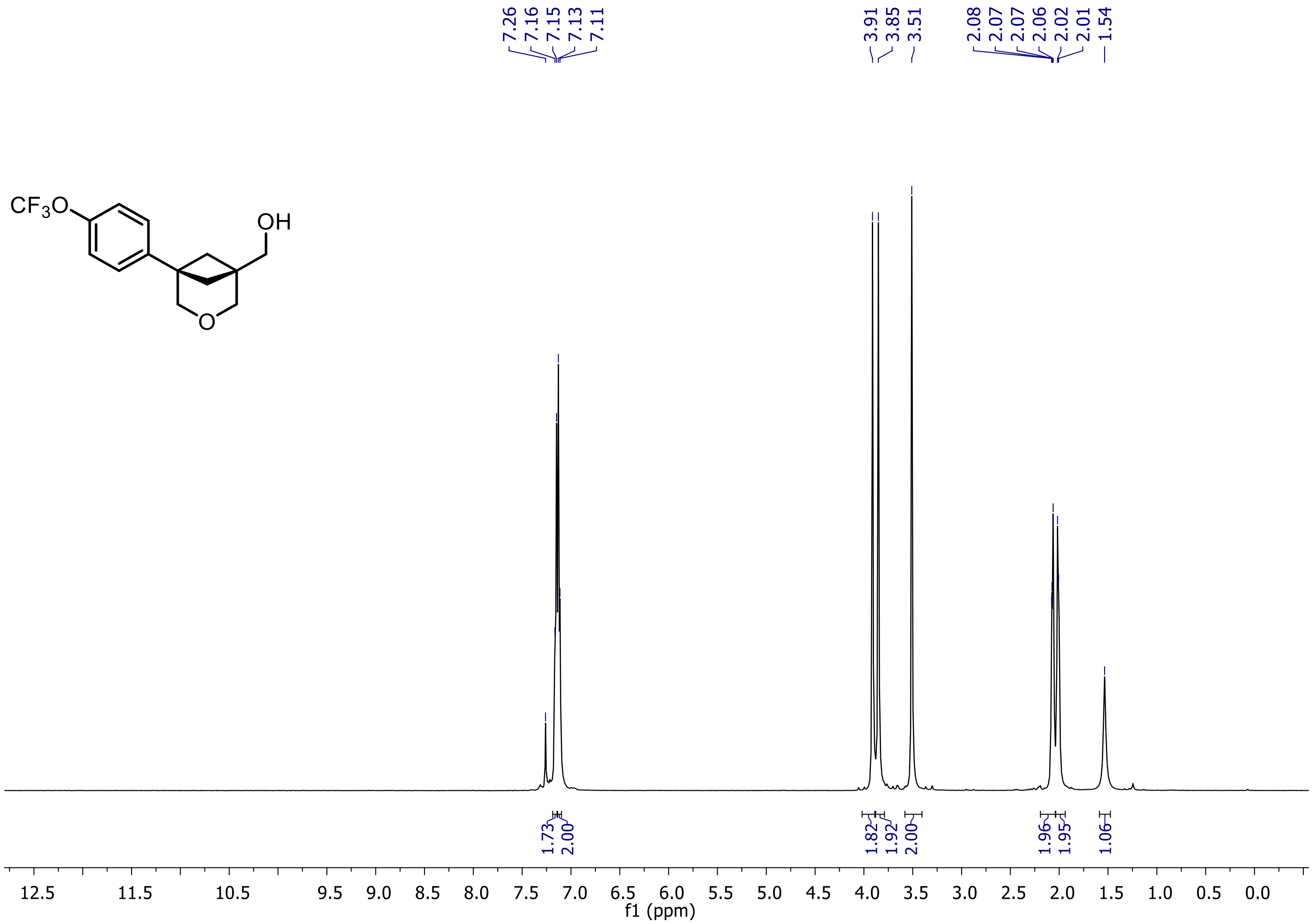

$^{13}C\{^{1}H\}$ NMR (151 MHz, $CDCl_3$)

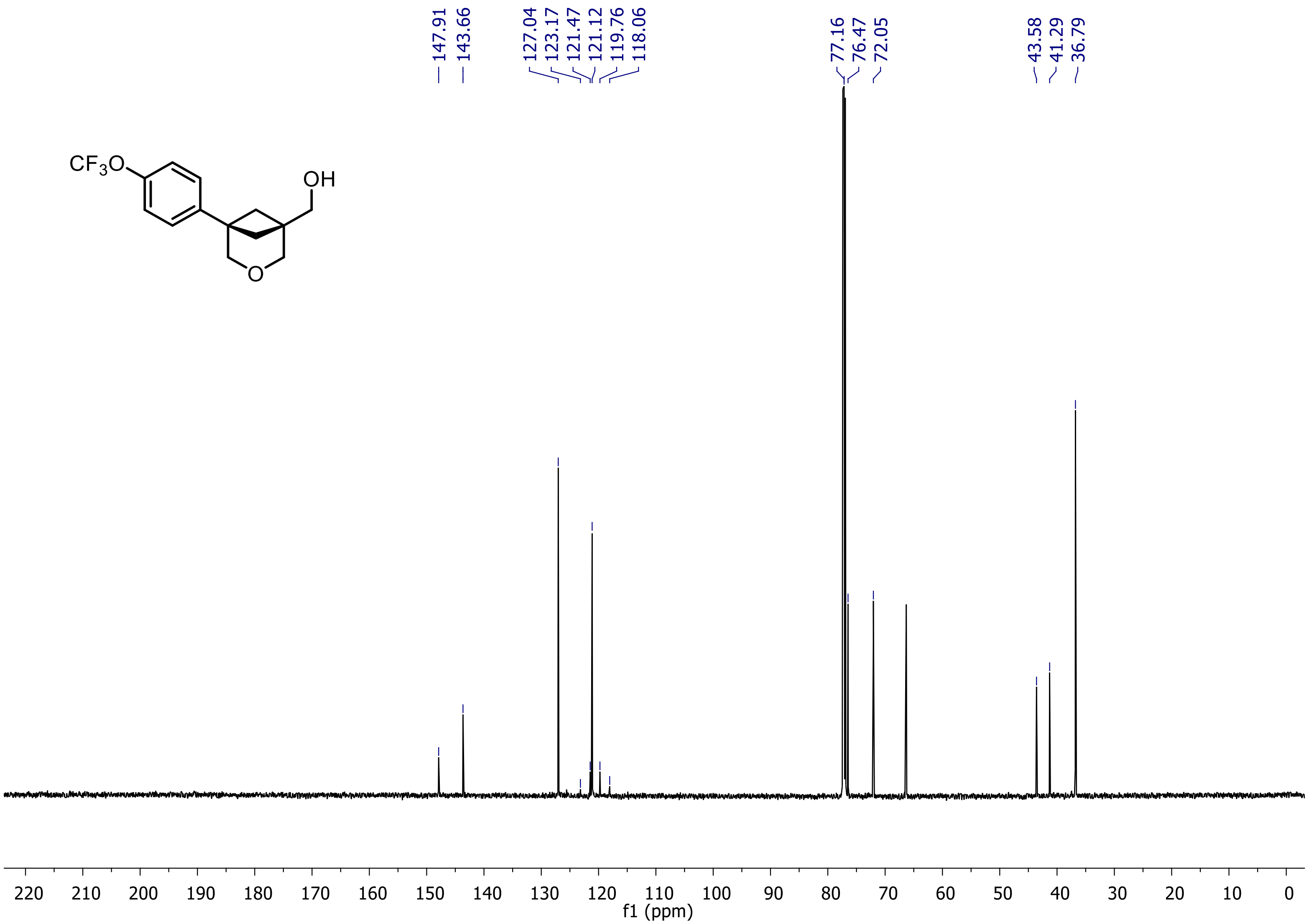

$^{19}F\{^{1}H\}$ NMR (376 MHz, $CDCl_3$)

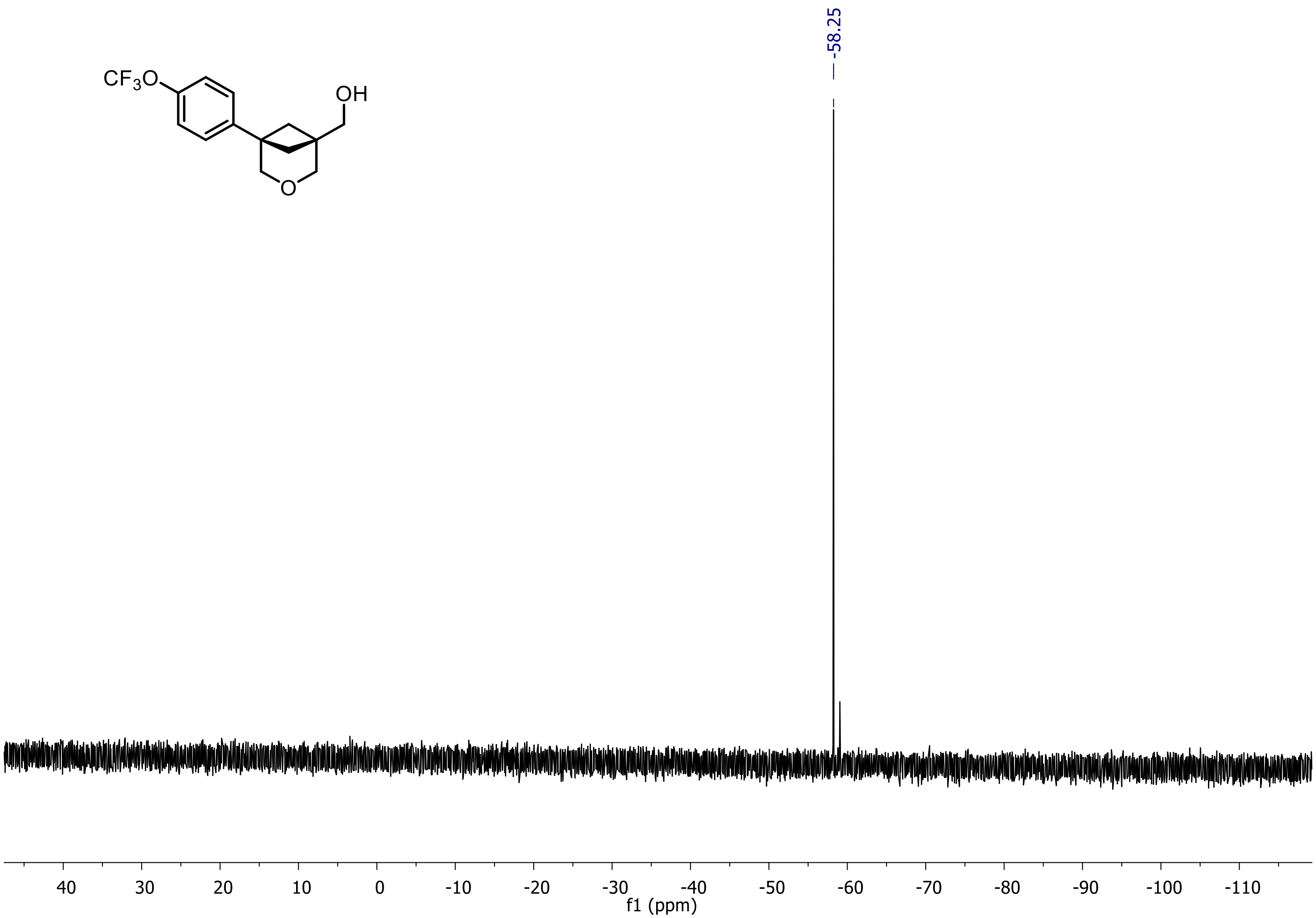

Compound 8a

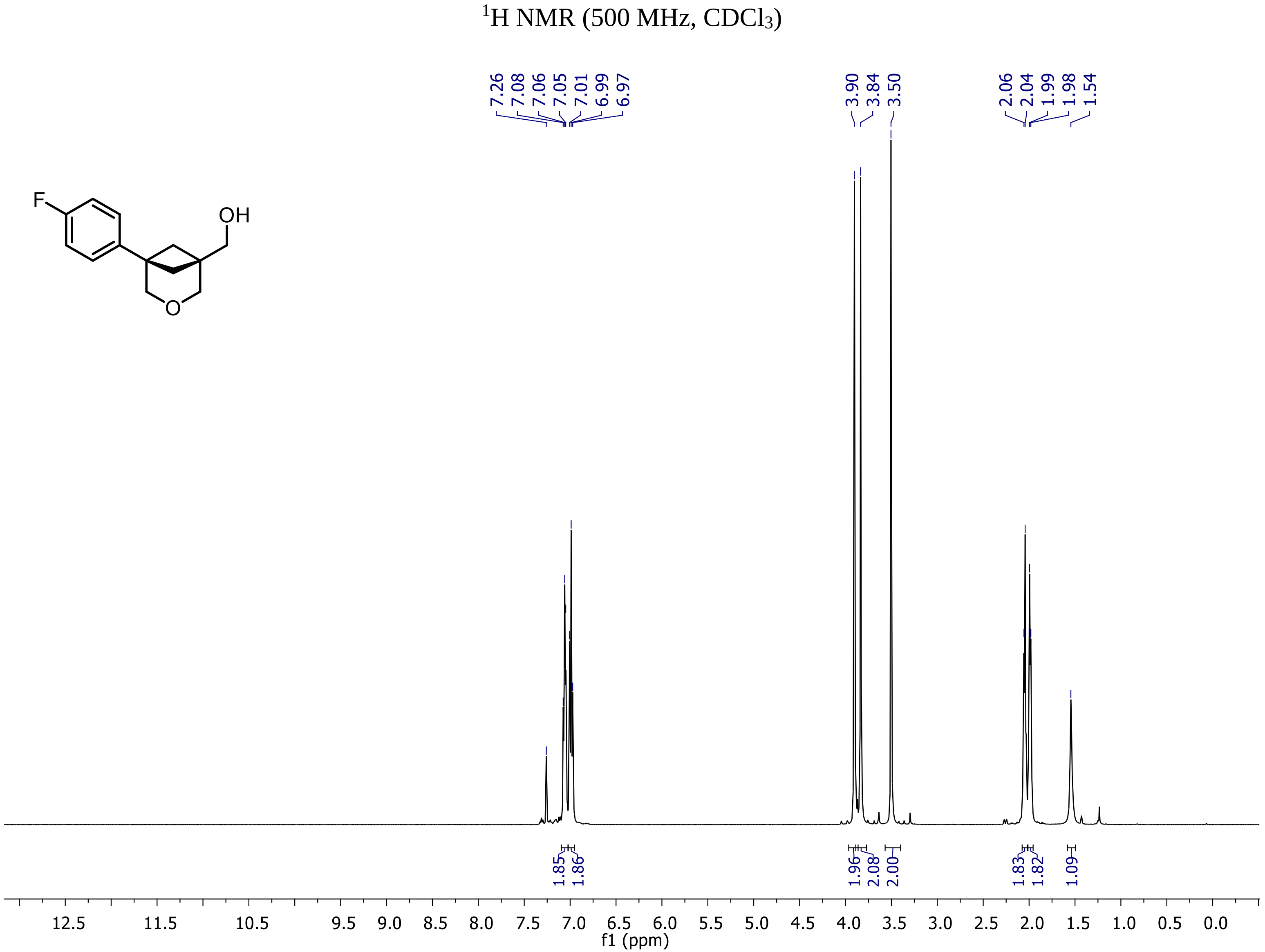
^{1}H NMR (500 MHz, CDCl$_3$)
F
OH
O
7.26
7.08
7.06
7.05
7.01
6.99
6.97
3.90
3.84
3.50
2.06
2.04
1.99
1.98
1.54
1.85
1.86
1.96
2.08
2.00
1.83
1.82
1.09
12.5
11.5
10.5
9.5
9.0
8.5
8.0
7.5
7.0
6.5
6.0
5.5
5.0
4.5
4.0
3.5
3.0
2.5
2.0
1.5
1.0
0.5
0.0
f1 (ppm)

$^{13}C\{^{1}H\}$ NMR (126 MHz, $CDCl_3$)

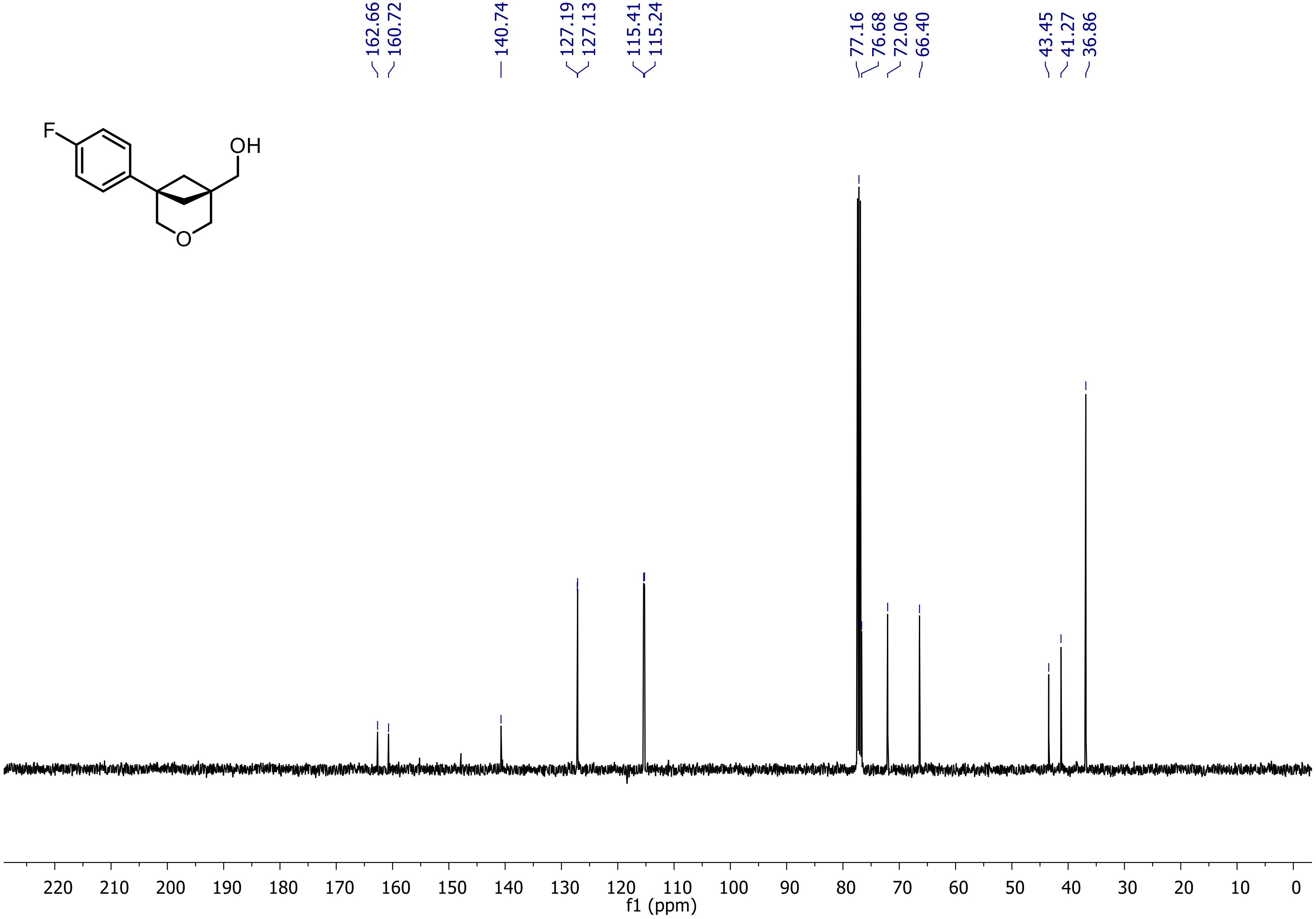

$^{19}F\{^{1}H\}$ NMR (376 MHz, $CDCl_3$)

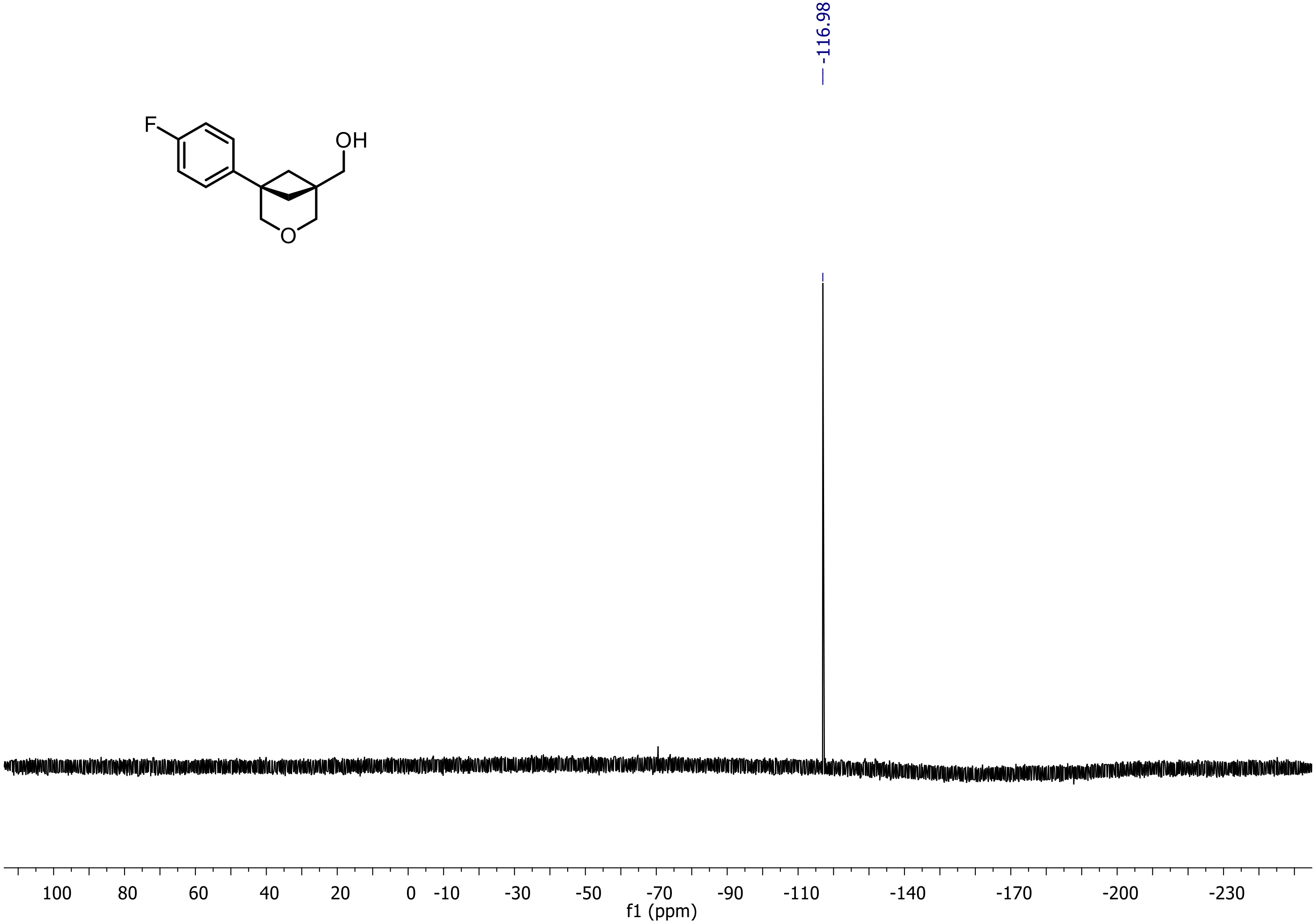

Compound 9a

^{1}H NMR (500 MHz, $CDCl_3$)

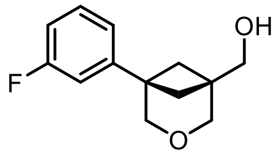

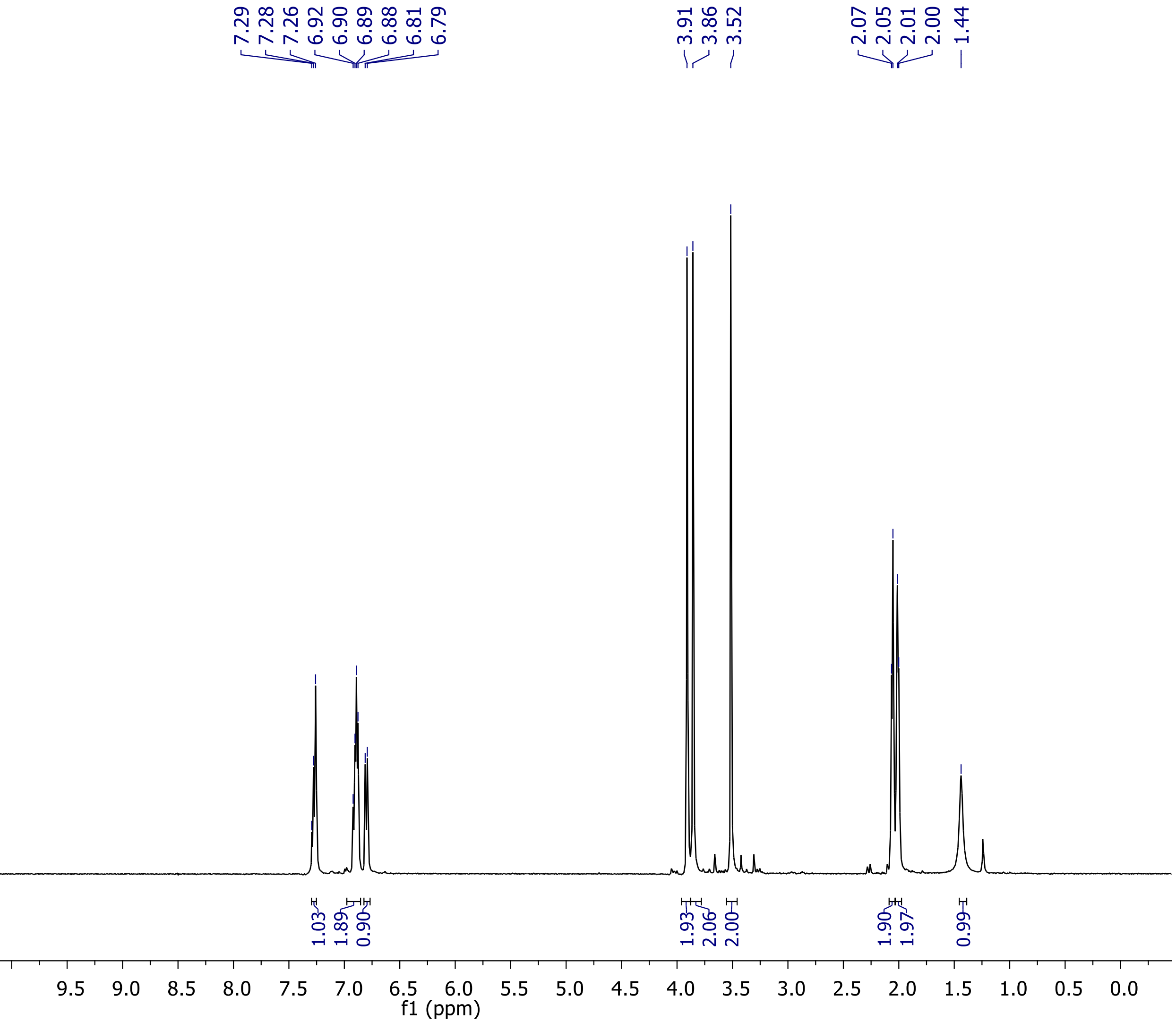

$^{13}C\{^{1}H\}$ NMR (151 MHz, $CDCl_3$)

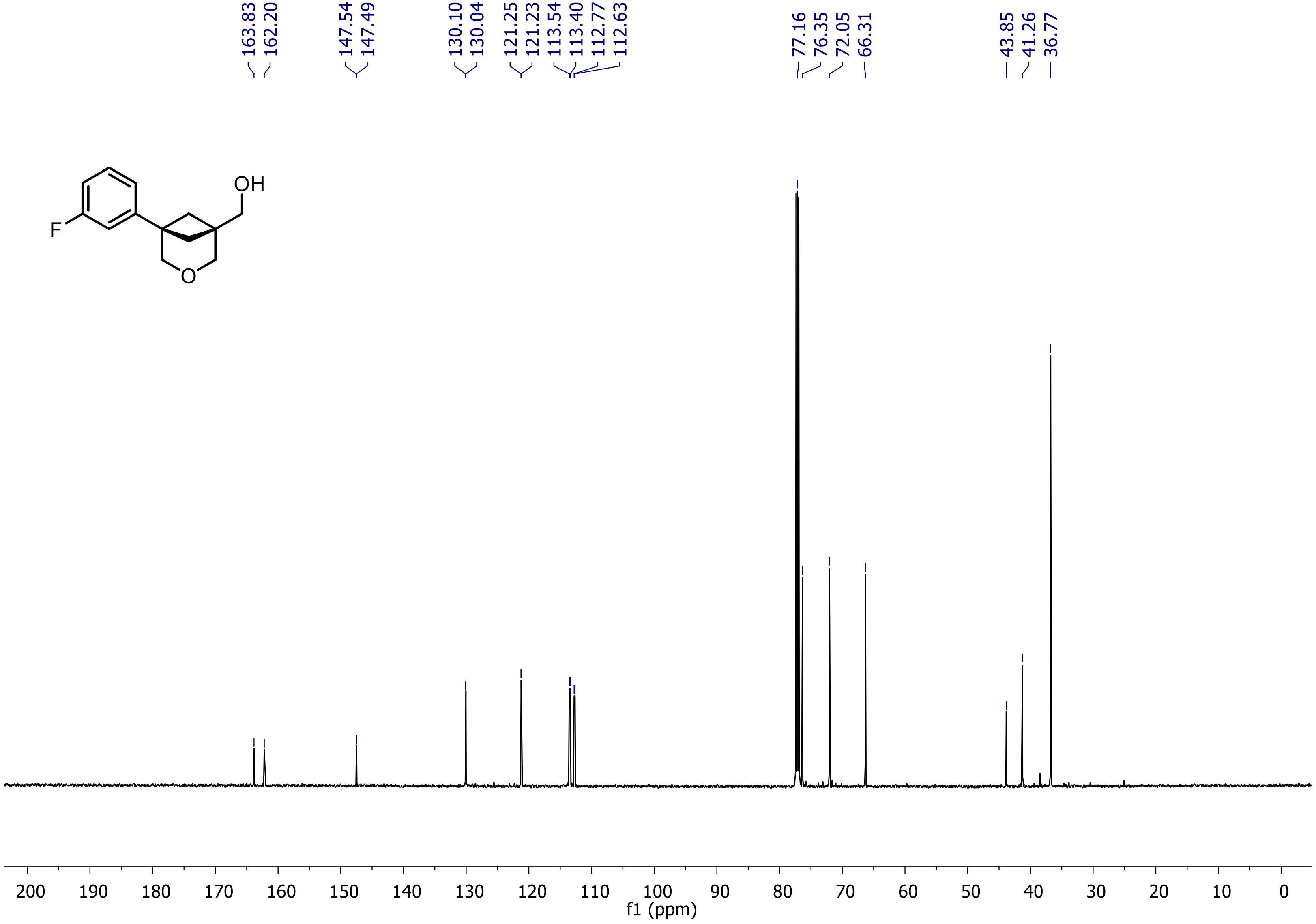

$^{19}F\{^1H\}$ NMR (376 MHz, $CDCl_3$)

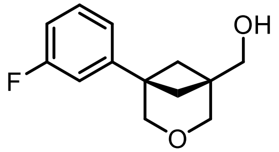

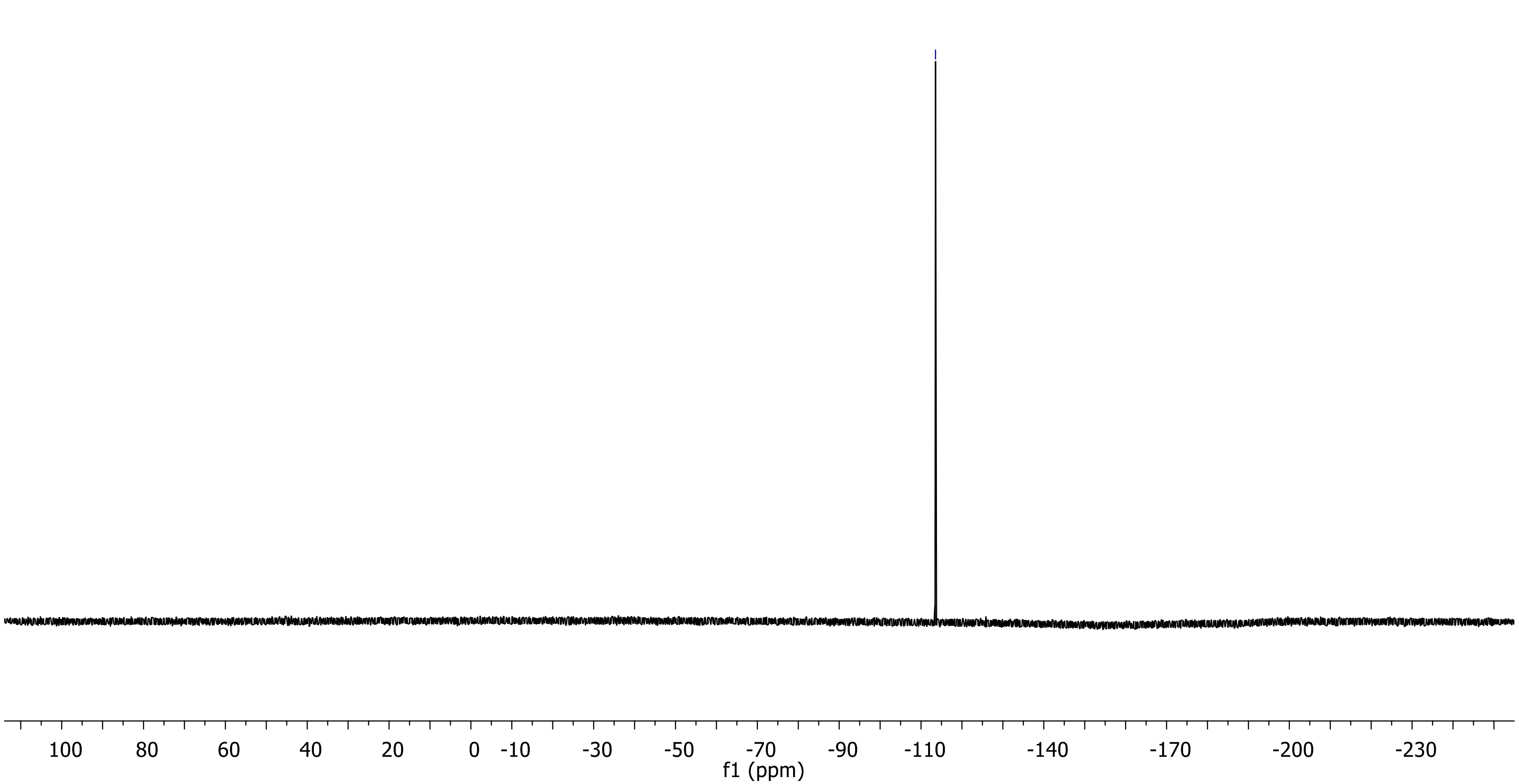

Compound 10a

^{1}H NMR (500 MHz, $CDCl_3$)

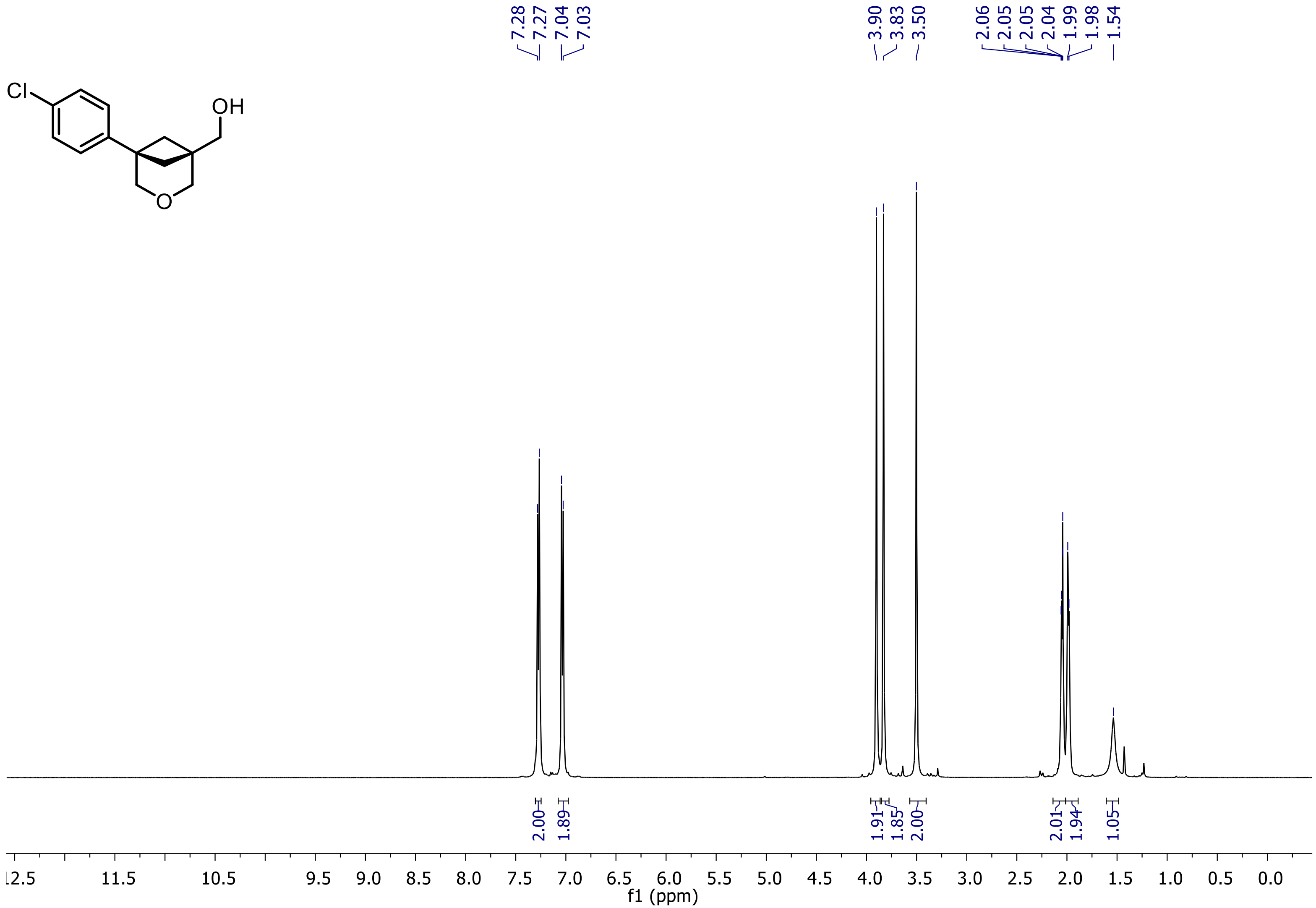

$^{13}C\{^{1}H\}$ NMR (126 MHz, $CDCl_3$)

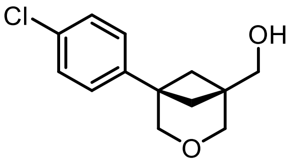

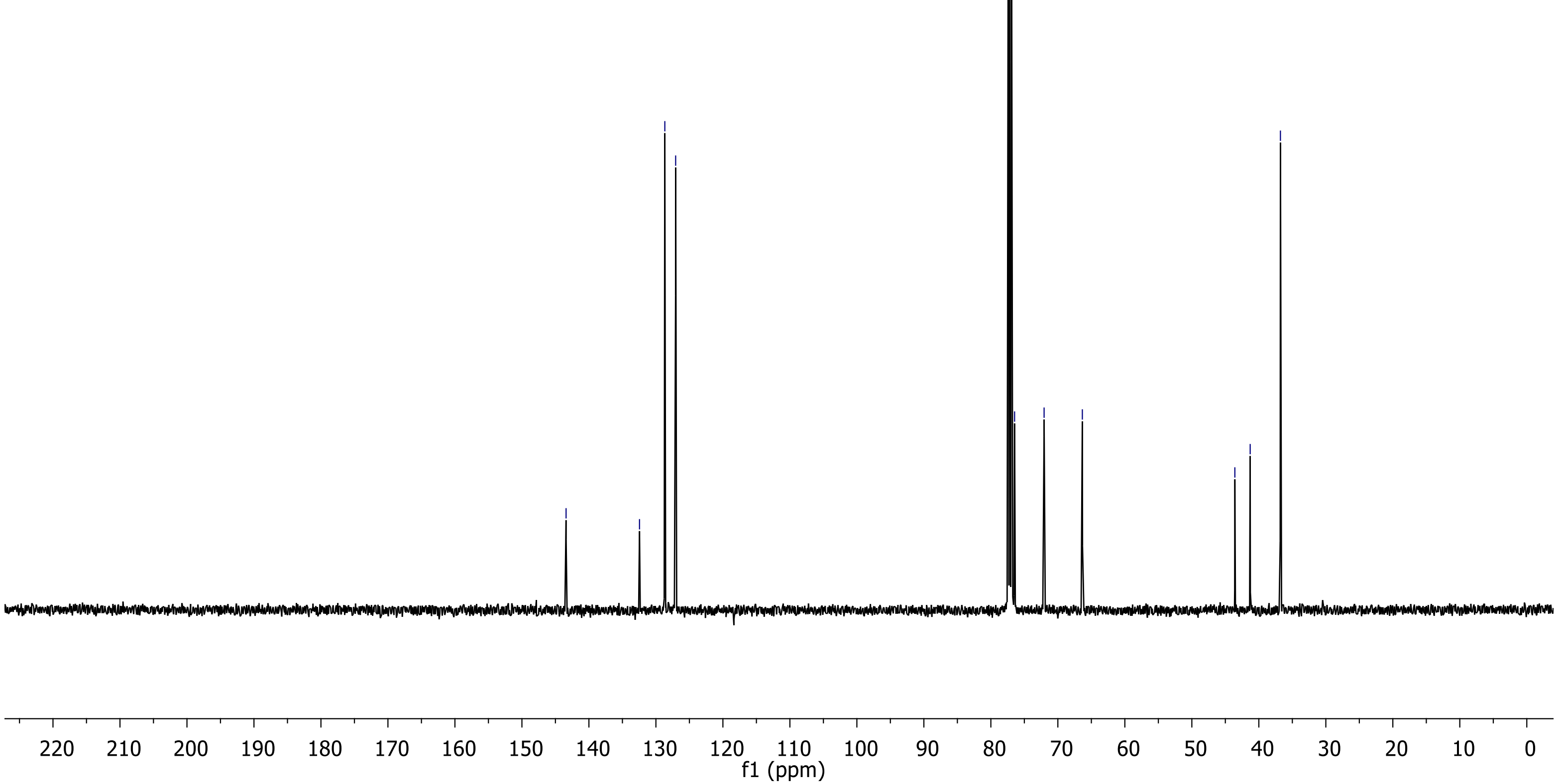

Compound 11a

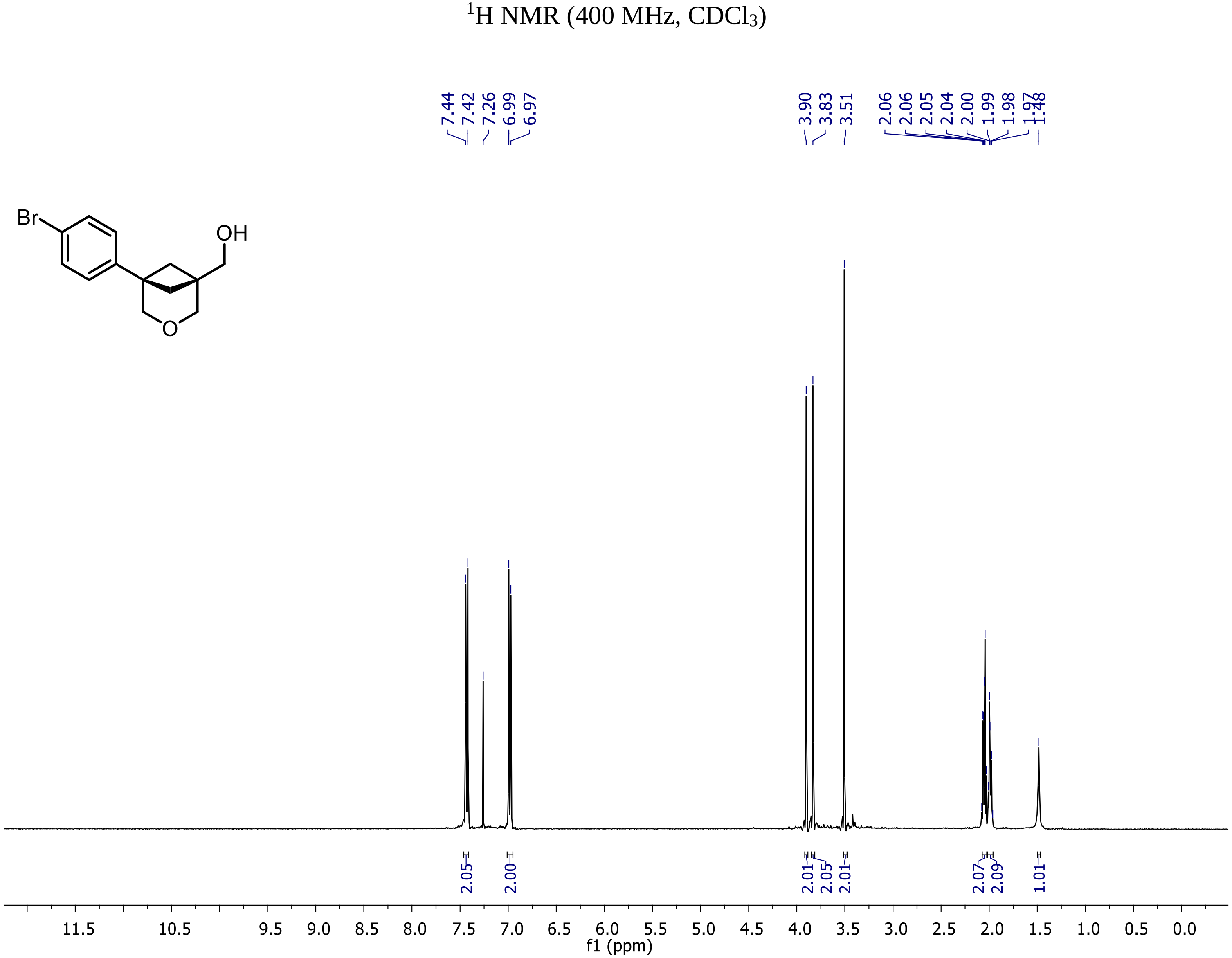

$^{13}C\{^{1}H\}$ NMR (101 MHz, $CDCl_3$)

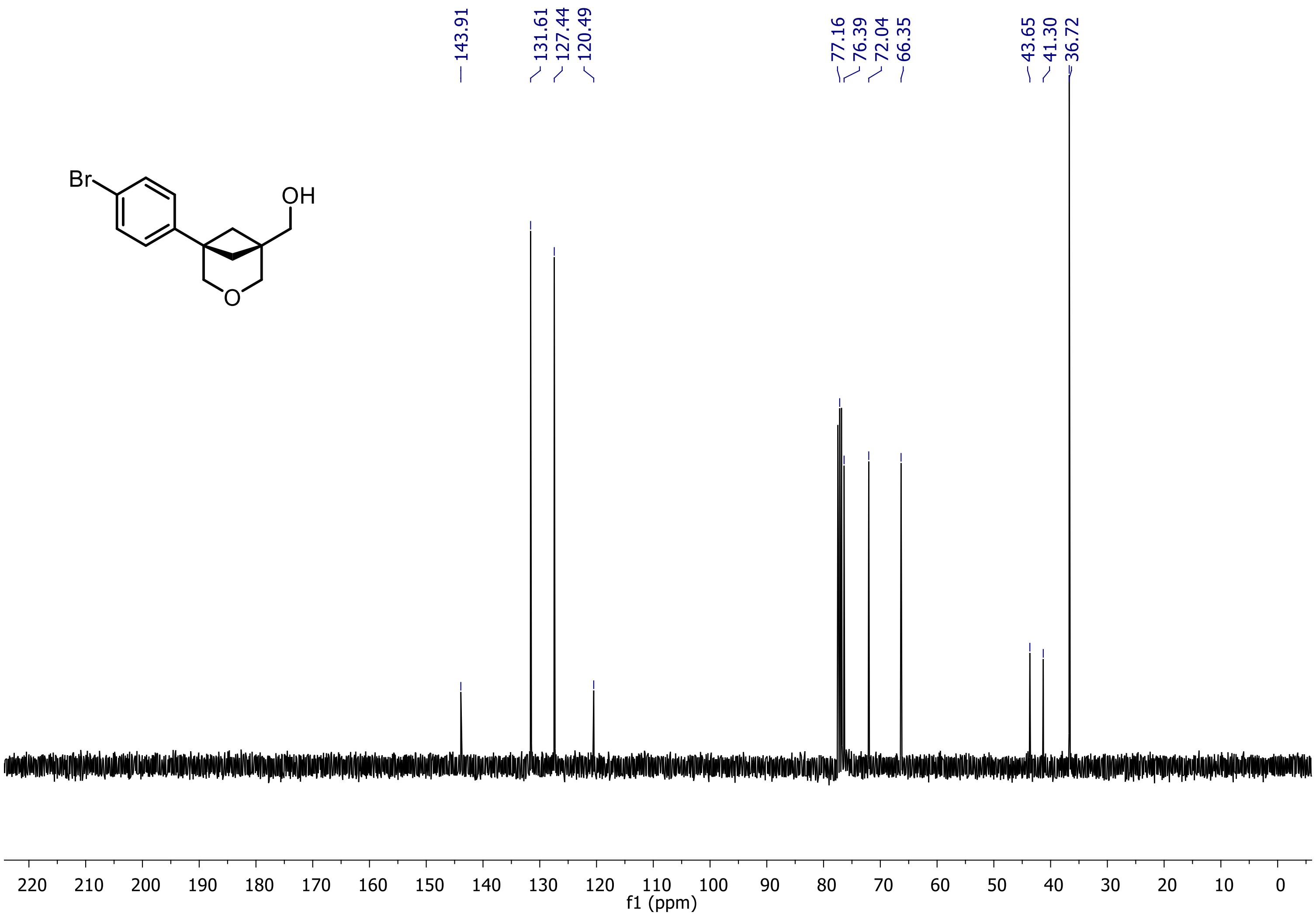

Compound 12a

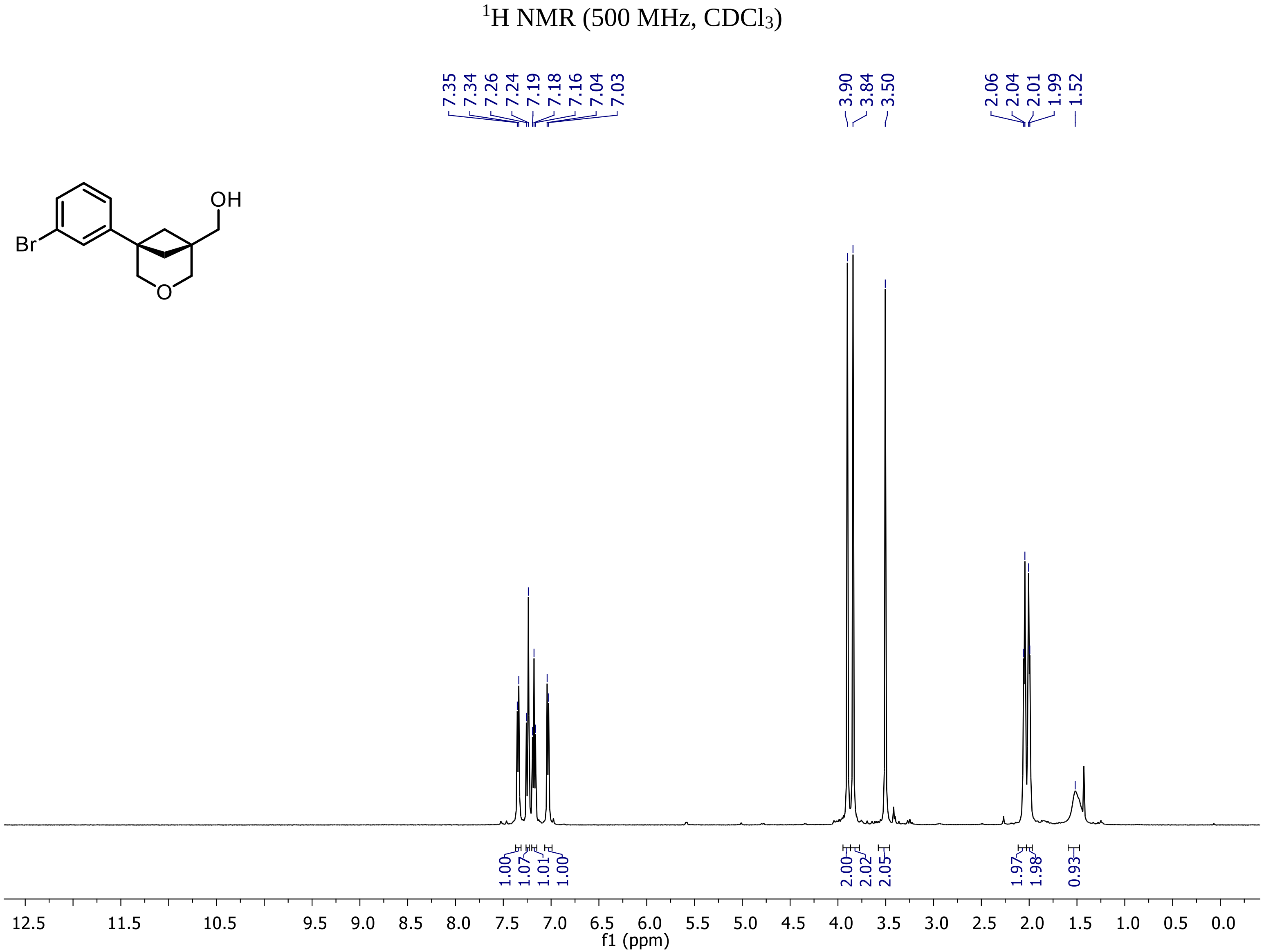

$^{13}C\{^{1}H\}$ NMR (126 MHz, $CDCl_3$)

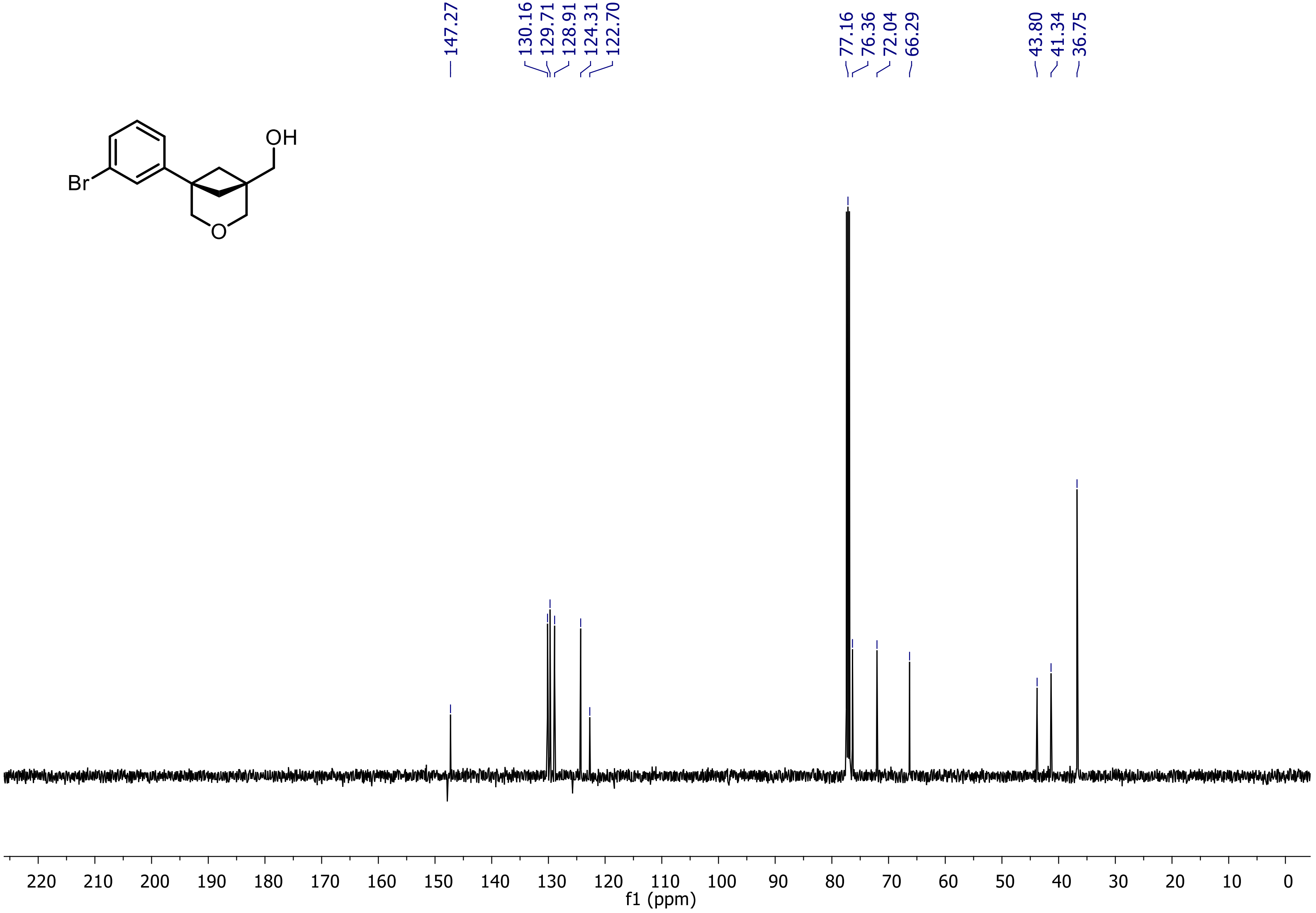

Compound 13a

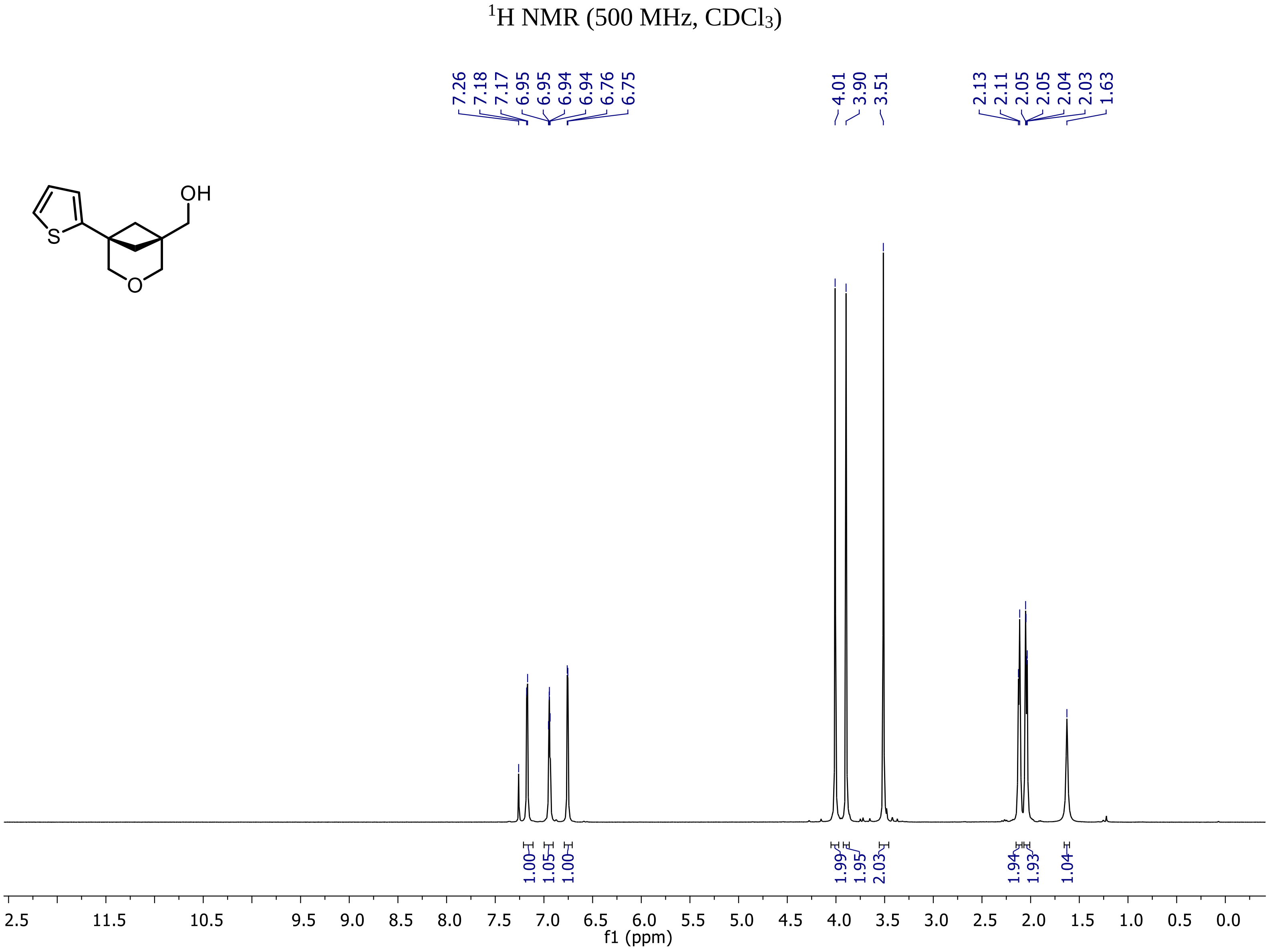

$^{13}C\{^1H\}$ NMR (126 MHz, $CDCl_3$)

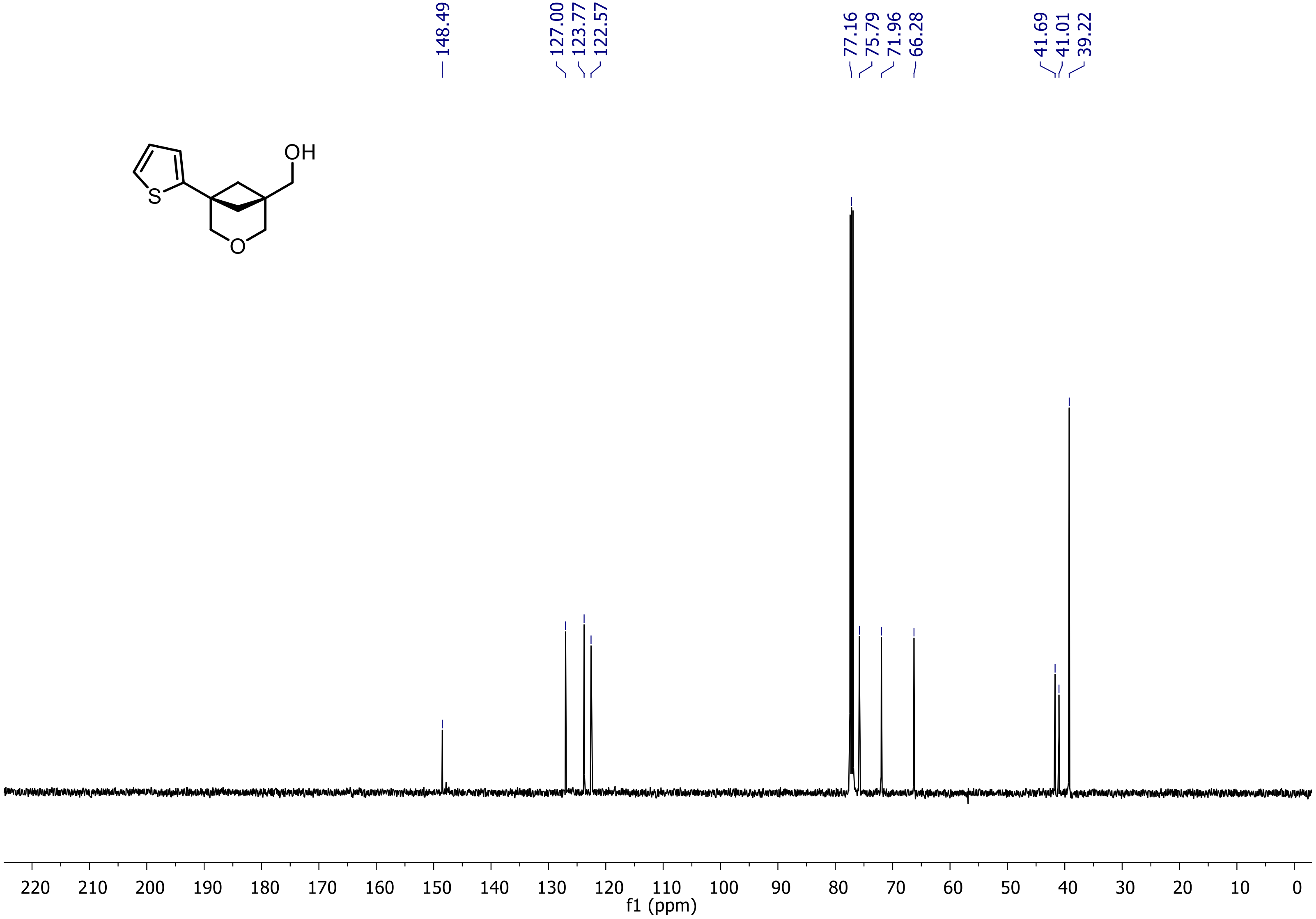

Compound 14a

^{1}H NMR (500 MHz, DMSO-d_6)

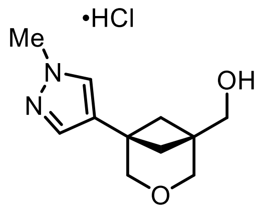

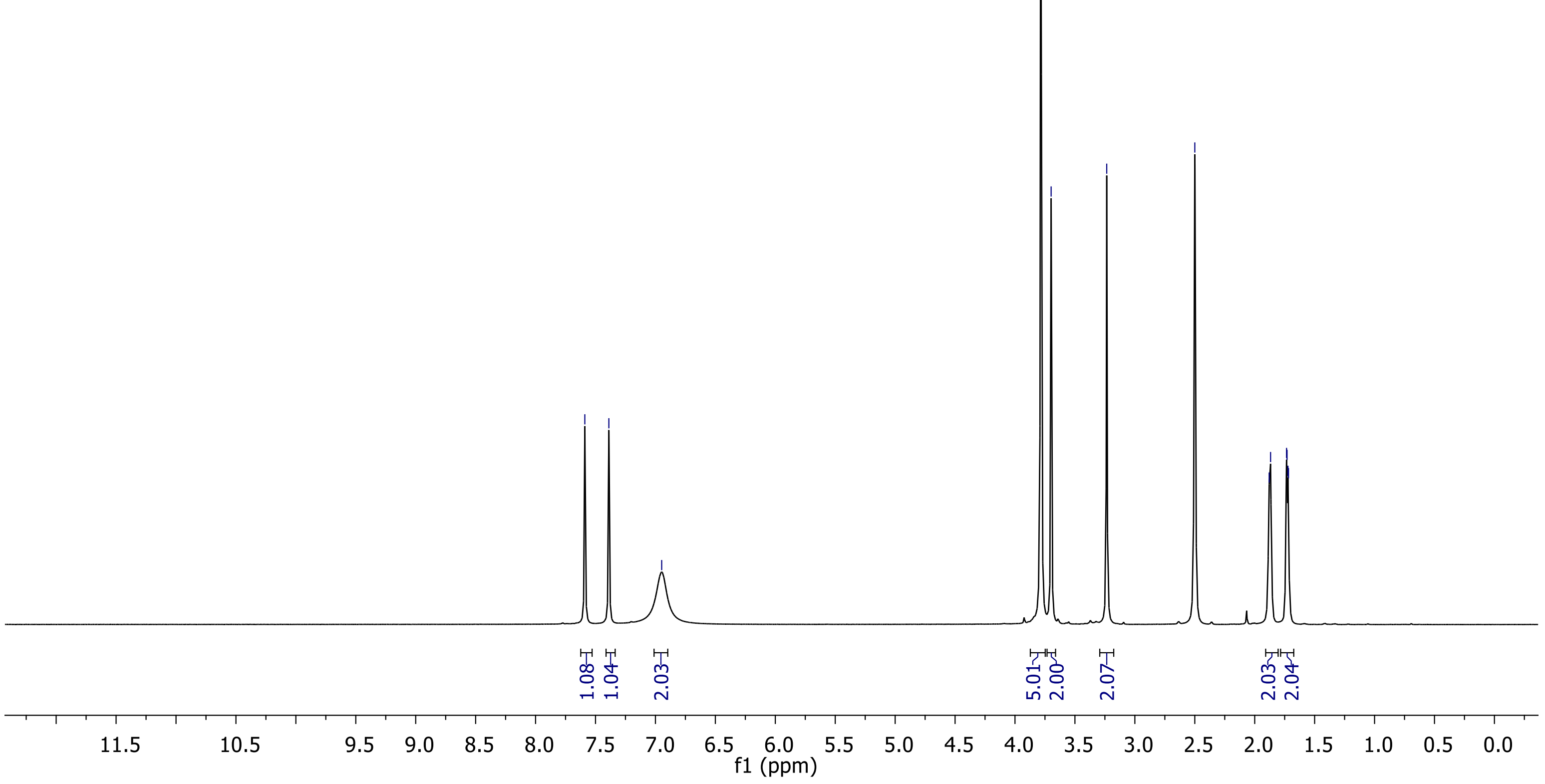

$^{13}C\{^{1}H\}$ NMR (126 MHz, DMSO-d_6)

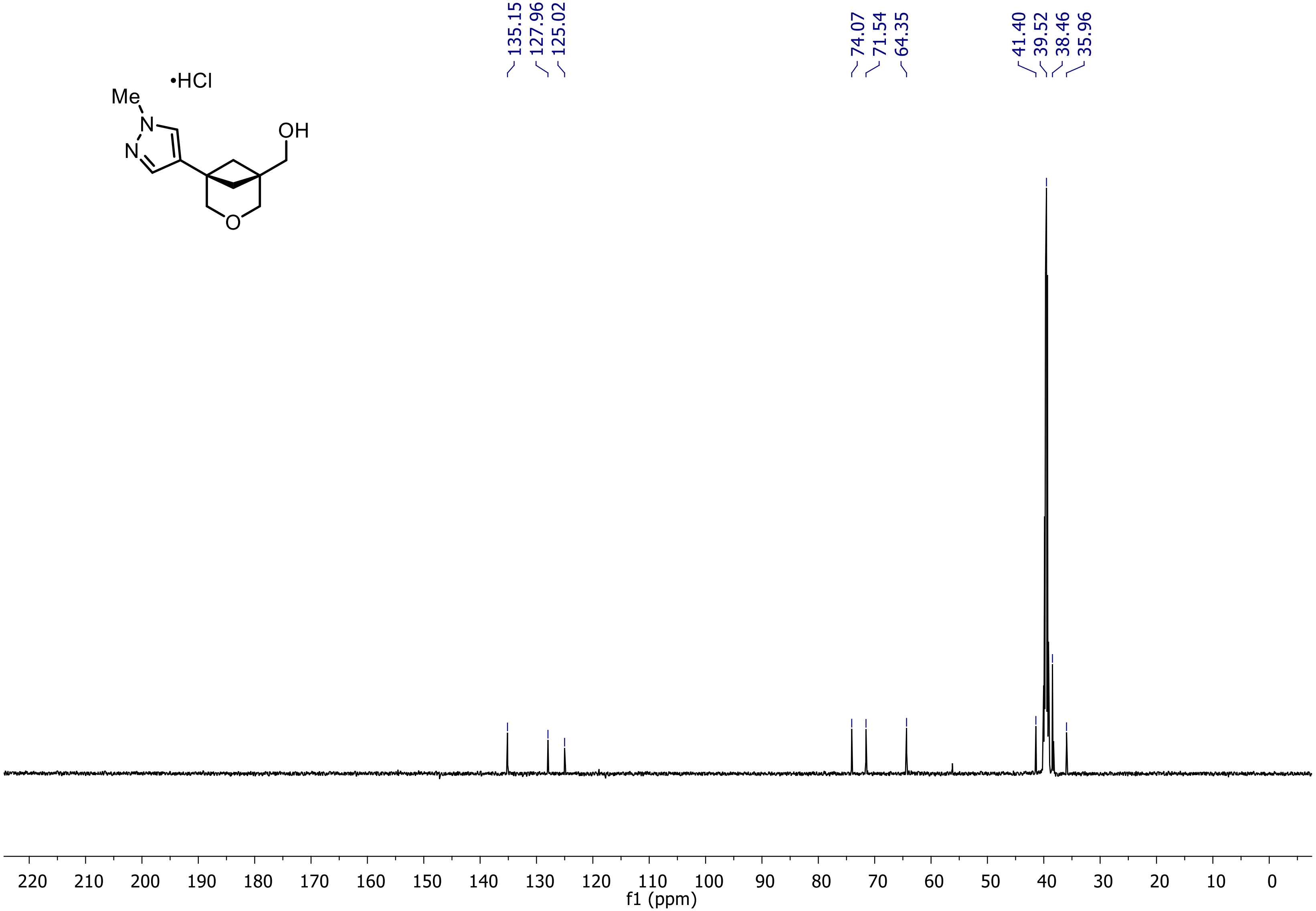

Compound 15a

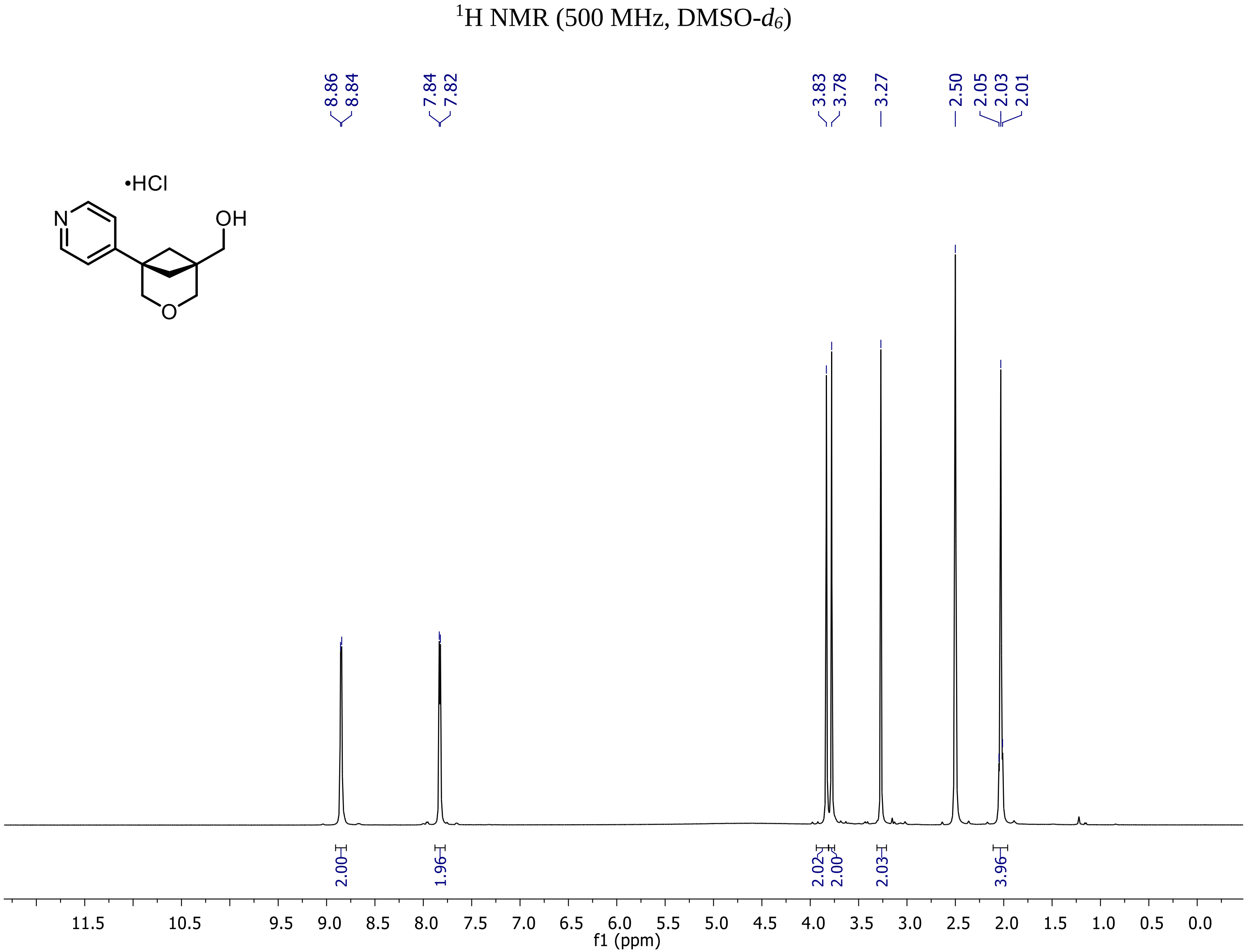

$^{13}C\{^{1}H\}$ NMR (126 MHz, DMSO-d_6)

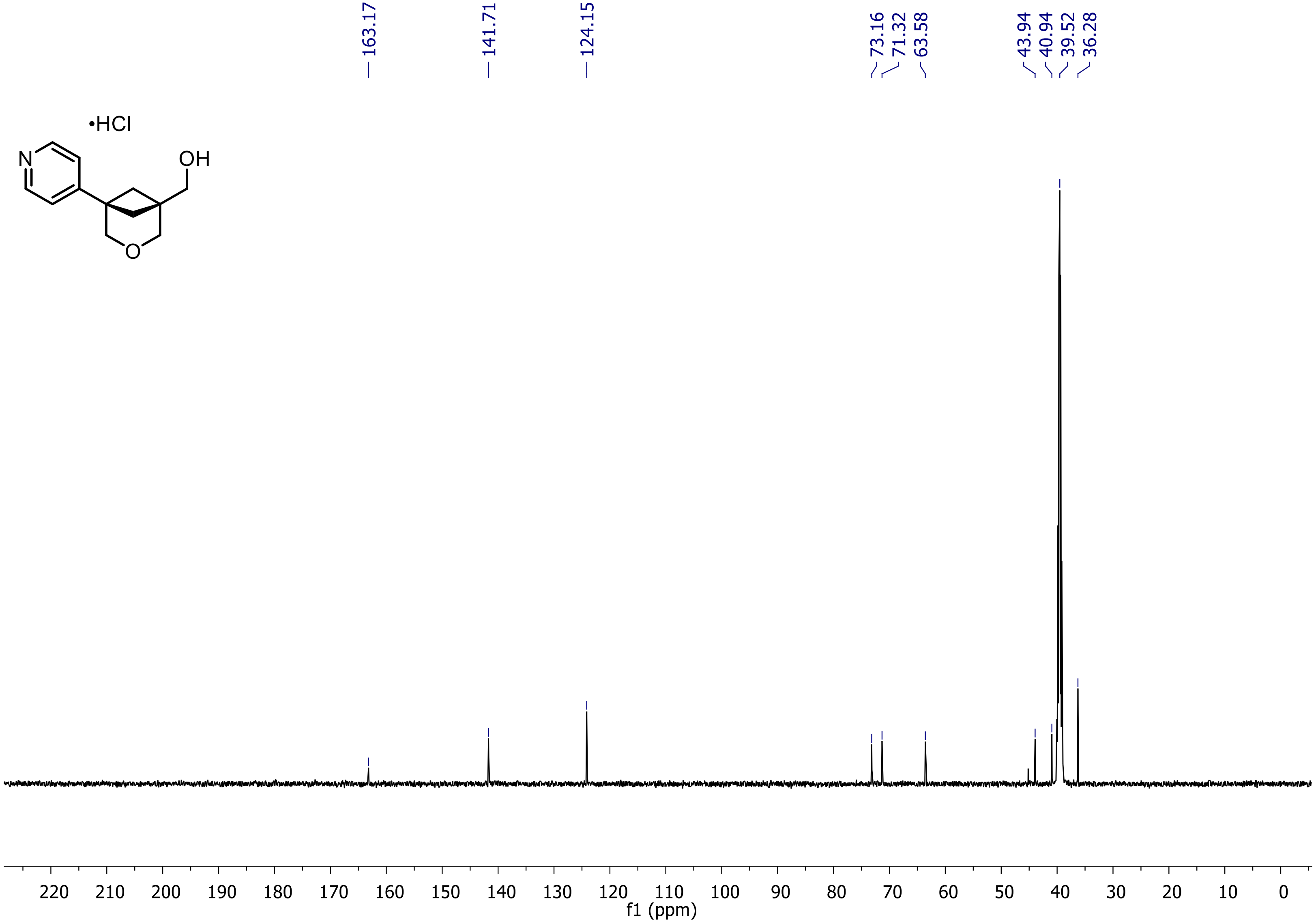

Compound 16a

^{1}H NMR (500 MHz, $CDCl_3$)

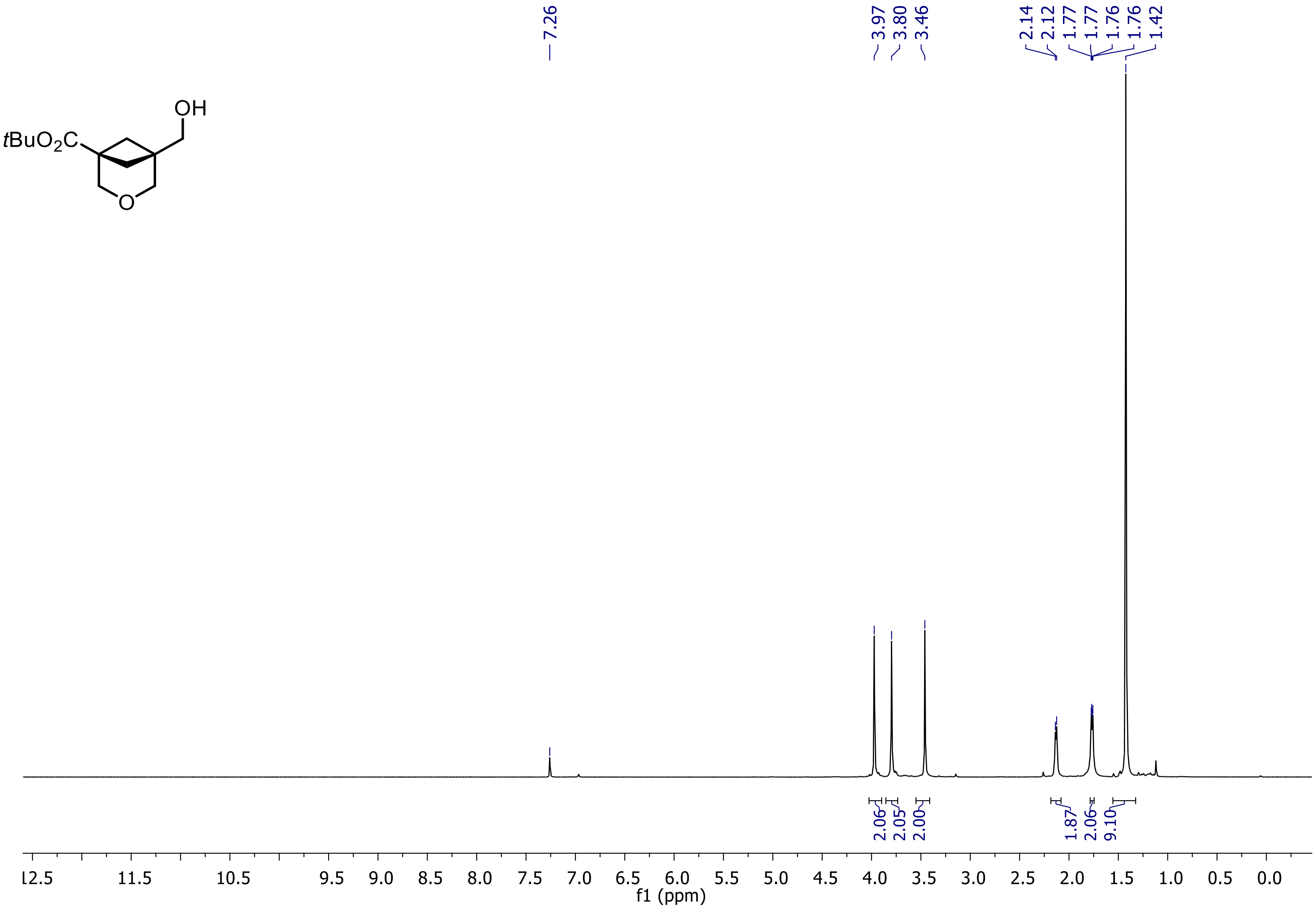

$^{13}C\{^1H\}$ NMR (151 MHz, $CDCl_3$)

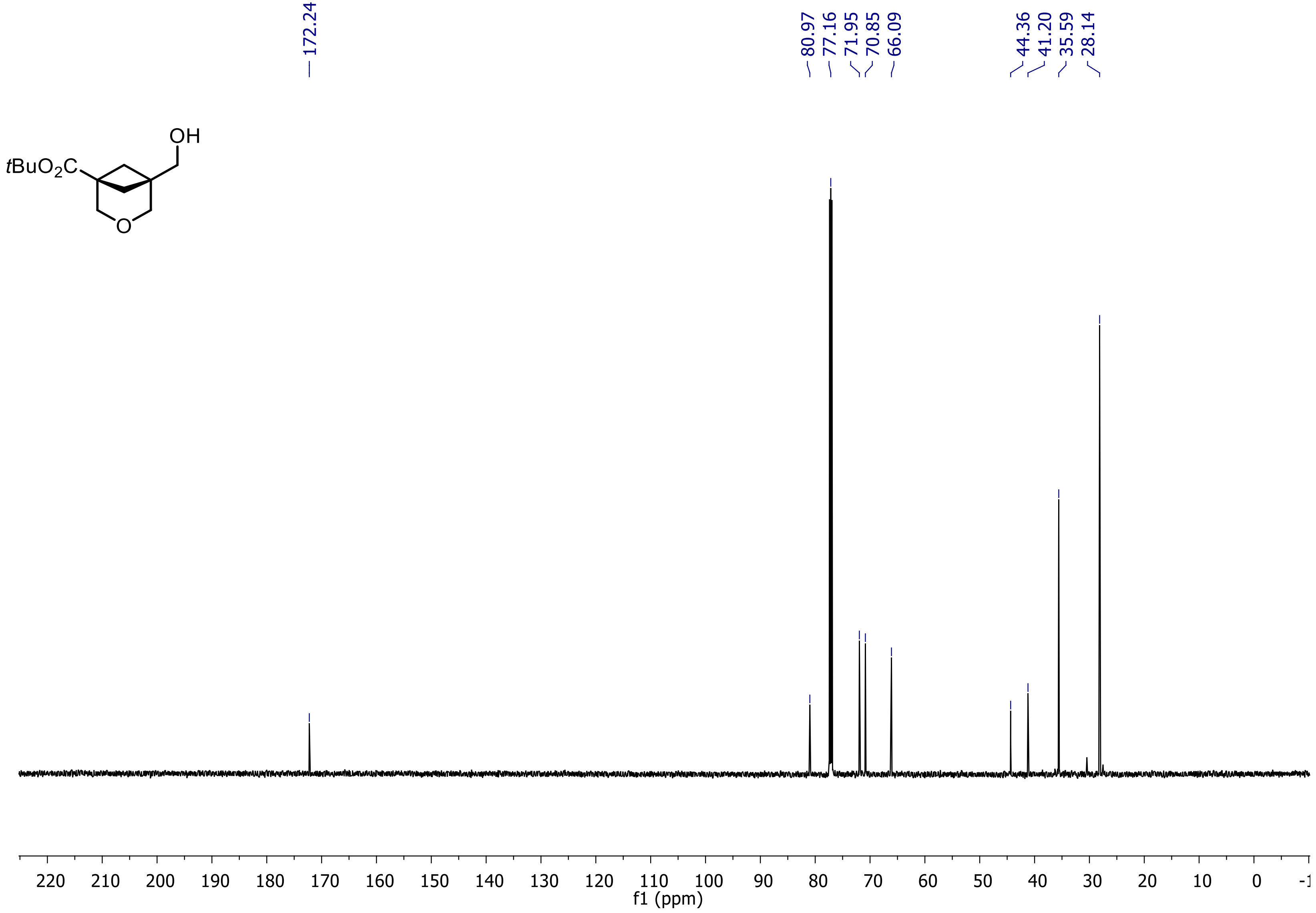

Compound 17a

^{1}H NMR (500 MHz, $CDCl_3$)

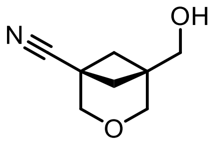

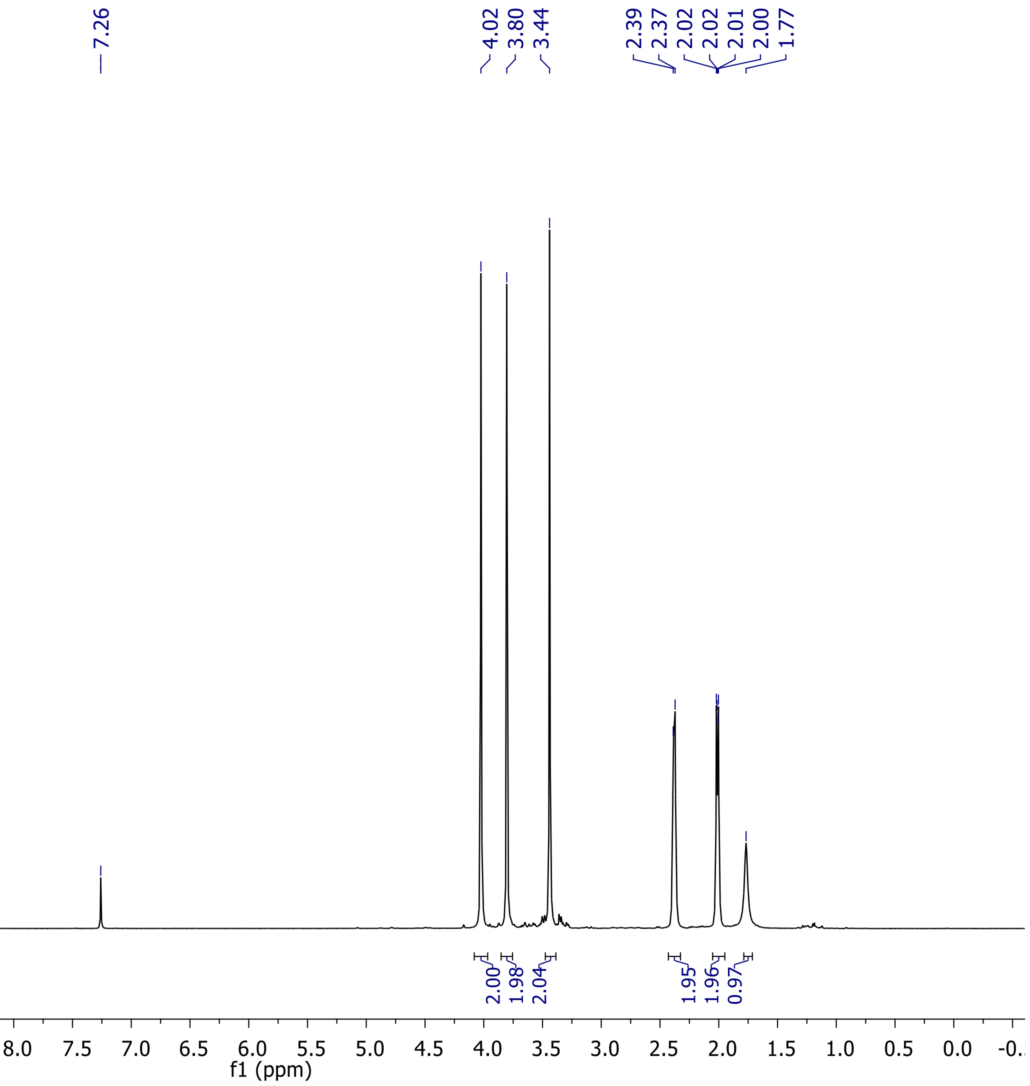

$^{13}C\{^1H\}$NMR (126 MHz, $CDCl_3$)

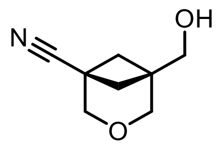

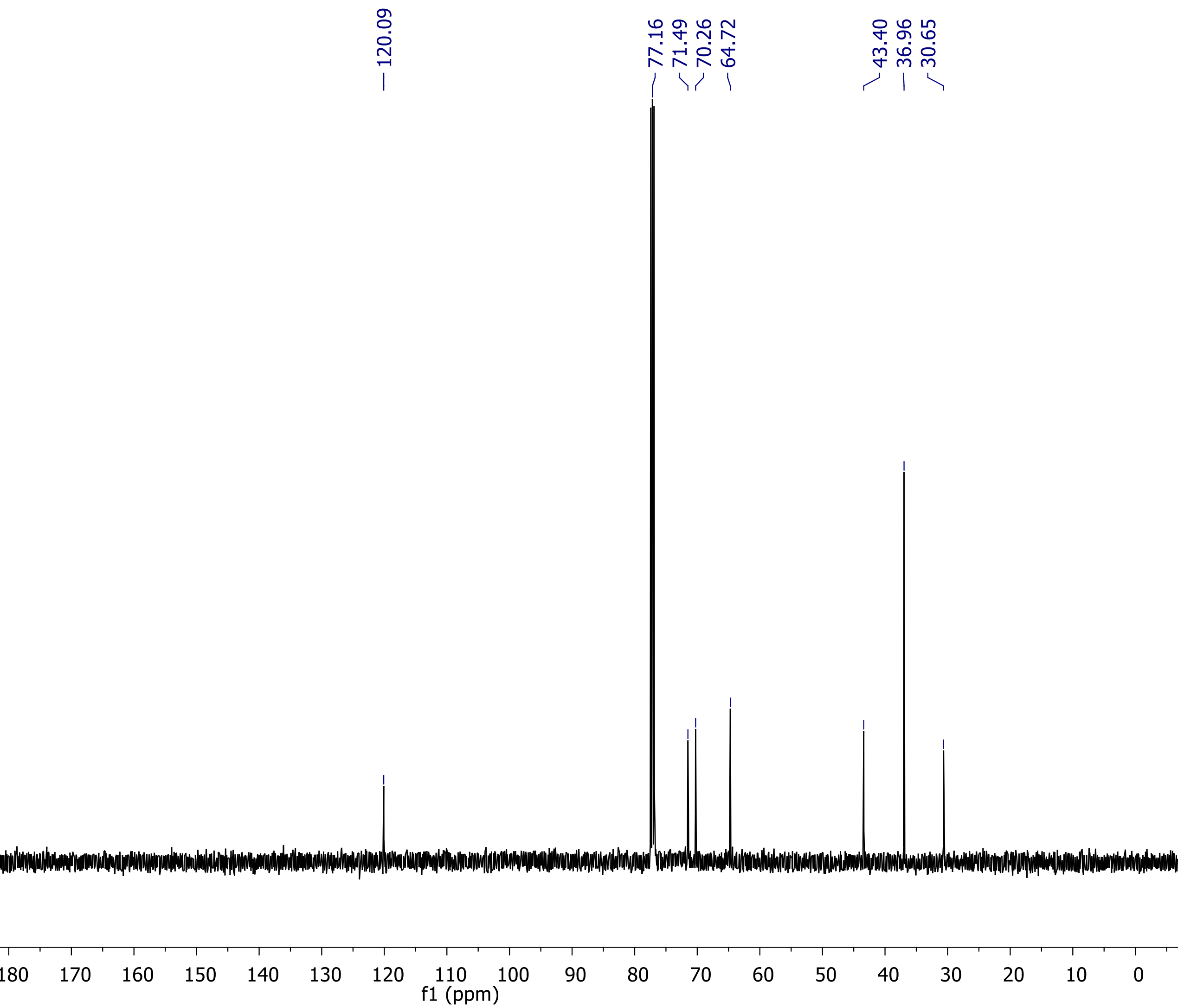

Compound 18a

^{1}H NMR (500 MHz, $CDCl_3$)

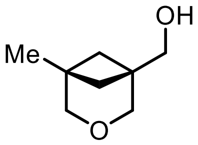

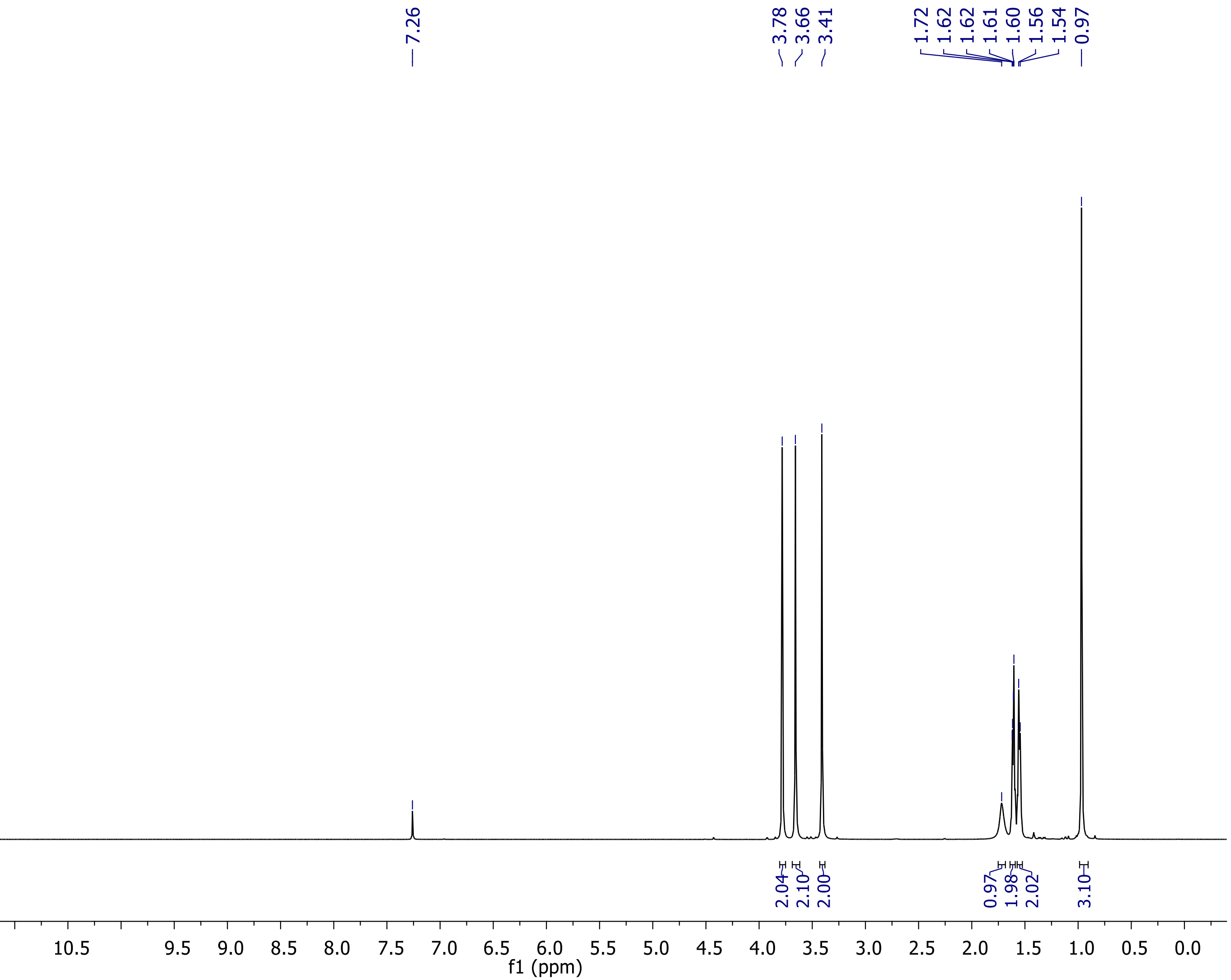

$^{13}C\{^1H\}$ NMR (151 MHz, $CDCl_3$)

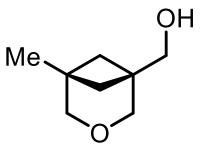

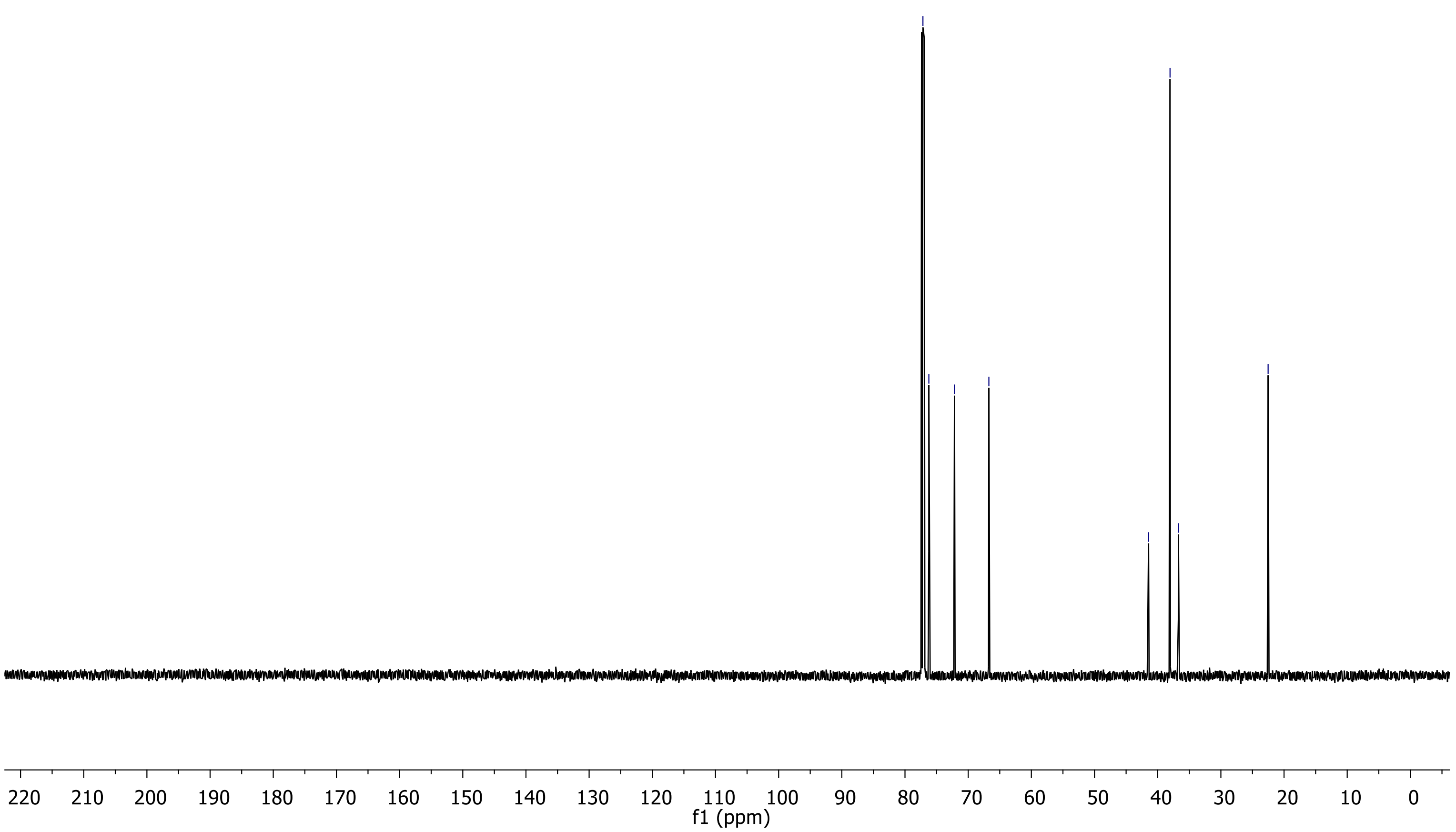

Compound 19a

^{1}H NMR (500 MHz, $CDCl_3$)

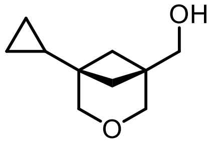

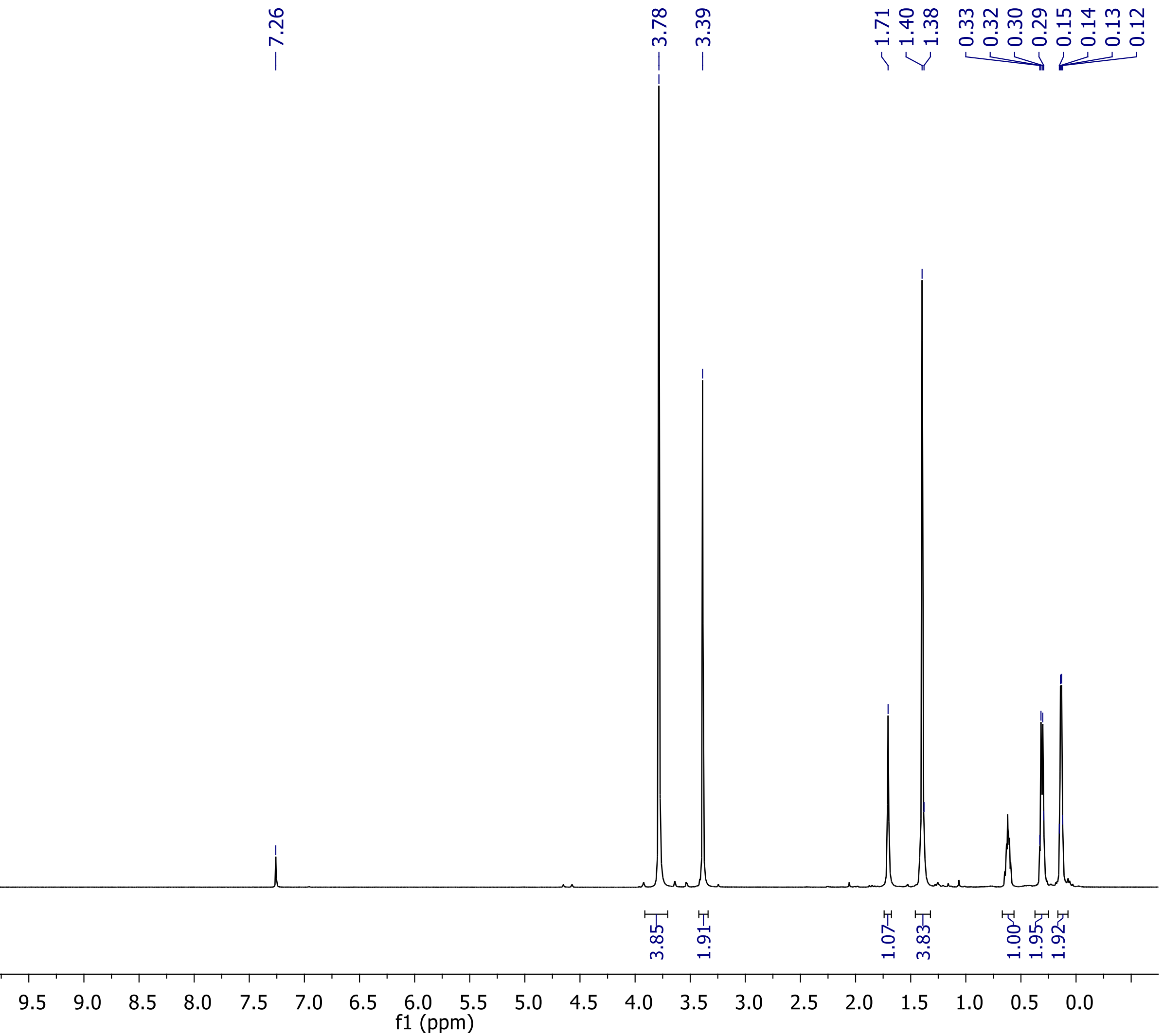

$^{13}C\{^{1}H\}$ NMR (126 MHz, $CDCl_3$)

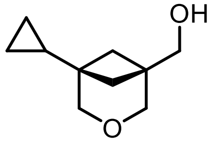

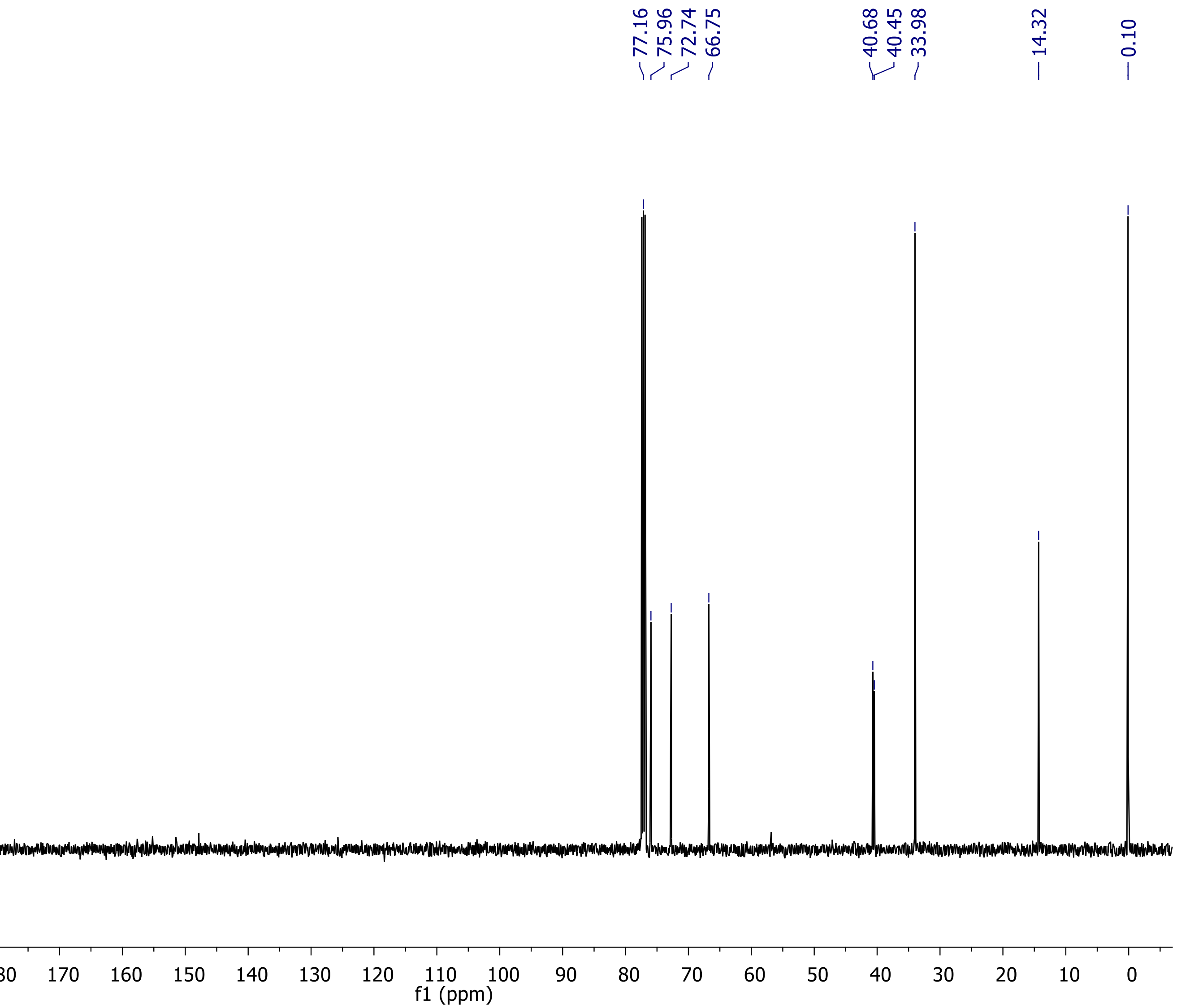

Compound 20a

^{1}H NMR (500 MHz, $CDCl_3$)

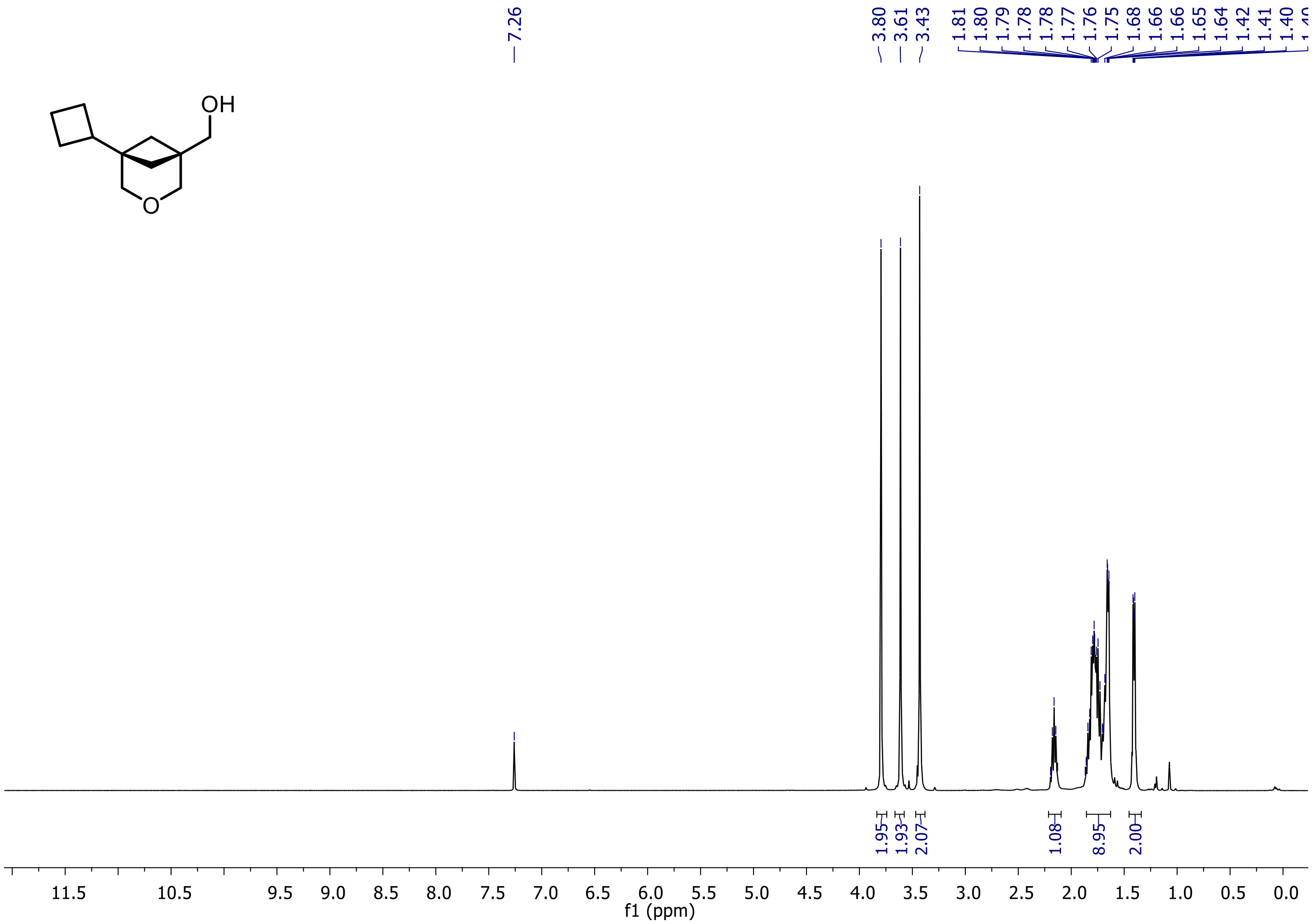

$^{13}C\{^1H\}$ NMR (126 MHz, $CDCl_3$)

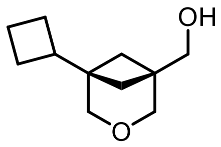

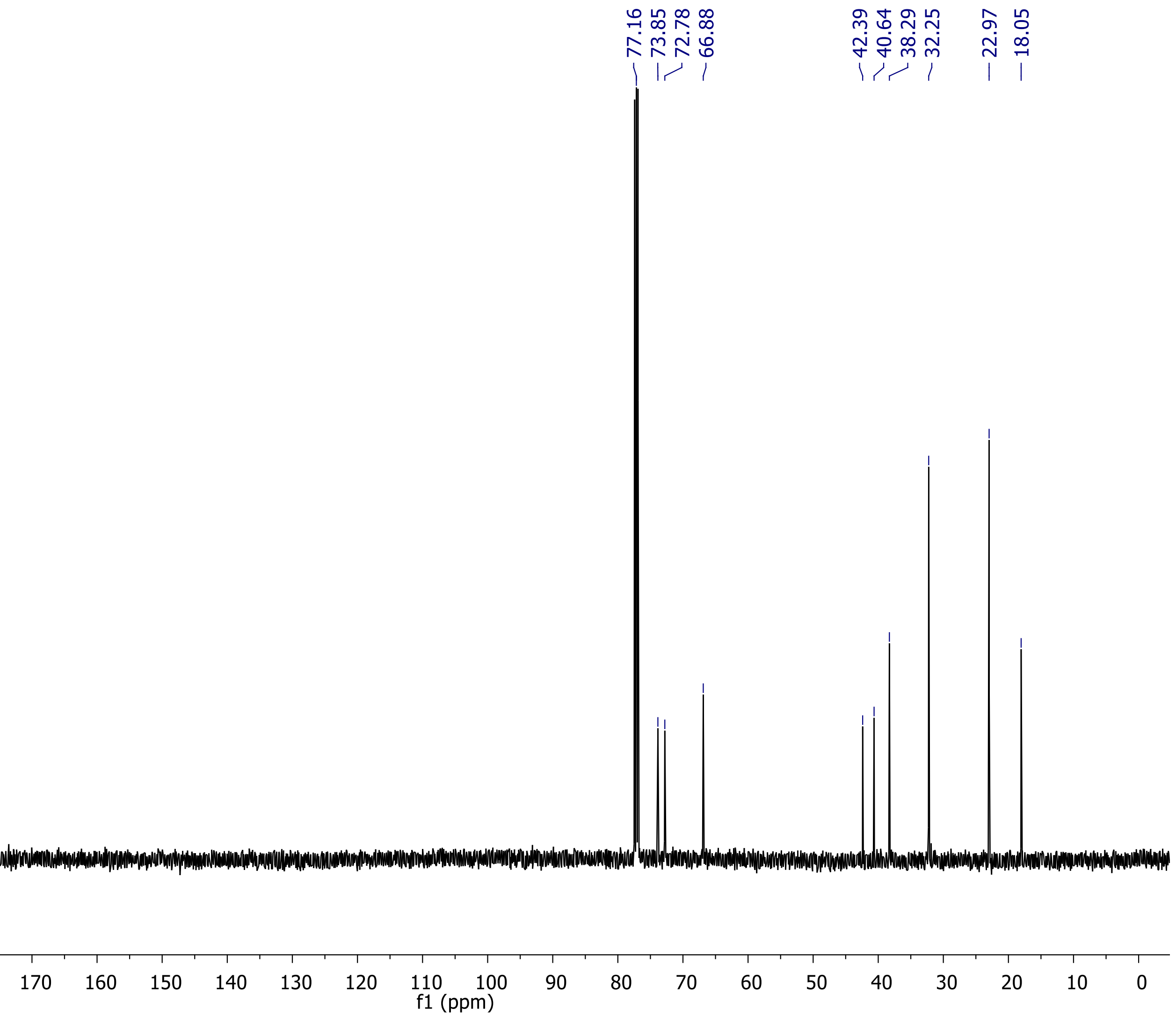

Compound 21a

^{1}H NMR (500 MHz, $CDCl_3$)

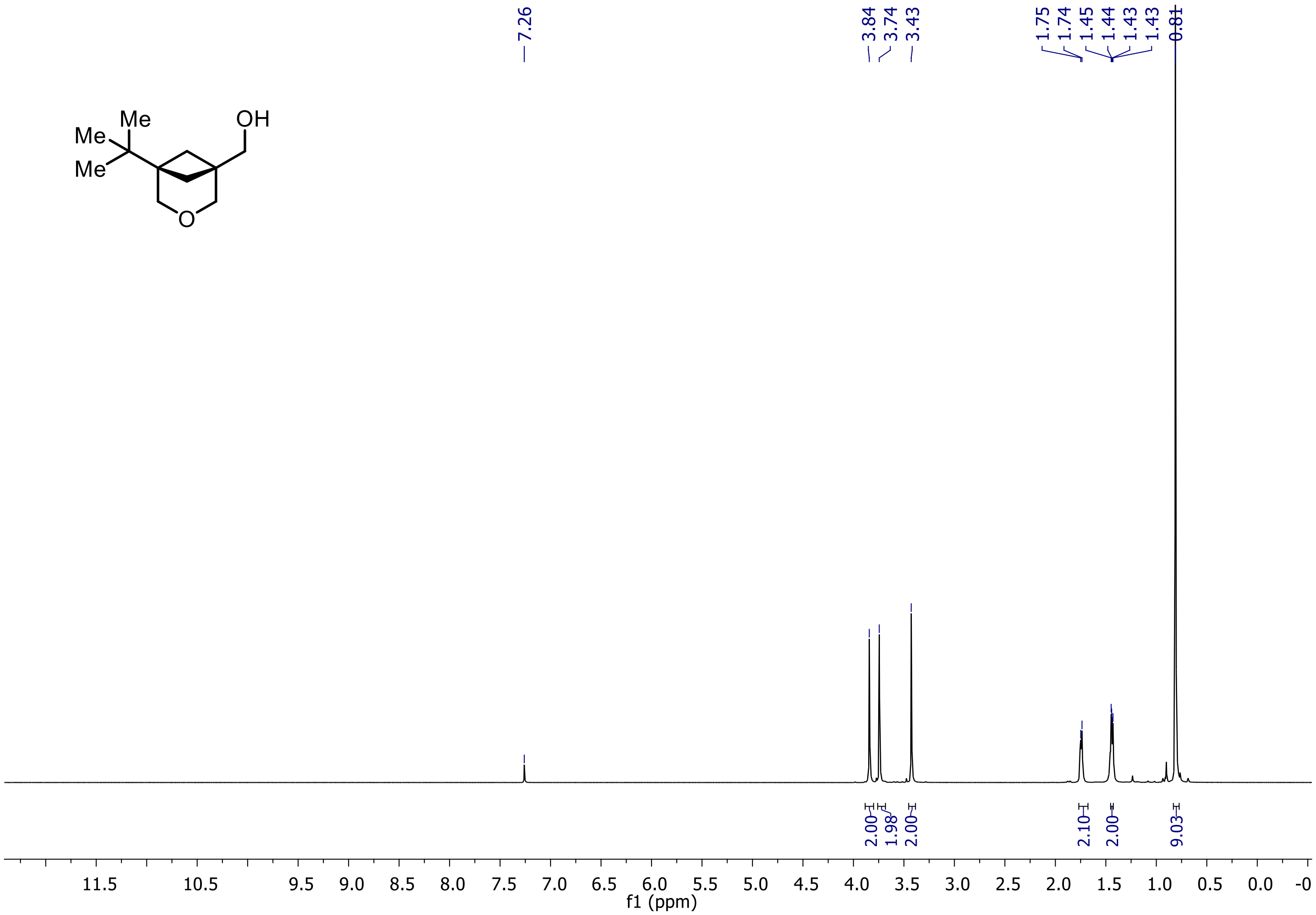

$^{13}C\{^{1}H\}$ NMR (126 MHz, $CDCl_3$)

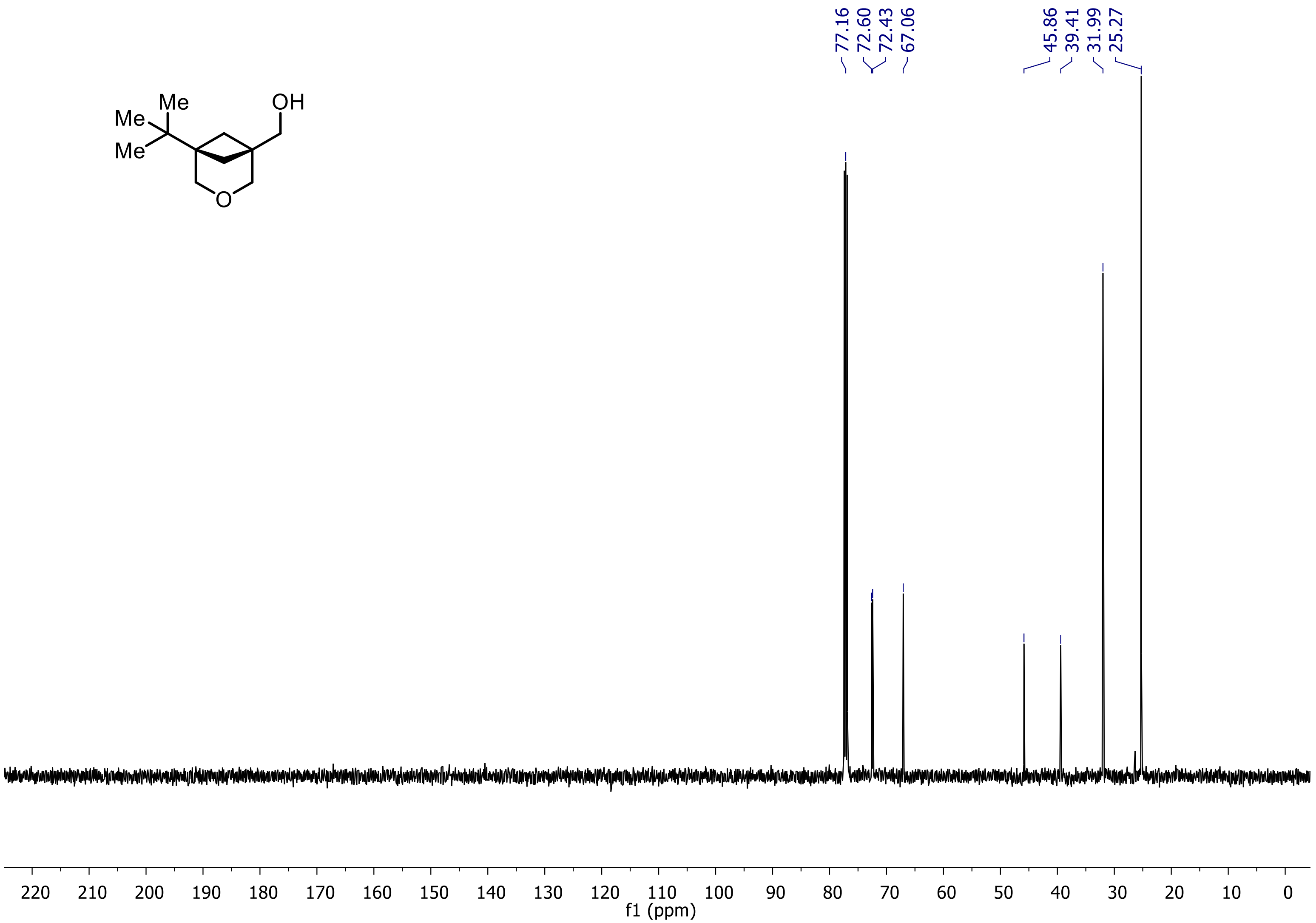

Compound 22a

^{1}H NMR (500 MHz, $CDCl_3$)

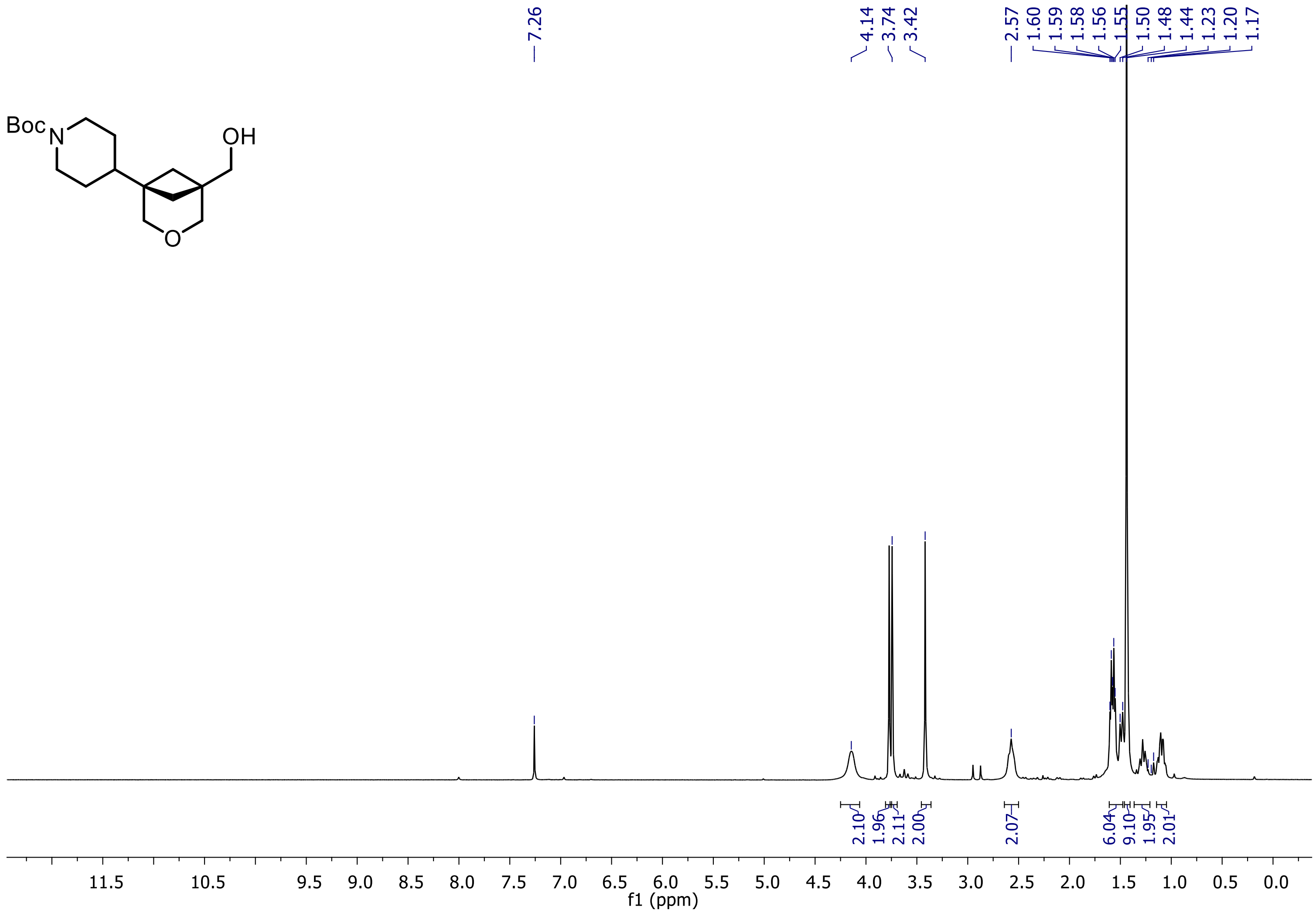

$^{13}C\{^1H\}$ NMR (151 MHz, $CDCl_3$)

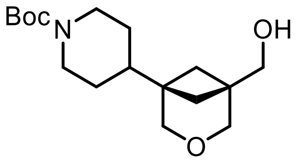

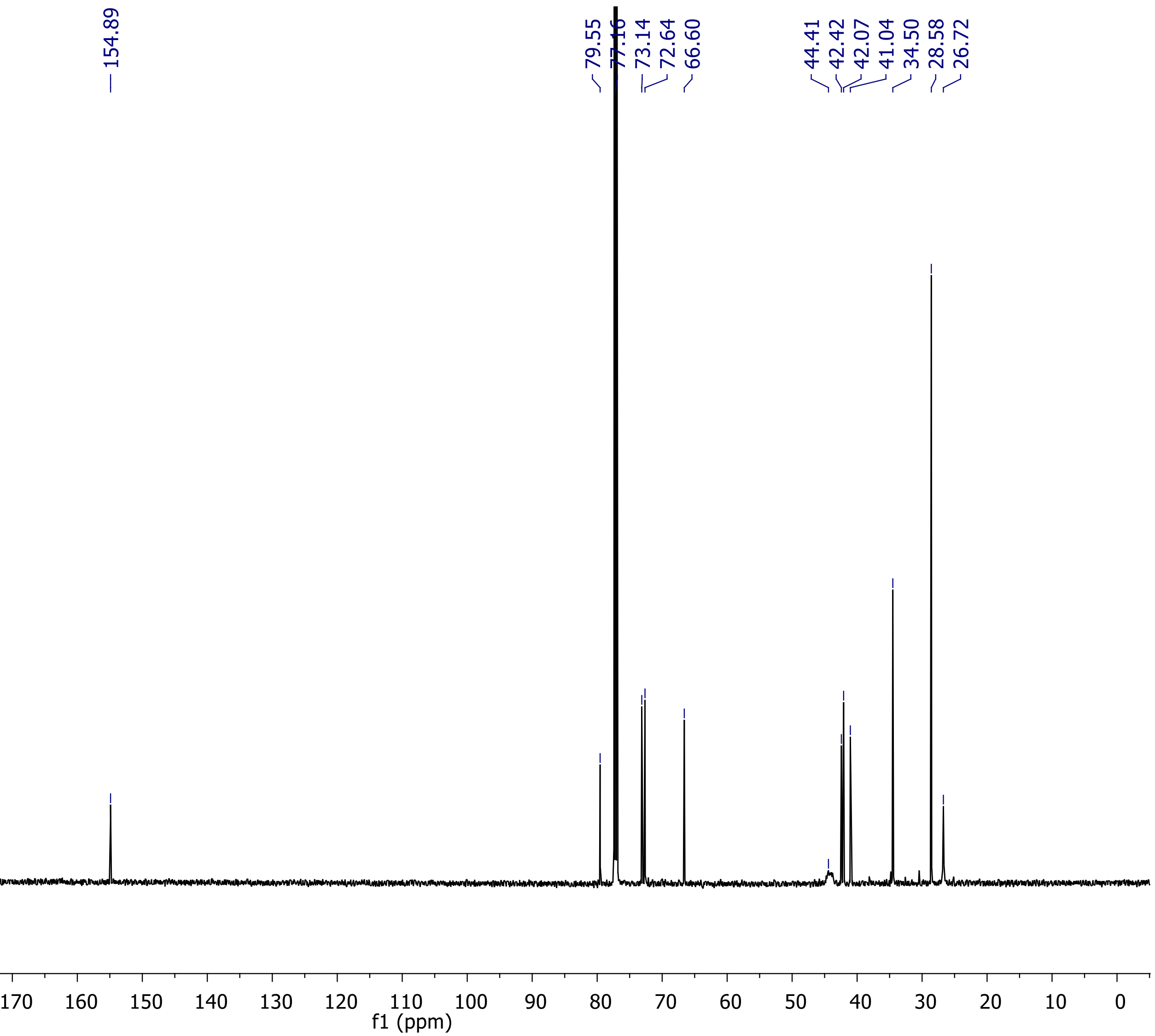

Compound 1b

^{1}H NMR (500 MHz, DMSO-d_6)

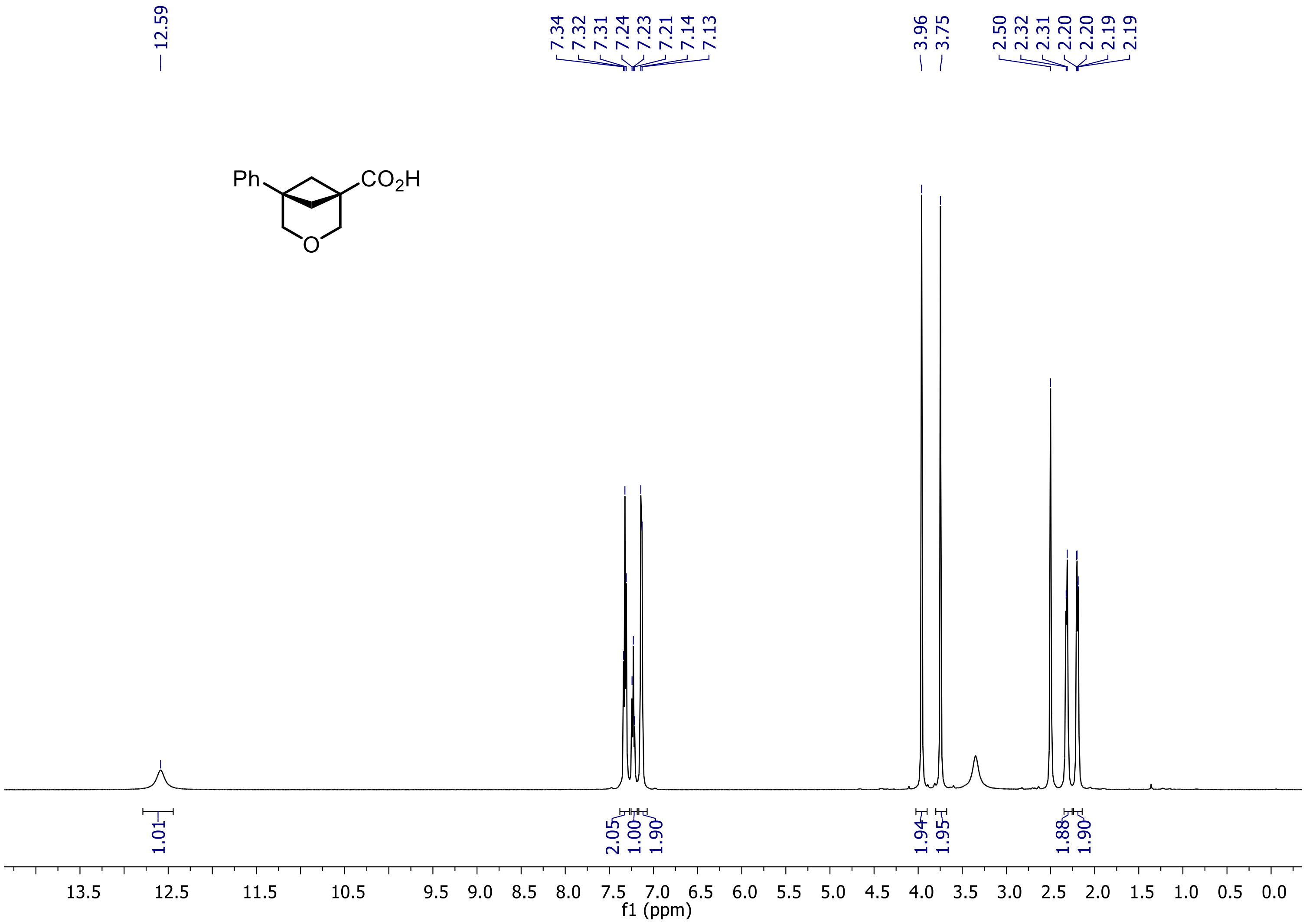

$^{13}C\{^1H\}$ NMR (126 MHz, DMSO-d_6)

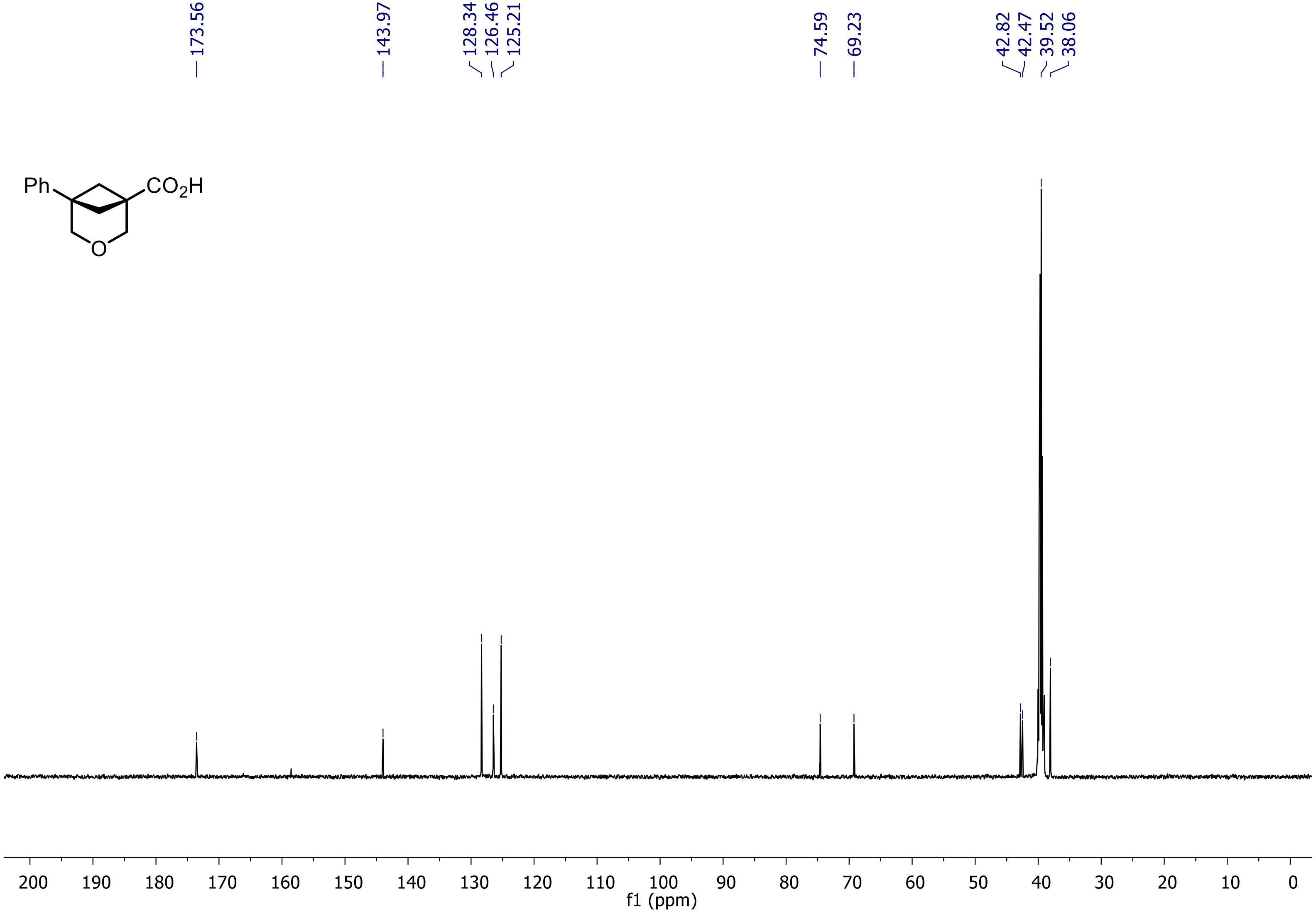

Compound 5b

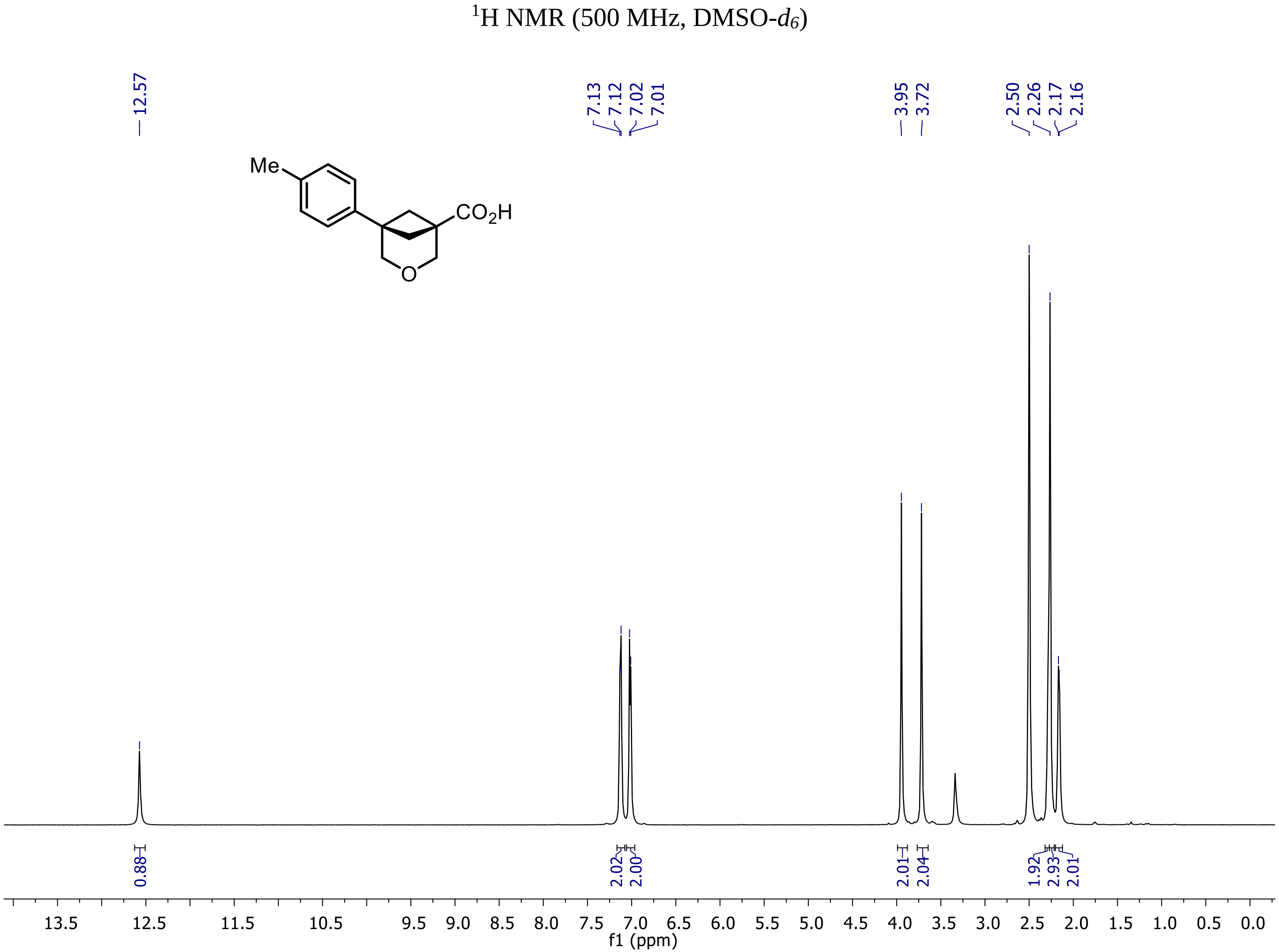

^{1}H NMR (500 MHz, DMSO-d_6)
12.57
7.13
7.12
7.02
7.01
3.95
3.72
2.50
2.26
2.17
2.16
Me
CO2H
O
0.88
2.02
2.00
2.01
2.04
1.92
2.93
2.01
13.5
12.5
11.5
10.5
9.5
9.0
8.5
8.0
7.5
7.0
6.5
6.0
5.5
5.0
4.5
4.0
3.5
3.0
2.5
2.0
1.5
1.0
0.5
0.0
f1 (ppm)

$^{13}C\{^{1}H\}$ NMR (126 MHz, DMSO-d_6)

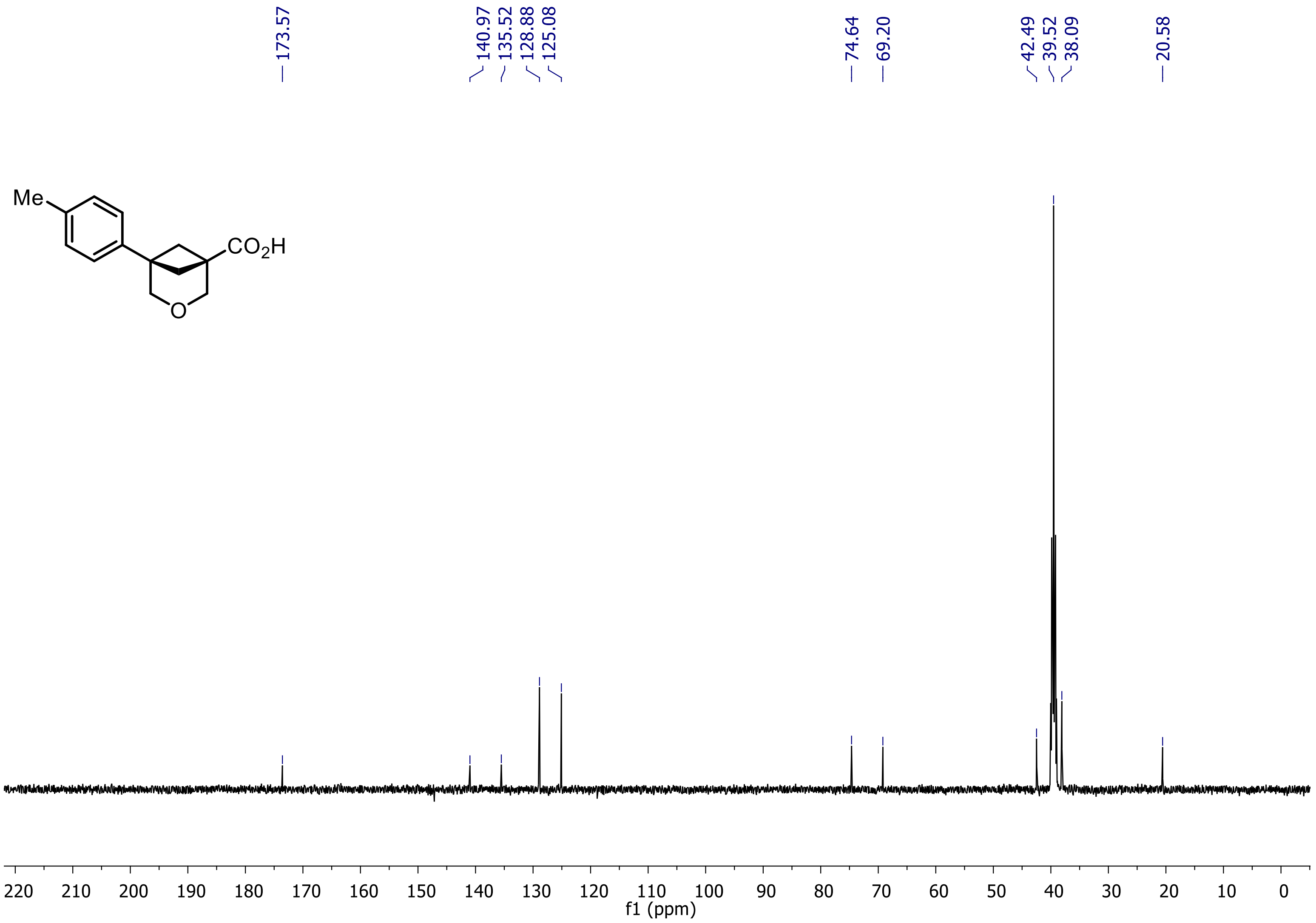

Compound 6b

^{1}H NMR (500 MHz, $CDCl_3$)

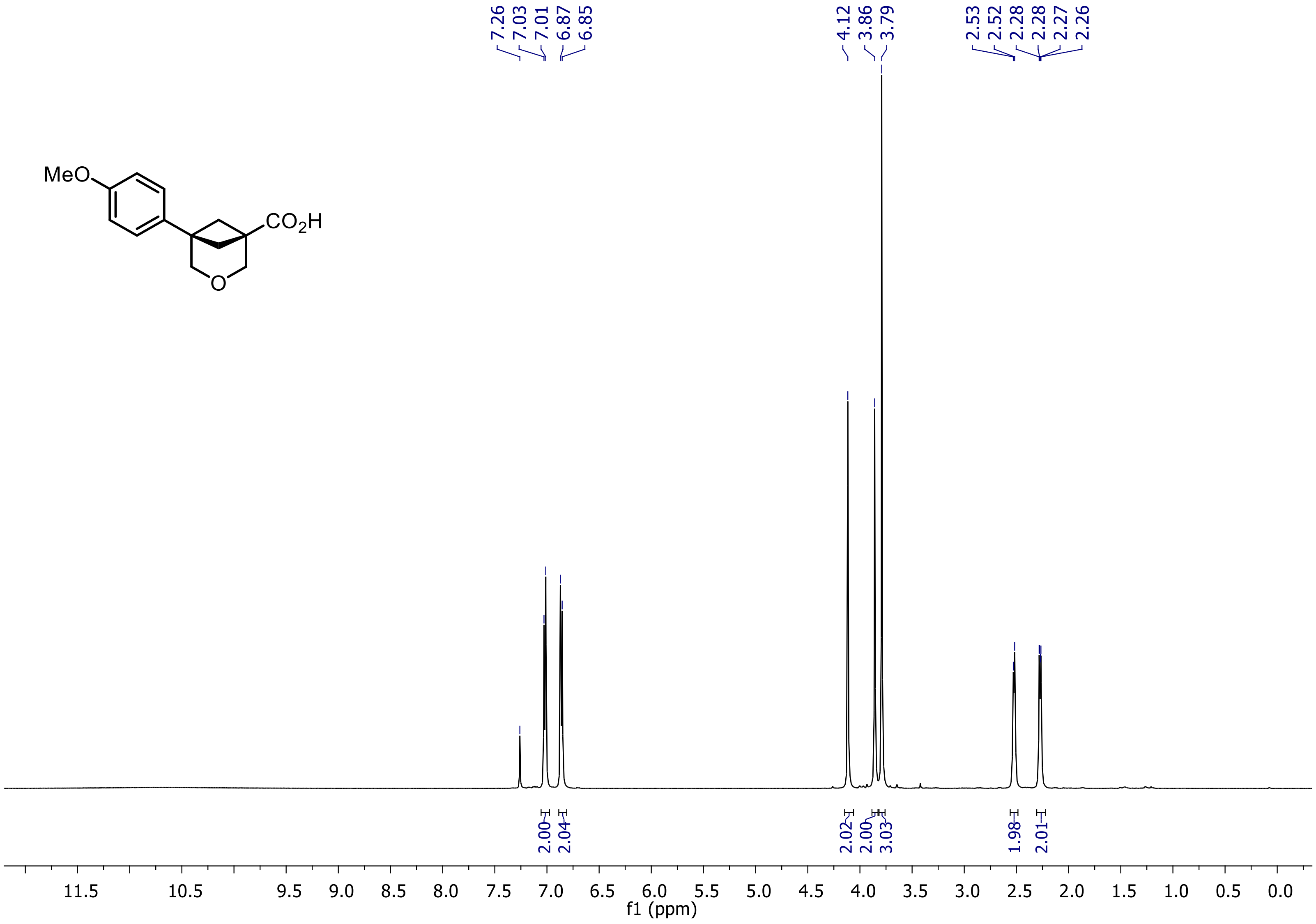

$^{13}C\{^1H\}$ NMR (126 MHz, $CDCl_3$)

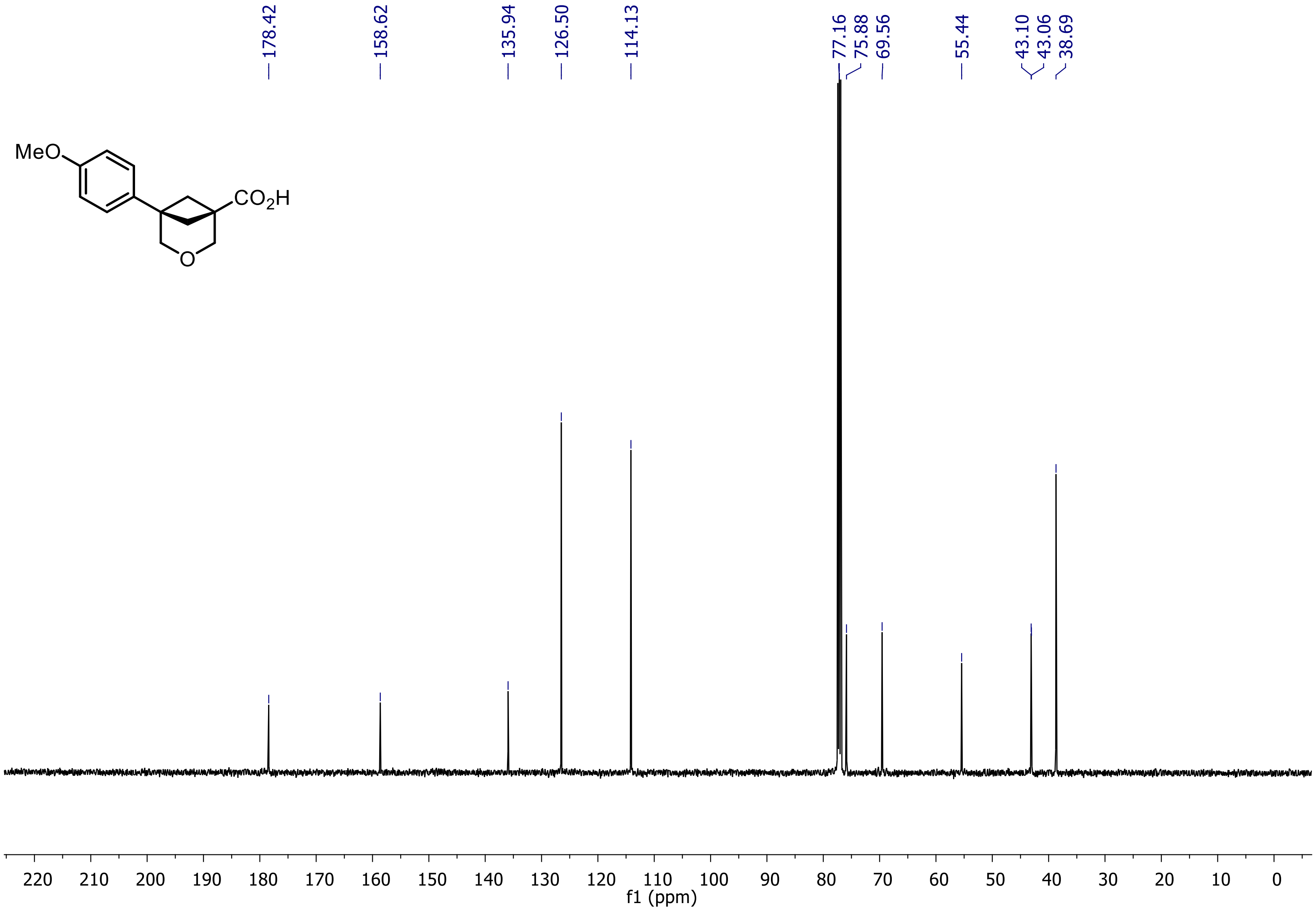

Compound 7b

^{1}H NMR (500 MHz, DMSO-d_6)

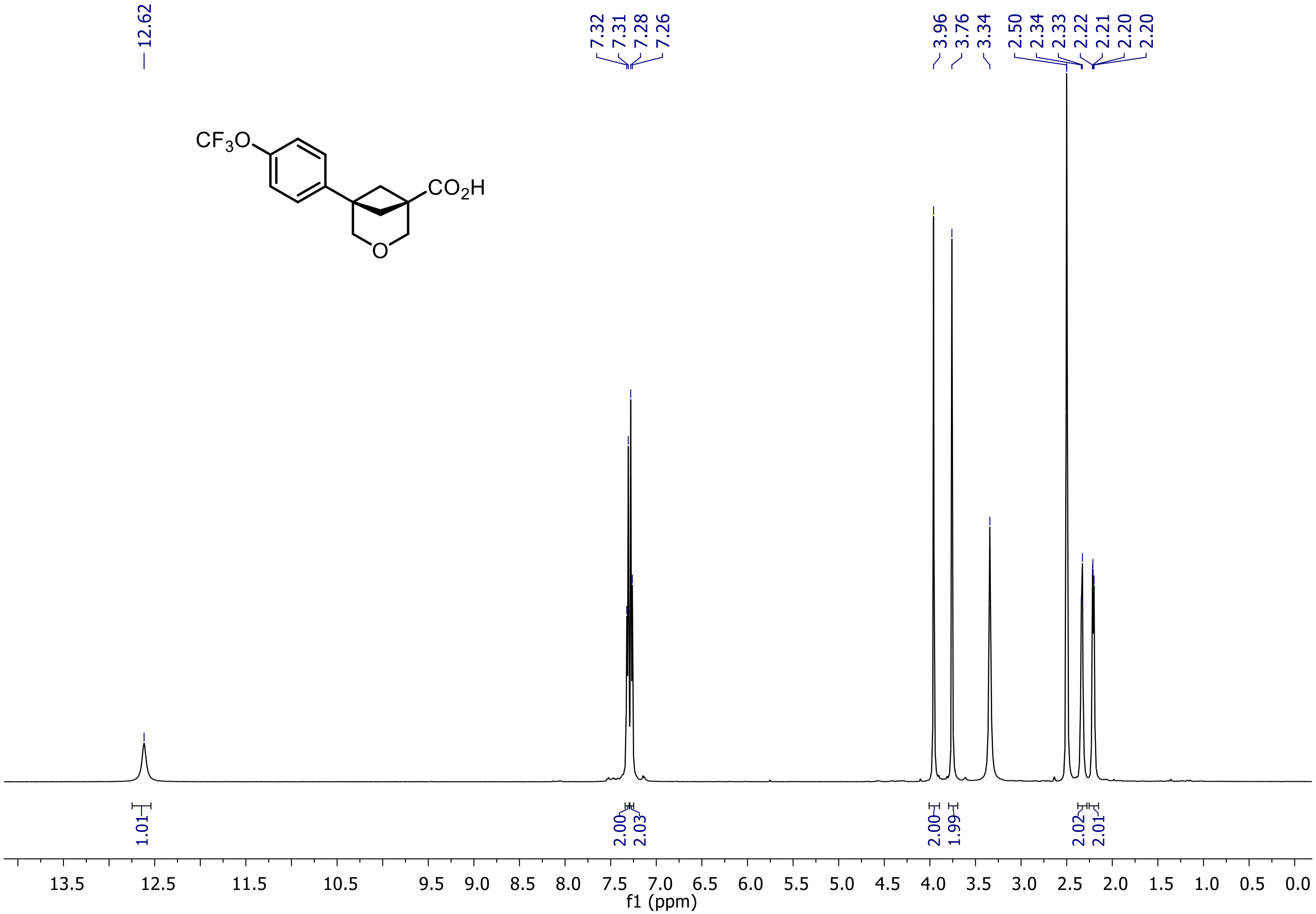

$^{13}C\{^{1}H\}$ NMR (151 MHz, DMSO-d_6)

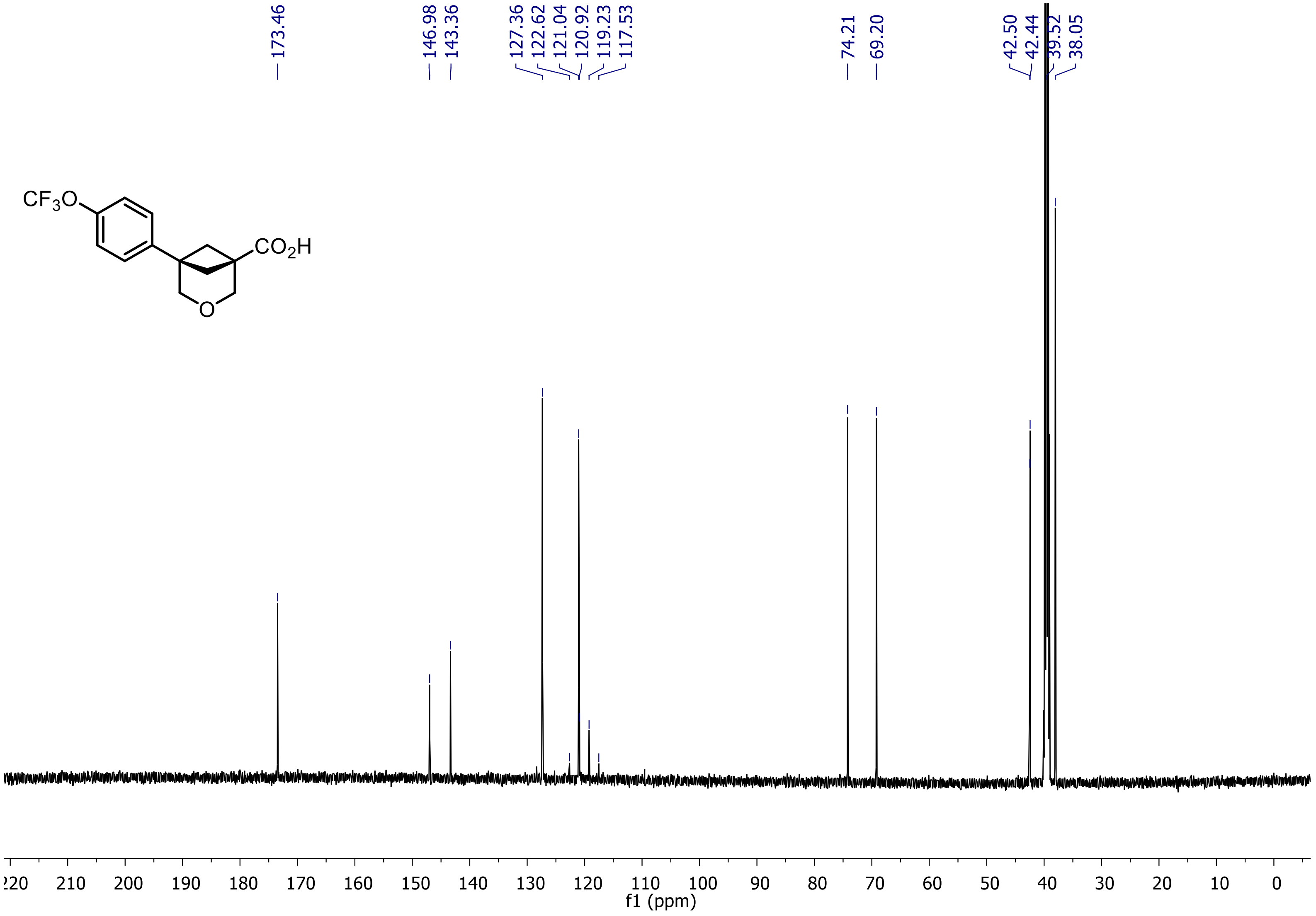

$^{19}F\{^{1}H\}$ NMR (376 MHz, DMSO-d_6)

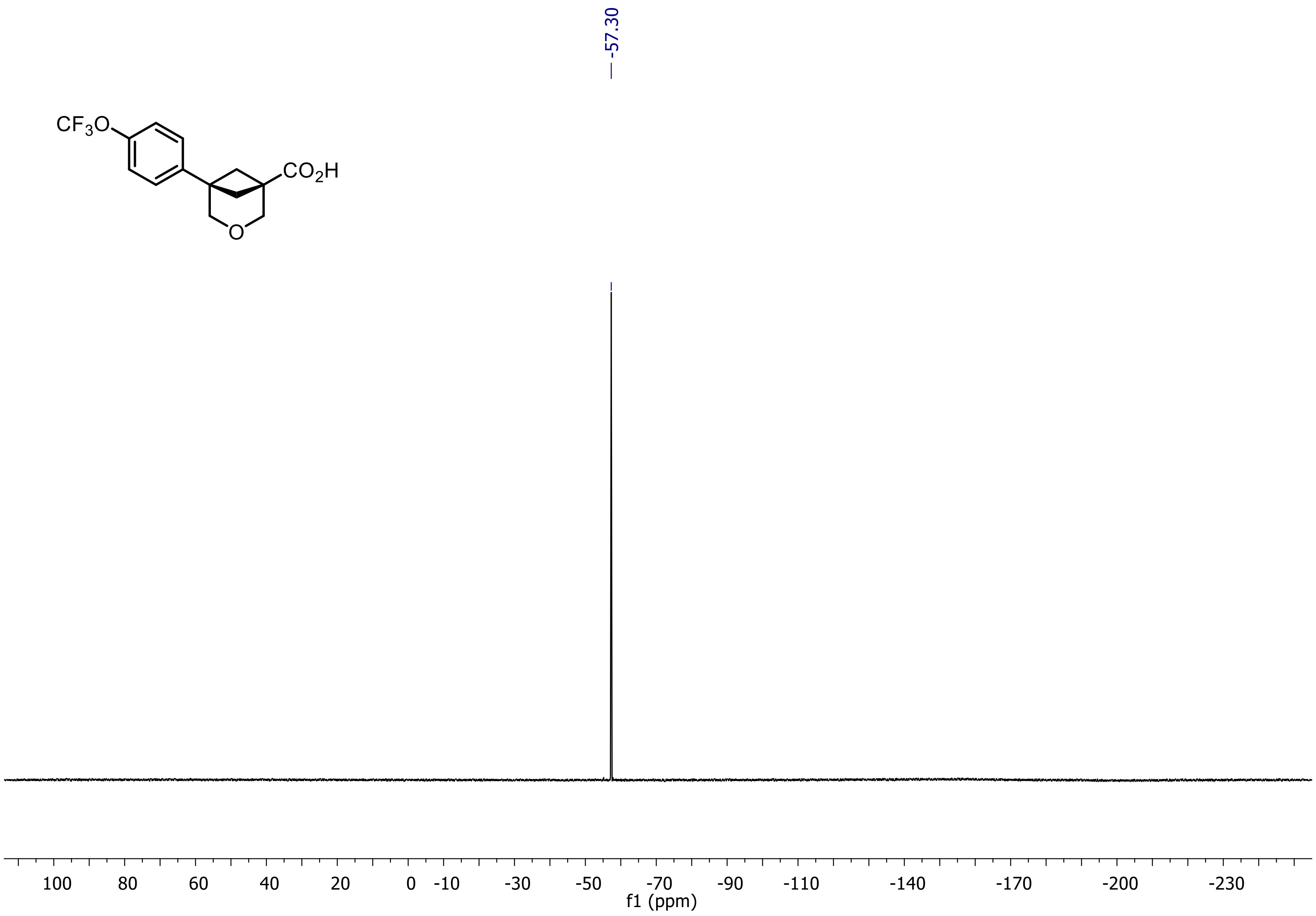

Compound 8b

^{1}H NMR (500 MHz, DMSO-d_6)

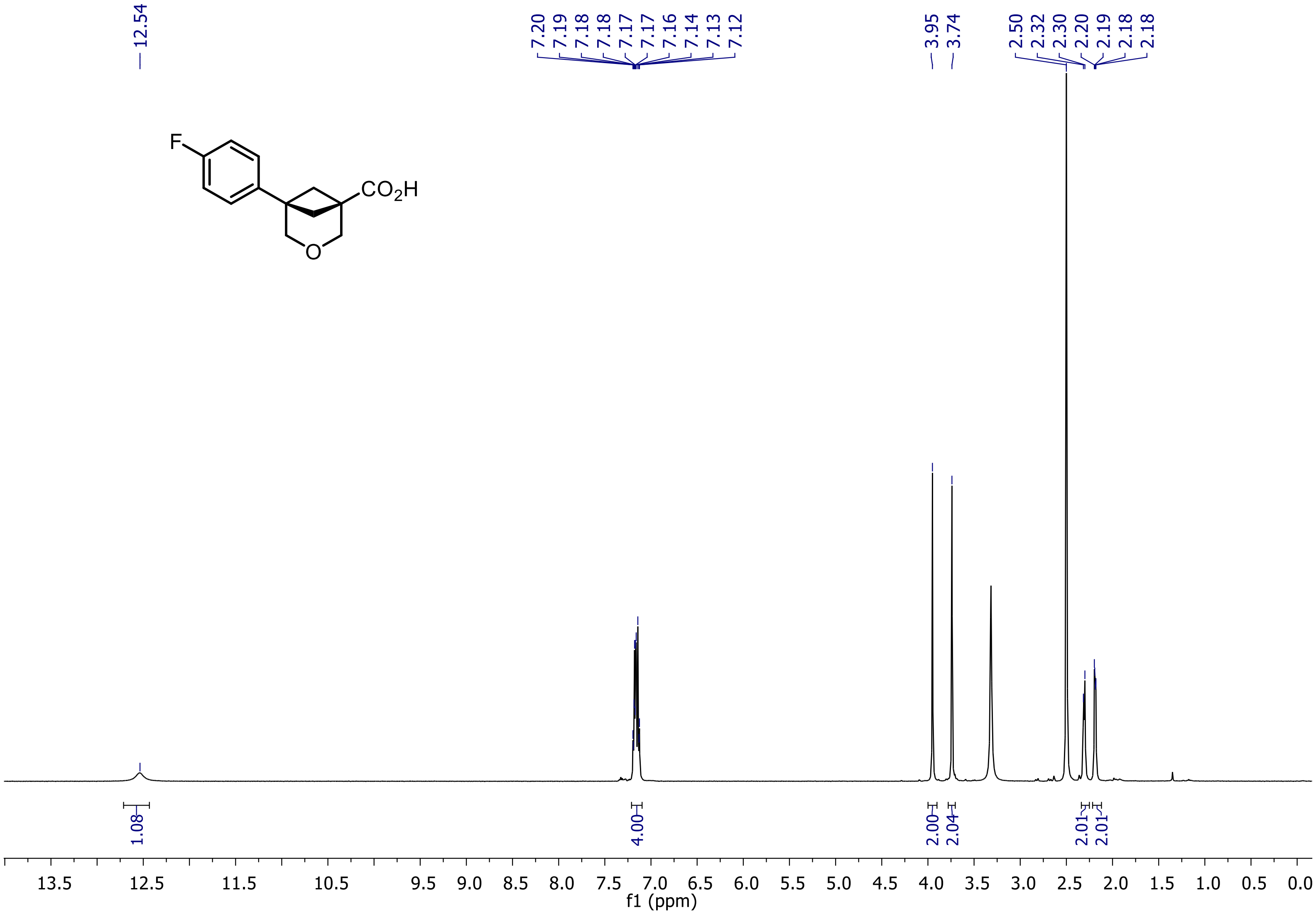

$^{13}C\{^{1}H\}$ NMR (151 MHz, DMSO-d_6)

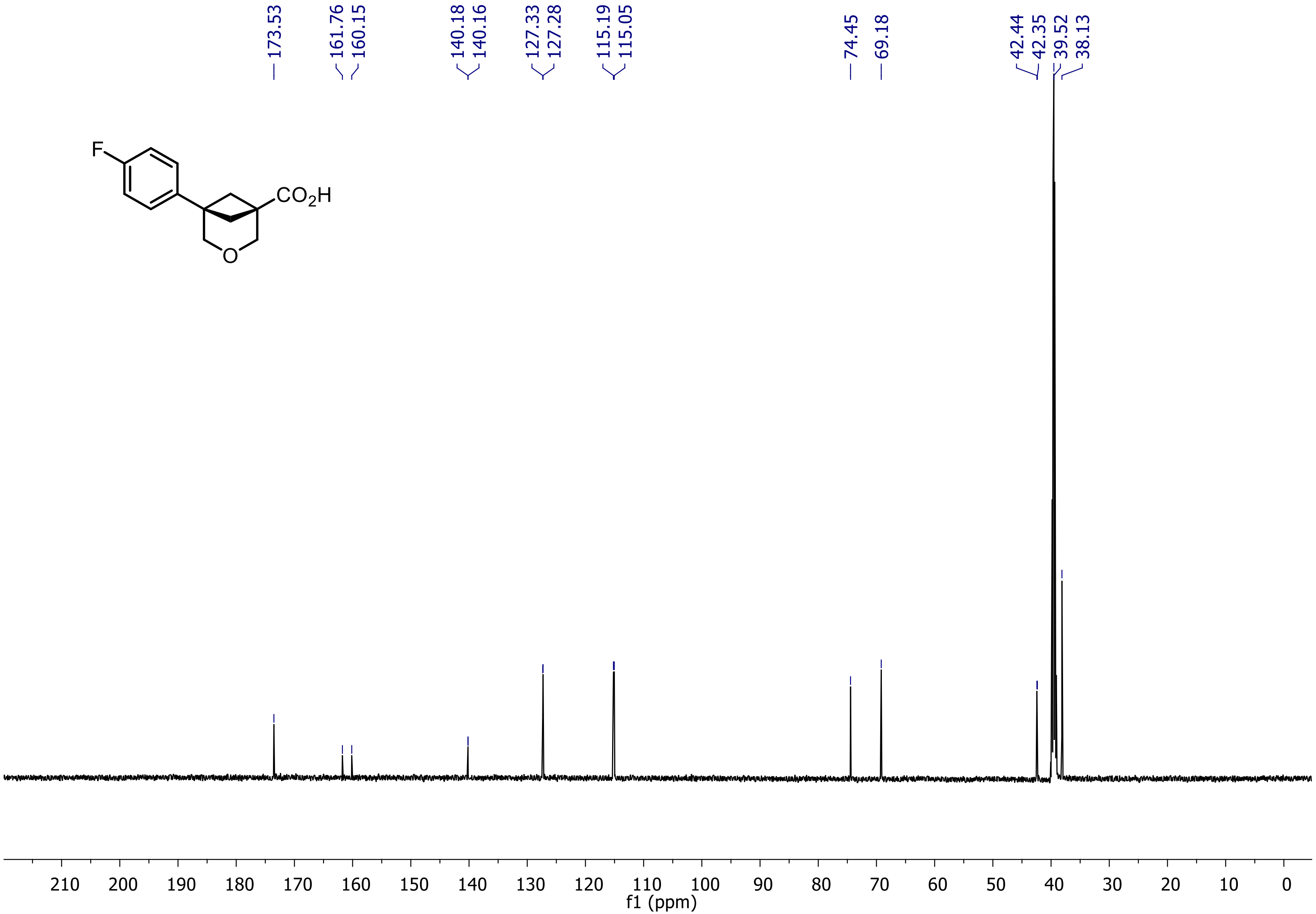

$^{19}F\{^{1}H\}$ NMR (376 MHz, DMSO-d_6)

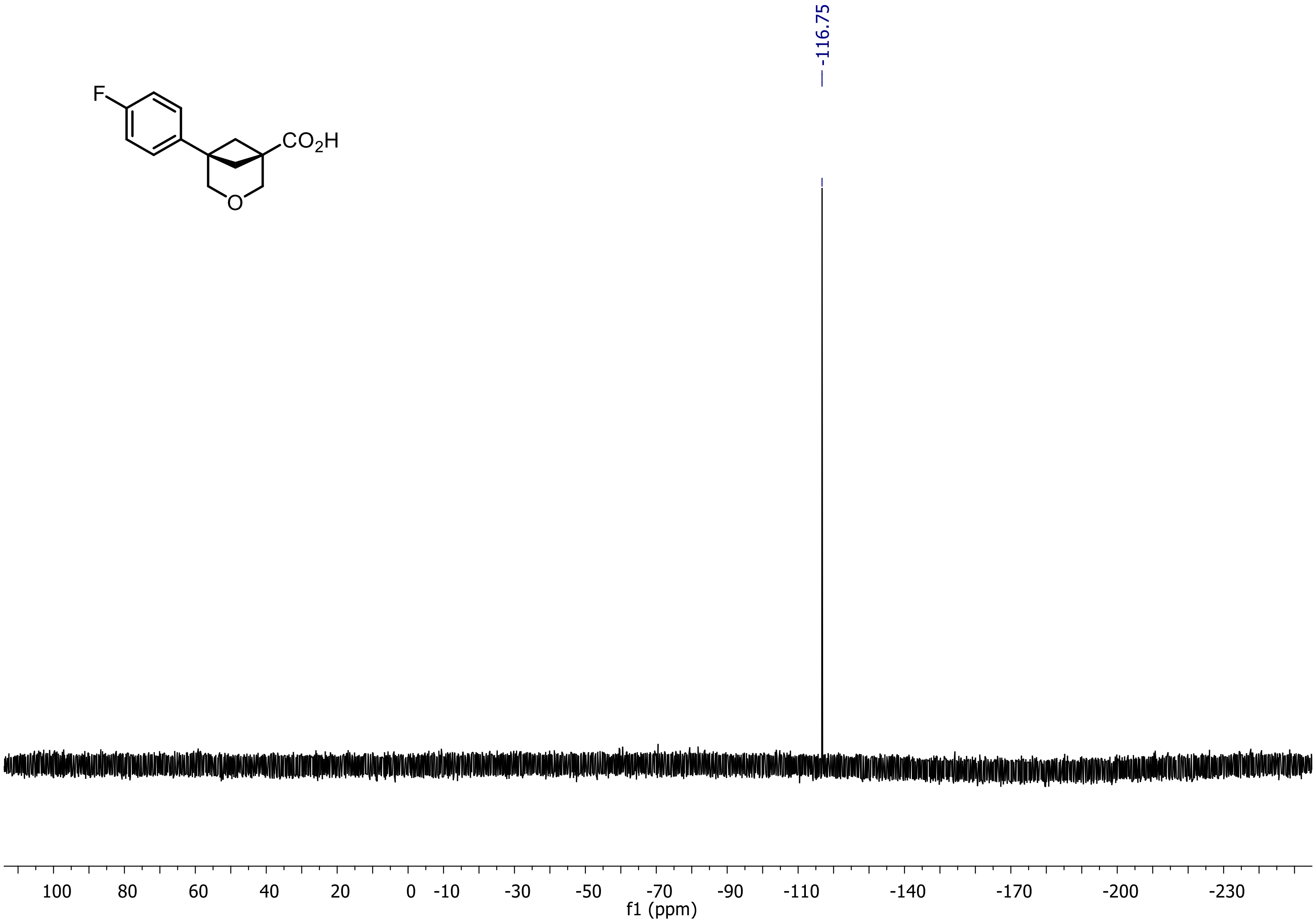

Compound 9b

^{1}H NMR (500 MHz, DMSO-d_6)

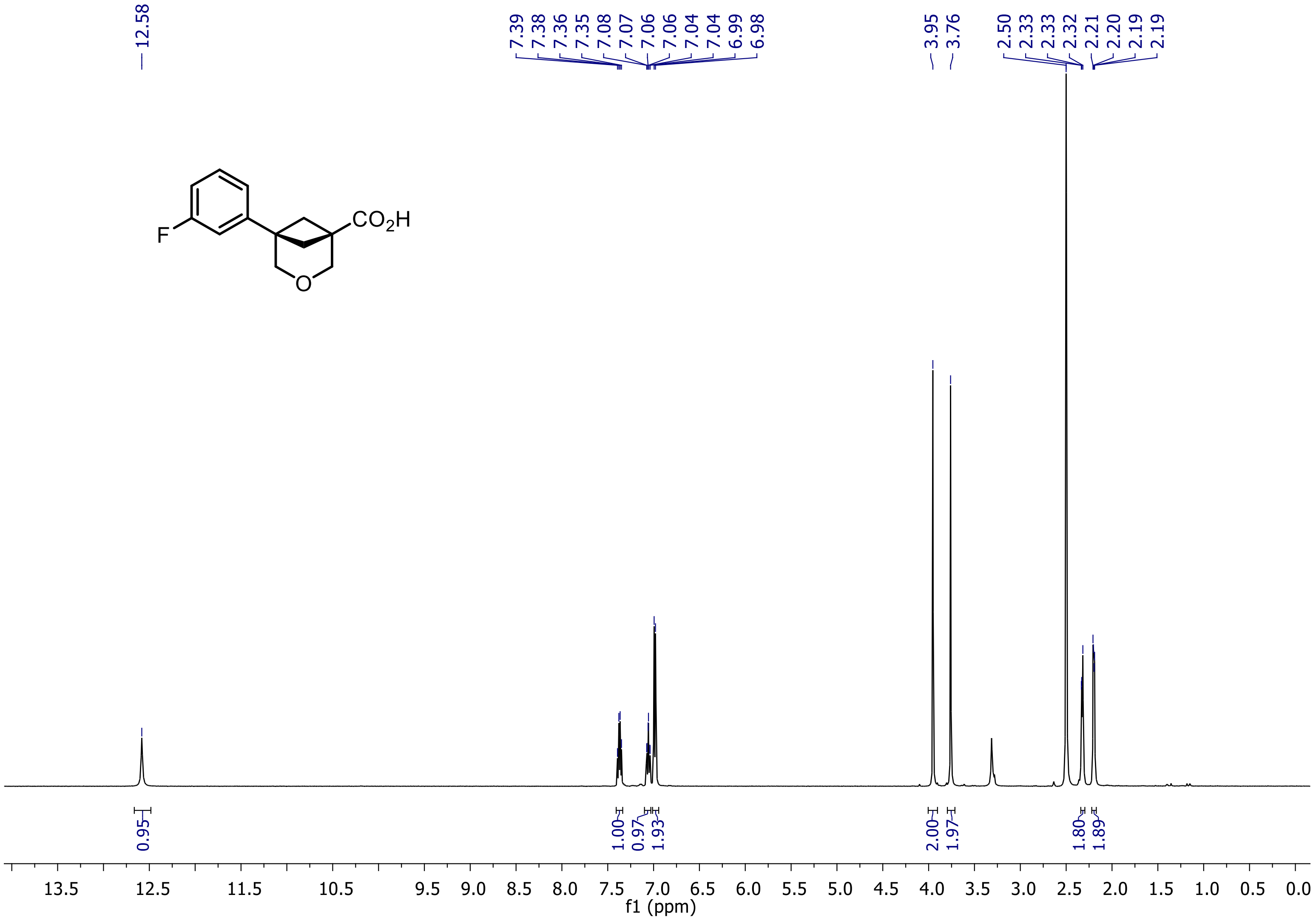

$^{13}C\{^{1}H\}$ NMR (126 MHz, DMSO-d_6)

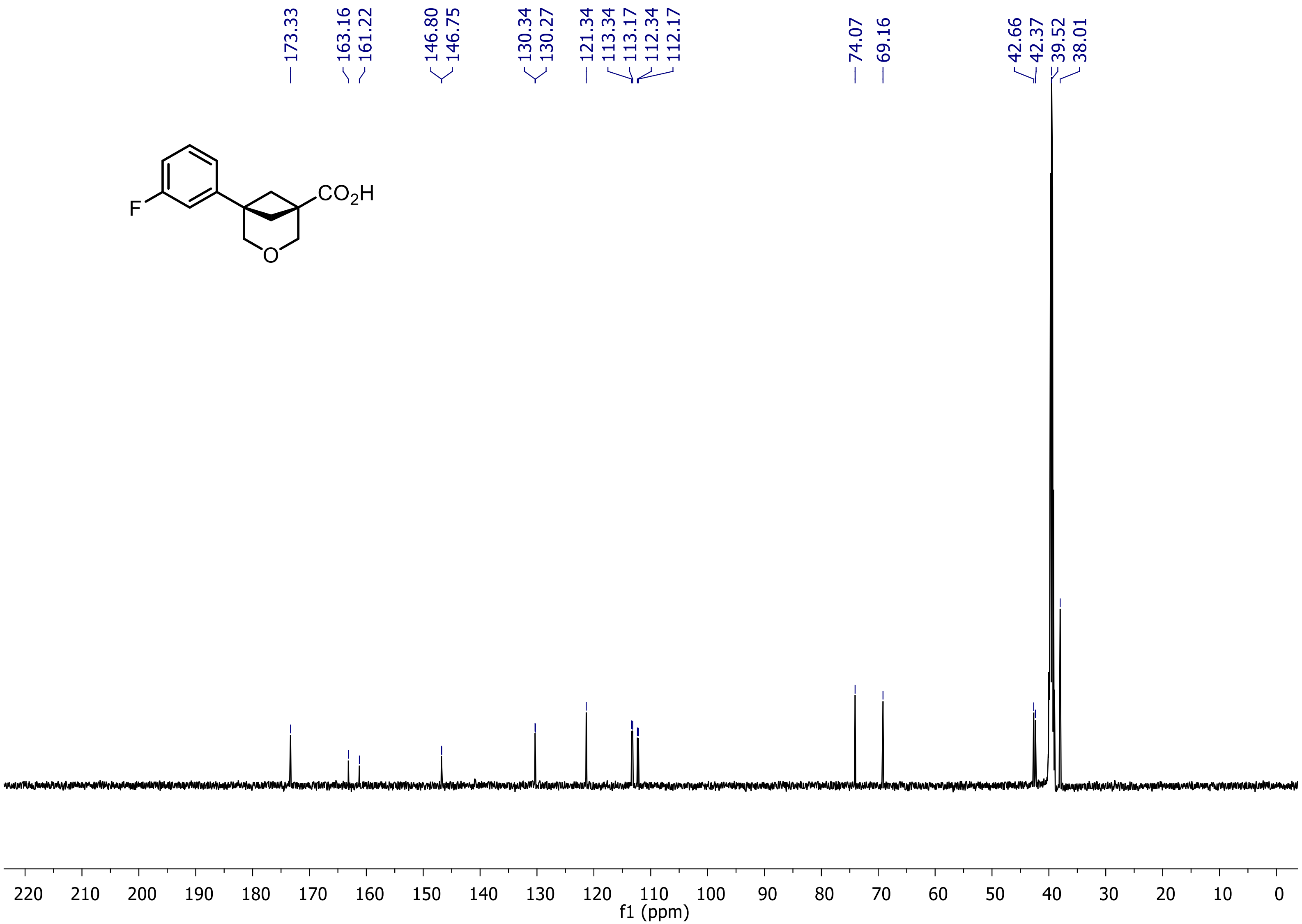

$^{19}F\{^{1}H\}$ NMR (376 MHz, DMSO-d_6)

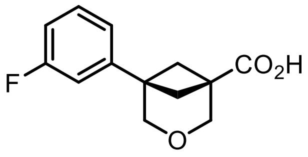

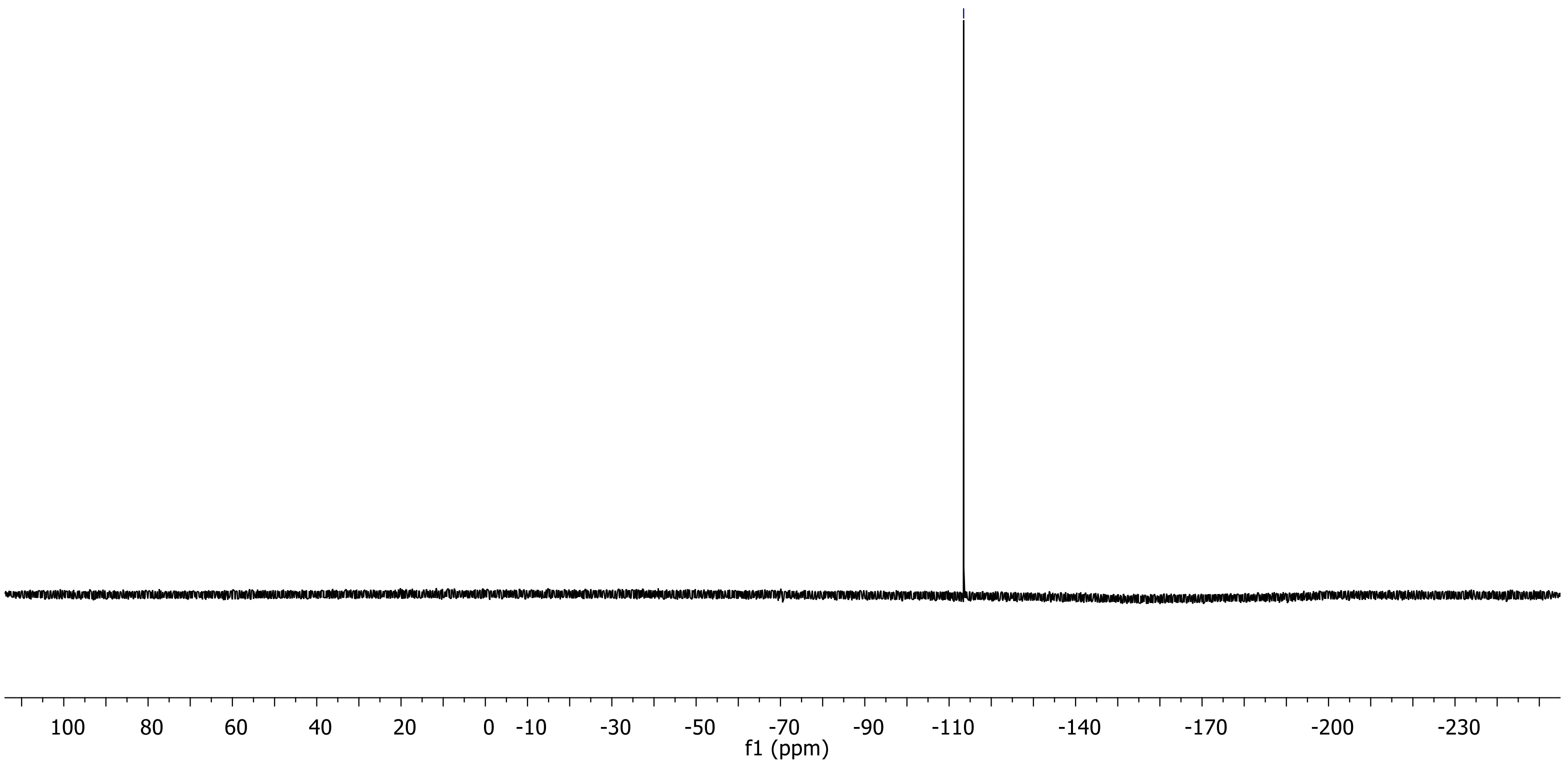

-113.45
100
80
60
40
20
0
-10
-30
-50
-70
-90
-110
-140
-170
-200
-230
f1 (ppm)

Compound 10b

^{1}H NMR (500 MHz, DMSO-d_6)

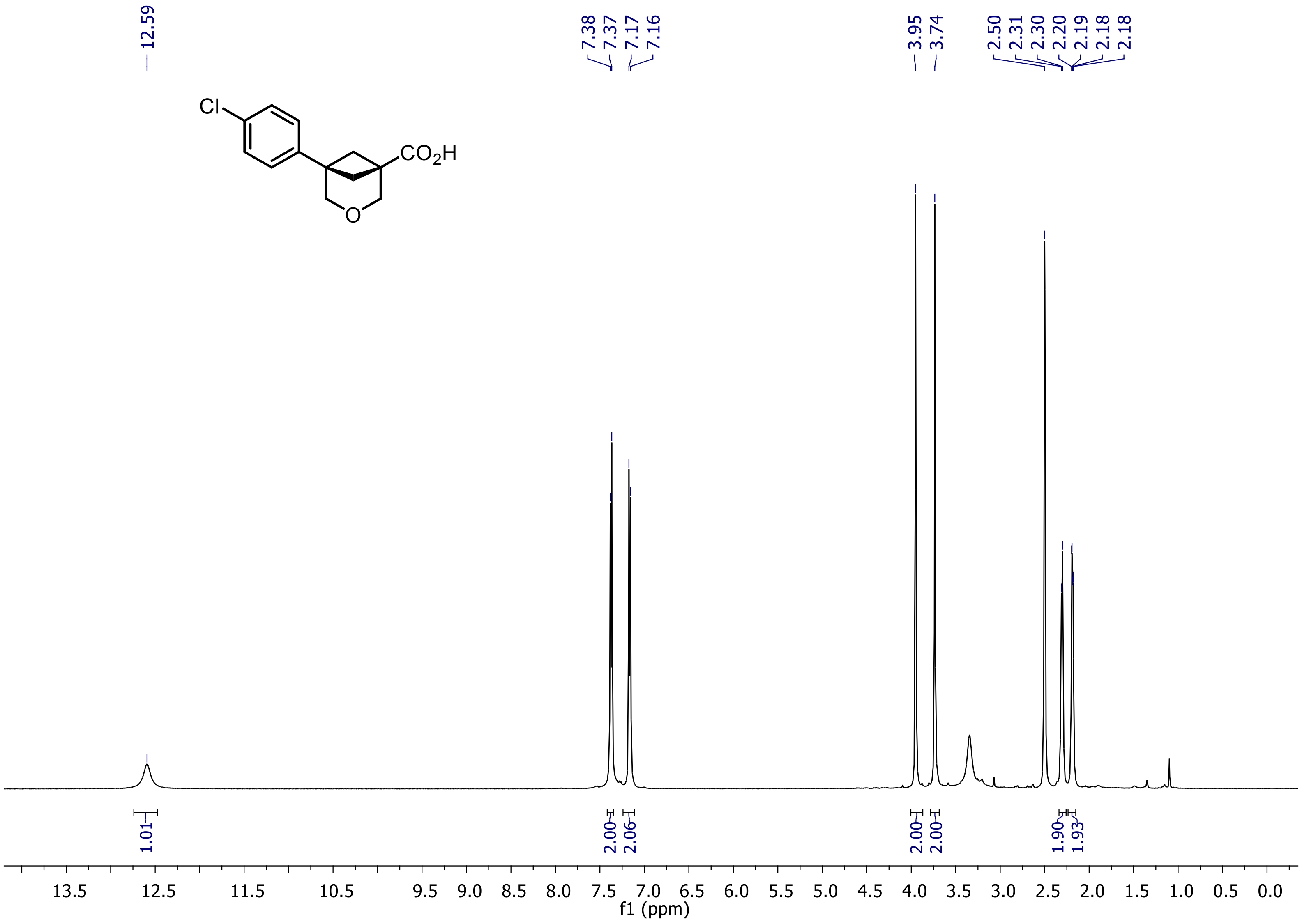

$^{13}C\{^{1}H\}$ NMR (126 MHz, DMSO-d_6)

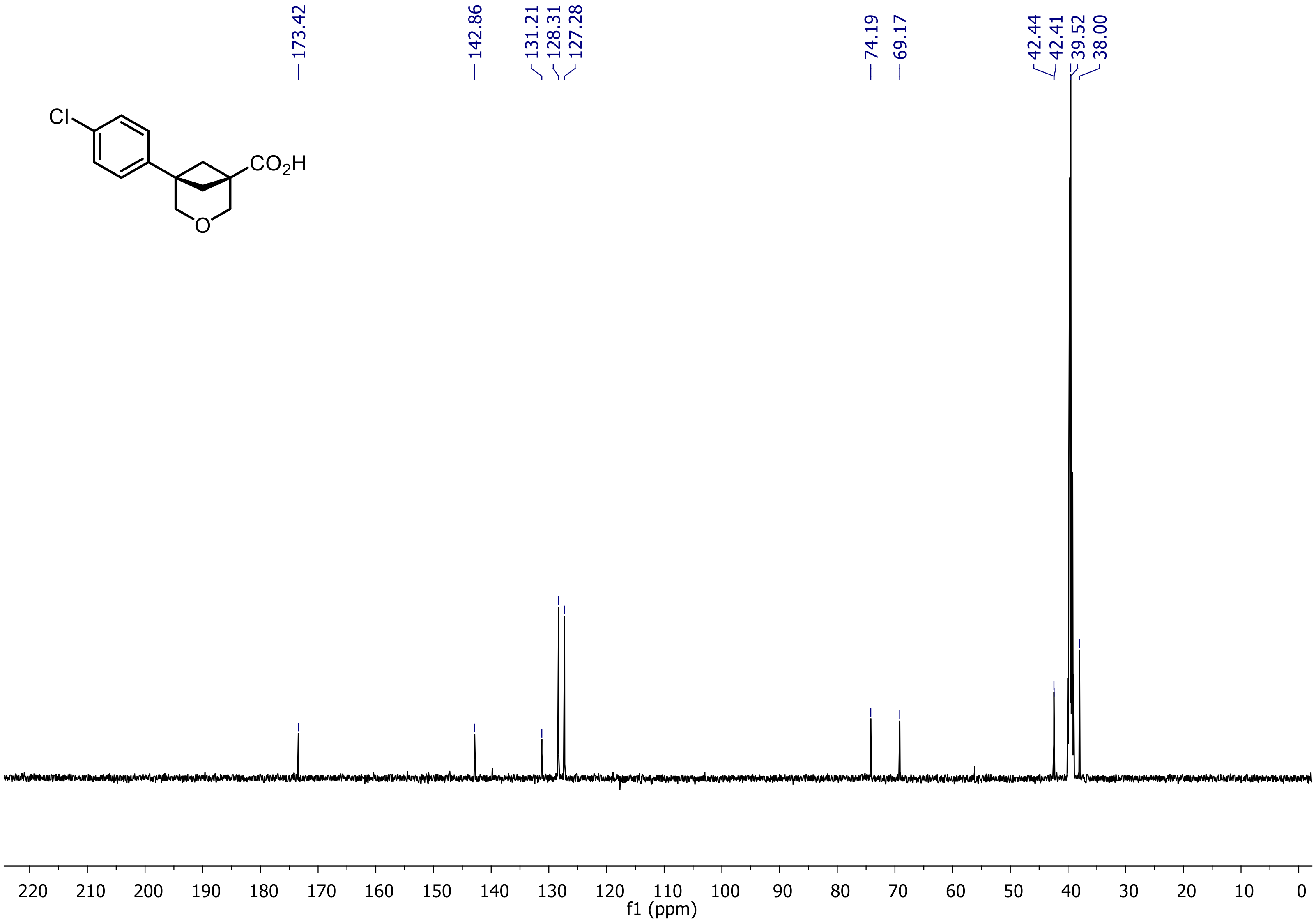

Compound 11b

^{1}H NMR (500 MHz, DMSO-d_6)

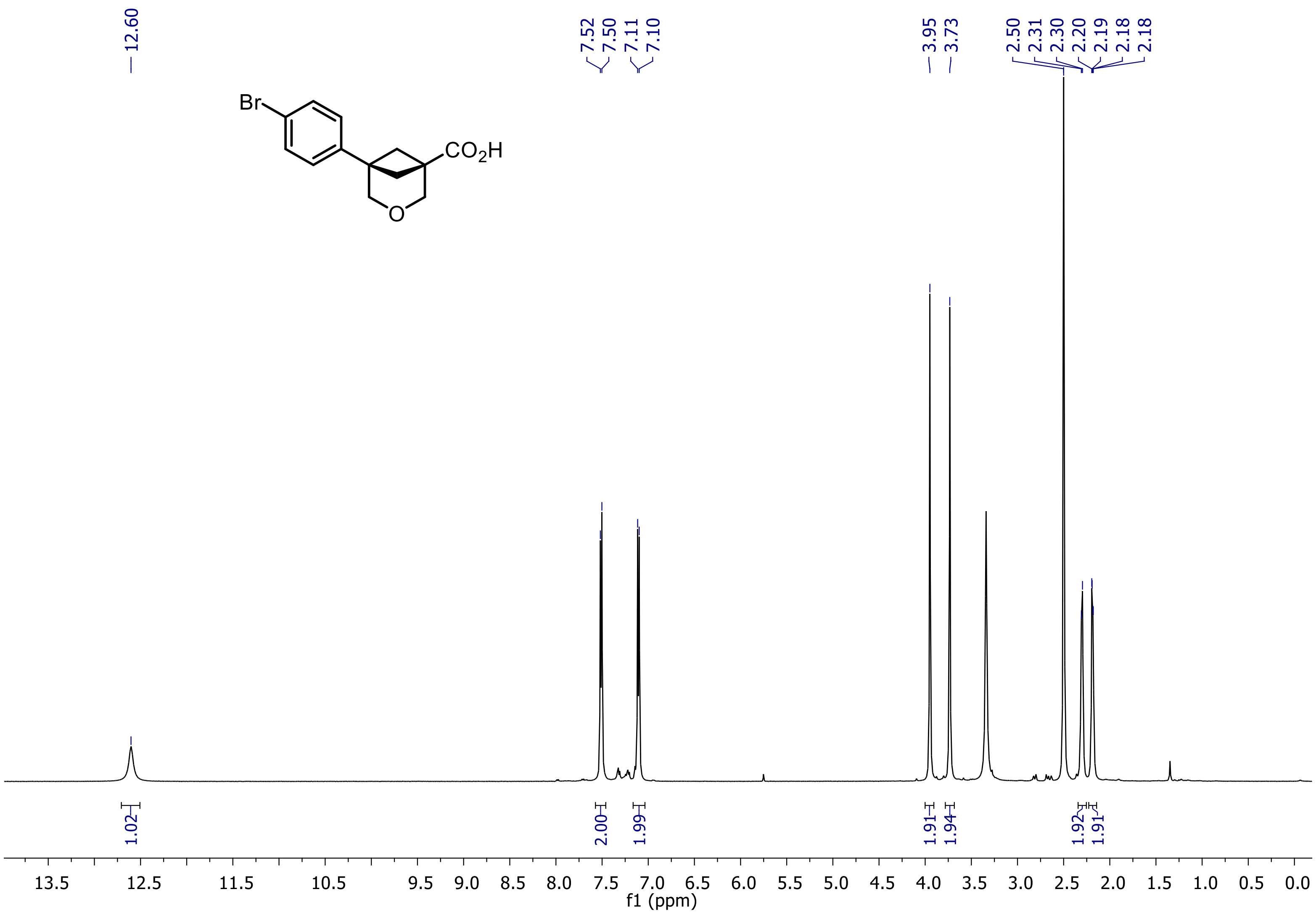

$^{13}C\{^{1}H\}$ NMR (126 MHz, DMSO-d_6)

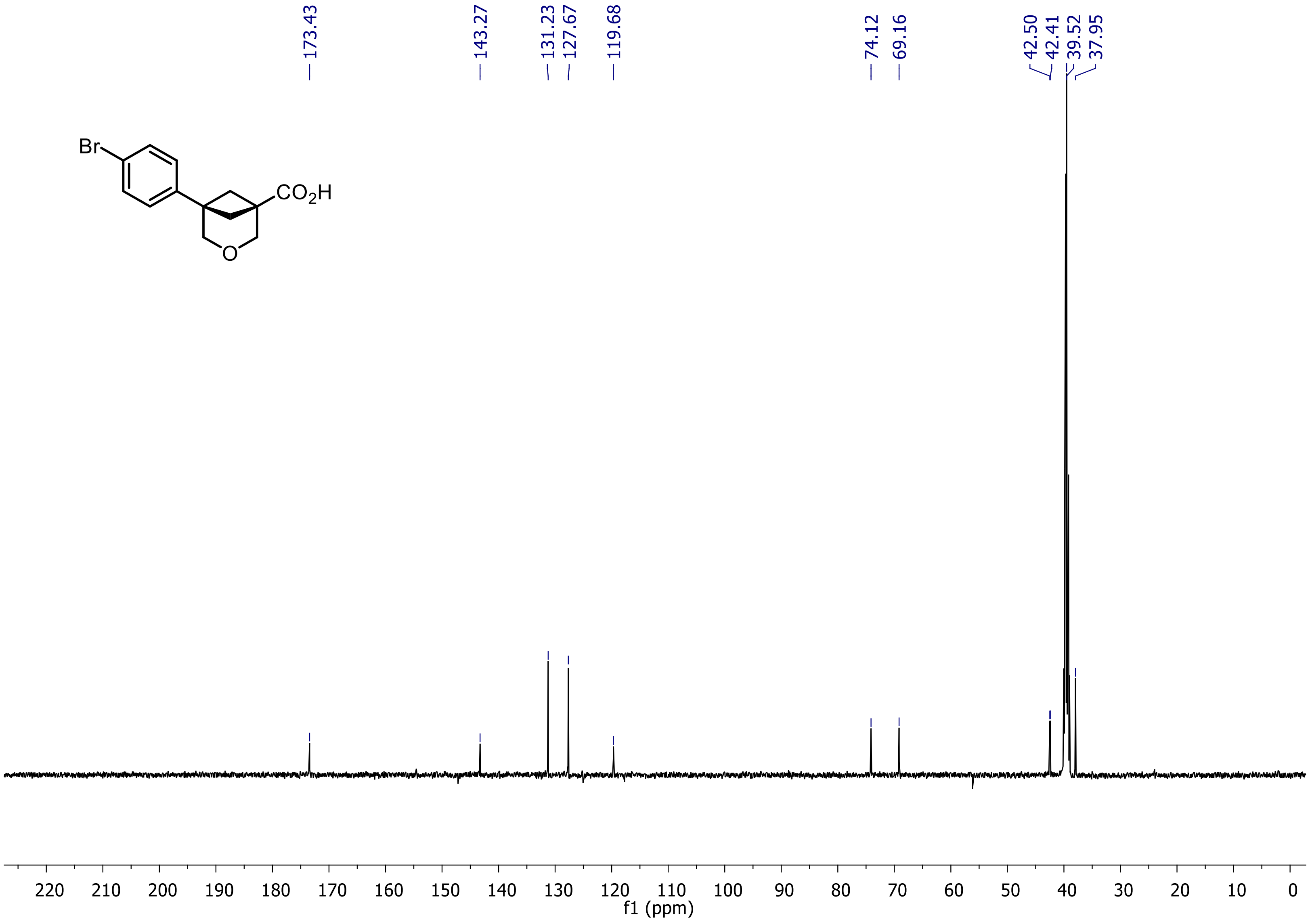
Br
CO_2H
O
173.43
143.27
131.23
127.67
119.68
74.12
69.16
42.50
42.41
39.52
37.95
220 210 200 190 180 170 160 150 140 130 120 110 100 90 80 70 60 50 40 30 20 10 0
f1 (ppm)

Compound 12b

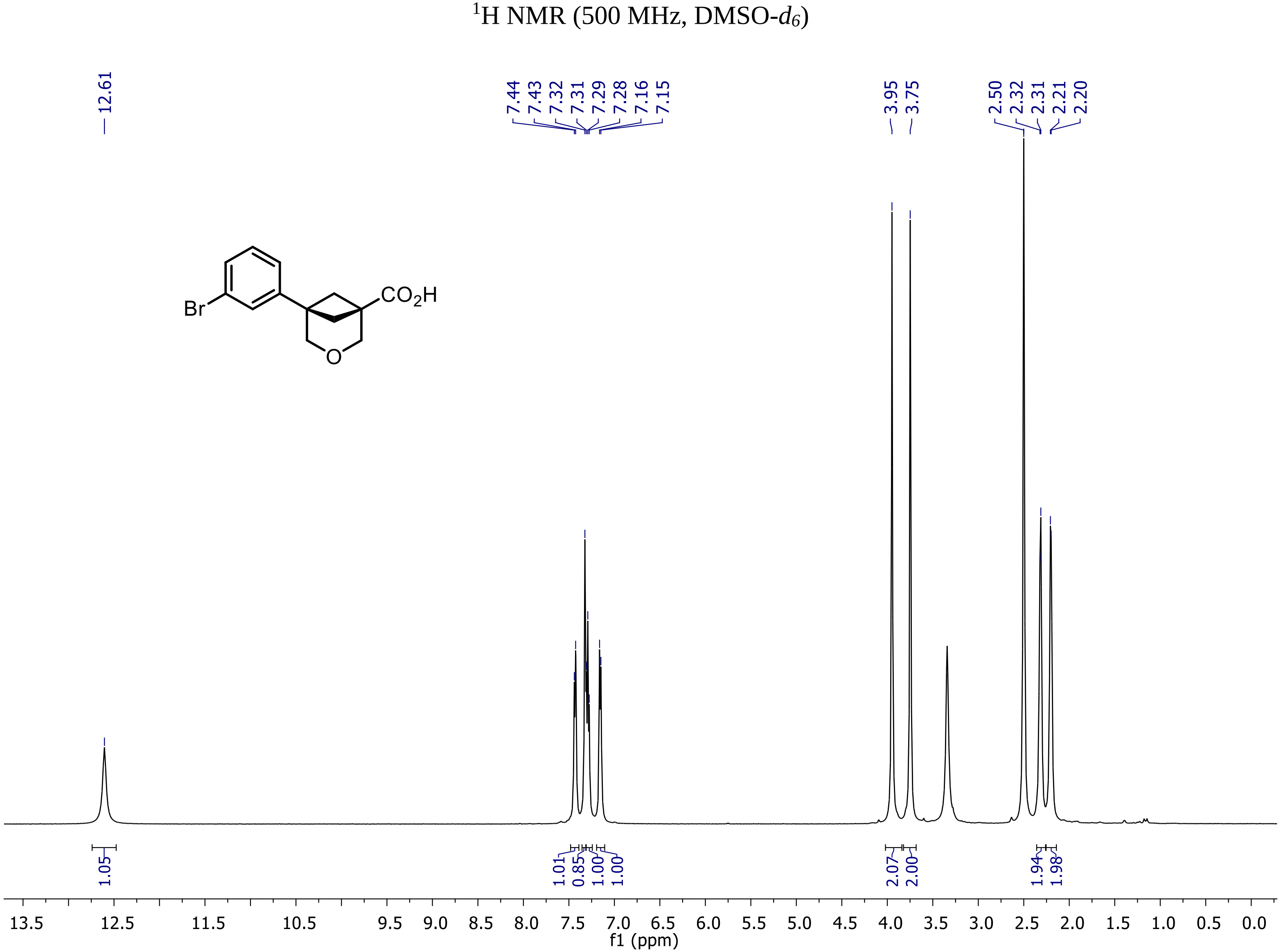

$^{13}C\{^{1}H\}$ NMR (151 MHz, DMSO-d_6)

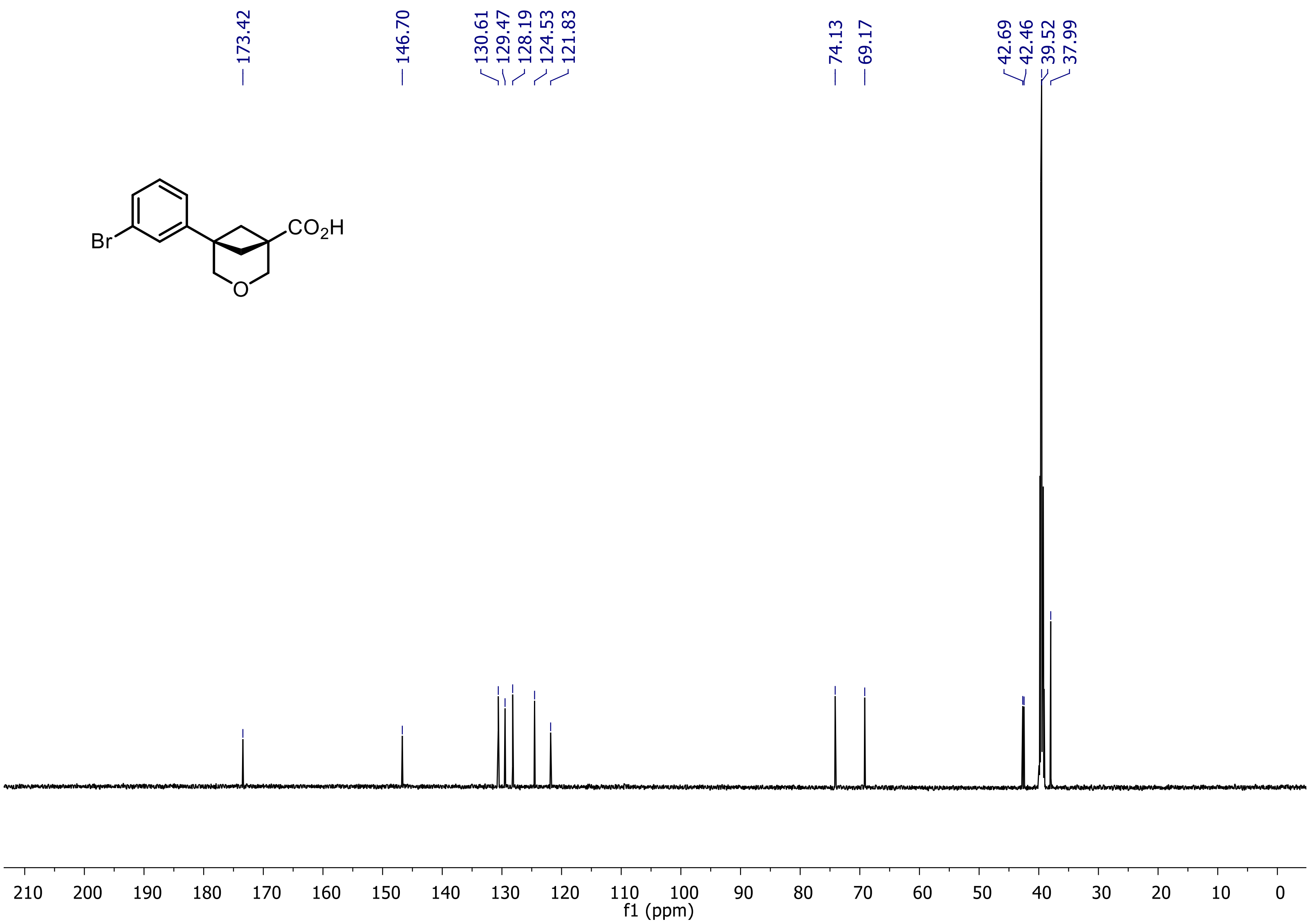

Compound 13b

^{1}H NMR (500 MHz, DMSO-d_6)

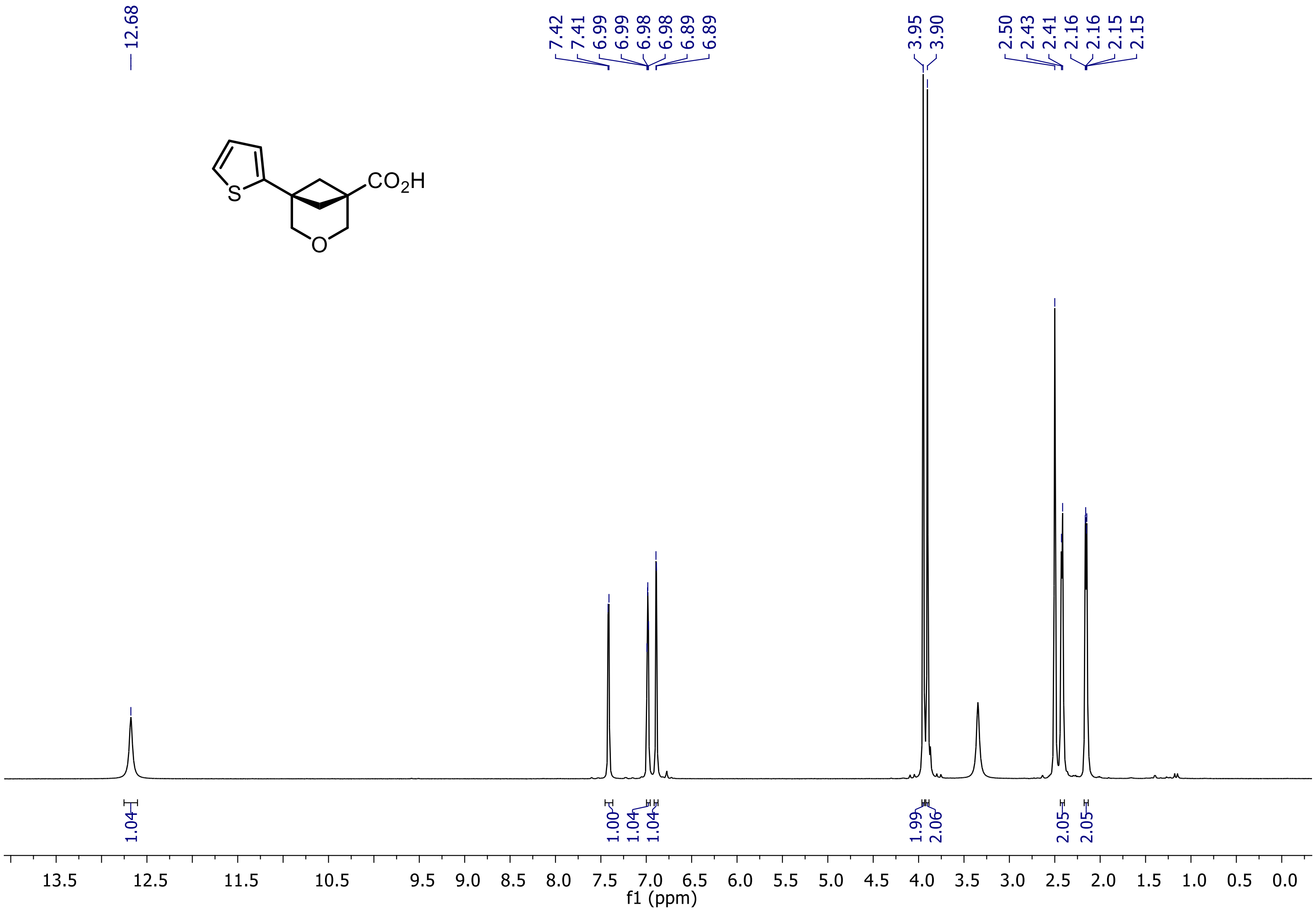

$^{13}C\{^{1}H\}$ NMR (126 MHz, DMSO-d_6)

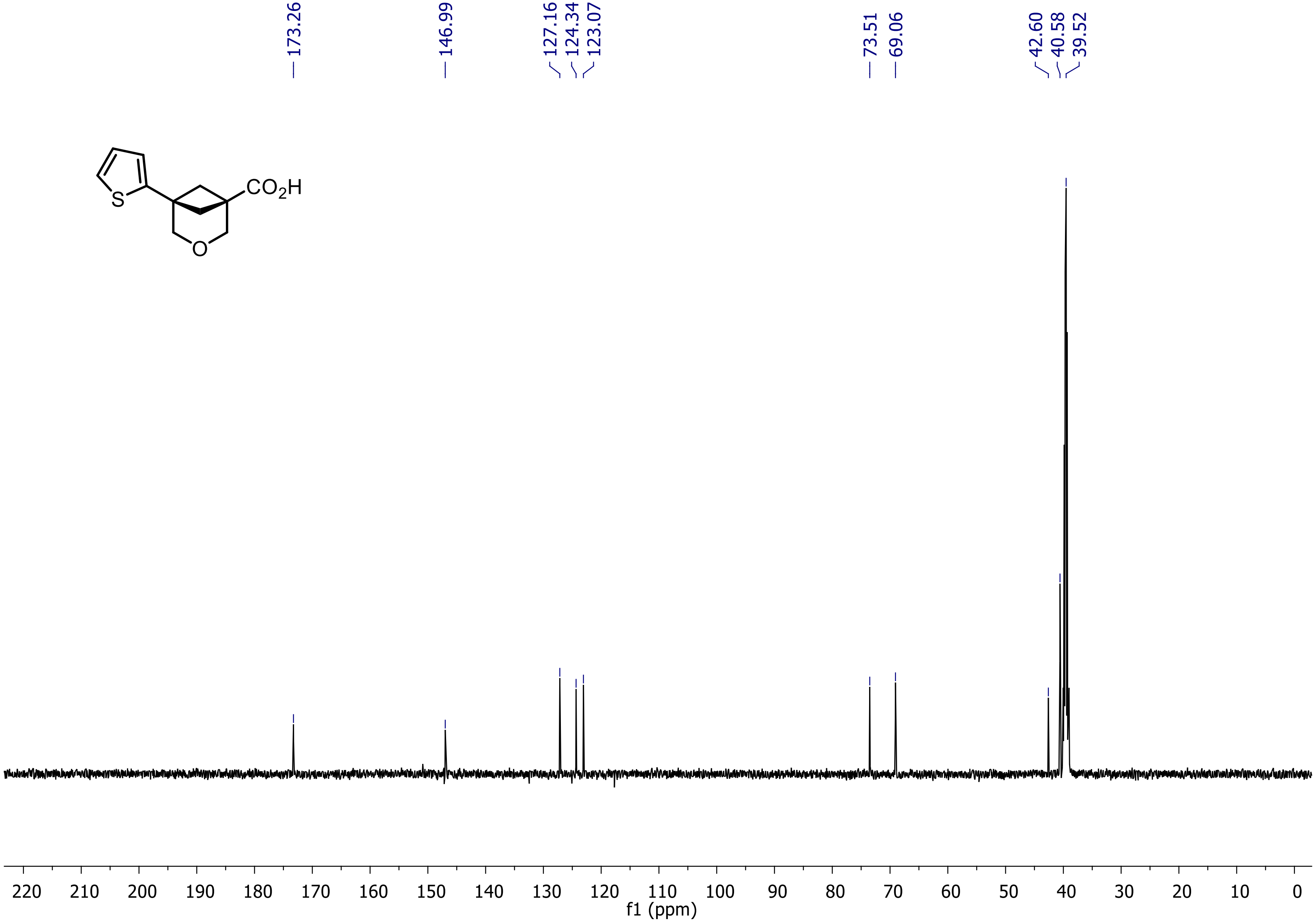

Compound 14b

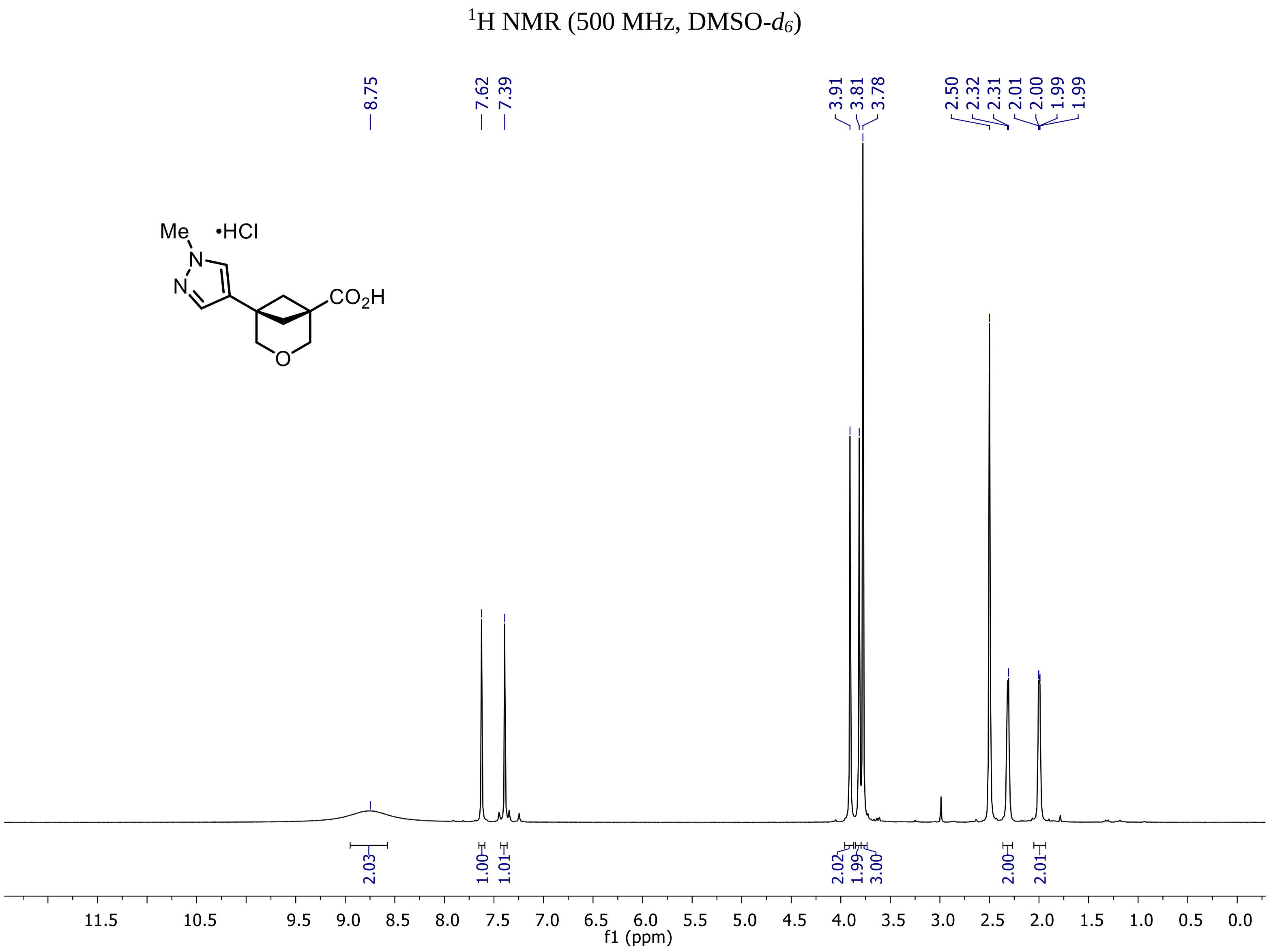

$^{13}C\{^1H\}$ NMR (151 MHz, DMSO-d_6)

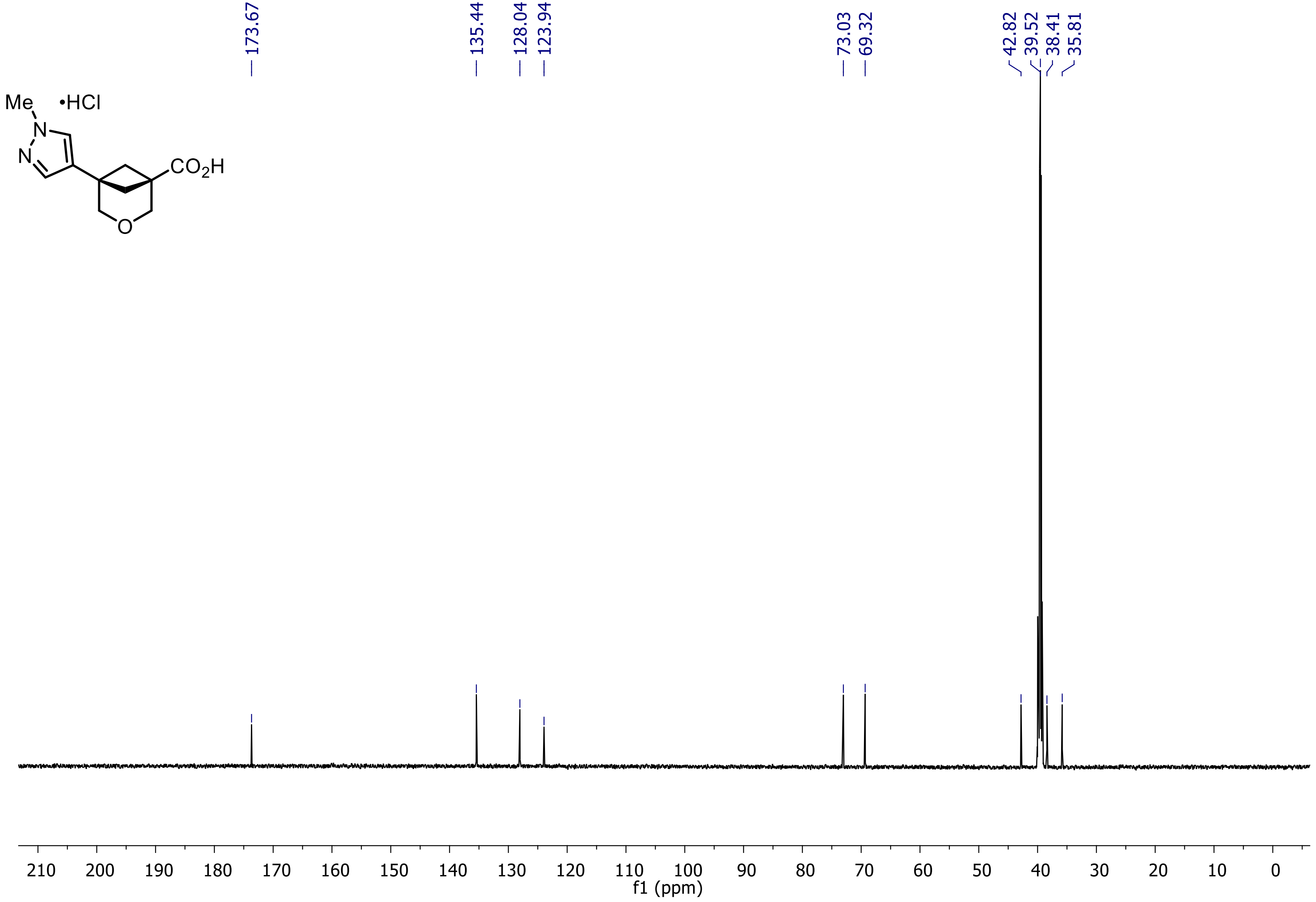

Compound 17b

^{1}H NMR (500 MHz, DMSO-d_6)

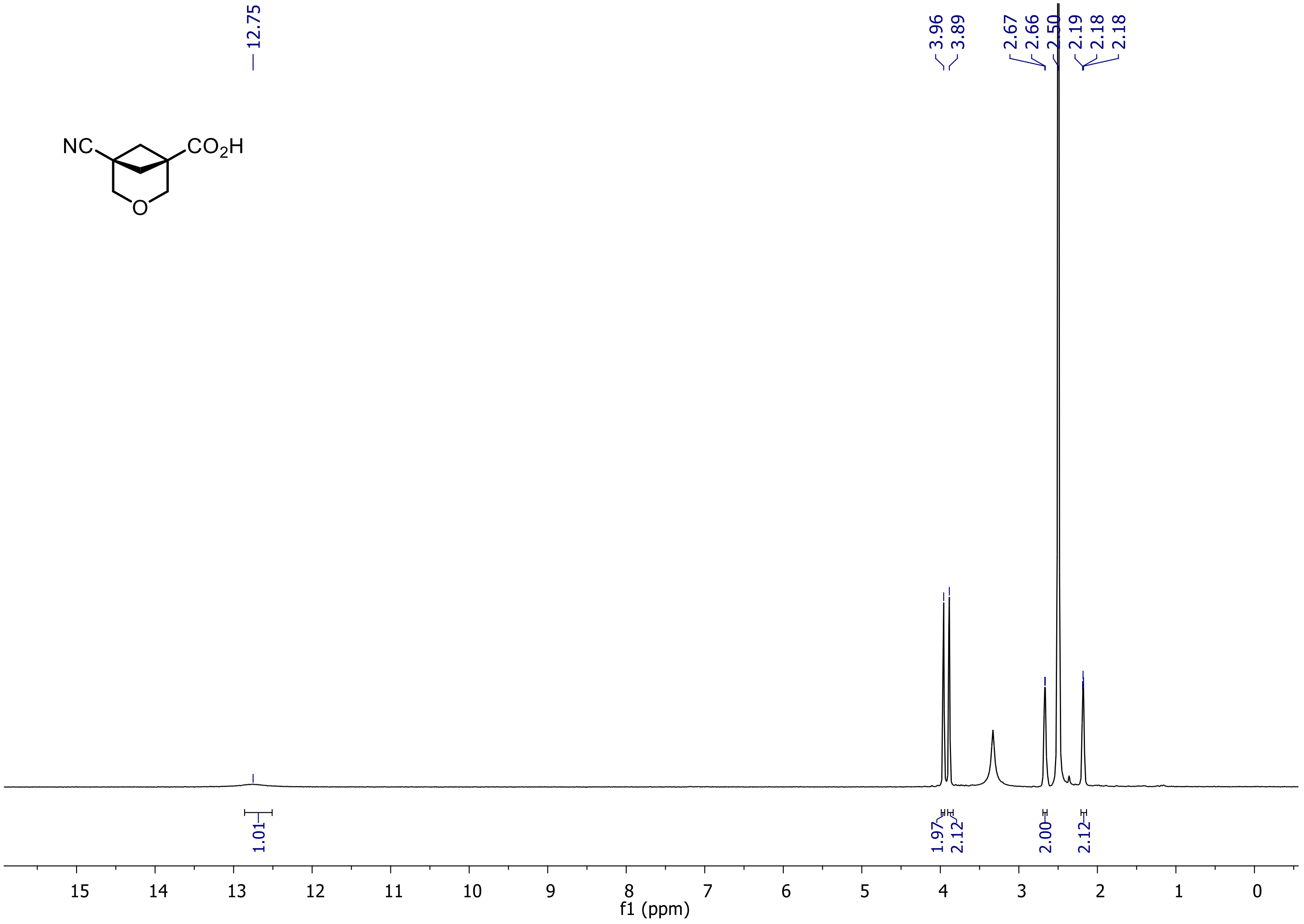

$^{13}C\{^{1}H\}$ NMR (126 MHz, DMSO-d_6)

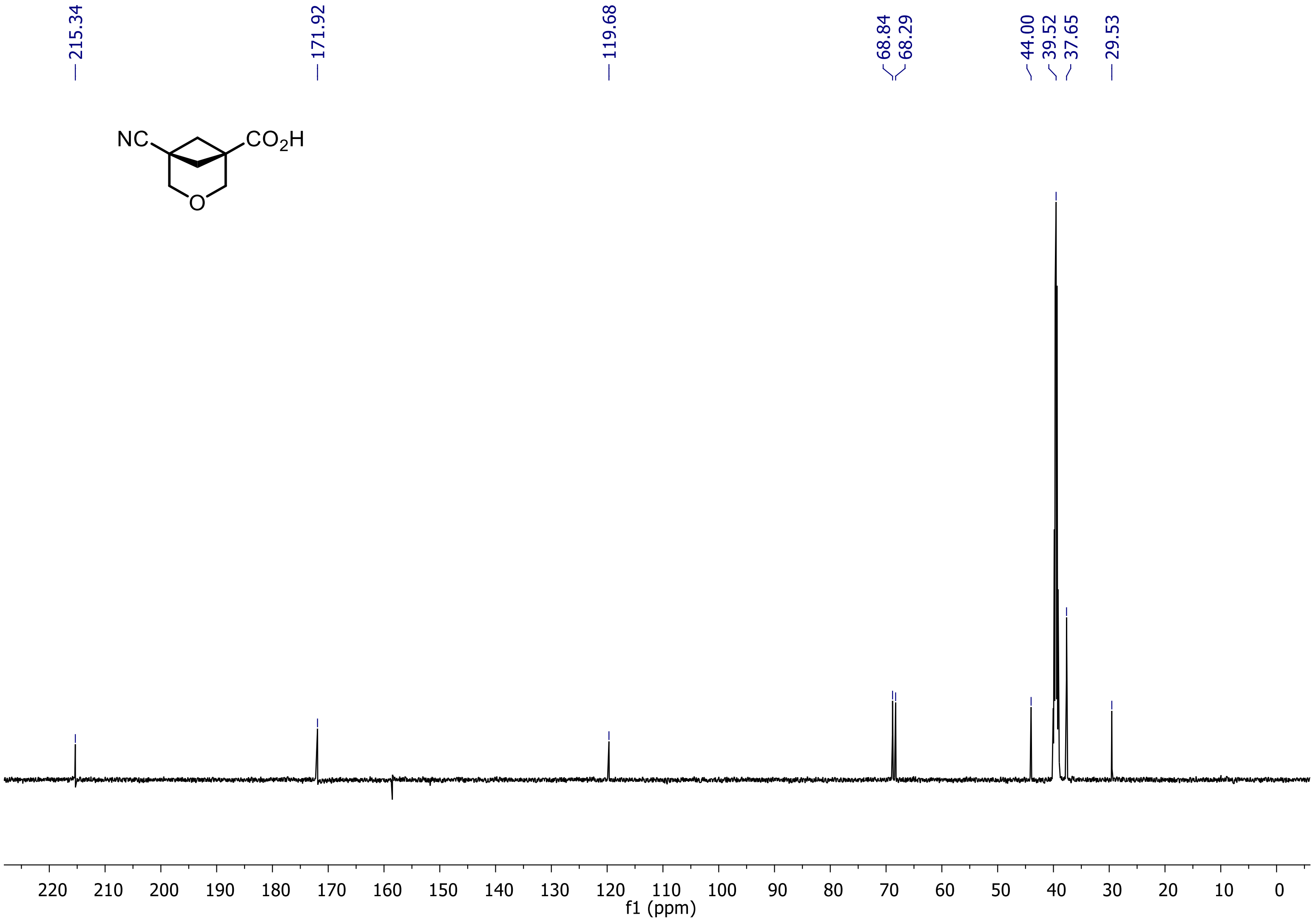

Compound 18b

^{1}H NMR (500 MHz, DMSO-d_6)

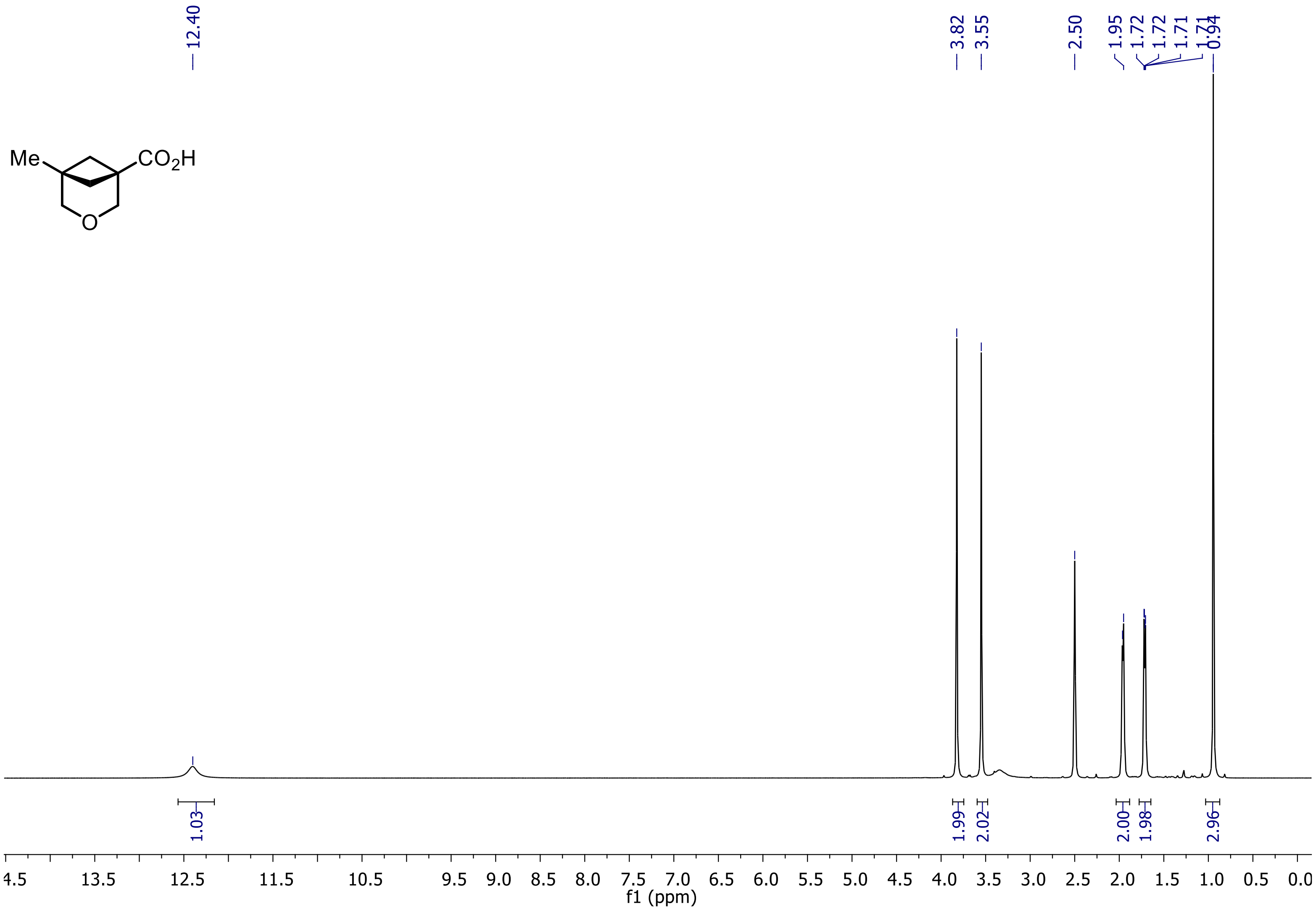

$^{13}C\{^{1}H\}$ NMR (126 MHz, DMSO-d_6)

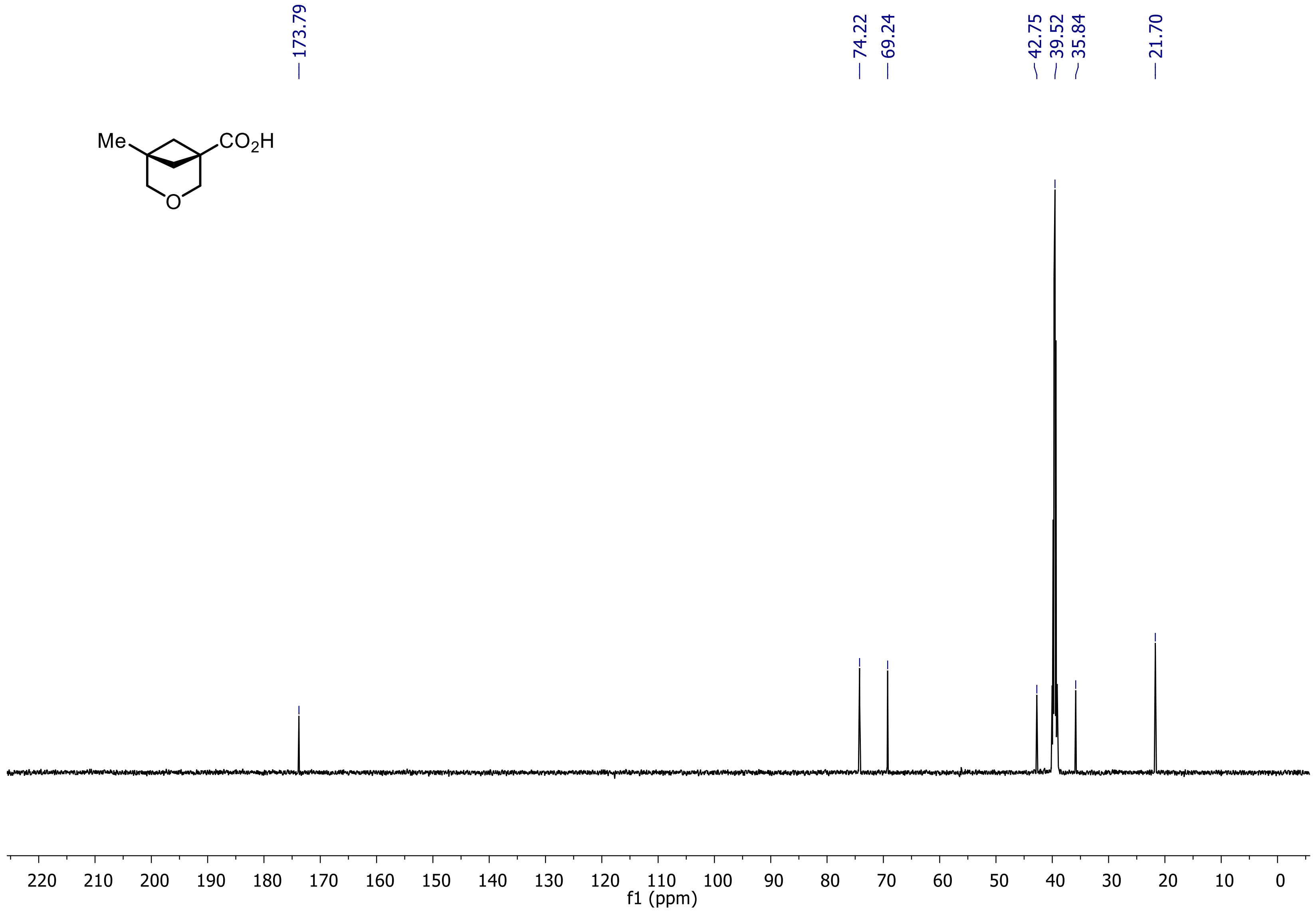

173.79
74.22
69.24
42.75
39.52
35.84
21.70
Me
CO2H
O
220 210 200 190 180 170 160 150 140 130 120 110 100 90 80 70 60 50 40 30 20 10 0
f1 (ppm)

Compound 19b

^{1}H NMR (500 MHz, DMSO-d_6)

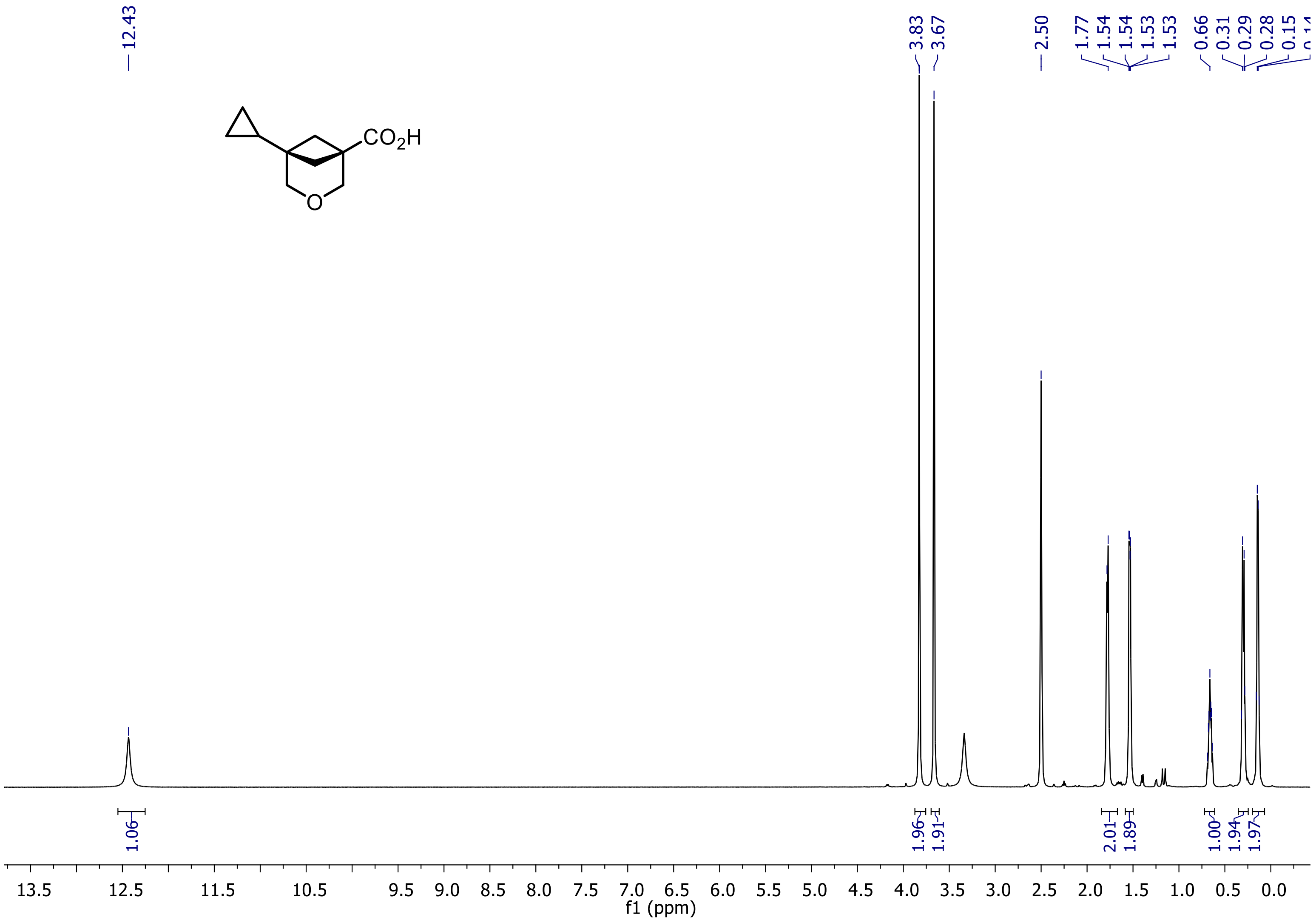

$^{13}C\{^{1}H\}$ NMR (126 MHz, DMSO-d_6)

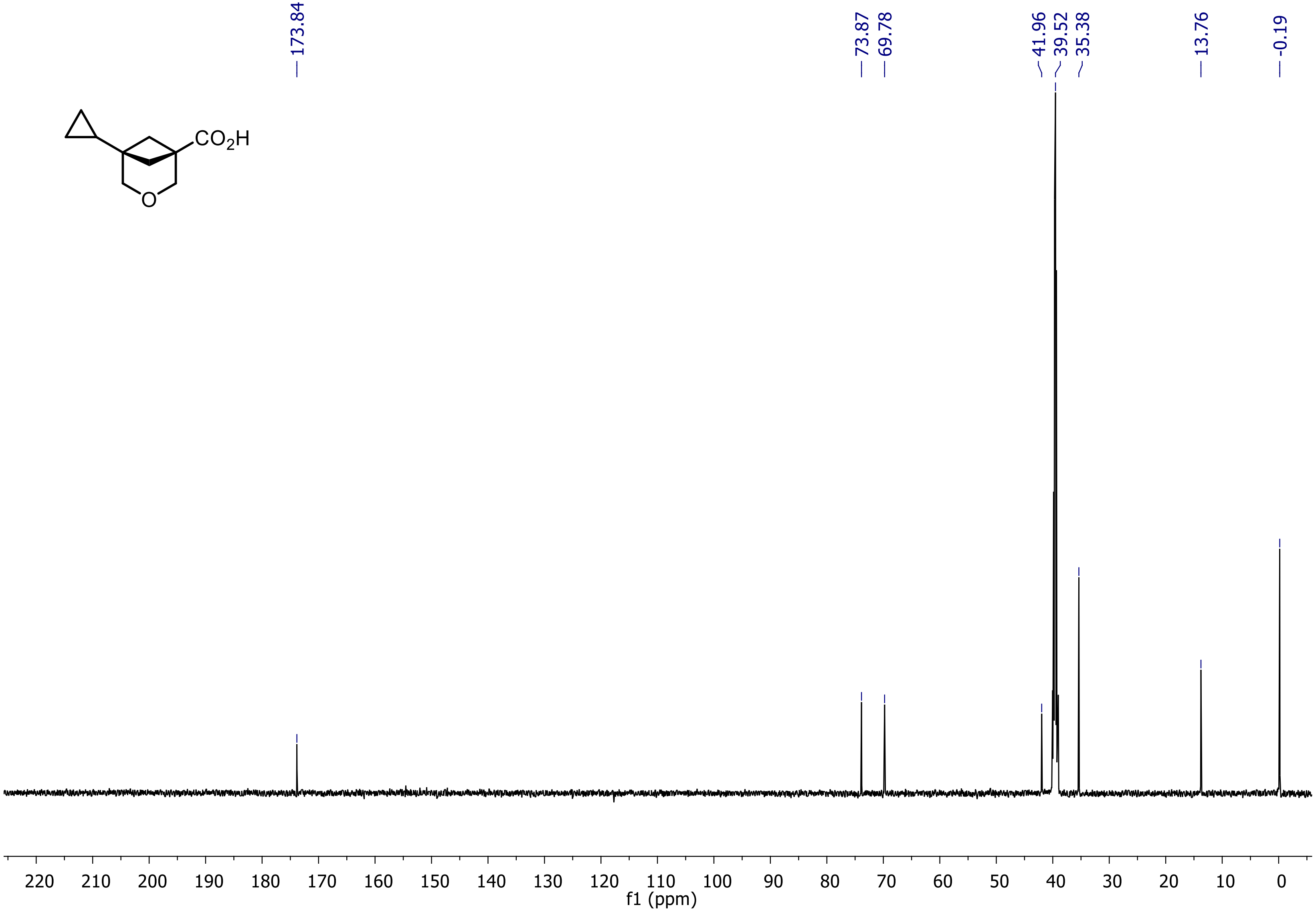

Compound 20b

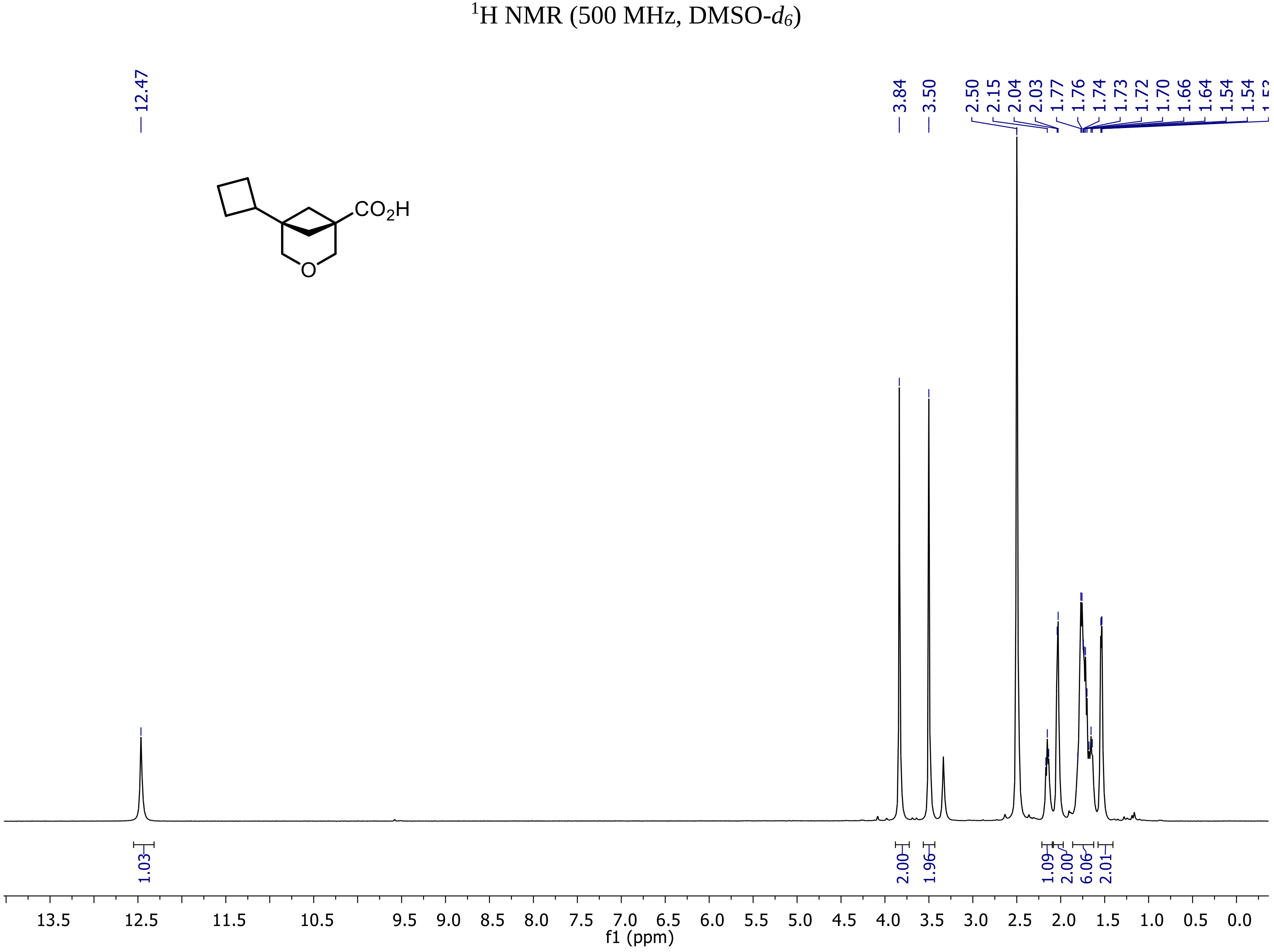

$^{13}C\{^1H\}$ NMR (126 MHz, DMSO-d_6)

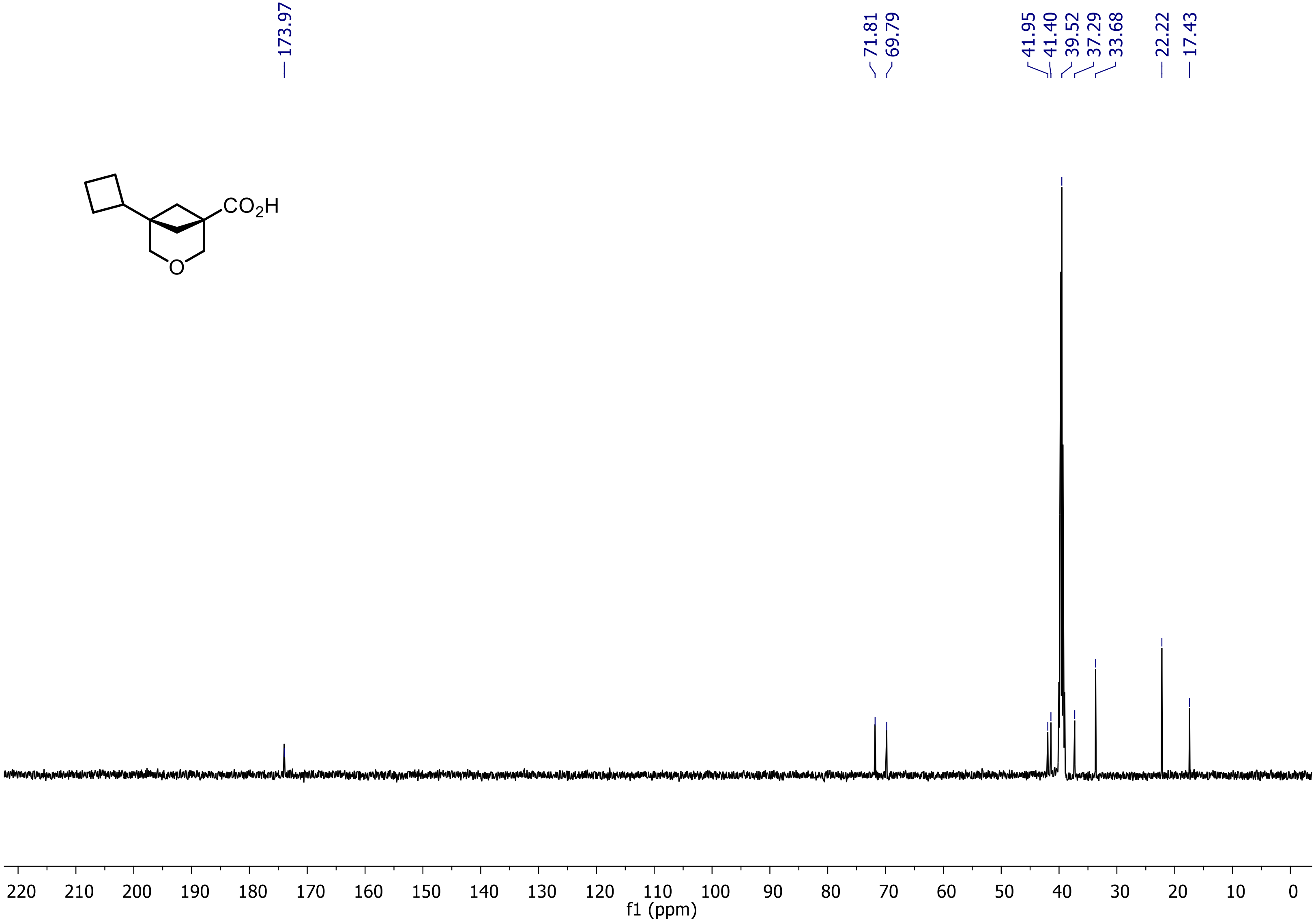

Compound 21b

^{1}H NMR (500 MHz, $CDCl_3$)

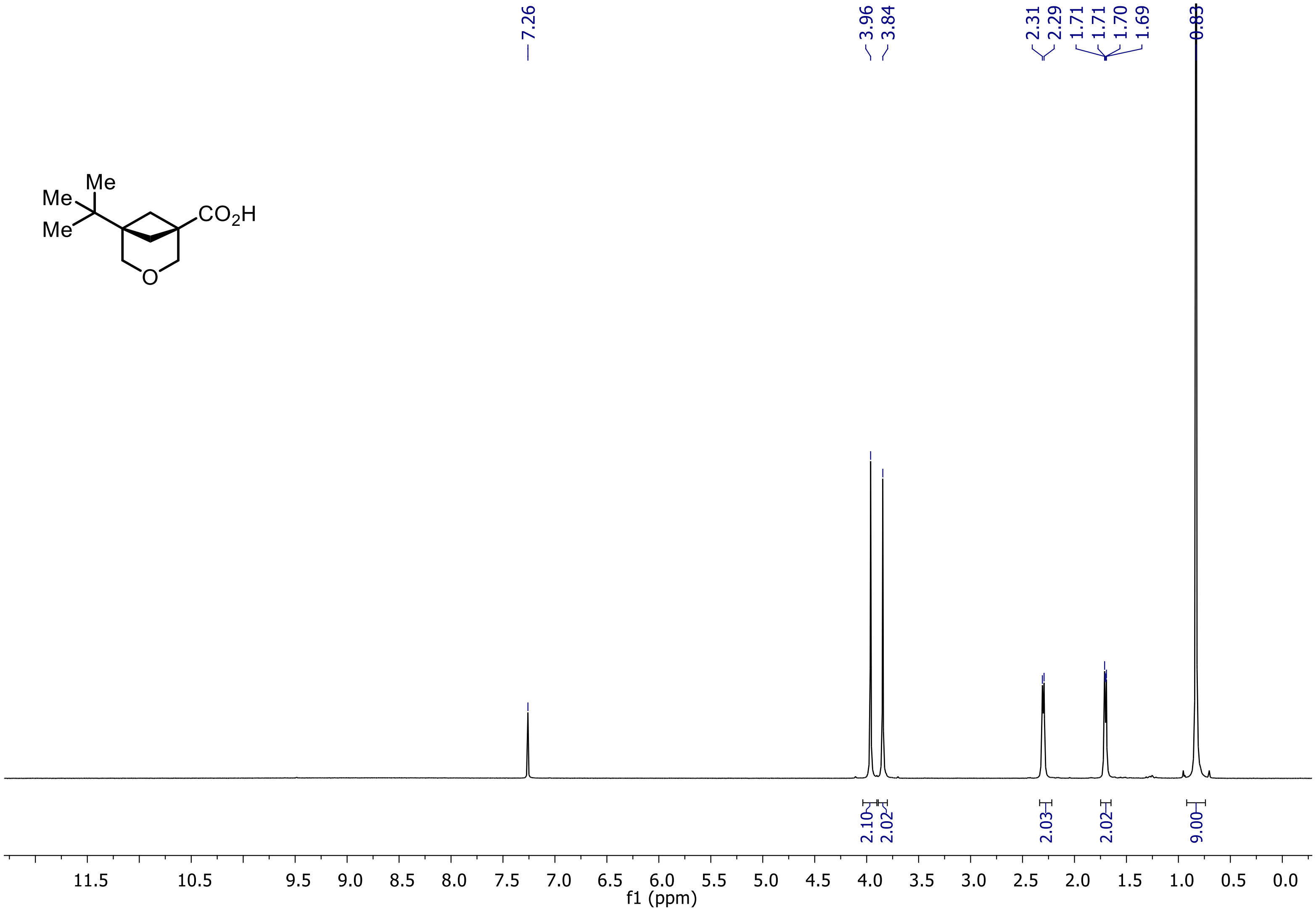

$^{13}C\{^1H\}$ NMR (151 MHz, $CDCl_3$)

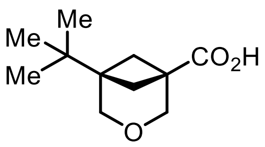

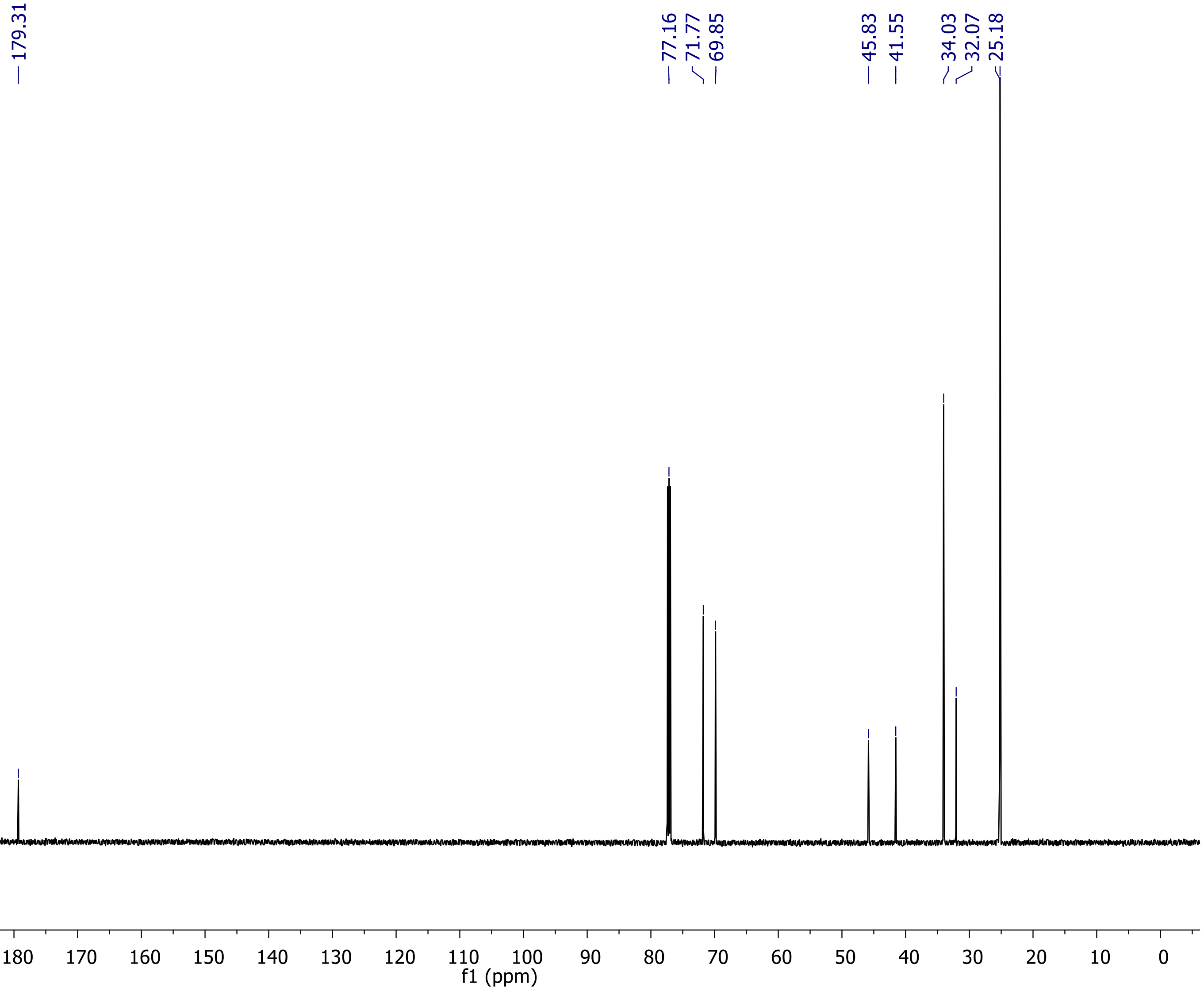

Compound 22b

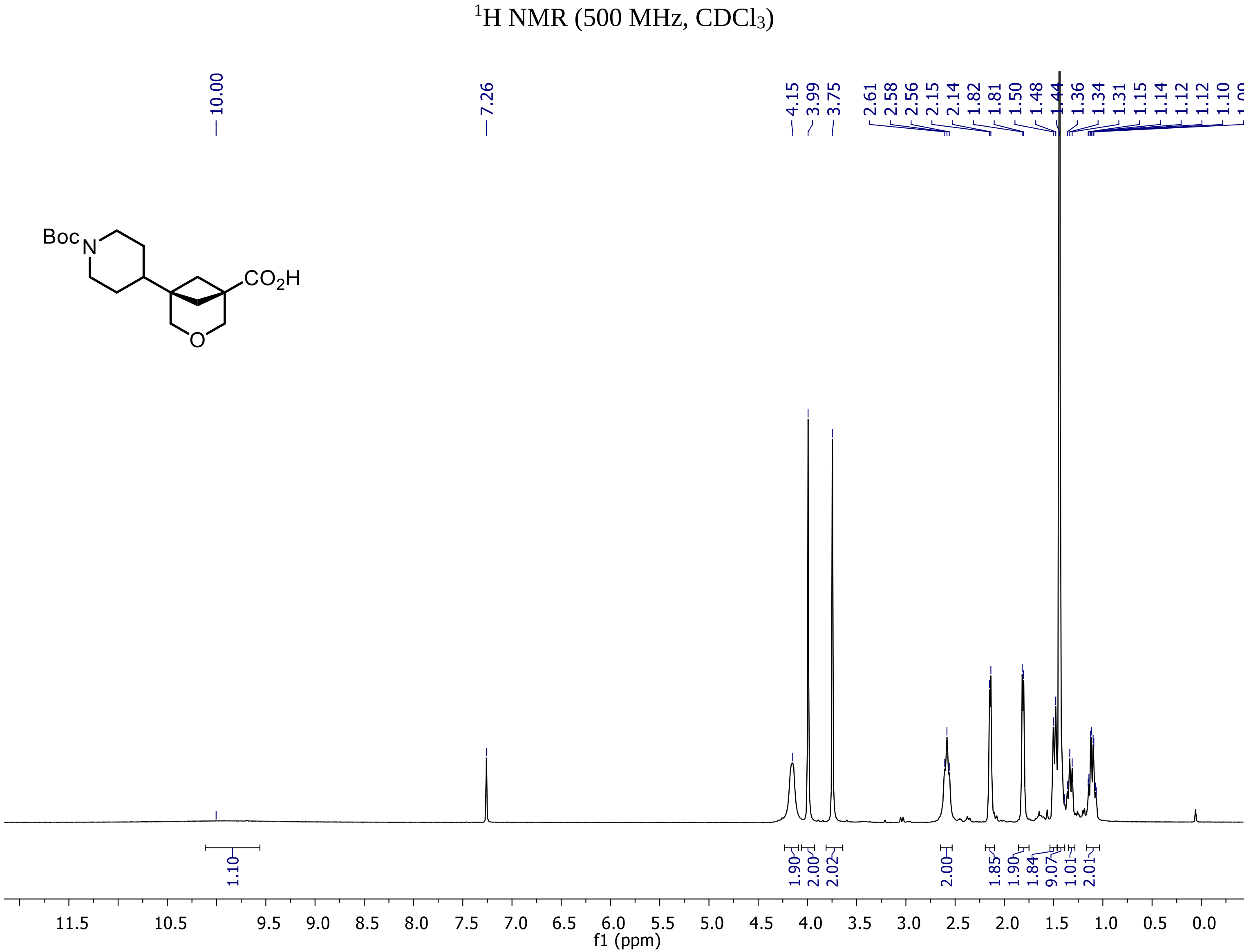

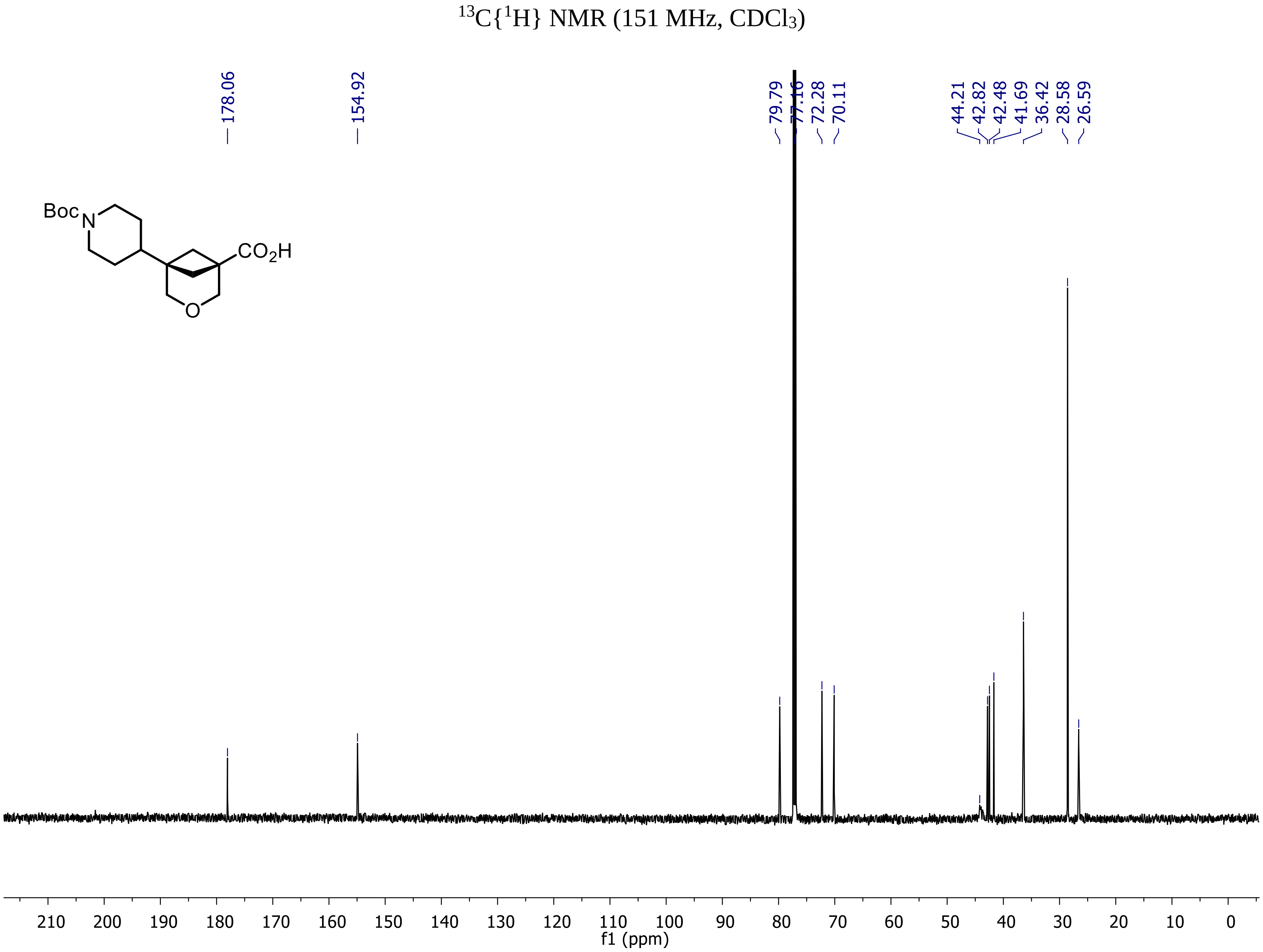

$^{13}C\{^{1}H\}$ NMR (151 MHz, $CDCl_3$)
178.06
154.92
79.79
77.16
72.28
70.11
44.21
42.82
42.48
41.69
36.42
28.58
26.59
Boc
N
CO_2H
O
210 200 190 180 170 160 150 140 130 120 110 100 90 80 70 60 50 40 30 20 10 0
f1 (ppm)

Compound 23

^{1}H NMR (500 MHz, $CDCl_3$)

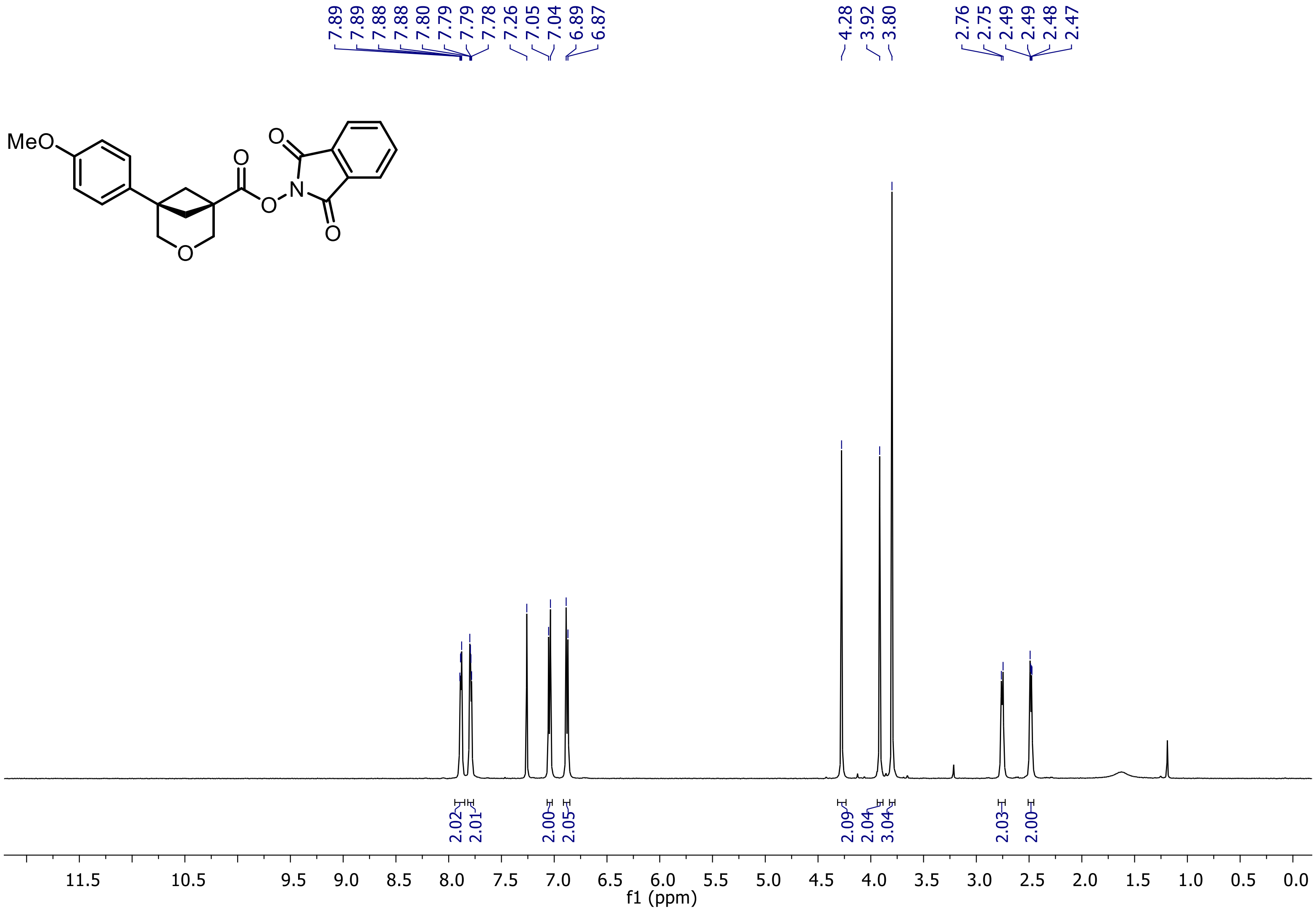

$^{13}C\{^{1}H\}$ NMR (126 MHz, $CDCl_3$)

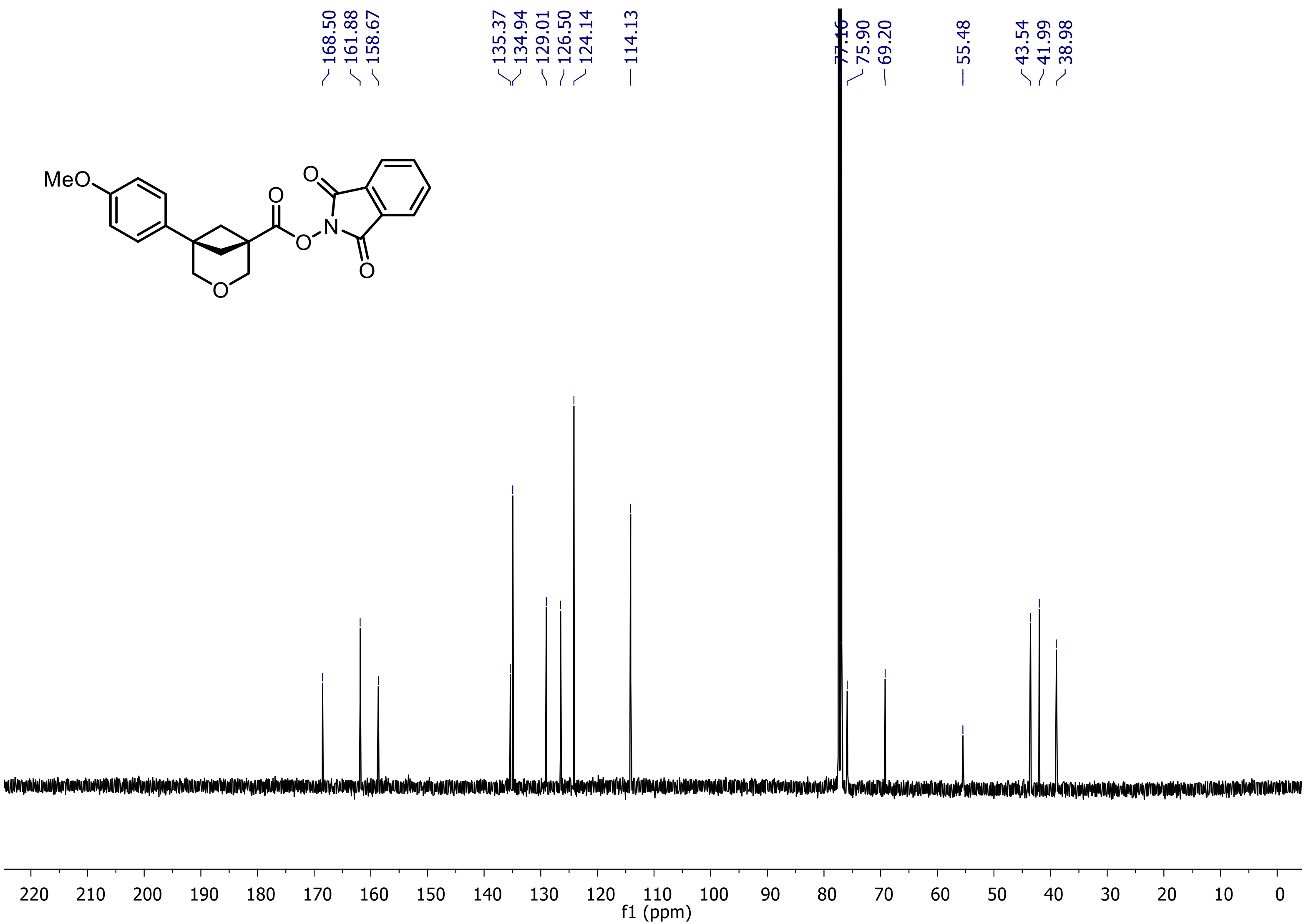

Compound 23a

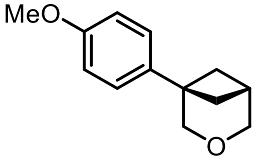

^{1}H NMR (500 MHz, $CDCl_3$)

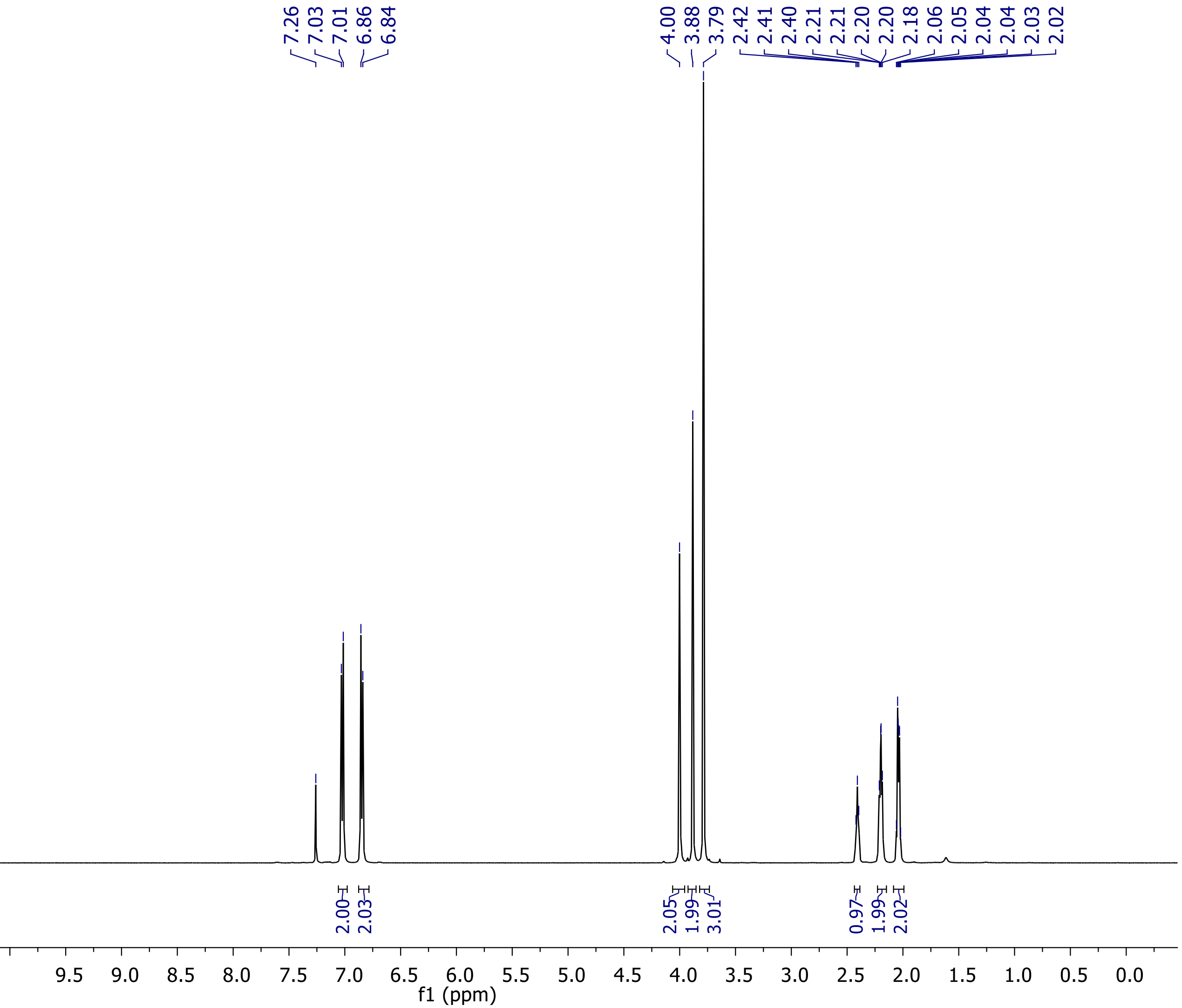

$^{13}C\{^1H\}$ NMR (151 MHz, $CDCl_3$)

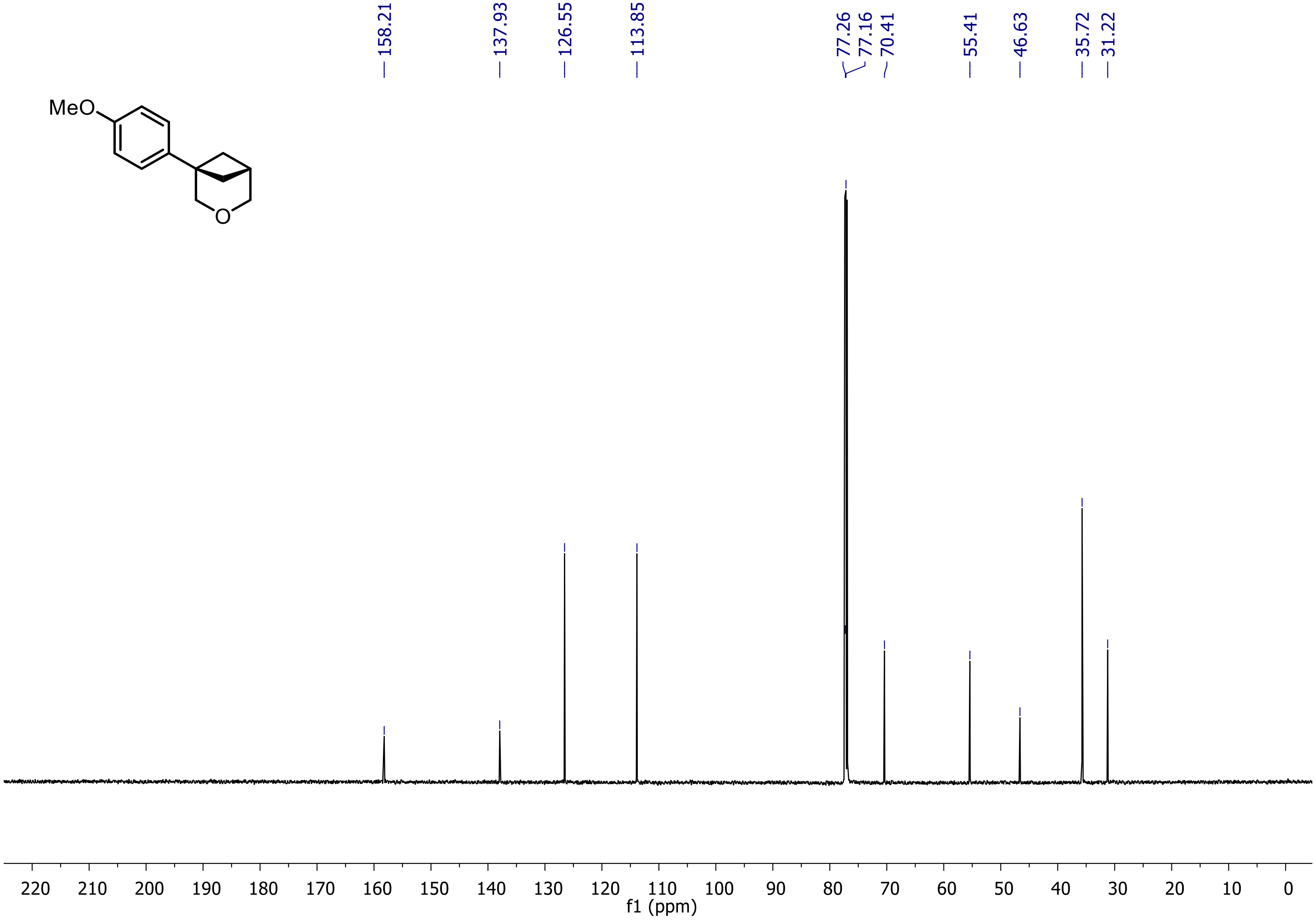

Compound 23b

^{1}H NMR (400 MHz, DMSO-d_6)

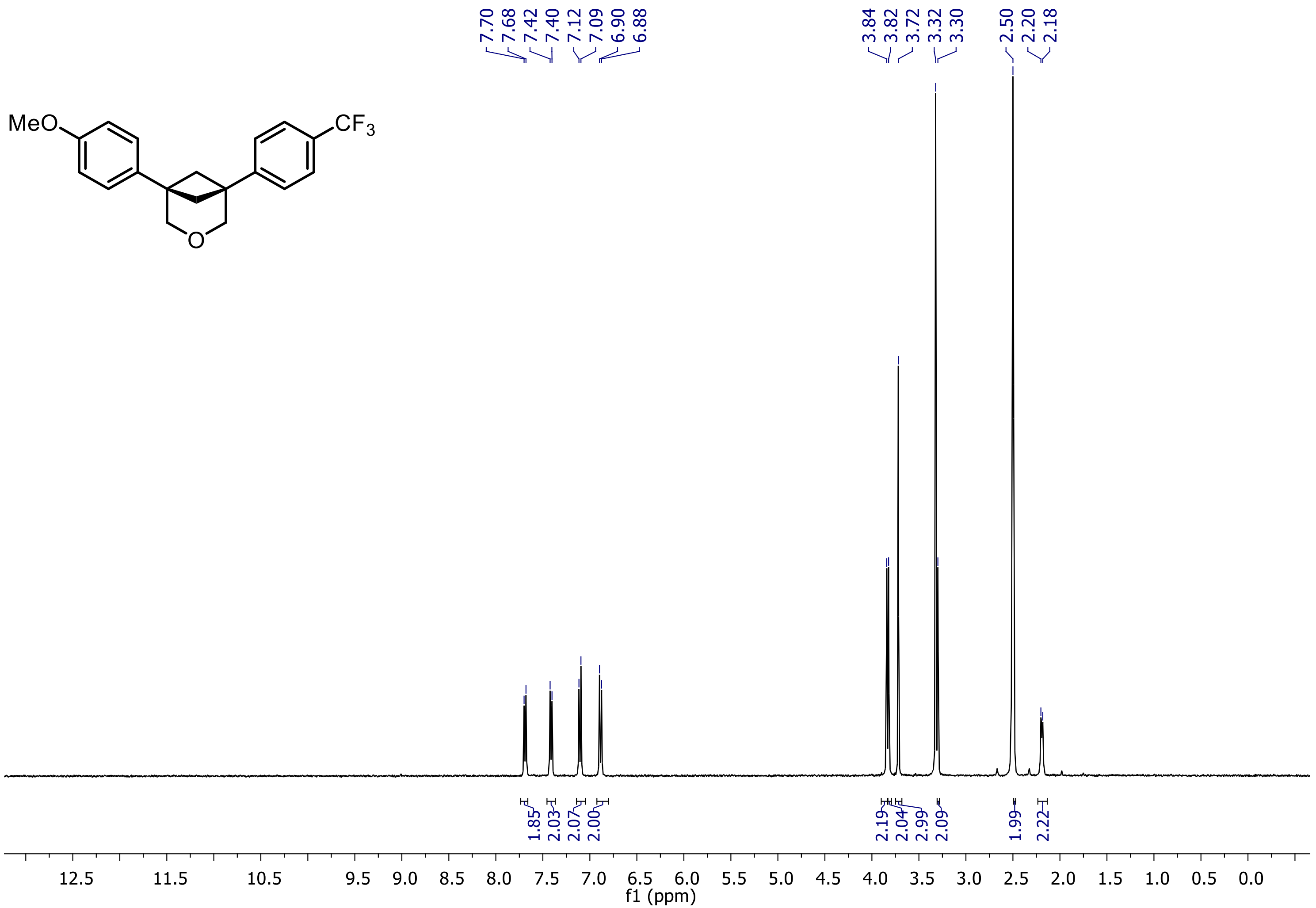

$^{13}C\{^{1}H\}$ NMR (151 MHz, DMSO-d_6)

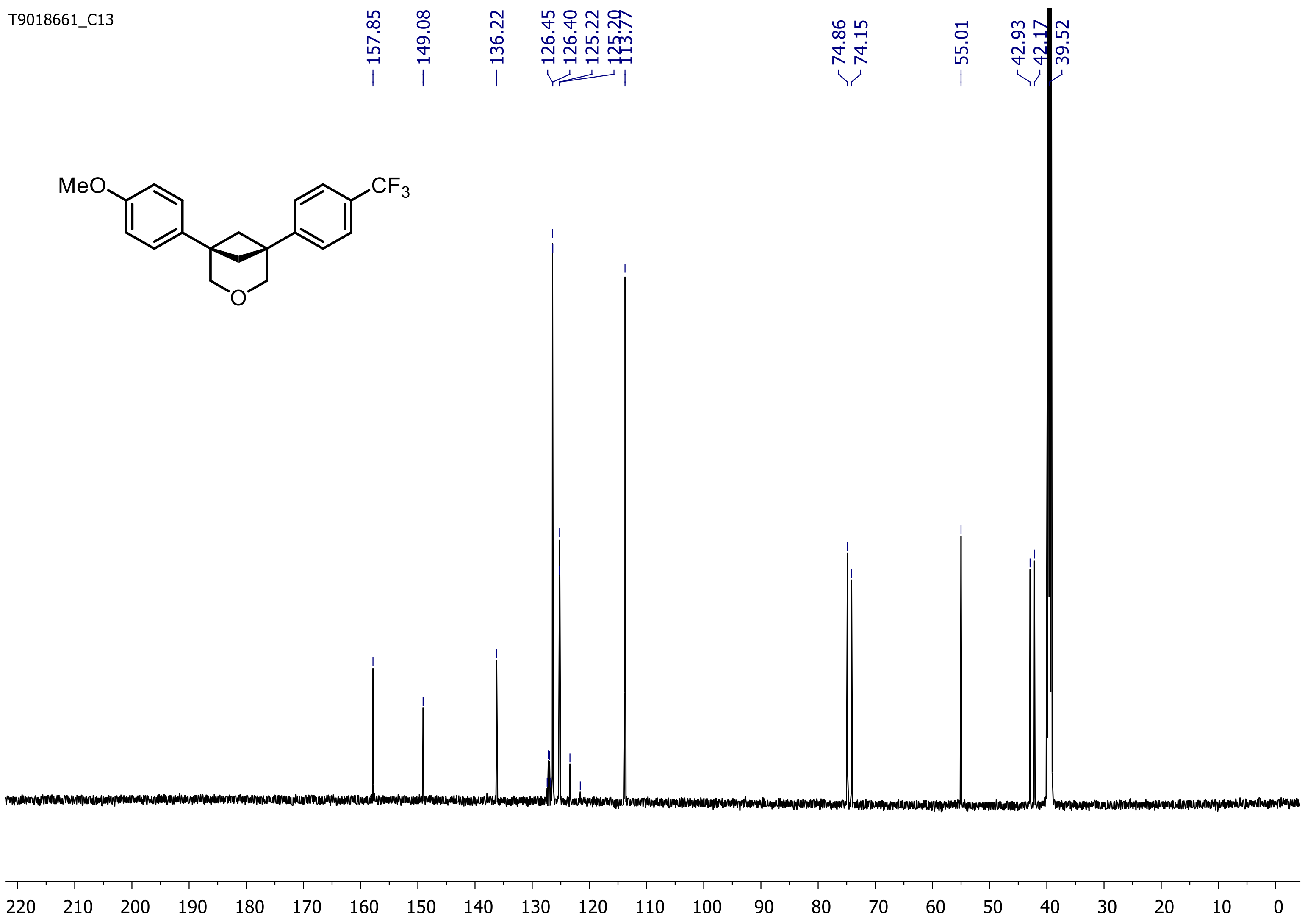

$^{19}F\{^1H\}$ NMR (376 MHz, DMSO-d_6)

T9018661_F19{H}

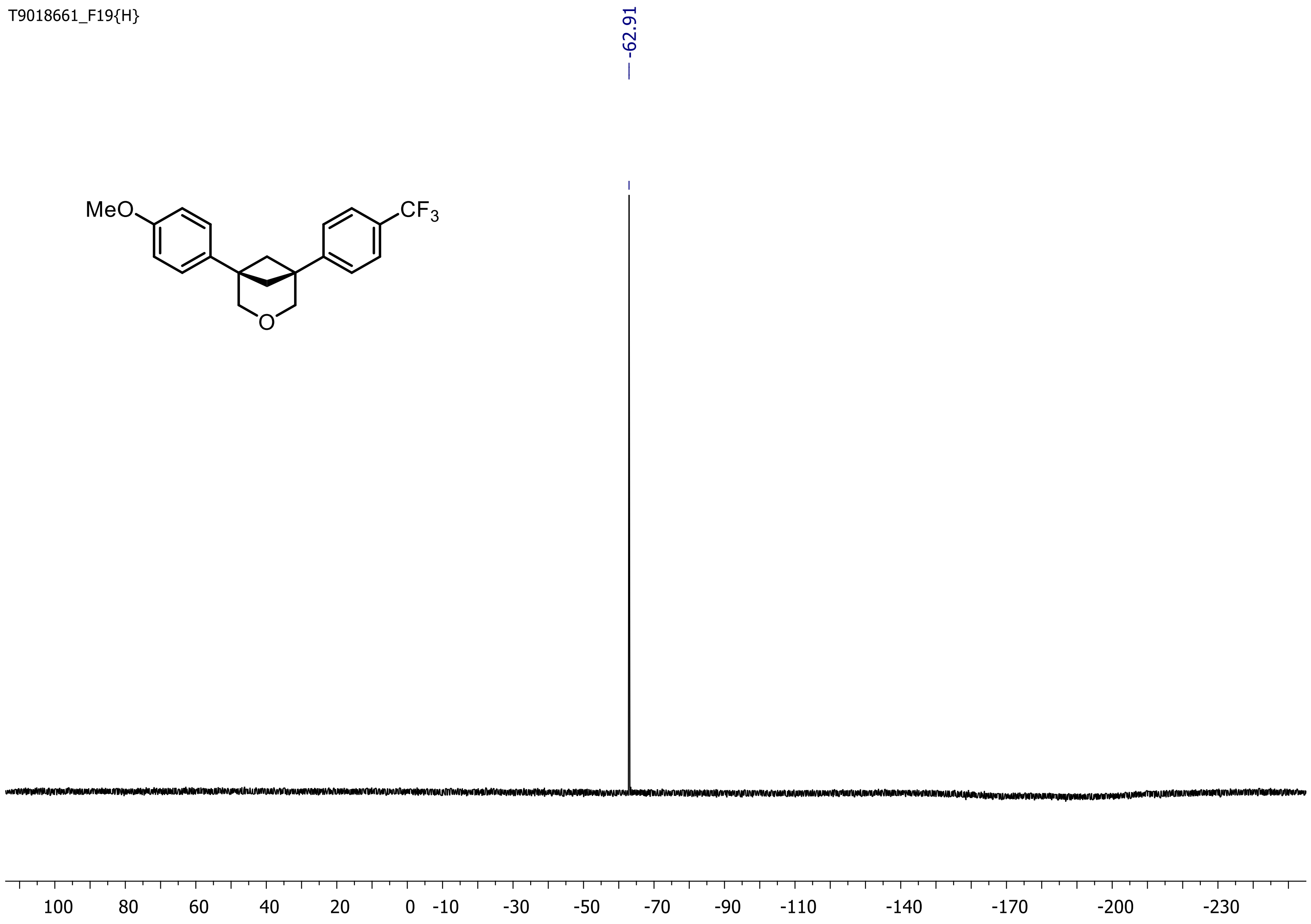

Compound 23c

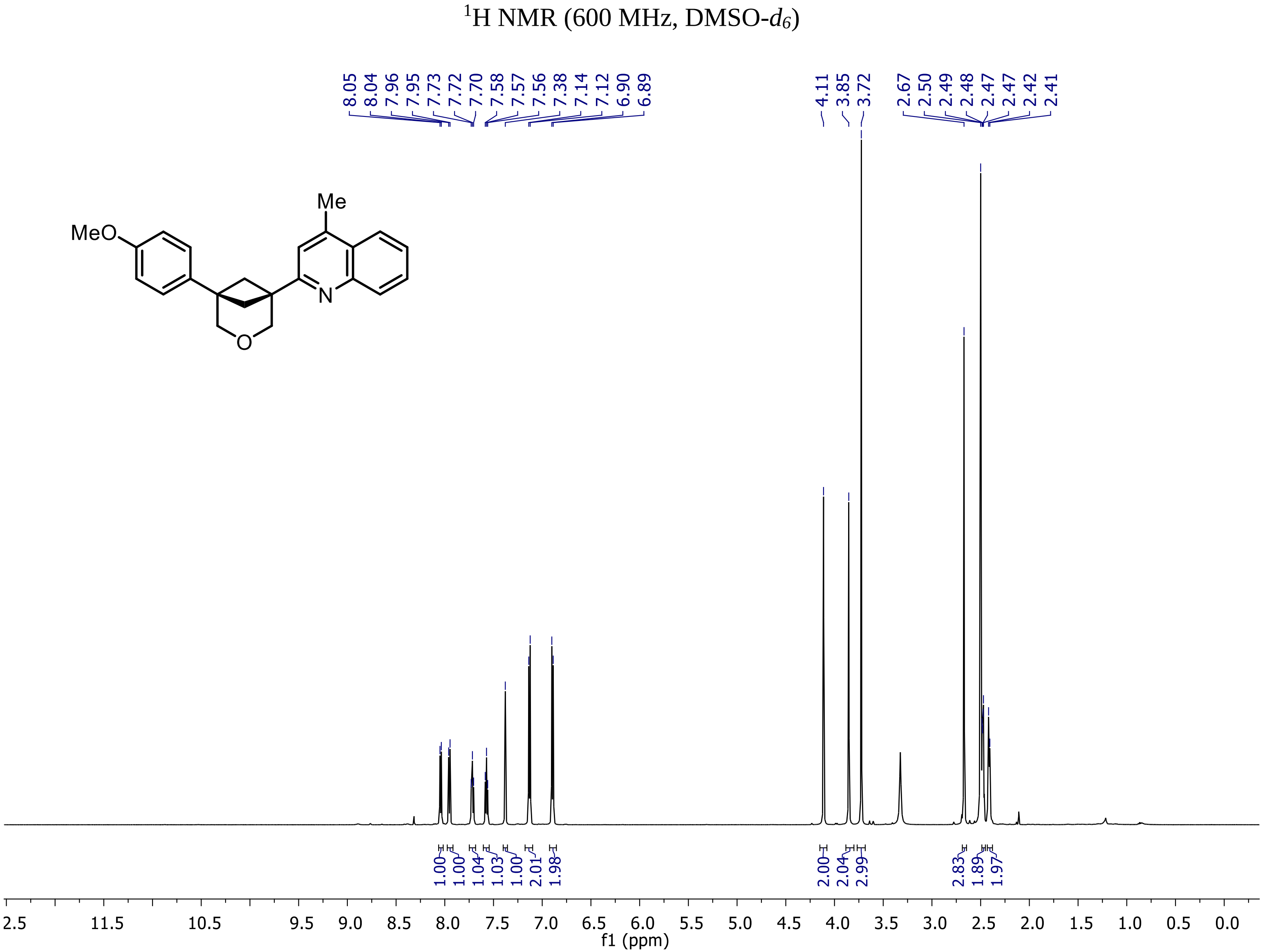

$^{13}C\{^1H\}$ NMR (151 MHz, DMSO-d_6)

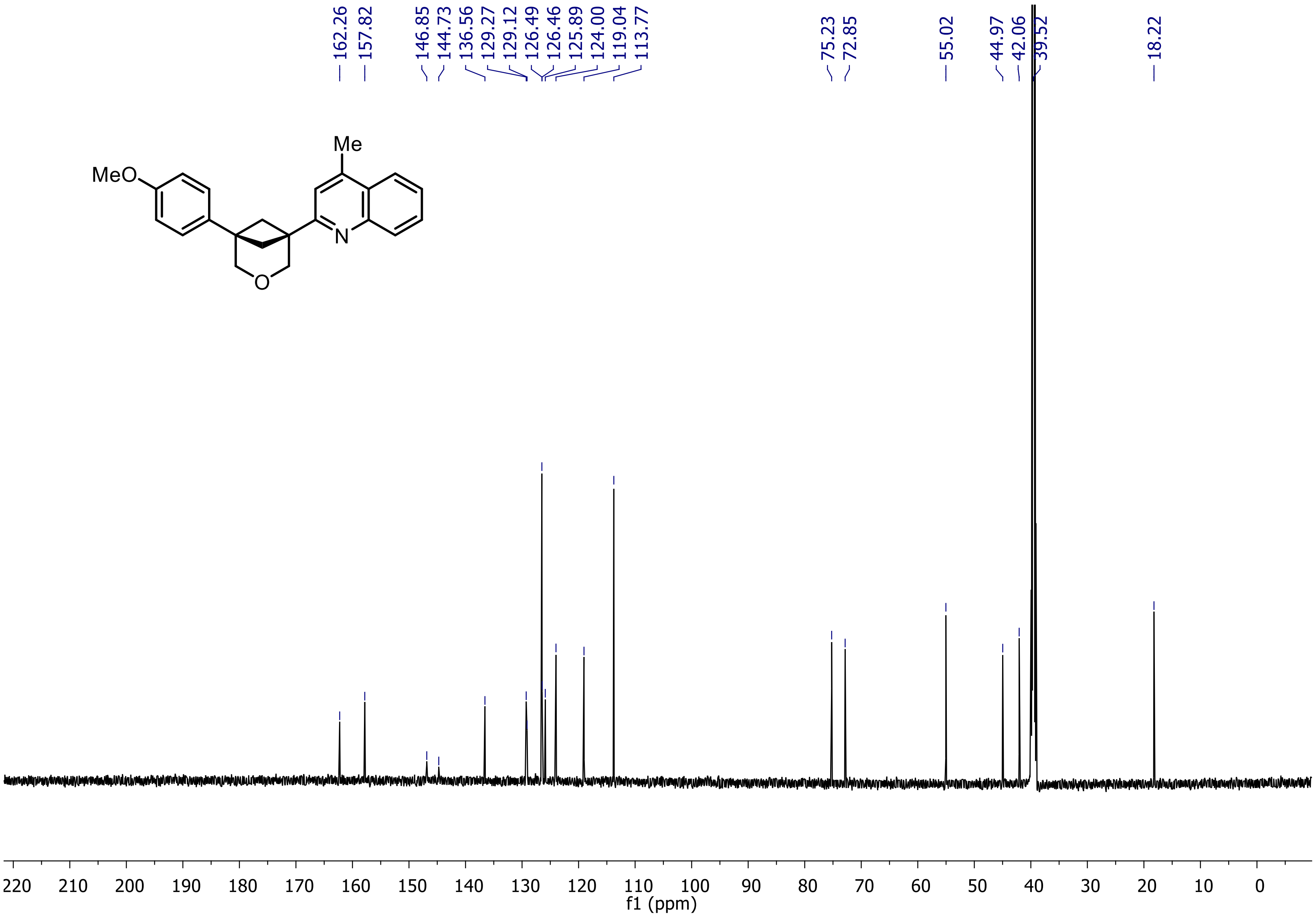

Compound 23d

^{1}H NMR (500 MHz, CDCl$_3$)

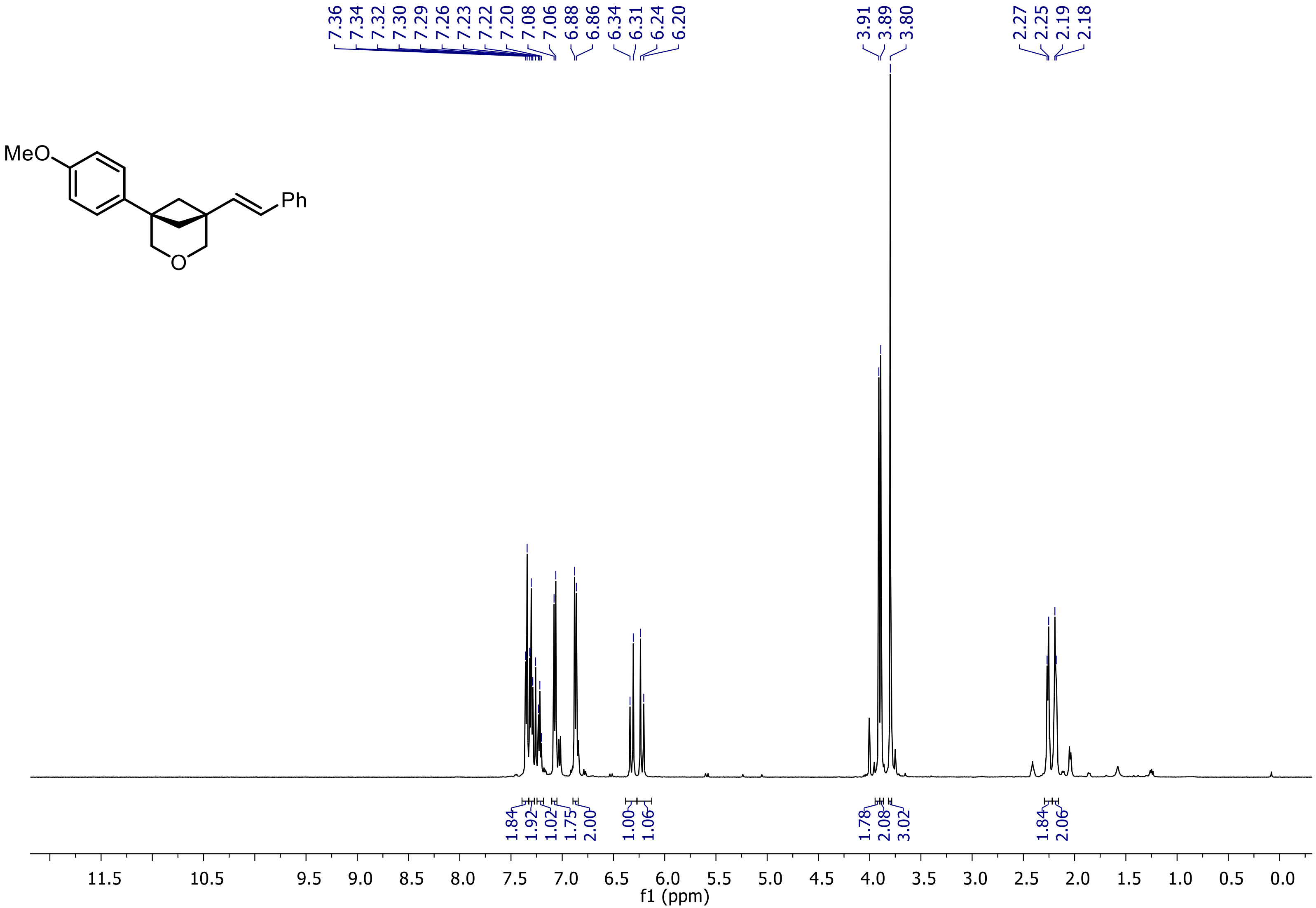

$^{13}C\{^1H\}$ NMR (151 MHz, $CDCl_3$)

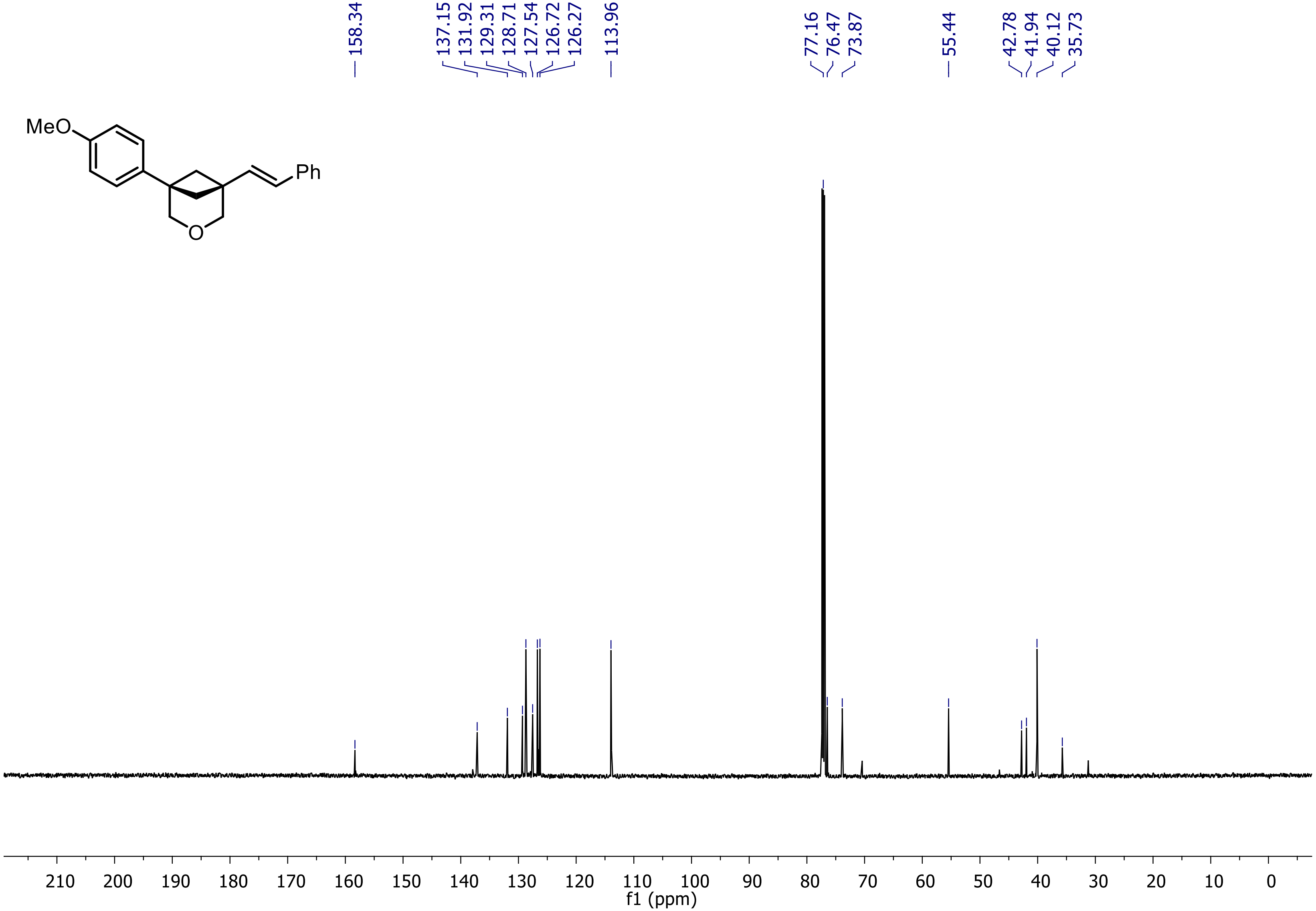

Compound 23e

^{1}H NMR (400 MHz, DMSO-d_6)

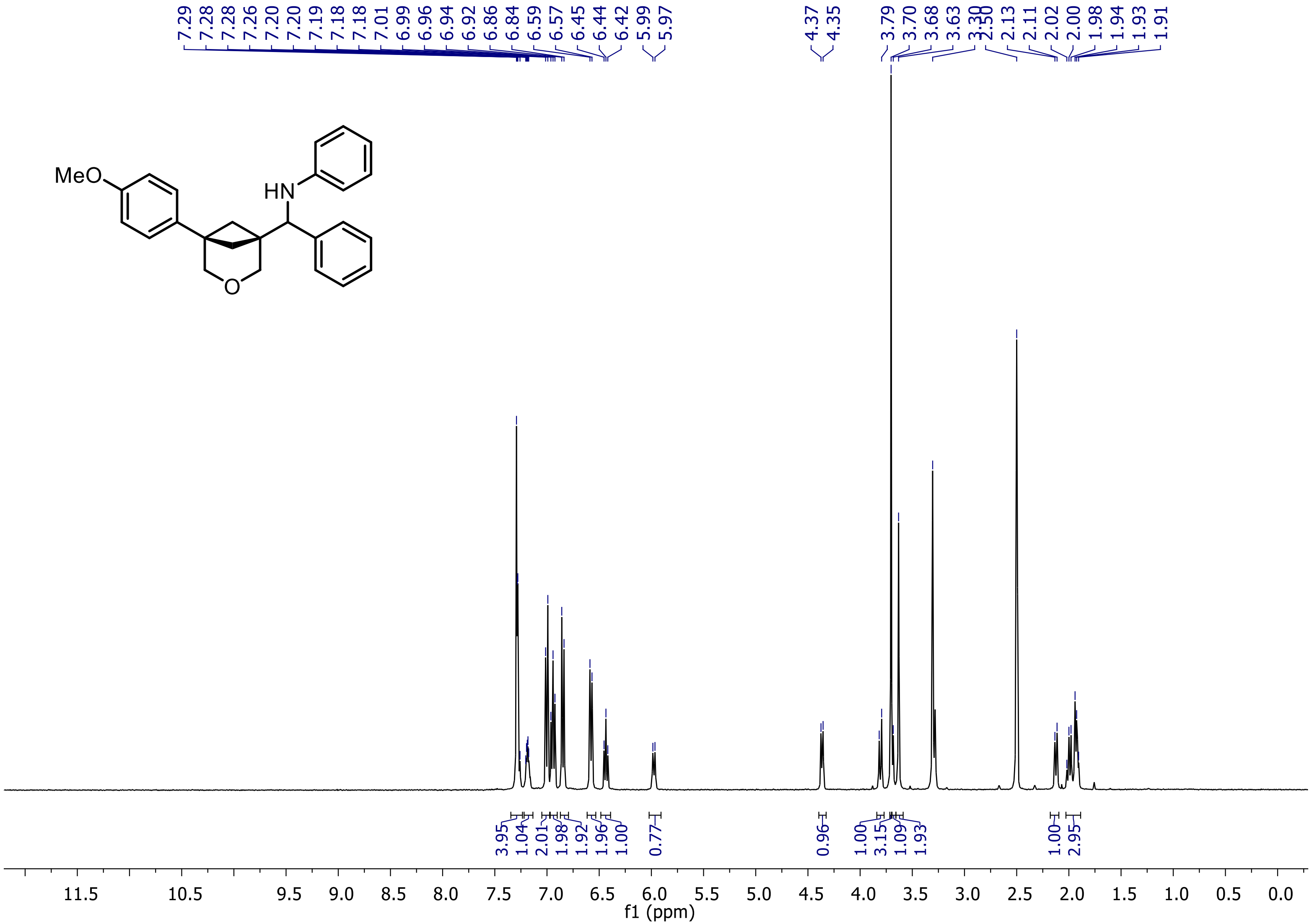

$^{13}C\{^1H\}$ NMR (151 MHz, DMSO-d_6)

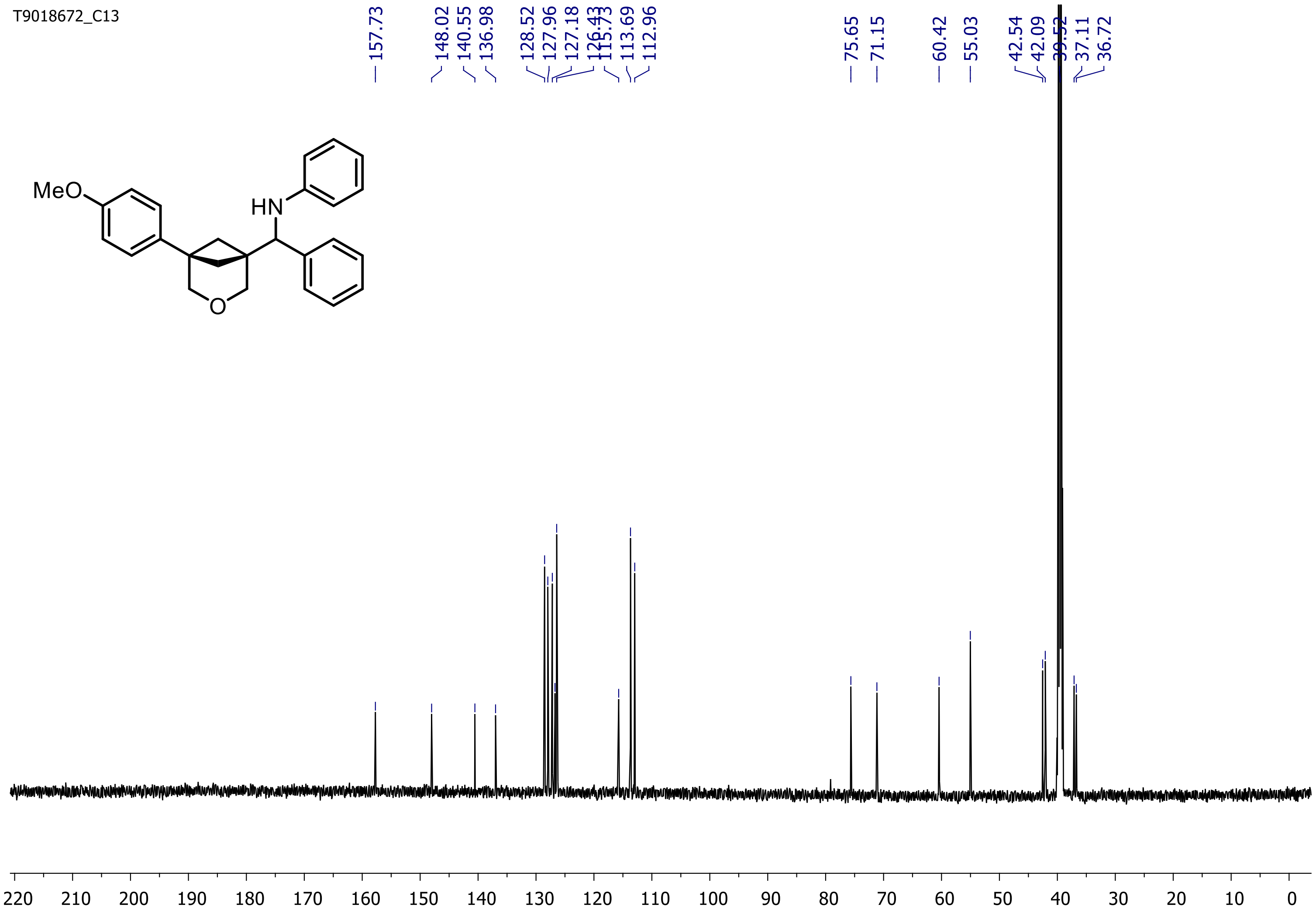

Compound 24

^{1}H NMR (400 MHz, DMSO-d_6)

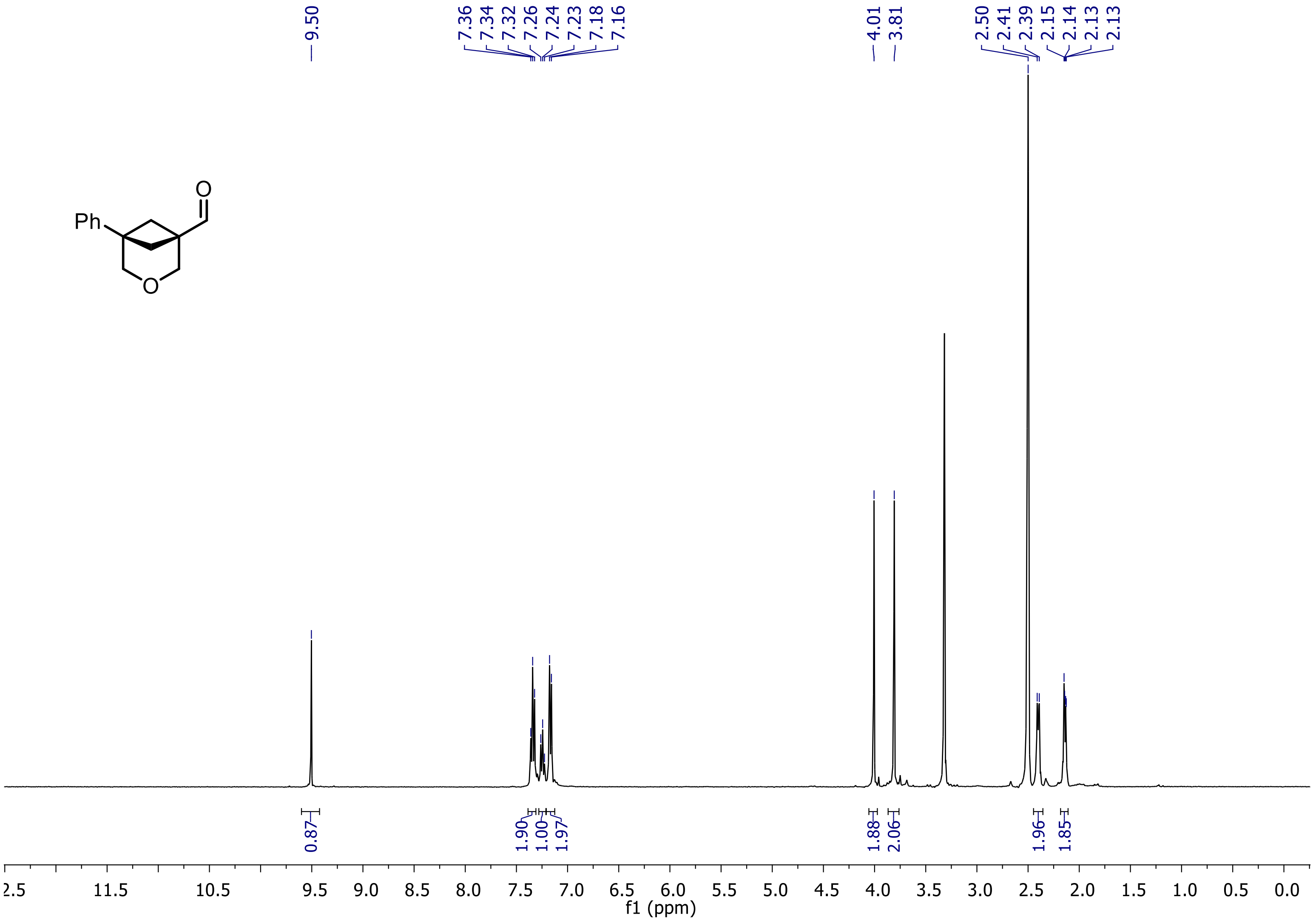

$^{13}C\{^1H\}$ NMR (126 MHz, $CDCl_3$)

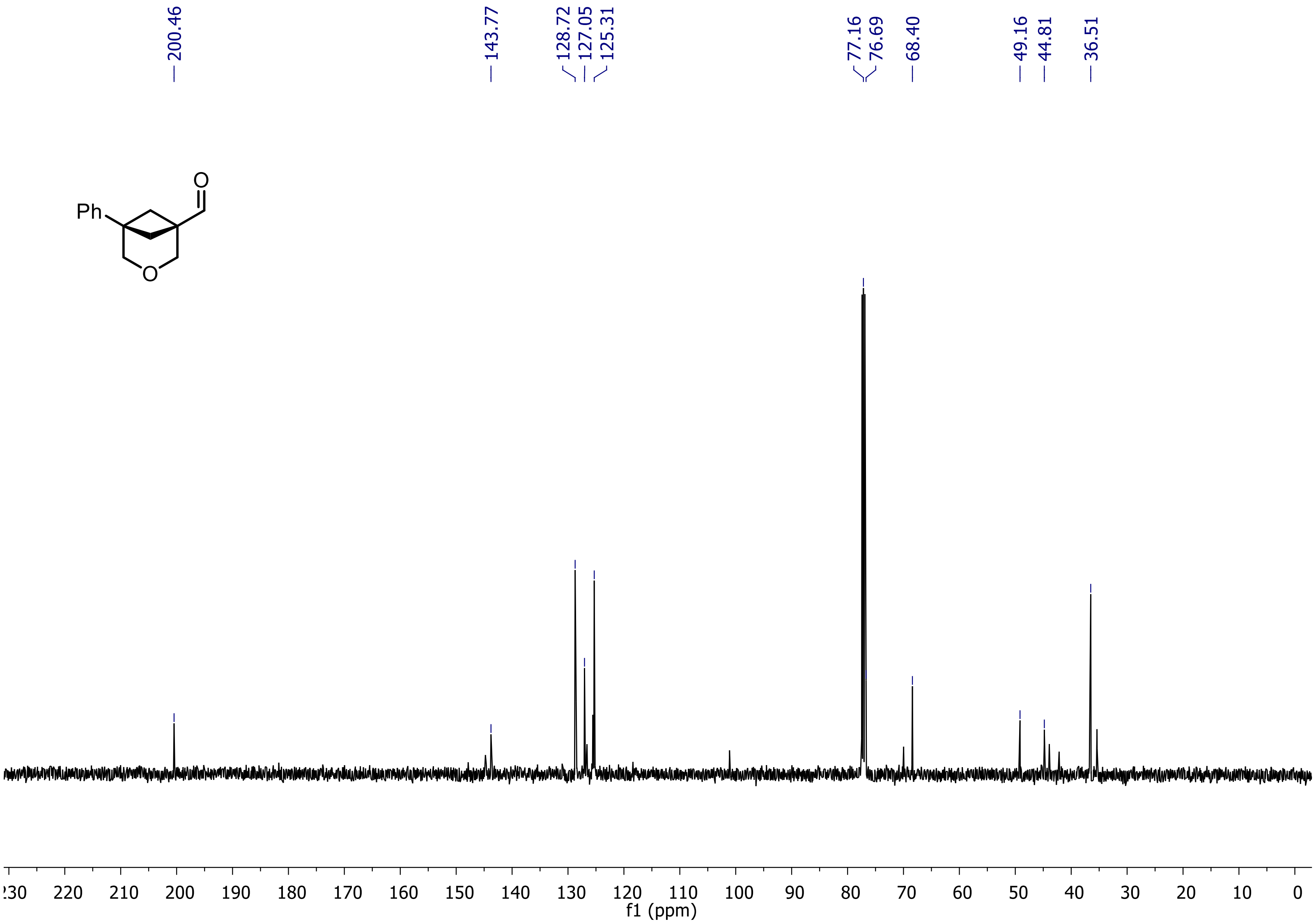

Compound 25

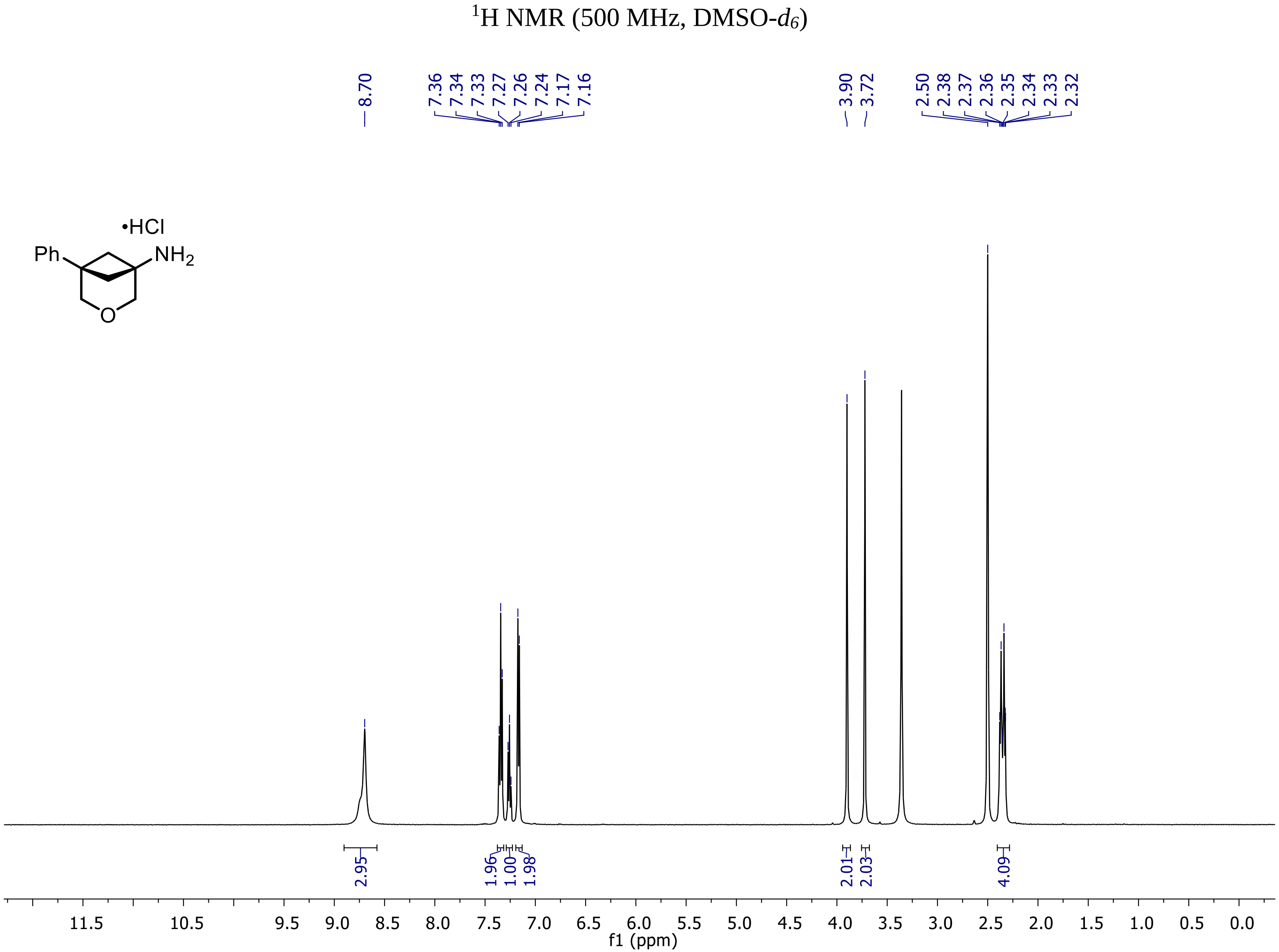
•HCl
Ph
NH_2
O
^{1}H NMR (500 MHz, DMSO-d_6)
8.70
7.36
7.34
7.33
7.27
7.26
7.24
7.17
7.16
3.90
3.72
2.50
2.38
2.37
2.36
2.35
2.34
2.33
2.32
2.95
1.96
1.00
1.98
2.01
2.03
4.09
11.5
10.5
9.5
9.0
8.5
8.0
7.5
7.0
6.5
6.0
5.5
5.0
4.5
4.0
3.5
3.0
2.5
2.0
1.5
1.0
0.5
0.0
f1 (ppm)

$^{13}C\{^1H\}$ NMR (151 MHz, DMSO-d_6)

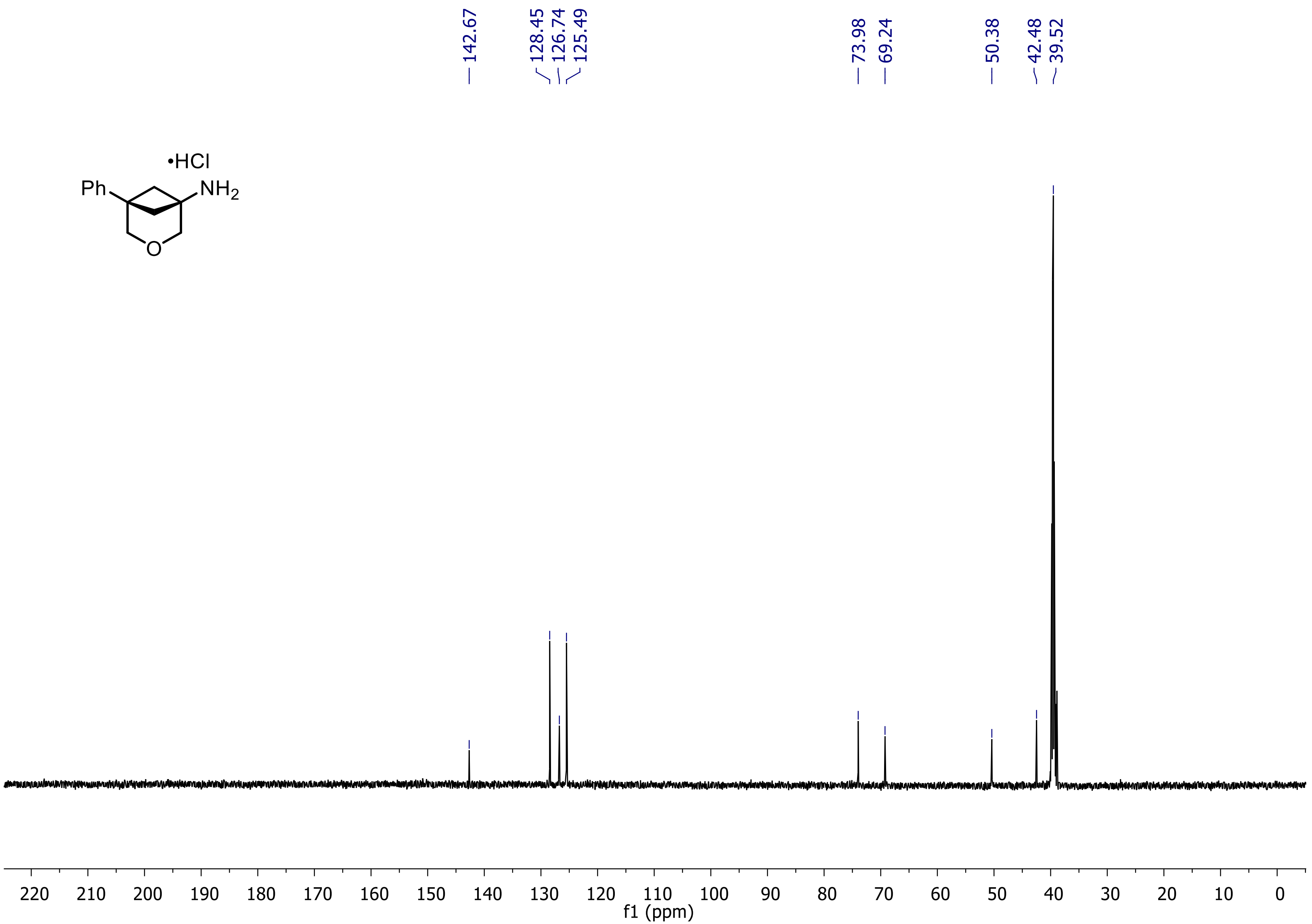

Compound 26

^{1}H NMR (500 MHz, DMSO-d_6)

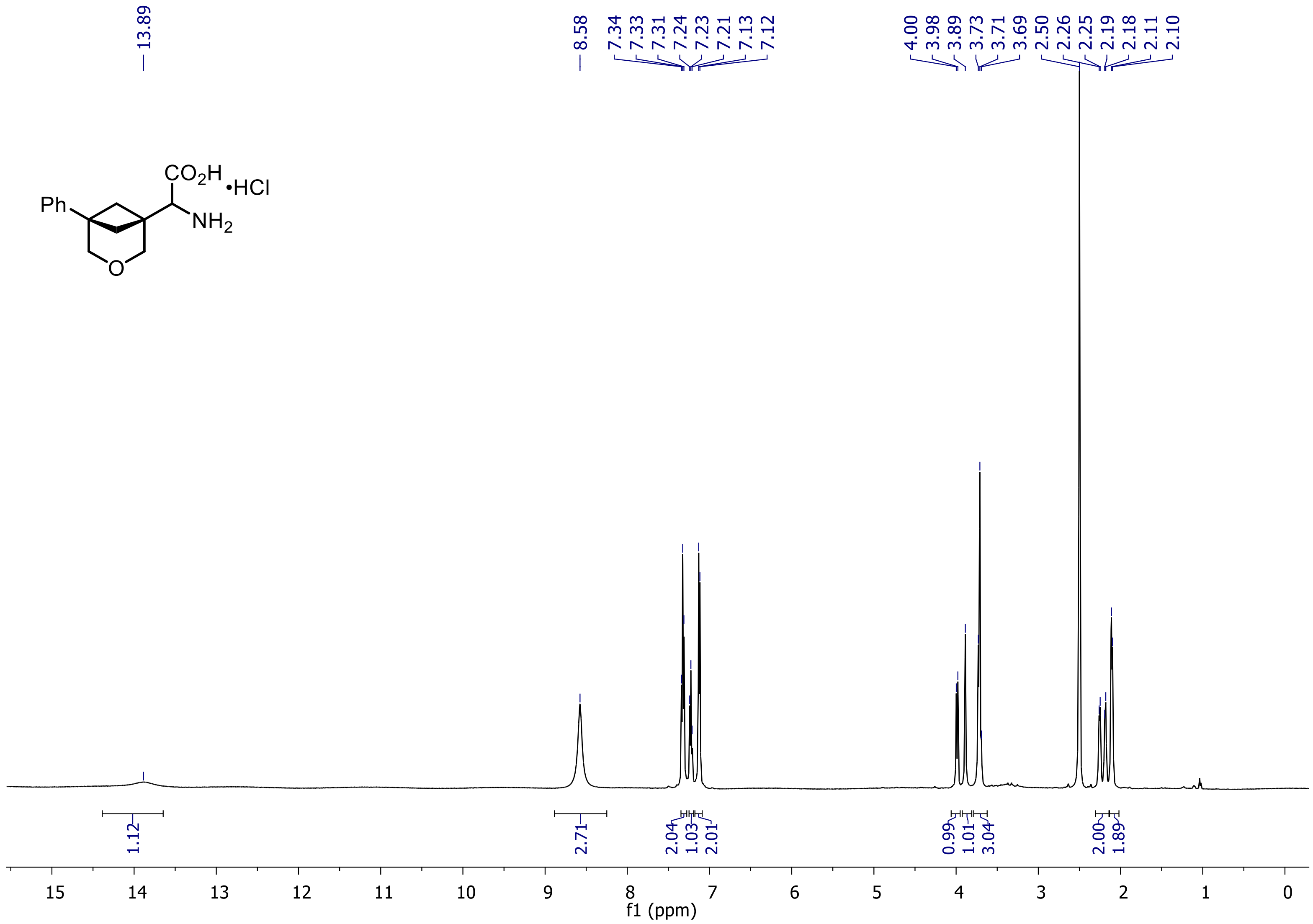

$^{13}C\{^{1}H\}$ NMR (126 MHz, DMSO-d_6)

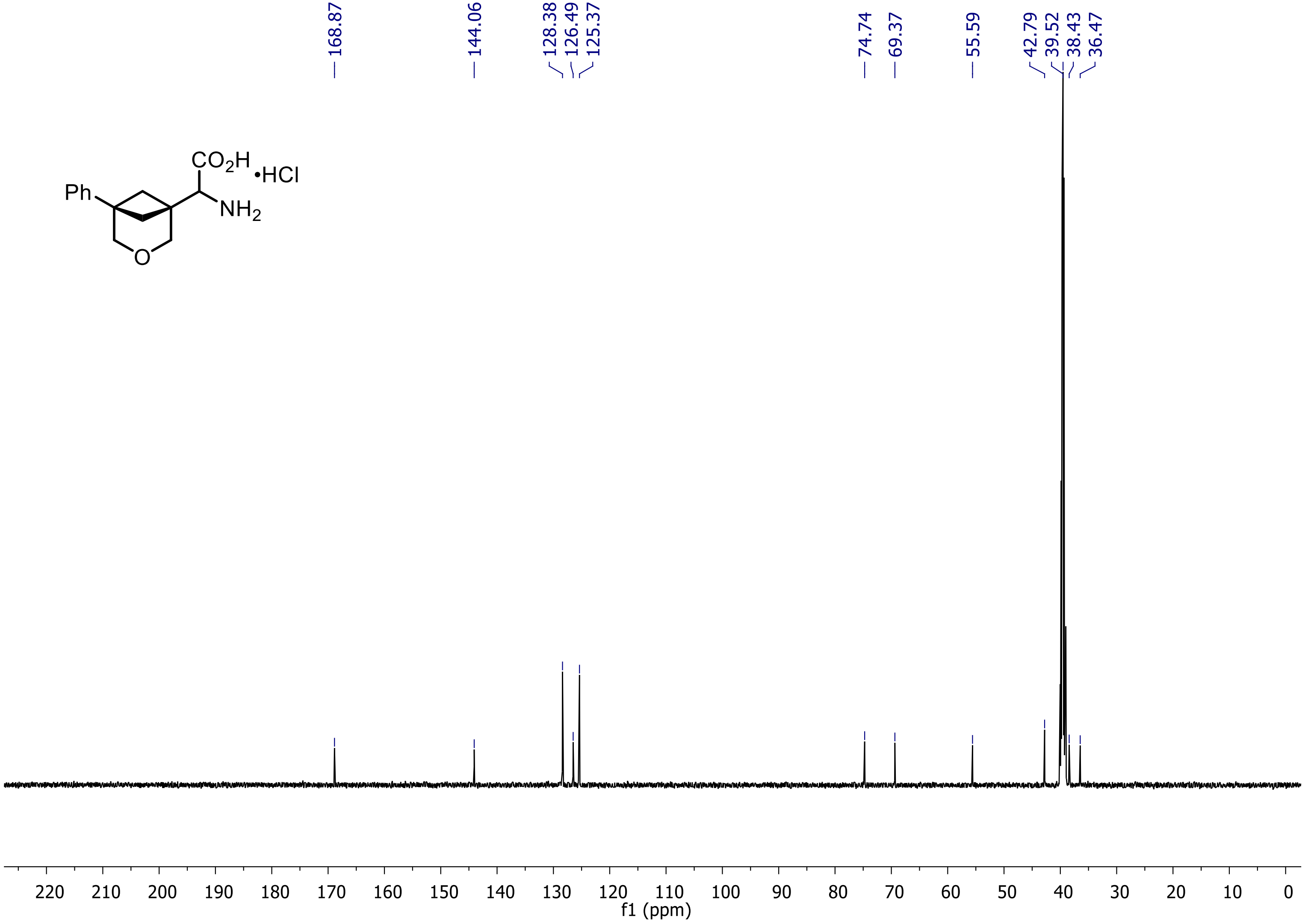

Compound 27

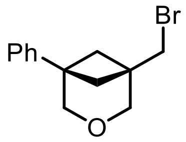
Br
Ph
O

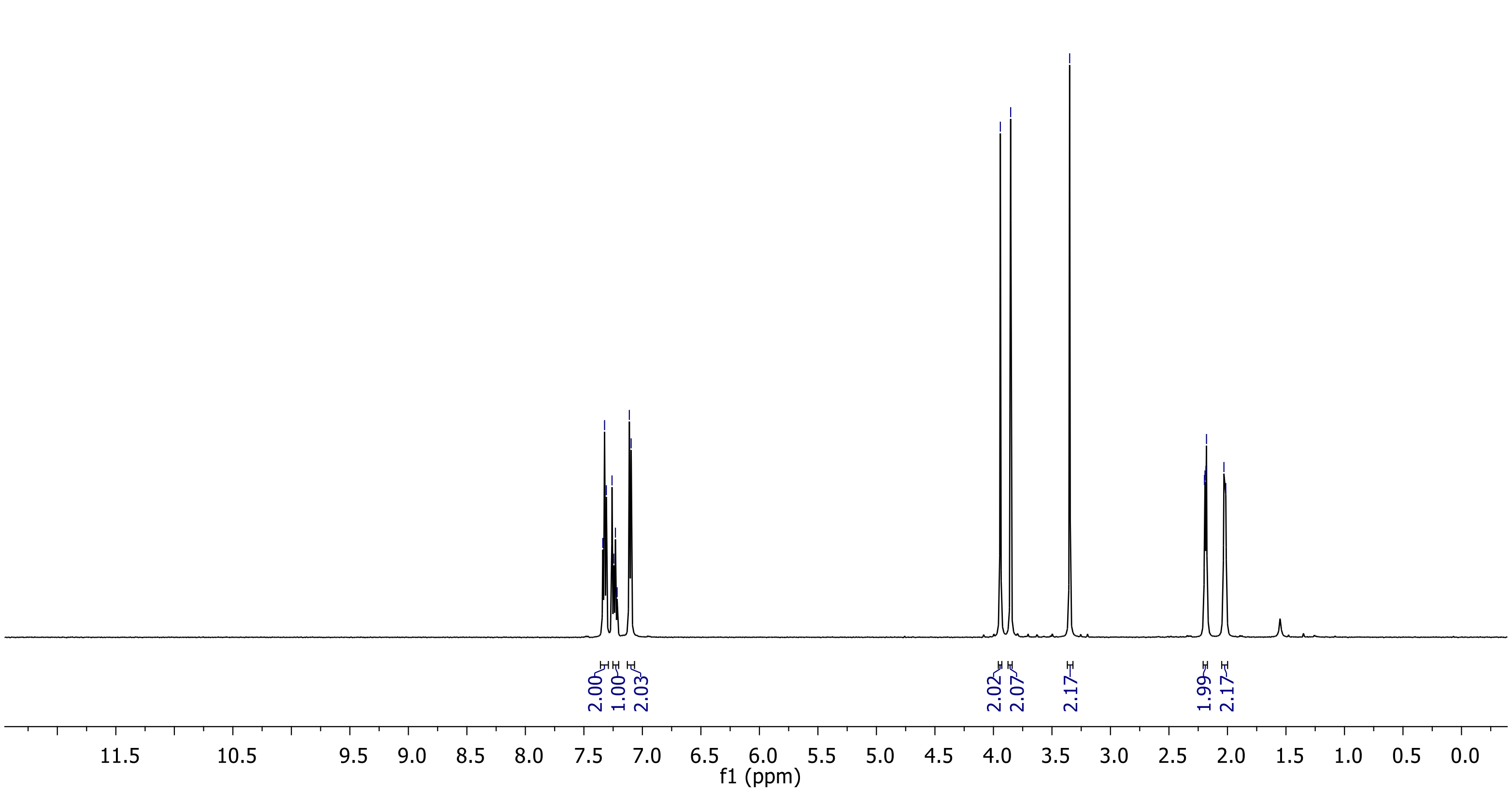
^{1}H NMR (500 MHz, CDCl$_3$)
7.34
7.32
7.31
7.26
7.25
7.23
7.22
7.11
7.10
3.94
3.85
3.35
2.20
2.19
2.18
2.18
2.03
2.01
2.00
1.00
2.03
2.02
2.07
2.17
1.99
2.17
11.5
10.5
9.5
9.0
8.5
8.0
7.5
7.0
6.5
6.0
5.5
5.0
4.5
4.0
3.5
3.0
2.5
2.0
1.5
1.0
0.5
0.0
f1 (ppm)

$^{13}C\{^1H\}$ NMR (126 MHz, $CDCl_3$)

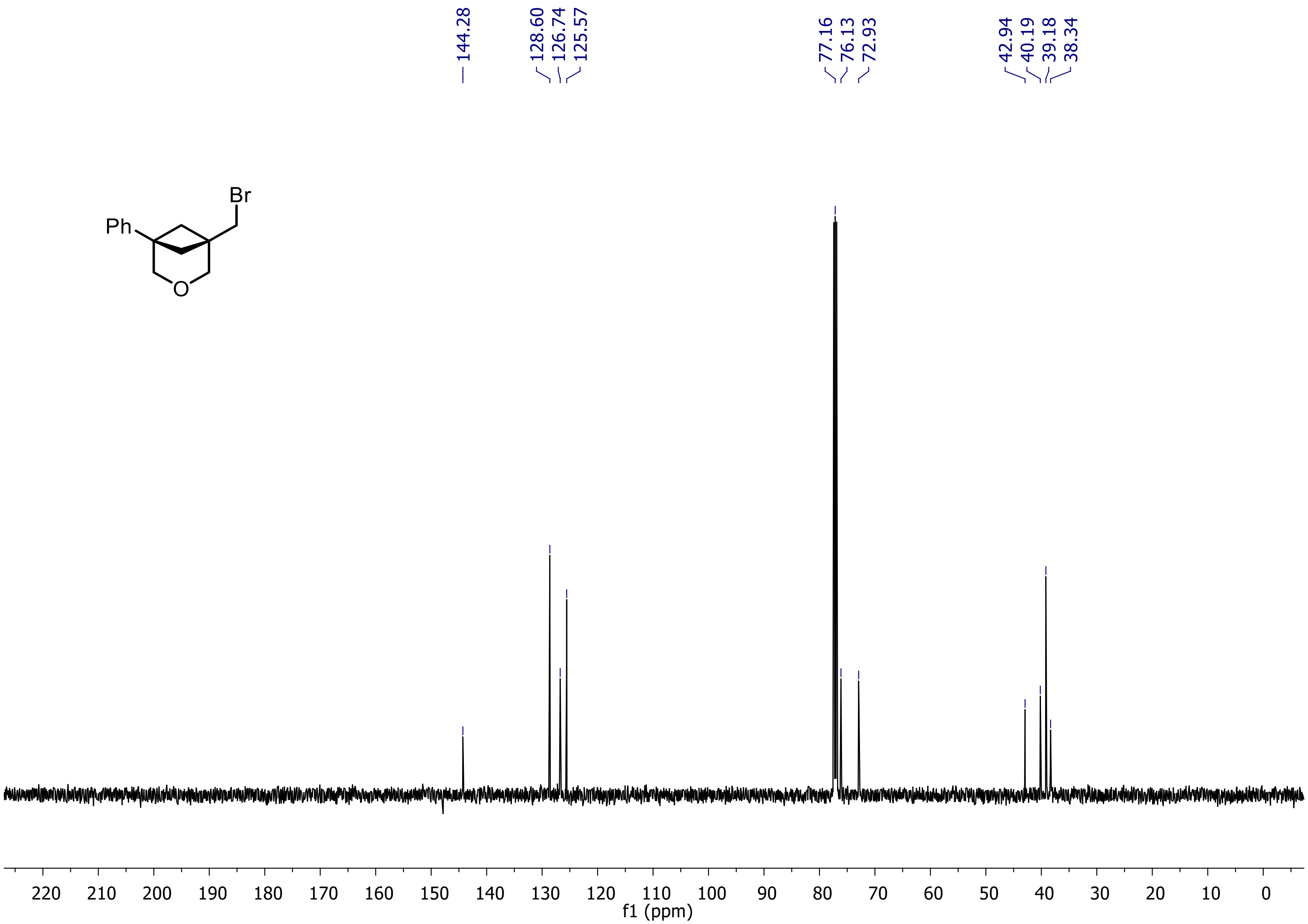

Compound 28

^{1}H NMR (500 MHz, DMSO-d_6)

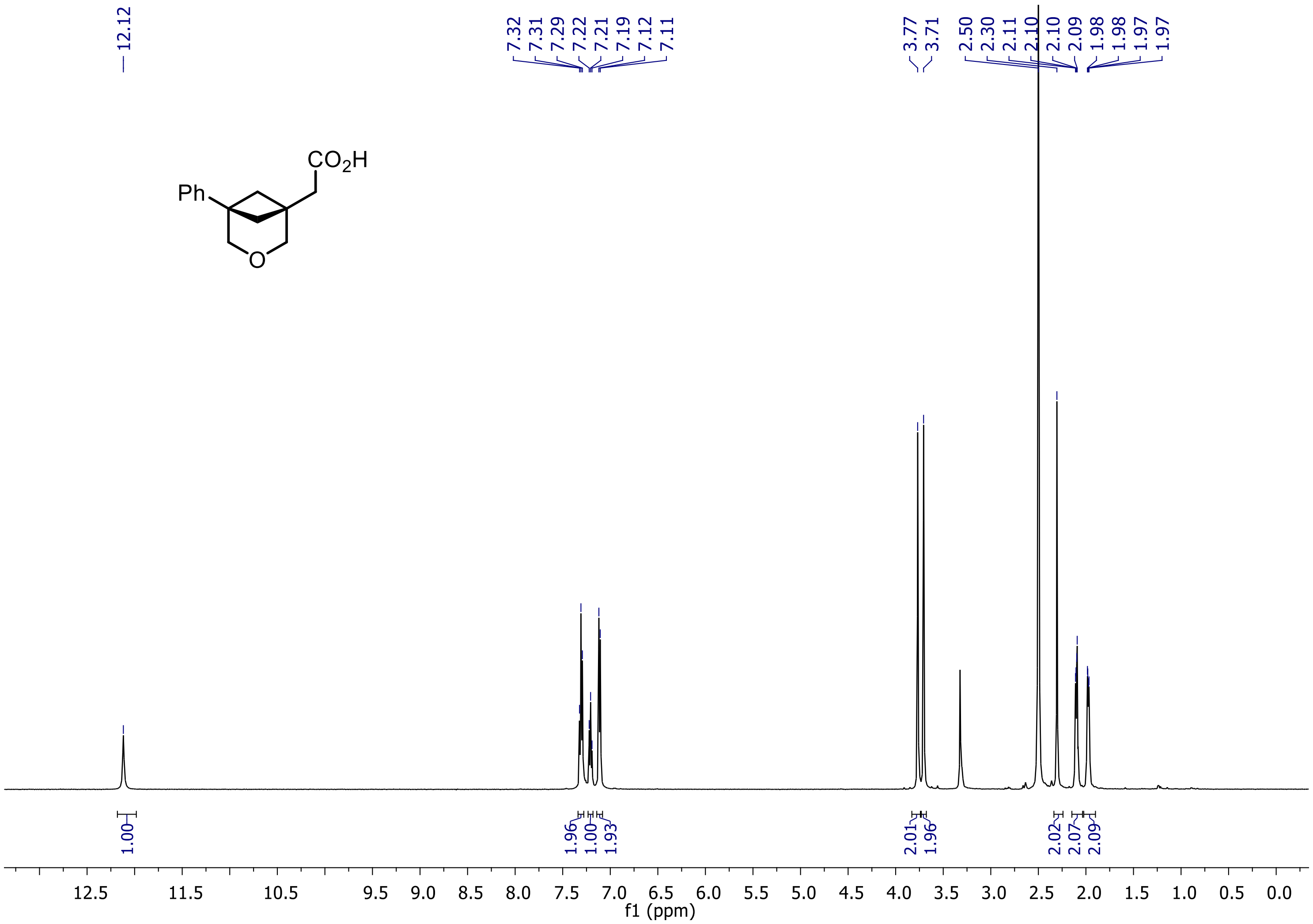

$^{13}C\{^1H\}$ NMR (151 MHz, DMSO-d_6)

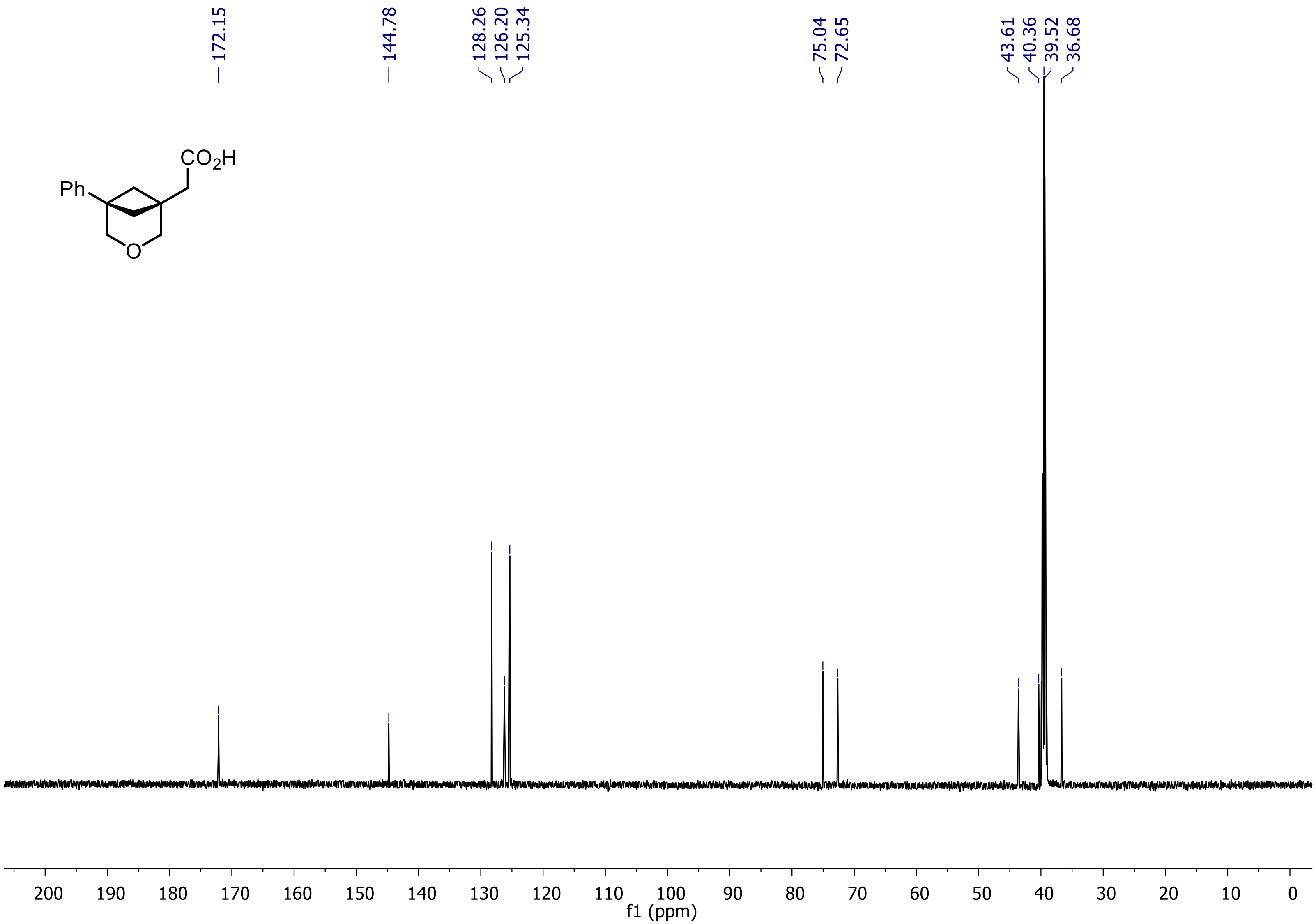

Compound 29

^{1}H NMR (500 MHz, DMSO-d_6)

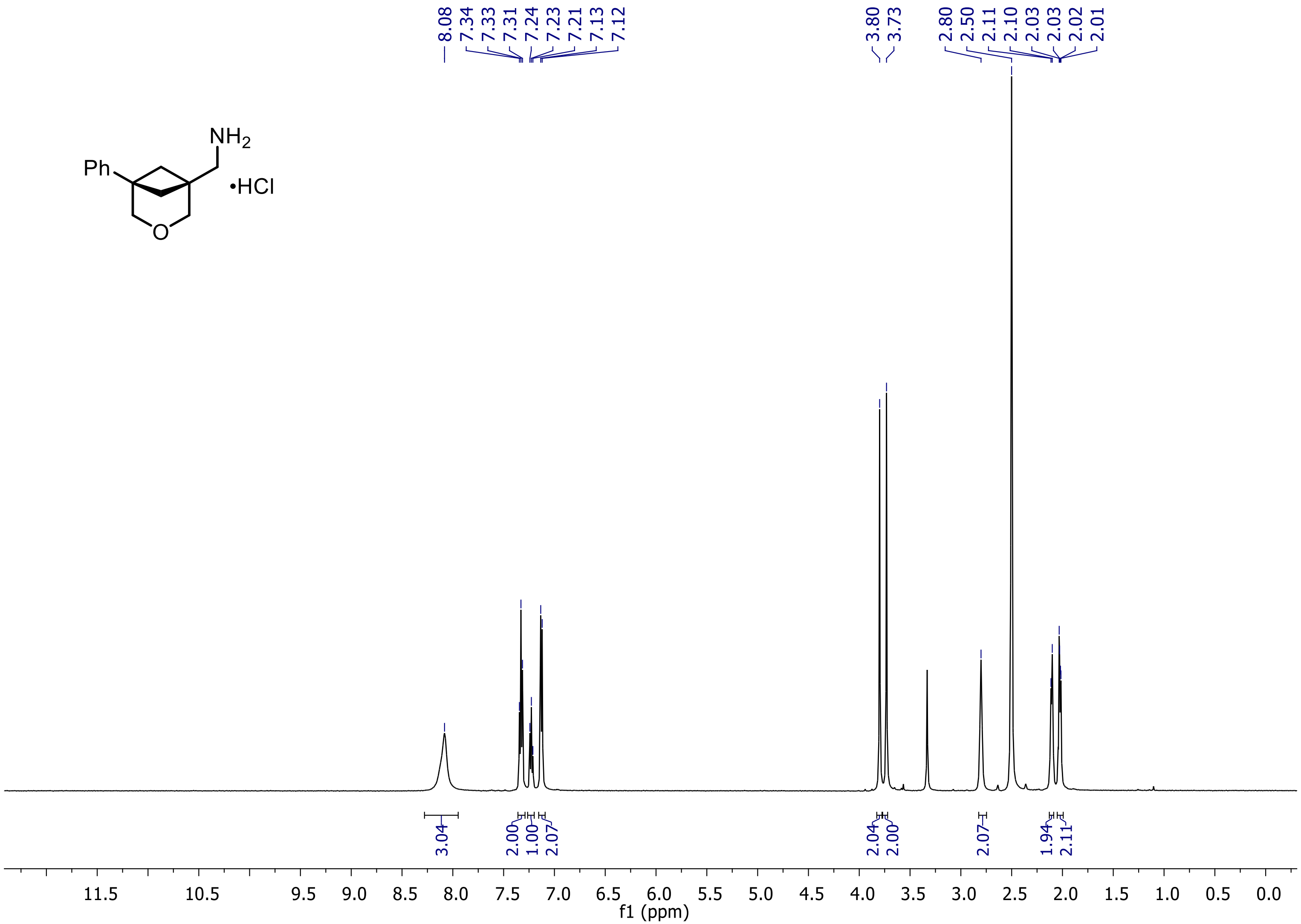

$^{13}C\{^{1}H\}$ NMR (126 MHz, DMSO-d_6)

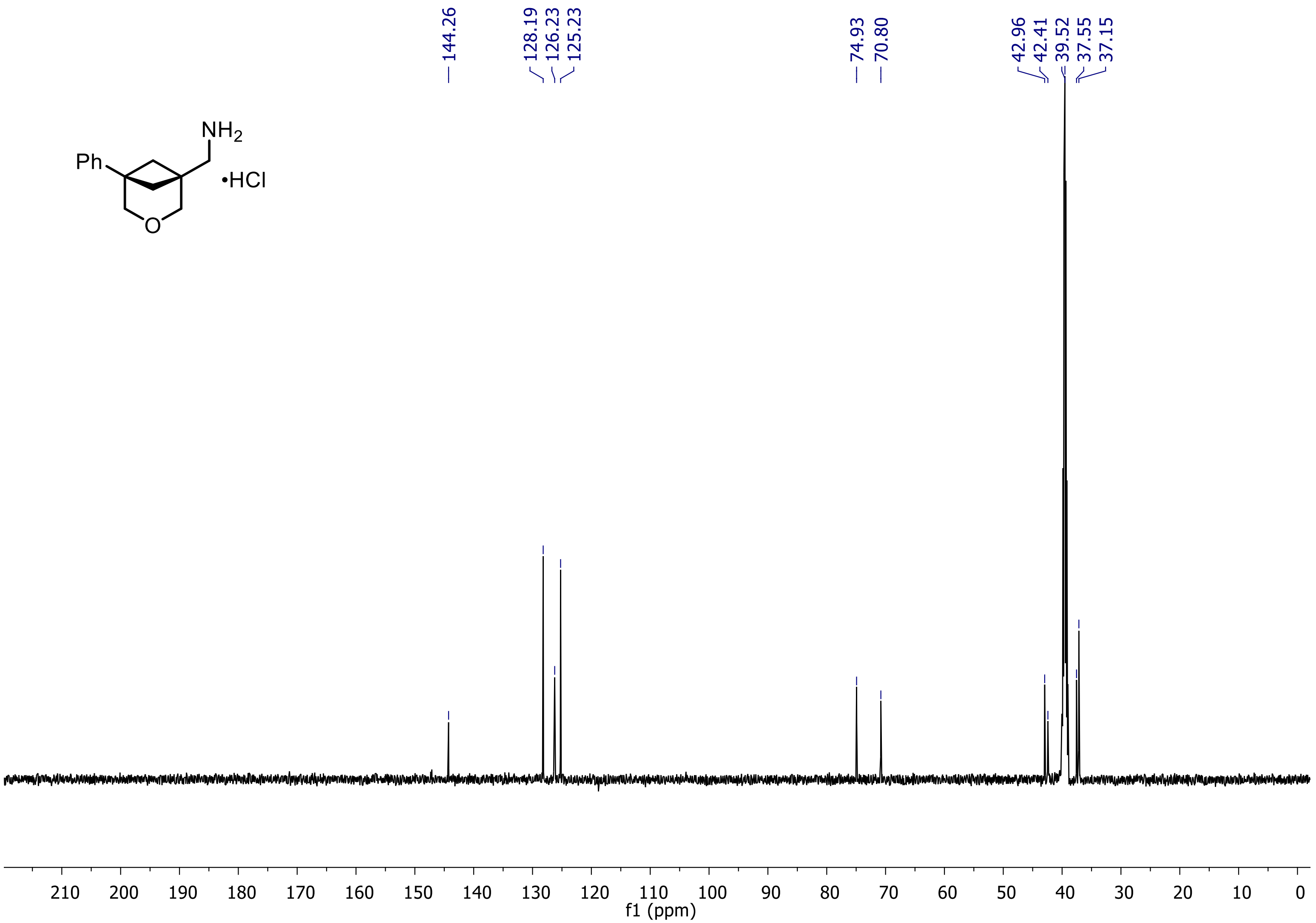

Compound 30

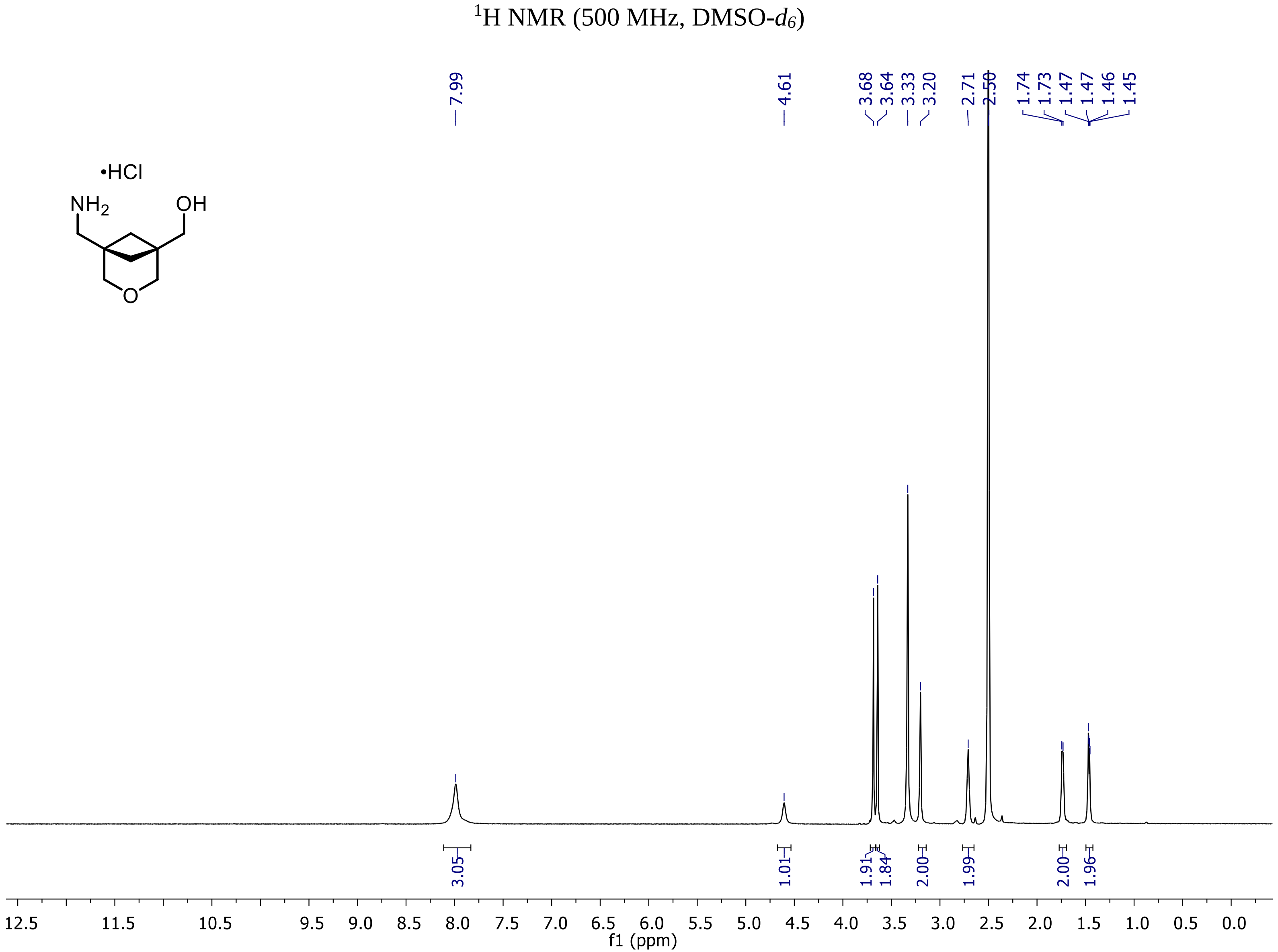
•HCl
NH2
OH
O
1H NMR (500 MHz, DMSO-d6)
7.99
4.61
3.68
3.64
3.33
3.20
2.71
2.50
1.74
1.73
1.47
1.47
1.46
1.45
3.05
1.01
1.91
1.84
2.00
1.99
2.00
1.96
f1 (ppm)

$^{13}C\{^{1}H\}$ NMR (126 MHz, DMSO-d_6)

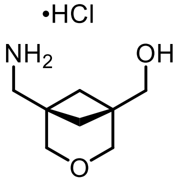

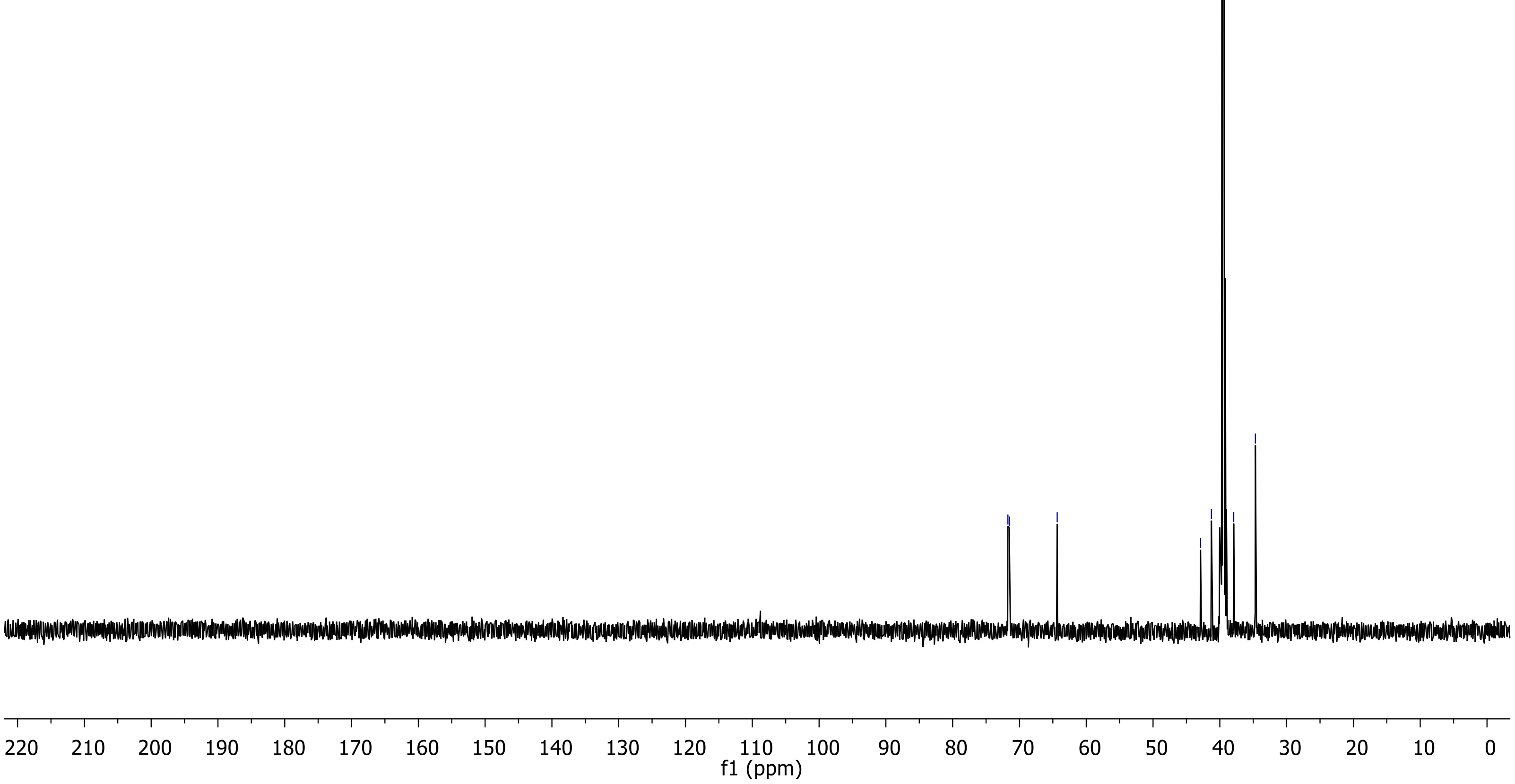

Compound 31

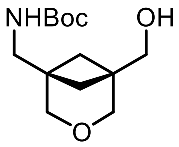

^1H NMR (500 MHz, $CDCl_3$)

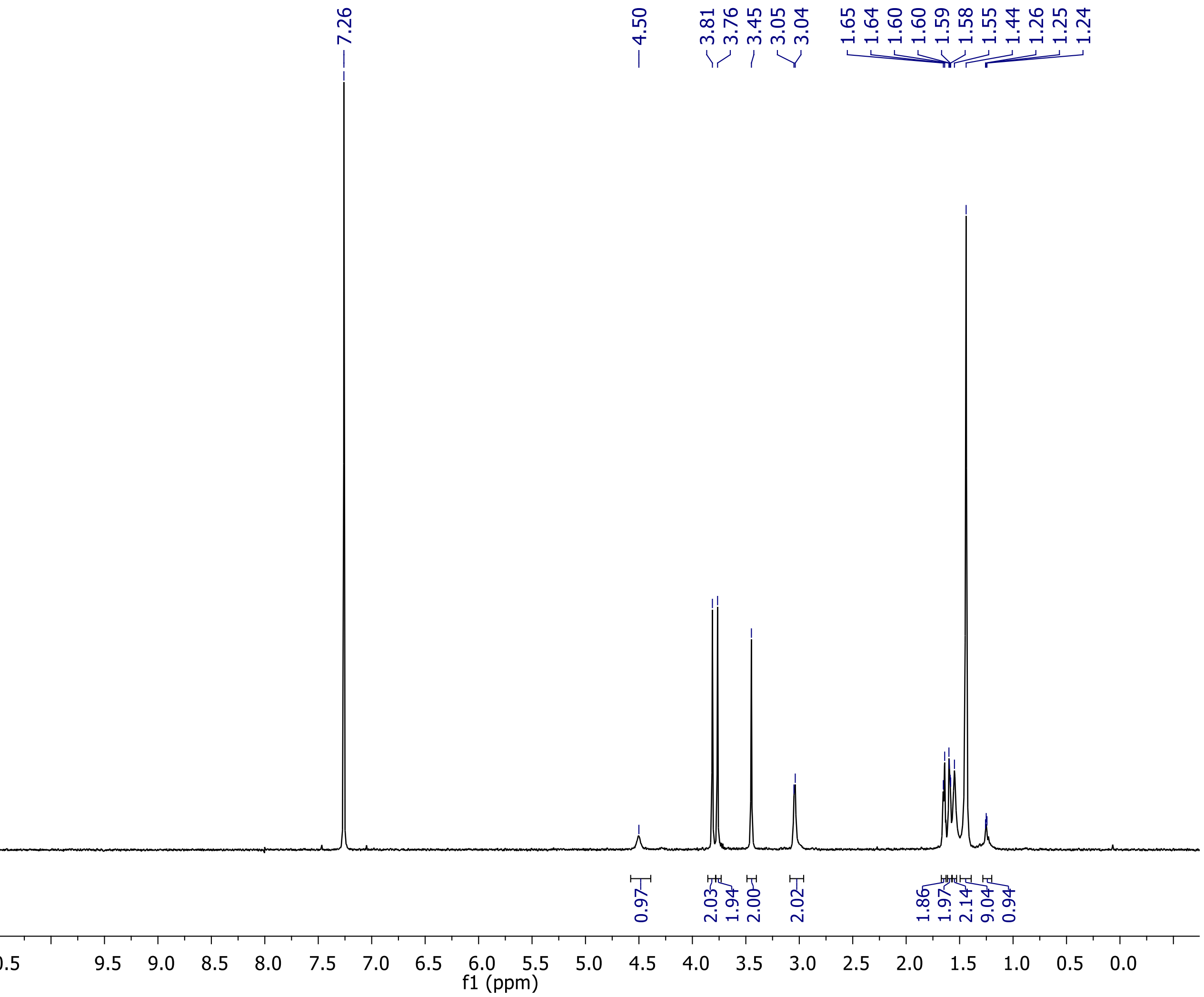

$^{13}C\{^{1}H\}$ NMR (126 MHz, $CDCl_3$)

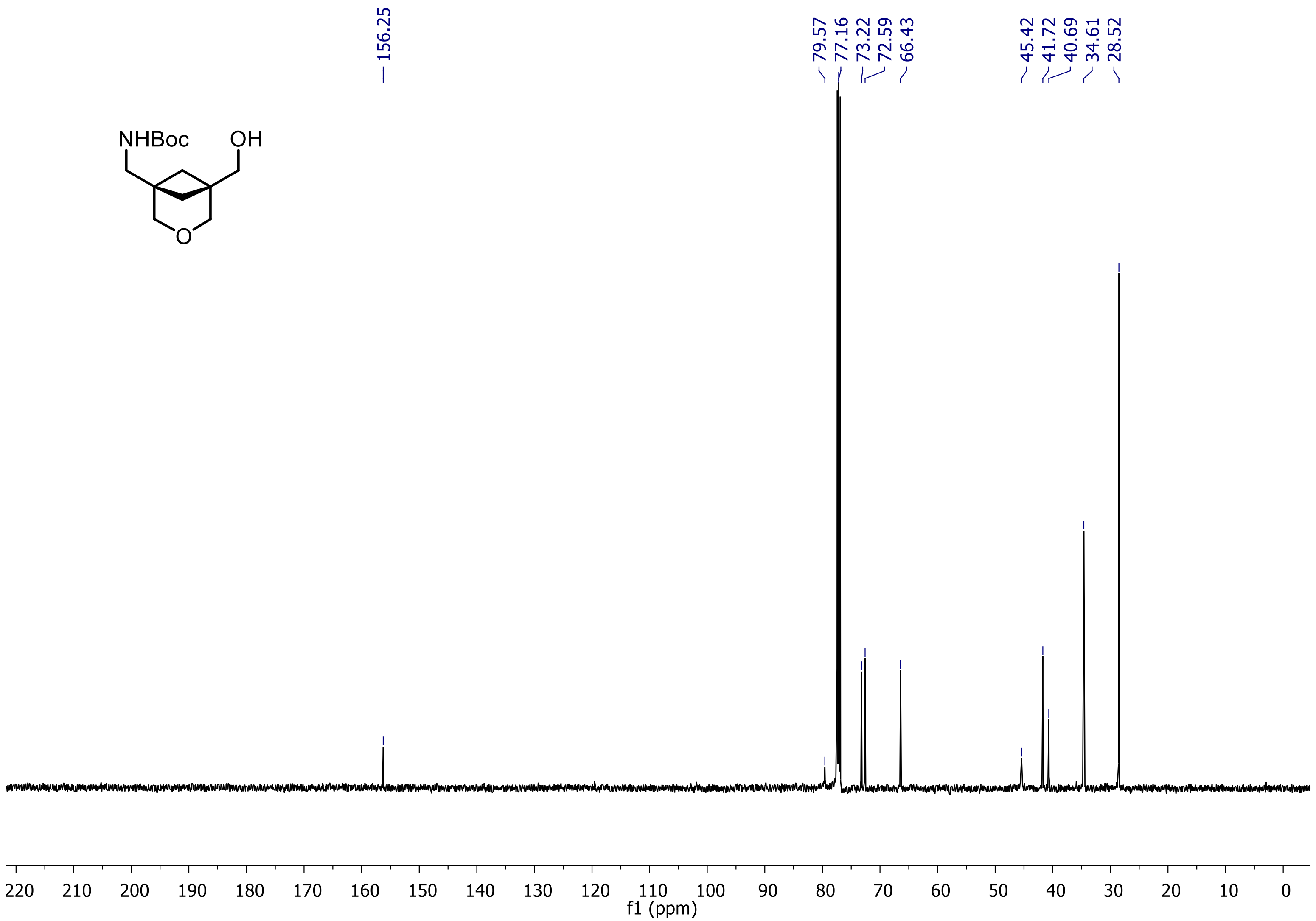

Compound 32

^{1}H NMR (500 MHz, DMSO-d_6)

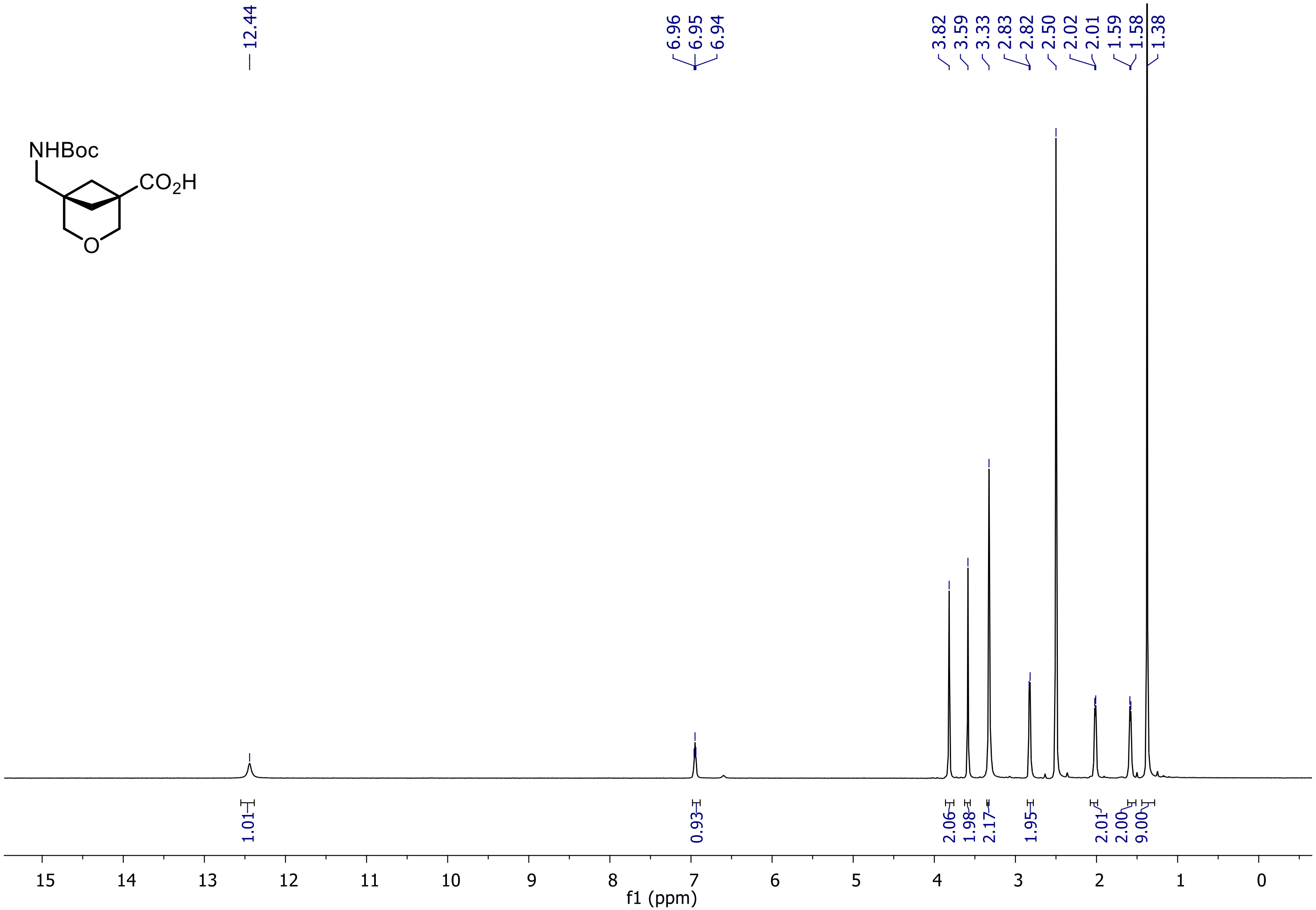

$^{13}C\{^1H\}$ NMR (151 MHz, $CDCl_3$)

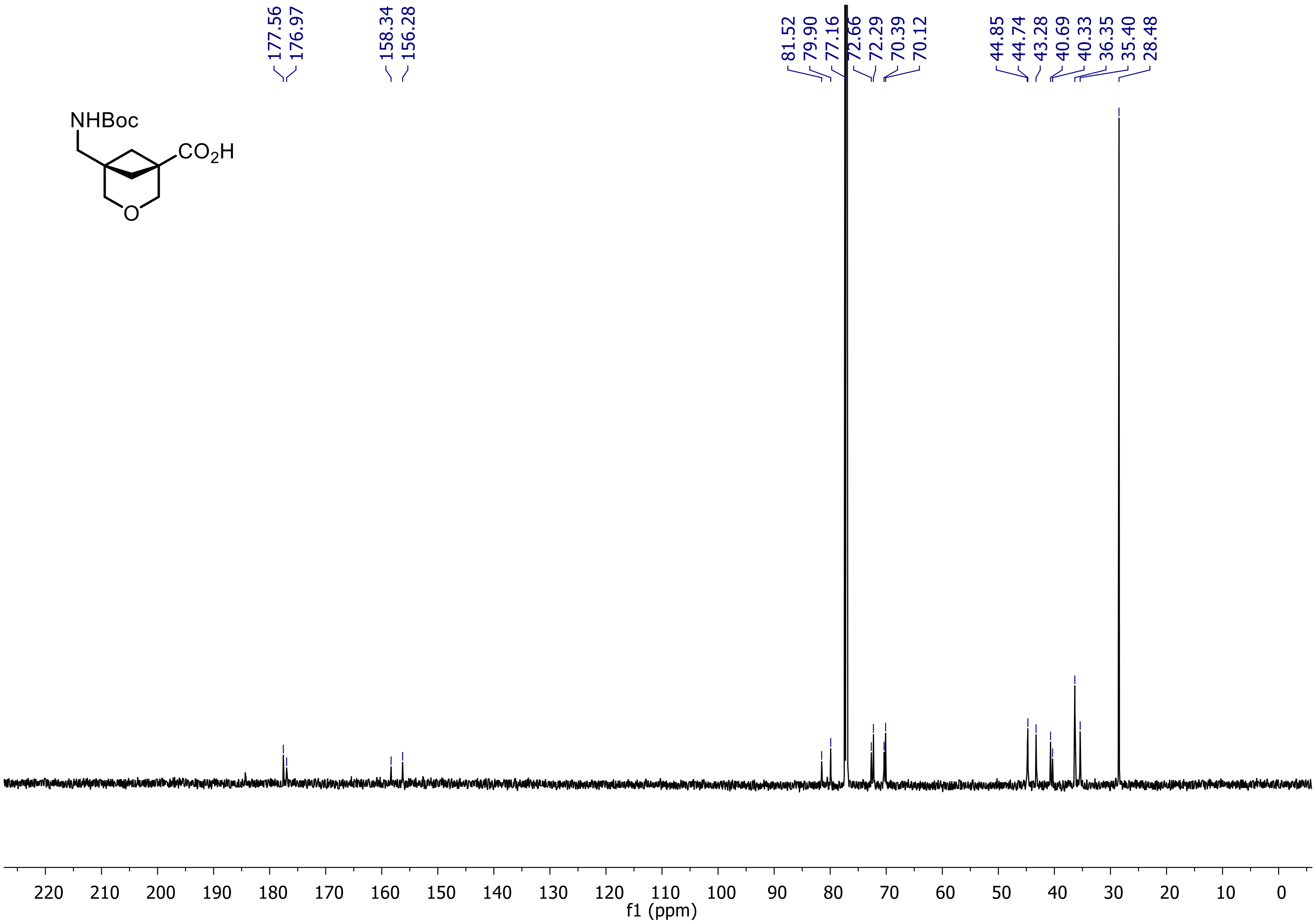

Compound 33

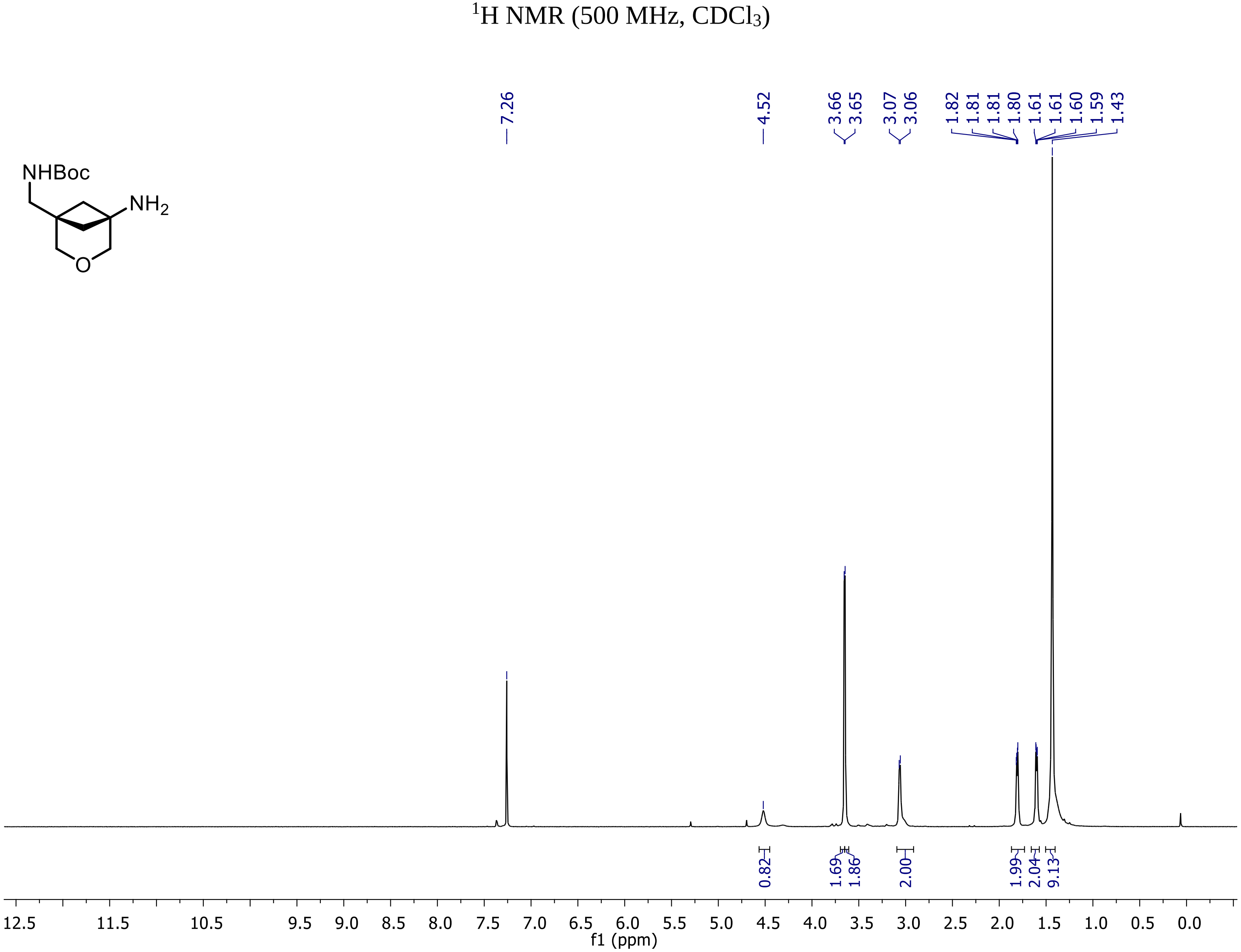
NHBoc
NH2
O
^{1}H NMR (500 MHz, $CDCl_3$)
7.26
4.52
3.66
3.65
3.07
3.06
1.82
1.81
1.81
1.80
1.61
1.61
1.60
1.59
1.43
0.82
1.69
1.86
2.00
1.99
2.04
9.13
12.5 11.5 10.5 9.5 9.0 8.5 8.0 7.5 7.0 6.5 6.0 5.5 5.0 4.5 4.0 3.5 3.0 2.5 2.0 1.5 1.0 0.5 0.0
f1 (ppm)

$^{13}C\{^{1}H\}$ NMR (126 MHz, $CDCl_3$)

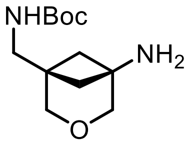

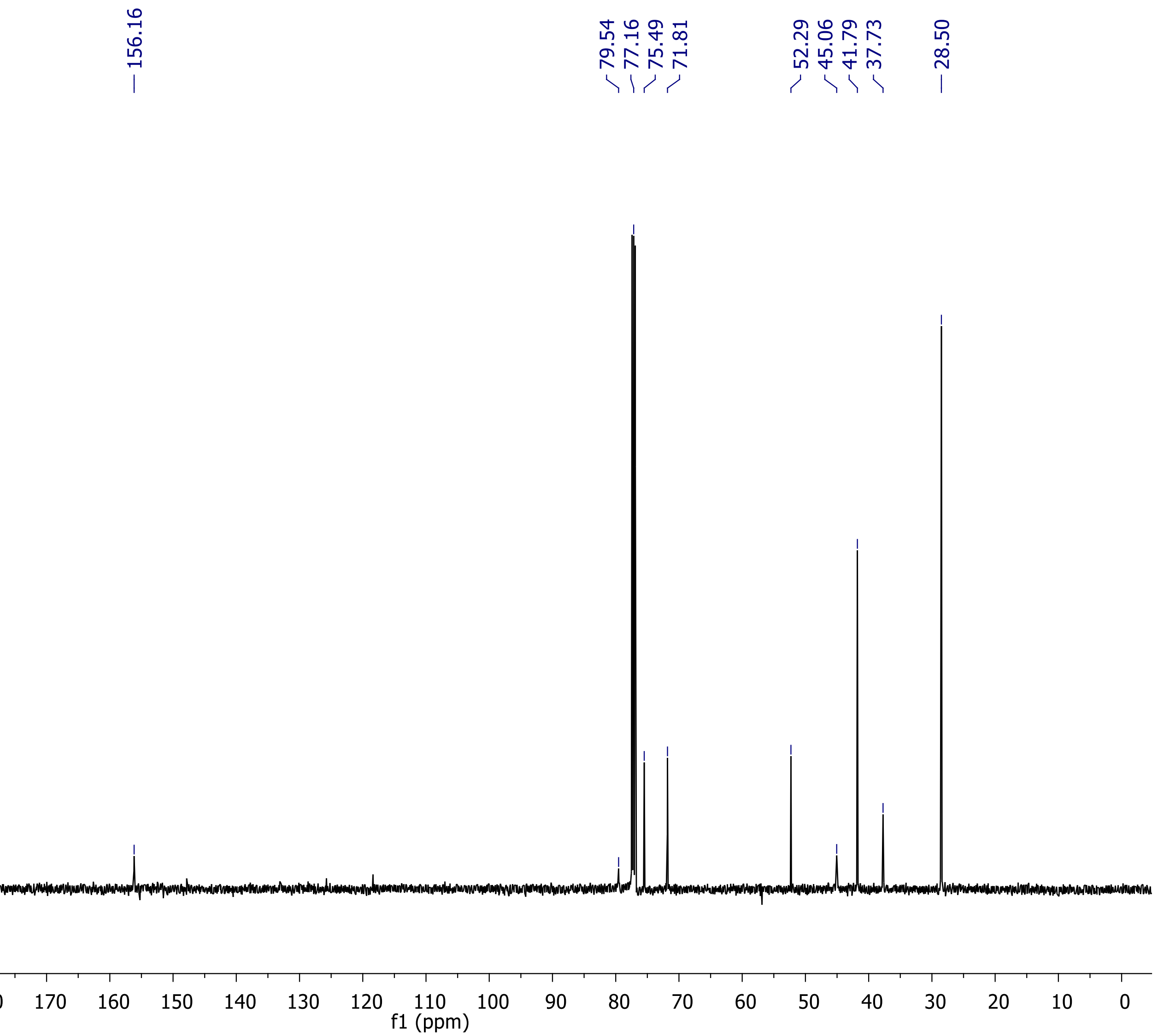

tert-Butyl ((5-(bromomethyl)-3-oxabicyclo[3.1.1]heptan-1-yl)methyl)carbamate

^{1}H NMR (500 MHz, CDCl$_3$)

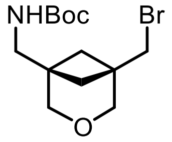

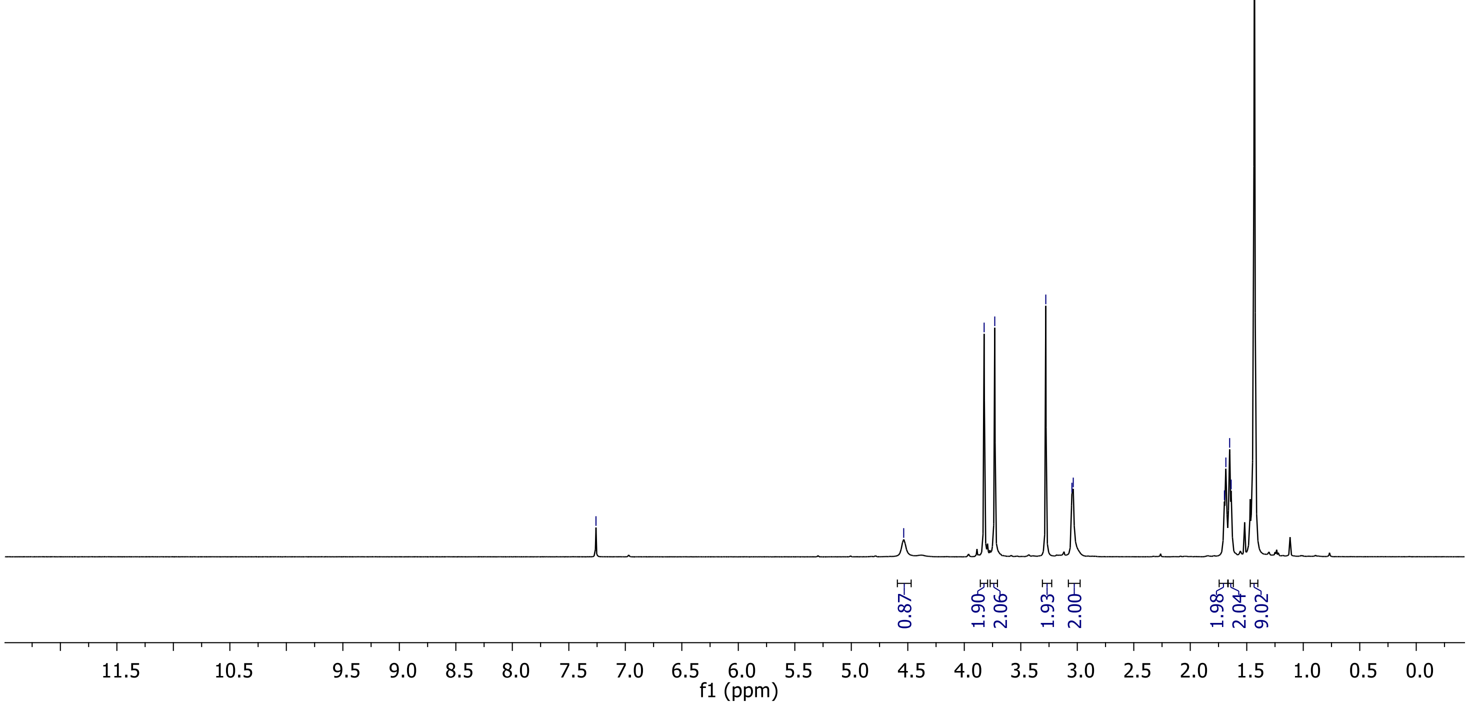

$^{13}C\{^1H\}$ NMR (151 MHz, $CDCl_3$)

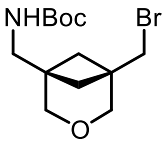

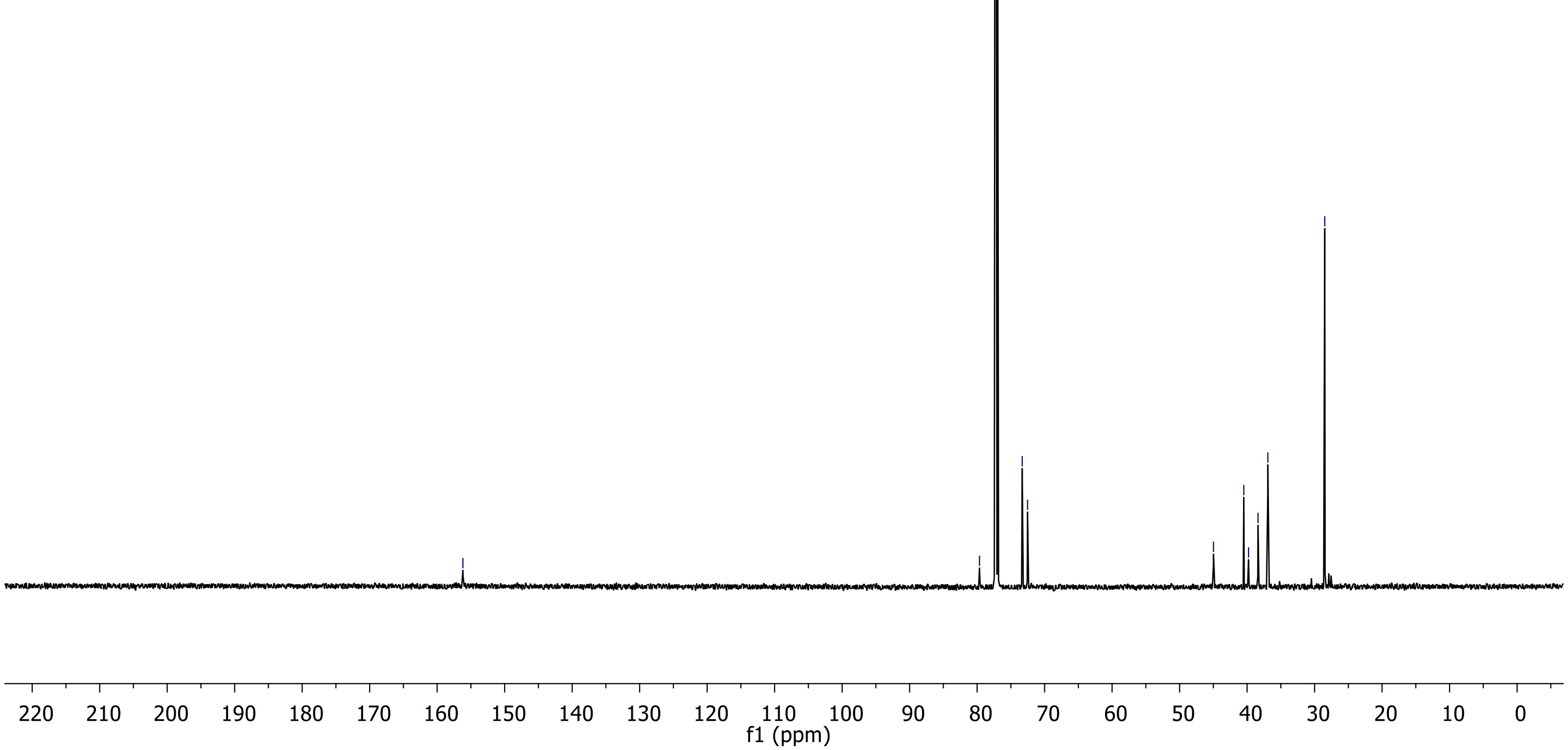

Compound 34

^{1}H NMR (500 MHz, $CDCl_3$)

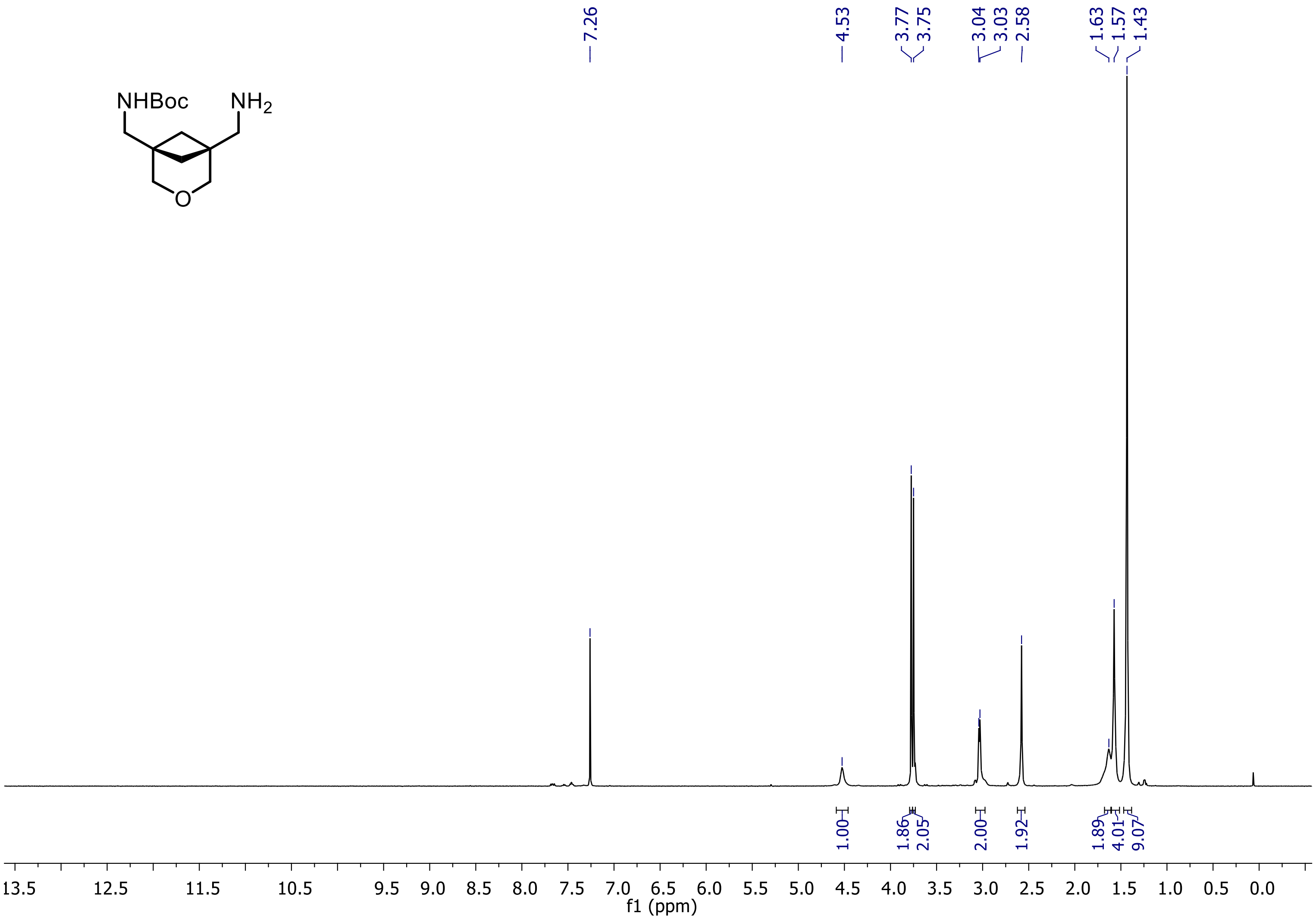

$^{13}C\{^1H\}$ NMR (151 MHz, $CDCl_3$)

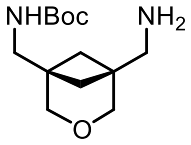

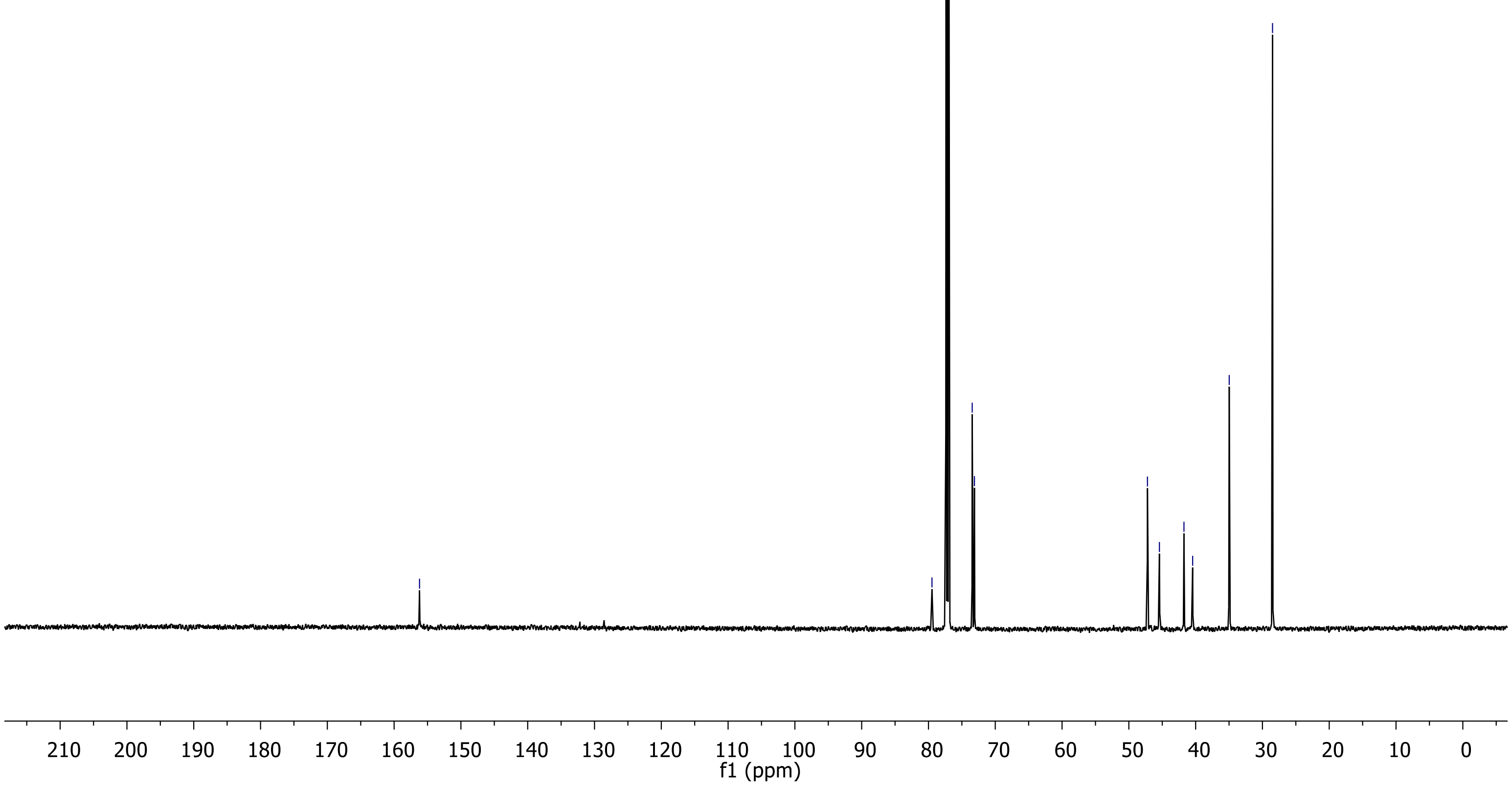

Compound 35

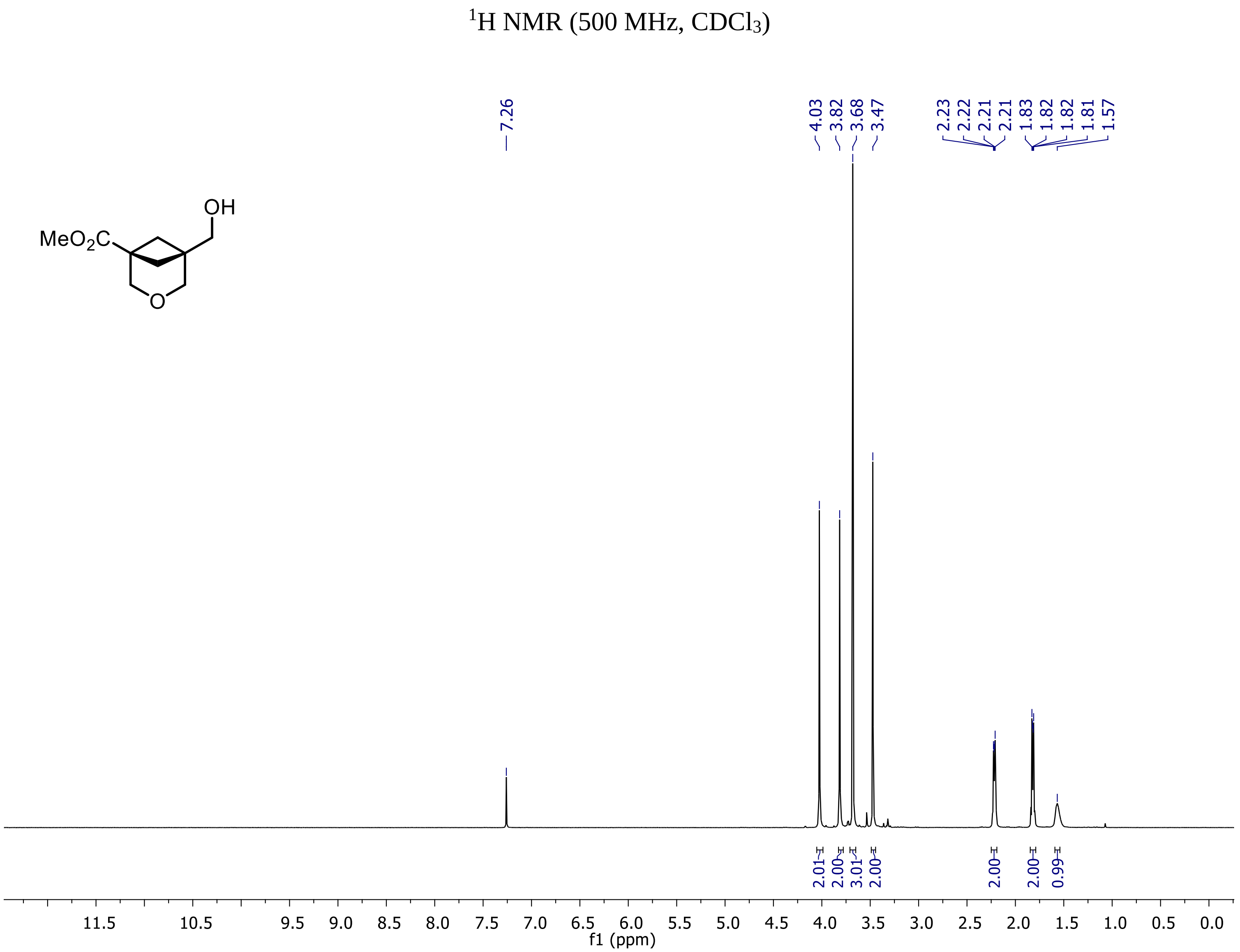

^{1}H NMR (500 MHz, CDCl$_3$)
7.26
4.03
3.82
3.68
3.47
2.23
2.22
2.21
2.21
1.83
1.82
1.82
1.81
1.57
2.01
2.00
3.01
2.00
2.00
2.00
0.99
11.5
10.5
9.5
9.0
8.5
8.0
7.5
7.0
6.5
6.0
5.5
5.0
4.5
4.0
3.5
3.0
2.5
2.0
1.5
1.0
0.5
0.0
f1 (ppm)

$^{13}C\{^1H\}$ NMR (126 MHz, $CDCl_3$)

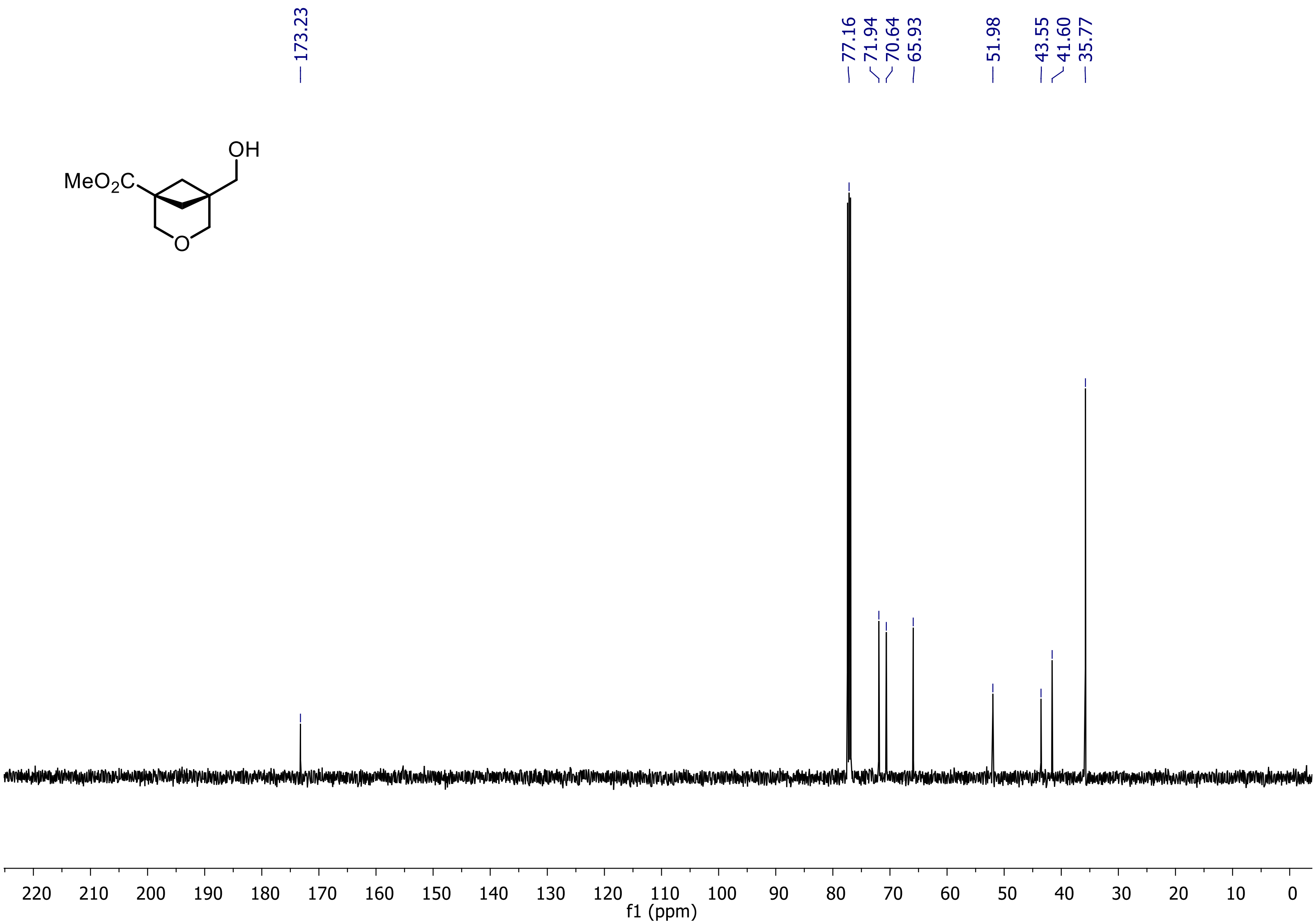

Compound 36

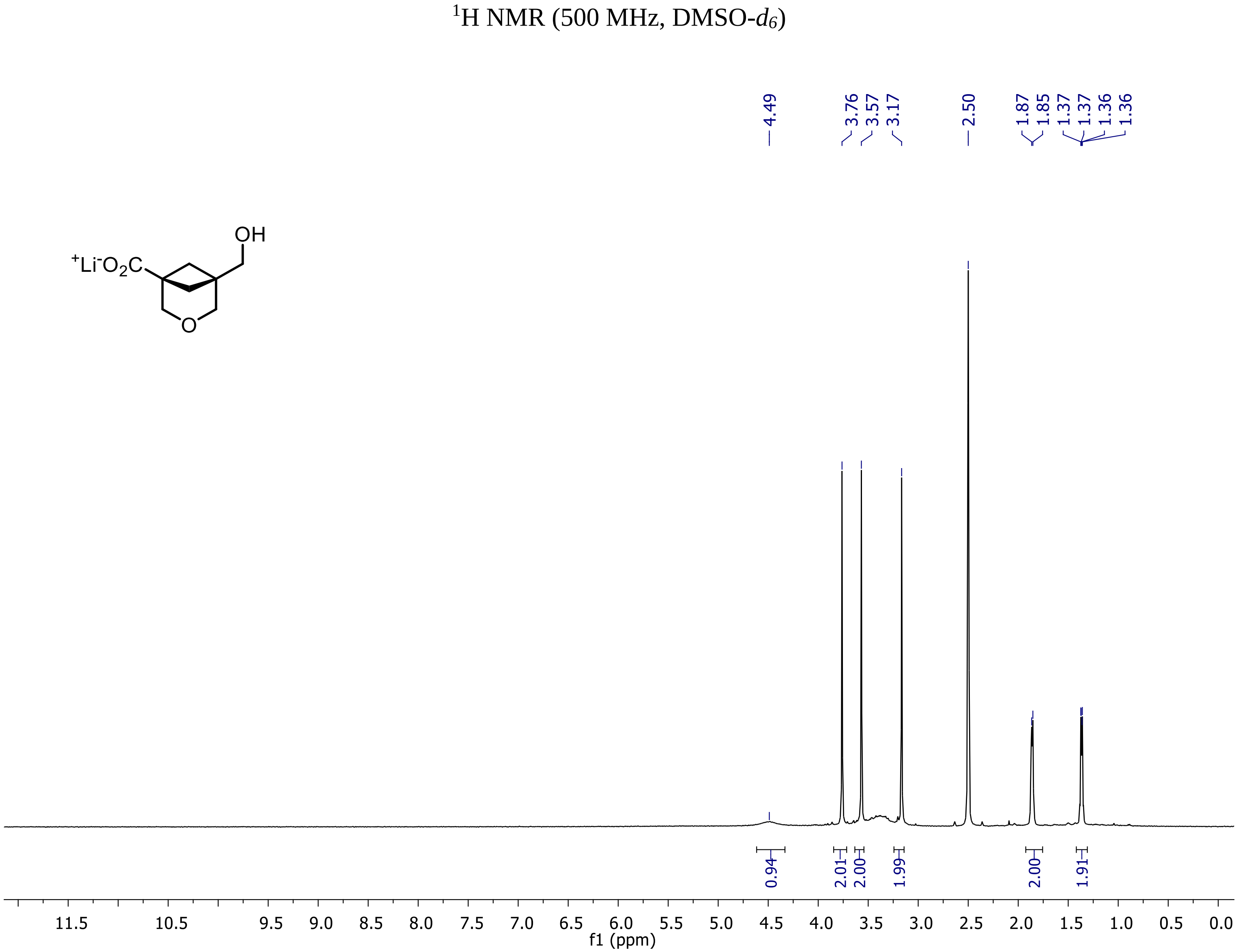

^{1}H NMR (500 MHz, DMSO-d_6)
4.49
3.76
3.57
3.17
2.50
1.87
1.85
1.37
1.37
1.36
1.36
0.94
2.01
2.00
1.99
2.00
1.91
11.5
10.5
9.5
9.0
8.5
8.0
7.5
7.0
6.5
6.0
5.5
5.0
4.5
4.0
3.5
3.0
2.5
2.0
1.5
1.0
0.5
0.0
f1 (ppm)
OH
+Li-O2C
O

$^{13}C\{^{1}H\}$ NMR (151 MHz, DMSO-d_6)

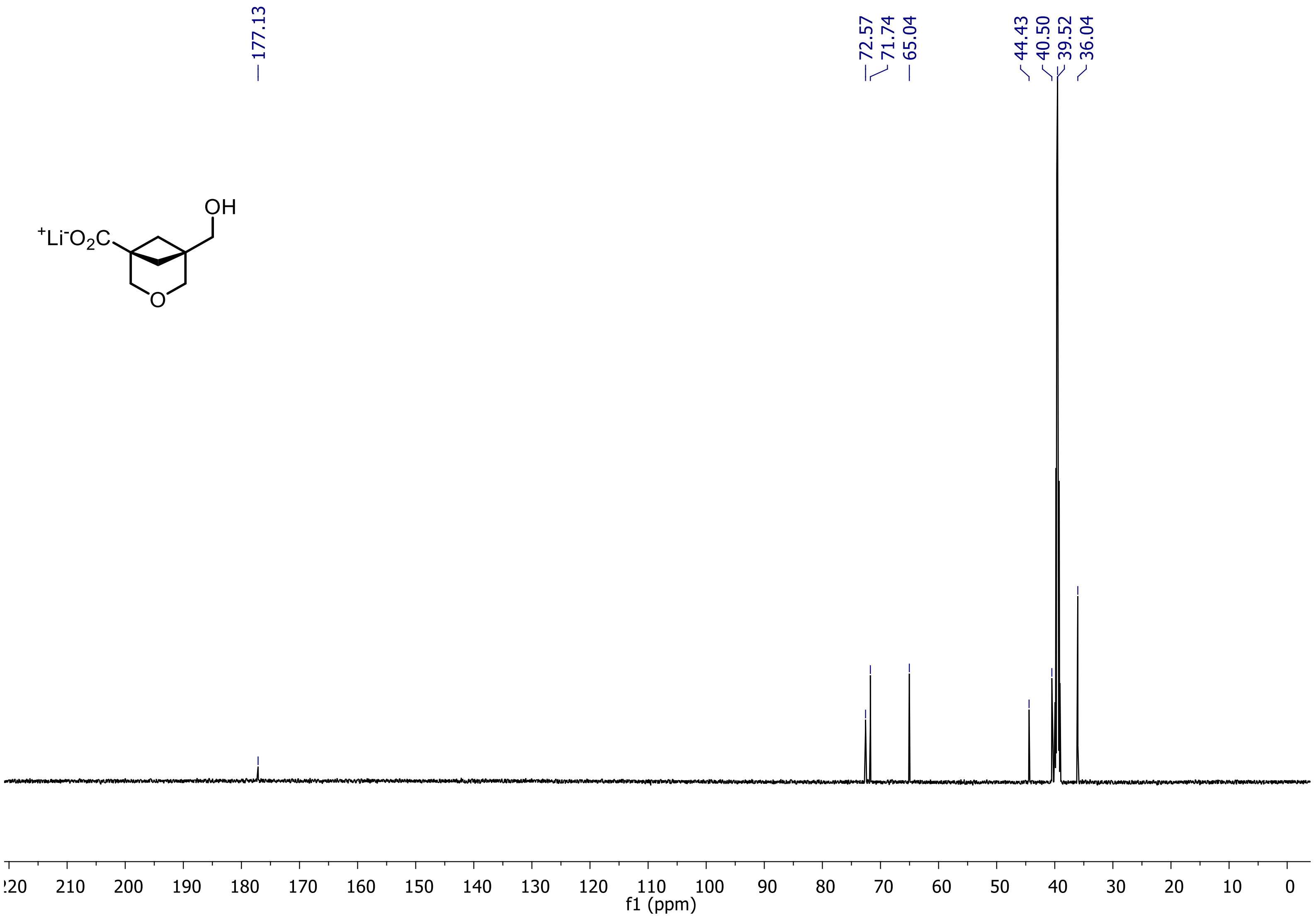

Compound 37

^{1}H NMR (500 MHz, $CDCl_3$)

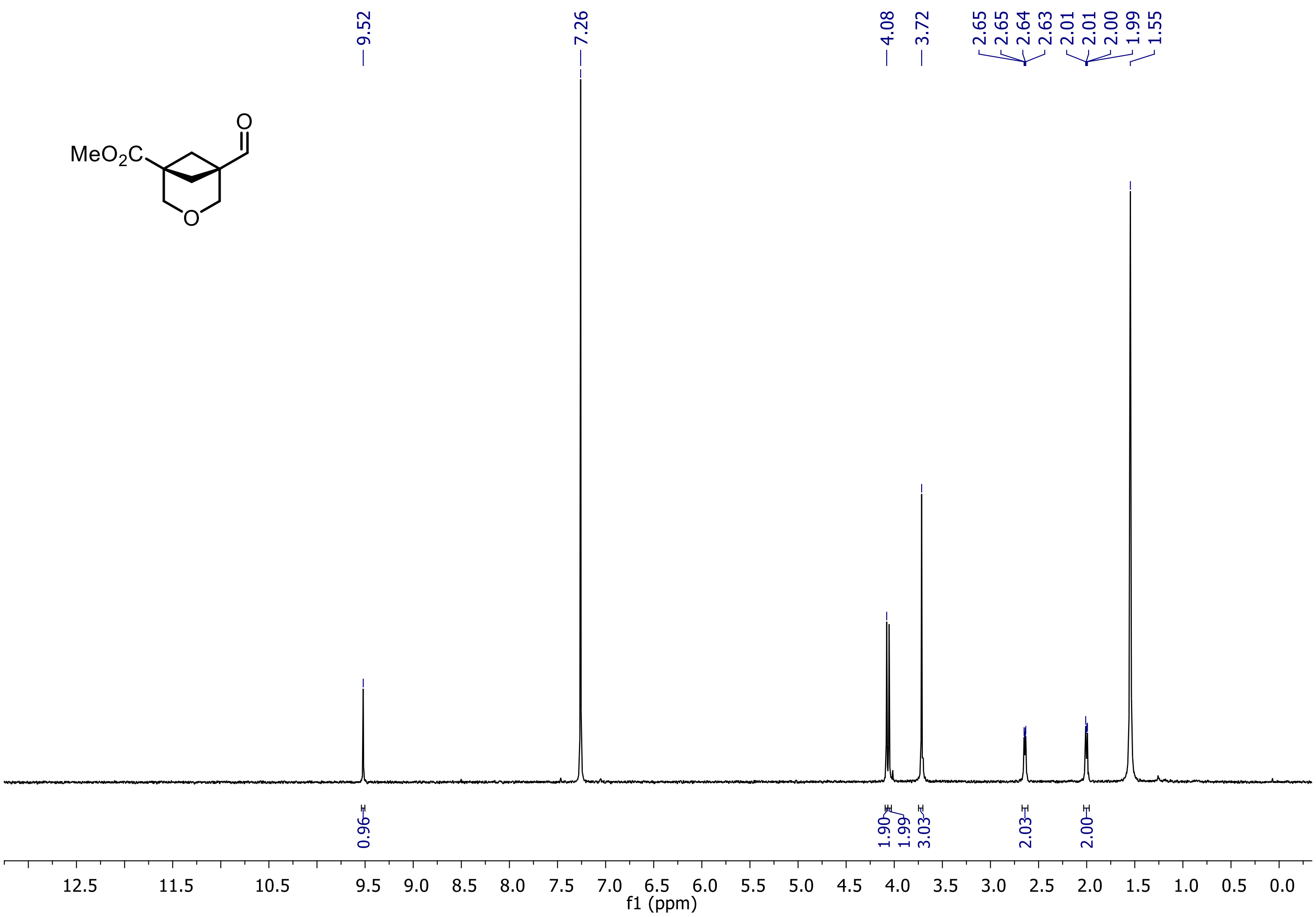

$^{13}C\{^1H\}$ NMR (126 MHz, $CDCl_3$)

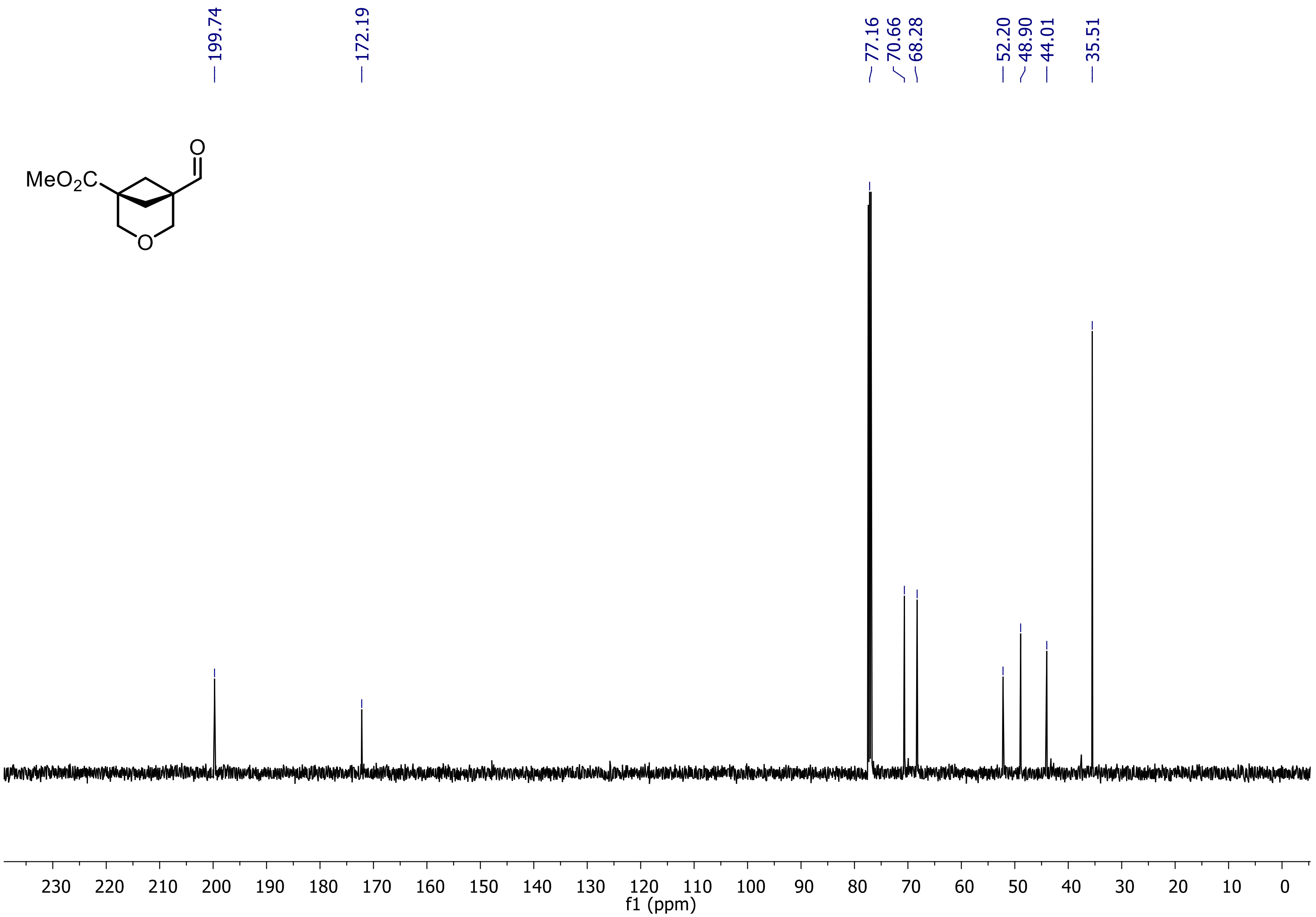

Compound 38

^{1}H NMR (500 MHz, $CDCl_3$)

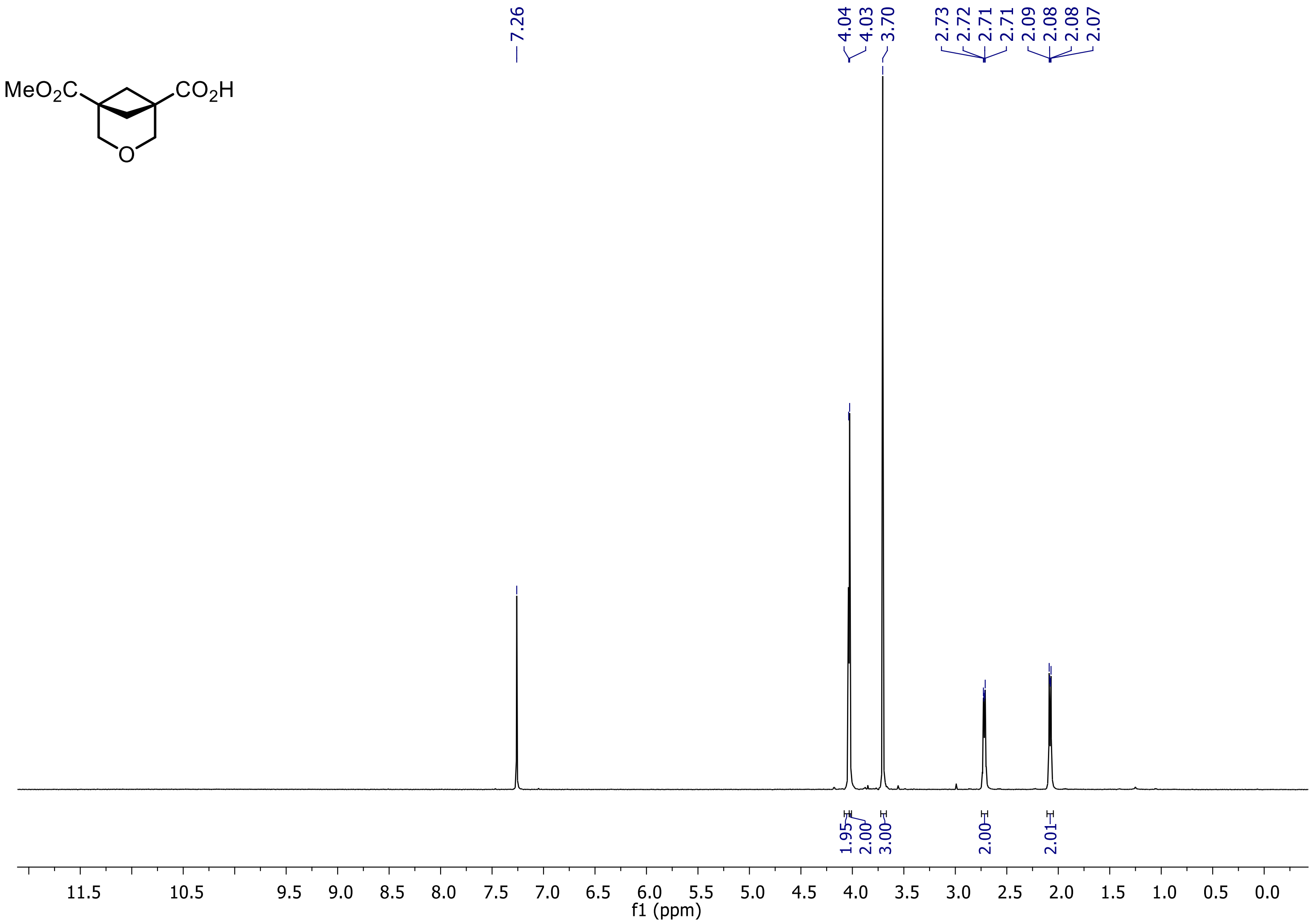

$^{13}C\{^{1}H\}$ NMR (126 MHz, $CDCl_3$)

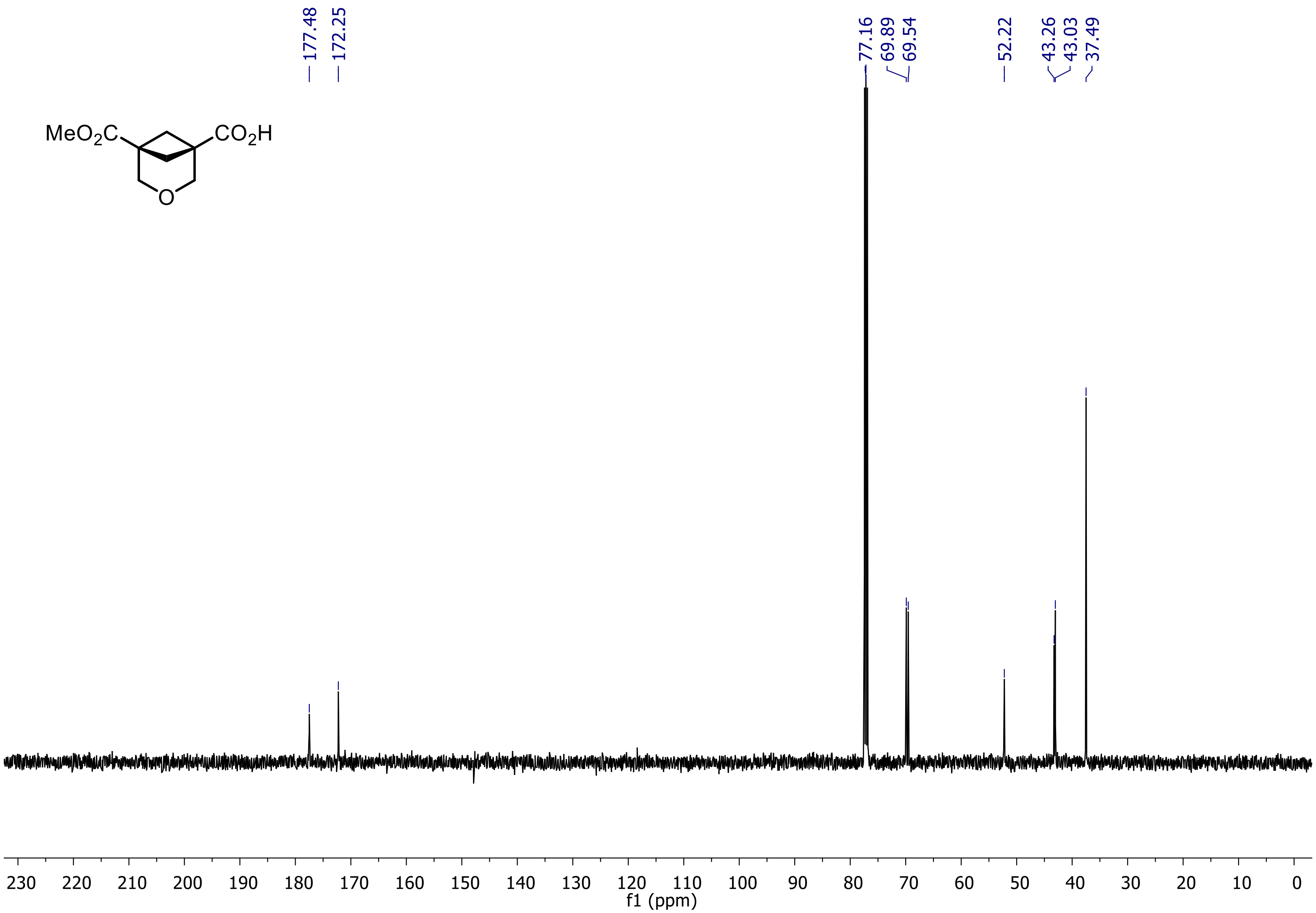

Compound 39

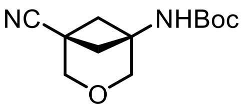

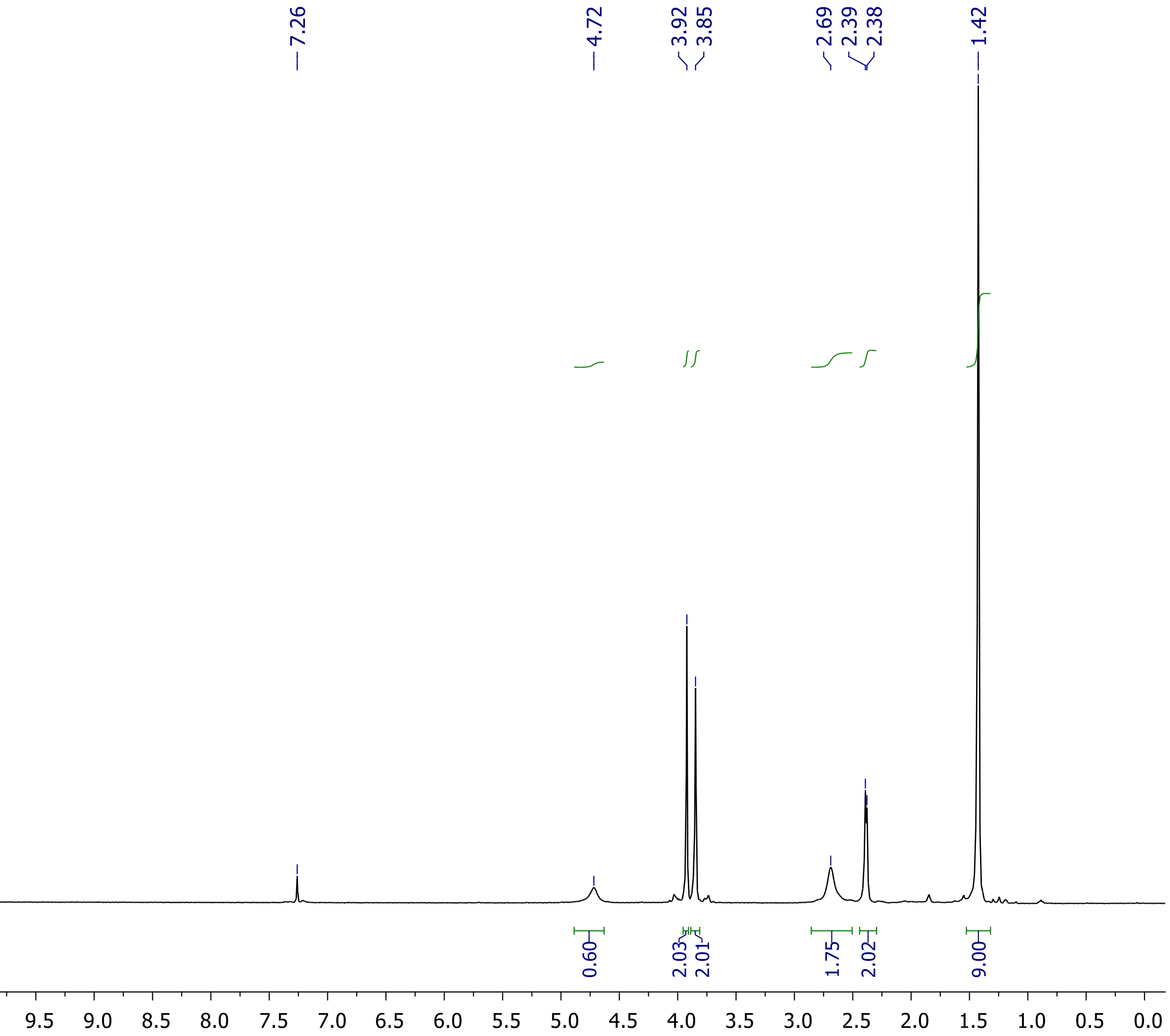
^{1}H NMR (500 MHz, CDCl$_3$)
BH089005-26
7.26
4.72
3.92
3.85
2.69
2.39
2.38
1.42
0.60
2.03
2.01
1.75
2.02
9.00
11.5
10.5
9.5
9.0
8.5
8.0
7.5
7.0
6.5
6.0
5.5
5.0
4.5
4.0
3.5
3.0
2.5
2.0
1.5
1.0
0.5
0.0

$^{13}C\{^1H\}$ NMR (101 MHz, $CDCl_3$)

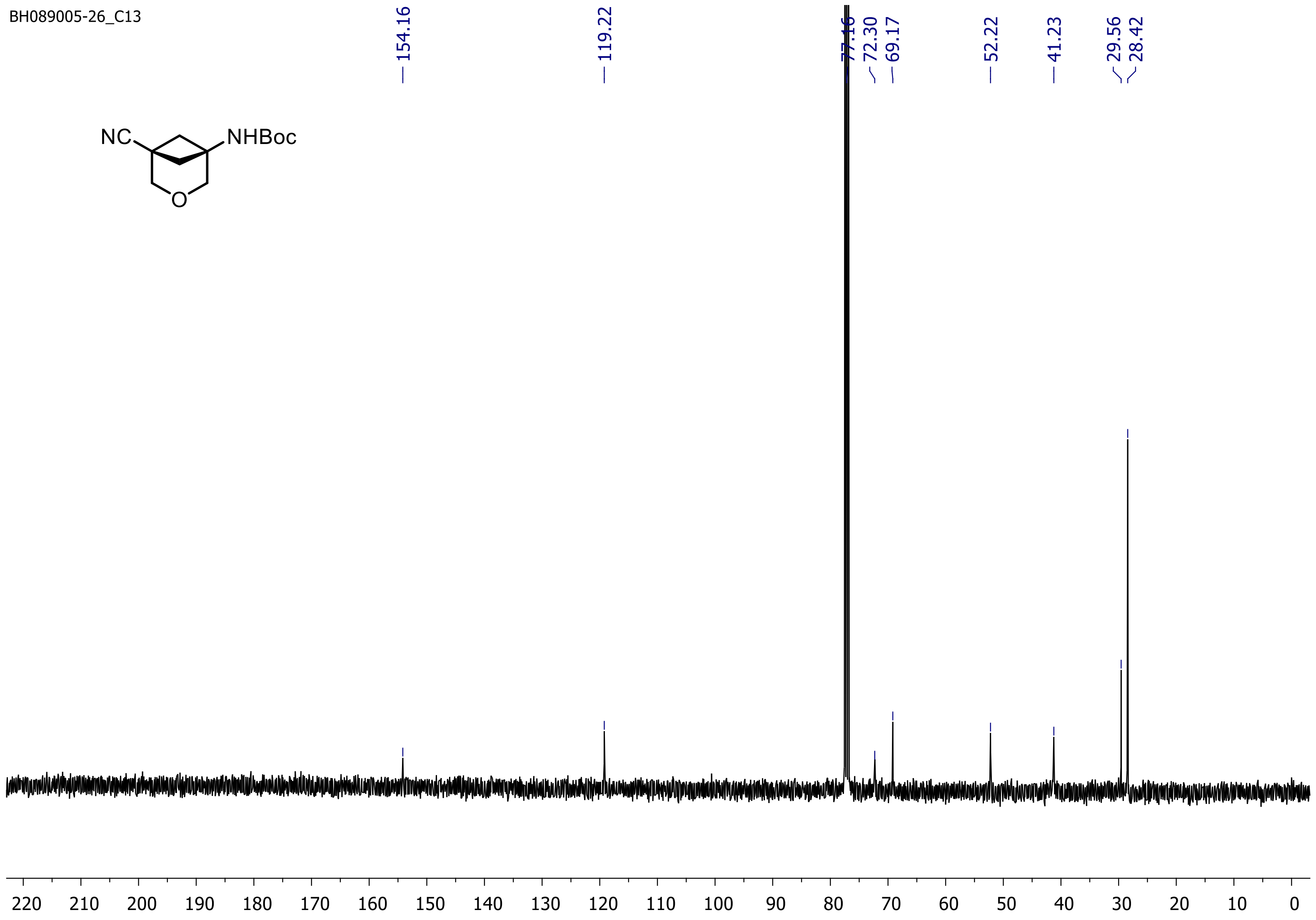

Compound 40

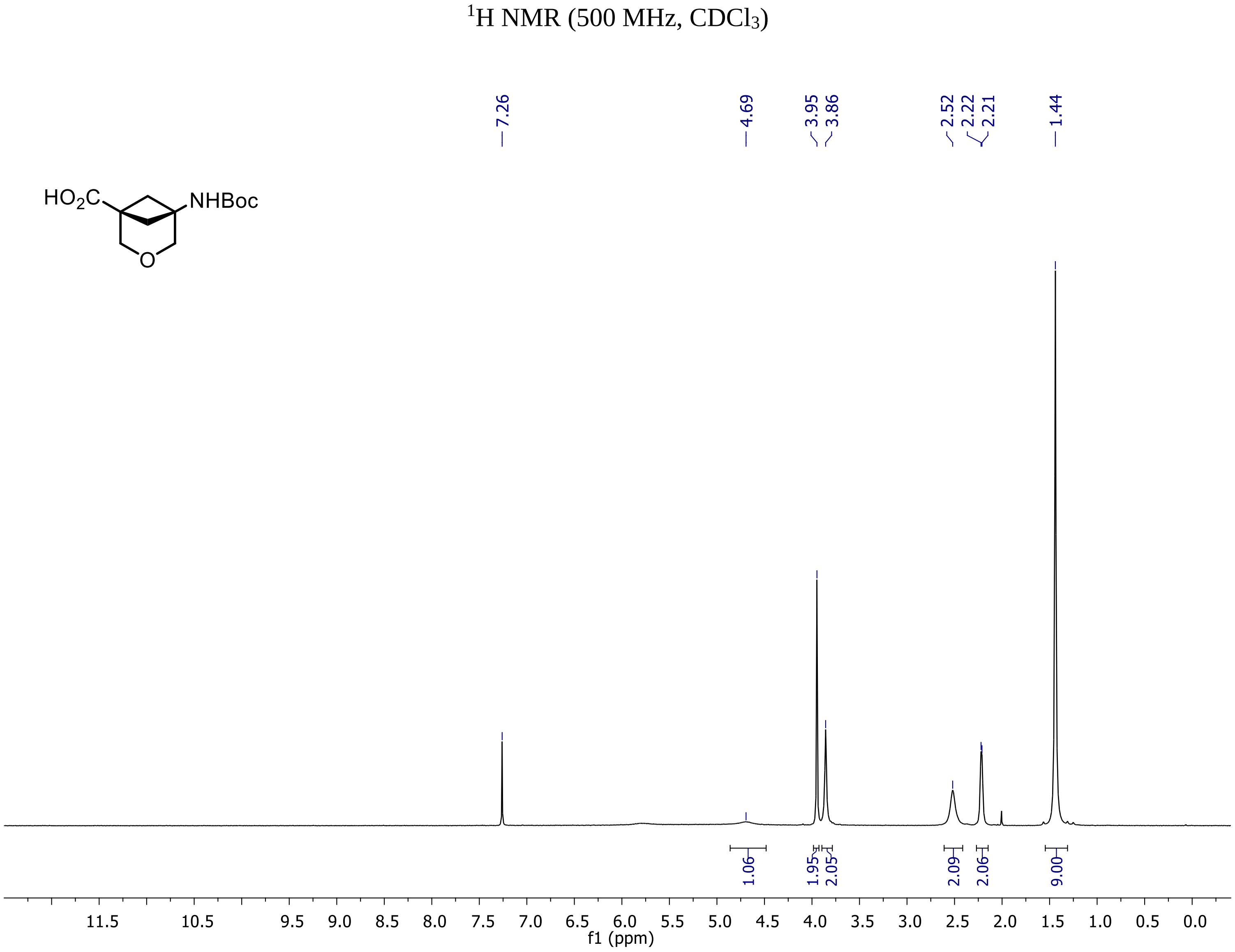

^{1}H NMR (500 MHz, CDCl$_3$)
HO$_2$C
NHBoc
O
7.26
4.69
3.95
3.86
2.52
2.22
2.21
1.44
1.06
1.95
2.05
2.09
2.06
9.00
11.5 10.5 9.5 9.0 8.5 8.0 7.5 7.0 6.5 6.0 5.5 5.0 4.5 4.0 3.5 3.0 2.5 2.0 1.5 1.0 0.5 0.0
f1 (ppm)

$^{13}C\{^{1}H\}$ NMR (151 MHz, DMSO-d_6)

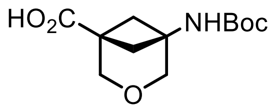

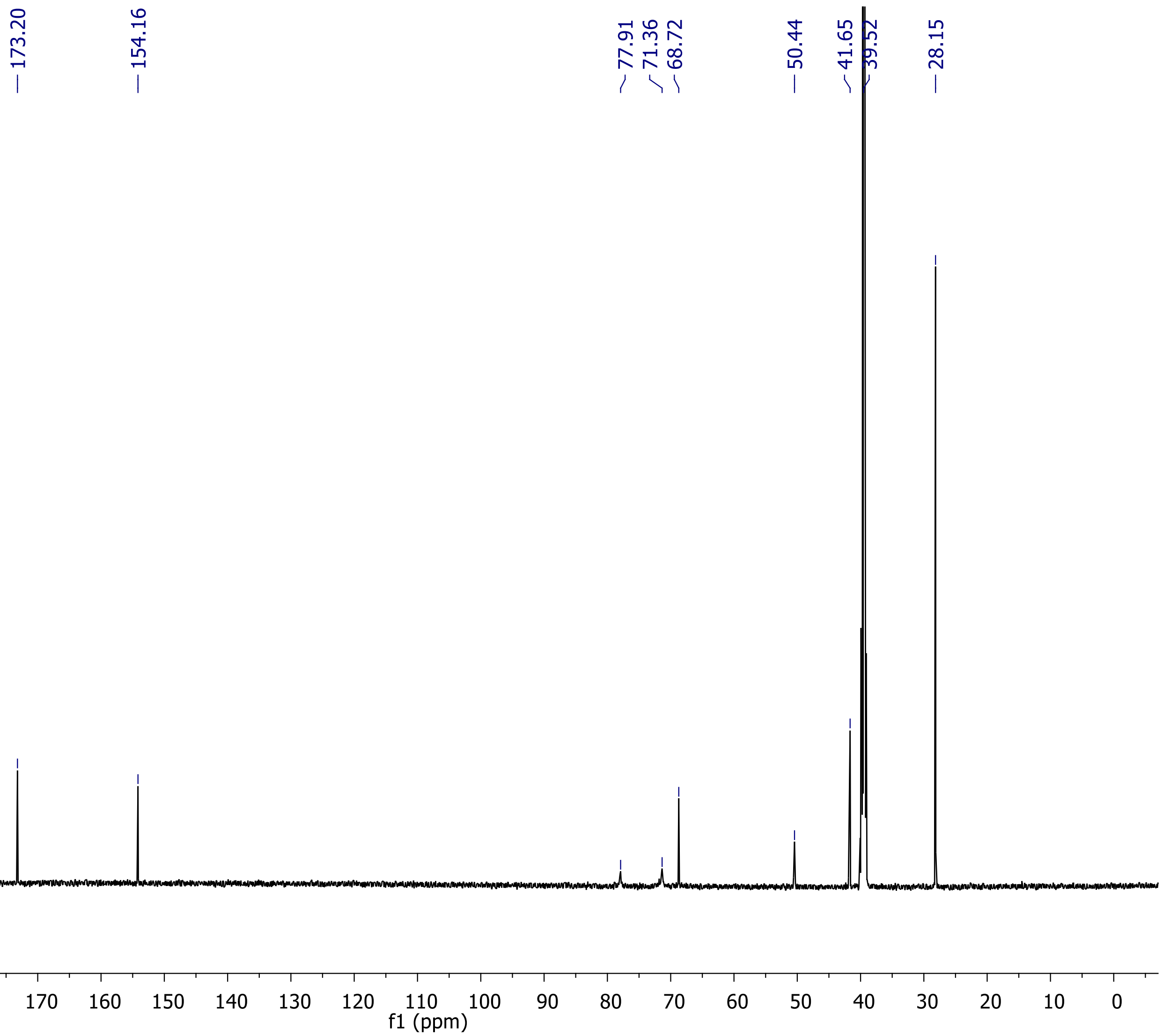

Compound 41

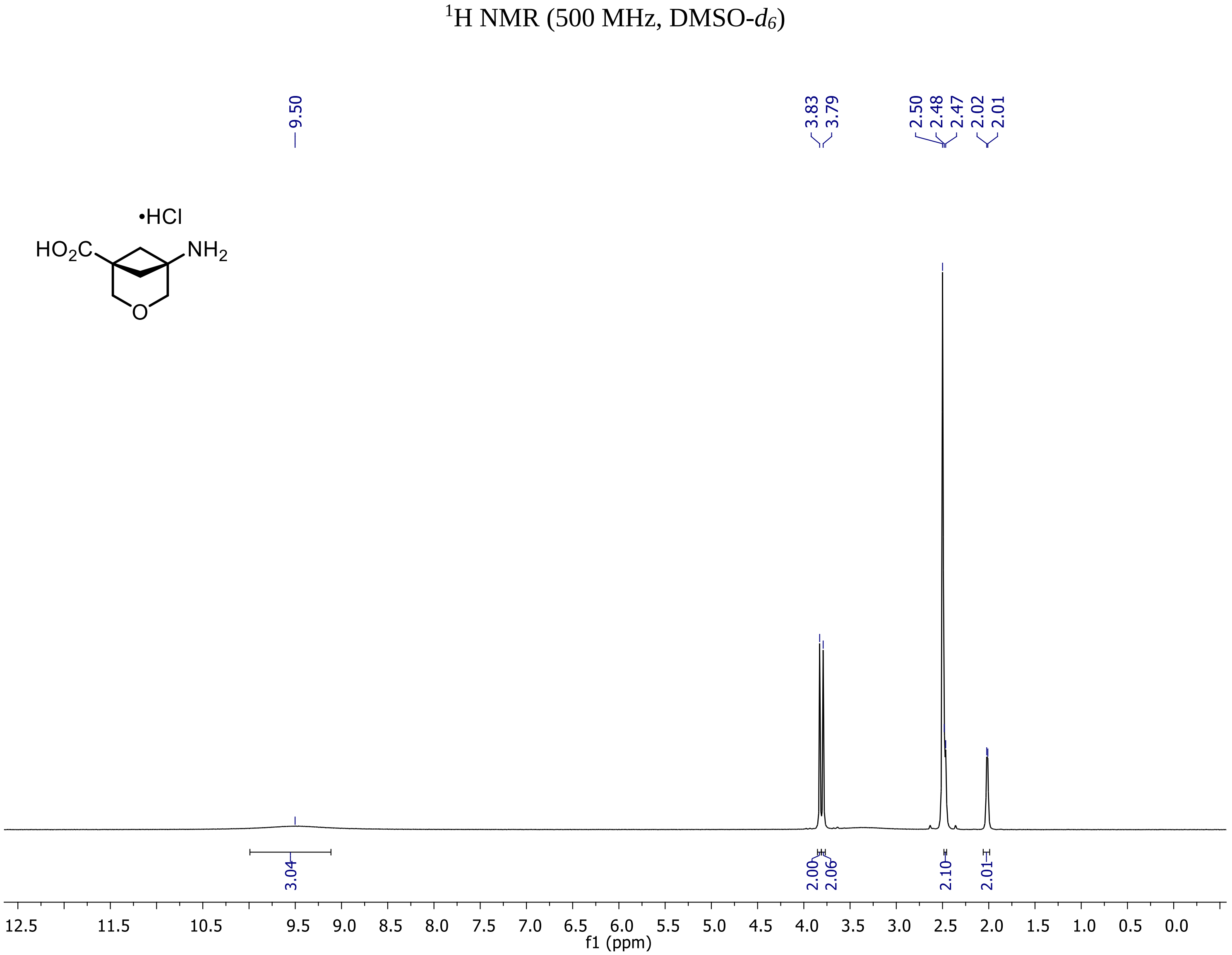

^{1}H NMR (500 MHz, DMSO-d_6)
9.50
3.83
3.79
2.50
2.48
2.47
2.02
2.01
•HCl
HO2C
NH2
O
3.04
2.00
2.06
2.10
2.01
12.5
11.5
10.5
9.5
9.0
8.5
8.0
7.5
7.0
6.5
6.0
5.5
5.0
4.5
4.0
3.5
3.0
2.5
2.0
1.5
1.0
0.5
0.0
f1 (ppm)

$^{13}C\{^{1}H\}$ NMR (151 MHz, DMSO-d_6)

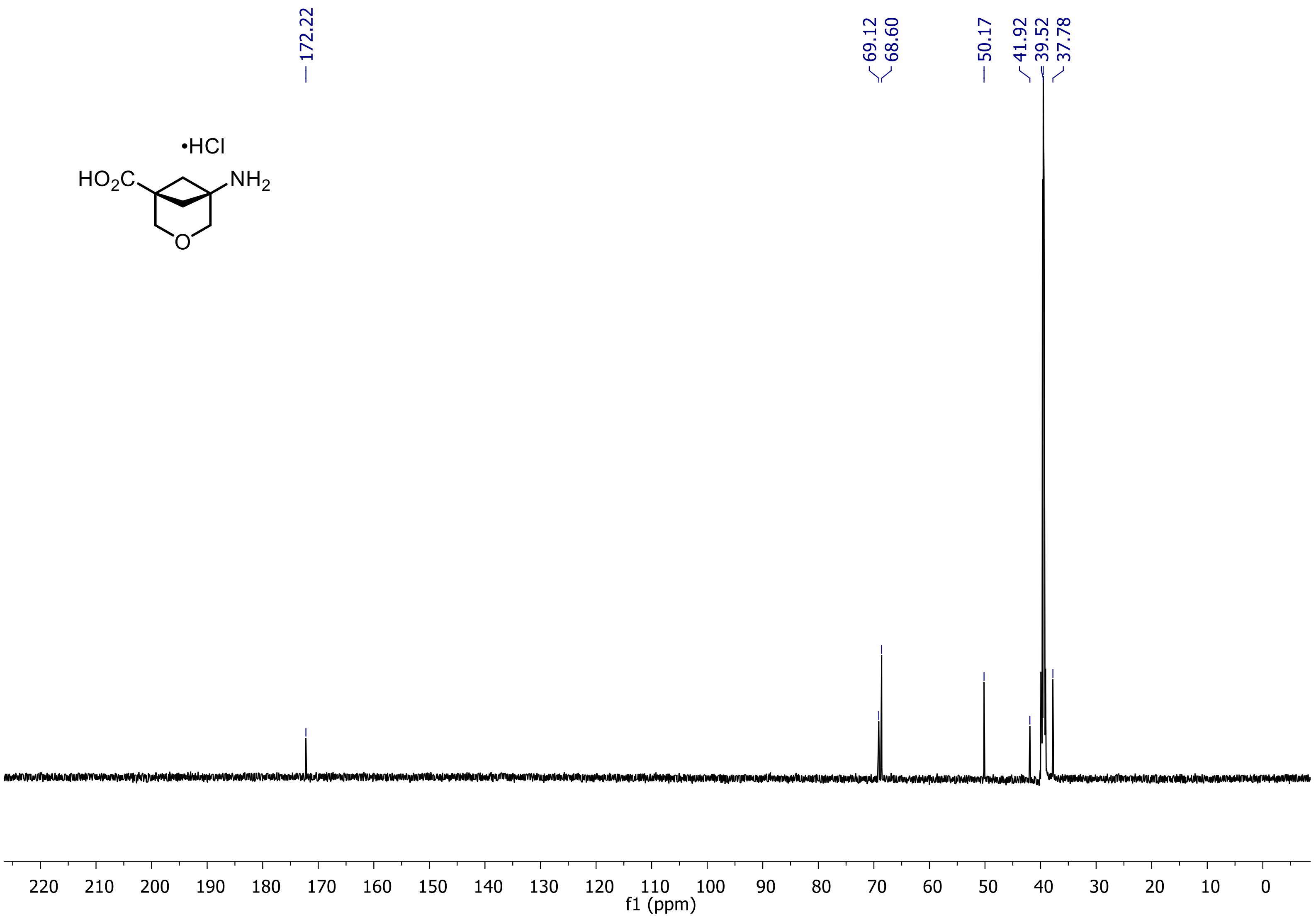

Compound 42

^{1}H NMR (500 MHz, $CDCl_3$)

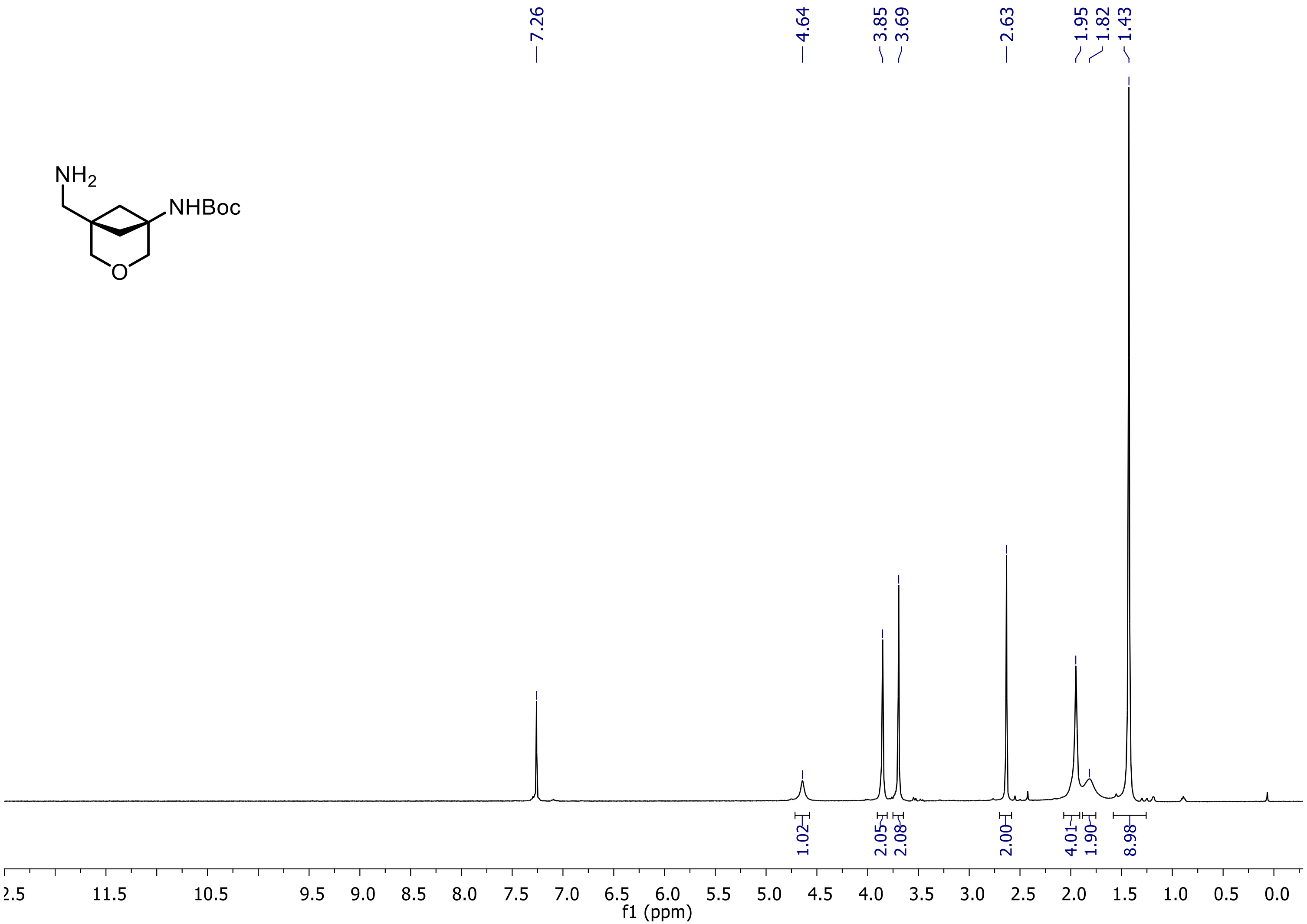

$^{13}C\{^1H\}$ NMR (126 MHz, $CDCl_3$)

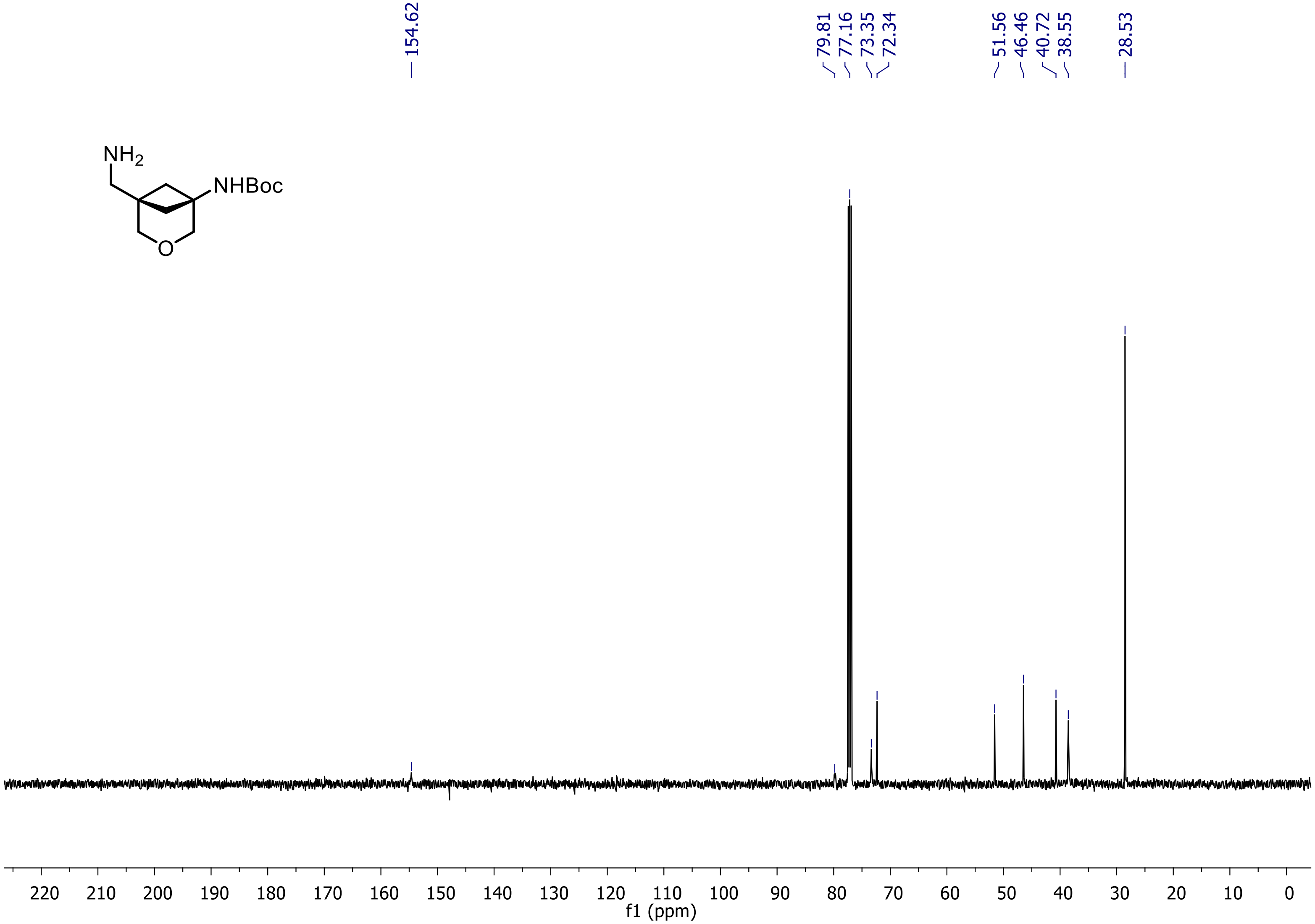

Compound 43

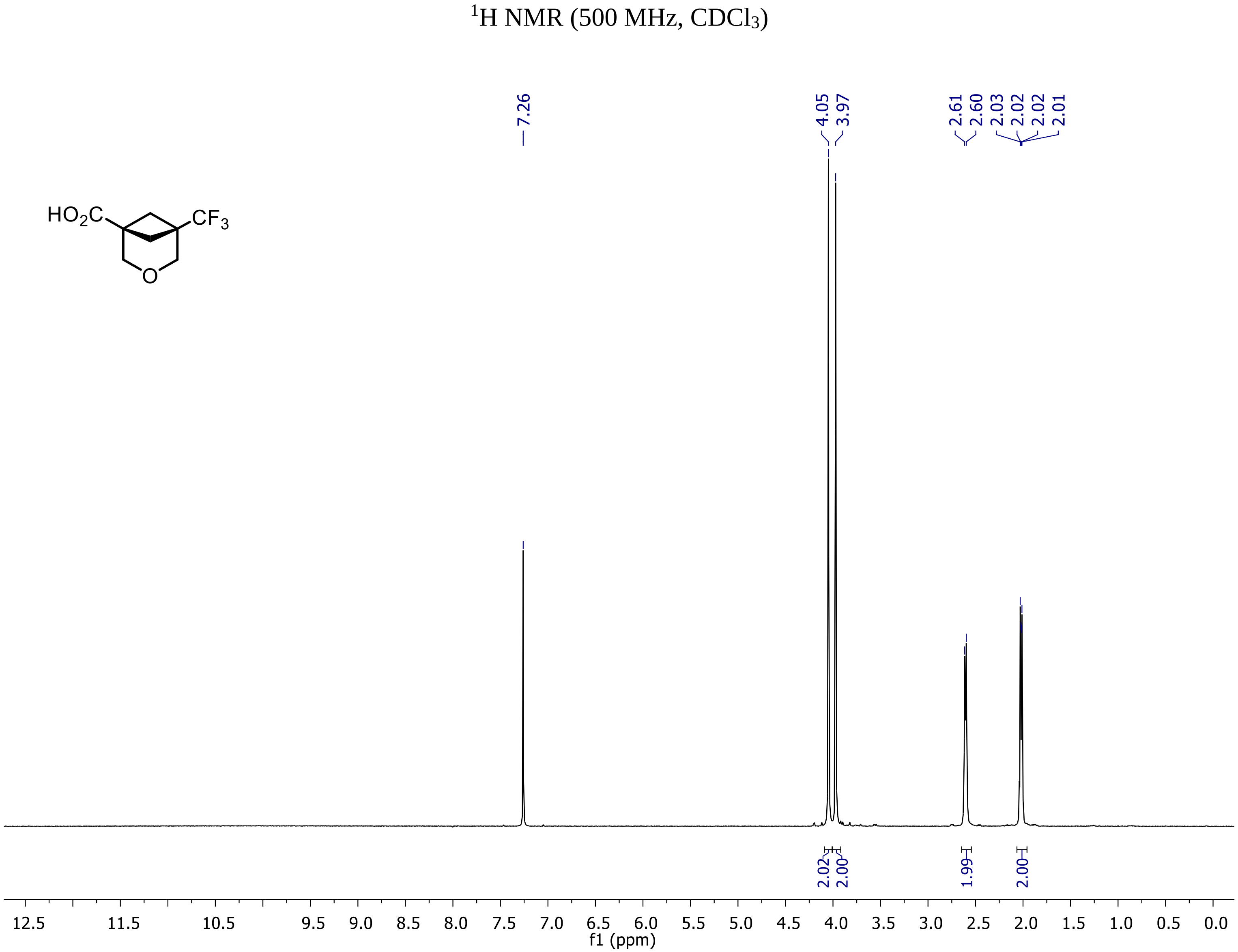

HO2C
CF3
O
^{1}H NMR (500 MHz, CDCl$_3$)
7.26
4.05
3.97
2.61
2.60
2.03
2.02
2.02
2.01
2.02
2.00
1.99
2.00
12.5
11.5
10.5
9.5
9.0
8.5
8.0
7.5
7.0
6.5
6.0
5.5
5.0
4.5
4.0
3.5
3.0
2.5
2.0
1.5
1.0
0.5
0.0
f1 (ppm)

$^{13}C\{^1H\}$ NMR (151 MHz, $CDCl_3$)

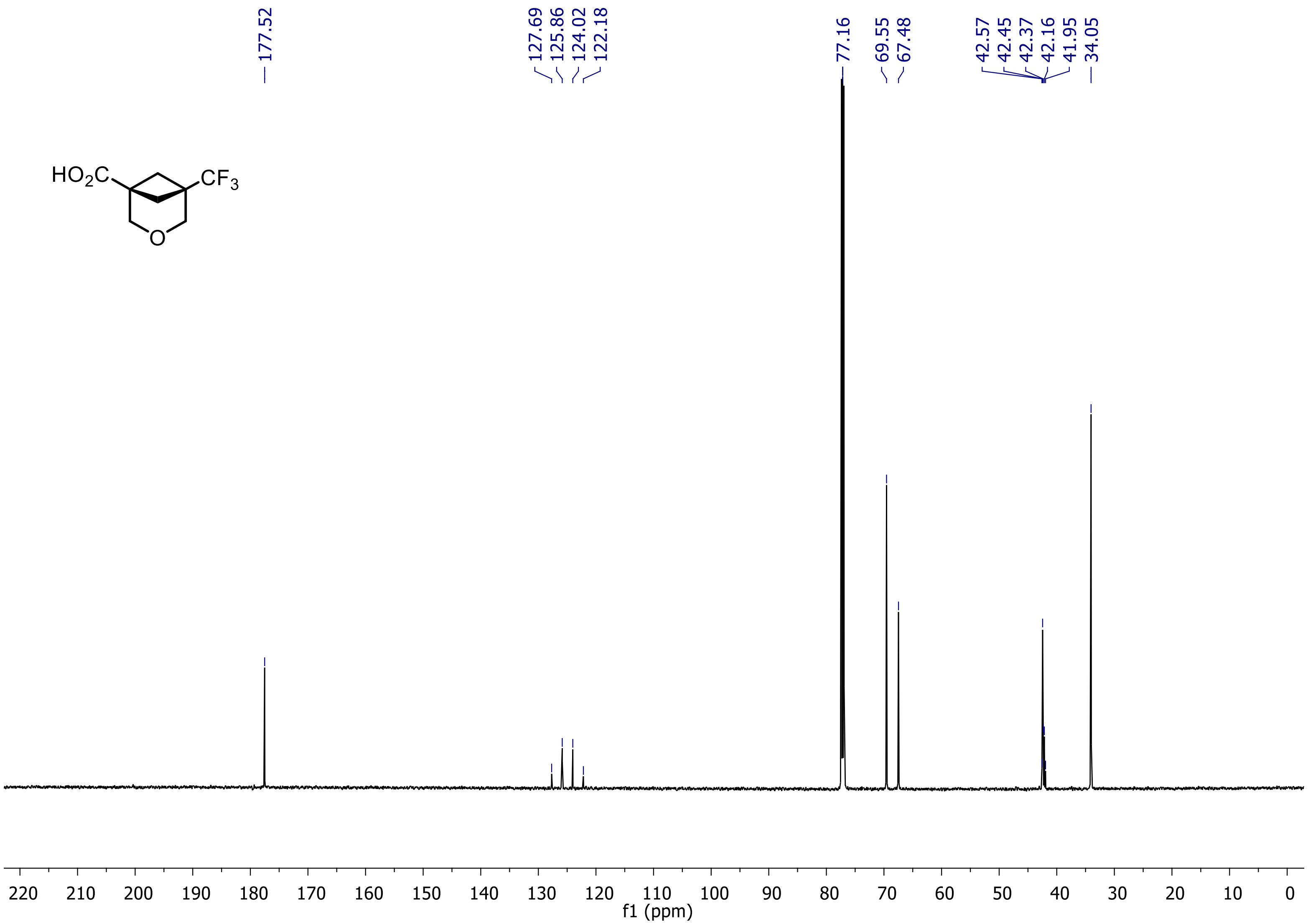

$^{19}F\{^{1}H\}$ NMR (376 MHz, $CDCl_3$)

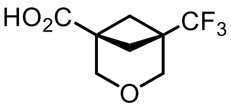

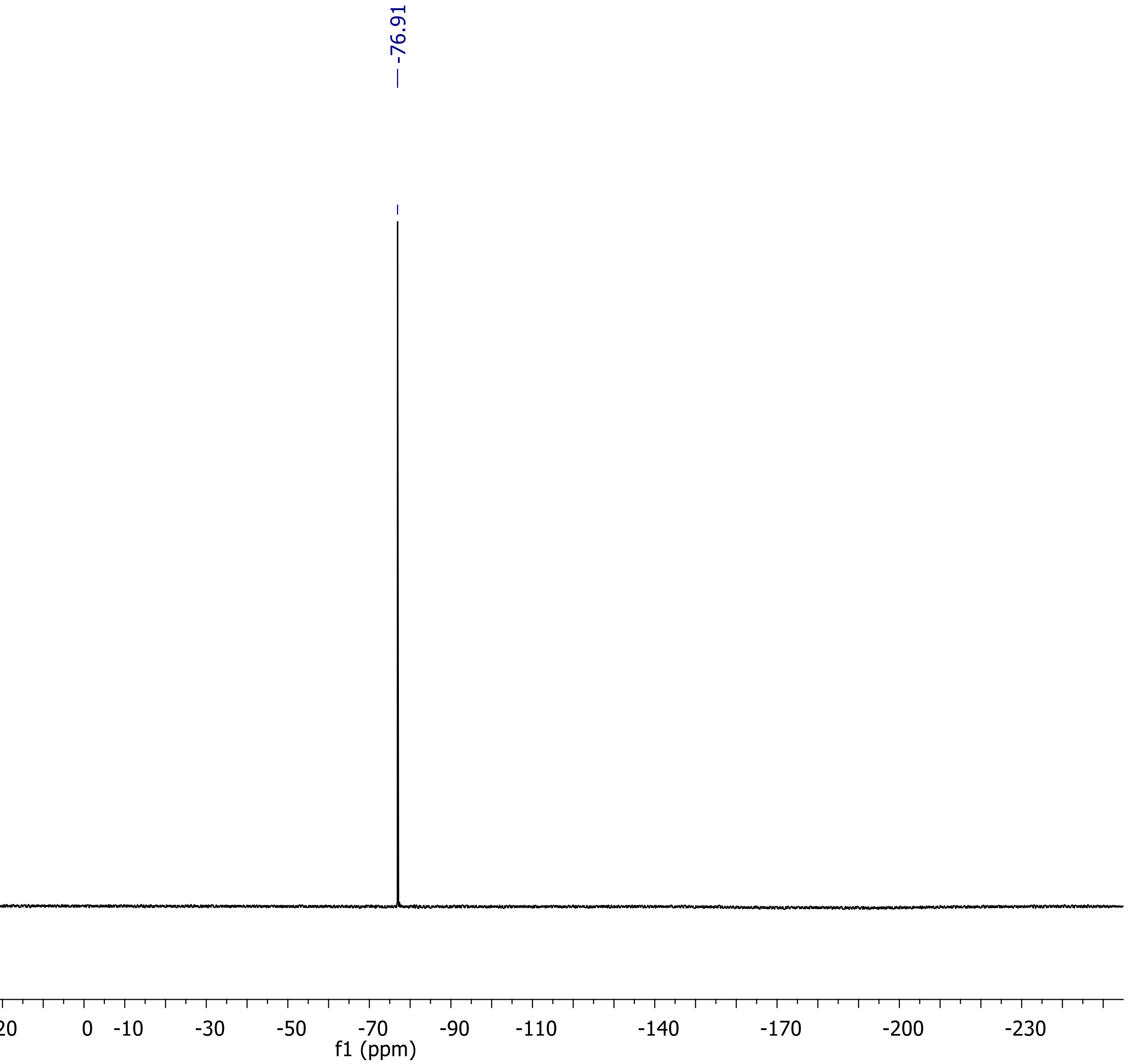

Compound 44

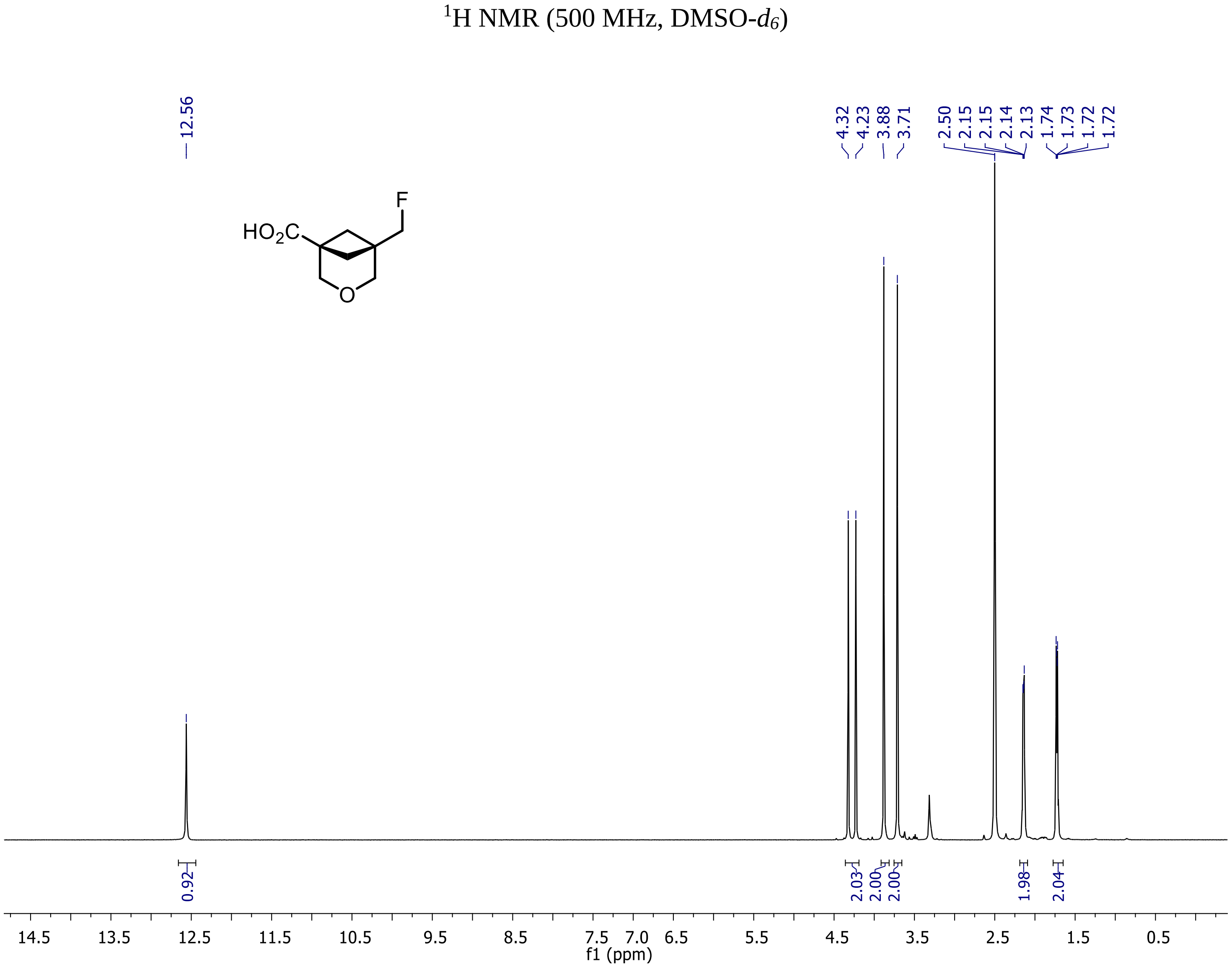

¹H NMR (500 MHz, DMSO-d6)
12.56
4.32
4.23
3.88
3.71
2.50
2.15
2.15
2.14
2.13
1.74
1.73
1.72
1.72
HO2C
F
O
0.92
2.03
2.00
2.00
1.98
2.04
14.5
13.5
12.5
11.5
10.5
9.5
8.5
7.5
7.0
6.5
5.5
4.5
3.5
2.5
1.5
0.5
f1 (ppm)

$^{13}C\{^1H\}$ NMR (151 MHz, DMSO-d_6)

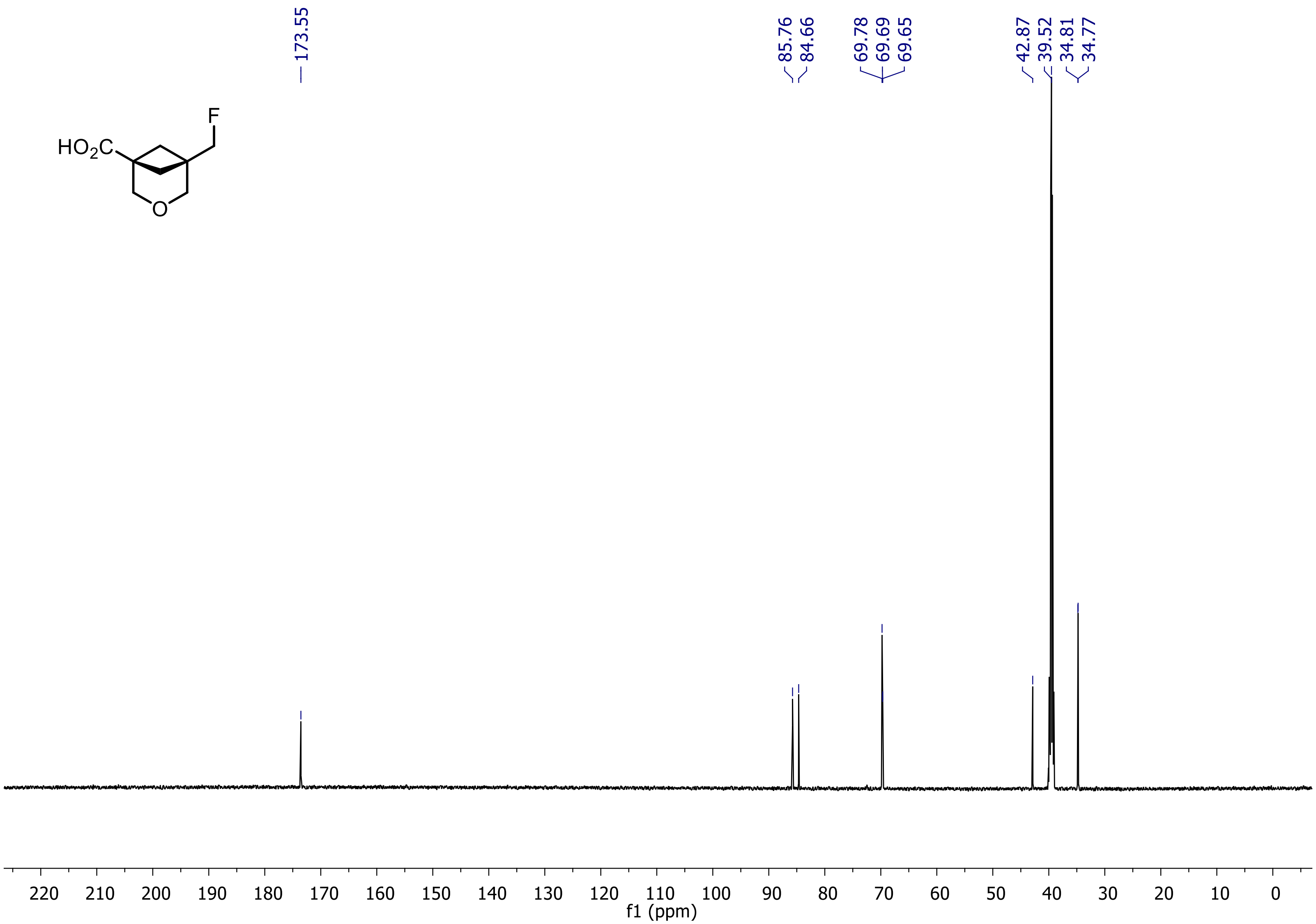

$^{19}F\{^{1}H\}$ NMR (376 MHz, DMSO-d_6)

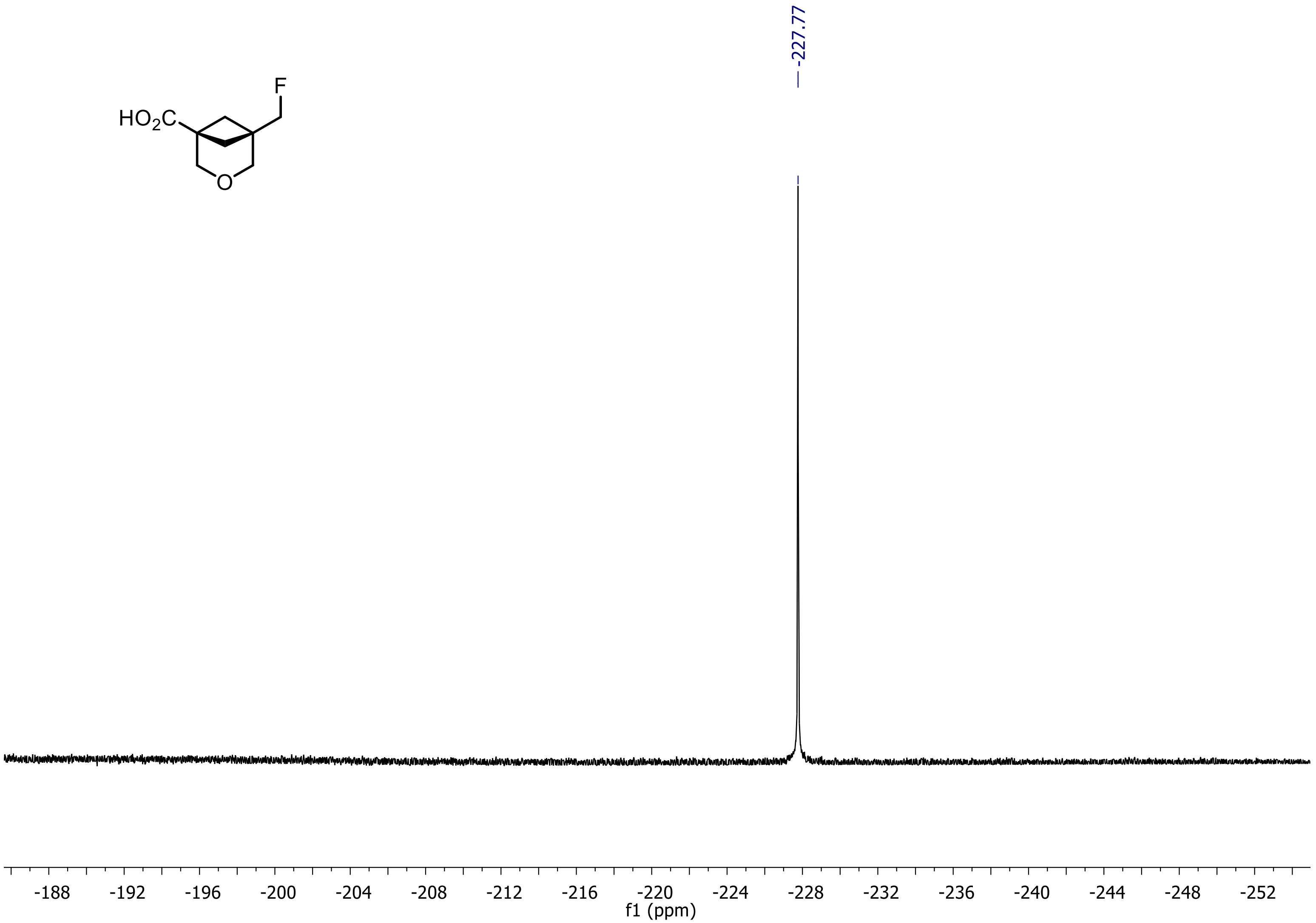

Compound 51

^{1}H NMR (500 MHz, DMSO-d_6)

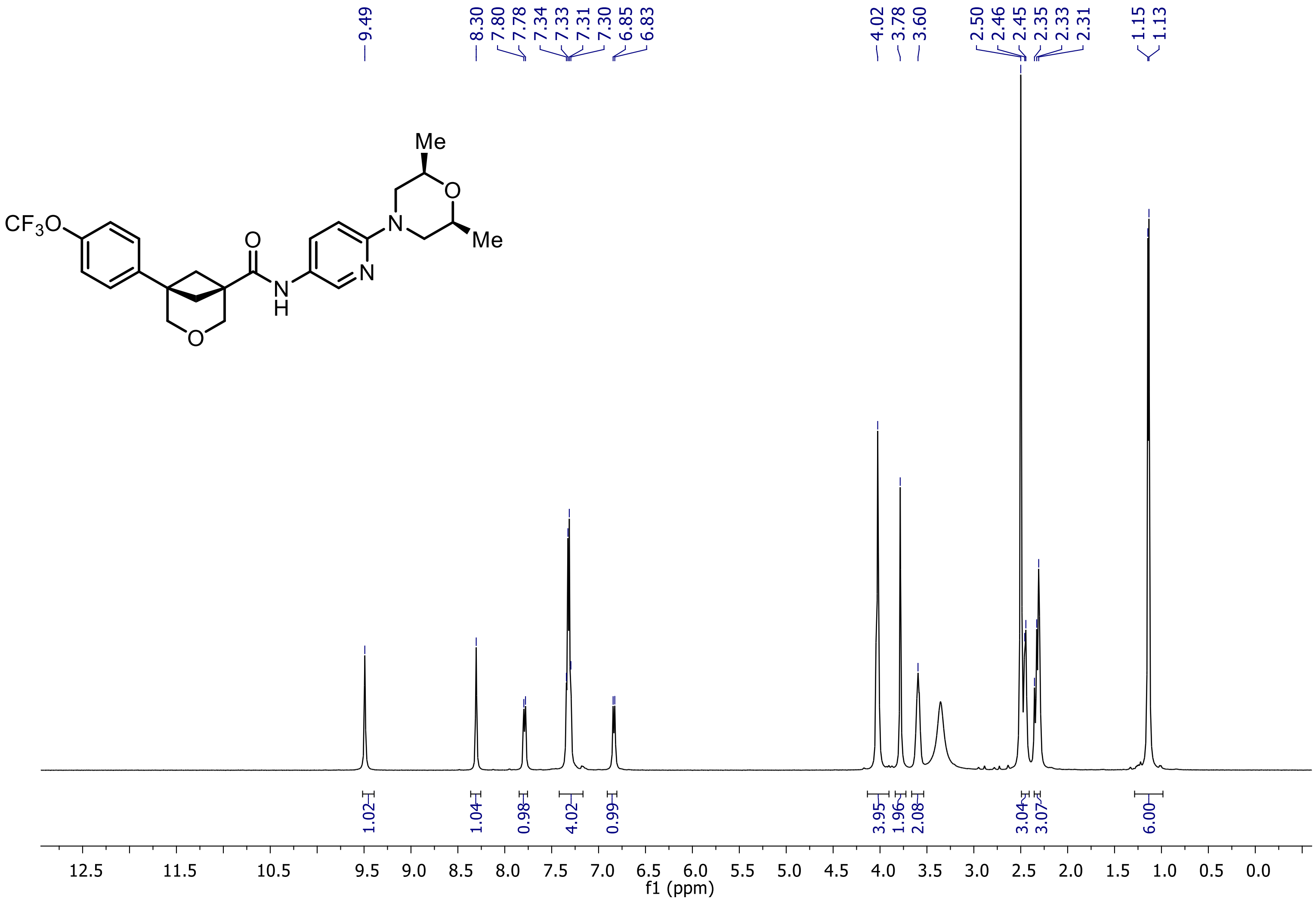

$^{13}C\{^{1}H\}$ NMR (151 MHz, DMSO-d_6)

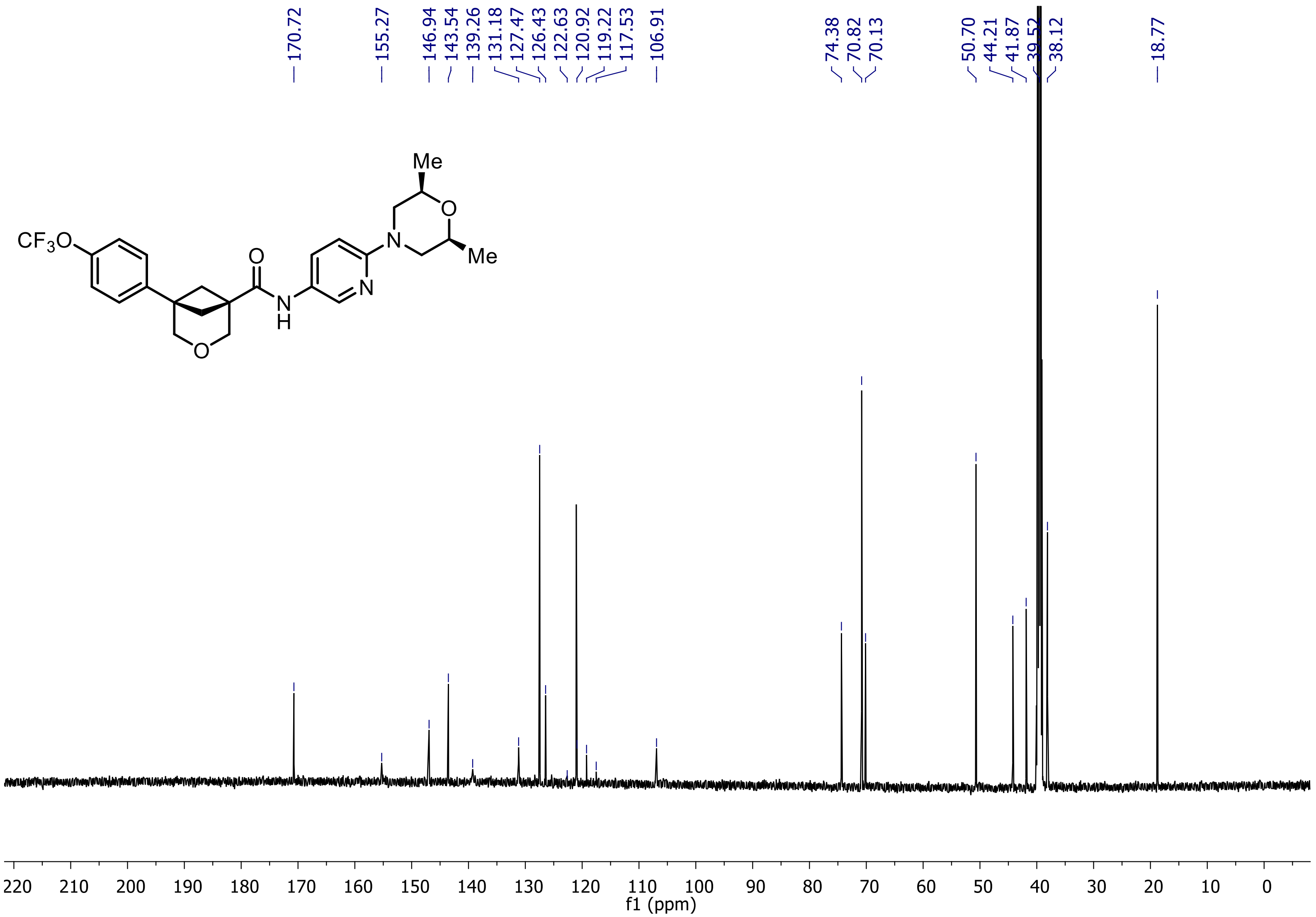

$^{19}F\{^{1}H\}$ NMR (376 MHz, DMSO-d_6)

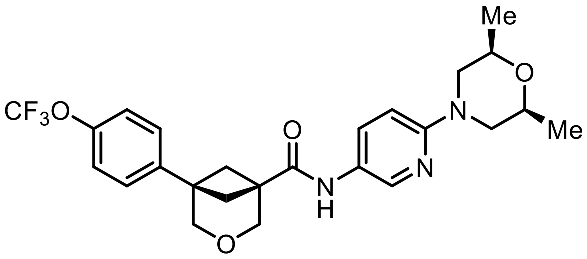

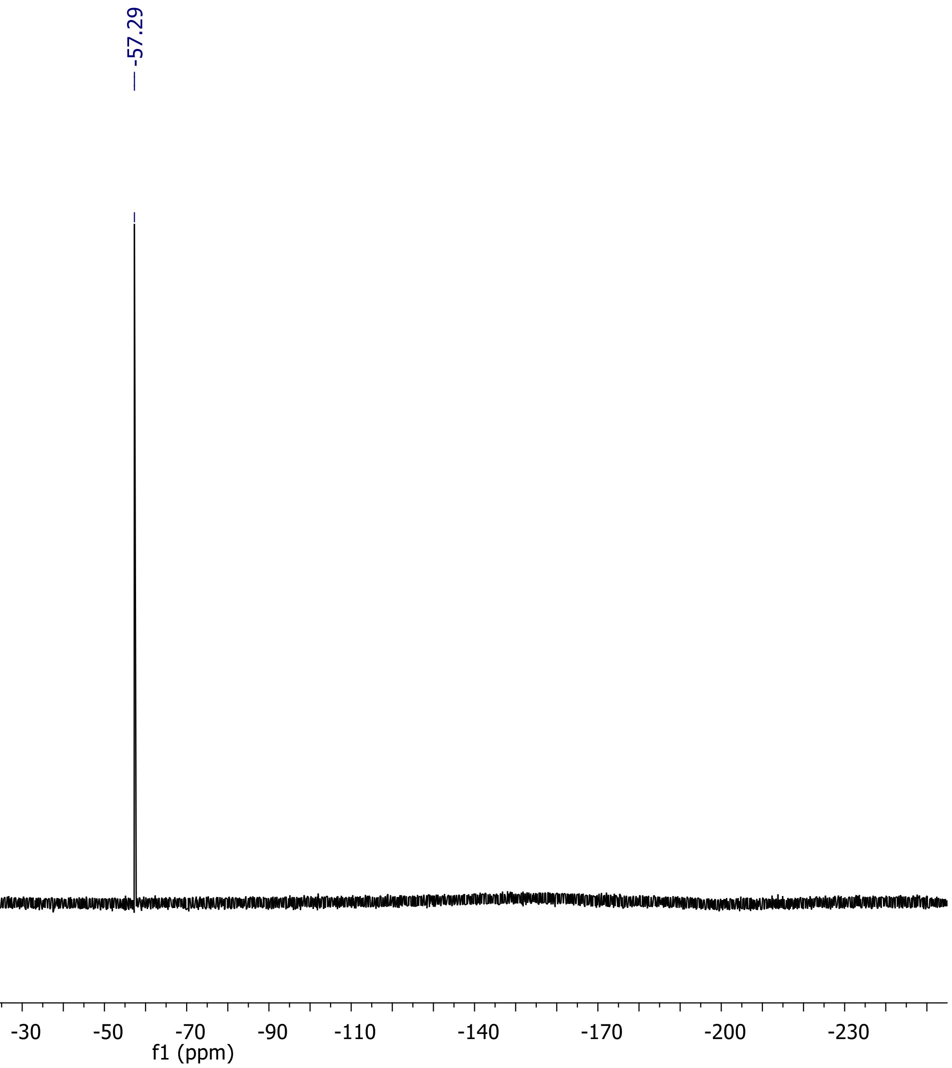

6. Crystallographic Data (X-ray)

X-Ray Structure Determinations of 15a, 8b, 10b, 11b, and 44

Crystals of compounds **15a**, **8b**, **10b**, **11b**, and **44** suitable for X-ray diffraction studies were obtained by a low evaporation of a solution of MeOH. Diffraction data were collected at room temperature on an Xcallibur-3 diffractometer with graphite-monochromated Mo Kα radiation (λ = 0.71073 Å) operating in the w-scans mode. The structure was solved by direct methods and refined by the full-matrix least-squares technique in the anisotropic approximation for non-hydrogen atoms using the SHELXTL program package. Crystallographic data for all structures in this paper have been deposited at Cambridge Crystallographic Data Centre. CCDC numbers: 2412358 (for **15a**), 2412360 (for **8b**), 2412357 (for **10b**), 2412359 (for **11b**), and 2412356 (for **44**). Copies of the data can be obtained, free of charge, on application to CCDC, 12 Union Road, Cambridge CB21EZ, UK, (fax: +44-(0)1223-336033 or e-mail: deposit@ccdc.cam.ac.uk).

Compound 15a

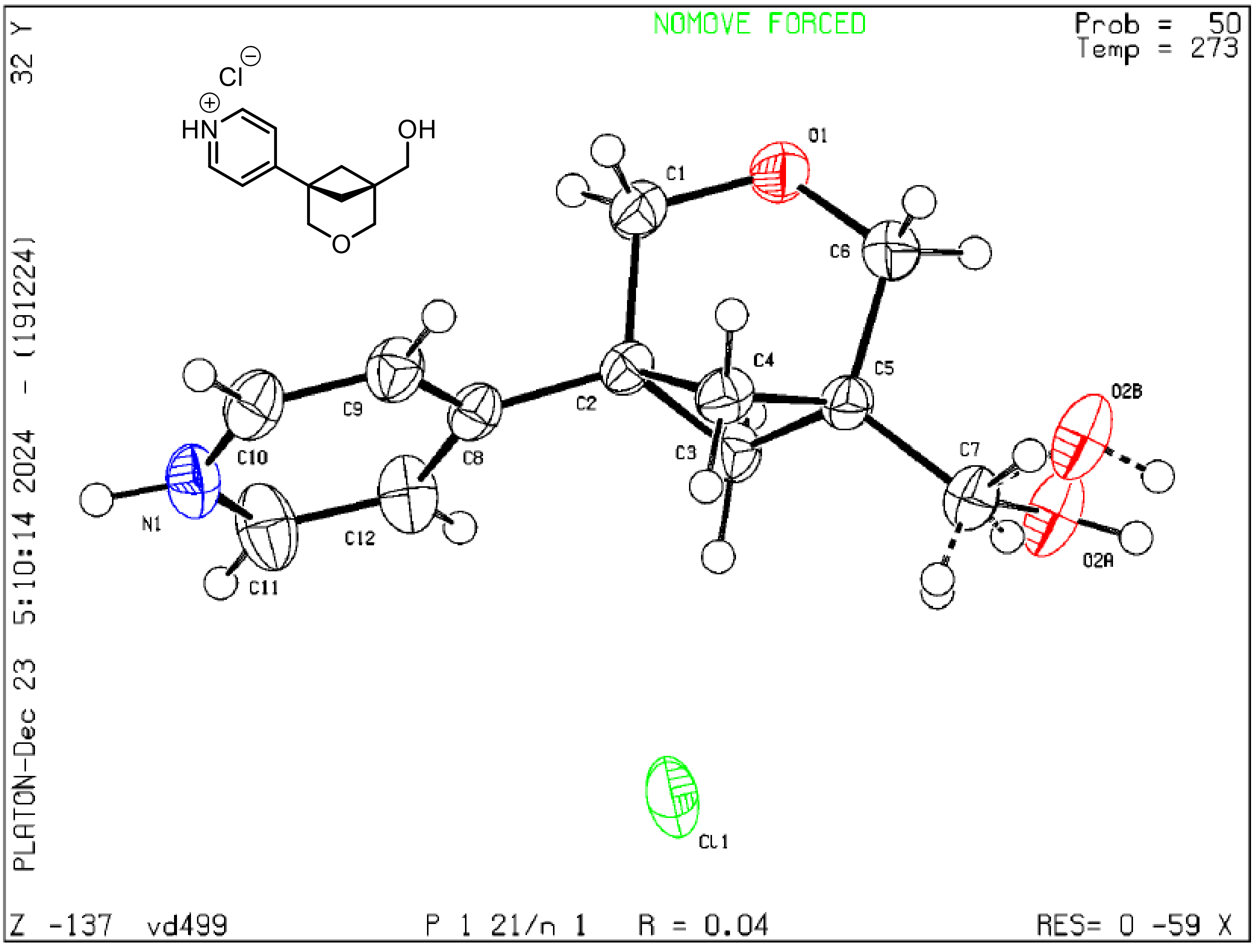

Figure S1. Molecular structure of compound **15a** according to X-ray diffraction data. Thermal ellipsoids are shown at a 50% probability level.

Crystal structure determination of 15a

data_vd499

_chemical_formula_moiety 'Cl, $C_{12}H_{16}NO_2$'

_chemical_formula_sum '$C_{12}H_{16}ClNO_2$'

_chemical_formula_weight 241.71

_space_group_crystal_system 'monoclinic'

_space_group_IT_number 14

_space_group_name_H-M_alt 'P 1 21/n 1'

_space_group_name_Hall '-P 2yn'

_cell_length_a 6.9164(5)

_cell_length_b 16.9799(11)

_cell_length_c 10.4967(7)

_cell_angle_alpha 90

_cell_angle_beta 102.464(2)

_cell_angle_gamma 90

_cell_volume 1203.68(14)

_cell_formula_units_Z 4

_cell_measurement_reflns_used 3400

_cell_measurement_temperature 273.15

_cell_measurement_theta_max 26.26

_cell_measurement_theta_min 2.32

_shelx_estimated_absorpt_T_max 0.973

_shelx_estimated_absorpt_T_min 0.953

_exptl_absorpt_coefficient_mu 0.303

_exptl_absorpt_correction_type none

_exptl_crystal_colour pink

_exptl_crystal_colour_primary pink

_exptl_crystal_density_diffrn 1.334

_exptl_crystal_description block

_exptl_crystal_F_000 512

_exptl_crystal_size_max 0.16

_exptl_crystal_size_mid 0.14

_exptl_crystal_size_min 0.09

_diffrn_reflns_av_R_equivalents 0.0251

_diffrn_reflns_av_unetI/netI 0.0224

_diffrn_reflns_Laue_measured_fraction_full 1.000
_diffrn_reflns_Laue_measured_fraction_max 1.000
_diffrn_reflns_limit_h_max 8
_diffrn_reflns_limit_h_min -5
_diffrn_reflns_limit_k_max 20
_diffrn_reflns_limit_k_min -19
_diffrn_reflns_limit_l_max 8
_diffrn_reflns_limit_l_min -12
_diffrn_reflns_number 8282
_diffrn_reflns_point_group_measured_fraction_full 1.000
_diffrn_reflns_point_group_measured_fraction_max 1.000
_diffrn_reflns_theta_full 24.994
_diffrn_reflns_theta_max 24.994
_diffrn_reflns_theta_min 2.321
_diffrn_ambient_temperature 273.15
_diffrn_measured_fraction_theta_full 1.000
_diffrn_measured_fraction_theta_max 1.000
_diffrn_measurement_device_type 'Bruker APEX-II CCD'
_diffrn_measurement_method '\f and \w scans'
_diffrn_radiation_type MoK\a
_diffrn_radiation_wavelength 0.71073
_diffrn_source_current 30.0
_diffrn_source_power 1.2
_diffrn_source_voltage 40.0
_diffrn_standards_number 0
_reflns_Friedel_coverage 0.000

Compound 8b

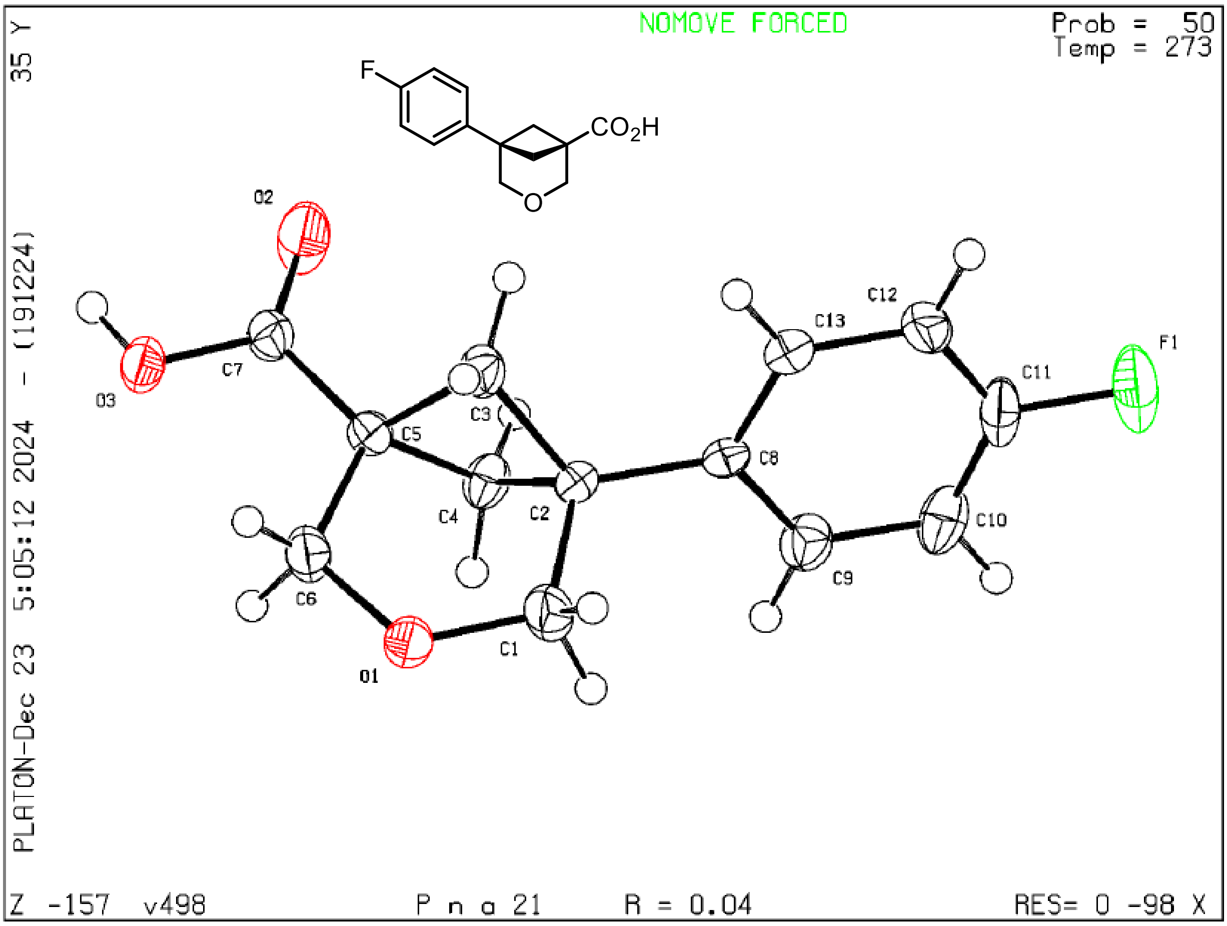

Figure S2. Molecular structure of compound **8b** according to X-ray diffraction data. Thermal ellipsoids are shown at a 50% probability level.

Crystal structure determination of 8b

data_v498

_chemical_formula_moiety	'$C_{13}H_{13}FO_3$'
_chemical_formula_sum	'$C_{13}H_{13}FO_3$'
_chemical_formula_weight	236.23
_cell_length_a	17.6626(13)
_cell_length_b	9.8936(6)
_cell_length_c	6.4581(6)
_cell_angle_alpha	90
_cell_angle_beta	90
_cell_angle_gamma	90
_cell_volume	1128.53(15)
_cell_formula_units_Z	4

_cell_measurement_reflns_used 5804
_cell_measurement_temperature 273.15
_cell_measurement_theta_max 26.31
_cell_measurement_theta_min 2.31
_shelx_estimated_absorpt_T_max 0.987
_shelx_estimated_absorpt_T_min 0.975
_exptl_absorpt_coefficient_mu 0.109
_exptl_absorpt_correction_type none
_exptl_crystal_colour colourless
_exptl_crystal_colour_primary colourless
_exptl_crystal_density_diffrn 1.390
_exptl_crystal_description block
_exptl_crystal_F_000 496
_exptl_crystal_size_max 0.23
_exptl_crystal_size_mid 0.18
_exptl_crystal_size_min 0.12
_diffrn_reflns_av_R_equivalents 0.0599
_diffrn_reflns_av_unetI/netI 0.0335
_diffrn_reflns_Laue_measured_fraction_full 1.000
_diffrn_reflns_Laue_measured_fraction_max 1.000
_diffrn_reflns_limit_h_max 21
_diffrn_reflns_limit_h_min -21
_diffrn_reflns_limit_k_max 11
_diffrn_reflns_limit_k_min -11
_diffrn_reflns_limit_l_max 7
_diffrn_reflns_limit_l_min -7
_diffrn_reflns_number 15426
_diffrn_reflns_point_group_measured_fraction_full 1.000
_diffrn_reflns_point_group_measured_fraction_max 1.000
_diffrn_reflns_theta_full 24.995
_diffrn_reflns_theta_max 24.995
_diffrn_reflns_theta_min 2.306
_diffrn_ambient_temperature 273.15
_diffrn_measured_fraction_theta_full 1.000
_diffrn_measured_fraction_theta_max 1.000

_diffrn_measurement_device_type 'Bruker APEX-II CCD'

_diffrn_measurement_method '\f and \w scans'

_diffrn_radiation_type MoK\a

_diffrn_radiation_wavelength 0.71073

_diffrn_source_current 30.0

_diffrn_source_power 1.2

_diffrn_source_voltage 40.0

_diffrn_standards_number 0

_reflns_Friedel_coverage 0.822

_reflns_Friedel_fraction_full 1.000

_reflns_Friedel_fraction_max 1.000

_reflns_number_gt 1802

_reflns_number_total 1975

Compound 10b

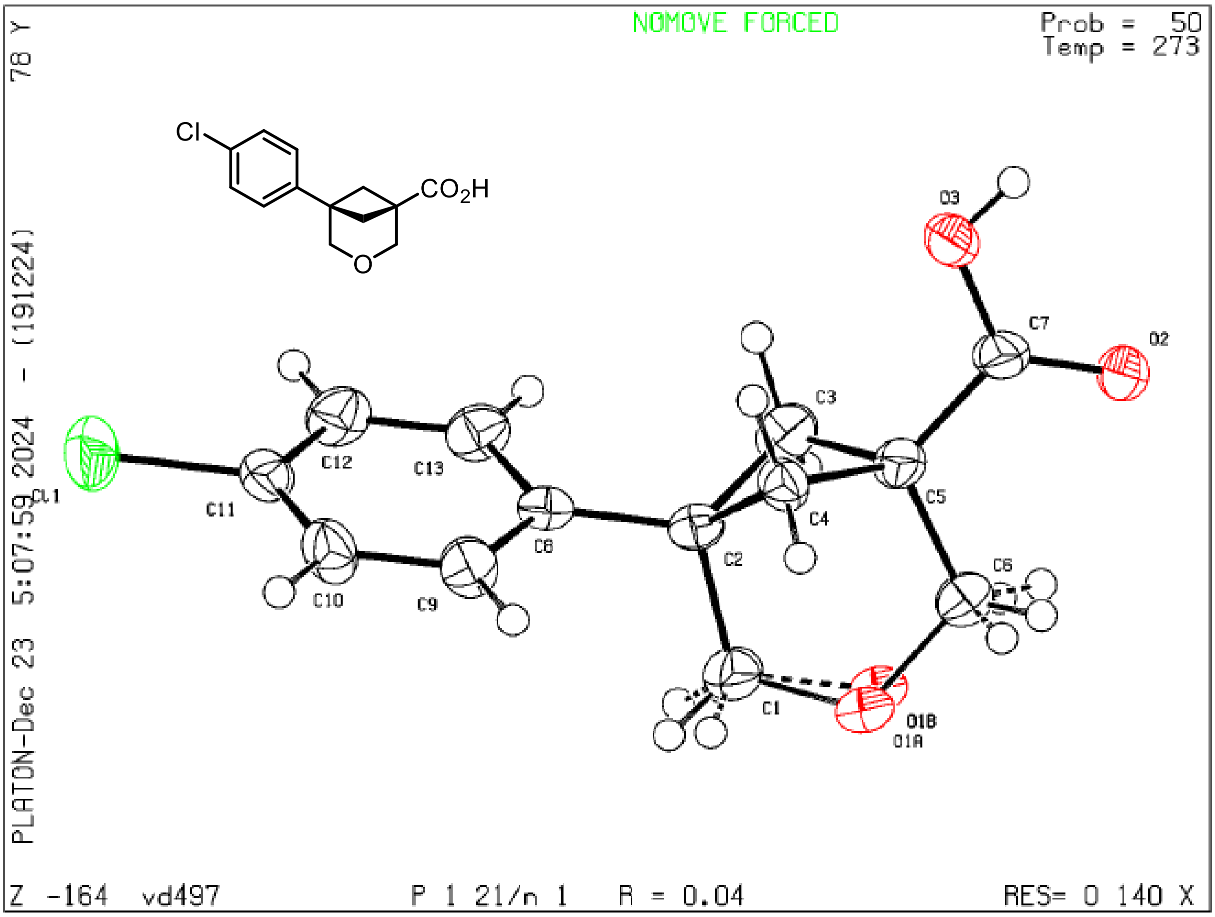

Figure S3. Molecular structure of compound **10b** according to X-ray diffraction data. Thermal ellipsoids are shown at a 50% probability level.

data_vd497

_chemical_formula_moiety	'$C_{13}H_{13}ClO_3$'
_chemical_formula_sum	'$C_{13}H_{13}ClO_3$'
_chemical_formula_weight	252.68
_cell_length_a	9.6105(7)
_cell_length_b	6.5062(5)
_cell_length_c	19.0985(12)
_cell_angle_alpha	90
_cell_angle_beta	92.349(4)
_cell_angle_gamma	90
_cell_volume	1193.18(15)
_cell_formula_units_Z	4
_cell_measurement_reflns_used	4849
_cell_measurement_temperature	273.15

_cell_measurement_theta_max 25.71
_cell_measurement_theta_min 3.31
_shelx_estimated_absorpt_T_max 0.949
_shelx_estimated_absorpt_T_min 0.923
_exptl_absorpt_coefficient_mu 0.313
_exptl_absorpt_correction_type none
_exptl_crystal_colour colourless
_exptl_crystal_colour_primary colourless
_exptl_crystal_density_diffrn 1.407
_exptl_crystal_description block
_exptl_crystal_F_000 528
_exptl_crystal_size_max 0.26
_exptl_crystal_size_mid 0.19
_exptl_crystal_size_min 0.17
_diffrn_reflns_av_R_equivalents 0.0379
_diffrn_reflns_av_unetI/netI 0.0240
_diffrn_reflns_Laue_measured_fraction_full 1.000
_diffrn_reflns_Laue_measured_fraction_max 1.000
_diffrn_reflns_limit_h_max 11
_diffrn_reflns_limit_h_min -11
_diffrn_reflns_limit_k_max 7
_diffrn_reflns_limit_k_min -7
_diffrn_reflns_limit_l_max 22
_diffrn_reflns_limit_l_min -22
_diffrn_reflns_number 15144
_diffrn_reflns_point_group_measured_fraction_full 1.000
_diffrn_reflns_point_group_measured_fraction_max 1.000
_diffrn_reflns_theta_full 24.999
_diffrn_reflns_theta_max 24.999
_diffrn_reflns_theta_min 2.134
_diffrn_ambient_temperature 273.15
_diffrn_measured_fraction_theta_full 1.000
_diffrn_measured_fraction_theta_max 1.000
_diffrn_measurement_device_type 'Bruker APEX-II CCD'
_diffrn_measurement_method '\f and \w scans'

_diffrn_radiation_type MoK\a

_diffrn_radiation_wavelength 0.71073

_diffrn_source_current 30.0

_diffrn_source_power 1.2

_diffrn_source_voltage 40.0

_diffrn_standards_number 0

_reflns_Friedel_coverage 0.000

Compound 11b

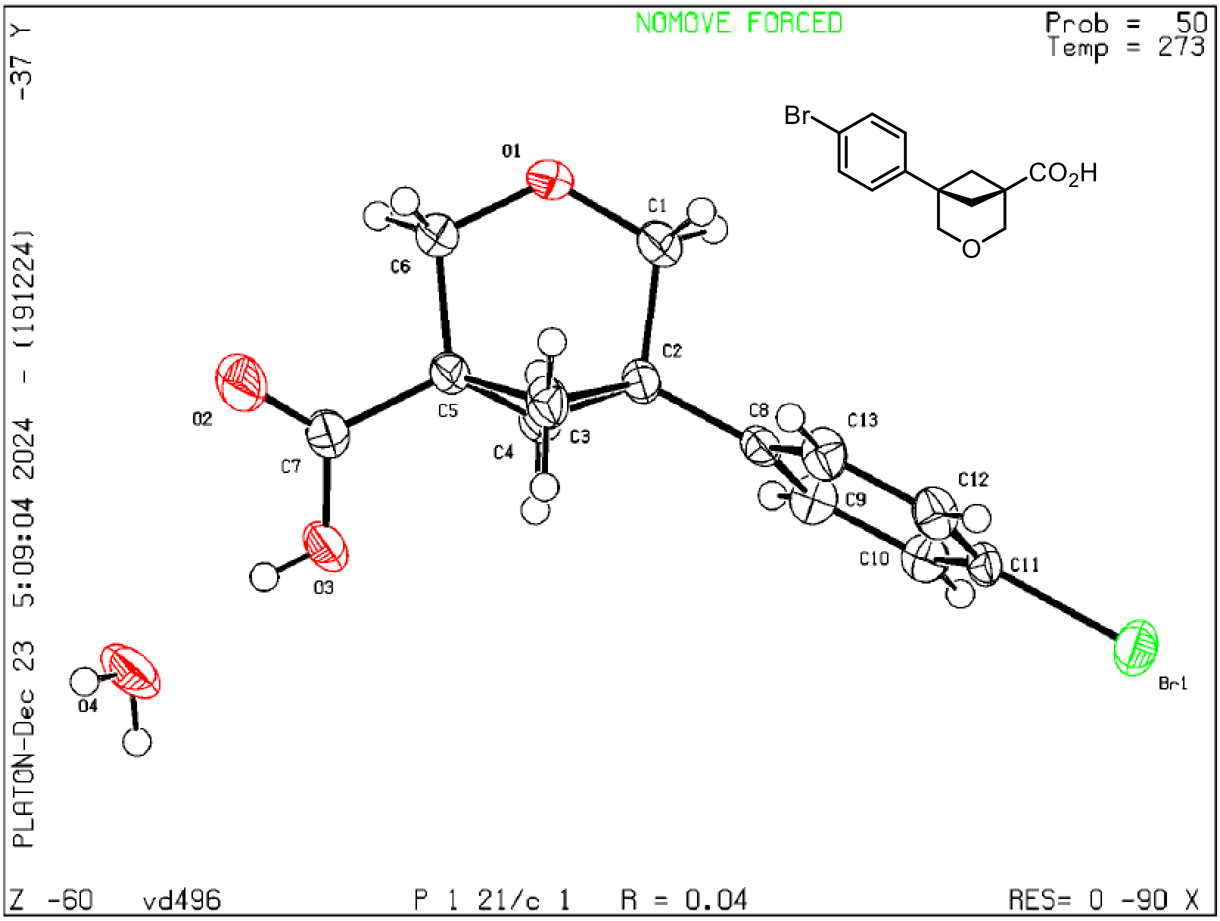

Figure S4. Molecular structure of compound **11b** according to X-ray diffraction data. Thermal ellipsoids are shown at a 50% probability level.

data_vd496

_chemical_formula_moiety	'$C_{13}H_{13}BrO_3$, H_2O'
_chemical_formula_sum	'$C_{13}H_{15}BrO_4$'
_chemical_formula_weight	315.16
_cell_length_a	7.1066(5)
_cell_length_b	5.9043(4)
_cell_length_c	31.480(2)
_cell_angle_alpha	90
_cell_angle_beta	96.210(4)
_cell_angle_gamma	90
_cell_volume	1313.14(16)
_cell_formula_units_Z	4
_cell_measurement_reflns_used	2905
_cell_measurement_temperature	273.15

_cell_measurement_theta_max 25.20
_cell_measurement_theta_min 2.60
_shelx_estimated_absorpt_T_max 0.705
_shelx_estimated_absorpt_T_min 0.508
_exptl_absorpt_coefficient_mu 3.134
_exptl_absorpt_correction_T_max 0.7454
_exptl_absorpt_correction_T_min 0.3282
_exptl_absorpt_correction_type numerical
_exptl_absorpt_process_details SADABS-2014/5
_exptl_crystal_colour colourless
_exptl_crystal_colour_primary colourless
_exptl_crystal_density_diffrn 1.594
_exptl_crystal_description prism
_exptl_crystal_F_000 640
_exptl_crystal_size_max 0.25
_exptl_crystal_size_mid 0.12
_exptl_crystal_size_min 0.12
_diffrn_reflns_av_R_equivalents 0.0525
_diffrn_reflns_av_unetI/netI 0.0519
_diffrn_reflns_Laue_measured_fraction_full 0.999
_diffrn_reflns_Laue_measured_fraction_max 0.999
_diffrn_reflns_limit_h_max 8
_diffrn_reflns_limit_h_min -8
_diffrn_reflns_limit_k_max 7
_diffrn_reflns_limit_k_min -7
_diffrn_reflns_limit_l_max 22
_diffrn_reflns_limit_l_min -37
_diffrn_reflns_number 8836
_diffrn_reflns_point_group_measured_fraction_full 0.999
_diffrn_reflns_point_group_measured_fraction_max 0.999
_diffrn_reflns_theta_full 24.992
_diffrn_reflns_theta_max 24.992
_diffrn_reflns_theta_min 2.603
_diffrn_ambient_temperature 273.15
_diffrn_measured_fraction_theta_full 0.999

_diffrn_measured_fraction_theta_max 0.999

_diffrn_measurement_device_type 'Bruker APEX-II CCD'

_diffrn_measurement_method '\f and \w scans'

_diffrn_radiation_type MoK\a

_diffrn_radiation_wavelength 0.71073

_diffrn_source_current 30.0

_diffrn_source_power 1.2

_diffrn_source_voltage 40.0

_diffrn_standards_number 0

_reflns_Friedel_coverage 0.000

Compound 44

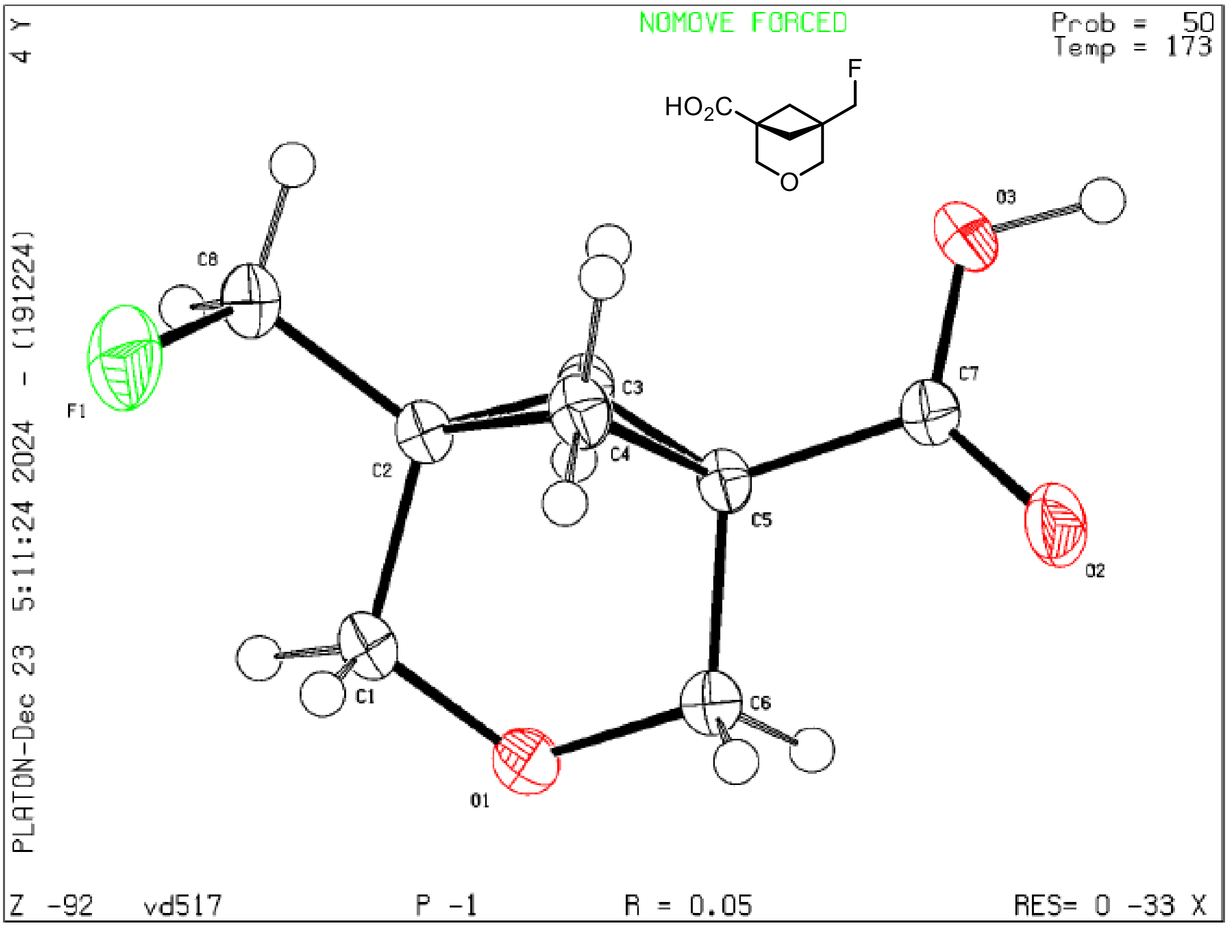

Figure S5. Molecular structure of compound **44** according to X-ray diffraction data. Thermal ellipsoids are shown at a 50% probability level.

data_vd517

_chemical_formula_moiety 'C8H11FO3'

_chemical_formula_sum 'C8H11FO3'

_chemical_formula_weight 174.17

_space_group_crystal_system 'triclinic'

_space_group_IT_number 2

_space_group_name_H-M_alt 'P -1'

_space_group_name_Hall '-P 1'

_cell_length_a 6.1331(3)

_cell_length_b 6.8560(3)

_cell_length_c 9.8371(4)

_cell_angle_alpha 84.537(3)

_cell_angle_beta 88.807(3)

_cell_angle_gamma 77.281(3)
_cell_volume 401.65(3)
_cell_formula_units_Z 2
_cell_measurement_reflns_used 2481
_cell_measurement_temperature 172.65
_cell_measurement_theta_max 30.25
_cell_measurement_theta_min 3.06
_shelx_estimated_absorpt_T_max 0.990
_shelx_estimated_absorpt_T_min 0.976
_exptl_absorpt_coefficient_mu 0.123
_exptl_absorpt_correction_type none
_exptl_crystal_colour colourless
_exptl_crystal_colour_primary colourless
_exptl_crystal_density_diffrn 1.440
_exptl_crystal_description plate
_exptl_crystal_F_000 184
_exptl_crystal_size_max 0.2
_exptl_crystal_size_mid 0.18
_exptl_crystal_size_min 0.08
_diffrn_reflns_av_R_equivalents 0.0282
_diffrn_reflns_av_unetI/netI 0.0274
_diffrn_reflns_Laue_measured_fraction_full 0.997
_diffrn_reflns_Laue_measured_fraction_max 0.995
_diffrn_reflns_limit_h_max 7
_diffrn_reflns_limit_h_min -7
_diffrn_reflns_limit_k_max 8
_diffrn_reflns_limit_k_min -8
_diffrn_reflns_limit_l_max 12
_diffrn_reflns_limit_l_min -12
_diffrn_reflns_number 6135
_diffrn_reflns_point_group_measured_fraction_full 0.997
_diffrn_reflns_point_group_measured_fraction_max 0.995
_diffrn_reflns_theta_full 25.242
_diffrn_reflns_theta_max 27.484
_diffrn_reflns_theta_min 2.080

_diffrn_ambient_temperature 172.65

_diffrn_measured_fraction_theta_full 0.997

_diffrn_measured_fraction_theta_max 0.995

_diffrn_measurement_device_type 'Bruker APEX-II CCD'

_diffrn_measurement_method '\f and \w scans'

_diffrn_radiation_type MoK\a

_diffrn_radiation_wavelength 0.71073

_diffrn_source_current 30.0

_diffrn_source_power 1.2

_diffrn_source_voltage 40.0

_diffrn_standards_number 0

_reflns_Friedel_coverage 0.000

7. Analysis of Aqueous Solubility

Test articles (**EN300-7362818 (Sonidegib)**, **(BCHep-Sonidegib (50)**, and **EN300-45381956 (51))**, and a reference compound (Ondansetron) were assessed for kinetic solubility in phosphate-buffered saline, pH 7.4.

Reagents and consumables

Phosphate buffered saline, pH 7.4 (Sigma-Aldrich, USA; Cat #P3813)
Acetonitrile Chromasolv, gradient grade, for HPLC, ≥99.9% (Sigma-Aldrich, USA; Cat #34851)
Ondansetron base powder (Enamine, Ukraine, Cat # EN300-117273)
DMSO (Sigma-Aldrich, USA; Cat # 34869)
Costar 96 Well Assay Blocks (Corning, USA; Cat # 3958)
MultiScreen HTS 96 Well Filter Plates (Millipore, Ireland; Cat # MSSLBPC10)
UV-Star® 96 Well Microplate (Greiner Bio-One, Germany; Cat #655801)
Matrix Disposable pipette tips (ThermoScientific, USA; Cat ## 8041, 7622, 7321)
Flex-Tubes Microcentrifuge Tubes, 1.5 mL (Eppendorf, Germany; Cat # 22364111)
Matrix Storage tubes, 1.4 mL (ThermoScientific, USA; Cat # 4247)

Equipment

Water purification system Millipore Milli-Q Gradient A10 (Sartorius Arium™ Mini)
Thermomixer R Block, 1.5 mL (Eppendorf, Germany; Cat # 5355)
Matrix Multichannel Electronic Pipette 2-125 µL, 5-250 µL, 15-1250 µL (Thermo Scientific, USA; Cat ## 2011, 2012, 2004)
SpectraMax Paradigm™ Reader (Multi-Mode Detection Platform, Product # 33270-1279)
Multi-Well Plate Vacuum Manifold (Pall Corporation, USA; Product # 5014)
Vacuum pump (Millipore, USA; Model # XX5500000)

Analytical System

The measurements were performed using a SpectraMax Paradigm reader in UV-Vis mode. The data were acquired and analyzed using SoftMax Pro v.5.4 (Molecular Devices) and Excel 2010 data analysis software.

Methods

Kinetic solubility assay was performed according to Enamine's aqueous solubility SOP. Briefly, using a 20 mM stock solution of the compound in 100% DMSO dilutions were prepared to a theoretical concentration of 400 μM in duplicates in phosphate-buffered saline pH 7.4 (138 mM NaCl, 2.7 mM KCl, 10 mM K-phosphate) with 2% final DMSO. The experimental compound dilutions in PBS were further allowed to equilibrate at 25 °C on a thermostatic shaker for two hours and then filtered through HTS filter plates using a vacuum manifold. The filtrates of test compounds were diluted 2-fold with acetonitrile with 2% DMSO before measuring.

In parallel, using a 20 mM stock solution of the compound in 100% DMSO dilutions were prepared to theoretical concentrations of 0 μM (blank), 10 μM, 25 μM, 50 μM, 100 μM, and 200 μM in 50% acetonitrile/PBS with 2% final DMSO to generate calibration curves. Ondansetron was used as a reference compound to control proper assay performance. 200 μL of each sample was transferred to a 96-well plate and measured in the 230-550 nm range with a 5 nm step.

The concentrations of compounds in PBS filtrate are calculated using a dedicated Microsoft Excel calculation script. Proper absorbance wavelengths for calculations are selected for each compound manually based on absorbance maximums (absolute absorbance unit values for the minimum and maximum concentration points within the 0 – 3 OD range). Each final dataset is visually evaluated by the operator, and goodness of fit (R^2) is calculated for each calibration curve. The effective range of this assay is approximately 2-400 μM and the compounds returning values close to the upper limit of the range may have higher actual solubility (e.g. 5'-deoxy-5-fluorouridine).

Results

The solubility data of the test and reference compounds are listed in the tables below. The calibration curves are shown in Appendix*.

Table S2. 1st batch

Compound ID	PBS solubility, pH 7.4, µM			SE
	Incubation 1	Incubation 2	Mean	
Ondansetron	128	126	**127****	1.1
**EN300-7362818 (Sonidegib)**	6	6	**6**	0.2
**BCHep-Sonidegib (50)**	5	3	4	0.9

Table S3. 2nd batch

Compound ID	PBS solubility, pH 7.4, µM			SE
	Incubation 1	Incubation 2	Mean	
Ondansetron	124	124	**124****	0.1
**EN300-45381956 (51)**	34	34	**34**	0.2

*Goodness of fit (R^2) in all titration curves as well as the variations between repeat measurements indicates a high quality of the experimental data in the current batch of test articles.

**Ondansetron solubility data are consistent with those previously obtained.

APPENDIX

1st batch

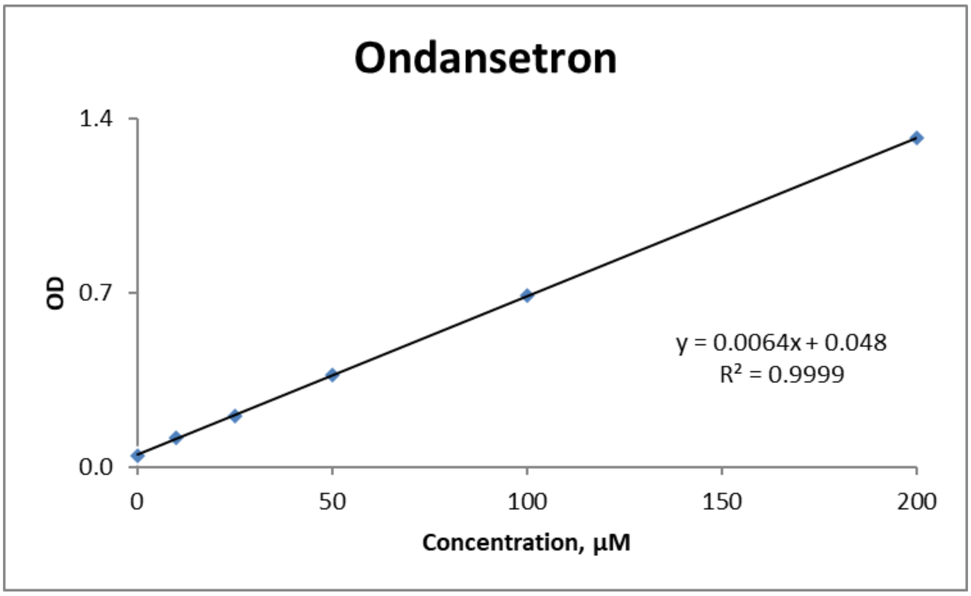

Figure S6. Calibration curve for **Ondansetron**

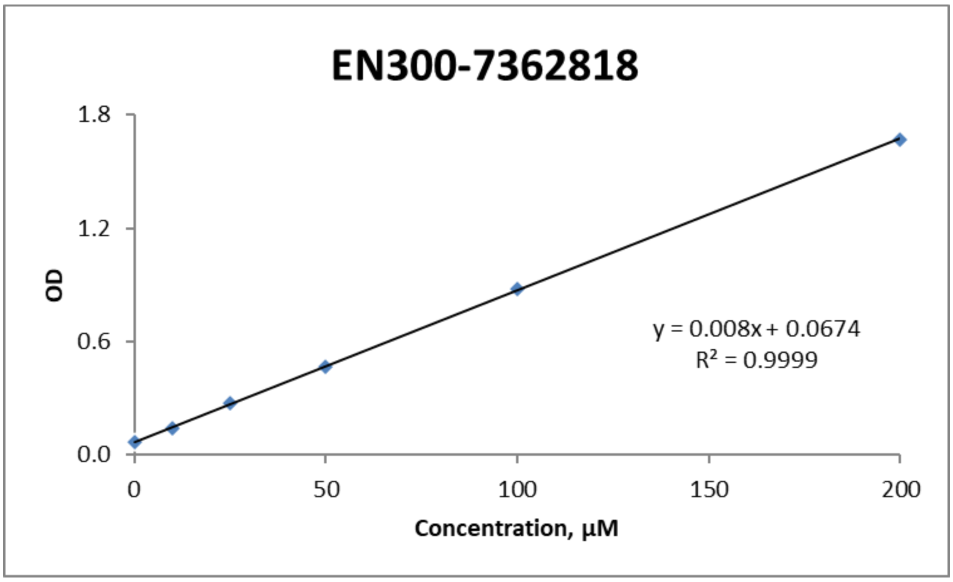

Figure S7. Calibration curve for **EN300-7362818 (Sonidegib)**

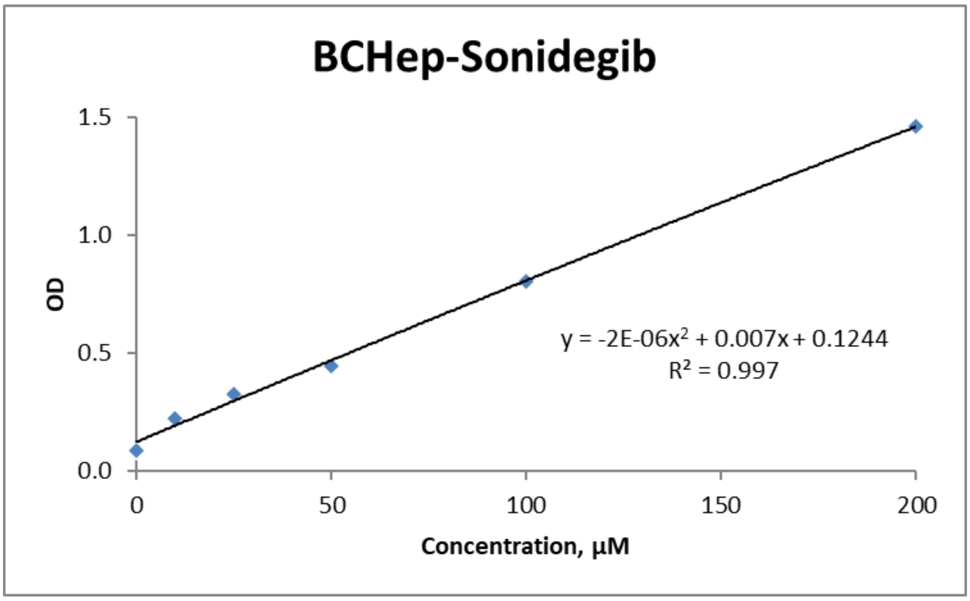

Figure S8. Calibration curve for **BCHep-Sonidegib (50)**

2nd batch

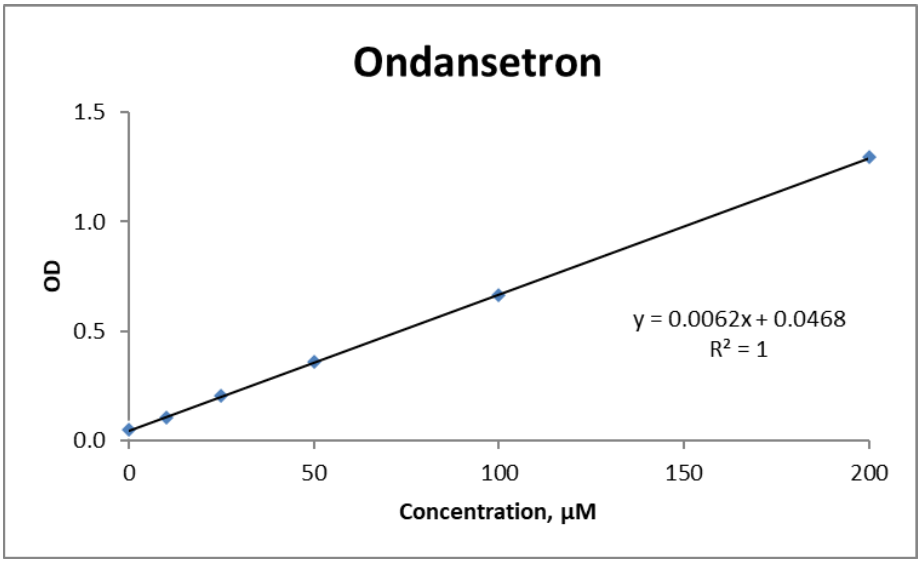

Figure S9. Calibration curve for **Ondansetron**

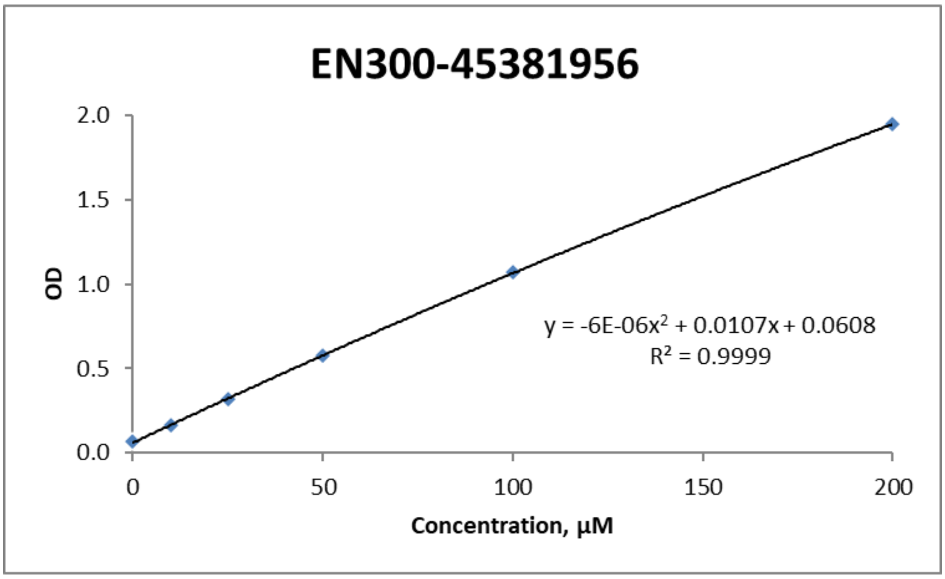

Figure S10. Calibration curve for **EN300-45381956 (51)**

8. Determination of Distribution Coefficient (LogD, pH 7.4)

The aim of this study was to determine distribution coefficients for the test articles **EN300-7362818 (Sonidegib)**, **BCHep-Sonidegib (50)**, **EN300-45381956 (51)**, and reference compound (Mebendazole) in *n*-octanol – phosphate-buffered saline (PBS), pH 7.4. The distribution coefficient (or LogD) is a logarithm of the ratio of drug concentrations in two immiscible solvents, typically pH-buffered water and *n*-octanol. It is a measure of the hydrophobic/hydrophilic properties of a given molecule. The partition of test compounds is determined using a shake-flask method, which involves mixing a certain amount of the solute of interest in defined volumes of *n*-octanol and an aqueous buffer of choice followed by equilibration of the mixture by incubation with efficient mixing. Then, the distribution of the compounds in each solvent was controlled using LC-MS/MS.

Reagents and consumables

DMSO Chromasolv Plus, HPLC grade, ≥99.7% (Sigma-Aldrich, USA; Cat #34869)
Acetonitrile Chromasolv, gradient grade, for HPLC, ≥99.9% (Sigma-Aldrich, USA; Cat #34851)
Formic acid for mass spectrometry, ~98% (Fluka, USA; Cat #94318)
Phosphate buffered saline, tablet (Sigma-Aldrich, USA; Cat # P4417)
1-Octanol ACS grade, ≥99% (Sigma-Aldrich, USA; Cat # 472328)
Mebendazole analytical standard, ≥ 98%, HPLC (Sigma-Aldrich, USA; Cat # M2523)
DMSO stock solutions of the test compounds 10 mM
Phenomenex Luna® C18 HPLC column, 2.0 × 30 mm, 3 μm (Cat #00A-4162-B0)
1.1 mL microtubes in microracks, pipettor tips (Thermo Scientific, USA).
National Scientific MicroTube™ Rack (Thermo Fisher Scientific, USA; Cat # TN094612R)

Equipment

Gradient HPLC system (Shimadzu, Japan)
Hybrid triple quadrupole/linear ion trap mass-detector 4000 QTRAP with Turbo V ion source (AB Sciex, Canada)
VWR Membrane Nitrogen Generators N2-04-L1466, nitrogen purity 99%+ (VWR, USA)
MTR22 Multi Mix Rotator (UNICO, USA)
Laboratory Centrifuge, Sigma 4-15C, Qiagen (SIGMA GmbH, Germany)
Water purification system Millipore Milli-Q Gradient A10 (Millipore, France)
Multichannel Electronic Pipettes 2-125 μL, 5-250 μL, 15-1250 μL, Matrix (Thermo Scientific, USA; Cat ## 2001, 2002, 2004)

Analytical System

All measurements were performed using the Shimadzu Prominence HPLC system including vacuum degasser, gradient pumps, reverse phase column, column oven, and autosampler. The HPLC system was coupled with a hybrid triple quadrupole/linear ion trap mass-detector 4000 QTRAP (PE Sciex). The TurboIonSpray ion source was used in both positive and negative ion modes. Acquisition and analysis of the data were performed using Analyst 1.6.3 software.

Methods

Incubations were carried out in Eppendorf-type polypropylene microtubes in triplicates. A 2.5 µL aliquot of 20 mM DMSO stock of a test compound was added into the previously mutually saturated mixture containing 500 µL of PBS (pH 7.4) and 500 µL of *n*-octanol. The solution was mixed in a rotator for 1 hour at 30 rpm. Phase separation was assured by centrifugation for 2 min at 6000 rpm. The octanol phase was diluted 100-fold with 40% acetonitrile, and the aqueous phase (PBS buffer) was diluted 10-fold, for **BCHep-Sonidegib** and **Mebendazole**, the aqueous phase was analyzed without dilution. The samples (both phases) were analyzed using an HPLC system coupled with a tandem mass spectrometer. Mebendazole was used as a reference compound. Calculations of the partition ratios were carried out using the equation below.

$$D = \frac{d_o \cdot S_O}{d_p \cdot S_P}$$

where: S_0 – peak area of the analyte in *n*-octanol phase

S_P – peak area of the analyte in PBS buffer

d_o – dilution coefficient for *n*-octanol phase

d_p – dilution coefficient for aqueous phase

Results

LogD data for the reference compound (Mebendazole) and test compounds are provided in the tables below.

Table S4. Experimental LogD, pH 7.4, 1st batch

Compound ID	Incuba-tion	S_P	S_O	D	LogD, pH 7.4	
**Mebendazole**	1	3.41E+05	2.80E+06	8.21E+02	2.92	2.9
	2	3.18E+05	2.82E+06	8.87E+02	2.95	
	3	3.56E+05	2.91E+06	8.17E+02	2.91	
**BCHep-Sonidegib (50)**	1	3.51E+03	3.04E+06	8.66E+04	4.94	>=4.5
	2	3.49E+03	2.90E+06	8.31E+04	4.92	
	3	3.55E+03	3.09E+06	8.70E+04	4.94	

*Reliable measurable range is approximately -1 to 4.5.

Table S5. Experimental LogD, pH 7.4, 2nd batch

Compound ID	Incuba-tion	S_P	S_O	D	LogD, pH 7.4	
**Mebendazole**	1	5.98E+03	5.69E+05	9.52E+02	2.98	3.0
	2	5.11E+03	5.81E+05	1.14E+03	3.06	
	3	6.16E+03	5.32E+05	8.63E+02	2.94	
**EN300-7362818 (Sonidegib)**	1	1.45E+03	8.13E+06	5.61E+04	4.75	>=4.5
	2	1.44E+03	8.05E+06	5.60E+04	4.75	
	3	1.22E+03	8.27E+06	6.79E+04	4.83	
**EN300-45381956 (51)**	1	4.44E+03	3.18E+06	7.16E+04	4.86	>=4.5
	2	4.99E+03	3.11E+06	6.24E+04	4.79	
	3	5.06E+03	3.20E+06	6.33E+04	4.80	

*Reliable measurable range is approximately -1 to 4.5.

9. Metabolic Stability in Human Liver Microsomes

The objective of this study was to determine the metabolic stability of 3 test articles (**EN300-7362818 (Sonidegib)**, **BCHep-Sonidegib (50)**, **EN300-45381956 (51)**) and reference compounds in human liver microsomes at five-time points over 40 minutes using HPLC-MS. Metabolic stability is defined as the percentage of parent compound lost over time in the presence of a metabolically active test system.

Reagents and consumables

DMSO Chromasolv Plus, HPLC grade, ≥99.7% (Sigma-Aldrich, USA; Cat# 34869)
Acetonitrile Chromasolv, gradient grade, for HPLC, ≥99.9% (Sigma-Aldrich, USA; Cat# 34851)
Methanol, HiPerSolv, HPLC-gradient grade, ≥99.9% (VWR Chemicals, USA, Cat# 20864.320)
Potassium phosphate monobasic (Bio-Basic, Canada; Lot #N9016010)
Potassium phosphate dibasic (Bio-Basic, Canada; Lot #MA7100050)
Magnesium chloride hexahydrate (Santa Cruz Biotechnology, Inc., USA; sc-203126A)
Human Liver Microsomes: pooled, mixed gender (XenoTech, H0630/lot N#1210097)
Glucose-6-phosphate dehydrogenase from baker's yeast, type XV (Sigma-Aldrich, USA; Cat #G6378)
D-Glucose-6-phosphate sodium salt (Sigma-Aldrich, USA; Cat #G7879-1G)
NADPH tetrasodium salt (BLD Pharmatech Ltd., Cat #BD116582)
Formic acid (Sigma-Aldrich, 94318)
Verapamil hydrochloride (Sigma Aldrich, USA; Cat #V4629)
Niclosamide (Sigma-Aldrich, USA; Cat #N3510)
DMSO stock solutions of the tested compounds 20 mM
(+,-) Propranolol hydrochloride (Sigma-Aldrich, P0884)
Diclofenac, 96% purity (Enamine, #EN300-119509)
Phenomenex Luna® C18 HPLC column, 2.1 × 50 mm, 5 µm (Cat #5291-126)
Phenomenex Luna® C18 HPLC column, 2 × 30 mm, 5 µm (S.N. 146953-2)
Matrix™ 0.75 mL blank tubes (Cat #4170), pipettor tips (Thermo Scientific).

Equipment

Gradient HPLC system (Shimadzu)
Triple quadrupole mass-detector API 3000 with TurboIonSpray Ion Source (AB Sciex, Canada)
Nitrogen generator N2-04-L1466, nitrogen purity 99%+ (Whatman)
Environmental Incubator Shaker G24; Digital Refrigerated Incubator/Shaker Innova 4330 (New Brunswick Scientific)

Water purification system Millipore Milli-Q Gradient A10 (Millipore, France)
Multichannel pipettors 1-30 μL, 2-125 μL, 30-850 μL (Thermo Scientific)

Analytical System

All measurements were performed using the Shimadzu HPLC system including vacuum degasser, gradient pumps, reverse phase HPLC column, column oven, and autosampler. Mass spectrometric analysis was performed using an API 3000 mass spectrometer from Applied Biosystems/MDS Sciex (AB Sciex) with Turbo V ion source and TurboIonspray interface. The TurboIonSpray ion source was used in both positive and negative ion modes. The data acquisition and system control were performed using Analyst 1.6.3 software from AB Sciex.

Methods

Microsomal incubations were carried out in 96-well plates in 5 aliquots of 30 μL each (one for each time point). Liver microsomal incubation medium comprised of phosphate buffer (100 mM, pH 7.4), $MgCl_2$ (3.3 mM), NADPH (3 mM), glucose-6-phosphate (5.3 mM), glucose-6-phosphate dehydrogenase (0.67 units/mL) with 0.42 mg of liver microsomal protein per mL. In the control reactions, the NADPH-cofactor system was substituted with phosphate buffer. Test compounds (2 μM, final solvent concentration 1.6 %) were incubated with microsomes at 37 °C, shaking at 100 rpm. Five-time points over 40 minutes were analyzed. The reactions were stopped by adding 5 volumes of acetonitrile with internal standard to incubation aliquots, followed by protein sedimentation by centrifuging at 5500 rpm for 5 min. Each reaction was performed in duplicates. Supernatants were analyzed using the HPLC system coupled with a tandem mass spectrometer.

The elimination constant (k_{el}), half-life ($t_{1/2}$), and intrinsic clearance (Cl_{int}) were determined in a plot of ln(AUC) versus time, using linear regression analysis:[1]

$$k_{el} = -slope \qquad t_{1/2} = \frac{0.693}{k} \qquad Cl_{\text{int}} = \frac{0.693}{t_{1/2}} \times \frac{\mu l_{incubation}}{mg_{microsomes}}$$

[1] In order to indicate the quality of the linear regression analysis, the R^2 (determination coefficient) values are provided. In some cases, the last time point is excluded from the calculations to ensure acceptable logarithmic linearity of decay.

Results

Human microsomal stability data for reference and test compounds are provided in the tables below.

Table S6. Human microsomal stability, 1st batch

Compound ID	Time, min	Peak Area Ratio Inc. 1	Peak Area Ratio Inc. 2	Peak Area Ratio, Mean of 2	% Remaining Mean of 2	R^2	k_{el}, min^{-1}	$t_{1/2}$, min	Cl_{int}, µL/min/mg	% Remaining without cofactor, Mean of 2
1	2	3	4	5	6	7	8	9	10	11
Diclofenac human	0	3.52E-01	3.41E-01	3.47E-01	100	0.995	0.118	5.9	285	100
	7	1.69E-01	1.51E-01	1.60E-01	46	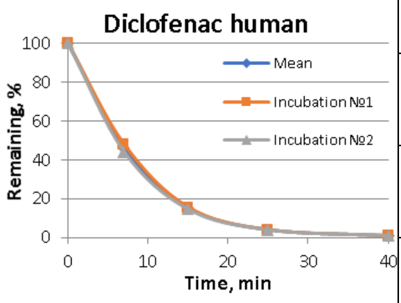				
	15	5.51E-02	5.02E-02	5.27E-02	15					
	25	1.42E-02	1.41E-02	1.41E-02	4					
	40	3.10E-03	3.64E-03	3.37E-03	1					95
Propranolol human	0	4.45E-02	4.69E-02	4.57E-02	100	0.892	0.009	81.4	21	100
	7	4.34E-02	4.83E-02	4.59E-02	100	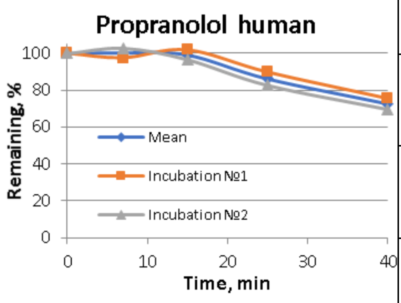				
	15	4.53E-02	4.54E-02	4.54E-02	99					
	25	4.00E-02	3.89E-02	3.94E-02	86					
	40	3.37E-02	3.27E-02	3.32E-02	73					107
BCHep-Sonidegib (50) human	0	1.27E+01	1.23E+01	1.25E+01	100	0.837	0.006*	120.2*	14*	100
	7	1.21E+01	1.08E+01	1.15E+01	92	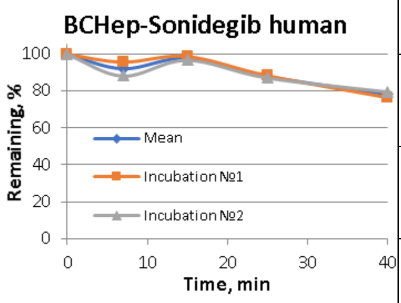				
	15	1.25E+01	1.19E+01	1.22E+01	98					
	25	1.12E+01	1.07E+01	1.10E+01	88					
	40	9.69E+00	9.77E+00	9.73E+00	78					93

*Parameter should be considered as approximate due to the high stability of the compound.

Table S7. Human microsomal stability, 2nd batch

Compound ID	Time, min	Analyte Peak Area		Analyte Peak Area, Mean of 2	% Remaining Mean of 2	R	k_{el} , min^{-1}	$t_{1/2}$, min	Cl_{int}, µL/min/mg	% Remaining without cofactor, Mean of 2
		Inc. 1	Inc. 2							
1	2	3	4	5	6	7	8	9	10	11
**Diclofenac human**	0	1.05E-01	1.41E-01	1.23E-01	100	1.000	0.092	7.5	223	100
	7	6.80E-02	7.01E-02	6.91E-02	56	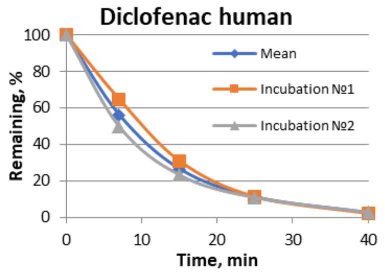				
	15	3.25E-02	3.26E-02	3.26E-02	26					
	25	1.20E-02	1.52E-02	1.36E-02	11					
	40	2.34E-03	3.79E-03	3.07E-03	2					86
**Propranolol human**	0	5.22E-02	4.99E-02	5.11E-02	100	0.902	0.008	86.8	19	100
	7	4.48E-02	4.60E-02	4.54E-02	89	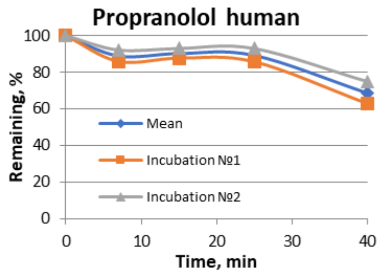				
	15	4.57E-02	4.64E-02	4.61E-02	90					
	25	4.47E-02	4.64E-02	4.56E-02	89					
	40	3.27E-02	3.75E-02	3.51E-02	69					104
	0	9.02E-01	9.23E-01	9.13E-01	100	0.978	0.007	103.6	16	100
**EN300-7362818 (Sonidegib) human**	**7**	9.04E-01	9.18E-01	9.11E-01	100	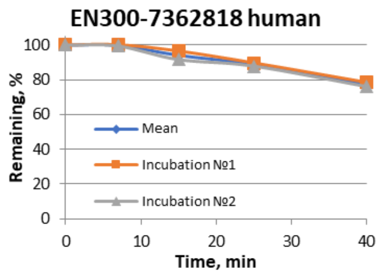				
	15	8.71E-01	8.46E-01	8.59E-01	94					
	25	8.09E-01	8.11E-01	8.10E-01	89					
	40	7.07E-01	7.02E-01	7.05E-01	77					103

**EN300-45381956 (51) human**	0	4.13E-01	3.96E-01	4.05E-01	100	0.993	0.011	60.5	28	100
	7	4.18E-01	3.61E-01	3.90E-01	96	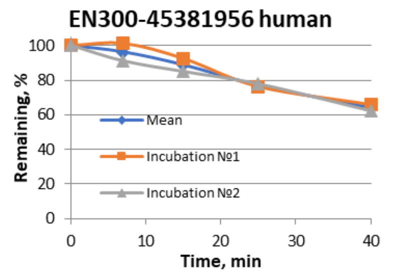				
	15	3.82E-01	3.37E-01	3.60E-01	89					
	25	3.15E-01	3.09E-01	3.12E-01	77					
	40	2.72E-01	2.48E-01	2.60E-01	64					104

*Parameter should be considered as approximate due to the high stability of the compound.

Interpretation of microsomal stability assay data

The test compounds can be classified in terms of their microsomal stability into low, medium and high clearance groups. Intrinsic Clearance (*in vitro*) can be recalculated to Intrinsic Clearance (*in vivo*) using literature data for liver weight and liver blood flow with the next equation[S1]:

$$Predicted\ in\ vivo\ CL_{int,u} = \frac{in\ vitro\ CL_{int} \times PBSF \times LW}{f_{u\,mic}\ or\ f_{u\,heps}}$$

where,

$in\ vivo\ CL_{int,u}$ – predicted *in vivo* intrinsic clearance, mL/min/kg

$in\ vitro\ CL_{int}$ – *in vitro* microsomal clearance, mL/min/mg

$PBSF$ – physiologically based scaling factor – the microsomal average recovery factor for microsomal predictions and hepatocellularity for hepatocyte predictions, mg/g

LW – liver weight/kg bodyweight, g/kg

$f_{u\,mic}\ or\ f_{u\,heps}$ – fraction unbound in either microsomes or hepatocytes (can be determined from Plasma Protein Binding study or assumed as 1, if it is unknown)

Using $in\ vivo\ Cl_{int}$ hepatic clearance can be predicted based on a "well-stirred" liver model using the next formula[S2]:

$$CL_H = \frac{Q_H \times f_u \times CL_{int}}{Q_H + f_u \times CL_{int}}$$

where,

CL_H – predicted hepatic clearance, mL/min/kg

Q_H – liver blood flow, mL/min/kg

f_u – fraction unbound in the blood

CL_{int} – predicted *in vivo* clearance, mL/min/kg

The CL*int* classification values were calculated for mouse, rat, and human species using the literature data on liver weight[S3] and microsomal protein concentration[S3,4] and are represented in the following Table S8.

Table S8. The intrinsic clearance groups for the classification of test compounds

Classification group	Intrinsic clearance (μL/min/mg protein)		
	Mouse	Rat	Human
**Low clearance**	< 8.6	< 13	< 8.8
**High clearance**	> 48	> 72	> 48

10. Assessment of Caco-2 Permeability of Sonidegib and its Saturated Analog 51

Study Objective

The purpose of this study was to evaluate the permeability of compounds **EN300-7362818 (Sonidegib)** and its saturated analog **EN300-45381956 (analog 51)** in the bidirectional Caco-2 assay, including the identification of P-glycoprotein substrate (Pgp-mediated transport).

Reagents and consumables

DMEM/High glucose with L-Glutamine (HyClone, USA; Cat# SH30003.04)
Fetal Bovine Serum (Sigma-Aldrich, USA; Cat# F7524)
Sodium bicarbonate (Sigma-Aldrich, USA; Cat# S5761)
Penicillin/Streptomycin (100x) (Sigma-Aldrich, USA; Cat# P4333)
Puromycin dihydrochloride, ≥98% (Santa Cruz, USA; Cat# sc-108071)
Trypsin-EDTA solution (10x) (Sigma-Aldrich, USA; Cat# T4174)
HEPES, High Purity Grade (Helicon, Am-0485-0.1)
Dulbecco's PBS (1x) without Ca & Mg (Gibco, Cat# 21600-044)
Hanks' BSS (1x) without Ca & Mg without Phenol Red (Sigma, Cat# H4891)
Multiwell Insert system, PET, 1 μm (Millicell, USA; Cat# PSRP010R5)
24 well plate Multiwell™ (Millicell, USA; Cat# PSMW010R5)
Centrifuge Tubes, 50 mL (Santa Cruz, USA; Cat# sc-200251)
Serological Pipettes 5 mL, 10 mL, 25 mL (Greiner Bio-One)
Disposable pipettor tips (Thermo Scientific, Fisherbrand, Eppendorf USA)
1.4 mL microtubes (Thermo Scientific, USA, Cat# 4140)
Verapamil hydrochloride (Sigma-Aldrich, USA; Cat# V4629)
Ketoprofen (Enamine, Ukraine; cat# EN300-120644)
Atenolol, analytical reference material, ≥98.5% (HPLC) (Sigma-Aldrich, USA; Cat# 74827)
Quinidine (Sigma-Aldrich, USA; Cat# Q3625)
DMSO Chromasolv Plus, HPLC grade, ≥99.7% (Sigma-Aldrich, USA; Cat# 34869)
Acetonitrile Chromasolv, gradient grade, for HPLC, ≥99.9% (Sigma-Aldrich, USA; Cat# 34851)
Formic acid for mass spectrometry, ~98% (Fluka, USA; Cat# 94318)
Methanol, for HPLC, ≥99.9% (Sigma-Aldrich, Cat# 34860)
Heptafluorobutyric acid (Sigma-Aldrich, Cat# 52411)

Equipment

Cell culture CO_2 incubator, model CCL-170B-8 (ESCO, Singapore)
Centrifuge 5804R (Eppendorf, USA)

Centrifuge 4-15C (Qiagen) (Sigma, Germany)

Etched Hemacytometer, dark line counting chamber (Hausser Scientific, USA; Cat# 3500)

Innova 4080 Incubator Shaker (New Brunswick Scientific, USA)

Millicell-ERS system ohm meter (Millipore, Cat# MERS 000 01)

Phenomenex Luna® C18 HPLC column, 2.1 × 50 mm, 5 μm (Cat #5291-126)

VWR Membrane Nitrogen Generators N2-04-L1466, nitrogen purity 99%+ (VWR, USA)

Multichannel manual pipette (Thermo Labsystems Finnpipette, FA16-50R)

Multichannel Electronic Pipettes 2-125 μL, 5-250 μL, 15-1250 μL, Matrix (Thermo Scientific, USA)

PIPETMAN pipettes 2-20 μL, 50-200 μL, 200-1000 μL (Gilson, USA)

Water purification system Millipore Milli-Q Gradient A10 (Millipore, France)

Opentrons OT-2 (Opentrons Labworks, USA)

Analytical System

All measurements were performed using the Shimadzu HPLC system including vacuum degasser, gradient pumps, reverse phase HPLC column, column oven, and autosampler. Mass spectrometric analysis was performed using a Triple quadrupole mass-detector API 3000 with TurboIonSpray Ion Source (AB Sciex, Canada) and TurboIonspray interface. The TurboIonSpray ion source was used in both positive and negative ion modes. The data acquisition and system control were performed using Analyst 1.6.3 software from AB Sciex.

Methods

Caco-2 cells were cultured in 75 cm^2 flasks to 80-90% confluence according to the ATCC and Millipore recommendations[S5] in a humidified atmosphere at 37 °C and 5% CO_2. Cells were detached with Trypsin/EDTA solution and resuspended in the complete medium containing DMEM high glucose (4500 mg/l) with L-glutamine (4 mM) supplemented with 10% heat-inactivated Fetal Bovine Serum, 1% non-essential amino acids, and 730 nM puromycin and seeded at a density 5×10^5 cells in 75 cm^2 flask[S10] After 5 days, cells were trypsinized and resuspended in the complete medium to a final concentration of 600×10^3 cells/mL. 400 μL of the cell suspension was added to each well of the HTS 24-Multiwell Insert System and 25 ml of prewarmed complete medium was added to the feeder tray. Caco-2 cells were incubated in Multiwell Insert System for 6-10 days before the transport experiments. The medium in the filter plate and feeder tray was refreshed every other day. Prior to the transport experiment, the integrity of the monolayer was verified by measuring the transepithelial electrical resistance (TEER) for every well using the Millicell-ERS system ohm meter. The final TEER values were within the range of 150-600 $\Omega \times cm^2$ as required for

the assay conditions.[S6-9] The 24-well insert plate was removed from its feeder plate and placed in a new sterile 24-well transport analysis plate. The inserts were washed with PBS after medium aspiration.

Ketoprofen, Atenolol, and Quinidine were used as reference compounds.

To determine the rate of compounds transport in apical (A)-to-basolateral (B) direction, 300 μL of the test compound dissolved in transport buffer (Hanks' BSS (9.5 g/L) and $NaHCO_3$ (0.35 g/L) with $MgSO_4$ to final concentration 0.81 mM, $CaCl_2$ to final concentration 1.26 mM, HEPES to final concentration 25 mM. pH adjusted to 7.4) was added into the filter wells; 1000 μL of transport buffer was added to transport analysis plate wells.

To determine transport rates in the basolateral (B)-to-apical (A) direction, 1000 μL of the test compound solutions was added into the wells of the transport analysis plate, the wells in the filter plate were filled with 300 μL of buffer (apical compartment).

The effect of the inhibitor on the P-gp-mediated transport of the tested compounds was assessed by determining the bidirectional transport in the presence or absence of verapamil. The Caco-2 cells were preincubated for 30 min at 37 °C with 100 μM of verapamil in both apical and basolateral compartments. After removal of the preincubation medium, the test compounds with verapamil (100 μM) in transport buffer were added to donor wells, while the receiver wells were filled with the appropriate volume of transport buffer with 100 μM of verapamil.

The final amount of test and reference compounds were 10 μM.

The plates were incubated for 90 min at 37 °C under continuous shaking at 100 rpm. 75 μL aliquots were taken from the donor and receiver compartments for LC-MS/MS analysis. All samples were mixed with 2 volumes of acetonitrile followed by protein sedimentation by centrifuging at 10000 rpm for 10 min. Supernatants were analyzed using the HPLC system coupled with a tandem mass spectrometer.

All solutions of test and reference compounds were prepared manually, and further manipulations with the solutions were performed with automation using Opentrons.

The apparent permeability (P_{app}) was calculated for the Caco-2 permeability assay using the following equation[S9,11]:

$$\boldsymbol{P_{app}} = \frac{\boldsymbol{V_A}}{\boldsymbol{Area} \times \boldsymbol{Time}} \times \frac{[\boldsymbol{drug}]_{\boldsymbol{acc}}}{[\boldsymbol{drug}]_{\boldsymbol{initial,d}}}$$

$\boldsymbol{V_A}$ – volume of transport buffer in acceptor well,

Area – surface area of the insert (equals to the effective growth area of the insert - 0.7 sq.cm),

Time – time of the assay,

$\boldsymbol{[drug]_{acc}}$ – amount of test compound in acceptor well,

$\boldsymbol{[drug]_{initial,d}}$ – initial amount of test compound in a donor well.

$\boldsymbol{P_{app}}$ is expressed in 10^{-6}cm/sec.

Efflux ratio (P_{app}(BA)/P_{app}(AB)) reveals the difference in P_{app} as a result of active transport. If the efflux ratio is greater than 2, this indicates the occurred active efflux.

To identify the P-gp substrate, the P-gp inhibitor verapamil was added to the incubation medium. A decrease of the efflux ratio in the presence of verapamil indicates that the compound is a P-gp substrate.

The % recovery can be useful in interpreting the Caco-2 data. If the recovery is very low, this may indicate poor solubility, binding of the compound to the test plate materials, metabolism by the Caco-2 cells, or accumulation of the compound in the cell monolayer. The %recovery was calculated using the following equation:

$$\%\,\boldsymbol{recovery} = \frac{\boldsymbol{C_{acc} \times V_{acc} + C_d \times V_d}}{\boldsymbol{C_{initial,d} \times V_d}} \times \mathbf{100},$$

$\boldsymbol{V_{acc}}$ – volume of compound solution in acceptor well (cm^3),

$\boldsymbol{V_d}$ – volume of compound solution in donor well (cm^3),

$\boldsymbol{C_{acc}}$ – peak area of the test compound in acceptor well,

$\boldsymbol{C_d}$ **–** peak area of the test compound in donor well,

$\boldsymbol{C_{initial,d}}$ – initial peak area of the test compound in a donor well.

Results and discussion

A-B and B-A permeability data as well as the efflux ratio (P_{app}B-A/P_{app}A-B) data for the test and reference compounds are listed in the tables below.

Table S9. A-B and B-A permeability data

Test compound	P_{app} (AB), 10^{-6} cm/s				P_{app} (BA), 10^{-6} cm/s				Efflux ratio
	**1**	**2**	**Mean**	**SD**	**1**	**2**	**Mean**	**SD**	
Atenolol	0.4	0.5	**0.4**	0.1					
Ketoprofen	19.8	23.8	**21.8**	2.8	19.5	17.9	**18.7**	1.1	**0.9**
Quinidine	14.2	14.3	**14.2**	0.1	34.4	26.2	**30.3**	5.8	**2.1**
EN300-7362818 **Sonidegib**	2.9	2.7	**2.8**	0.1	1.0	0.9	**0.9**	0.1	**0.3**
EN300-45381956 **analog 51**	6.3	4.5	**5.4**	1.3	7.3	8.7	**8.0**	1.0	**1.5**

*Efflux ratio is expressed as the quotient of P_{app}(BA) to P_{app}(AB).

A-B permeability data for all the reference compounds correspond to the literature data.[S5-12]

Table S10. Date of A-B and B-A permeability in the presence of Verapamil

Test compound	P_{app} (AB), 10^{-6} cm/s				P_{app} (BA), 10^{-6} cm/s				Efflux ratio
	**1**	**2**	**Mean**	**SD**	**1**	**2**	**Mean**	**SD**	
Quinidine	23.4	33.5	**28.5**	7.1	20.1	21.6	**20.9**	1.0	**0.7**
EN300-7362818 **Sonidegib**	0.2	2.6	**1.4**	1.7	0.9	1.0	**0.9**	0.1	**0.6**
EN300-45381956 **analog 51**	9.6	8.4	**9.0**	0.9	13.2	10.5	**11.8**	2.0	**1.3**

*Efflux ratio is expressed as the quotient of P_{app} (BA) to P_{app} (AB).

The efflux ratio for Quinidine in the presence or absence of Verapamil corresponds to the literature data thus validating this assay.

Table S11. Recovery values for test and reference compounds

Compound ID	Recovery, %											
	A-B			B-A			A-B in presence of Verapamil			B-A in presence of Verapamil		
	1	2	Mean	1	2	Mean	1	2	Mean	1	2	Mean
Ketoprofen	96	106	**101**	93	95	**94**						
Atenolol	122	111	**117**									
Quinidine	87	98	**92**	96	98	**97**	95	103	**99**	84	89	**87**
EN300-7362818 **Sonidegib**	35	37	**36**	46	43	**45**	67	63	**65**	63	60	**61**
EN300-45381956 **analog 51**	44	43	**43**	71	69	**70**	53	49	**51**	69	86	**77**

Conclusions

All test articles under the condition of the experiment exhibit moderate permeability in A-B direction and do not undergo active efflux.

It may also be noted that the recovery value (Table S11) for all compounds is low in some cases. This might be due to the poor solubility of the compound in transport solution, metabolism by the Caco-2 cells, and accumulation of the compound in the cell monolayer or binding to the test plate materials. Therefore, the permeability values for the test compounds should be considered approximate.

11. Analysis of novel Hedgehog signaling pathway inhibitors in the cell-based Gli-Luc reporter system

Activation of the hedgehog signaling pathway is important for embryonic development and tissue regeneration. Its misregulation in adults is involved in the pathogenesis of various types of tumors. Currently, Smo is the most druggable target for the development of small molecule inhibitors to this pathway. Sonidegib (LDE-225) is one of such inhibitors, FDA-approved for use in cancers. Resistance to this agent can occur due to adaptive mutations in SMO. Additionally, adverse reactions of this compound include elevated creatine kinase, muscle spasms, musculoskeletal pain, myalgia, diarrhea, abdominal pain, vomiting dysgeusia, alopecia, pruritus, nausea, decreased weight, and others.[S12,13] Optimization of Sonidegib's structure may improve its characteristics. Previously, Sonidegib demonstrated IC50 from 0.6 to 8 nM (depending on the activator Ag1.5 concentration) on the TM3Hh12 (TM3-Gli-Luc reporter) cell line.[S14]

BCH-Sonidegib (**50**) produced an effect with higher IC50 (616 ± 131 nM) compared to the control compound **Sonidegib** (6.4 ± 0.5 nM). The data are presented as mean ± SD from 2 independent experiments.

Illustrations

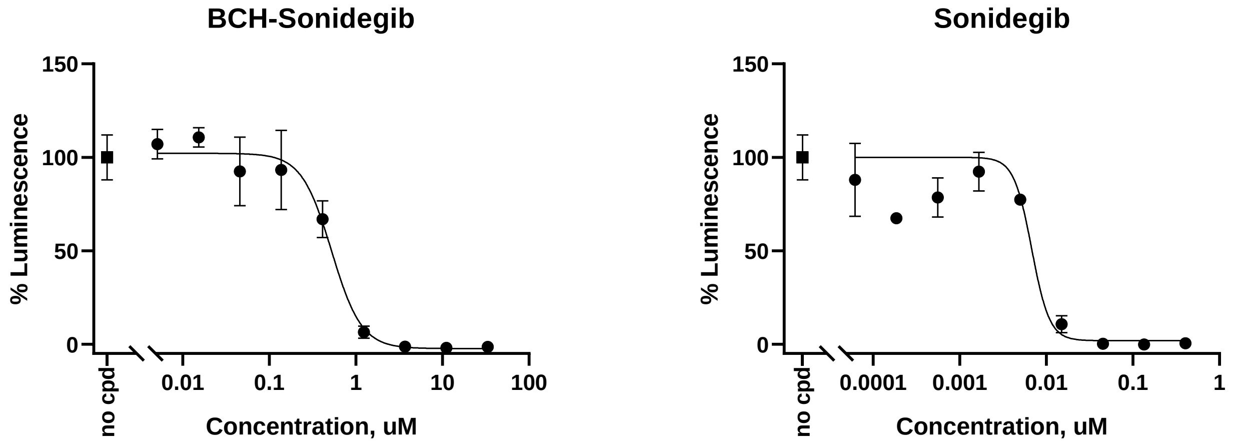

Figure S10. Inhibition of the Hedgehog signaling pathway by Sonidegib and its analog, BCH-Sonidegib (**50**). The intensity of luminescence correlates with the pathway activation. The activation was considered 100% without inhibiting substances (no cpd) and 0% without activator. The data are presented as mean (n = 3) ± SD.

EN300-45381956 (**51**) IC50 = 0.096 uM, pIC50 = 7.0.

The tested compounds produced an effect with higher IC50 compared to the control compound Sonidegib. The reference compound Sonidegib demonstrated IC50 1.462 nM (pIC50 8.8).

Illustrations

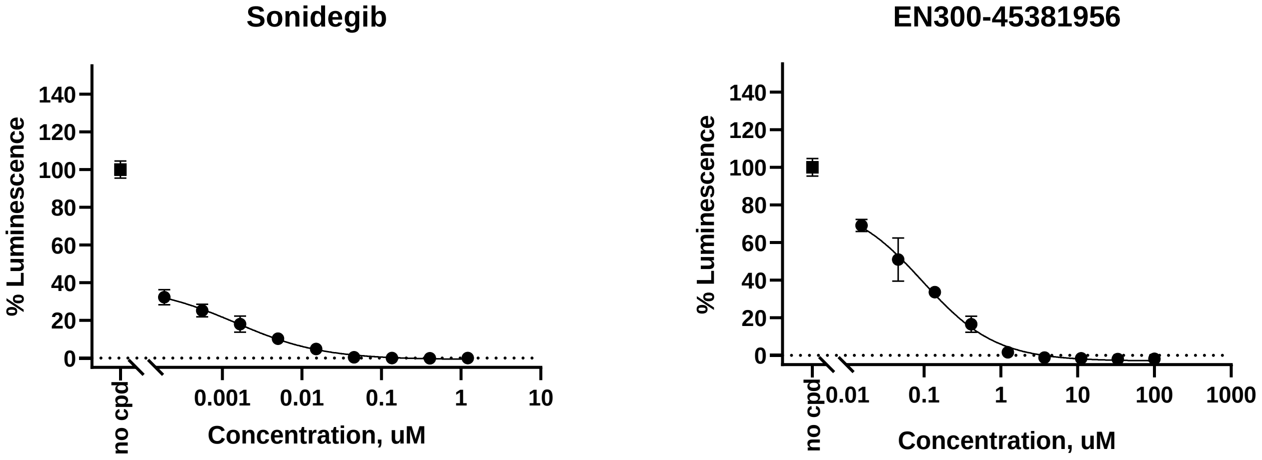

Figure S11. Inhibition of the Hedgehog signaling pathway by **Sonidegib** and EN300-45381956 (**51**). The intensity of luminescence correlates with the pathway activation. The activation was considered 100% without inhibiting substances (no cpd) and 0% without activator. The data are presented as mean (n = 3) ± SD.

Methods

For investigation of the compound's inhibition of the Hedgehog signaling pathway, Gli Reporter – NIH3T3 Cell Line (BPS Bioscience, #60409) was thawed, cultured, and used for the assay according to the vendor's instruction. Cells were seeded into white clear-bottom 96-well microplates at a density of 25.000 cells per well and incubated at 37 ºC in a CO_2 incubator overnight. The next day cells reached confluency. The medium was carefully removed and compound DMSO (0.5%) solution was added for 1 h. Then mouse Sonic Hedgehog (Shh) Recombinant Protein (Cell Signaling, #51772) at a final concentration of 1.9 ug/mL was added for the pathway activation. Plates were incubated at 37 °C in a CO_2 incubator for 24 h. Luciferase assay was performed using the ONE-Step™ Luciferase Assay System (BPS Bioscience, #60690-1) according to the manufacturer's instruction. ONE-Step™ Luciferase reagent was added to the wells and then plates were gently rocked on a platform shaker at room temperature for 20 min, avoiding exposure to excessive heat or light during incubation. After that luminescence was measured. All

compound concentrations were tested in triplicates. Sonidegib (LDE225 (phosphate), Cayman Chemical, #16263) was used as a reference compound. IC50 values were determined by nonlinear regression of the GLI-driven luciferase luminescence vs log10 (concentration) of test compounds using GraphPad Prism 8. Additionally, pIC50 values were calculated.

12. References

[S1] F. L. Wood, J. B. Houston, D. Hallifax. Clearance prediction methodology needs fundamental improvement: Trends common to rat and human hepatocytes/microsomes and implications for experimental methodology. *Drug Metab. Dispos.* **2017**, *45*, 1178-1188.

[S2] T. Laveé, C. Funk. In vivo absorption, distribution, metabolism, and excretion studies in Discovery and Development. *Comprehensive Medicinal Chemistry II*, **2007**, 31.

[S3] Z. E. Barter, M. K. Bayliss, P. H. Beaune, A. R. Boobis, D. J. Carlile, R. J. Edwards, J. B. Houston, B. G. Lake, J. C. Lipscomb, O. R. Pelkonen, G. T. Tucke, A. Rostami-Hodjegan. Scaling factors for the extrapolation of *in vivo* metabolic drug clearance from *in vitro* data: reaching a consensus on values of human microsomal protein and hepatocellularity per gram of liver. *Curr. Drug Metab.* **2007**, *8*, 33-45.

[S4] T. Iwatsubo, H. Suzuki, Y. Sugiyama. Prediction of species differences (rats, dogs, humans) in the *in vivo* metabolic clearance of YM796 by the liver from *in vitro* data. *J. Pharmacol. Exp. Ther.* **1997**, *283*, 462-469.

[S5] A. Arena, J. Phillips. Optimization of Caco-2 cell growth and differentiation for drug transport assay studies using a 96 well MultiScreen Caco-2 Assay System. Millipore protocol note PC1060EN00P, rev. *08*/**2003**.

[S6] B. Srinivasan, A. R. Kolli, M. B. Esch, H. E. Abaci, M. L. Shuler, J. J. Hickman. TEER measurement techniques for *in vitro* barrier model systems. *J. Lab. Autom.* **2015**, *20*, 107-126.

[S7] J. Rautio, J. E. Humphreys, L. O. Webster, A. Balakrishnan, J. P. Keogh, J. R. Kunta, C. J. Serabjit-Singh, J. W. Polli. In vitro, *p*-glycoprotein inhibition assays for assessment of clinical drug interaction potential of new drug candidates: a recommendation for probe substrates. *Drug Metab. Dispos*. **2006**, *34*, 786-792.

[S8] http://www.cyprotex.com/admepk/in-vitro-permeability/caco-2-permeability

[S9] Y. Shirasaka, T. Sakane, S. Yamashita. Effect of P-Glycoprotein Expression Levels on the Concentration-Dependent Permeability of Drugs to the Cell Membrane. *J. Pharm. Sci.* **2008**, 97, 553-565.

[S10] E. Sevin, L. Dehouck, A. Fabulas-da Costa, R. Cecchelli, M. P. Dehouck, S. Lundquist, M. Culot. Accelerated Caco-2 cell permeability model for drug discovery. *J. Pharmacol. Toxicol. Methods.* **2013**, *68*, 334-339.

[S11] Z. S. Teksin, P. R. Seo, J. E. Poli. Comparison of Drug Permeabilities and BCS Classification: Three Lipid-Component PAMPA System Method *versus* Caco-2 Monolayers. *AAPS J.* **2010**, *12*, 238-241.

[S12] R. L. Carpenter, H. Ray. Safety and Tolerability of Sonic Hedgehog Pathway Inhibitors in Cancer. *Drug Saf.* **2019**, *42*, *2*, 263.

[S13] J. Zhang, Z. Liu, J. Jia. Mechanisms of Smoothened Regulation in Hedgehog Signaling. *Cells* **2021**, *10*, 2138.

[S14] S. Pan, X. Wu, J. Jiang, W. Gao, Y. Wan, D. Cheng, D. Han, J. Liu, N. P. Englund, Y. Wang, S. Peukert, K. Miller-Moslin, J. Yuan, R. Guo, M. Matsumoto, A. Vattay, Y. Jiang, J. Tsao, F. Sun, A. M. C. Pferdekamper, S. Dodd, T. Tuntland, W. Maniara, J. F. Kelleher III, Y. Yao, M. Warmuth, J. Williams, M. Dorsch. Discovery of NVP-LDE225, a Potent and Selective Smoothened Antagonist. *CS Med. Chem. Lett.* **2010**, *1*, 130-134.
